# Inference on dengue epidemics with Bayesian regime switching models

**DOI:** 10.1371/journal.pcbi.1007839

**Published:** 2020-05-01

**Authors:** Jue Tao Lim, Borame Sue Dickens, Sun Haoyang, Ng Lee Ching, Alex R. Cook

**Affiliations:** 1 Saw Swee Hock School of Public Health, National University of Singapore and National University Health System, Singapore; 2 Environmental Health Institute, National Environmental Agency, Singapore; University of Zurich, SWITZERLAND

## Abstract

Dengue, a mosquito-borne infectious disease caused by the dengue viruses, is present in many parts of the tropical and subtropical regions of the world. All four serotypes of dengue viruses are endemic in Singapore, an equatorial city-state. Frequent outbreaks occur, sometimes leading to national epidemics. However, few studies have attempted to characterize breakpoints which precede large rises in dengue case counts. In this paper, Bayesian regime switching (BRS) models were employed to infer epidemic and endemic regimes of dengue transmissions, each containing regime specific autoregressive processes which drive the growth and decline of dengue cases, estimated using a custom built multi-move Gibbs sampling algorithm. Posterior predictive checks indicate that BRS replicates temporal trends in Dengue transmissions well and nowcast accuracy assessed using a post-hoc classification scheme showed that BRS classification accuracy is robust even under limited data with the AUC-ROC at 0.935. LASSO-based regression and bootstrapping was used to account for plausibly high dimensions of climatic factors affecting Dengue transmissions, which was then estimated using cross-validation to conduct statistical inference on long-run climatic effects on the estimated regimes. BRS estimates epidemic and endemic regimes of dengue in Singapore which are characterized by persistence across time, lasting an average of 20 weeks and 66 weeks respectively, with a low probability of transitioning away from their regimes. Climate analysis with LASSO indicates that long-run climatic effects up to 20 weeks ago do not differentiate epidemic and endemic regimes. Lastly, by fitting BRS to simulated disease data generated from a stochastic Susceptible-Infected-Recovered model, mechanistic links between infectivity and regimes classified using BRS were provided. The model proposed could be applied to other localities and diseases under minimal data requirements where transmission counts over time are collected.

## Author summary

Dengue, a mosquito-borne infectious disease caused by the dengue viruses, is present in many parts of the tropical and subtropical regions of the world. All four serotypes of dengue viruses are endemic in Singapore, an equatorial city-state. Frequent outbreaks occur, sometimes leading to national epidemics. However, few studies have attempted to characterize breakpoints which precede large rises in dengue case counts. In this paper, Bayesian regime switching (BRS) models were employed to infer epidemic and endemic regimes of dengue transmissions, each containing regime specific processes which drive the growth and decline of dengue cases, estimated using a custom built multi-move Gibbs sampling algorithm. Assessments against various baseline showed that BRS performs better in characterizing dengue transmissions. The dengue regimes estimated by BRS are characterized by their persistent nature. Next, climate analysis showed no short nor long term associations between classified regimes with climate. Lastly, fitting BRS to simulated disease data generated from a mechanistic model, we showed links between disease infectivity and regimes classified using BRS. The model proposed could be applied to other localities and diseases under minimal data requirements where transmission counts over time are collected.

## Introduction

An estimated 390 million dengue infections occur annually creating considerable health and economic burdens [1]. Dengue is widespread across South-East Asian countries and is classified as hyper-endemic due to all four serotypes being in active circulation [2]. Widespread ongoing urbanization and greater host movement rates via both domestic and international travel have increased transmission, particularly across highly connected cities such as Singapore. With favorable climatic conditions, a large daily influx of travelers and high population density, the conditions for dengue transmission are ideal, as reflected in national case counts being non-zero for every week in the past 10 years.

Vector control remains the primary control method for dengue, of the two dengue mosquito vectors *Aedes aegypti* and *Aedes albopictus* in Singapore [3]. The low seroprevalence rates across the national population make the implementation of vaccination using Dengvaxia (CYD-TDV) challenging, therefore techniques such as Wolbachia, fogging and breeding site reduction are utilized to both prevent and control epidemics [4]. The successful application of these methods in epidemics depends on the correct timing for control ramp up in which house inspections increase, community engagement campaigns are rolled out to generate awareness in breeding site reduction and fogging in areas deemed at high risk of transmission [5].The characterization of dengue transmission dynamics through time is therefore critical. Finite resources for ramp up of vector control measures also beg the question of estimating the duration and severity of epidemics in different climates.

Compartmental models and statistical models such as time series estimation and machine learning can characterize dengue transmission dynamics. Compartmental models model infection as a function of separate compartments, and the evolution of the epidemic may be described by ordinary differential equations [6]. However, parameter estimation and inputting initial values for compartmental models often only estimate an epidemic curve, but usually cannot explicitly model different endemic and epidemic dynamics [6]. Statistical frameworks often characterize time series through autoregressive integrated moving average (ARIMA) modelling or machine learning. ARIMA and machine learning both explain the current realization of infectious disease dynamics by its past observations and past exogenous variables. ARIMA type models have been widely used to fit dengue time serieses [7] due to the ease of interpretability. Lagged climatic variables have been assessed to affect dengue and influenza transmission counts linearly in temperate climates [8], but the signal for climatic variables on dengue transmission counts has been found to be weak in tropical climates [9]. A combined approach using time series subsceptible-infected-recovered (TSIR) models takes into account the evolution of population across time as well as autoregressive disease dynamics, was first introduced by Finkenstädt and Grenfell for measles [10]. It was then further developed for modelling multi-strain diseases such as dengue by including cross-immunity dynamics [11–13]^,^. A limiting factor of these models is that they cannot account for the plausibly nonlinear and time varying structure of infectious disease transmission across time. Calibration of multi-strain TSIR models also require virological surveillance data, which may not be always available. Machine learning tools such as random forest and least squares shrinkage operator (LASSO) have been proven to outperform ARIMA type models in predictive metrics such as root mean square error for H5N1 [14] and the ROC in classifying dengue outbreaks respectively [9]. However, these tools have difficulties in inferring the variables driving infectious disease transmission counts and do not have standard confidence intervals to determine model compatibility with data. The variable importance factor calculated for ensemble methods such as random forest remains only asymptotically valid and may not be useful for small count infectious disease time series data [15].

Regime switching models are used to model phenomena in which time series are characterized by characteristic changes in behavior [16]. They originated from econometrics to account for the changes in behavior in macroeconomic variables such as inflation and debt [17] and they have potential applicability in modelling disease states and transmission due to the differential behavior of disease transmissions in epidemic and endemic periods [18]. Martinez et al. explored influenza epidemic detection in Spain using a regime switching framework where highly seasonal dynamics of infection allowed distinct classification of epidemic and endemic disease states [19]. The framework used could detect periods and behavior of high influenza transmission counts and low influenza transmission counts. However, regime switching models have yet to be applied for transmission dynamics which are highly non-seasonal, irregular and persistent such as dengue within tropical climates [2,20].

This paper therefore explores the utility of regime switching models to investigate the dynamic signature of dengue within Singapore. We aim to classify the irregularity in epidemic lengths, estimate the different dynamics in dengue transmission across the different regimes and examine whether climate characterizes the estimated regimes. First, Bayesian Autoregressive (BAR) models of various lags derived using Markov chain Monte Carlo (MCMC) estimate dengue transmissions across time as a benchmark model to explain dengue transmission counts. Next, we utilized Bayesian fixed transition probability regime switching models (BRS) to account for the endemic-epidemic structure of dengue while allowing autoregressive parameters to vary in separate regimes. Model explanatory power was assessed with the mean-absolute percentage error, log Bayes factor, relative deviance information criterion, as well as the predictive power of the BRS by ex-ante classification accuracy of regimes. Next, we estimate the influence of climate on dengue transmission behavior by using the classified regimes from BRS as a dependent variable to climate with the least absolute shrinkage operator (LASSO) with logistic link using area under the receiver operator characteristic (AUC-ROC) as a tuning criterion. The LASSO was subject to nonparametric bootstrapping to recover confidence intervals for inference of climatic variables on the classified regimes. Lastly, using simulated data generated from a stochastic Susceptible-Infected-Recovered model, we provide possible mechanistic links between infectivity and regimes classified using BRS.

## Results

We fitted BAR and BRS models for up to 2/3 lagged differenced dengue case counts, with an additional BAR 3 specification containing climatic variables. (Tables 1 and 2, S1 Appendix) Convergence was achieved on Gibbs sampling the posterior of BAR and BRS parameters (S2 Appendix). Testing univariate MCMC samples across parameters also indicates convergence at the 0.05 level with the Geweke diagnostic test (S1 Appendix). Residual autocorrelation was adequately accounted for by BRS and BAR models, but with autocorrelation on around the 1^st^ to 5^th^ lags exceeding the 95% confidence interval for the BAR-2/3 Lag and BRS-2 Lag models (S2 Appendix). We use the BRS model with 3 lags for parsimony and its ability to account for residual autocorrelation across both regimes. The BRS-3 Lag model performed marginally better on fitting the time series with 5.43% mean absolute percentage error (MAPE) compared to 5.61% and 5.55% on the BAR 2 and 3 Lag model respectively. (Tables 1 and 2) Regime switching models also characterized the likelihood of change in Dengue transmissions better with the Bayes factor and relative DIC highly favoring regime switching models over autoregressive models (Tables 1 and 2).

**Table 1:** Coefficients of AR(2) and 2 Regime AR(2) models.

	BAR-2 Lag^1^		BRS-2 Lag–Regime 1 (Endemic)^2^		BRS-2 Lag-Regime 2 (Epidemic)	
Coefficients	Posterior Mean	95% Credible Interval	Posterior Mean	95% Credible Interval	Posterior Mean	95% Credible Interval
Lag 1	-0.47	(-0.114, 0.051)	-0.291	(-0.404, -0.174)	0.012	(-0.134, 0.161)
Lag 2	0.12	(0.051, 0.187)	0.011	(-0.1, 0.118)	0.136	(-0.016, 0.285)
MAPE	5.61%		3.11%		13.33%	
MAPE (Aggregate)			5.43%			
Bayes Factor			906			
Relative DIC			-592			
Average Regime Length			60 Weeks	(36.55, 69.00)	19 Weeks	(11.06, 24.00)
Regime AUC-ROC			0.927			

**Table 2:** Coefficients of AR(3) and 2 Regime AR(3) models.

	BAR-3 Lag		BRS-3 Lag–Regime 1 (Endemic)		BRS-3 Lag–Regime 2 (Epidemic)	
Coefficients	Posterior Mean	95% Credible Interval	Posterior Mean	95% Credible Interval	Posterior Mean	95% Credible Interval
Lag 1	-0.057	(-0.123, 0.012)	-0.289	(-0.403, -0.173)	0.007	(-0.144, 0.161)
Lag 2	0.124	(0.057, 0.193)	0.055	(-0.056, 0.162)	0.132	(-0.021, 0.287)
Lag 3	0.086	(0.016, 0.153)	0.136	(0.039, 0.231)	0.058	(-0.101, 0.216)
MAPE	5.55%		3.10%		13.40%	
MAPE (Aggregate)			5.41%			
Bayes Factor			631			
Relative DIC			-649			
Average Regime Length			66 Weeks	(36.30, 67.45)	20 Weeks	(10.63, 27.70)
Regime AUC-ROC			0.935			

^1^ Autoregressive models coefficients were estimated using Gibbs samplings, with the dependent variable being change in dengue cases, and independent variables being the first 2/3 lags of the change in dengue cases.^2^ Regime switching models were estimated using Gibbs sampling, with the dependent variable being change in dengue cases, and independent variables being the first 2/3 lags of the change in dengue cases.

**Fig 1:**
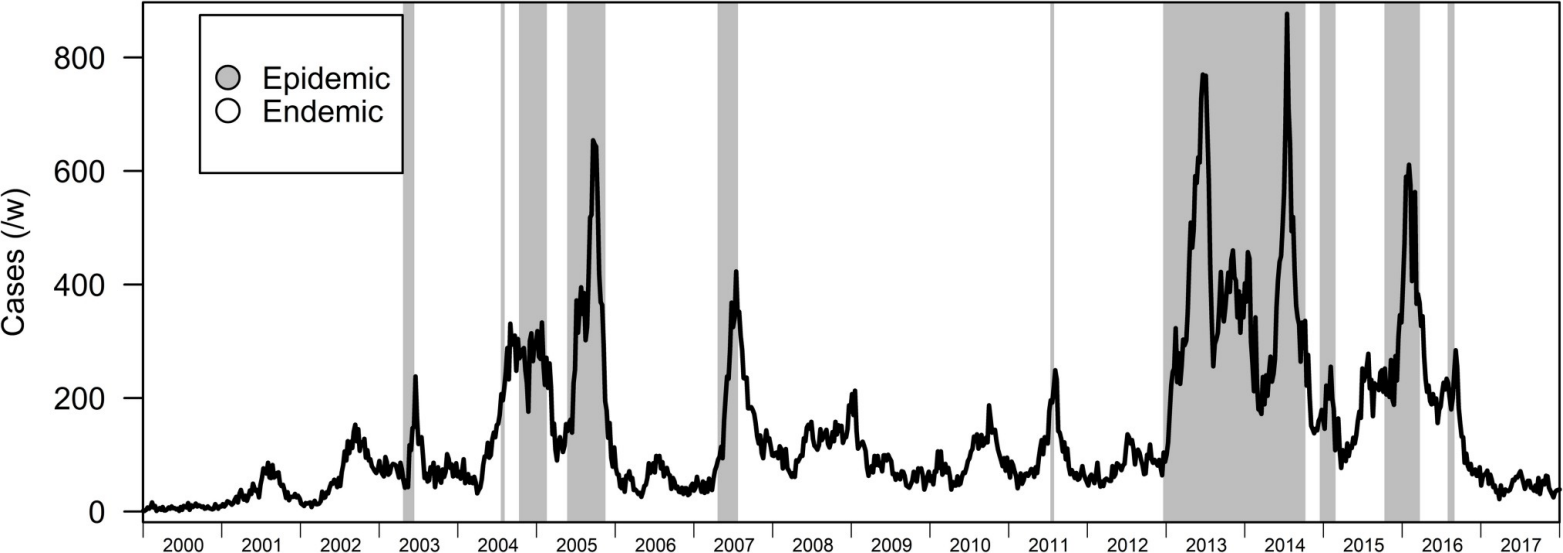
Illustration of regimes to dengue case count data. Highlighted portions indicate fitted regimes of the BRS-3 Lag to dengue counts from 2000–2017.

**Fig 2:**
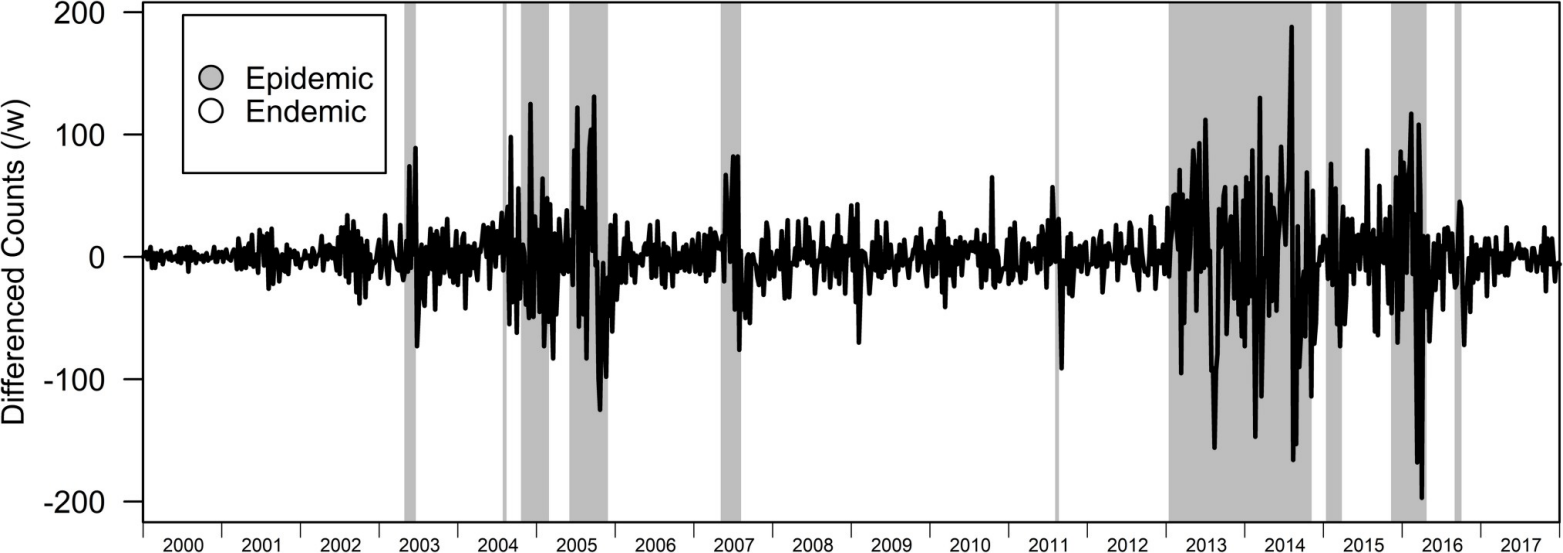
Fit of regimes to dengue case incidence. Highlighted portions indicate fitted regimes of the BRS-3 Lag to dengue incidence from 2000–2017.

**Fig 3:**
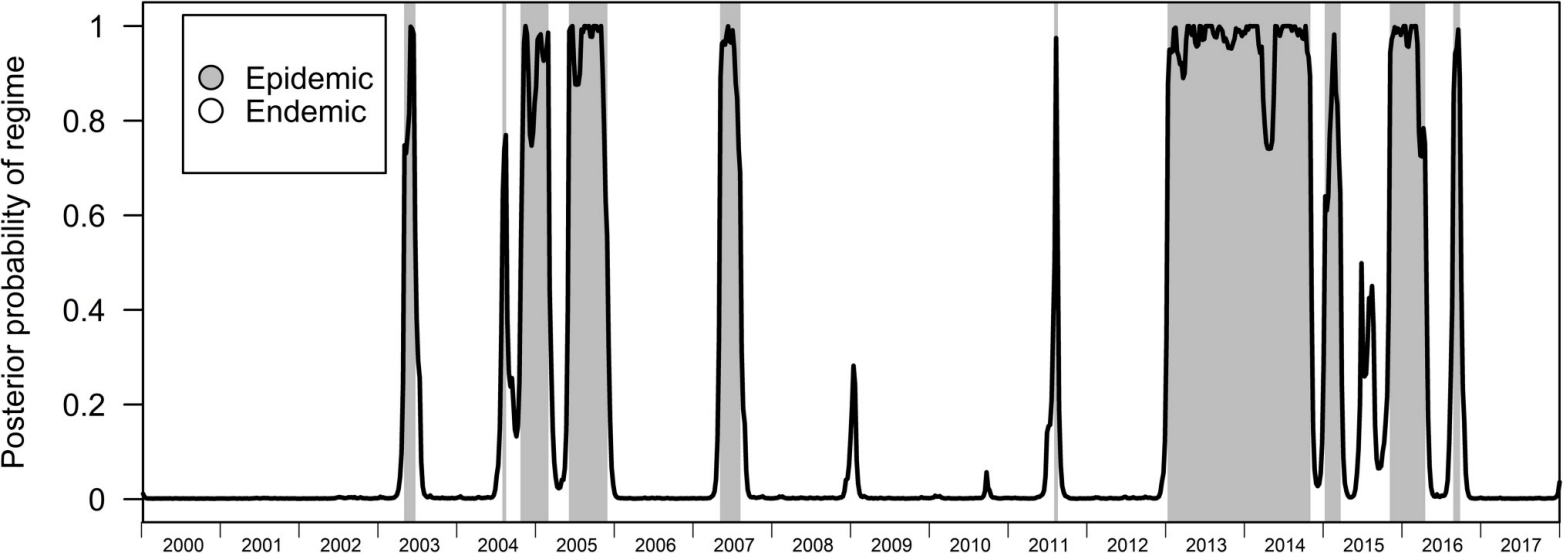
Posterior smoothed probabilities for the epidemic regime. Highlighted portions indicate regimes of the BRS-3 Lag to their corresponding posterior probabilities from 2000–2017.

**Fig 4:**
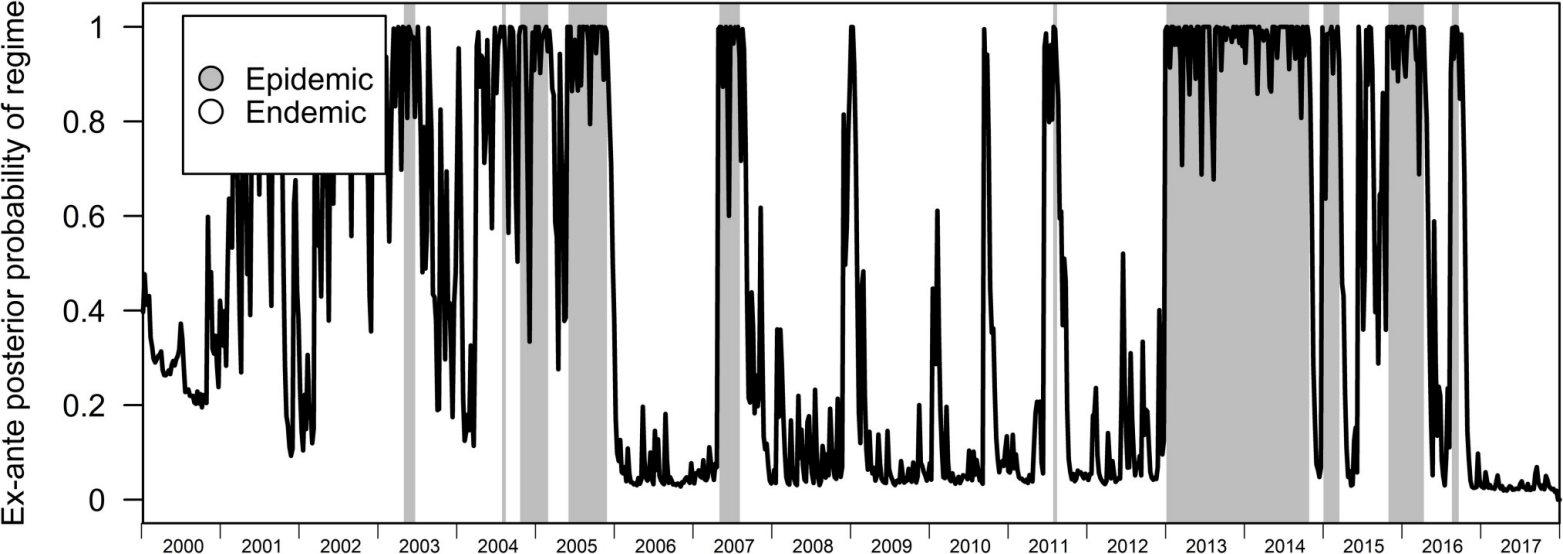
Ex-ante classification accuracy of regimes. Highlighted portions indicate regimes of the BRS-3 Lag to their corresponding posterior probabilities from 2000–2017.

In the BRS-3 Lag model, the endemic and epidemic regime lasts for around 66 and 20 weeks respectively (Table 2). While our variable of interest is differenced dengue case counts and identifying restriction set to the variance of the differenced dengue transmission counts, the model was able to correctly identify periods of high levels of dengue transmissions and periods where levels of dengue transmissions are relatively low even when we compare regimes to the undifferenced original time series (Figs 1 and 2). Posterior probabilities assigned to the epidemic state also assign high probabilities near 1 to the epidemic regime when it occurs. (Fig 3), with contemporaneous classification of regimes being fairly accurate (BRS-3 Lag Model AUC-ROC: 0.935) when we restrict the dataset to a previous timepoint compared to post-hoc assessment after sufficient data (after 2005) was provided to train the BRS-3 Lag model (Fig 4).

**Fig 5:**
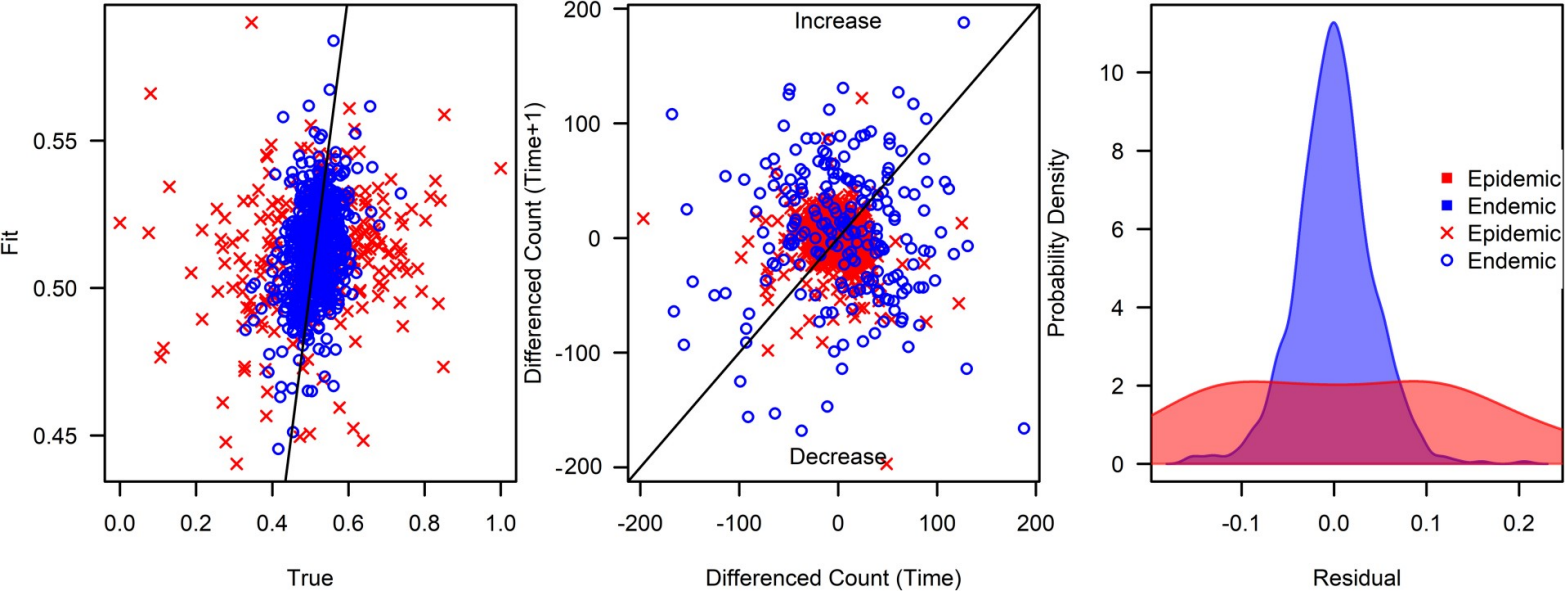
Posterior predictive check on BRS-3 Lag model. Figures from left to right represent: (1) Fitted dengue transmission counts on observed values from the BRS-3 Lag model, with line representing the Y = X function across endemic and epidemic regimes. (2) Dengue transmission counts fitted against dengue transmission counts one week ago, with line representing the Y = X function across endemic and epidemic regimes. (3) Probability density function of dengue transmission counts across endemic and epidemic regimes.

Posterior predictive checks indicate that the posterior predictive density replicates the true distribution of the data in the BRS 3 Lag model, however, more error and noise is observed in fitting the epidemic regime. (Fig 5) 95% Credible intervals exclude 0 for lags 2 and lags 2 and 3 on BAR-2 and BAR-3 Lag models respectively, but only the endemic regime lag 1 and 3 coefficient on the BRS-3 Lag model. Plotting posterior samples of coefficients across regimes showed that this is likely due to correlation between sampled parameters rather than a result of unimportant dimensions being specified (S2 Appendix).

Our results suggest persistent epidemic and endemic regimes. Transitions across regimes are characterized by the transition probabilities (TP) matrix in Table 3. This matrix shows the likelihood of being in the same regime or switching over to another Regime in the next time period. TP across the endemic (EN) and epidemic (EP) regimes are low (Table 3, TP regimes EN to EP: 2.0%, TP regimes EP to EN, 5.5%), while the TP of staying within the EN and EP regimes are high (Table 3, TP regime EN, 98.0%, TP regime EP, 94.5%). The average lengths of the endemic and epidemic regimes appear persistent but the endemic regime is marginally more so in comparison to the epidemic regime, with the epidemic regime less likely to remain in its current regime compared to the endemic regime (Table 3).

**Table 3:** Posterior transition probability matrix.

Posterior Transition Probability Matrix		
	Endemic Regime	Epidemic Regime
Endemic Regime	98.0%	2.0%
Epidemic Regime	5.5%	94.5%

^1^ Transition probability matrices were computed by averaging the sampled probabilities of being in the same regime or transitioning into another regime across MCMC samples

The BRS model characterizes 2 different stages of dengue transmission dynamics, which are apparent from the estimated regime-specific autoregressive parameters. Future changes in dengue transmission counts in the endemic regime are expected to go lower as a proportion of the observed change in dengue differenced counts one week before (Table 2, BRS-3 Lag Model, Lag 1 Autoregessive Endemic Coefficient: -0.289). The epidemic regime values are expected to increase as a proportion of the observed dengue transmission counts one to three weeks before. (Table 2, BRS-3 Lag Model, Lag 1–3 Autoregessive Epidemic Coefficients: 0.007,0.132,0.058).

**Fig 6:**
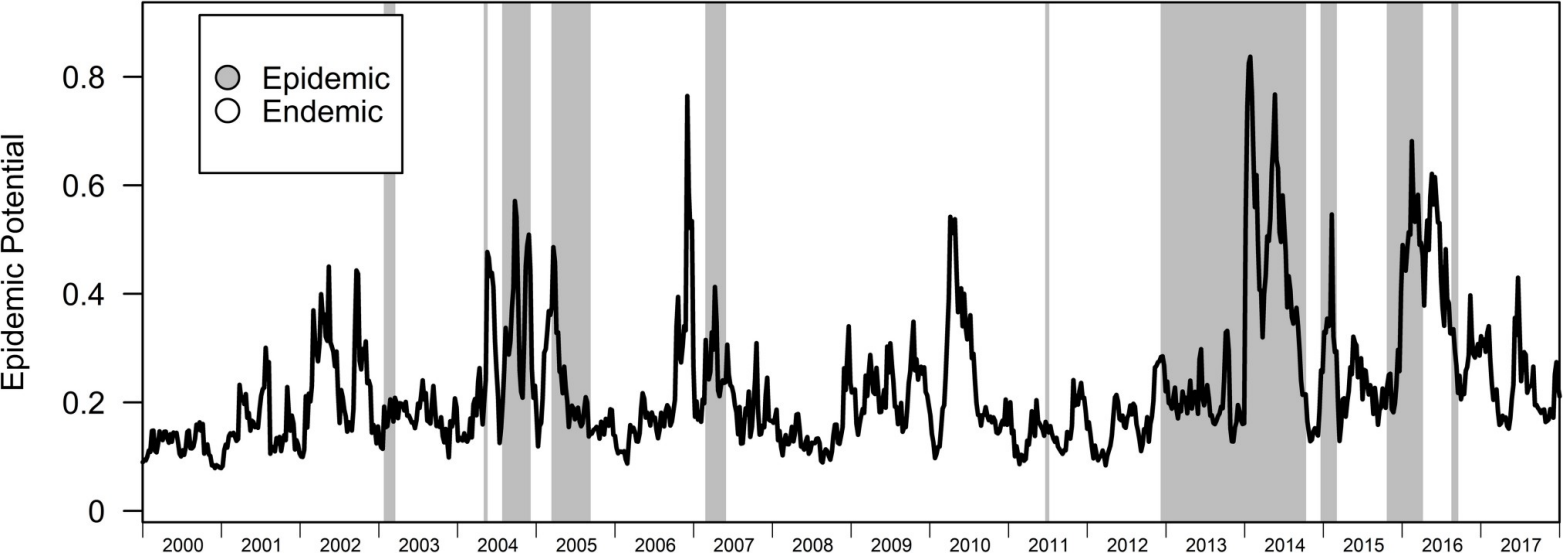
Predicted epidemic potential due to climatic factors. Highlighted portions indicate regimes of the BRS-3 Lag to their corresponding LASSO estimated epidemic potential from 2000–2017.

LASSO with logistic link was conducted using regimes from the BRS-3 Lag model as dependent variables (Table 4). Ranking the 600 independent variables according to coefficient magnitude showed that a quadratic 5 week lagged response to air temperature, equivalent 14–20 week lag for relative humidity and 2–5 week lag for 2^nd^ order interactions between relative humidity and air temperature, absolute humidity and dewpoint temperature are correlated to epidemic regime shift initiation with poor predictive ability in climatic responses with AUC = 0.603 (Fig 6). Bootstrapping the LASSO over 1000 repetitions to recover estimated coefficient intervals for inference also confirms high parameter uncertainty (S1 Appendix) with all parameter intervals crossing 0 and highly undefined curvatures over the mean, 2.5% and 97.5% quantiles of climate over the epidemic response. (S2 Appendix). The bootstrap results also suggest joint unimportance of climatic variables on epidemic probabilities due to assignment of null-values for more than 80% of the iterations across the bootstrap (S2 Appendix).

**Table 4:** Top coefficients of LASSO Model with logistic link.

LASSO Logistic Regression^1^			
Coefficient (Smallest)	Value	Coefficient (Largest)	Value
Air Temperature Squared lag 5	-0.42	Air Temperature: Relative Humidity Squared lag 13	2.75
Relative Humidity lag 19	-0.04	Dewpoint Temperature: Relative Humidity Squared lag 5	2.75
Relative Humidity lag 18	-0.03	Relative Humidity Squared lag 20	2.96
Relative Humidity lag 20	-0.02	Absolute Humidity: Relative Humidity Squared lag 2	3.29
Relative Humidity lag 16	-0.01	Absolute Humidity: Relative Humidity Squared lag 3	3.48
Relative Humidity lag 14	-0.01	Air Temperature: Relative Humidity Squared lag 4	4.54
AUC	0.603		

^1^LASSO with logistic link was tuned with 5 fold cross validation, with the dependent variable being the allocated regimes from the BRS–3 Lag model. Colons represent 2^nd^ order interaction terms between the variables listed.

**Fig 7:**
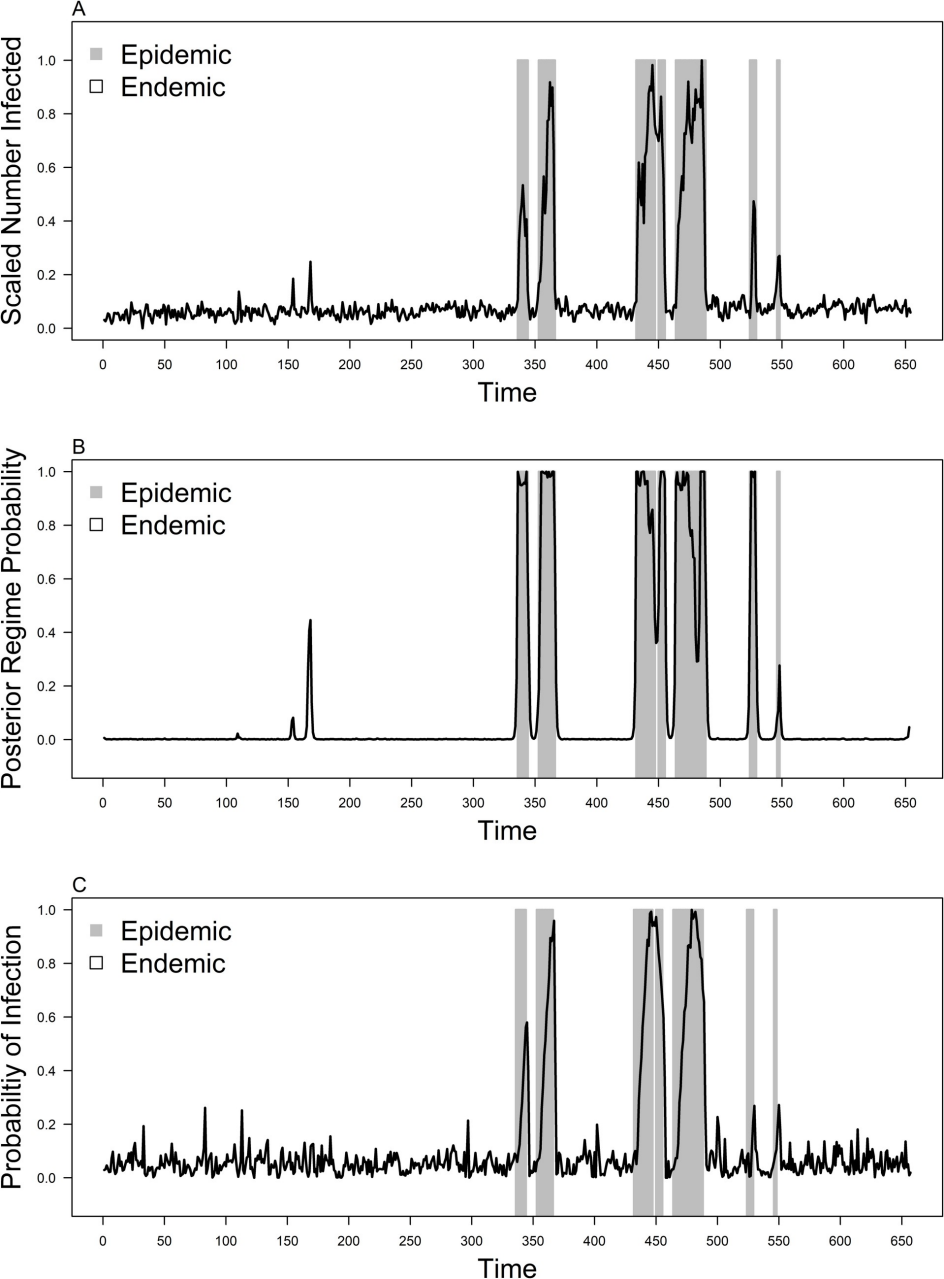
Fit of BRS to sSIR simulated case counts. Figure represents: A) Simulated infected individuals B) Fitted epidemic regime probability over the infected time series. C) Infectivity parameter used for the simulated sSIR model. Highlighted portions indicate BRS 3-Lag fitted regimes to the corresponding timepoint.

We simulated the sSIR model over a daily timescale for 6600 time points, with the burnin of 2000 time points being discarded. The remaining time points were then aggregated into the weekly level (Fig 7A). This provided a simulated dataset which has irregular fluctuations in simulated infected case counts, corresponding to the probability of infection in the imposed epidemic and endemic phases using the sSIR transition matrix (Fig 7A, 7B and 7C). Fitting BRS to the differenced and normalized simulated case counts using 3 lag terms showed that all residual auto-correlation was accounted for within the model. The fitted regimes using BRS correspond to periods where the number of infected individuals is elevated (Fig 7A) and when the probability of infection is above 0.2 in general (Fig 7C).

## Discussion

BRS models above can identify characteristic changes in the behavior of dengue case counts, which form different repeating phases where regimes alternate. The methods utilized here can also estimate the variables which explain the trends of dengue case counts in each of these regimes. We interpret regime 1 as a stable endemic regime where changes in dengue counts are pushed down to a proportion of the week before, and regime 2 an epidemic regime which is characterized by an increase in change of dengue differenced dengue counts in comparison to the week before. Both regimes, the endemic and epidemic regimes are highly noncyclical with varying temporal lengths across time in Singapore (Figs 1 and 2). Although 3 regimes were considered for dengue transmission, constant switching and non-persistence between regimes indicated overfitting for this specification (S1 Appendix) therefore 2 regimes were used to be representative of Singapore’s dengue transmission behavior.

The results show varying levels of persistence across the epidemic and endemic dengue regimes with the endemic regime generally being more persistent on average. However, when we explored the effects of climate on the estimated regimes, results suggest that climatic factors up to even 20 weeks before do not affect the probability of being within a regime or another. (S1 Appendix, S2 Appendix). While the importance of climatic factors such as temperature and humidity for dengue counts were previously discussed for Singapore [21,22] and other countries [23–25], along with preliminary estimation of a BAR-3 with climatic variables pointing towards near term effects of precipitation and dewpoint temperature on change in dengue counts up to 3 weeks before (S1 Appendix), our results suggest that regimes in dengue transmissions are driven structurally by the changes in dengue counts themselves rather than climatic factors. Mechanistically, the epidemic regimes classified using BRS on simulated data also point towards the epidemic regime as periods of high infection probability (Fig 7). Lastly, the BRS method allows nowcasting of dengue epidemics through inferring differenced dengue transmission counts with fairly high predictive accuracy (Fig 4, Table 2).

Exploring long-term structural dynamics of dengue is important for vector control as it signals that considerable forward planning and financial resource allocation is necessary for successful implementation. Distinguishing between epidemic and endemic regimes, prediction of upcoming regimes and characterizing the persistence and climatic differences of epidemics provides policy makers with the estimated duration required for epidemic control where other data such as serotype switching may be unavailable. The methods described here could be easily applied to other countries where dengue transmission counts are collected. One potential application could be to compare the regime lengths and dynamics of different countries, and explore the factors driving different dengue regime patterns.

There are several limitations of the approach outlined above. The regime switching model structure demands parsimony as each additional regime requires an additional fold of explanatory variables for estimation. Longer dynamics are thus harder to estimate for BRS. While the model estimates phenomological components, such as the evolution of dengue case counts through autoregressive parameters and structural components, such as the regimes of dengue transmission, the interpretation of structural breaks within the model remains a largely qualitative exercise. Serotype switching, which is documented to be a possible cause of epidemics in endemic regions [26] is omitted due to the unavailability of data. Sensitivity to misclassification may also make BRS models a suboptimal forecasting tool [27], which limits BRS to in-sample analysis of dengue counts. Lastly, further work is required to enhance the model to incorporate more policy components which may affect dengue transmission such as vector control efforts. Incorporating vector control and serotype switching will allow investigation in the important interactions between structural and phenomological effects on the temporal evolution of dengue.

To the best of the authors’ knowledge, this is the first application of regime switching autoregressive models for analyzing dengue transmission dynamics across separable states. We found evidence that epidemic and endemic regimes which characterize dengue transmission are highly persistent and are not associated to climatic factors. Our results point towards the need for long-term policy formation for effective vector control which is timed with upcoming epidemic switches.

## Material and methods

### Sources of data

Dengue incidence data is collected by the Ministry of Health, Singapore with mandatory notification of virologically confirmed or laboratory-confirmed cases [28]. We aggregated individual-level data into the weekly number of cases from 2000 to 2017. The Institutional Review Board of the National University of Singapore provided the ethical approval for this study.

Climate data was obtained from ERA5, published by the European Centre for Medium-Range Weather Forecasts. ERA5 provides hourly estimates across a 30km grid [29], which we have aggregated over a weekly timescale and spatially averaged over Singapore. Mean, minimum and maximum air temperature at 2m was calculated to represent thermal forcing and stress on vector population growth, and total rainfall for the weekly interval obtained for breeding site availability. Air temperature and dewpoint temperature were utilized to calculate saturation vapor pressure and actual vapor pressure using Tetens formula, whence relative and absolute humidity could be estimated using standard formula [30].

### Statistical Analysis

#### Bayesian Autoregressive (BAR) Models

Firstly, we built parsimonious BAR models with 2 to 4 lags with differenced dengue counts as the dependent variable to study the effects of past differenced dengue counts and climatic variables on current differenced dengue counts, until residual autocorrelation was sufficiently accounted for (S2 Appendix). Differenced dengue counts were utilized to ensure that our dependent variable is a difference stationary process. We let *Y*_*t*_ denote dengue differenced dengue counts for week t, *X*_*t*_ denote one or more exogenous climatic variables while *ε*_*t*_~*N*(0,*σ*^2^) represents white noise. *β*_*i*_ represents the autoregressive term which is estimated for a maximum of *p* number of lags

$$Y_{t}=\beta_{0}+\sum_{i=1}^{p}{(\beta }_{i}Y_{t-i}+\theta_{i}X_{t-i})+\varepsilon_{t}$$ (1)

We placed the canonical normal prior on *β*_0_,*β*_*i*_,*θ*_*i*_~*N*(0,100) having a large variance for the intercept, AR and exogenous climatic parameters to impose noninformativeness. The inverse gamma prior is placed on the variance parameter *σ*~*IG*(0.5,0.5) with rate and shape hyperparameters made equal to also impose noninformativeness. Conditional conjugacy between model priors and likelihood allows for Gibbs sampling of parameter posteriors. Gibbs sampling for BAR is run with 50000 iterations with a burnin of 5000. S1 Appendix details the derivation of these distributions and full computational strategy.

#### Bayesian Regime Switching Models

Regime switching models [31] were used to estimate the dynamics and change points in dengue transmission across time. In contrast to normal autoregressive models, regime switching models are characterized by multiple autoregressive models contingent on which regime the dependent variables are currently in. The estimation detects and fits separate models depending on its classification at the current time point of an epidemic or endemic regime.

The Bayesian fixed transition probability regime switching (BRS) model [16] was utilized (2).

$$Y_{t}=\beta_{s_{t},0}+\sum_{i=1}^{p}\beta_{s_{t},i}Y_{t-i}+\varepsilon_{s_{t}}$$ (2)

Where *Y*_*t*_ denote differenced dengue counts, *s*_*t*_ indexes the regime at the *t*^*th*^ timepoint and $\varepsilon_{s_{t}}∼N(0,\sigma_{s_{t}}^{2})$ represents white noise. *s*_*t*_ follows the Markov property with a transition matrix to be estimated. The intercept $\beta_{S_{t},0}$ may vary across regimes, as well as the regime specific autoregressive and variance terms parameterized by $\beta_{s_{t},i}$ and $\sigma_{s_{t}}^{2}$ for a maximum of *p* number of lags. Additionally, as climatic signals on dengue counts were found to be weak in the Bayesian autoregressive case, they were omitted from the BRS specification for model parsimony.

To estimate our model, we placed the same normal and inverse gamma priors on our regression and variance parameters *β*_*s*_~*N*(0,100) and *σ*_*s*_~*IG*(0.5,0.5) respectively. Regimes are sampled using multi-move Gibbs sampling via the Carter-Kohn recursion [16] with up to 3 regimes considered. The recursion first conducts a forward pass filtering step to infer the probability of arriving at a regime given the first *t* observations for all *tϵ*{1,…,*T*}, where *T* denotes the final time point. Next, the backward pass smoothing step provides the probabilities of being in a regime at *t*, given its future observations {*t*+1,…,*T*}. The second step allows the recursion to consider the full data likelihood and provides assignment of datapoints to each regime, which were then post-hoc labelled based on their behavior. The Dirichlet prior *ξ*_1_~*Dir*(25,5),*ξ*_2_~*Dir*(5,25) was also placed on each row of the transition matrix, dictating the belief that the probability of staying within one regime is higher than the probability of transitioning to another. We impose the identifiability constraint *σ*_*epidemic*_>*σ*_*endemic*_ to account for label switching. This is reasonable as dengue transmission counts should fluctuate more in absolute numbers within an epidemic compared to endemic period. These steps are nested within a Gibbs sampling framework due to prior-likelihood conditional conjugacy. Gibbs sampling for BRS is run with 50000 iterations with a burn-in of 5000. Full computational details are provided in the S1 Appendix.

#### Model assessment

First, Geweke convergence diagnostic checks are conducted to ensure that MCMC estimation is well-behaved [32]. Residual autocorrelation is computed to ascertain whether dengue transmission dynamics are properly accounted for and to determine the maximum lag order for each specification. Next, posterior predictive checks are conducted by comparing the fit of the posterior predictive distribution with the actual data. Fourth, posterior probabilities of the fitted regimes, which provide a measure of uncertainty to regime classification are computed. Fifth, we used mean-absolute percentage error (MAPE) and log Bayes factor as the model assessment criterion for comparing model fit of dengue differenced transmission counts between BAR and BRS as it balances explanatory power of the estimated model along with parsimony. The log Bayes factor was computed using naïve Monte Carlo simulation as detailed in S1 Appendix. Additionally, the relative deviance information criterion (DIC) comparing BRS models to the BAR models was computed, as detailed in S1 Appendix. Lastly, ex-ante classification efficacy of BRS on regimes is conducted in a rolling manner, where we fit the BRS specification sequentially from around 1/3 of the data set at the 250^th^ week onwards and increase the information set provided to the BRS by 1 more week in each refitting. We obtain the contemporaneously classified regime from the regime fitted to the final timepoint in each model iteration and compared the classification to the case where BRS is estimated on the full dataset.

#### Least Absolute Shrinkage and Selection Operator (LASSO)

The least absolute shrinkage and selection operator (LASSO) was used to estimate the influence of climate on dengue transmission behavior, due to its ability to provide both model parsimony and regularization in a high dimensional climatic space to enhance predictive accuracy and interpretability. Briefly, we fit LASSO (3) with a logistic link with *Y*_*t−i*,*j*_ locally measured climatic factors on *S*_*t*_ estimated regimes obtained from (2). Factors considered were dewpoint temperature, air temperature, precipitation, absolute and relative humidity of up to 20 weeks so that possibly long-term climatic fluctuations may be taken into account. These factors were normalized 0 to 1 by subtracting each factor by its minimum value and dividing each differenced factor by the range of values each factor observes. Normalization was conducted to account for different units of measurement and the non-invariance of LASSO regularization to scale [33]. Squared transformations and 2^nd^ order interactions were also considered to estimate possibly nonlinear relationships between climate and mosquito biology.

$$\log\frac{p(S_{t}=1)}{1-p(S_{t}=1)}=\beta_{0}+\sum_{j\in \{climate\}}\sum_{i=1}^{20}(\beta_{i,j,1}Y_{t-i,j}+\beta_{i,j,2}Y_{t-i,j}^{2})+\sum_{j,k\in \{climate\},j\neq k}\sum_{i=1}^{20}(\beta_{i,jk,1}Y_{t-i,j}Y_{t-i,k}+\beta_{i,jk,2}Y_{t-i,j}^{2}Y_{t-i,k}^{2})$$ (3)

subject to the constraint that ||***β***||_1_≤*λ*, for some penalty term *λ*, as estimated below.

Five-fold cross validation was first conducted to yield test error rates which do not suffer from unreasonably high bias or variance [34]. The cross-validation step optimizes the regularization parameter *λ* using area under curve of the receiving operator characteristic (AUC-ROC) as the tuning criterion. We then refitted our data using the optimal regularization parameter λ* to produce probabilities for being in each regime at each timepoint. Next, bootstrapping was conducted over 1000 iterations to recover confidence intervals and bootstrap mean estimates [33] for each of our LASSO dependent variables. The bootstrap also allows computation of LASSO inclusion probabilities, which provides a measure of the number of times the LASSO estimation strategy assigns a parameter null value.

#### Stochastic Susceptible-Infected-Recovered Model

Lastly, to provide a mechanistic interpretation of the labelled regimes, in addition to looking at posterior transition probabilities and the data fit to the regimes on actual case count data, we fit BRS to data simulated using a stochastic Susceptible-Infected-Recovered (sSIR) model. The sSIR model was used due to its ability to generate realistic time series of disease case counts [6]. For the sSIR, we first let infections be parameterized by separate infection functions with seasonality, state (epidemic or endemic) and population dependence. sSIR difference equations are then iterated forward in time to provide a simulated time series of infected individuals. Simulated infected individuals were then pre-processed through normalization and differencing, with regime classification conducted by fitting the simulated time series using Bayesian regime switching following the same estimation steps conducted for dengue case counts. The full technical details are provided in S3 Appendix.

## Supporting information

S1 Appendix (PDF)

S2 Appendix (PDF)

S3 Appendix (PDF)
