# Supplementary material for: Inference on dengue epidemics with Bayesian regime switching models
Source: PLoS Comput Biol. 2020 May 1;16(5):e1007839. doi: 10.1371/journal.pcbi.1007839 (PMC7219790; doi:10.1371/journal.pcbi.1007839)
Supplement: S1 Appendix — (PDF) [file pcbi.1007839.s001.pdf]

Technical Appendix 1

Inference on Dengue epidemics with Bayesian regime switching models

February 8, 2020

Contents

1 Model Specification (Bayesian Autoregression) 1

2 Model Estimation (Bayesian Autoregression) 2

3 Model Specification (Bayesian Regime Switching) 3

4 Model Estimation (Bayesian Regime Switching) 3

5 Bayes Factor Computation 4

6 Deviance Information Criterion Computation 4

7 3 Regime Bayesian Regime Switching Fit to dengue case counts 5

8 Bayesian Autoregression with Lagged Climatic Parameters 6

9 LASSO Bootstrap Mean and Quantiles 7

10 Geweke Convergence Diagnostics 11

1 Model Specification (Bayesian Autoregression)

We consider the Bayesian autoregressive (AR) model of order p as follows,

$$y_t = \beta_1 + y_{t-1}\beta_2 + \cdots + y_{t-p}\beta_p + \epsilon_t$$

Notation is suppresed to matrix form as follows for ease of exposition of posterior distributions, with n denoting the number of observations, ϵ some common noise term parameterized by σ^2:

$$Y_{n\times 1} = X_{n\times p}\beta_{p\times 1} + \epsilon \tag{1}$$

$$\epsilon \sim N\left(0, \sigma^2\right)$$

We estimate sequentially $\theta = \{\boldsymbol{\beta}, \sigma^2\}$ by placing the following priors on parameters:

$$\boldsymbol{\beta} \sim N\left(\boldsymbol{\beta_0}, \sigma^2\mathbf{P}_0\right)$$

with $\boldsymbol{\beta_0} = \mathbf{0}_{p\times 1}$ and $\sigma^2\mathbf{P}_0 = \text{diag}(100)_{p\times p}$ for our parameters to be centered around 0 and having a wide variance to impose noninformativeness.

$$\sigma^2 \sim IG\left(\frac{v_0}{2}, \frac{v_0\sigma_0^2}{2}\right)$$

with scalar $v_0 = v_0\sigma_0^2 = 1$ to yield a non-informative inverse-gamma prior distribution for σ.

2 Model Estimation (Bayesian Autoregression)

The Gibbs sampler proceeds by sequentially sampling the conditional posteriors for each of our parameters specified above.

For β, we block sample sequentially across each dimension, conjugacy between the normally distributed prior and normally distributed likelihood yields a normally distributed posterior with mean $\beta^*_{p\times 1}$ and variance $\sigma^2_\beta \mathbf{P}_{1,p\times p}$,

$$\begin{aligned} p\left(\beta \mid \theta_{-\beta}\right) &\propto p\left(Y \mid \beta, \sigma^2\right) p\left(\beta \mid \sigma^2\right) \\ &\sim N\left(\beta^*, \sigma^2_\beta \mathbf{P}_1\right) \\ \mathbf{P}_1 &= \left(\mathbf{P}_0^{-1} + X'X\right)^{-1} \\ \beta^* &= \mathbf{P}_1\left(X'Y + \mathbf{P}_0\beta_0\right) \end{aligned} \tag{2}$$

For σ^2, conjugacy between the inverse gamma prior and normally distributed likelihood yields an inverse gamma posterior with scalar shape and rate parameters $v_1/2$ and v_1/σ_1^2 respectively,

$$\begin{aligned} p\left(\sigma^2 \mid \theta_{-\sigma^2}\right) &\propto p\left(Y \mid \theta\right) p\left(\beta \mid \sigma^2, Y\right) p\left(\sigma^2\right) \\ &\sim IG\left(v_1/2, v_1\sigma_1^2\right) \\ v_1 &= v_0 + (n-p) \\ \sigma_1^2 &= v_1^{-1}\left(v_0\sigma_0^2 + a + b\right) \\ a &= \left(y - X\beta^*\right)'\left(y - X\beta^*\right) \\ b &= \left(\beta^* - \beta_0\right)' \mathbf{P_0}\left(\beta^* - \beta_0\right) \end{aligned} \tag{3}$$

Steps (1) and (2) are iterated until convergence.

3 Model Specification (Bayesian Regime Switching)

We consider a 2 regime Bayesian regime switching (BRS) model of order p with regime specific autoregressive parameters. $\beta_{p,s_t=k}$ denoting the pth autoregressive term for the kth regime at time t and similarly $\sigma^2_{s_t} = k$ the variance parameter for the noise term of the kth regime at time t as follows,

$$y_t = \beta_{1,s_t} + y_{t-1}\beta_{2,s_t} + \cdots + y_{t-p}\beta_{p,s_t} + \epsilon_t$$

Notation here is also suppressed to matrix form for ease of deriving posterior distributions, with n_s denoting the number of observations, ϵ_s a regime specific noise term parameterized by σ^2_s:

$$Y_{n_s\times 1} = X_{n_s\times p}\beta_{s,p\times 1} + \epsilon_s \tag{4}$$

$$\epsilon_s \sim N\left(0, \sigma^2_s\right)$$

The transition matrix $\boldsymbol{\xi}_t$ characterizes probabilities of switching between two states at each time point t:

$$\boldsymbol{\xi_t} = \begin{bmatrix} p_{11,t} & p_{12,t} \\ p_{21,t} & p_{22,t} \end{bmatrix}$$

We estimate sequentially $\theta = \{\beta_{s,p\times 1}, \sigma^2_s, s_{1:t}, \boldsymbol{\xi}_t\}$ by placing the following priors on parameters, states and transition matrix:

$$\beta_{s,p\times 1} \sim N\left(\beta_0, \sigma^2_s \mathbf{P}_0\right)$$

with $\boldsymbol{\beta_0} = \mathbf{0}_{p\times 1}$ and $\sigma^2_s\mathbf{P}_0 = \text{diag}(100)_{p\times p}$ for our parameters to be centered around 0 and having a wide variance to impose noninformativeness.

$$\sigma^2_s \sim IG\left(\frac{v_0}{2}, \frac{v_0\sigma^2_0}{2}\right)$$

with scalar $v_0 = v_0\sigma^2_0 = 1$ to yield a non-informative inverse-gamma prior distribution for σ_s.

$$\boldsymbol{\xi} \sim Dir(e_{j1}, \ldots, e_{jk})$$

with $j, k \in \{1, 2\}$ denoting the regime index and $e_{11} = 5, e_{12} = 15$ for the two regime BRS to impose the belief that dengue transmissions are more likely to stay within their own regime rather than move to another regime.

4 Model Estimation (Bayesian Regime Switching)

The Gibbs sampler proceeds by sequentially sampling the conditional posteriors for each of our parameters specified above.

First, we conduct multi-move sampling on $\mathbf{S}_{1:t}$ with the conditional posteriors given by (5), with $p(S_t \mid Y_t)$ obtained using the Hamilton filter and $\prod_{k=1}^{T-1} p(S_k \mid S_{k+1}, Y_t)$ through the Carter-Kohn recursion as described by Kim and Nelson 1999 [2].

$$p(\mathbf{S}_{1:t} \mid \theta_{-S}) = p(S_t \mid Y_t) \prod_{k=1}^{T-1} p(S_k \mid S_{k+1}, Y_t) \tag{5}$$

Next, to sample the transition probability matrix ξ_t, we note that with K denoting the number of regimes estimated for the BRS, $\mathbf{S} \in \{s_1 \ldots s_t\}$ has the complete likelihood:

$$p(\mathbf{S} \mid \xi_t) \ = \prod_{j=1}^{K}\prod_{k=1}^{K} \xi_{jk,t}^{N_{jk,t}}(s)$$

where $N_{jk,t}$ denotes counts for the transition from j^{th} to k^{th} states at time t, we derive:

$$p(\xi_t \mid \mathbf{S}) \propto Dir(e_{j1} + N_{j1,t}(s), \ldots, e_{jk} + N_{jk,t}(s)) \tag{6}$$

For σ^2_s, conditional on the state sampled from (5), conjugacy between the inverse gamma prior and normally distributed likelihood yields an inverse gamma posterior with scalar shape and rate parameters $v_1/2$ and v_1/σ^2_1 respectively,

$$p\left(\sigma^2_s \mid \theta_{-\sigma^2}\right) \propto p\left(Y \mid \theta_{-Y}\right) p\left(\beta \mid \sigma^2_s, Y\right) p\left(\sigma^2_s\right)$$

$$\sim IG\left(v_1/2, v_1\sigma_1^2\right) \tag{7}$$
$$v_1 = v_0 + (n-p)$$
$$\sigma_1^2 \ = v_1^{-1}\left(v_0\sigma_0^2 + a_s + b_s\right)$$
$$a_s = (y - X\beta_s^*)'\,(y - X\beta_s^*)$$
$$b_s = (\beta_s^* - \beta_0)'\,\mathbf{P_0}\,(\beta_s^* - \beta_0)$$

Similarly for β_s, conditional on the state sampled from (5), conjugacy between the normally distributed prior and normally distributed likelihood yields a normally distributed posterior with mean β_s^* and variance $\sigma_{\beta,s}^2\mathbf{P}_1$

$$p\left(\beta_s \mid \theta_{-\beta_s}\right) \propto p\left(Y \mid \beta_s, \sigma^2, s_t\right) p\left(\beta_s \mid s_t, \sigma^2\right)$$
$$\sim N\left(\beta_s^*, \sigma_{\beta,s}^2\mathbf{P}_1\right) \tag{8}$$
$$\mathbf{P}_1 = \left(\mathbf{P}_0^{-1} + X_s'X_s\right)^{-1}$$
$$\beta_s^* = \mathbf{P}_1\left(X_s'Y_s + \mathbf{P}_0\beta_{0,s}\right)$$

Lastly, to prevent label switching, we place an identification restriction on the sigma parameter such that $\sigma_{1,S=2} > \sigma_{1,S=1}$

Steps (5) - (8) are iterated until convergence

5 Bayes Factor Computation

We compute the Bayes Factor by naive Monte Carlo simulation following Gelman 2013 [1]. The posterior model likelihood for the Bayesian Autoregression and Bayesian Regime switching models are averaged over posterior samples (7) after burnin is discarded. Namely, D denotes the observed elements $D = \{X, Y\}$. For the Bayesian Autoregression $\theta = \{\beta, \sigma\}$ and for the Bayesian Autoregression, it is sufficient to consider $\theta = \{\beta, \sigma, \mathbf{S}\}$ for computing the posterior model likelihood.

$$P(D|M_i) \approx 1/n \sum_{\theta \sim P(\theta|M_i)}^{n} P(D|\theta, M_i) \tag{9}$$

Thereafter, the log Bayes factor is computed as (10).

$$\text{log Bayes factor} \approx \text{log}P(D|M_{\text{Bayesian Regime Switching}}) - \text{log}P(D|M_{\text{Bayesian Autoregression}}) \tag{10}$$

6 Deviance Information Criterion Computation

The deviance information criterion (DIC) follows [3], by defining the deviance as $D(\theta) = -2log(p(y|\theta)) + C$, with C some constant which cancels in model comparison and $log(p(y|\theta))$ the log likelihood of the model. To calculate the effective number of parameters, we take $p_D = \overline{D(\theta)} - D(\bar{\theta})$ where $\bar{\theta}$ denotes the posterior expectation of parameters, and $\overline{D(\theta)}$ denotes expectation of the log likelihood computed over posterior draws of θ. The DIC is then given by:

$$\text{DIC} = p_D + \overline{D(\theta)} \tag{11}$$

We obtain the relative DICs by computing:

$$\text{relative DIC} = \text{DIC}_{BAR} - \text{DIC}_{BRS} \tag{12}$$

7 3 Regime Bayesian Regime Switching Fit to dengue case counts

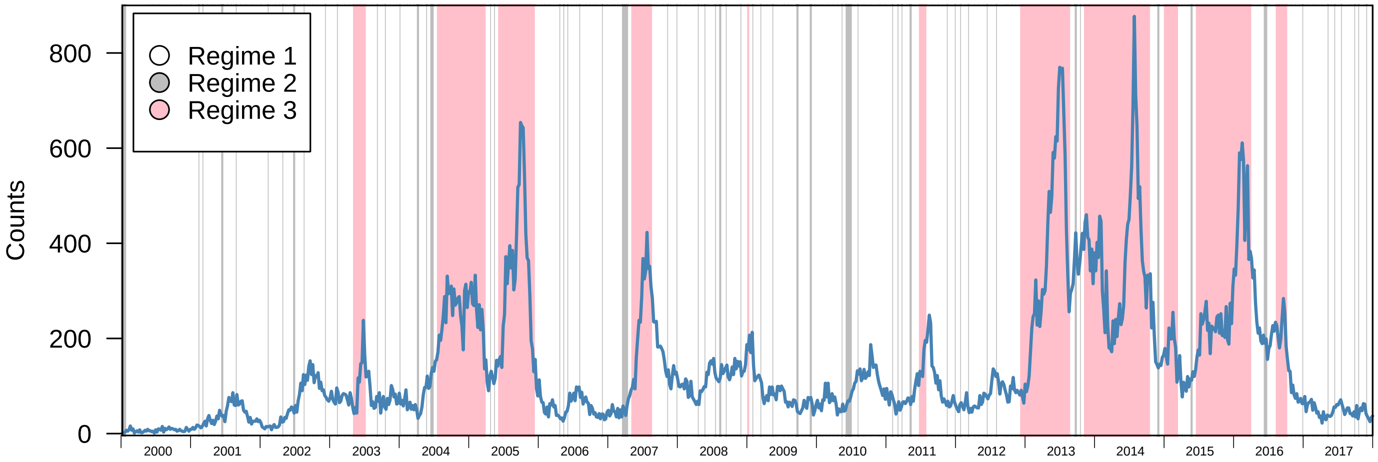

Regime switching models were estimated using Gibbs sampling, with the dependent variable being change in dengue cases, and independent variables being the first 2/3 lags of the change in dengue cases

8 Bayesian Autoregression with Lagged Climatic Parameters

	Posterior Mean	2.5% Credible Interval	97.5% Credible Interval
Lag 1 Dengue Counts	-0.08	-0.15	-0.02
Lag 2 Dengue Counts	0.11	0.04	0.17
Lag 3 Dengue Counts	0.09	0.02	0.15
Absolute Humidity Lag 1	0.35	-0.06	0.76
Absolute Humidity Lag 2	0.10	-0.34	0.54
Absolute Humidity Lag 3	-0.47	-0.86	-0.07
Relative Humidity Lag 1	-0.02	-0.12	0.07
Relative Humidity Lag 2 lag 2	-0.03	-0.14	0.07
Relative Humidity Lag 3 lag 3	-0.04	-0.14	0.06
Total Precipitation lag 1	0.02	-0.03	0.07
Total Precipitation lag 2	-0.04	-0.10	0.01
Total Precipitation lag 3	0.05	0.00	0.10
Air Temperature lag 1	-0.08	-0.53	0.37
Air Temperature lag 2	-0.19	-0.67	0.28
Air Temperature lag 3	-0.20	-0.66	0.25
Dewpoint Temperature lag 1	-0.31	-1.01	0.40
Dewpoint Temperature lag 2	0.06	-0.67	0.81
Dewpoint Temperature lag 3	0.77	0.10	1.45

*Bayesian autoregression with lagged climatic parameters was estimated Gibbs Sampling as detailed in Technical Appendix Section 2

9 LASSO Bootstrap Mean and Quantiles

	Bootstrap Mean	2.5% Bootstrap Quantile	97.5% Bootstrap Quantile
Absolute Humidity lag 1	0.076	-1.514	3.314
Absolute Humidity lag 2	0.106	-1.271	2.735
Absolute Humidity lag 3	0.095	-0.308	2.901
Absolute Humidity lag 4	0.161	0.000	3.084
Absolute Humidity lag 5	0.222	-0.003	5.709
Absolute Humidity lag 6	0.198	0.000	3.524
Absolute Humidity lag 7	0.133	-0.000	2.951
Absolute Humidity lag 8	0.150	-0.005	2.391
Absolute Humidity lag 9	0.179	0.000	2.685
Absolute Humidity lag 10	0.180	-0.000	2.736
Absolute Humidity lag 11	0.153	0.000	3.026
Absolute Humidity lag 12	0.124	0.000	1.126
Absolute Humidity lag 13	0.197	-0.024	5.276
Absolute Humidity lag 14	0.125	-0.000	2.750
Absolute Humidity lag 15	0.161	-0.003	3.175
Absolute Humidity lag 16	0.162	-0.000	3.208
Absolute Humidity lag 17	0.123	0.000	2.388
Absolute Humidity lag 18	0.150	0.000	2.794
Absolute Humidity lag 19	0.216	-0.500	6.655
Absolute Humidity lag 20	0.178	0.000	3.082
Absolute Humidity Squared lag 1	0.176	0.000	3.389
Absolute Humidity Squared lag 2	0.121	-0.581	3.817
Absolute Humidity Squared lag 3	0.168	0.000	2.666
Absolute Humidity Squared lag 4	0.051	-1.082	2.228
Absolute Humidity Squared lag 5	-1.431	0.000	0.000
Absolute Humidity Squared lag 6	0.217	-0.279	3.381
Absolute Humidity Squared lag 7	0.133	0.000	1.868
Absolute Humidity Squared lag 8	0.162	0.000	2.935
Absolute Humidity Squared lag 9	0.141	-0.046	2.955
Absolute Humidity Squared lag 10	0.090	0.000	0.000
Absolute Humidity Squared lag 11	0.157	-0.019	3.029
Absolute Humidity Squared lag 12	0.155	-0.017	2.767
Absolute Humidity Squared lag 13	0.168	-0.000	2.784
Absolute Humidity Squared lag 14	0.146	-0.000	2.819
Absolute Humidity Squared lag 15	0.301	-0.000	8.825
Absolute Humidity Squared lag 16	0.197	0.000	3.083
Absolute Humidity Squared lag 17	0.172	-0.016	0.000
Absolute Humidity Squared lag 18	0.180	-0.002	2.763
Absolute Humidity Squared lag 19	0.000	0.000	0.000
Absolute Humidity Squared lag 20	0.185	-0.012	3.342
Absolute Humidity:Relative Humidity lag 1	0.110	-0.107	2.072
Absolute Humidity:Relative Humidity lag 2	0.162	-0.001	3.219
Absolute Humidity:Relative Humidity lag 3	0.166	0.000	3.119
Absolute Humidity:Relative Humidity lag 4	0.175	-0.012	3.099
Absolute Humidity:Relative Humidity lag 5	0.146	-0.000	2.259
Absolute Humidity:Relative Humidity lag 6	0.115	-0.002	2.699
Absolute Humidity:Relative Humidity lag 7	0.112	-0.045	3.422
Absolute Humidity:Relative Humidity lag 8	0.102	-0.022	1.295
Absolute Humidity:Relative Humidity lag 9	0.151	-0.019	2.354
Absolute Humidity:Relative Humidity lag 10	0.139	0.000	2.484
Absolute Humidity:Relative Humidity lag 11	0.175	0.000	2.797
Absolute Humidity:Relative Humidity lag 12	0.154	-0.001	2.267
Absolute Humidity:Relative Humidity lag 13	0.106	-0.510	2.952
Absolute Humidity:Relative Humidity lag 14	0.402	0.000	0.005
Absolute Humidity:Relative Humidity lag 15	0.192	-0.000	3.686
Absolute Humidity:Relative Humidity lag 16	0.083	-0.931	2.864
Absolute Humidity:Relative Humidity lag 17	0.092	-0.039	3.262
Absolute Humidity:Relative Humidity lag 18	0.116	-0.005	2.527
Absolute Humidity:Relative Humidity lag 19	0.221	0.000	4.529
Absolute Humidity:Relative Humidity lag 20	0.148	-0.000	2.248
Absolute Humidity:Relative Humidity Squared lag 1	0.152	-0.050	3.325
Absolute Humidity:Relative Humidity Squared lag 2	0.133	-0.000	2.700

Absolute Humidity:Relative Humidity Squared lag 3	0.158	-0.003	2.822
Absolute Humidity:Relative Humidity Squared lag 4	0.102	-0.245	3.265
Absolute Humidity:Relative Humidity Squared lag 5	0.173	-0.001	2.786
Absolute Humidity:Relative Humidity Squared lag 6	0.180	-0.000	3.451
Absolute Humidity:Relative Humidity Squared lag 7	0.156	-0.021	3.721
Absolute Humidity:Relative Humidity Squared lag 8	-0.074	-3.561	0.183
Absolute Humidity:Relative Humidity Squared lag 9	0.100	-0.002	3.337
Absolute Humidity:Relative Humidity Squared lag 10	0.117	-2.004	5.099
Absolute Humidity:Relative Humidity Squared lag 11	0.129	-0.001	3.450
Absolute Humidity:Relative Humidity Squared lag 12	0.185	-0.028	4.445
Absolute Humidity:Relative Humidity Squared lag 13	0.229	-0.012	3.818
Absolute Humidity:Relative Humidity Squared lag 14	0.190	0.000	3.242
Absolute Humidity:Relative Humidity Squared lag 15	0.195	-0.011	3.361
Absolute Humidity:Relative Humidity Squared lag 16	0.258	-0.001	3.829
Absolute Humidity:Relative Humidity Squared lag 17	0.164	-0.000	2.873
Absolute Humidity:Relative Humidity Squared lag 18	0.227	0.000	0.001
Absolute Humidity:Relative Humidity Squared lag 19	-0.004	-1.113	1.634
Absolute Humidity:Relative Humidity Squared lag 20	0.117	-0.001	2.263
Air Temperature lag 1	0.026	-0.389	1.539
Air Temperature lag 2	0.009	-0.133	1.081
Air Temperature lag 3	0.174	-0.000	3.402
Air Temperature lag 4	-0.050	-2.460	0.708
Air Temperature lag 5	0.193	0.000	2.750
Air Temperature lag 6	0.850	0.000	0.000
Air Temperature lag 7	0.142	0.000	1.820
Air Temperature lag 8	0.122	-0.008	3.039
Air Temperature lag 9	0.138	-0.001	3.096
Air Temperature lag 10	0.158	-0.024	2.938
Air Temperature lag 11	0.359	-0.099	6.099
Air Temperature lag 12	0.160	-0.000	3.311
Air Temperature lag 13	0.149	-0.077	2.759
Air Temperature lag 14	0.194	-0.002	3.617
Air Temperature lag 15	0.133	0.000	1.936
Air Temperature lag 16	0.178	-0.016	4.254
Air Temperature lag 17	0.127	-0.003	1.761
Air Temperature lag 18	0.126	-0.000	2.427
Air Temperature lag 19	0.149	-0.096	2.431
Air Temperature lag 20	0.127	-0.002	3.524
Air Temperature Squared lag 1	0.000	0.000	0.000
Air Temperature Squared lag 2	0.374	0.000	5.735
Air Temperature Squared lag 3	0.188	0.000	3.739
Air Temperature Squared lag 4	0.100	-0.082	2.932
Air Temperature Squared lag 5	0.151	0.000	3.165
Air Temperature Squared lag 6	0.347	0.000	5.995
Air Temperature Squared lag 7	0.122	-0.085	2.572
Air Temperature Squared lag 8	0.132	-0.005	2.503
Air Temperature Squared lag 9	0.065	-1.314	3.019
Air Temperature Squared lag 10	0.173	0.000	2.962
Air Temperature Squared lag 11	0.146	-0.086	3.016
Air Temperature Squared lag 12	0.170	0.000	2.878
Air Temperature Squared lag 13	0.161	-0.000	2.550
Air Temperature Squared lag 14	0.103	-0.016	2.881
Air Temperature Squared lag 15	0.135	0.000	1.778
Air Temperature Squared lag 16	0.099	-0.001	1.555
Air Temperature Squared lag 17	0.226	-0.064	3.806
Air Temperature Squared lag 18	0.117	-0.014	2.471
Air Temperature Squared lag 19	0.144	-0.002	2.373
Air Temperature Squared lag 20	0.167	-0.002	3.583
Air Temperature:Absolute Humidity lag 1	0.121	-0.001	2.301
Air Temperature:Absolute Humidity lag 2	0.164	0.000	3.164
Air Temperature:Absolute Humidity lag 3	0.147	-0.001	2.690
Air Temperature:Absolute Humidity lag 4	0.171	0.000	3.780
Air Temperature:Absolute Humidity lag 5	0.125	-0.028	2.958
Air Temperature:Absolute Humidity lag 6	0.167	-0.000	2.934
Air Temperature:Absolute Humidity lag 7	0.151	-0.049	2.571

Air Temperature:Absolute Humidity lag 8	0.134	-0.000	2.895
Air Temperature:Absolute Humidity lag 9	0.204	-0.000	3.523
Air Temperature:Absolute Humidity lag 10	0.213	0.000	4.048
Air Temperature:Absolute Humidity lag 11	0.144	0.000	2.669
Air Temperature:Absolute Humidity lag 12	0.154	0.000	2.646
Air Temperature:Absolute Humidity lag 13	0.212	-0.206	3.609
Air Temperature:Absolute Humidity lag 14	0.211	0.000	3.766
Air Temperature:Absolute Humidity lag 15	0.079	-0.344	3.044
Air Temperature:Absolute Humidity lag 16	0.132	-0.001	1.860
Air Temperature:Absolute Humidity lag 17	0.140	-0.128	3.448
Air Temperature:Absolute Humidity lag 18	0.133	0.000	2.504
Air Temperature:Absolute Humidity lag 19	0.096	-0.000	3.035
Air Temperature:Absolute Humidity lag 20	0.116	-0.000	2.040
Air Temperature:Absolute Humidity Squared lag 1	0.158	0.000	2.141
Air Temperature:Absolute Humidity Squared lag 2	0.173	0.000	3.021
Air Temperature:Absolute Humidity Squared lag 3	0.142	0.000	2.172
Air Temperature:Absolute Humidity Squared lag 4	0.083	-0.006	2.397
Air Temperature:Absolute Humidity Squared lag 5	0.141	-0.025	2.219
Air Temperature:Absolute Humidity Squared lag 6	0.148	-0.000	1.846
Air Temperature:Absolute Humidity Squared lag 7	-0.065	0.000	0.000
Air Temperature:Absolute Humidity Squared lag 8	0.134	-0.005	2.349
Air Temperature:Absolute Humidity Squared lag 9	0.175	0.000	3.323
Air Temperature:Absolute Humidity Squared lag 10	0.183	-0.000	3.299
Air Temperature:Absolute Humidity Squared lag 11	0.110	-0.057	2.846
Air Temperature:Absolute Humidity Squared lag 12	0.155	-0.010	2.636
Air Temperature:Absolute Humidity Squared lag 13	0.095	-0.090	1.320
Air Temperature:Absolute Humidity Squared lag 14	0.153	0.000	3.593
Air Temperature:Absolute Humidity Squared lag 15	0.223	-0.031	4.121
Air Temperature:Absolute Humidity Squared lag 16	0.083	0.000	1.475
Air Temperature:Absolute Humidity Squared lag 17	0.225	0.000	3.892
Air Temperature:Absolute Humidity Squared lag 18	0.158	0.000	2.996
Air Temperature:Absolute Humidity Squared lag 19	0.125	0.000	1.827
Air Temperature:Absolute Humidity Squared lag 20	0.150	0.000	1.800
Air Temperature:Relative Humidity lag 1	0.123	0.000	1.882
Air Temperature:Relative Humidity lag 2	0.270	-0.012	3.286
Air Temperature:Relative Humidity lag 3	0.049	-3.004	2.355
Air Temperature:Relative Humidity lag 4	0.144	-0.002	3.986
Air Temperature:Relative Humidity lag 5	0.128	-0.000	2.220
Air Temperature:Relative Humidity lag 6	0.155	0.000	3.176
Air Temperature:Relative Humidity lag 7	0.199	-0.000	4.955
Air Temperature:Relative Humidity lag 8	0.034	-0.383	1.273
Air Temperature:Relative Humidity lag 9	0.113	-0.014	3.205
Air Temperature:Relative Humidity lag 10	0.128	0.000	2.766
Air Temperature:Relative Humidity lag 11	0.200	0.000	0.002
Air Temperature:Relative Humidity lag 12	0.154	-0.018	2.232
Air Temperature:Relative Humidity lag 13	0.196	0.000	4.162
Air Temperature:Relative Humidity lag 14	0.238	-0.650	3.540
Air Temperature:Relative Humidity lag 15	0.127	-0.000	1.821
Air Temperature:Relative Humidity lag 16	0.109	0.000	2.585
Air Temperature:Relative Humidity lag 17	0.247	0.000	4.331
Air Temperature:Relative Humidity lag 18	0.226	0.000	3.809
Air Temperature:Relative Humidity lag 19	0.196	0.000	4.266
Air Temperature:Relative Humidity lag 20	0.183	-0.124	3.114
Air Temperature:Relative Humidity Squared lag 1	0.169	0.000	2.354
Air Temperature:Relative Humidity Squared lag 2	0.178	0.000	2.663
Air Temperature:Relative Humidity Squared lag 3	0.157	0.000	2.936
Air Temperature:Relative Humidity Squared lag 4	0.182	-0.245	3.984
Air Temperature:Relative Humidity Squared lag 5	0.144	-0.018	3.050
Air Temperature:Relative Humidity Squared lag 6	0.144	-0.018	3.050
Air Temperature:Relative Humidity Squared lag 7	0.151	0.000	3.071
Air Temperature:Relative Humidity Squared lag 8	0.166	-0.000	3.019
Air Temperature:Relative Humidity Squared lag 9	0.142	-0.003	2.358
Air Temperature:Relative Humidity Squared lag 10	0.103	-0.004	2.741
Air Temperature:Relative Humidity Squared lag 11	0.153	0.000	1.857
Air Temperature:Relative Humidity Squared lag 12	0.196	-0.000	3.221

Air Temperature:Relative Humidity Squared lag 13	0.155	-0.000	3.298
Air Temperature:Relative Humidity Squared lag 14	0.134	-0.001	2.692
Air Temperature:Relative Humidity Squared lag 15	0.314	-0.000	5.057
Air Temperature:Relative Humidity Squared lag 16	-0.050	-2.646	1.279
Air Temperature:Relative Humidity Squared lag 17	0.154	-0.010	3.482
Air Temperature:Relative Humidity Squared lag 18	0.170	-0.001	3.189
Air Temperature:Relative Humidity Squared lag 19	0.130	-0.001	1.866
Air Temperature:Relative Humidity Squared lag 20	0.060	-0.335	4.963

LASSO coefficients were estimated using 5-fold cross validation with the dependent variable being regimes estimated from the 2-regime 3 lag BRS specification and independent variables being 20 lags of observed climatic factors. Bootstrapping was conducted by sampling the dataset with replacement and conducting the LASSO estimation procedure on the subsetted data over 1000 repetitions. The bootstrap repetitions were then used to reconstruct the LASSO estimated coefficient distributions and summary statistics.

10 Geweke Convergence Diagnostics

Table 2:

Variable	Z Statistic
$\beta_{Endemic,0,BRS-2}$	-0.024
$\beta_{Endemic,1,BRS-2}$	-1.52
$\beta_{Endemic,2,BRS-2}$	-0.52
$\beta_{Epidemic,0,BRS-2}$	-1.68
$\beta_{Epidemic,1,BRS-2}$	0.38
$\beta_{Epidemic,2,BRS-2}$	0.46
$\beta_{Endemic,0,BRS-3}$	0.86
$\beta_{Endemic,1,BRS-3}$	-0.30
$\beta_{Endemic,2,BRS-3}$	1.12
$\beta_{Endemic,3,BRS-3}$	-0.25
$\beta_{Epidemic,0,BRS-3}$	0.45
$\beta_{Epidemic,1,BRS-3}$	-1.36
$\beta_{Epidemic,2,BRS-3}$	0.39
$\beta_{Epidemic,3,BRS-3}$	1.04
$\beta_{Endemic,0,BRS-4}$	-0.53
$\beta_{Endemic,1,BRS-4}$	-0.45
$\beta_{Endemic,2,BRS-4}$	0.22
$\beta_{Endemic,3,BRS-4}$	0.17
$\beta_{Endemic,4,BRS-4}$	1.5
$\beta_{Epidemic,0,BRS-4}$	-0.53
$\beta_{Epidemic,1,BRS-4}$	-0.45
$\beta_{Epidemic,2,BRS-4}$	0.22
$\beta_{Epidemic,3,BRS-4}$	0.17
$\beta_{Epidemic,4,BRS-4}$	1.47

*Geweke convergence diagnostic test [1] was compared against $\alpha = 0.05$, $z^* = 1.95$

References

[1] Andrew Gelman, Hal S Stern, John B Carlin, David B Dunson, Aki Vehtari, and Donald B Rubin. *Bayesian data analysis*. Chapman and Hall/CRC, 2013.

[2] Chang-Jin Kim and Charles R Nelson. State-space models with regime switching: classical and gibbs-sampling approaches with applications. *MIT Press Books*, 1, 1999.

[3] David J Spiegelhalter, Nicola G Best, Bradley P Carlin, and Angelika Van Der Linde. Bayesian measures of model complexity and fit. *Journal of the royal statistical society: Series b (statistical methodology)*, 64(4):583–639, 2002.
