# Supplementary material for: Inference on dengue epidemics with Bayesian regime switching models
Source: PLoS Comput Biol. 2020 May 1;16(5):e1007839. doi: 10.1371/journal.pcbi.1007839 (PMC7219790; doi:10.1371/journal.pcbi.1007839)

Technical Appendix 2

Inference on Dengue epidemics with Bayesian regime switching models

February 8, 2020

Contents

1 Posterior Samples Across Regimes **2**

2 Auto-correlation Functions for Bayesian Autoregression and Bayesian Regime Switching **3**

3 Convergence Plots for Bayesian Autoregression and Bayesian Regime Switching **4**

4 Prior-Posterior Plots for Bayesian Regime Switching **6**

5 Bootstrap Distributions for LASSO **9**

6 Curvature Representations for LASSO **108**

7 Bootstrap Inclusion Probabilities for LASSO **133**

S1, Figure 1 : Posterior Samples of β_1 Across Regimes

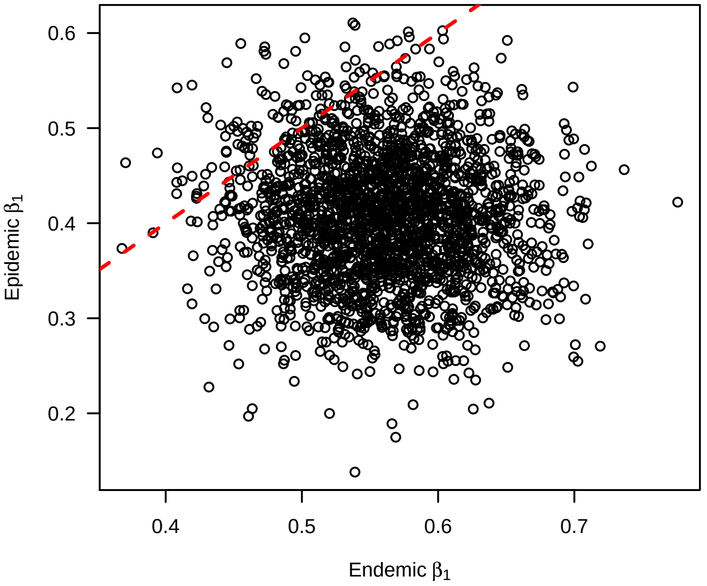

S1, Figure 2 : Posterior Samples of β_2 Across Regimes

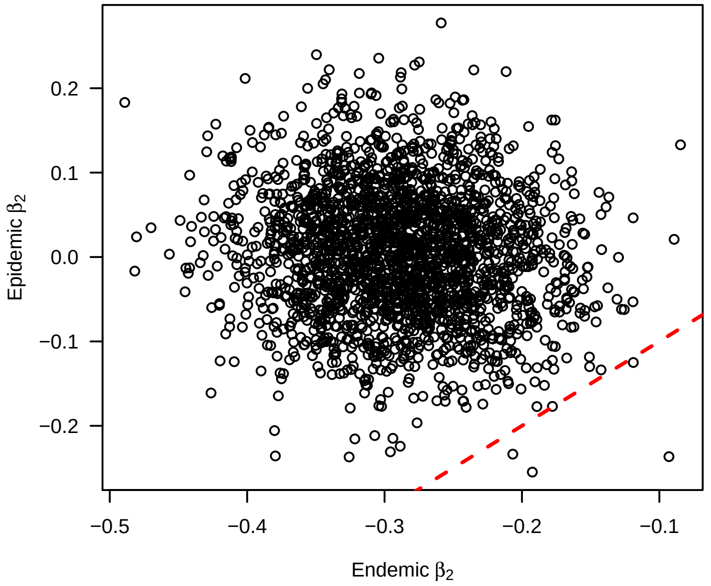

S1, Figure 3 : Posterior Samples of β_3 Across Regimes

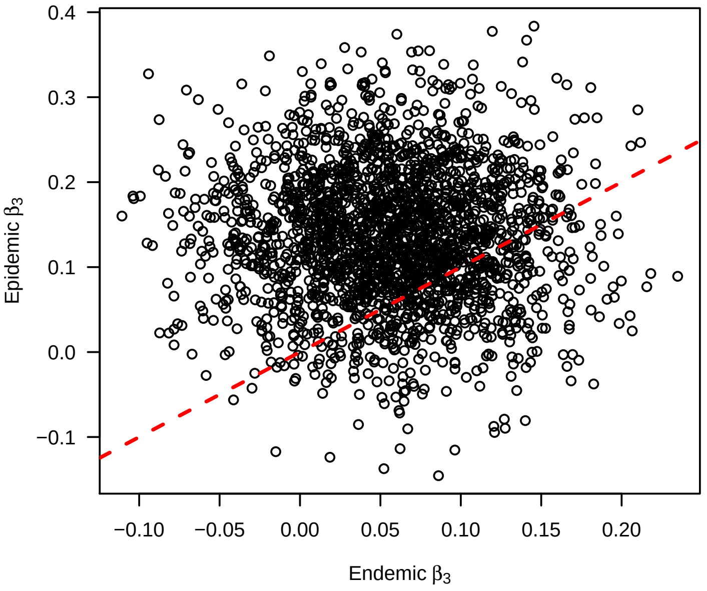

S1, Figure 4 : Posterior Samples of β_4 Across Regimes

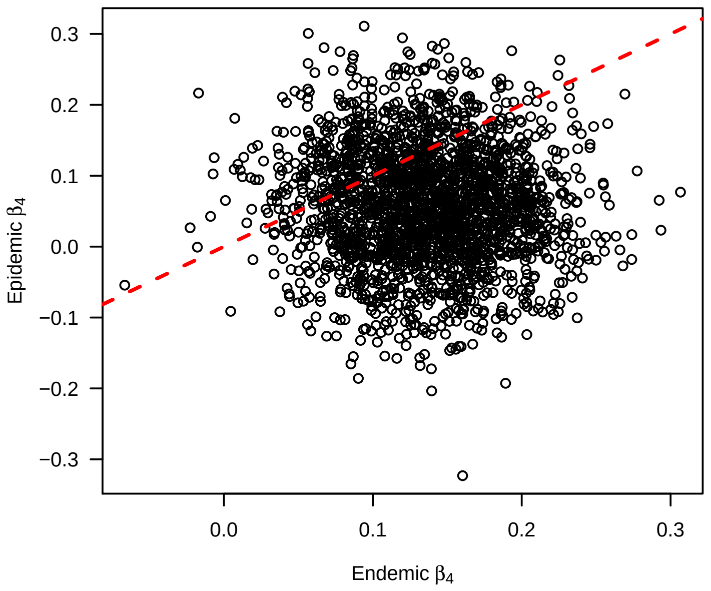

S1, Figure 6 : Posterior Samples of σ Across Regimes

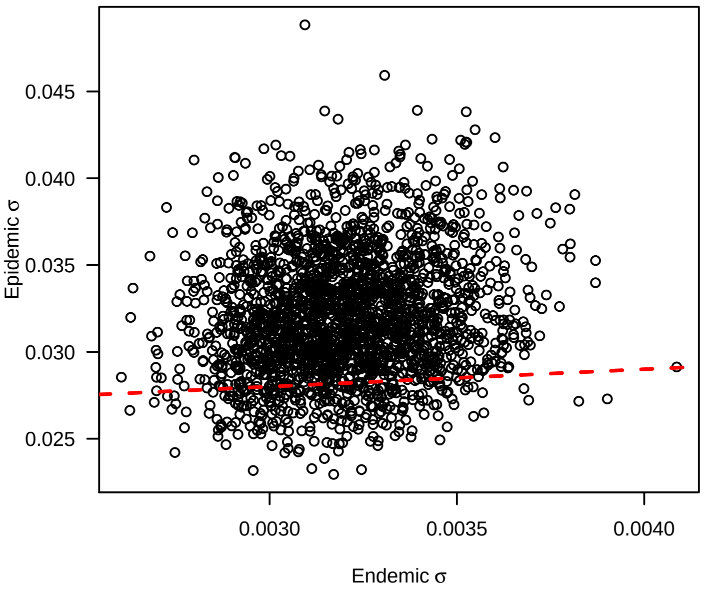

S2, Figure 7 : Autocorrelation of BSR2, Endemic Regime

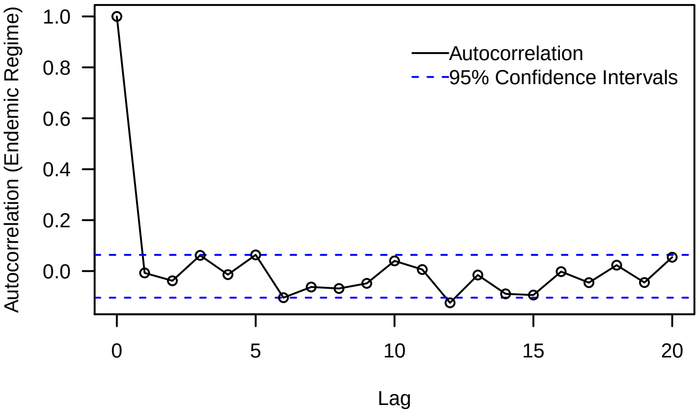

S2, Figure 8 : Autocorrelation of BSR2, Epidemic Regime

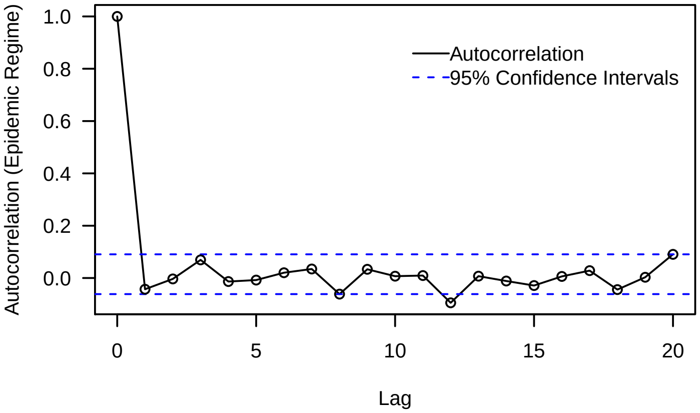

S2, Figure 9 : Autocorrelation of BSR3, Endemic Regime

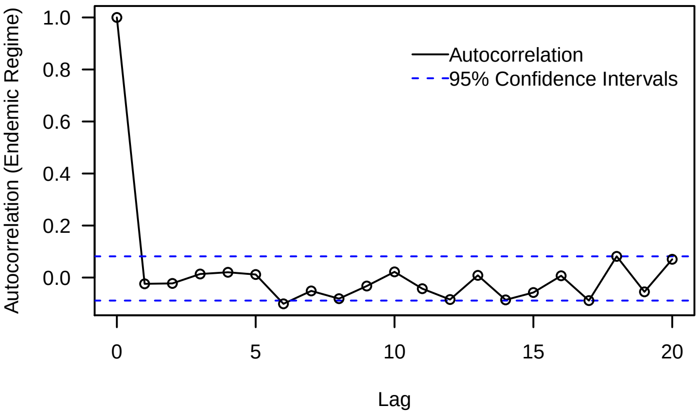

S2, Figure 10 : Autocorrelation of BSR3 Across, Regime

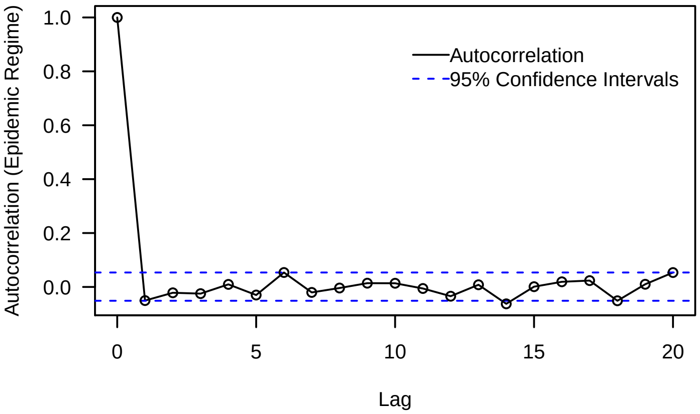

S2, Figure 11 : Autocorrelation of BAR2

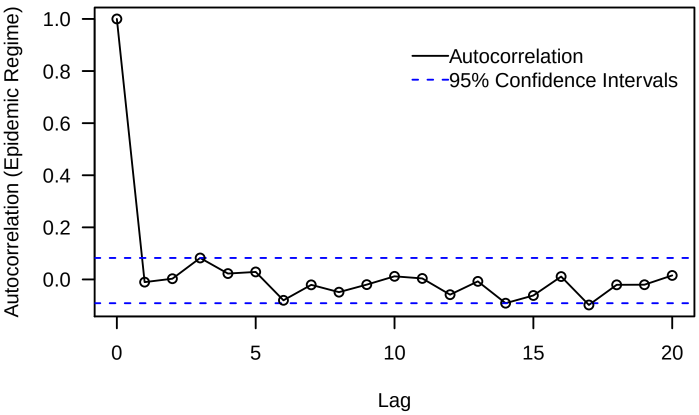

S2, Figure 12 : Autocorrelation of BAR3

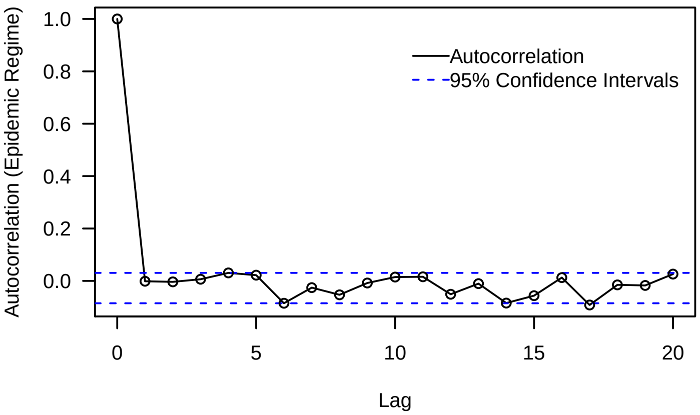

S2, Figure 13 : Autocorrelation of BAR4

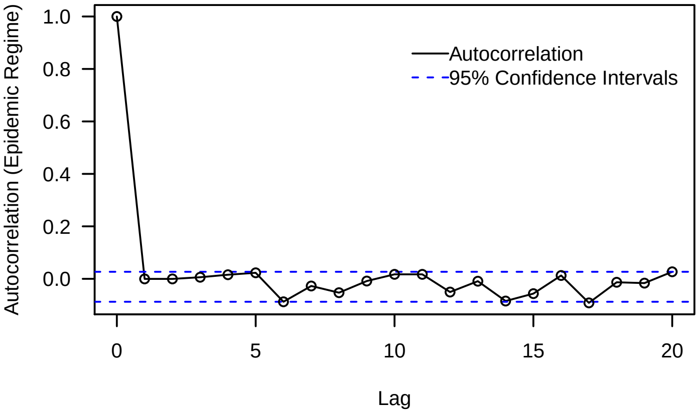

S3, Figure 14 : Convergence Plot of BSR3 Endemic β_1

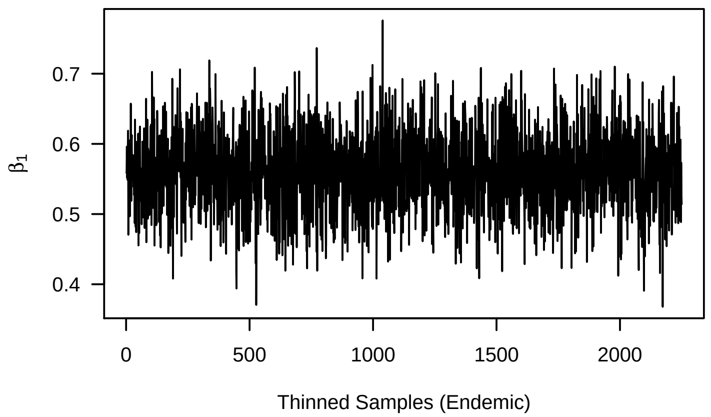

S3, Figure 15 : Convergence Plot of BSR3 Endemic β_2

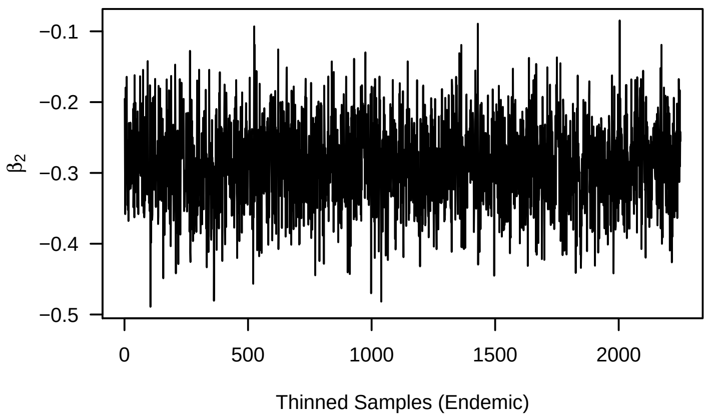

S3, Figure 16 : Convergence Plot of BSR3 Endemic β_3

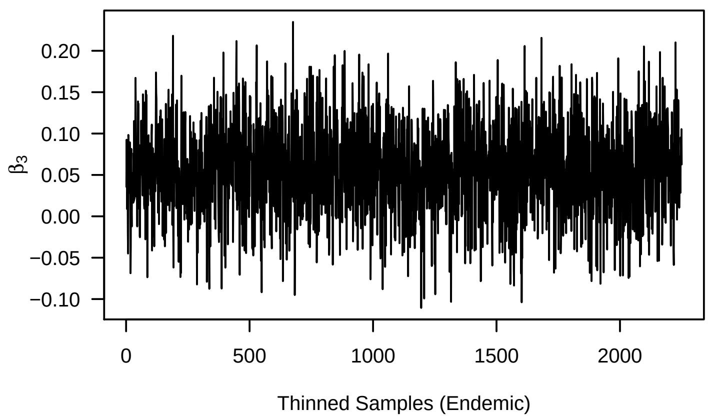

S3, Figure 17 : Convergence Plot of BSR3 Endemic β_4

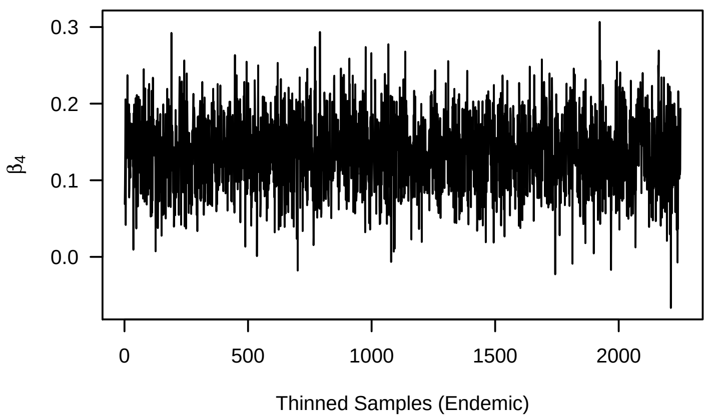

S3, Figure 18 : Convergence Plot of BSR3 Epidemic β_1

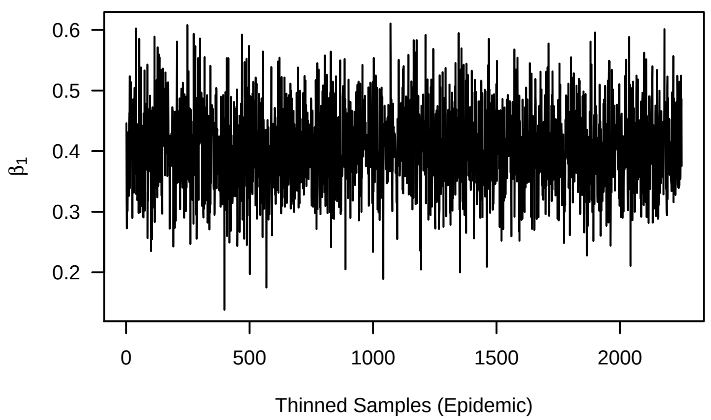

S3, Figure 19 : Convergence Plot of BSR3 Epidemic β_2

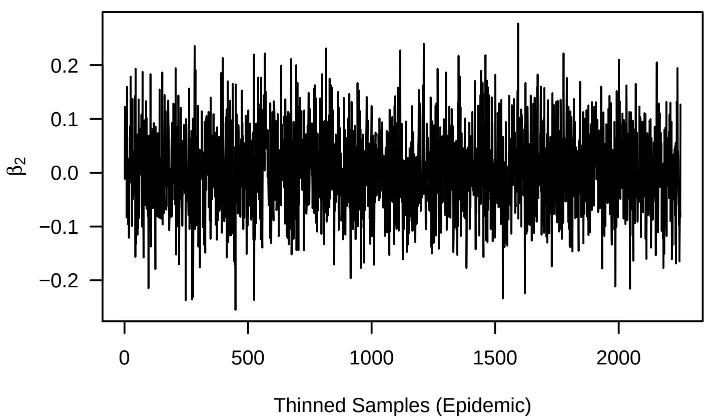

S3, Figure 20 : Convergence Plot of BSR3 Epidemic β_3

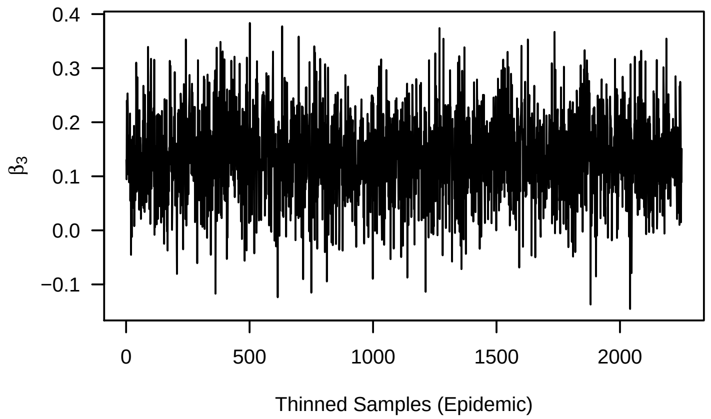

S3, Figure 21 : Convergence Plot of BSR3 Epidemic β_4

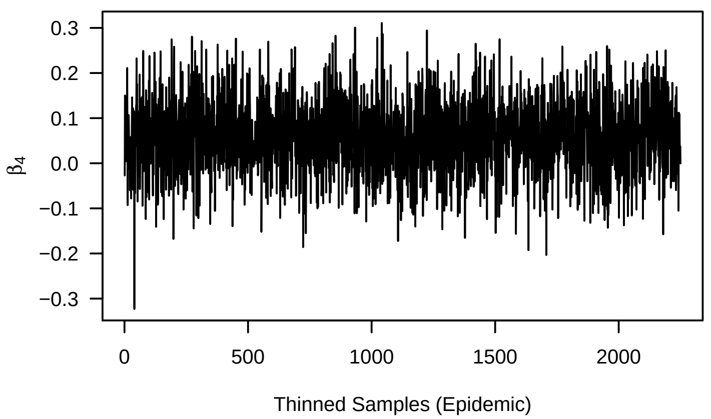

S3, Figure 22 : Convergence Plot of BSR2 Endemic β_1

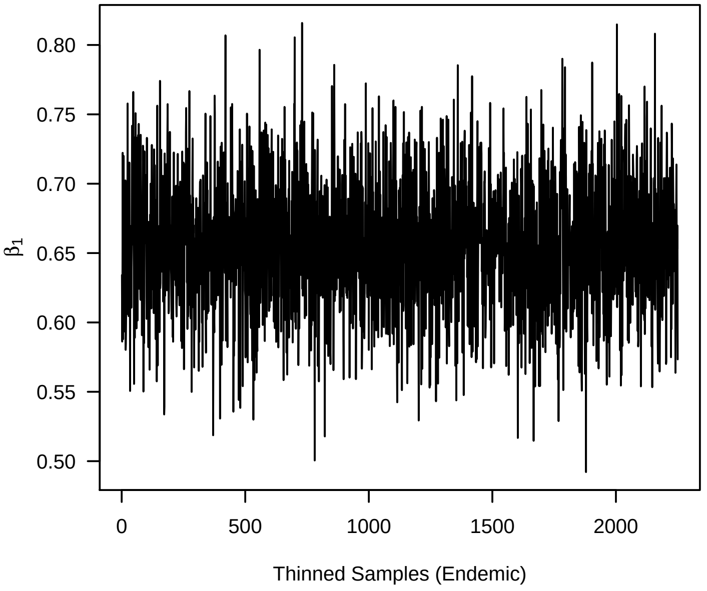

S3, Figure 23 : Convergence Plot of BSR2 Endemic β_2

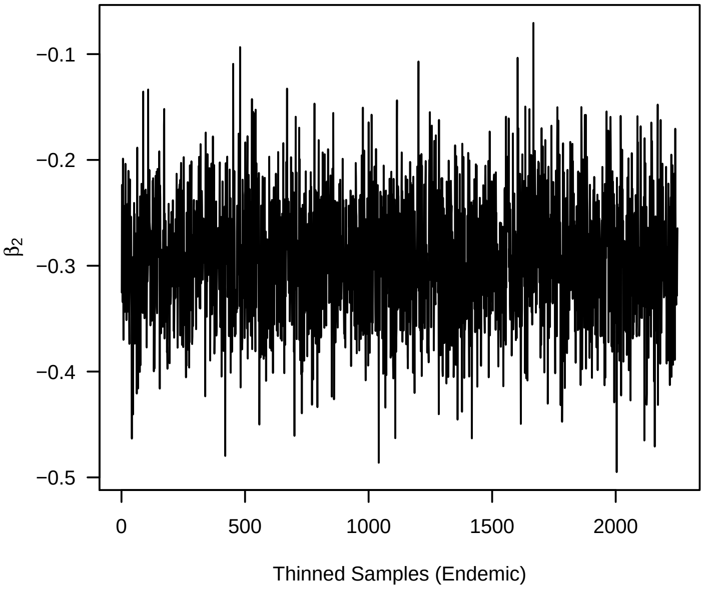

S3, Figure 24 : Convergence Plot of BSR2 Endemic β_3

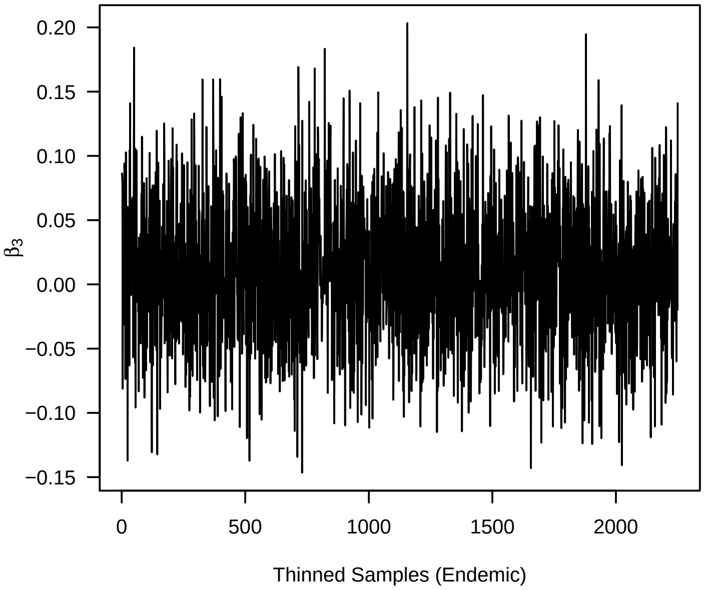

S3, Figure 25 : Convergence Plot of BSR2 Epidemic β_1

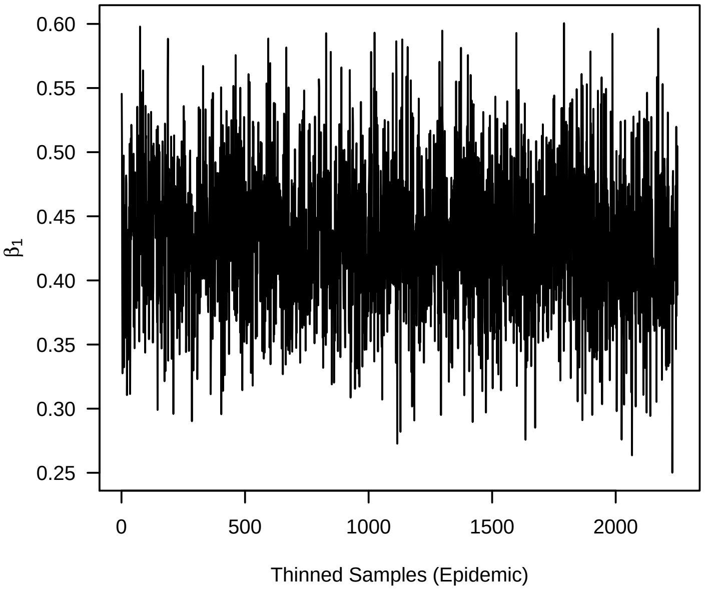

S3, Figure 26 : Convergence Plot of BSR2 Epidemic β_2

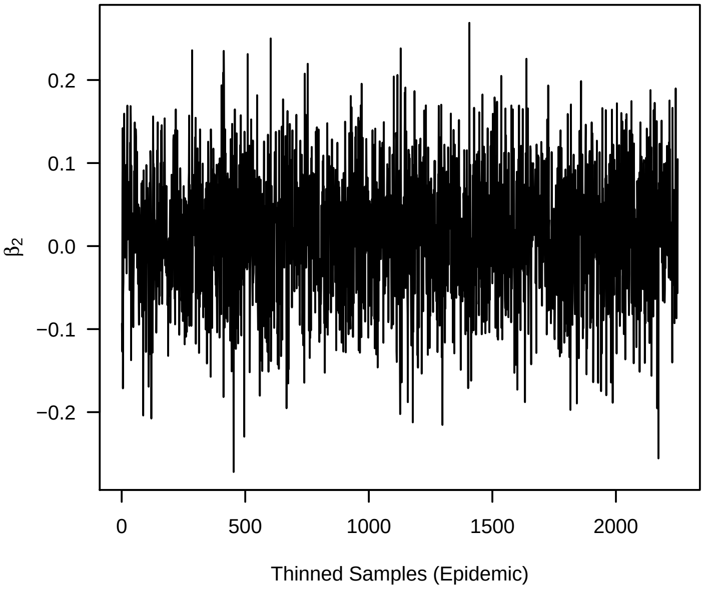

S3, Figure 27 : Convergence Plot of BSR2 Epidemic β_3

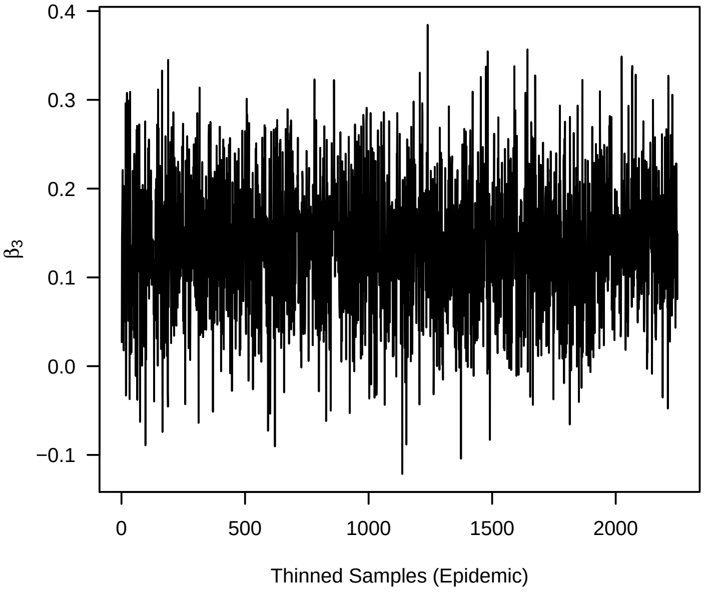

S4, Figure 28 : Prior−Posterior Plot of BSR3 Endemic β_1

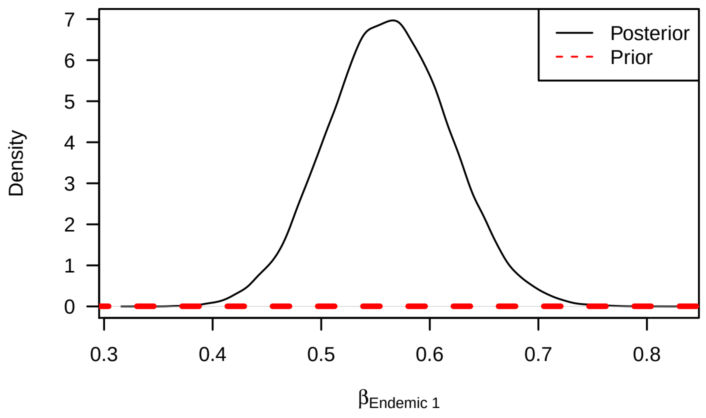

S4, Figure 29 : Prior−Posterior Plot of BSR3 Endemic β_2

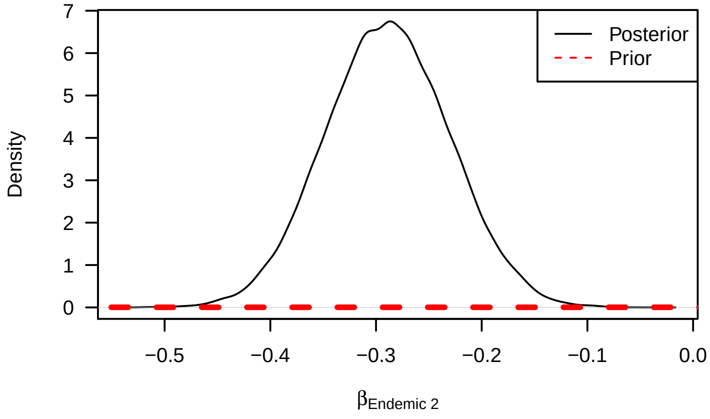

S4, Figure 30 : Prior−Posterior Plot of BSR3 Endemic β_3

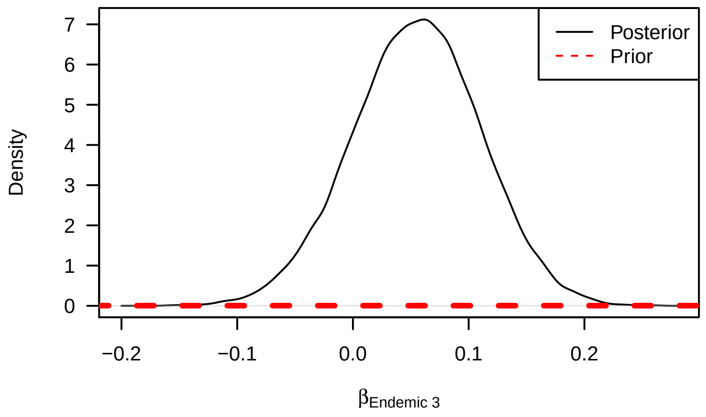

S4, Figure 31 : Prior−Posterior Plot of BSR3 Endemic β_4

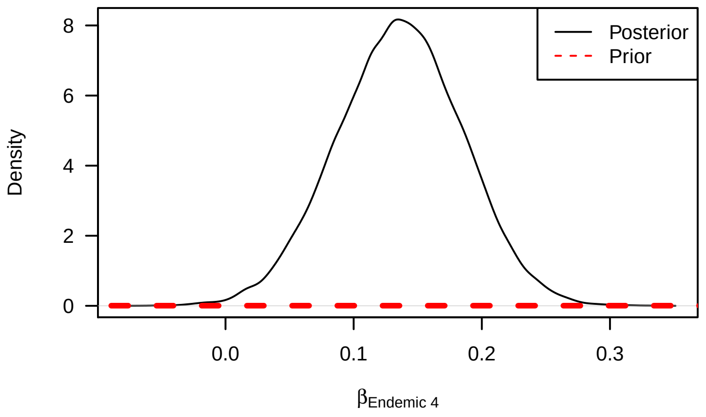

S4, Figure 32 : Prior−Posterior Plot of BSR3 Epidemic β_1

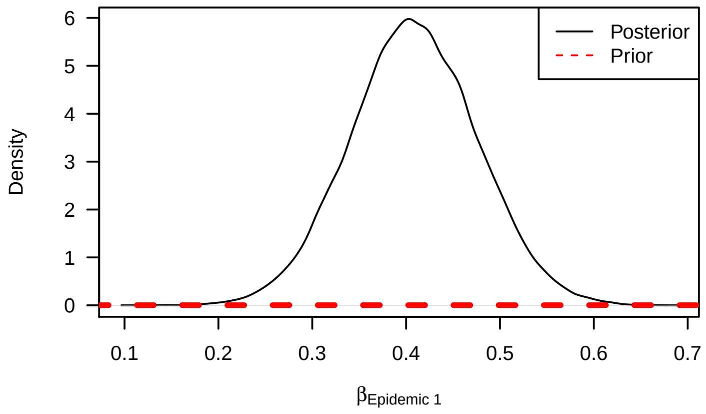

S4, Figure 33 : Prior−Posterior Plot of BSR3 Epidemic β_2

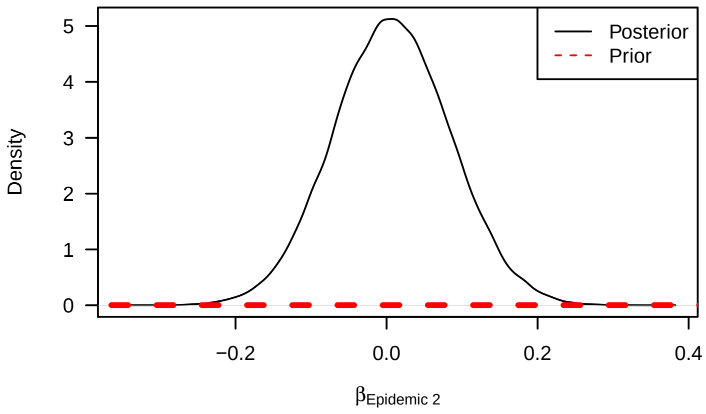

S4, Figure 34 : Prior−Posterior Plot of BSR3 Epidemic β_3

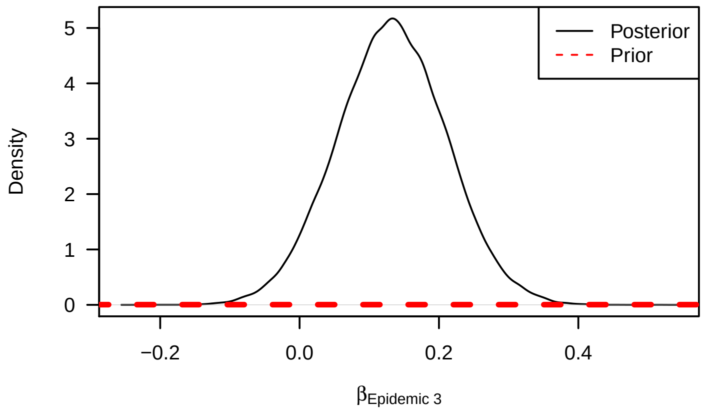

S4, Figure 35 : Prior−Posterior Plot of BSR3 Epidemic β_4

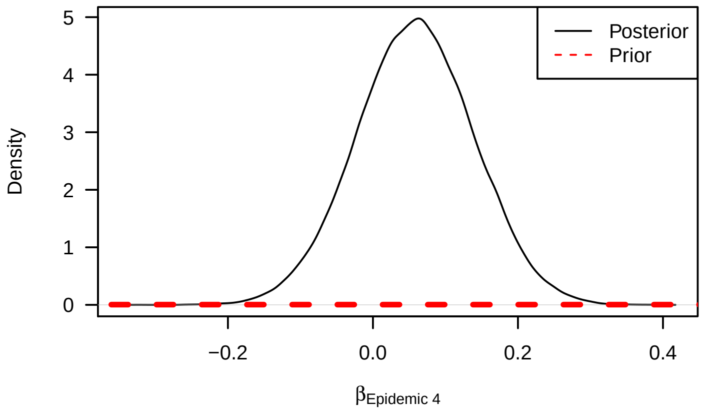

S4, Figure 36 : Prior−Posterior Plot of BSR2 Endemic β_1

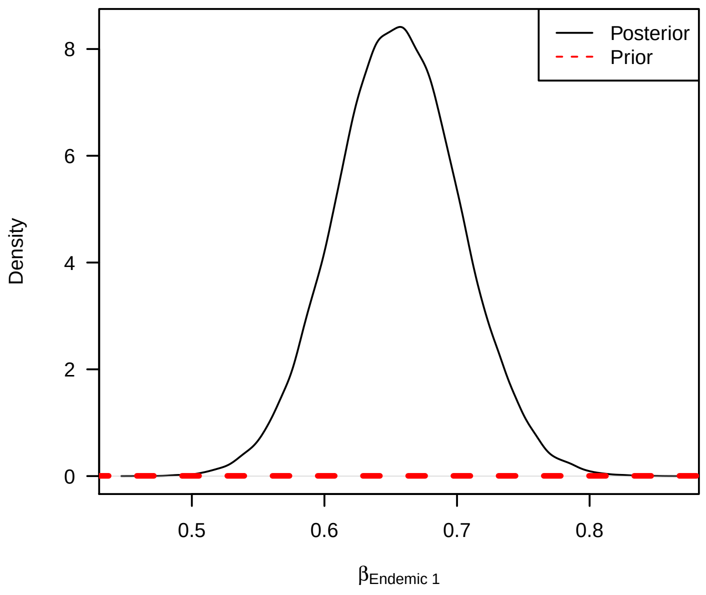

S4, Figure 37 : Prior−Posterior Plot of BSR2 Endemic β_2

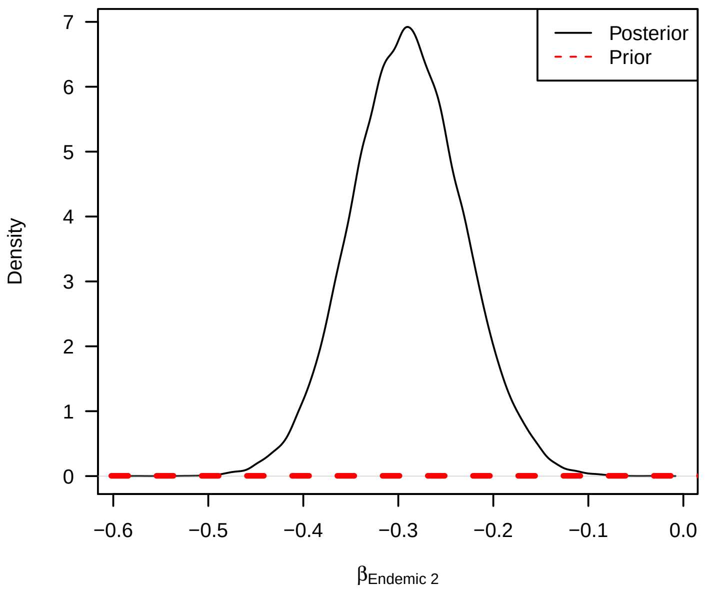

S4, Figure 38 : Prior−Posterior Plot of BSR2 Endemic β_3

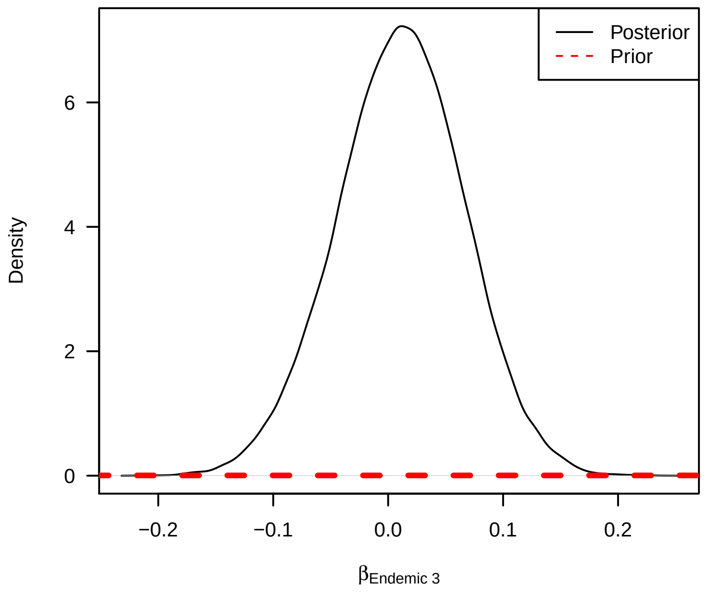

S4, Figure 39 : Prior−Posterior Plot of BSR2 Epidemic β_1

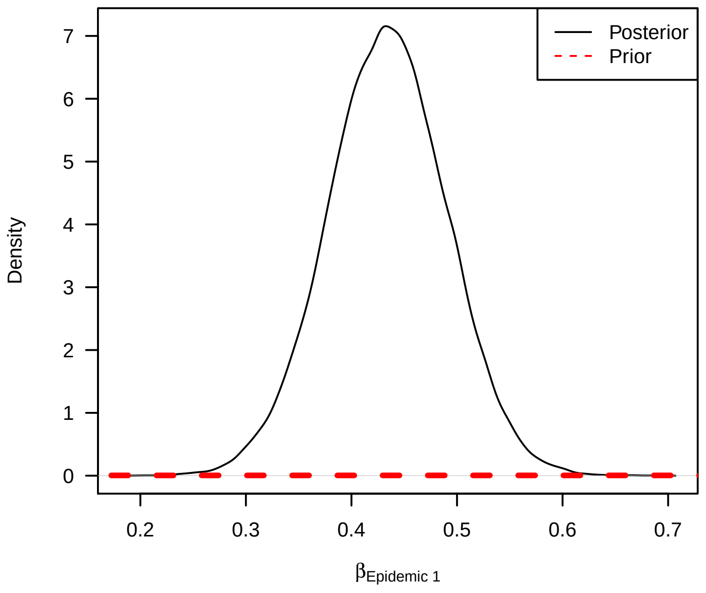

S4, Figure 40 : Prior−Posterior Plot of BSR2 Epidemic β_2

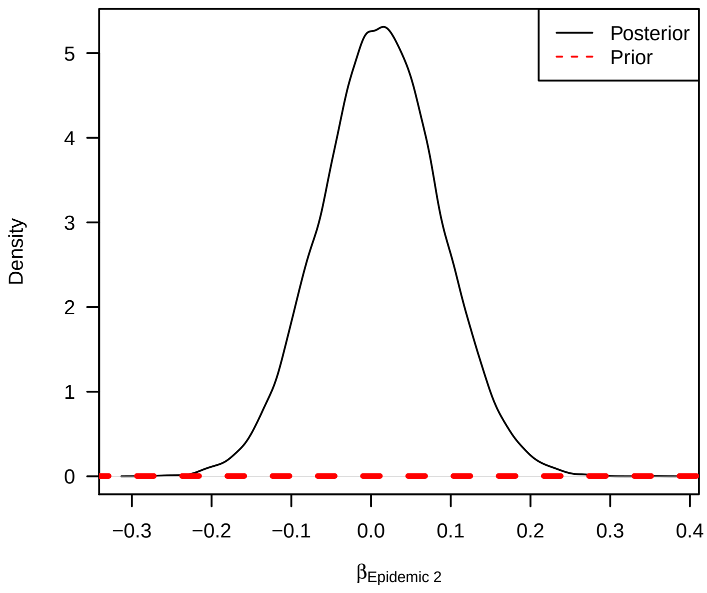

S4, Figure 41 : Prior−Posterior Plot of BSR2 Epidemic β_3

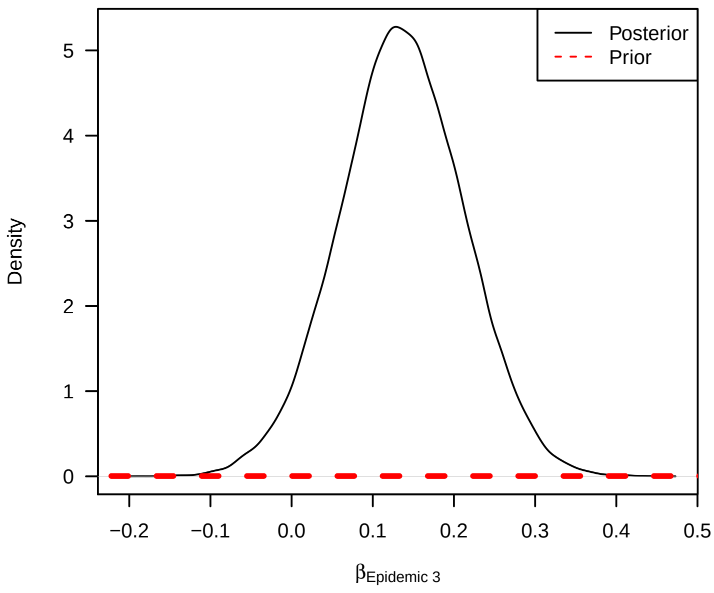

S5, Figure 42 : Bootstrap Distribution of Dewpoint Temperature lag 1

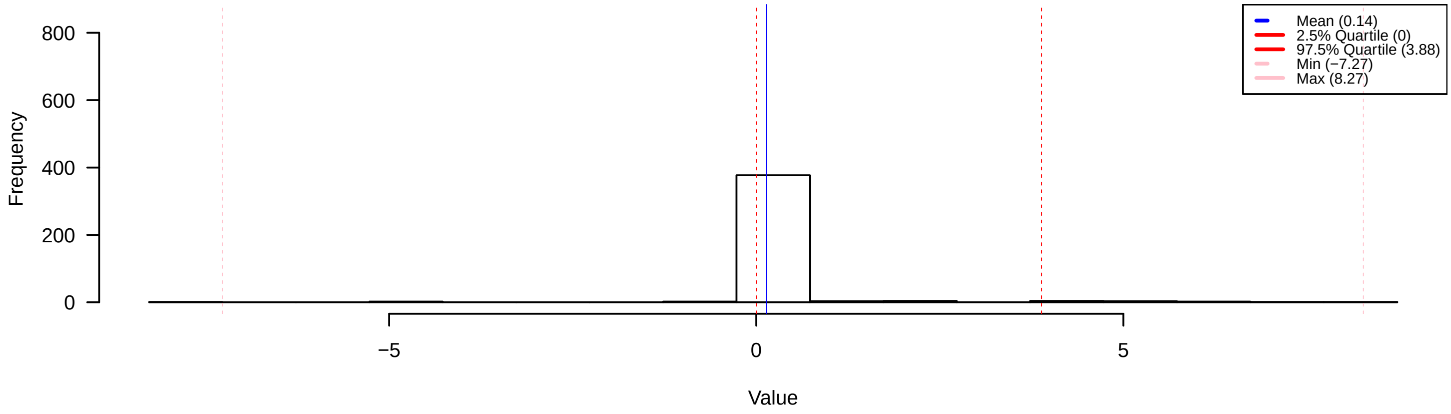

S5, Figure 43 : Bootstrap Distribution of Dewpoint Temperature lag 2

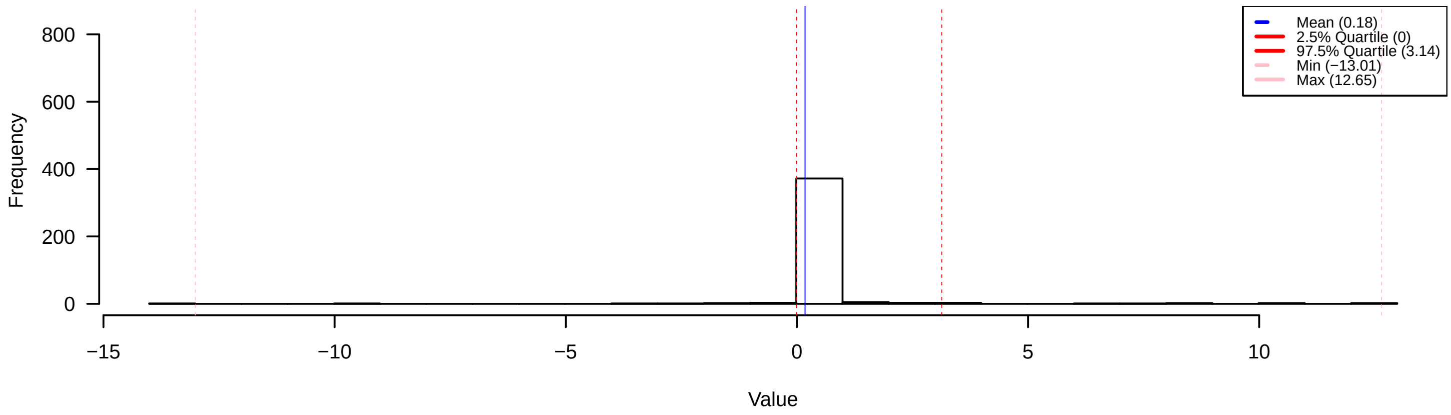

S5, Figure 44 : Bootstrap Distribution of Dewpoint Temperature lag 3

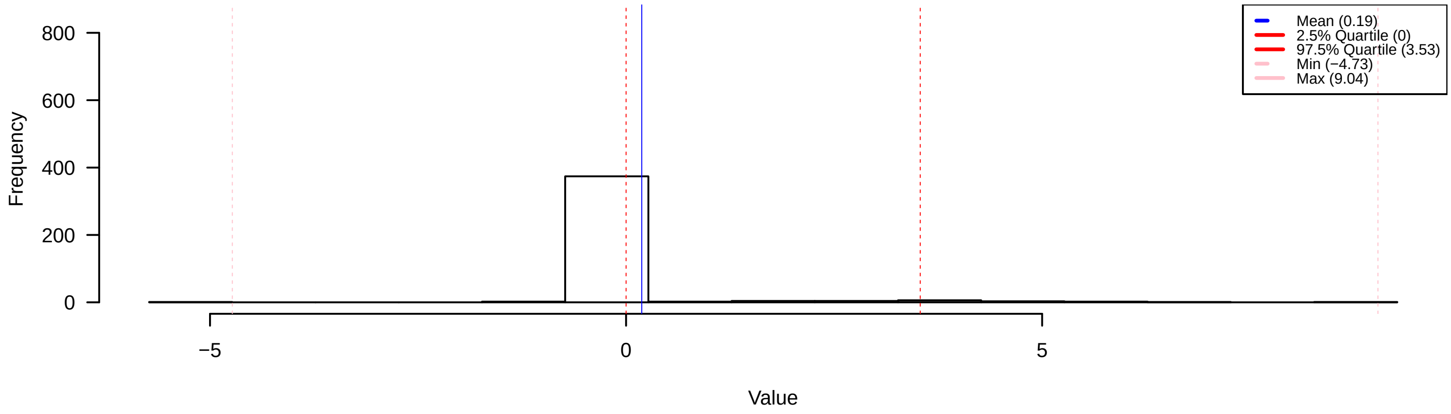

S5, Figure 45 : Bootstrap Distribution of Dewpoint Temperature lag 4

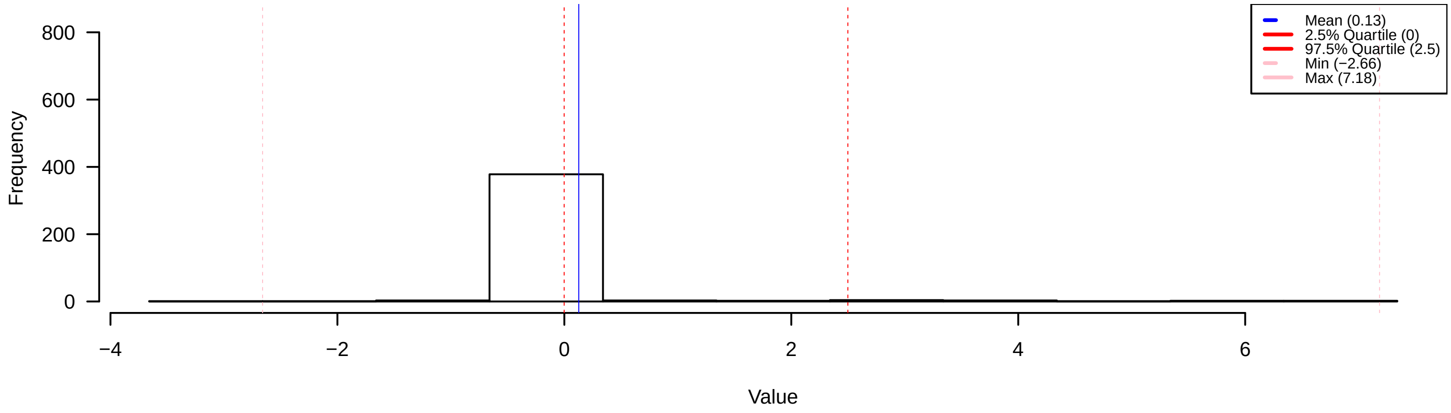

S5, Figure 46 : Bootstrap Distribution of Dewpoint Temperature lag 5

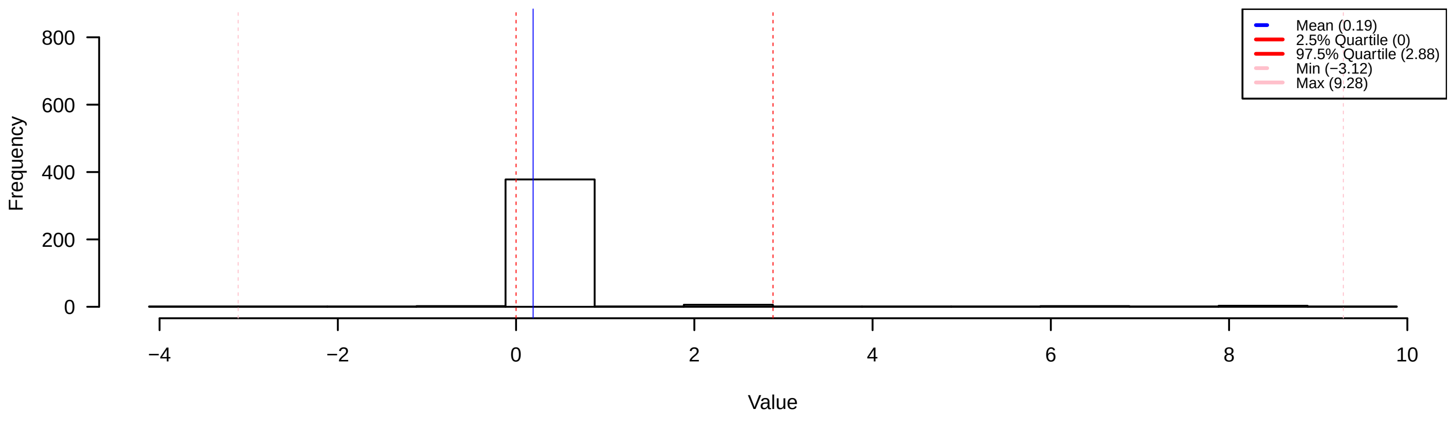

S5, Figure 47 : Bootstrap Distribution of Dewpoint Temperature lag 6

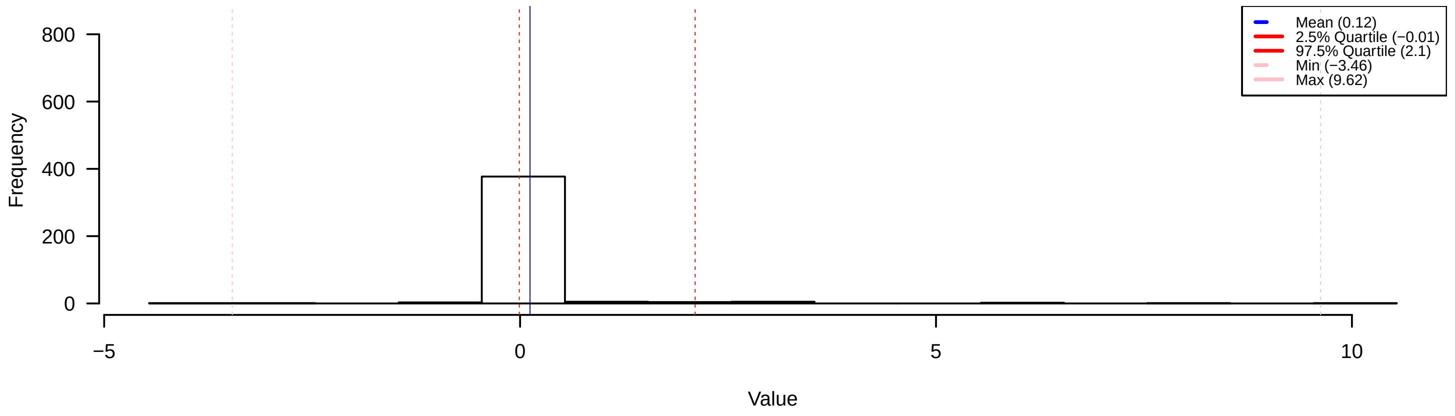

S5, Figure 48 : Bootstrap Distribution of Dewpoint Temperature lag 7

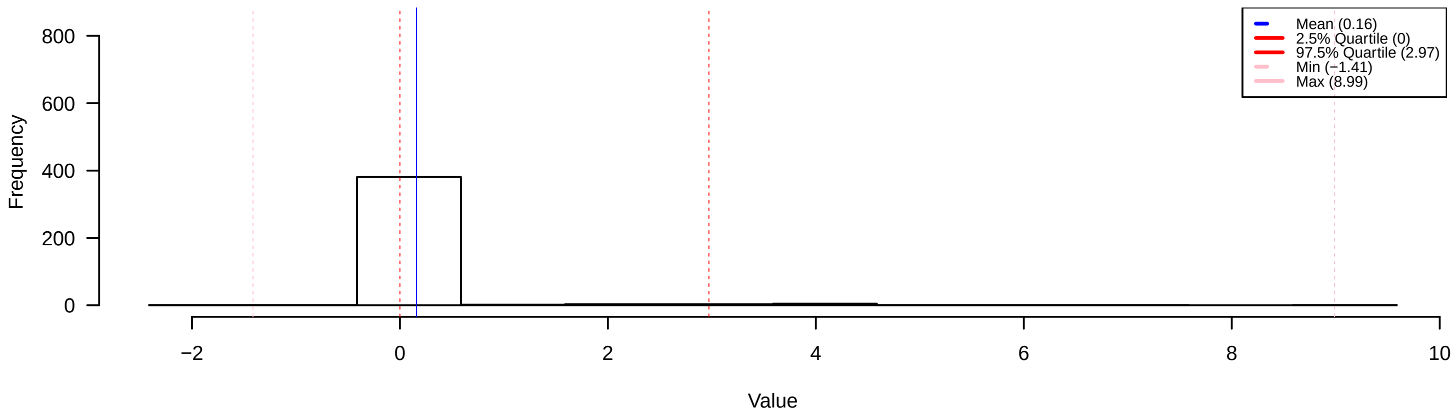

S5, Figure 49 : Bootstrap Distribution of Dewpoint Temperature lag 8

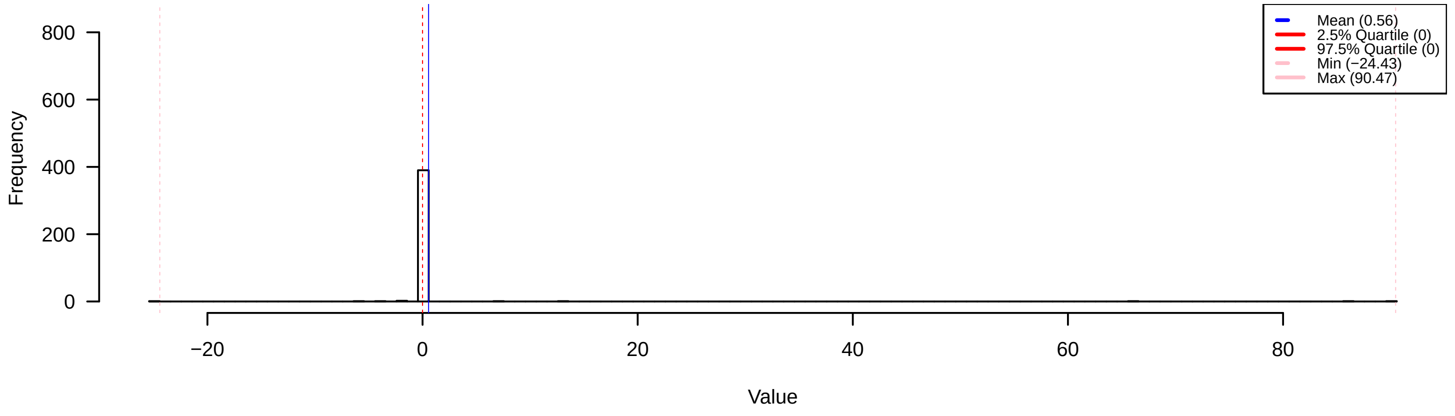

S5, Figure 50 : Bootstrap Distribution of Dewpoint Temperature lag 9

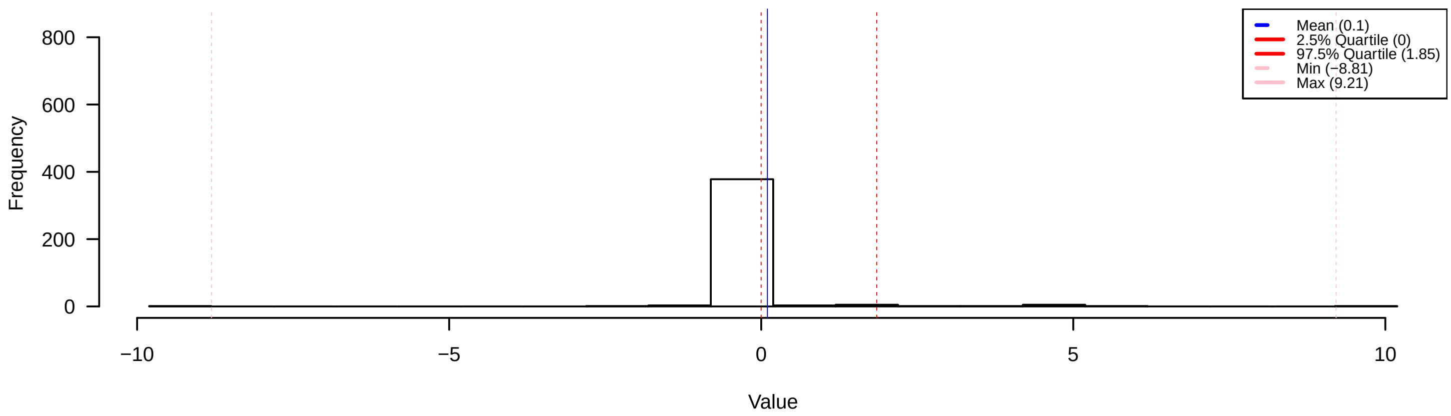

S5, Figure 51 : Bootstrap Distribution of Dewpoint Temperature lag 10

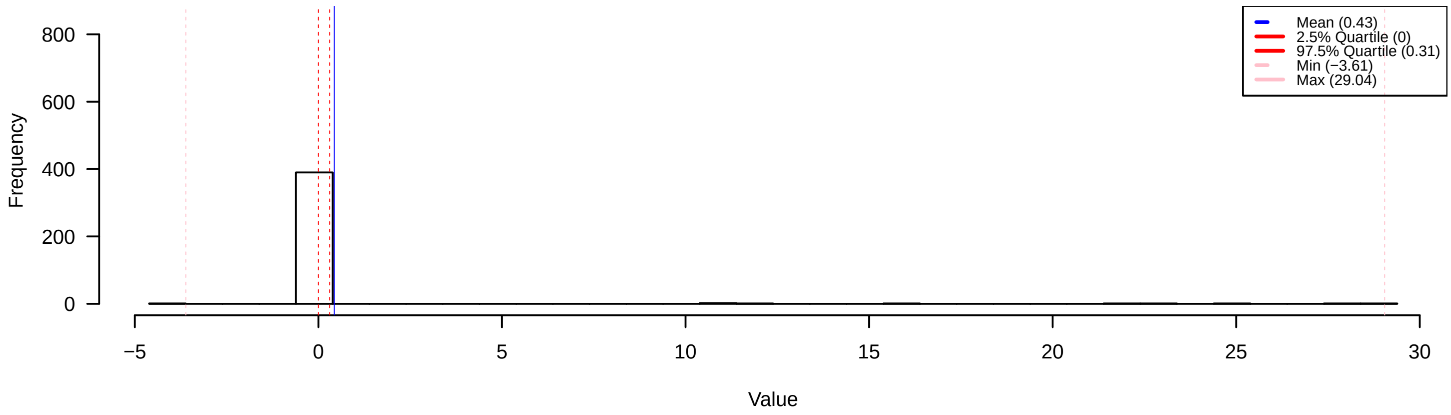

S5, Figure 52 : Bootstrap Distribution of Dewpoint Temperature lag 11

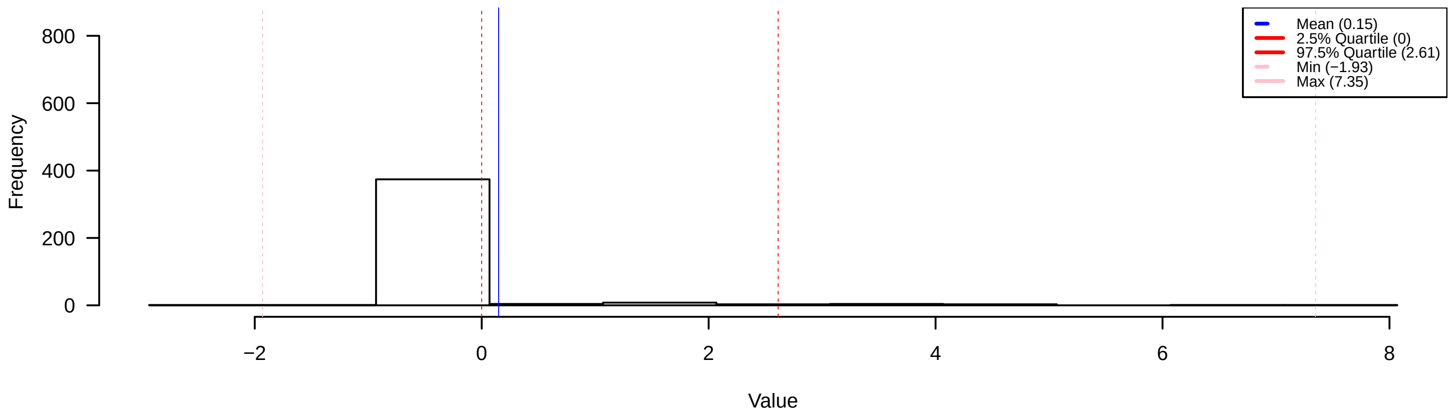

S5, Figure 53 : Bootstrap Distribution of Dewpoint Temperature lag 12

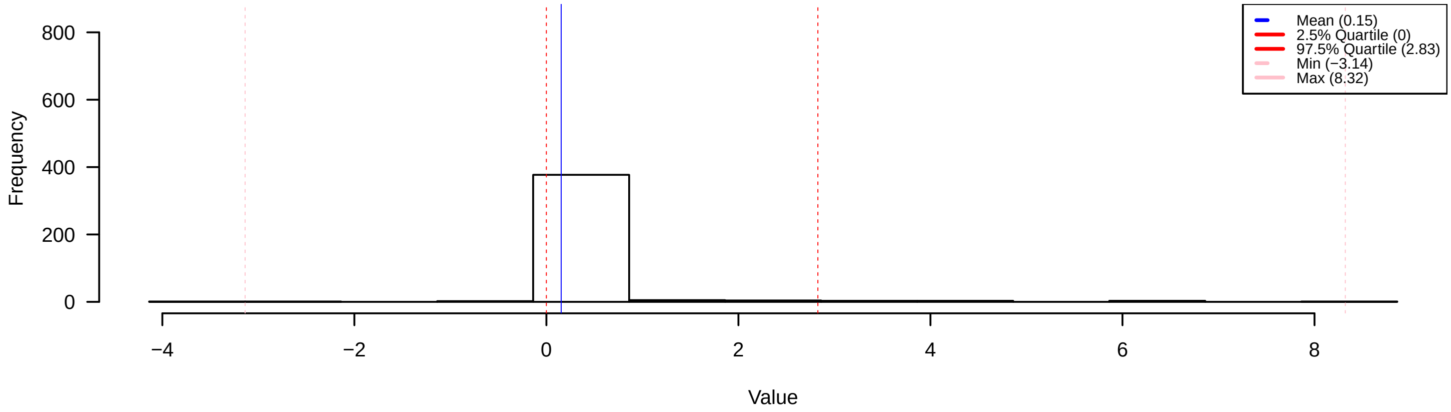

S5, Figure 54 : Bootstrap Distribution of Dewpoint Temperature lag 13

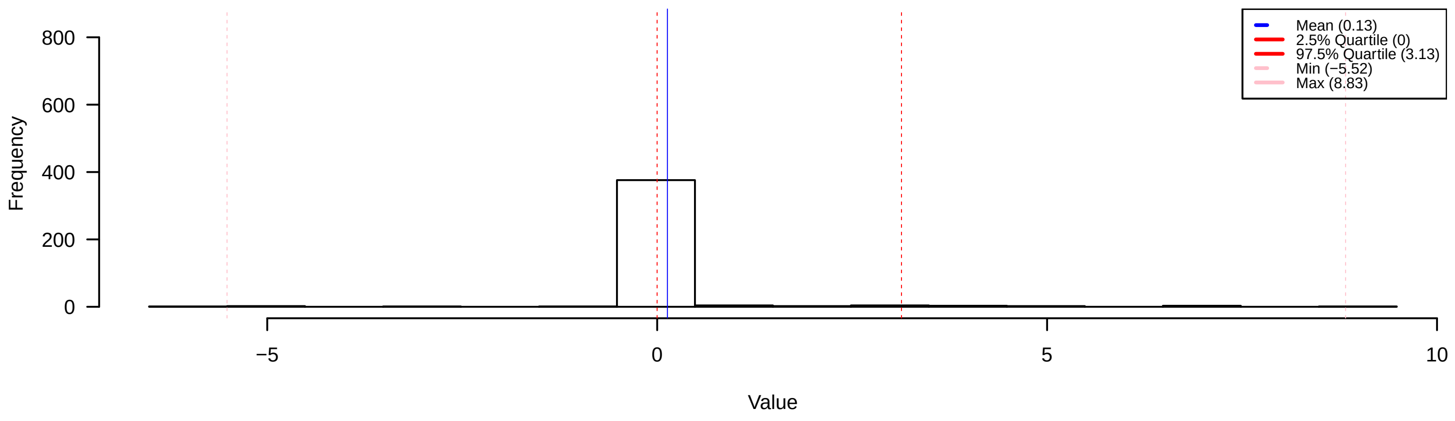

S5, Figure 55 : Bootstrap Distribution of Dewpoint Temperature lag 14

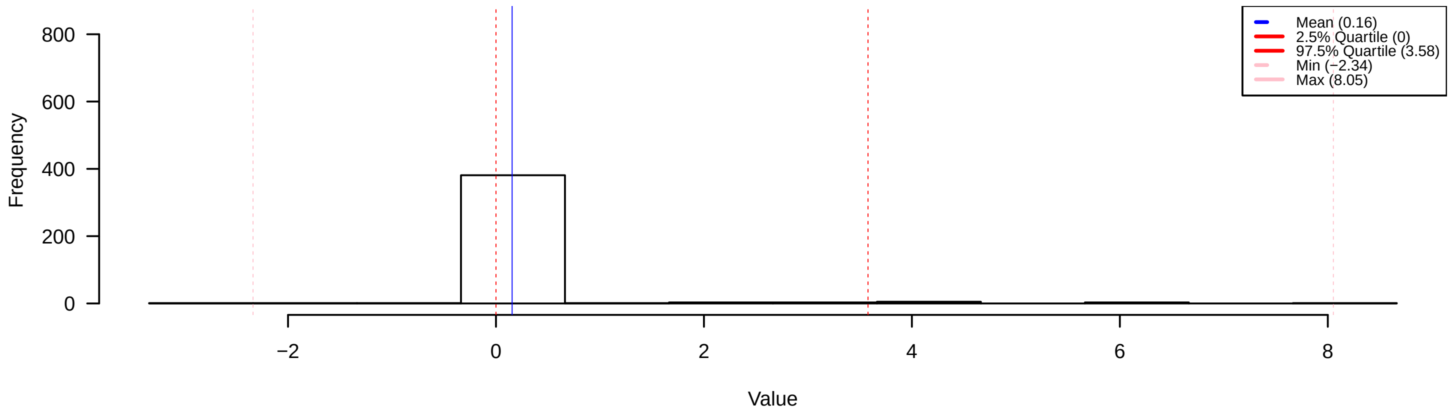

S5, Figure 56 : Bootstrap Distribution of Dewpoint Temperature lag 15

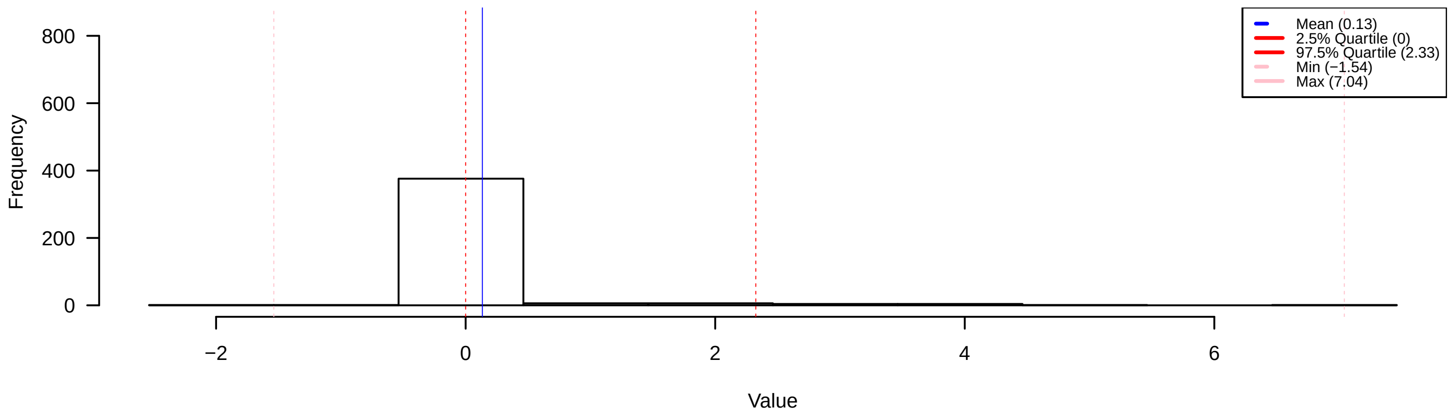

S5, Figure 57 : Bootstrap Distribution of Dewpoint Temperature lag 16

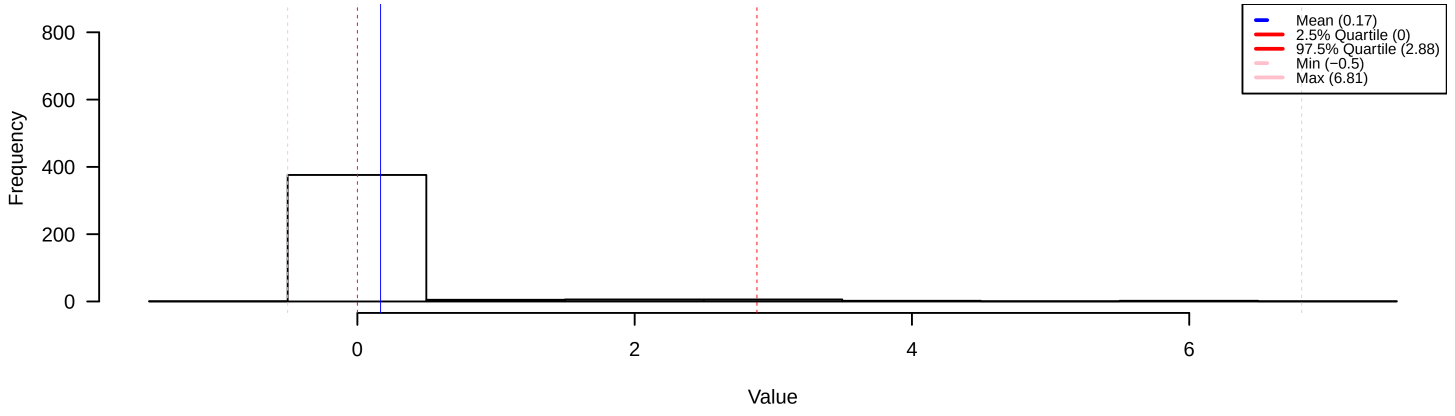

S5, Figure 58 : Bootstrap Distribution of Dewpoint Temperature lag 17

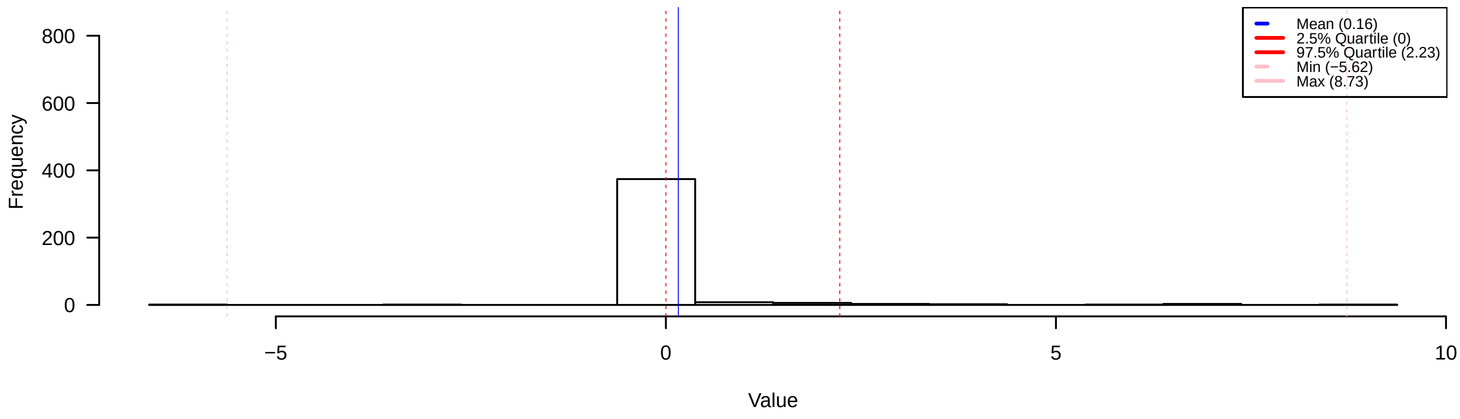

S5, Figure 59 : Bootstrap Distribution of Dewpoint Temperature lag 18

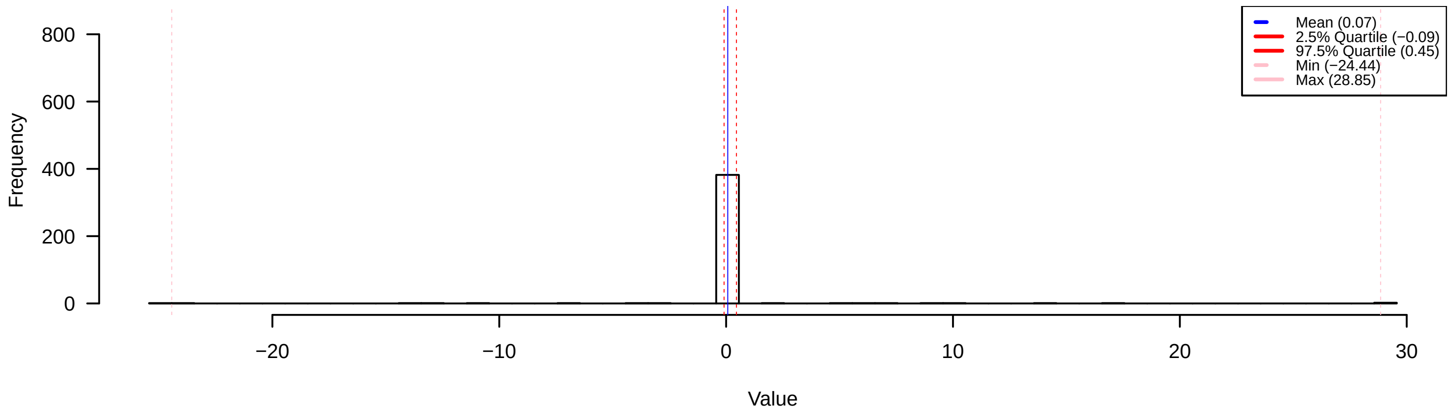

S5, Figure 60 : Bootstrap Distribution of Dewpoint Temperature lag 19

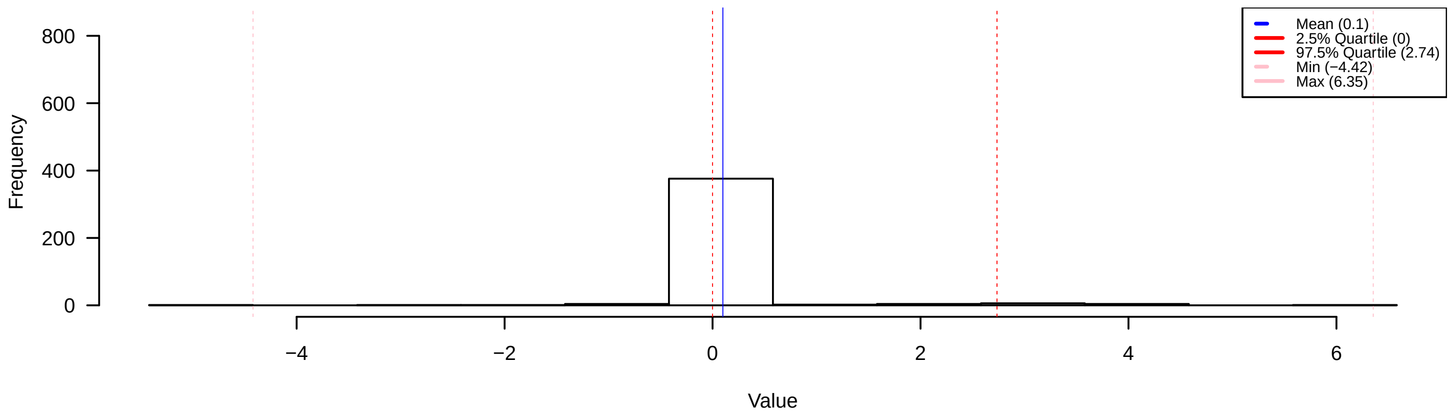

S5, Figure 61 : Bootstrap Distribution of Dewpoint Temperature lag 20

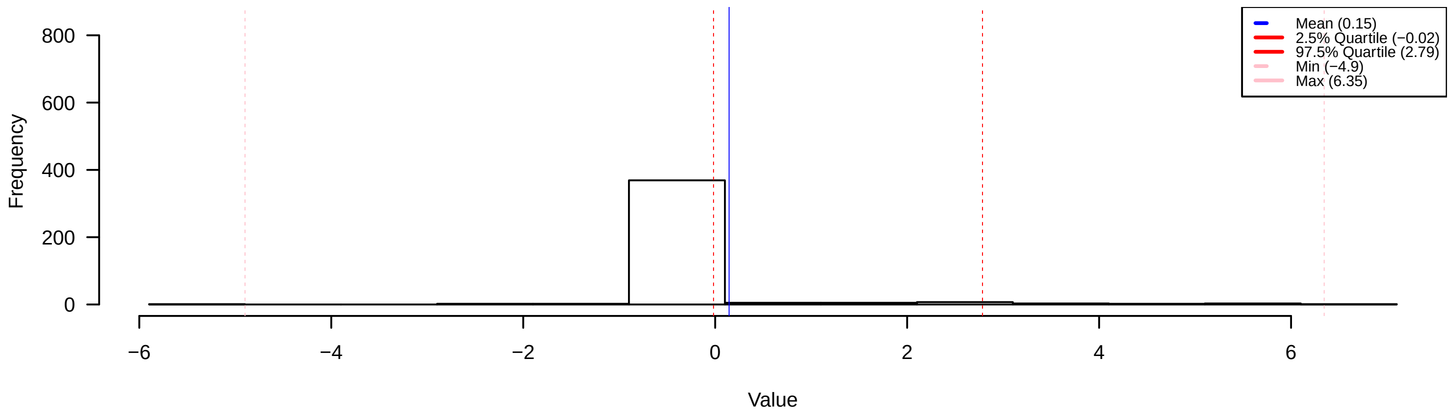

S5, Figure 62 : Bootstrap Distribution of Air Temperature lag 1

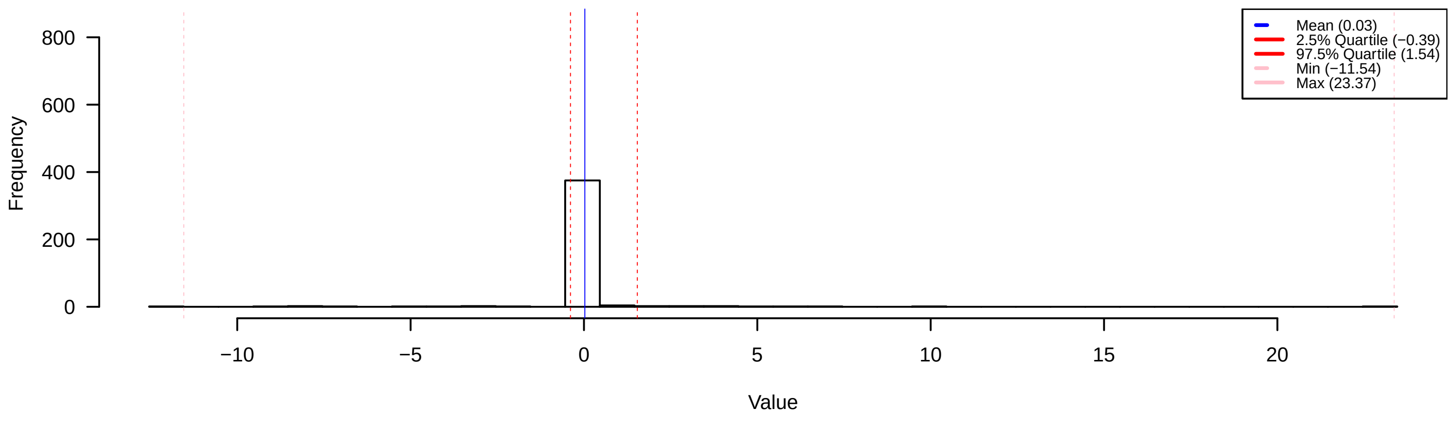

S5, Figure 63 : Bootstrap Distribution of Air Temperature lag 2

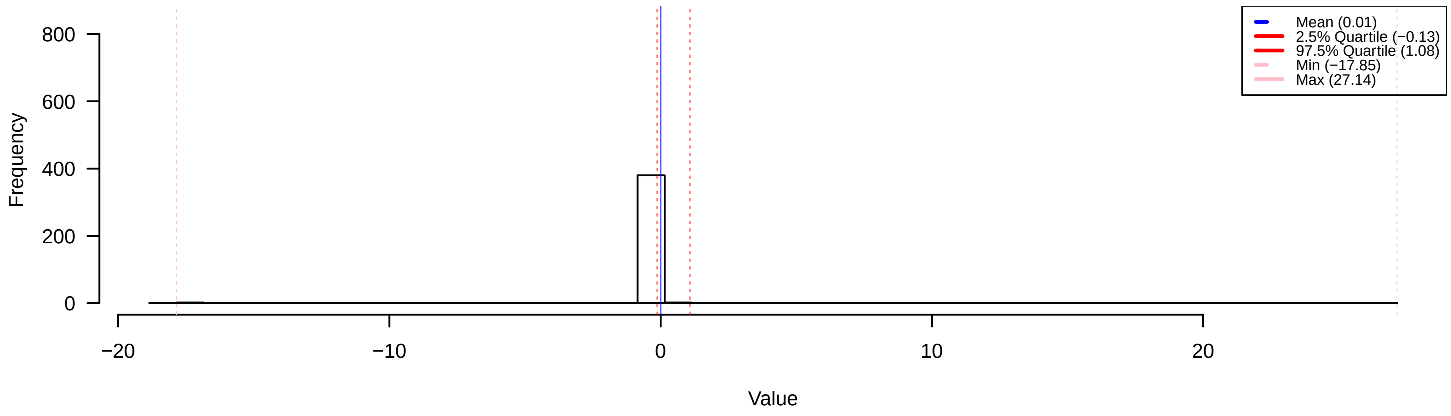

S5, Figure 64 : Bootstrap Distribution of Air Temperature lag 3

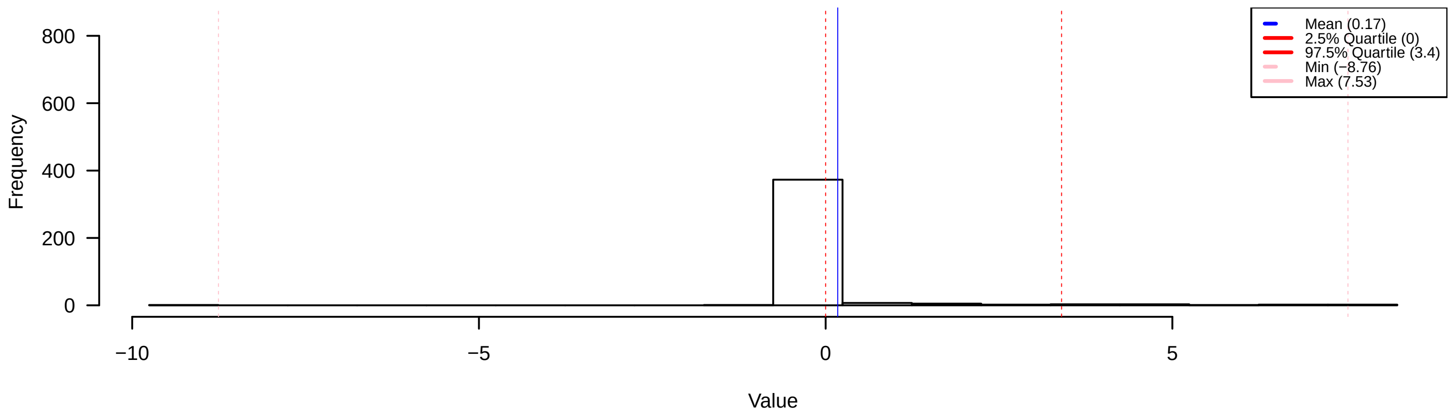

S5, Figure 65 : Bootstrap Distribution of Air Temperature lag 4

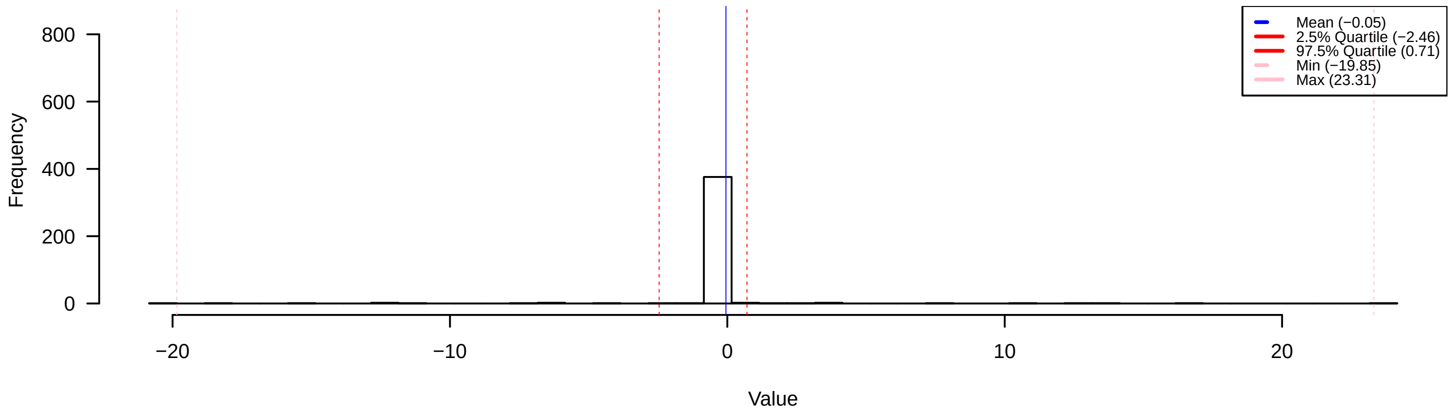

S5, Figure 66 : Bootstrap Distribution of Air Temperature lag 5

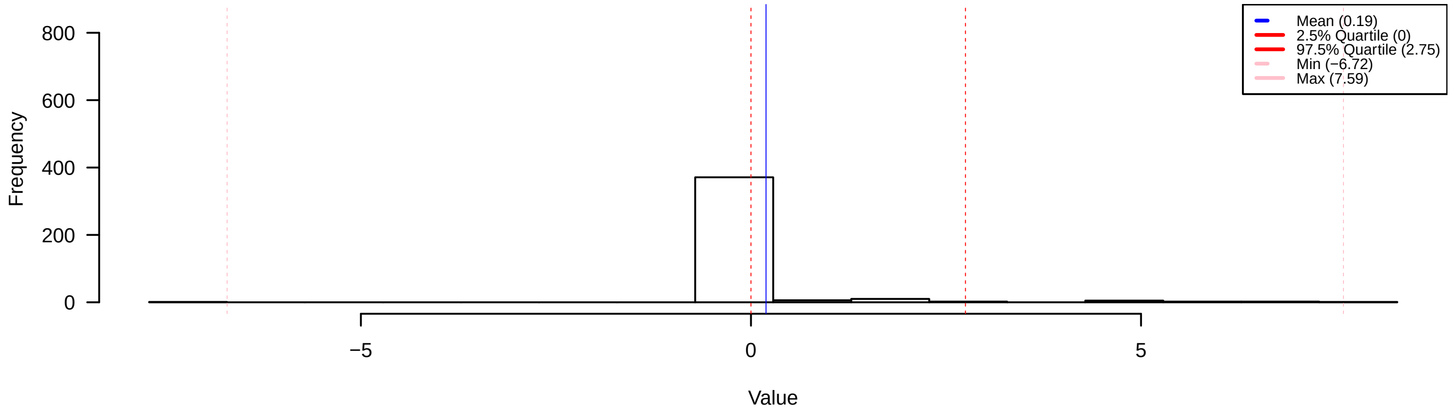

S5, Figure 67 : Bootstrap Distribution of Air Temperature lag 6

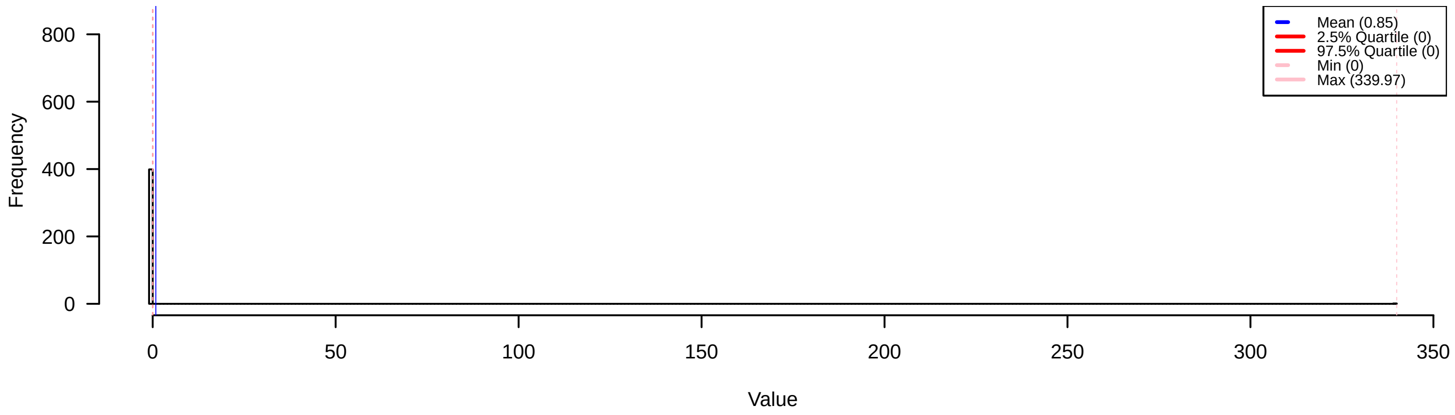

S5, Figure 68 : Bootstrap Distribution of Air Temperature lag 7

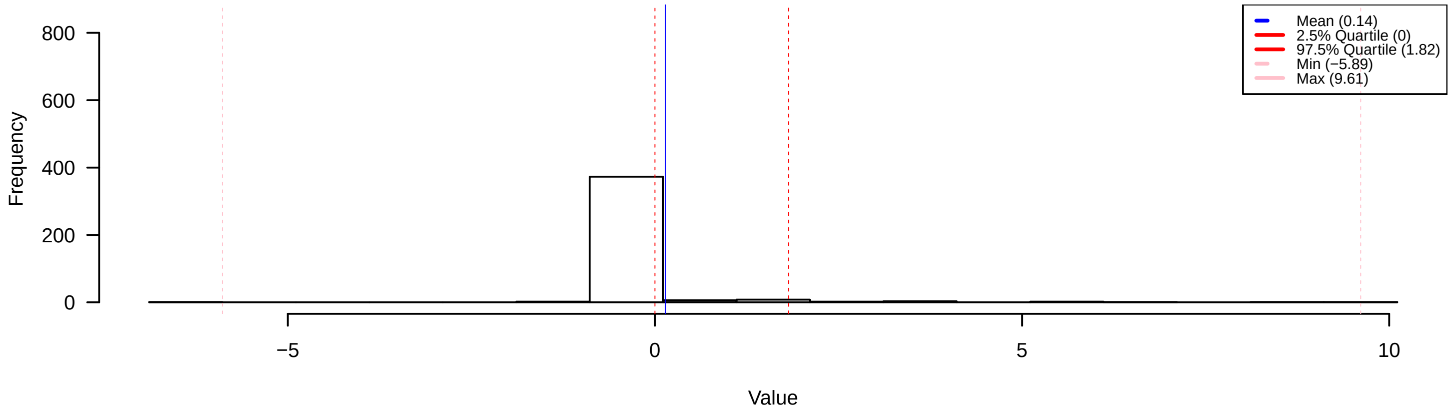

S5, Figure 69 : Bootstrap Distribution of Air Temperature lag 8

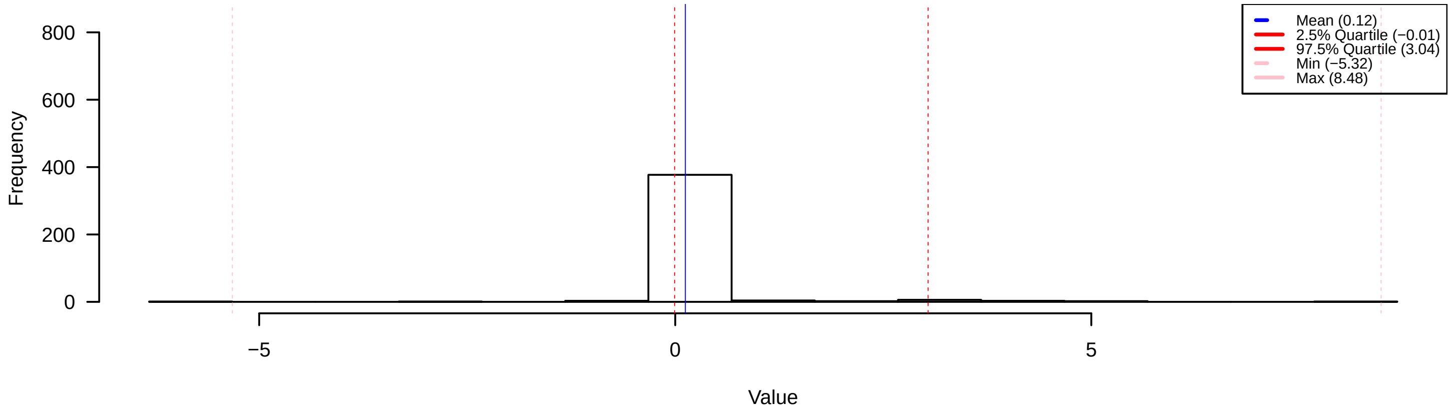

S5, Figure 70 : Bootstrap Distribution of Air Temperature lag 9

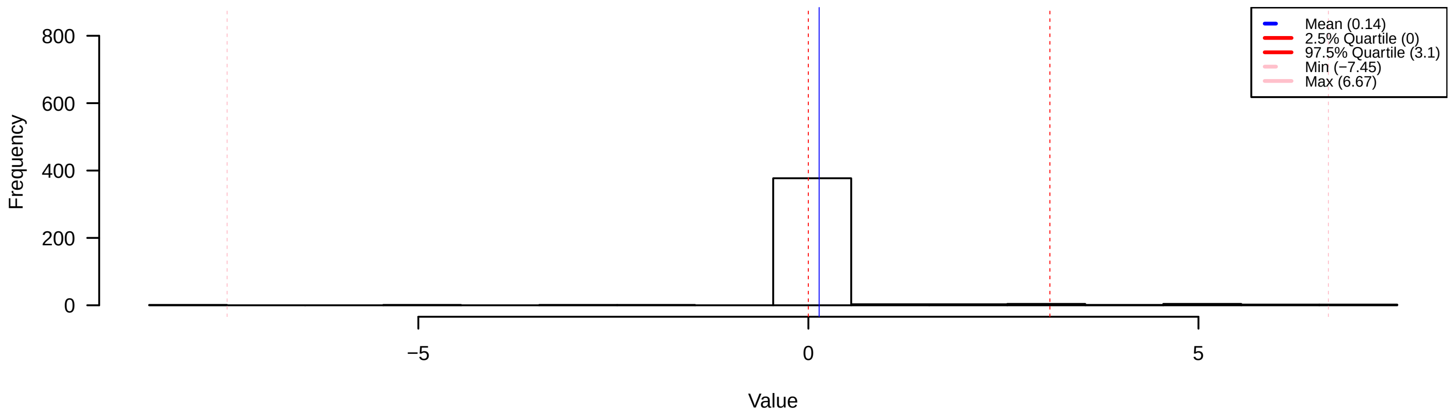

S5, Figure 71 : Bootstrap Distribution of Air Temperature lag 10

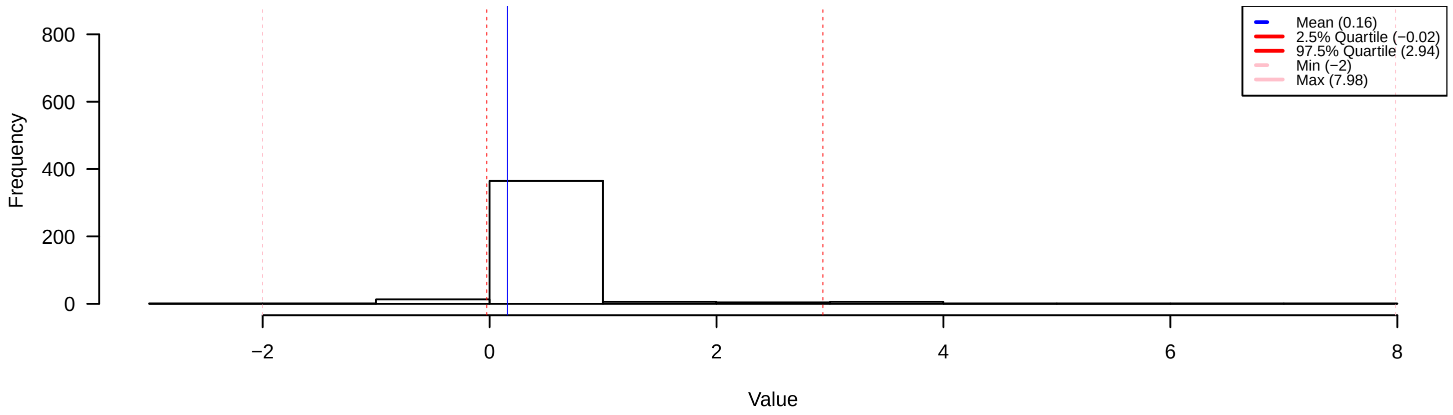

S5, Figure 72 : Bootstrap Distribution of Air Temperature lag 11

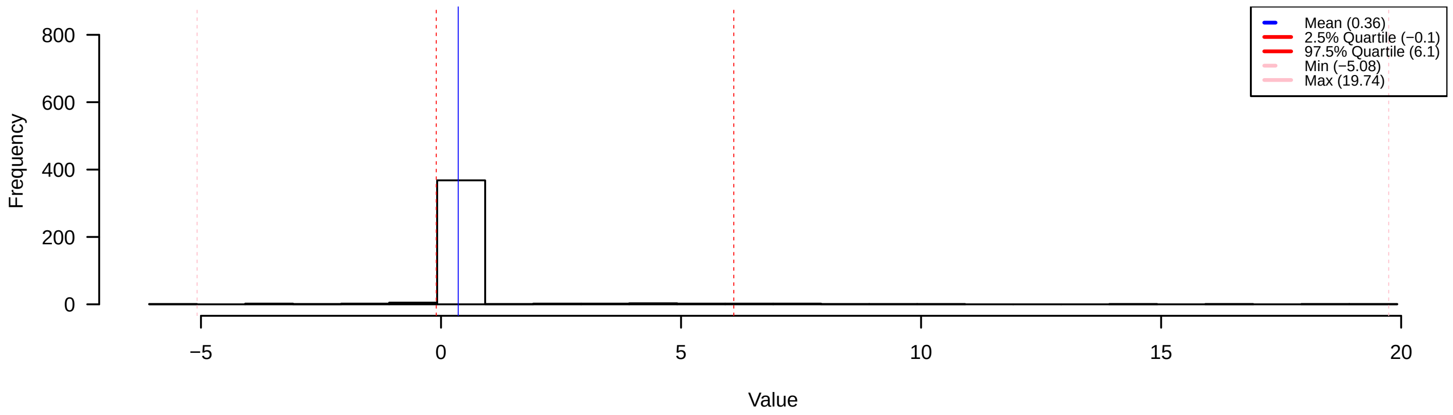

S5, Figure 73 : Bootstrap Distribution of Air Temperature lag 12

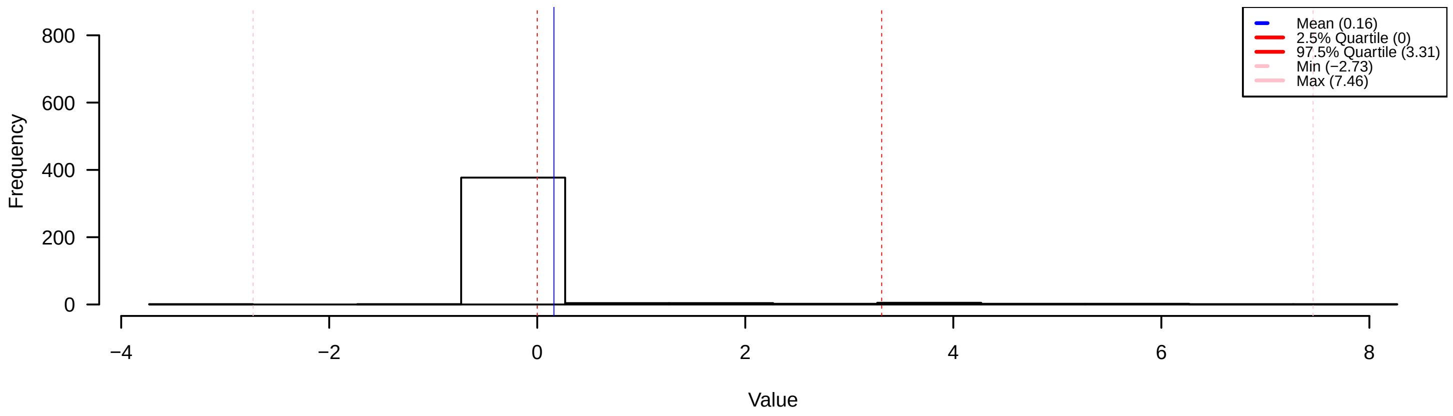

S5, Figure 74 : Bootstrap Distribution of Air Temperature lag 13

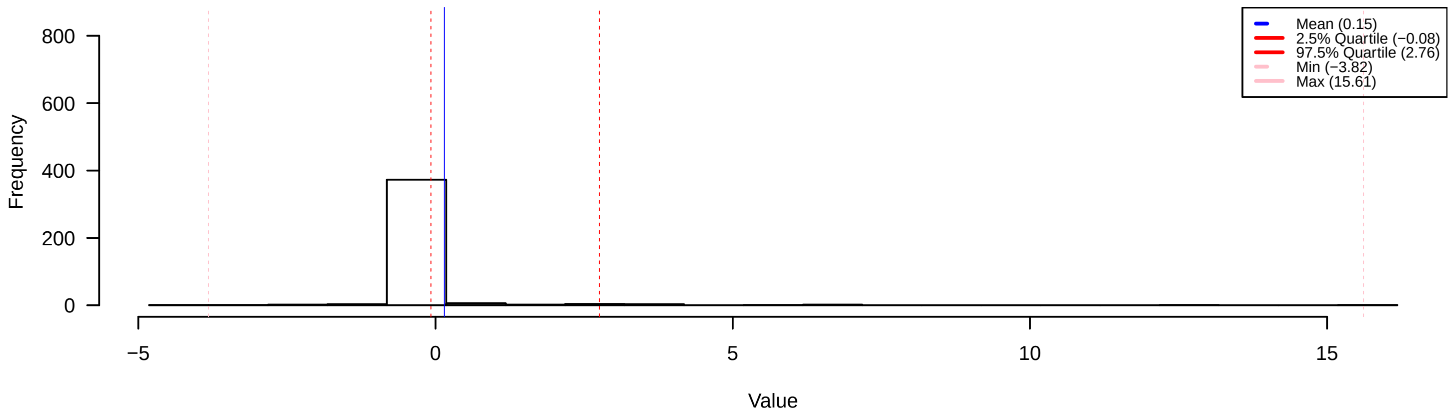

S5, Figure 75 : Bootstrap Distribution of Air Temperature lag 14

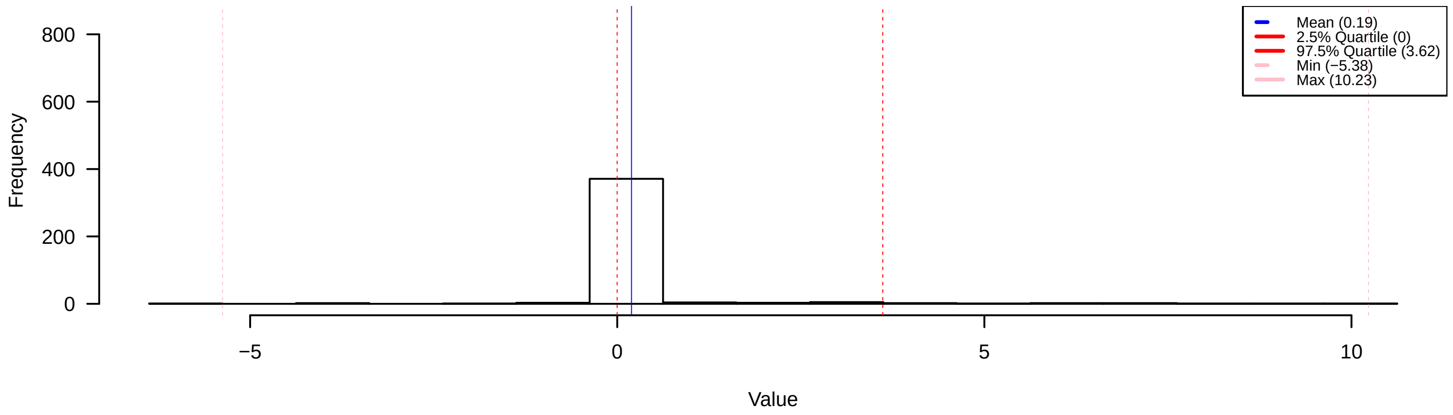

S5, Figure 76 : Bootstrap Distribution of Air Temperature lag 15

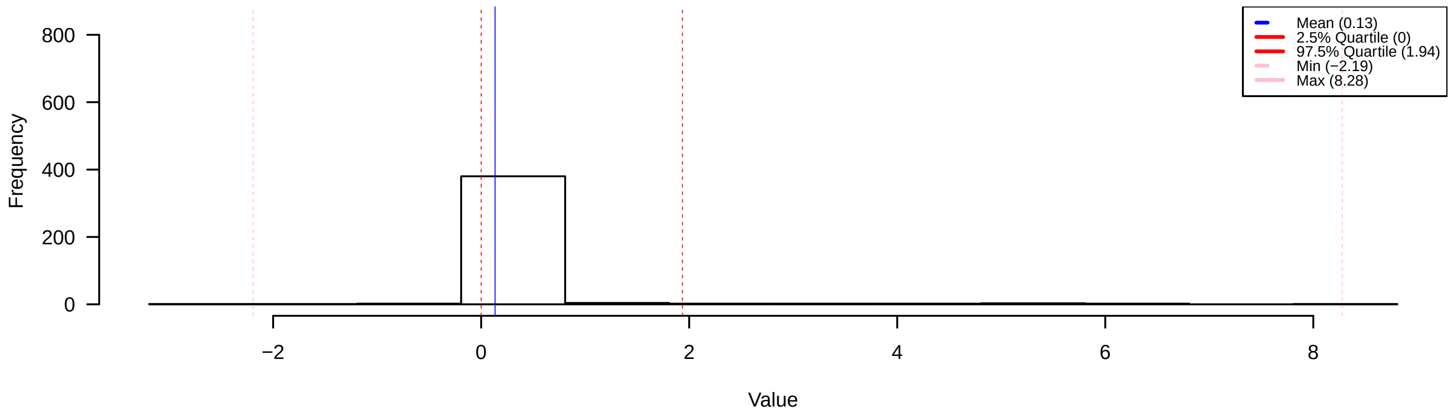

S5, Figure 77 : Bootstrap Distribution of Air Temperature lag 16

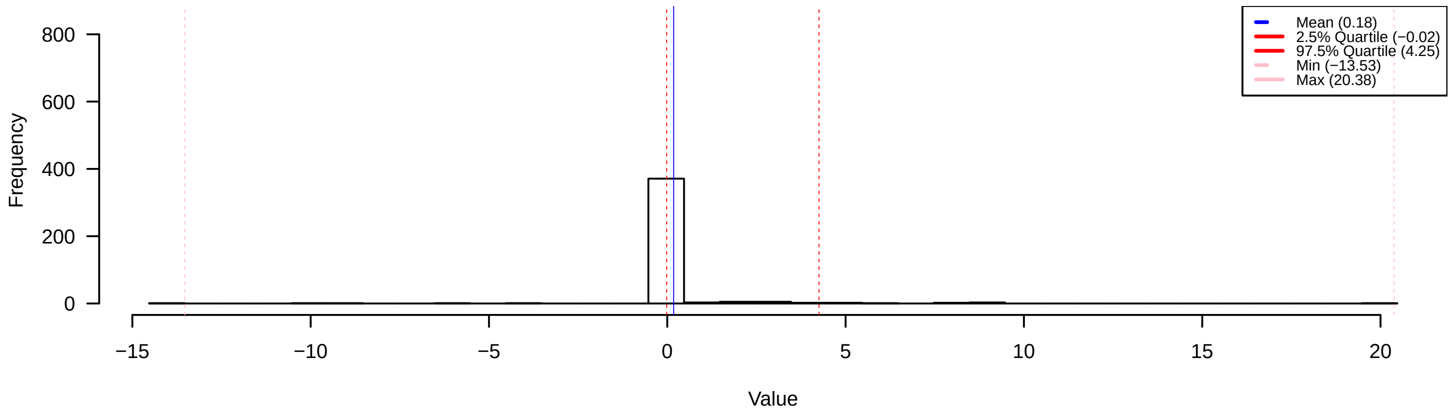

S5, Figure 78 : Bootstrap Distribution of Air Temperature lag 17

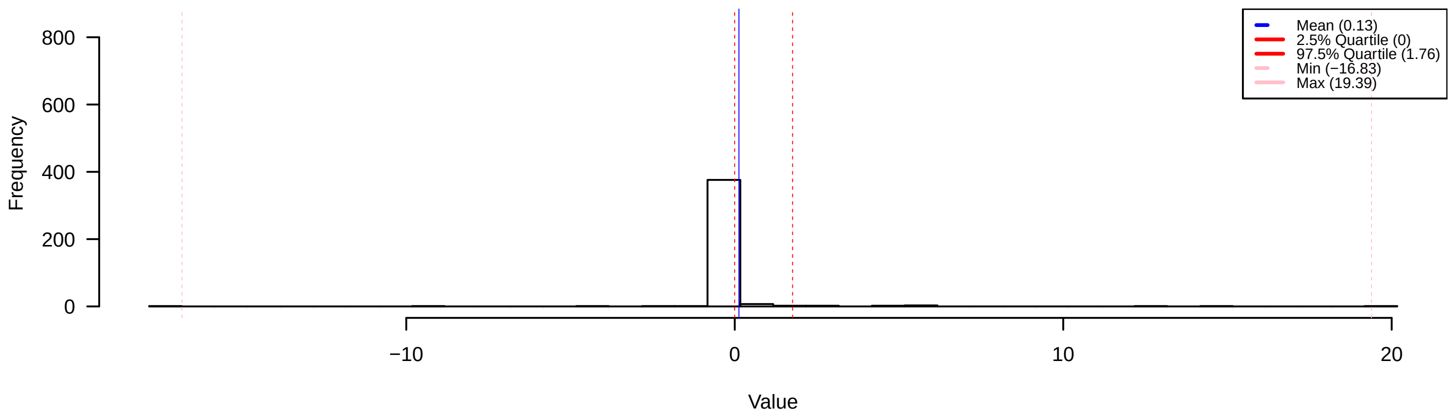

S5, Figure 79 : Bootstrap Distribution of Air Temperature lag 18

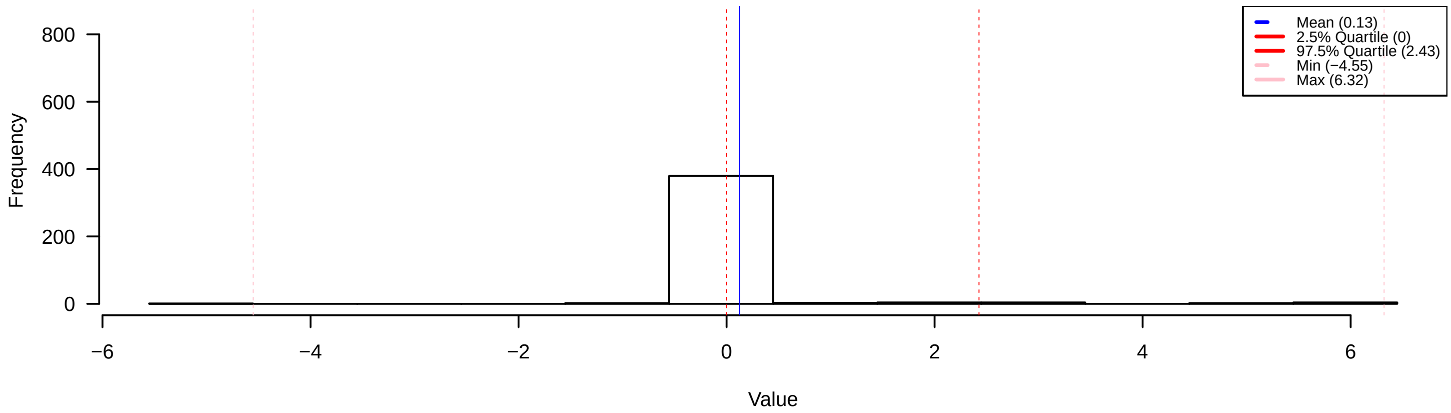

S5, Figure 80 : Bootstrap Distribution of Air Temperature lag 19

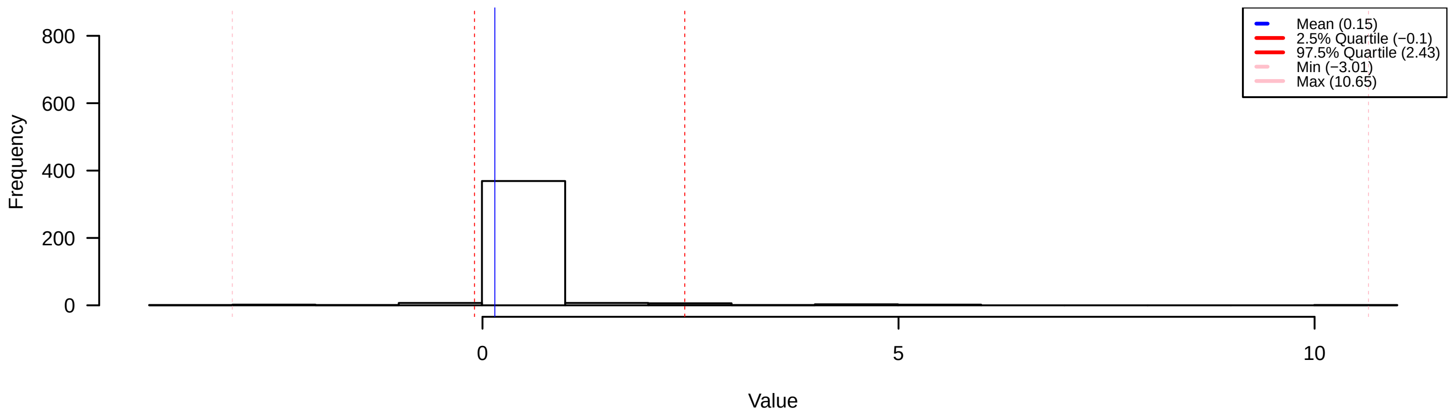

S5, Figure 81 : Bootstrap Distribution of Air Temperature lag 20

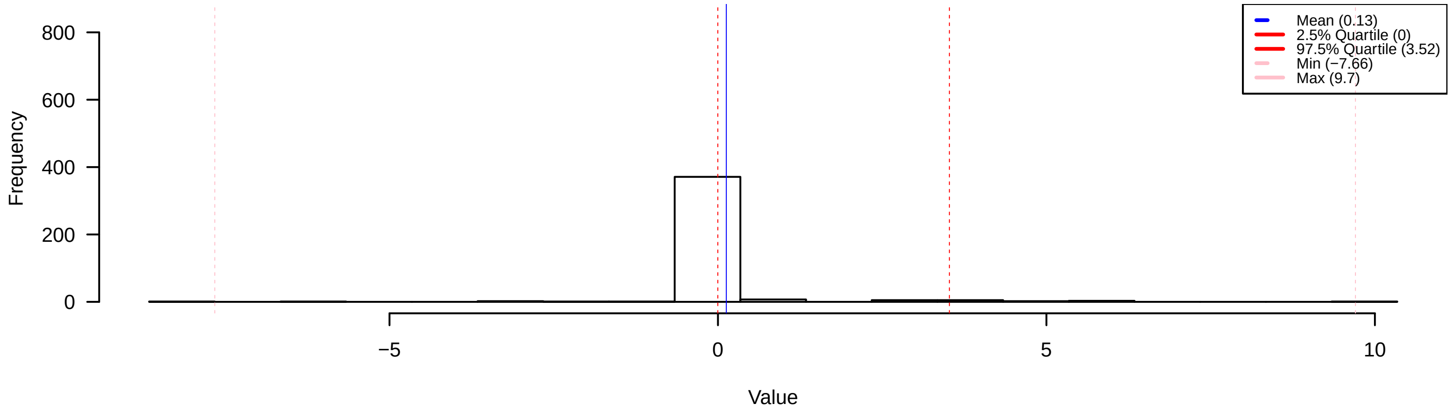

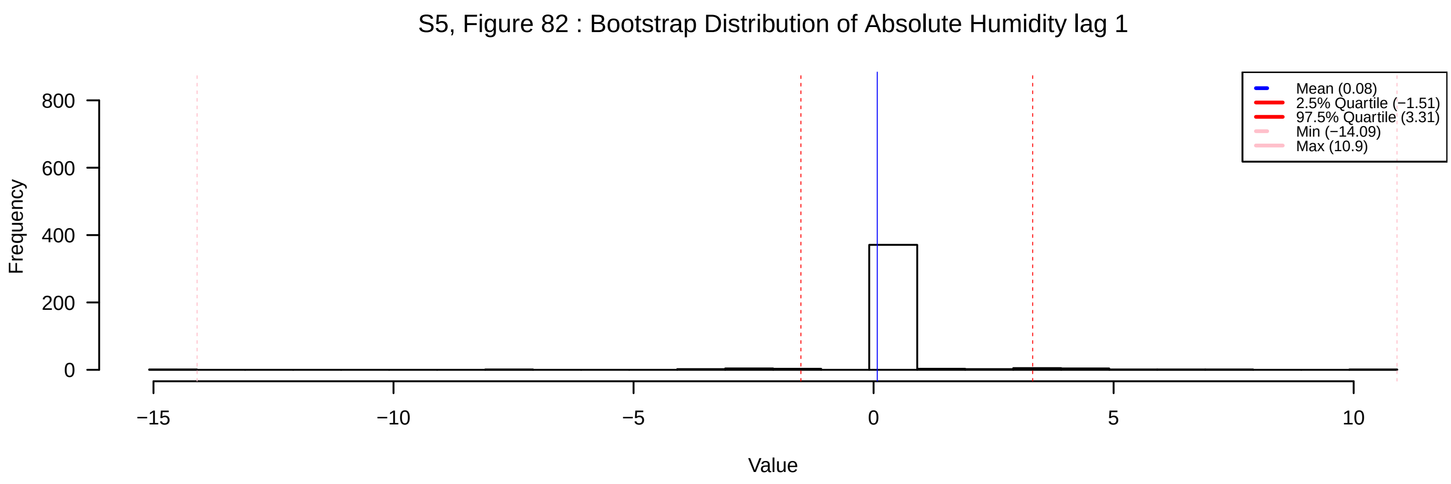
S5, Figure 82 : Bootstrap Distribution of Absolute Humidity lag 1
Mean (0.08)
2.5% Quartile (−1.51)
97.5% Quartile (3.31)
Min (−14.09)
Max (10.9)
Frequency
Value

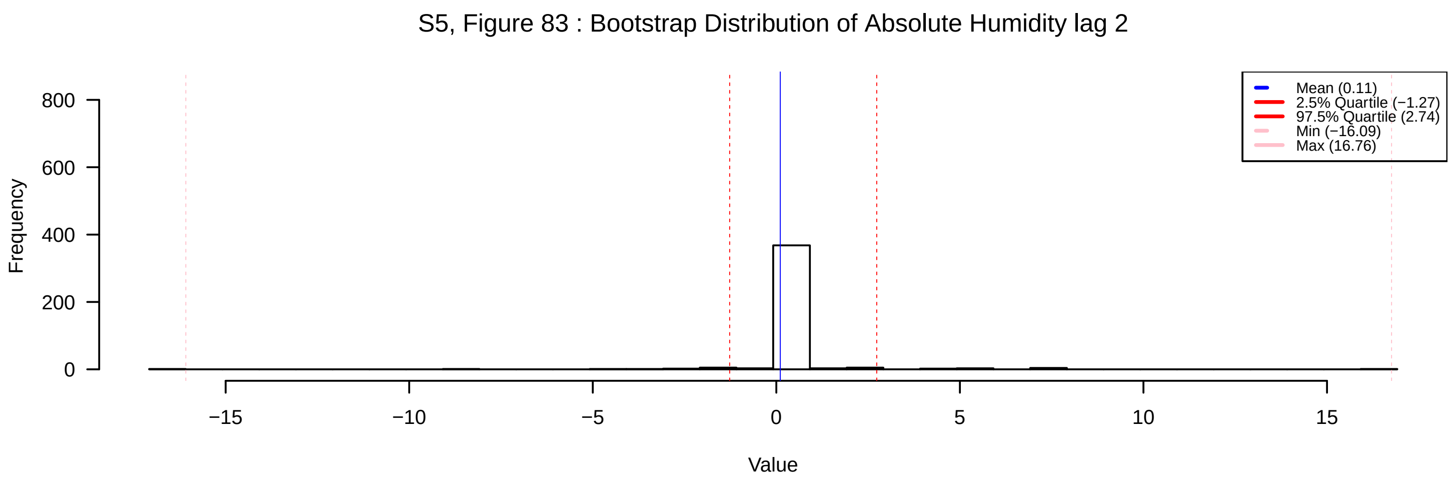
S5, Figure 83 : Bootstrap Distribution of Absolute Humidity lag 2
Mean (0.11)
2.5% Quartile (−1.27)
97.5% Quartile (2.74)
Min (−16.09)
Max (16.76)
Frequency
Value

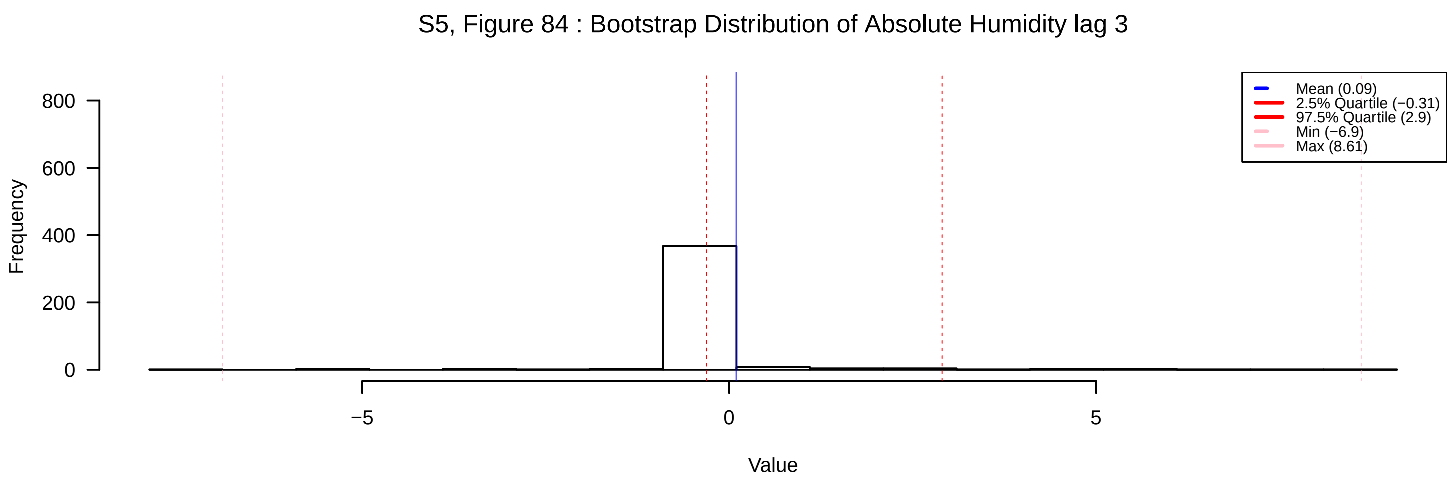
S5, Figure 84 : Bootstrap Distribution of Absolute Humidity lag 3
Mean (0.09)
2.5% Quartile (−0.31)
97.5% Quartile (2.9)
Min (−6.9)
Max (8.61)
Frequency
Value

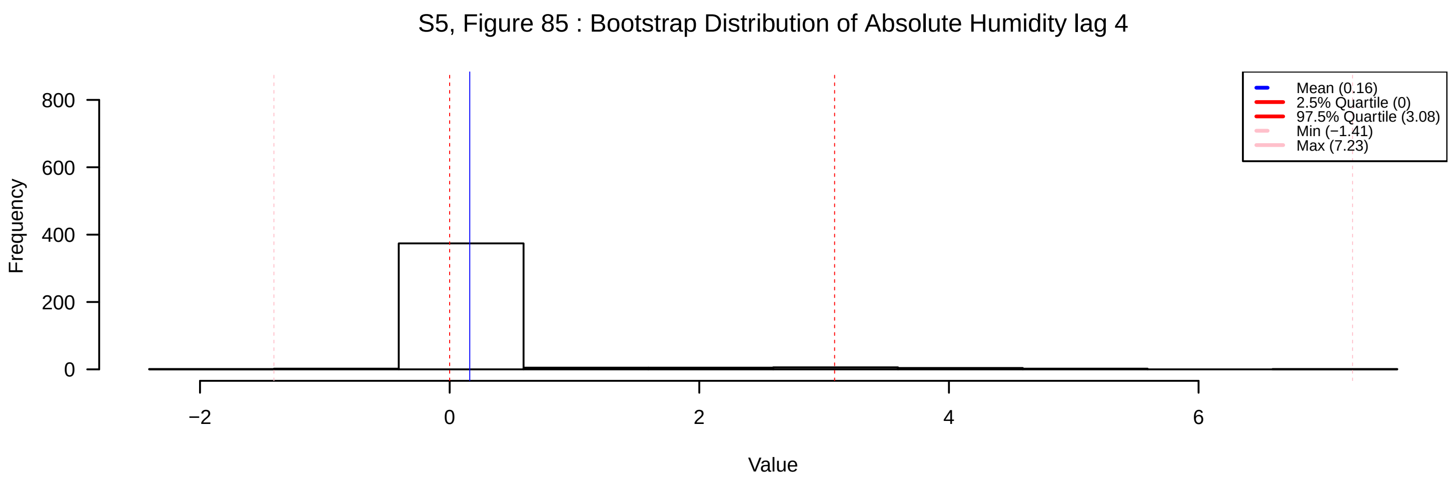
S5, Figure 85 : Bootstrap Distribution of Absolute Humidity lag 4
Mean (0.16)
2.5% Quartile (0)
97.5% Quartile (3.08)
Min (−1.41)
Max (7.23)
Frequency
Value

S5, Figure 86 : Bootstrap Distribution of Absolute Humidity lag 5

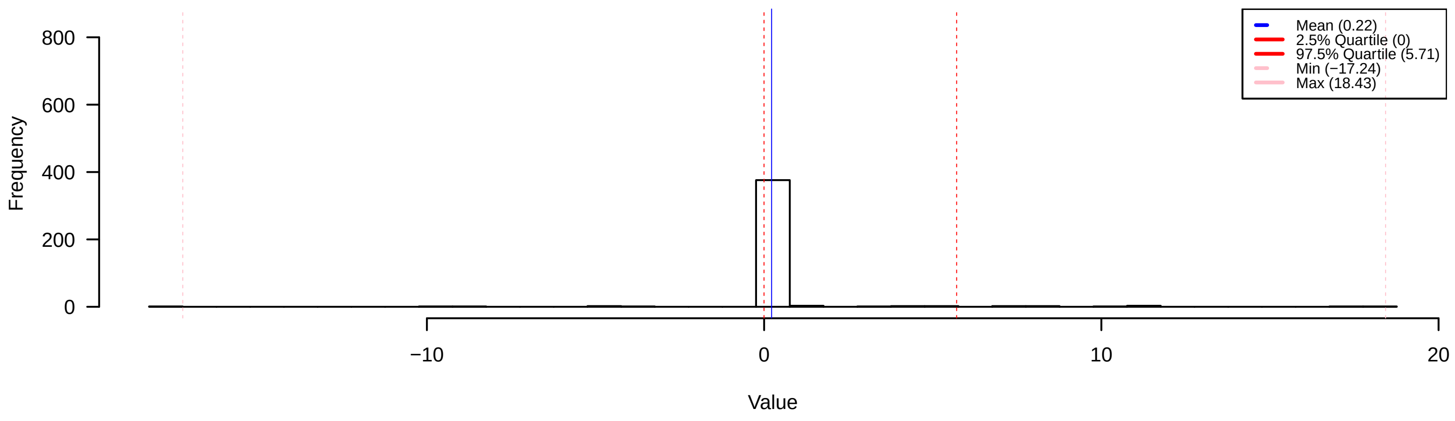

S5, Figure 87 : Bootstrap Distribution of Absolute Humidity lag 6

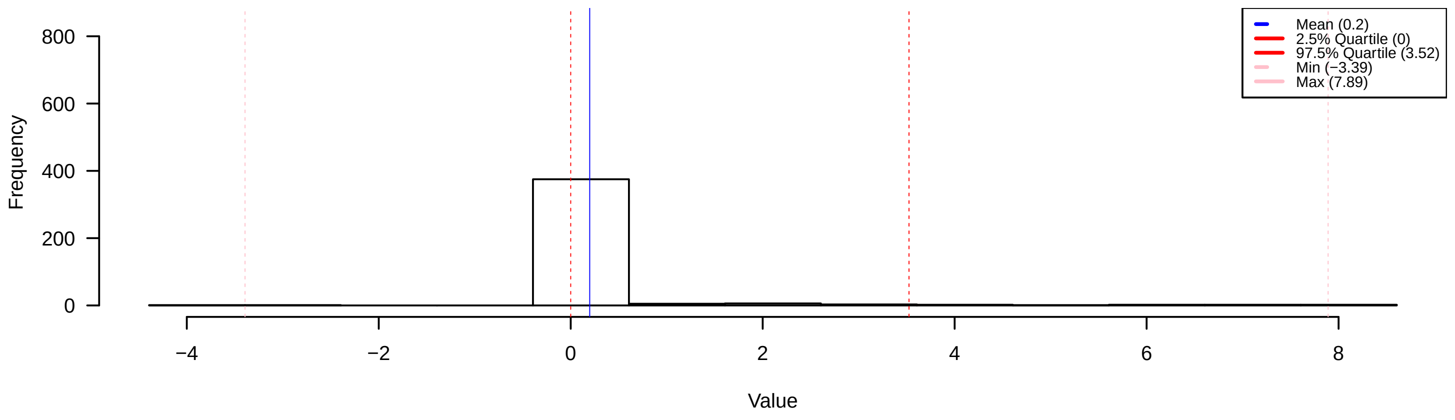

S5, Figure 88 : Bootstrap Distribution of Absolute Humidity lag 7

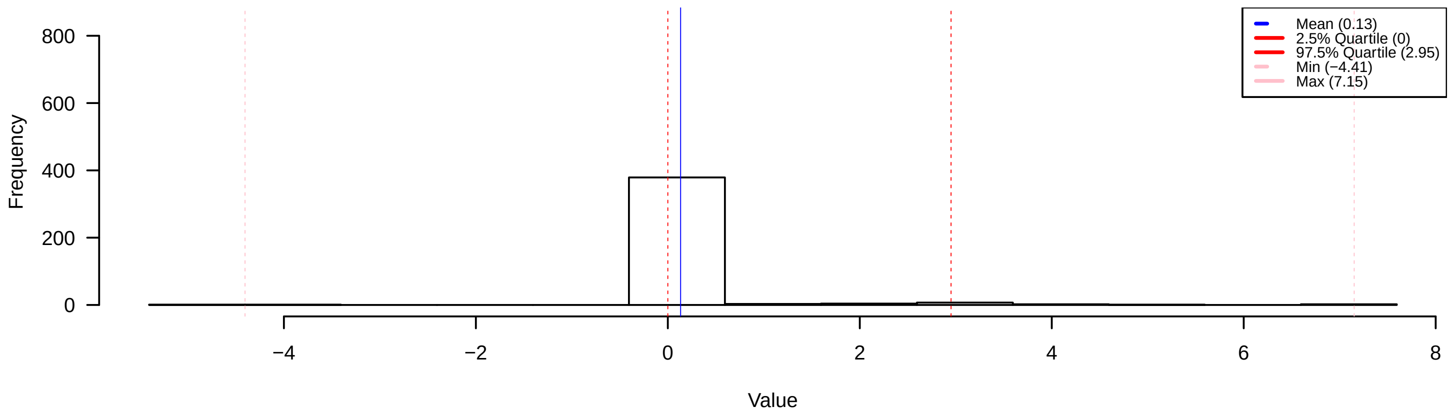

S5, Figure 89 : Bootstrap Distribution of Absolute Humidity lag 8

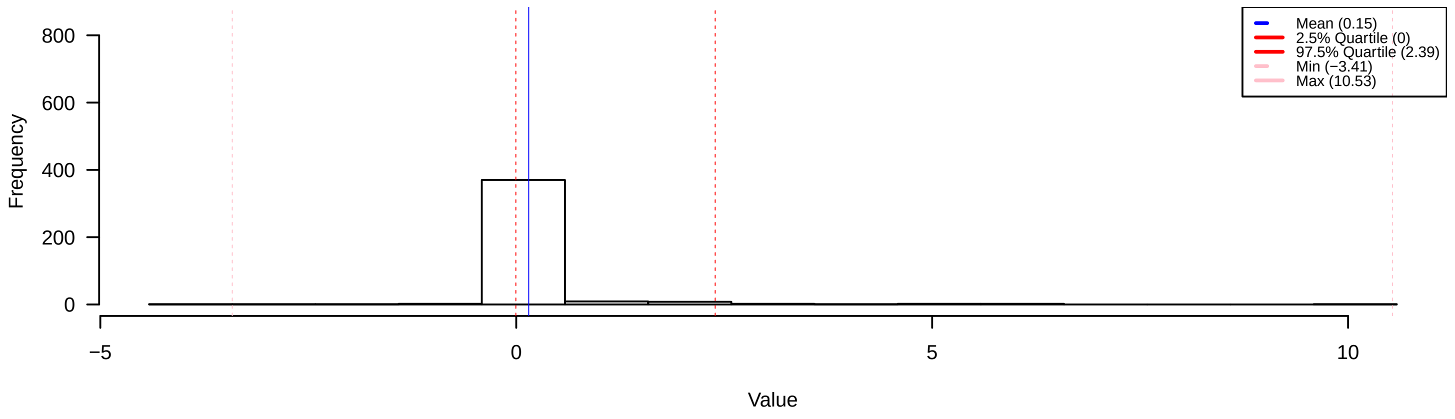

S5, Figure 90 : Bootstrap Distribution of Absolute Humidity lag 9

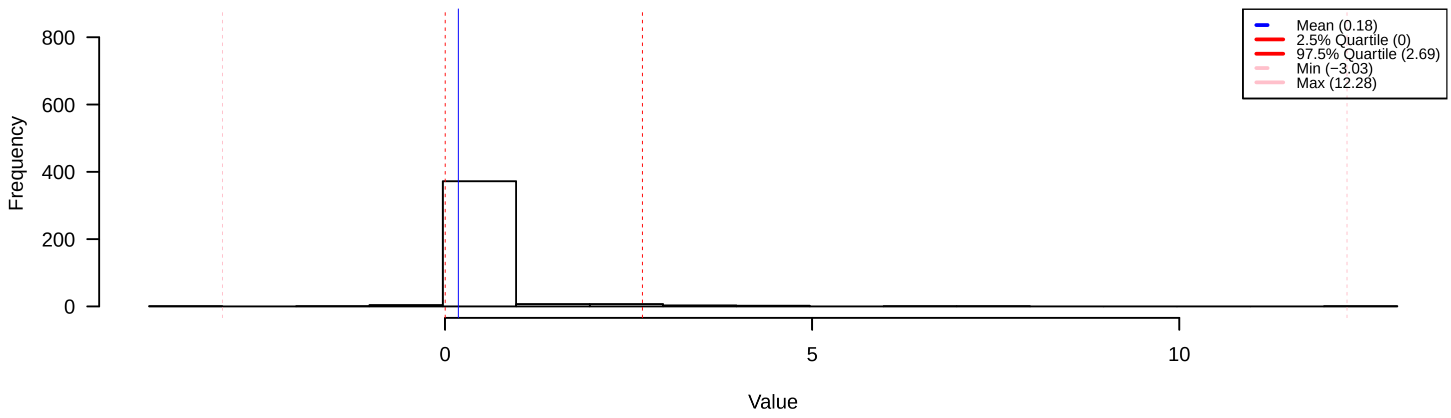

S5, Figure 91 : Bootstrap Distribution of Absolute Humidity lag 10

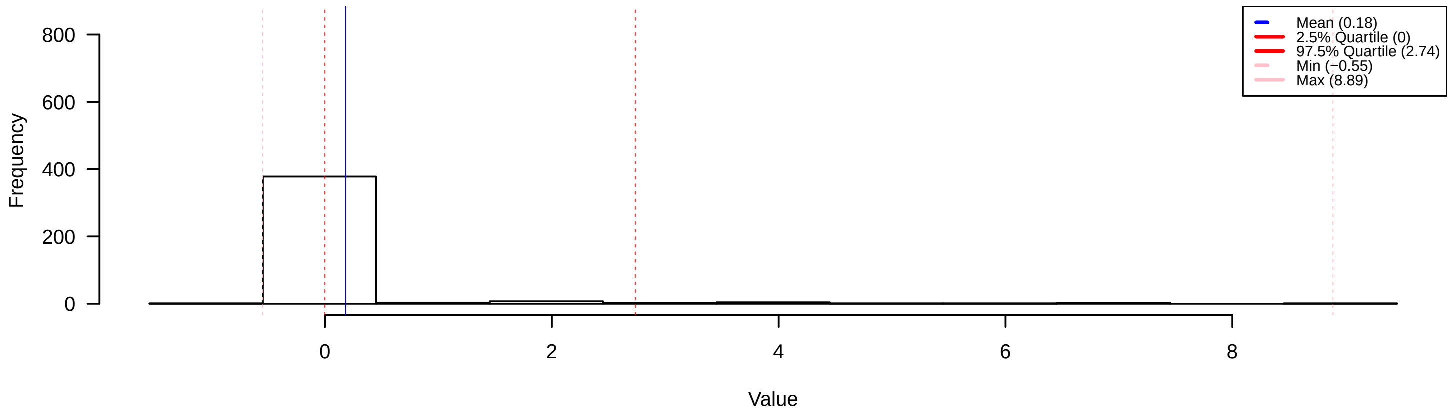

S5, Figure 92 : Bootstrap Distribution of Absolute Humidity lag 11

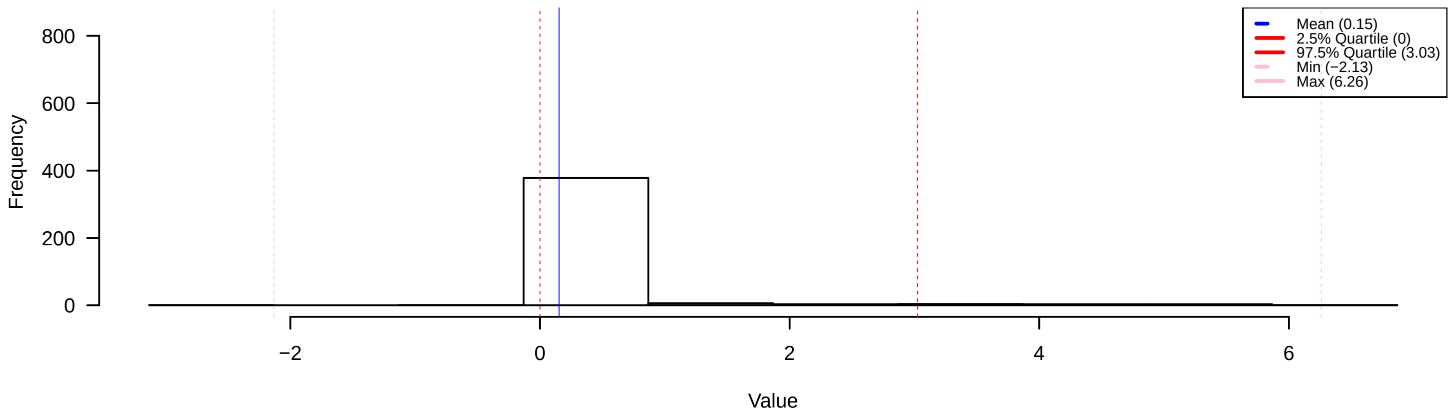

S5, Figure 93 : Bootstrap Distribution of Absolute Humidity lag 12

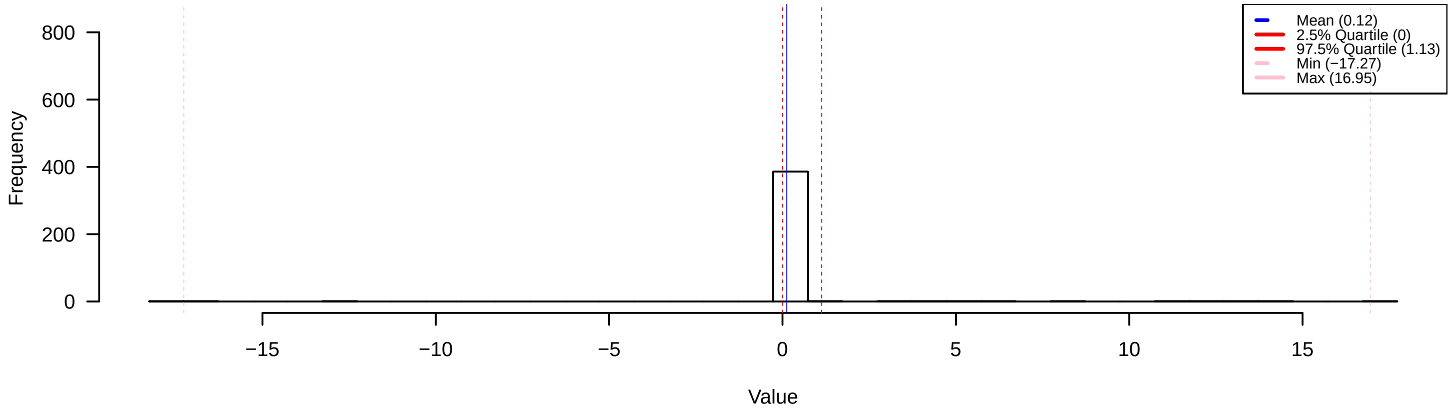

S5, Figure 94 : Bootstrap Distribution of Absolute Humidity lag 13

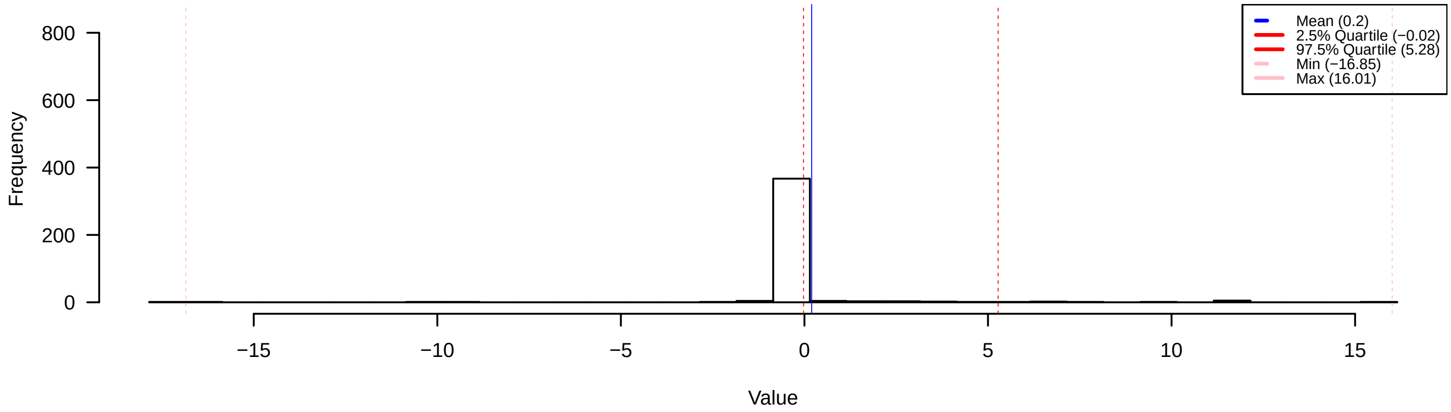

S5, Figure 95 : Bootstrap Distribution of Absolute Humidity lag 14

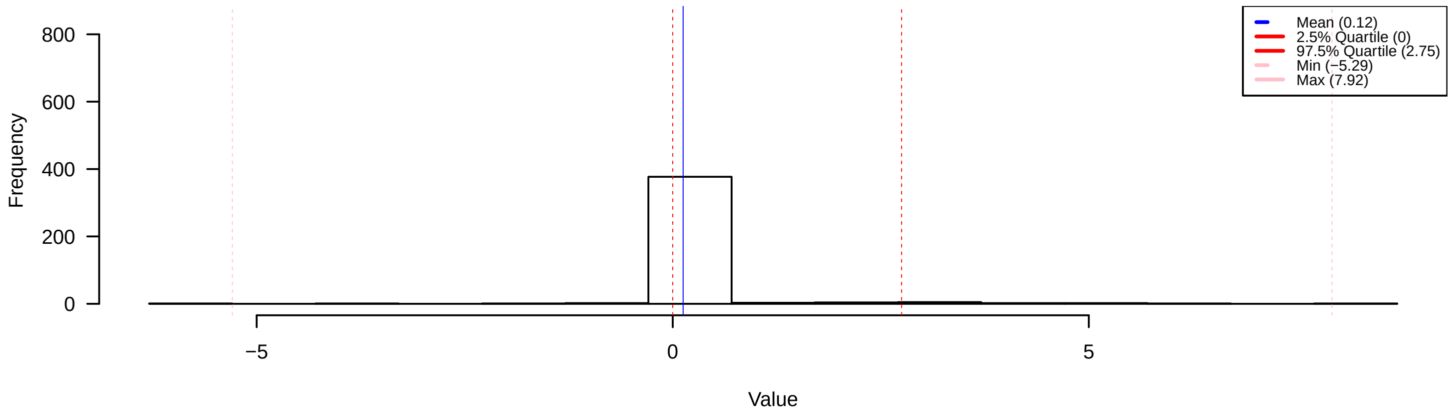

S5, Figure 96 : Bootstrap Distribution of Absolute Humidity lag 15

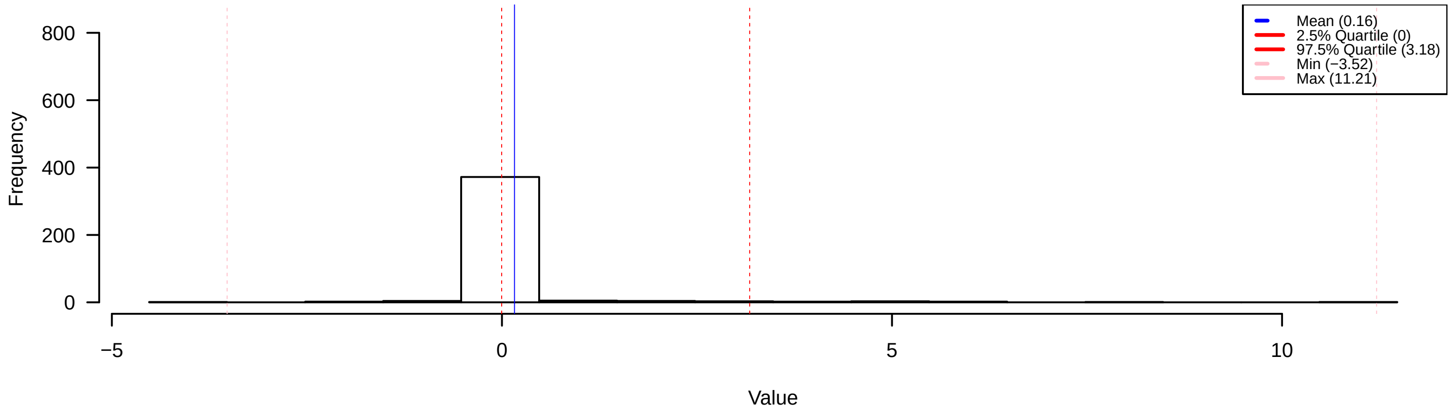

S5, Figure 97 : Bootstrap Distribution of Absolute Humidity lag 16

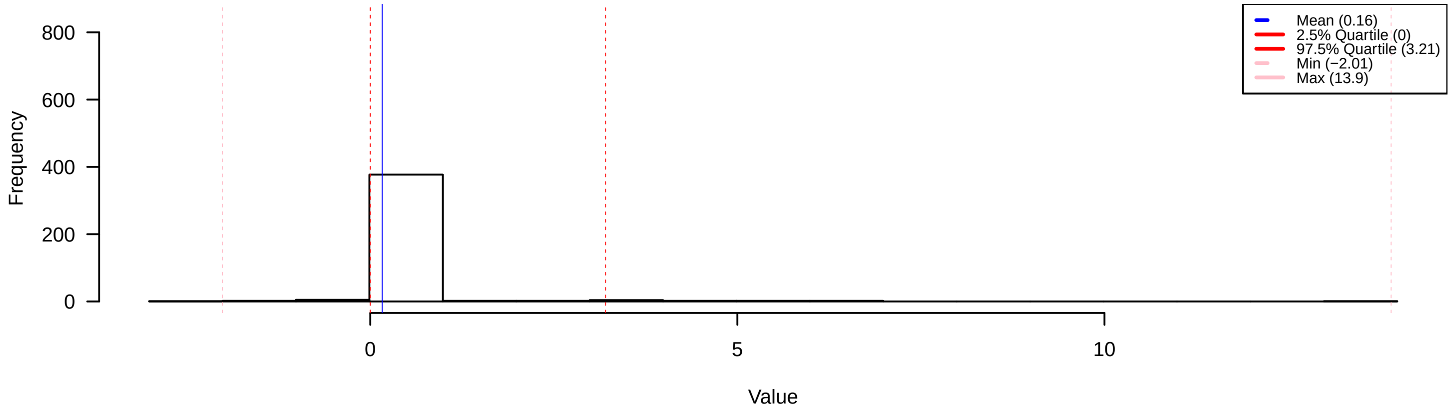

S5, Figure 98 : Bootstrap Distribution of Absolute Humidity lag 17

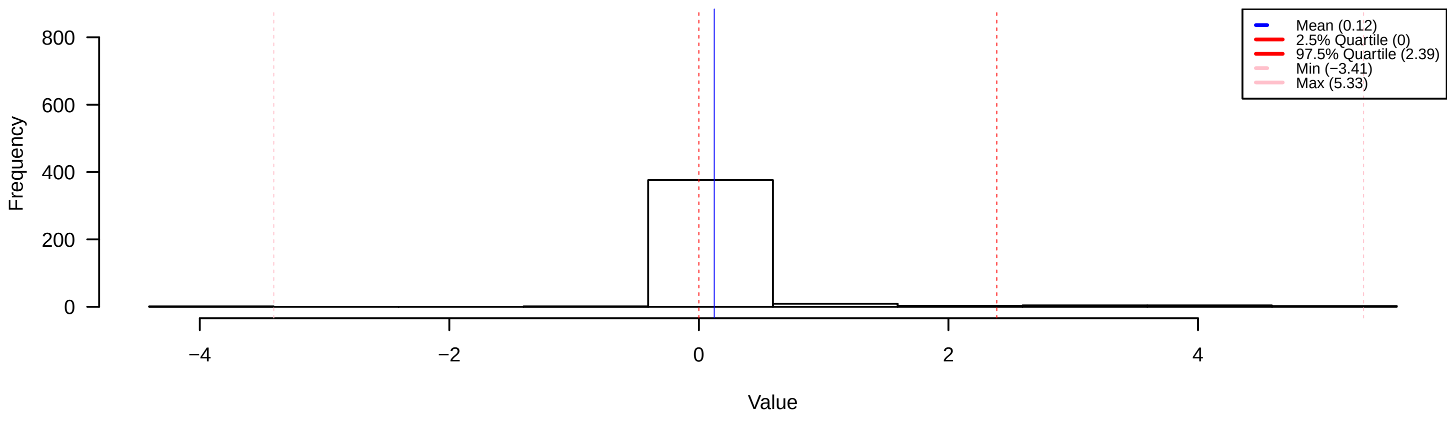

S5, Figure 99 : Bootstrap Distribution of Absolute Humidity lag 18

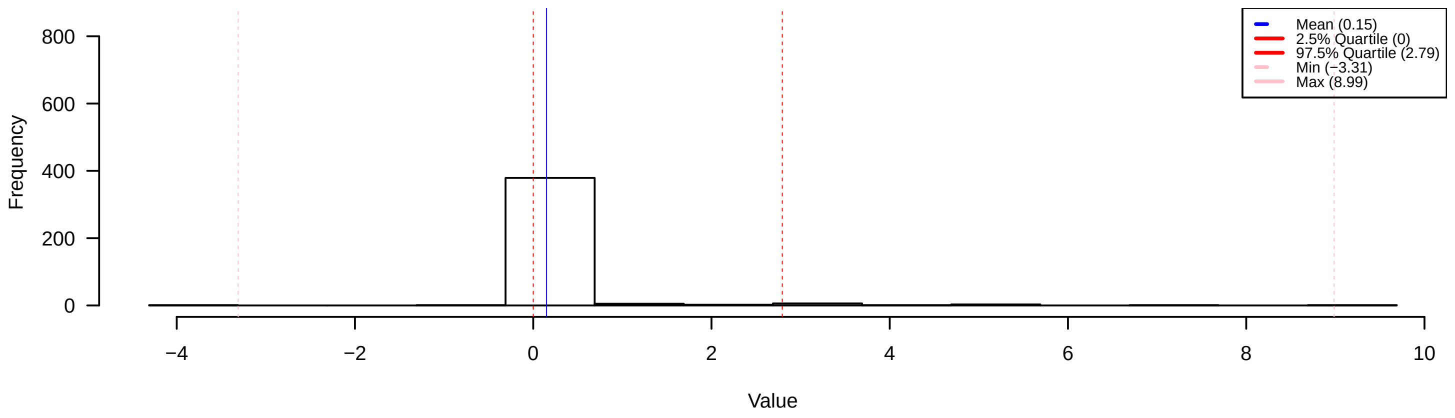

S5, Figure 100 : Bootstrap Distribution of Absolute Humidity lag 19

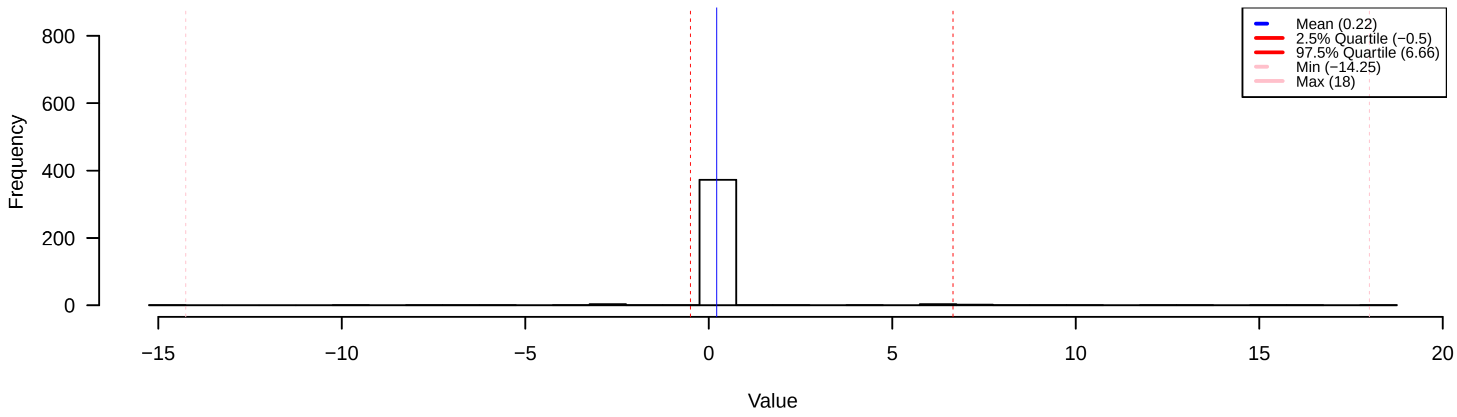

S5, Figure 101 : Bootstrap Distribution of Absolute Humidity lag 20

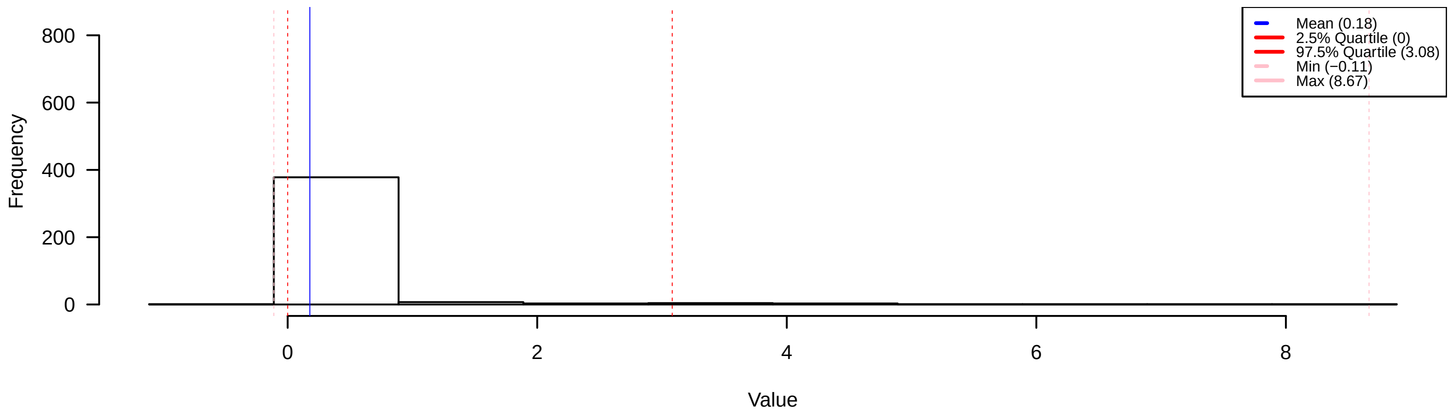

S5, Figure 102 : Bootstrap Distribution of Relative Humidity lag 1

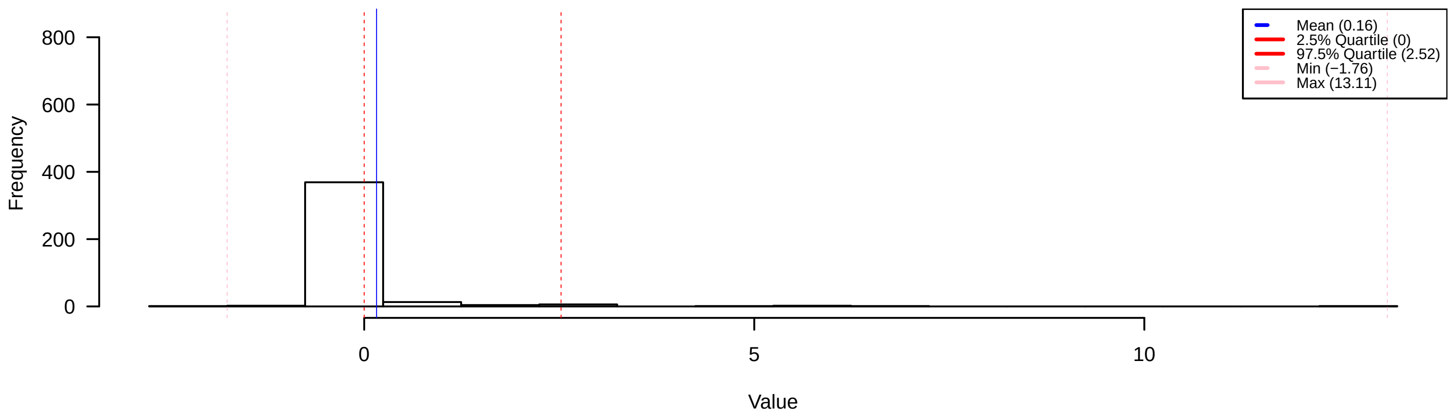

S5, Figure 103 : Bootstrap Distribution of Relative Humidity lag 2

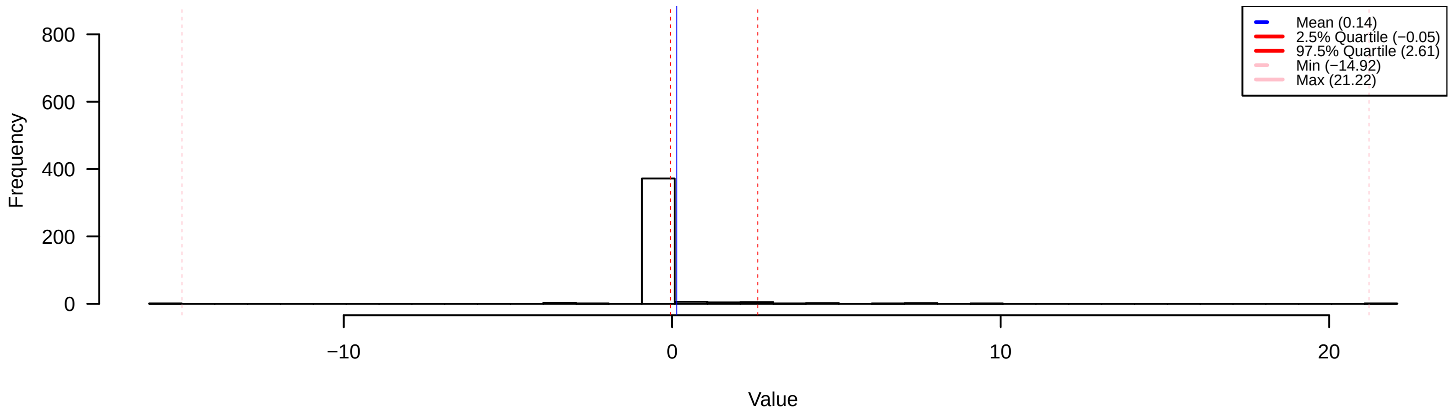

S5, Figure 104 : Bootstrap Distribution of Relative Humidity lag 3

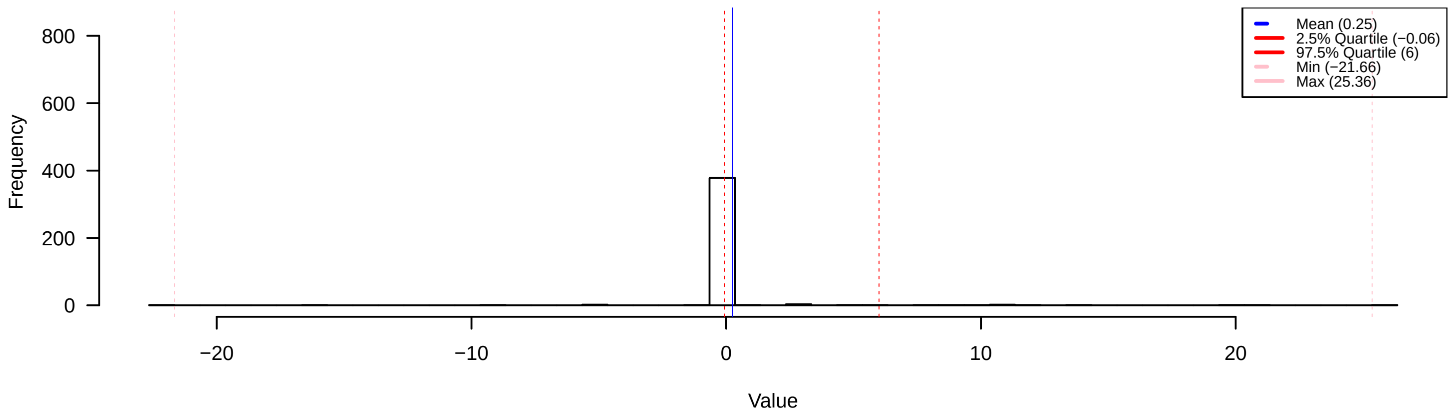

S5, Figure 105 : Bootstrap Distribution of Relative Humidity lag 4

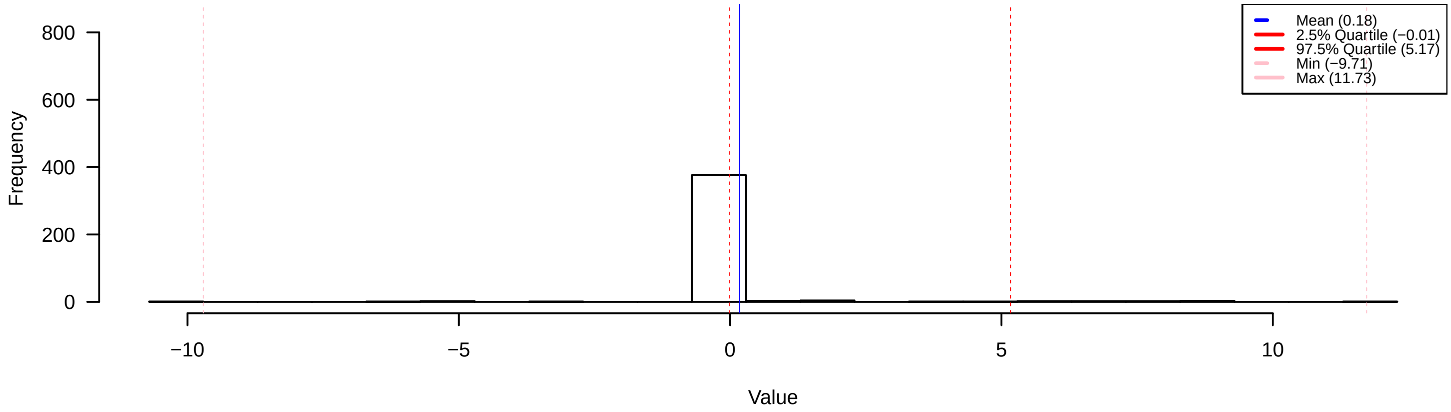

S5, Figure 106 : Bootstrap Distribution of Relative Humidity lag 5

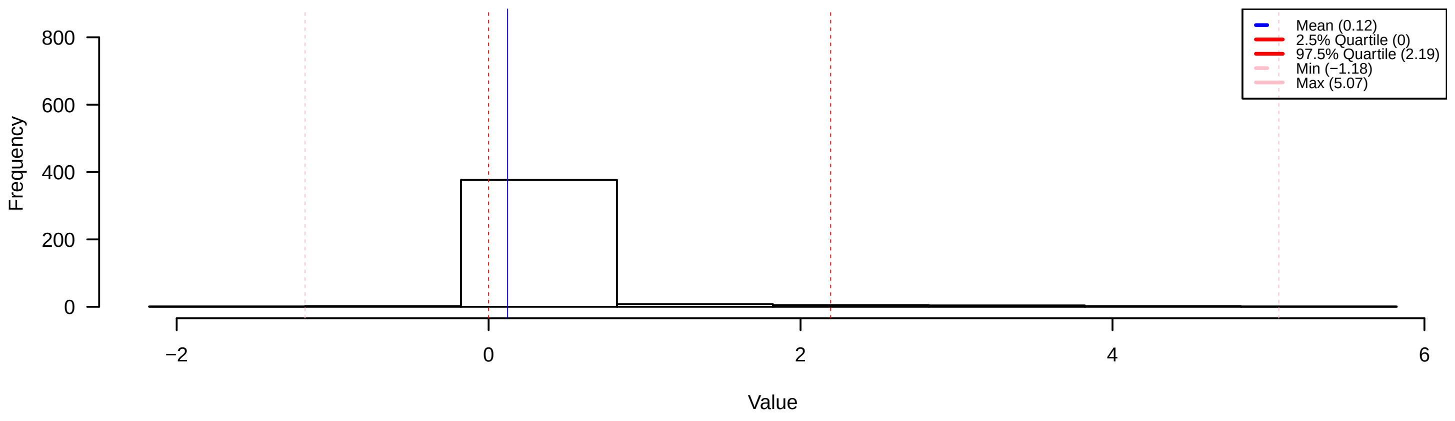

S5, Figure 107 : Bootstrap Distribution of Relative Humidity lag 6

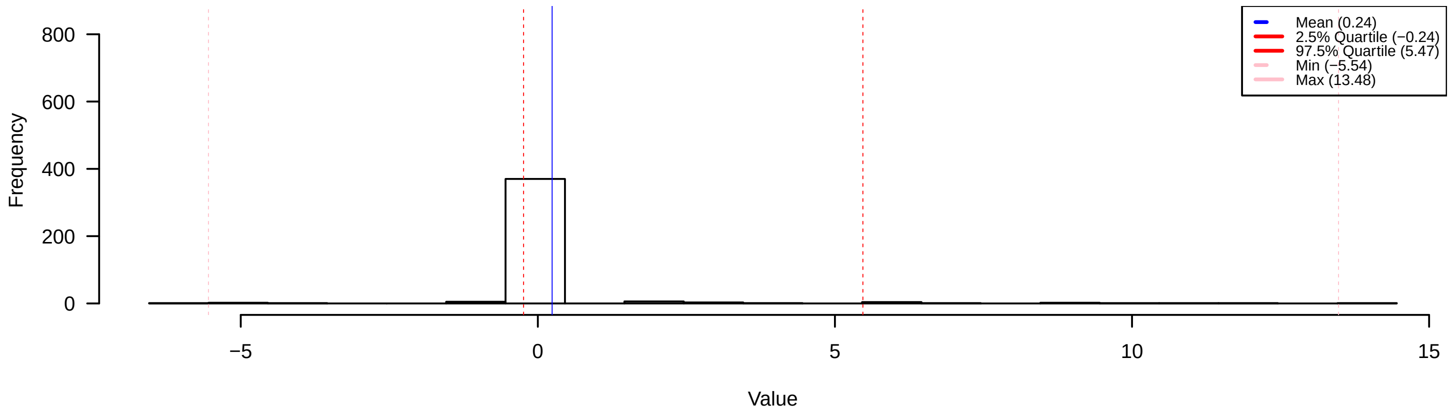

S5, Figure 108 : Bootstrap Distribution of Relative Humidity lag 7

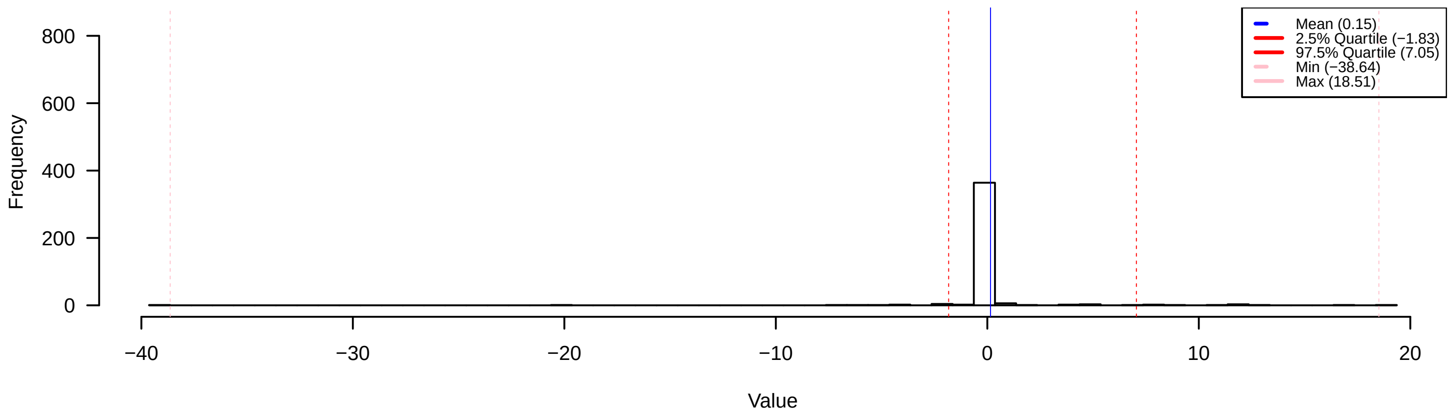

S5, Figure 109 : Bootstrap Distribution of Relative Humidity lag 8

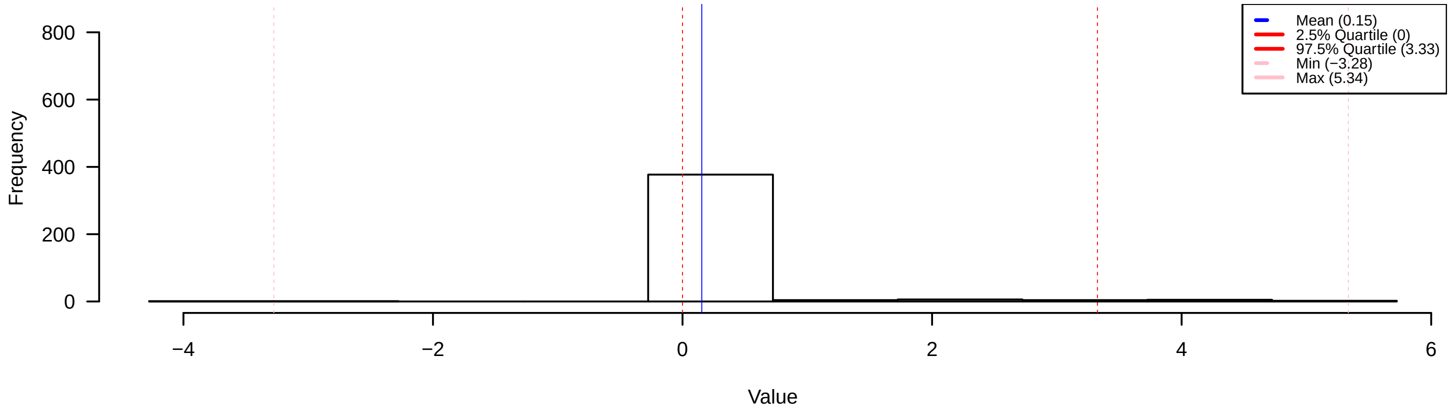

S5, Figure 110 : Bootstrap Distribution of Relative Humidity lag 9

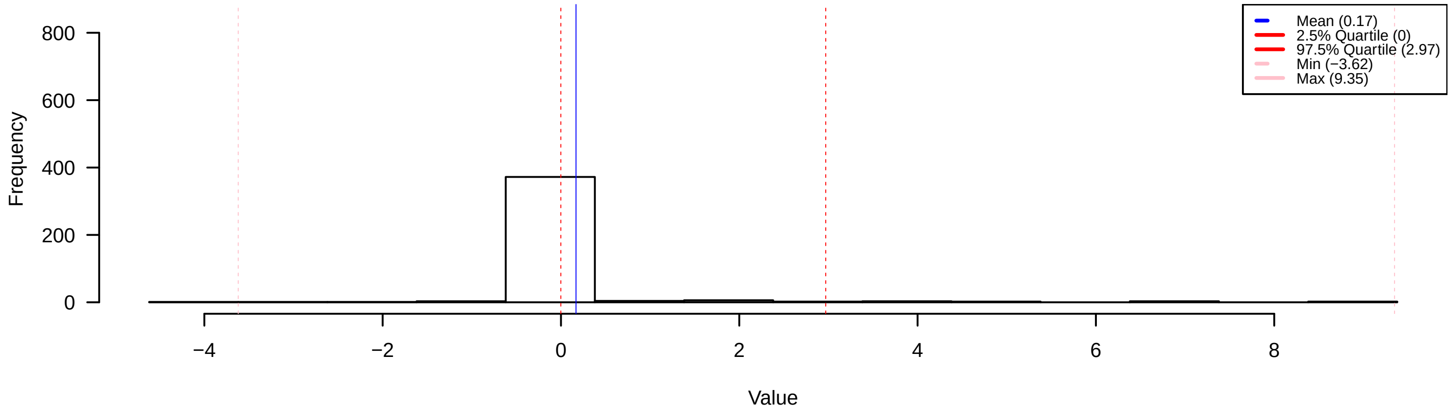

S5, Figure 111 : Bootstrap Distribution of Relative Humidity lag 10

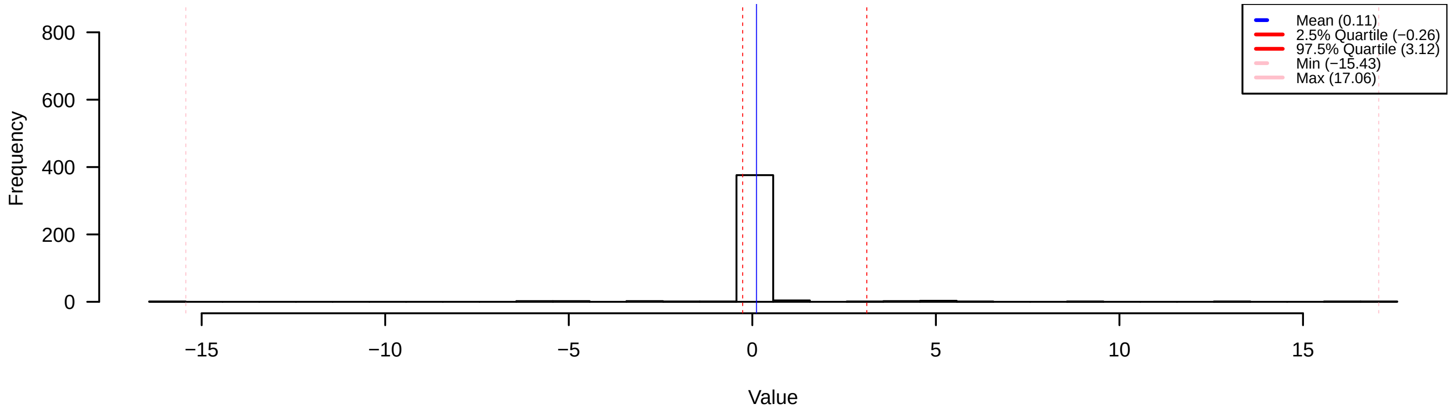

S5, Figure 112 : Bootstrap Distribution of Relative Humidity lag 11

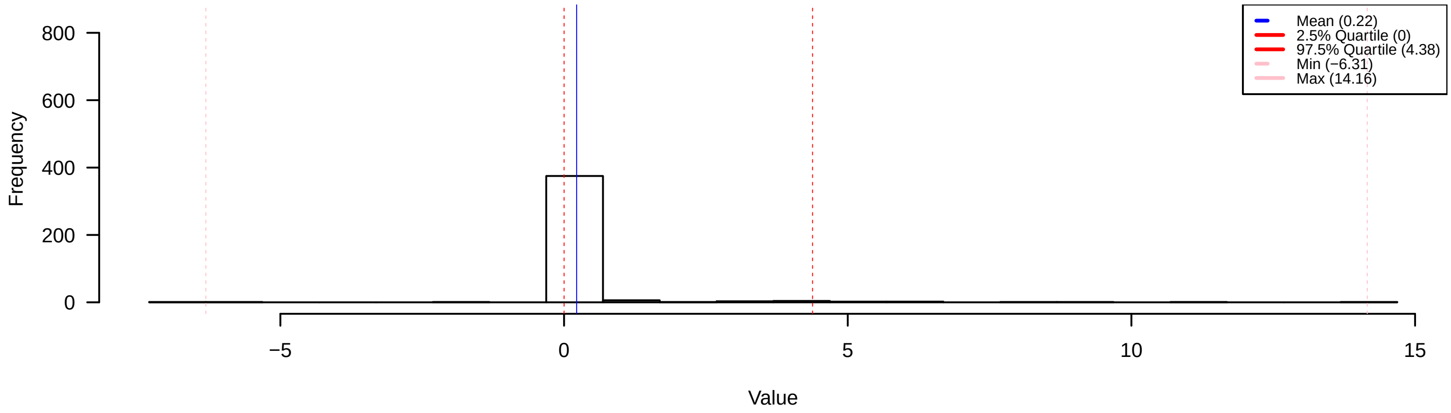

S5, Figure 113 : Bootstrap Distribution of Relative Humidity lag 12

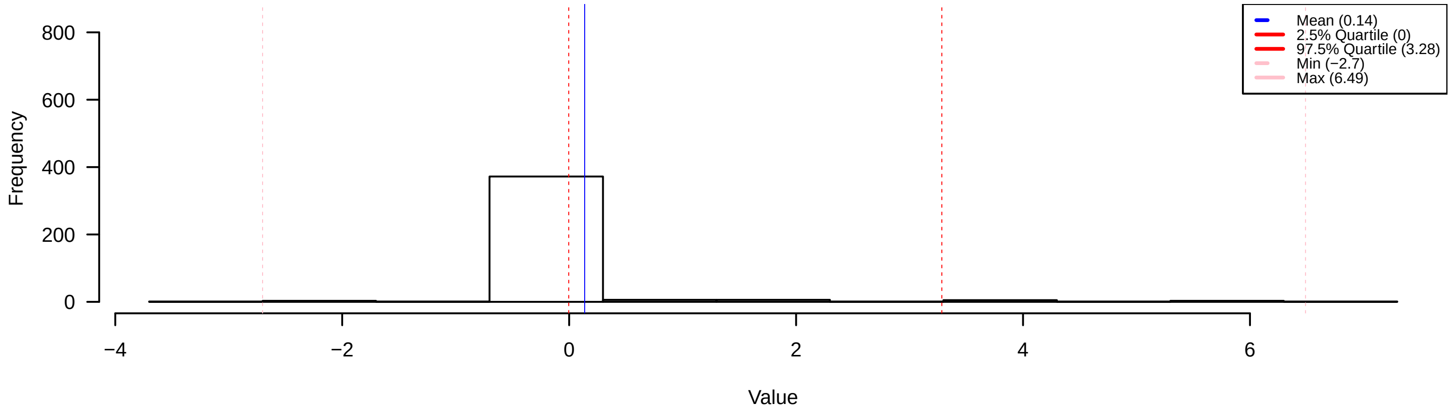

S5, Figure 114 : Bootstrap Distribution of Relative Humidity lag 13

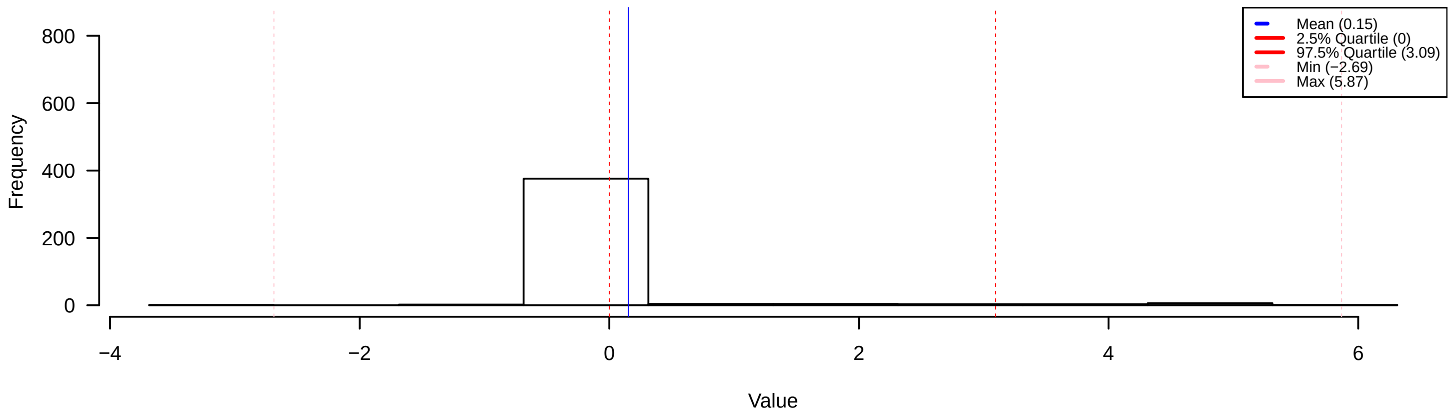

S5, Figure 115 : Bootstrap Distribution of Relative Humidity lag 14

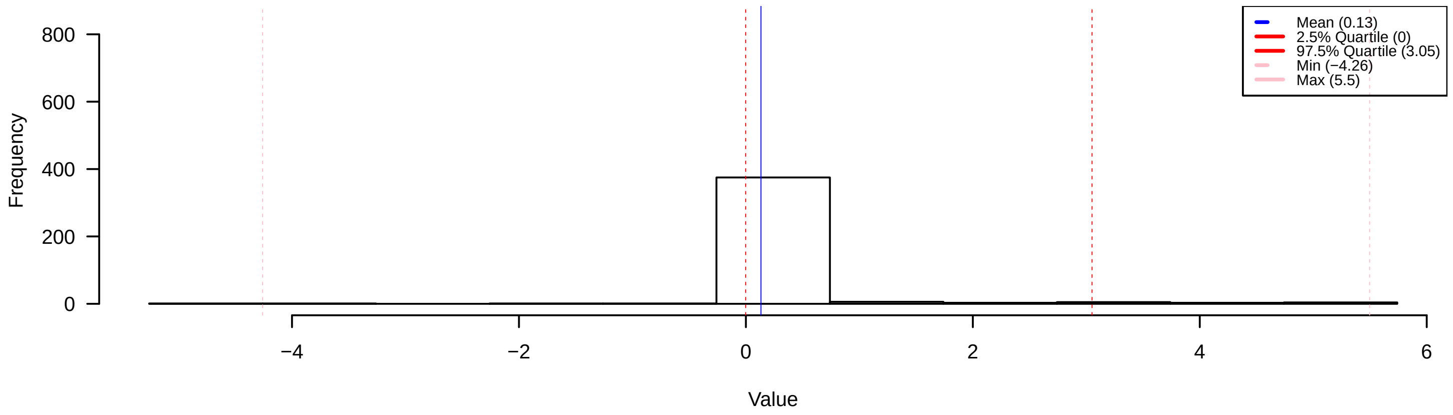

S5, Figure 116 : Bootstrap Distribution of Relative Humidity lag 15

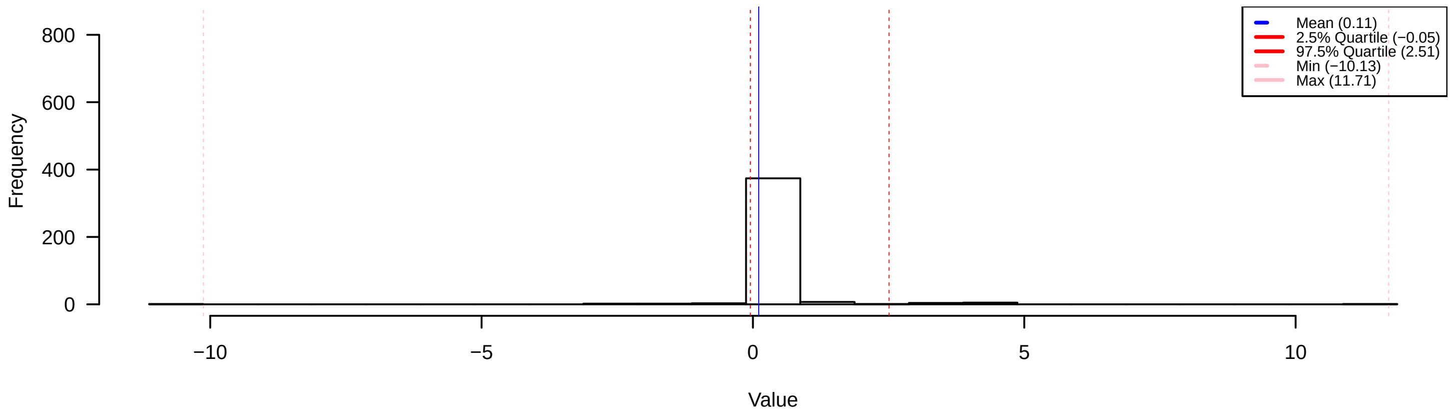

S5, Figure 117 : Bootstrap Distribution of Relative Humidity lag 16

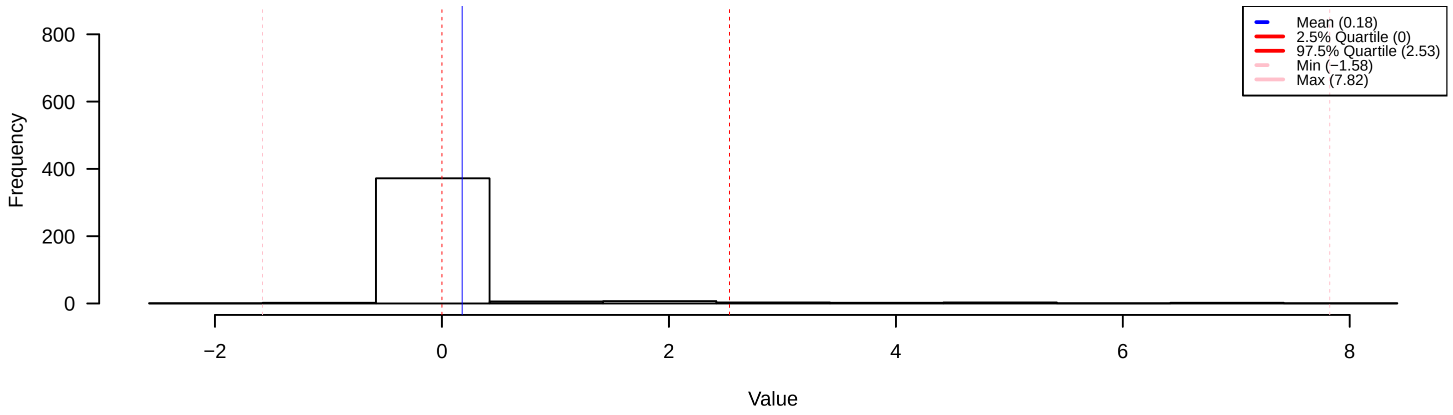

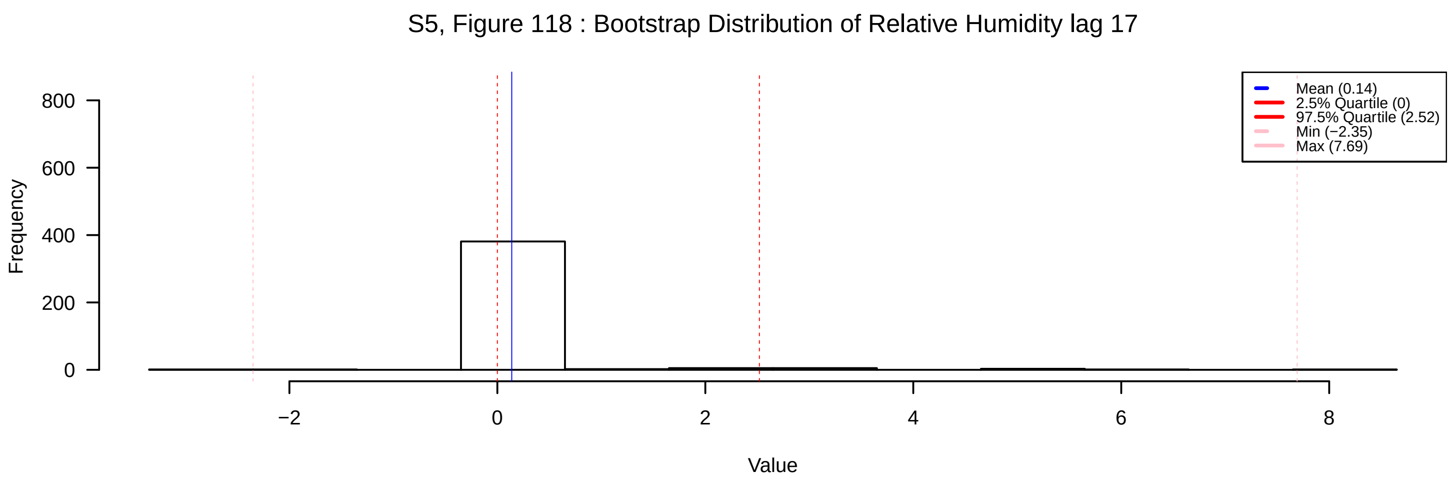
S5, Figure 118 : Bootstrap Distribution of Relative Humidity lag 17
Mean (0.14)
2.5% Quartile (0)
97.5% Quartile (2.52)
Min (−2.35)
Max (7.69)
Frequency
Value

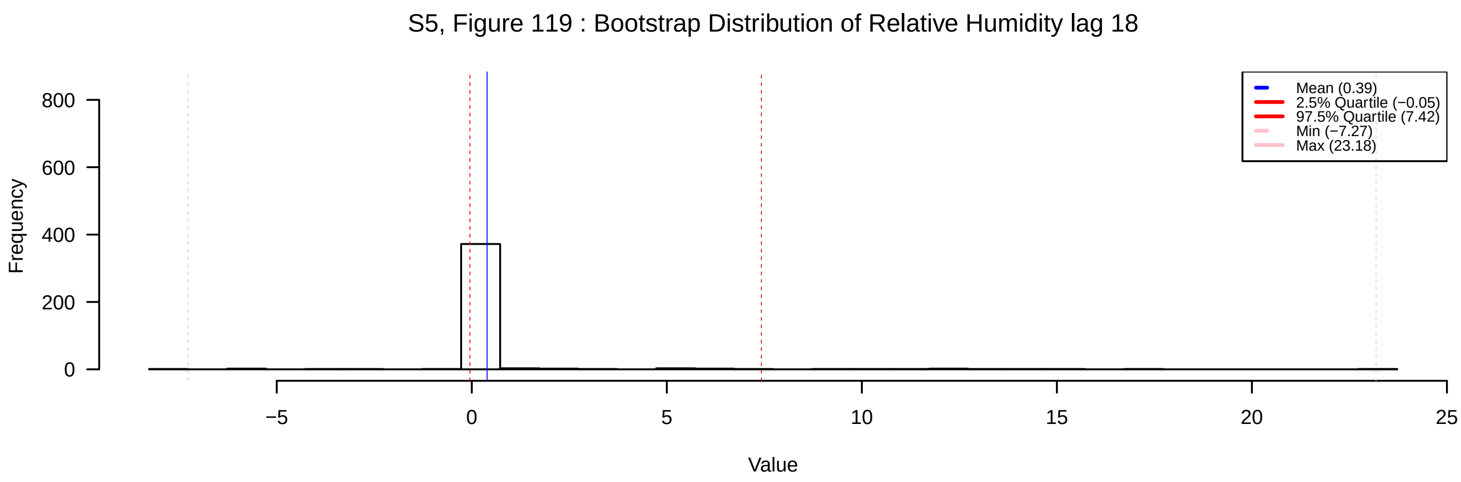
S5, Figure 119 : Bootstrap Distribution of Relative Humidity lag 18
Mean (0.39)
2.5% Quartile (−0.05)
97.5% Quartile (7.42)
Min (−7.27)
Max (23.18)
Frequency
Value

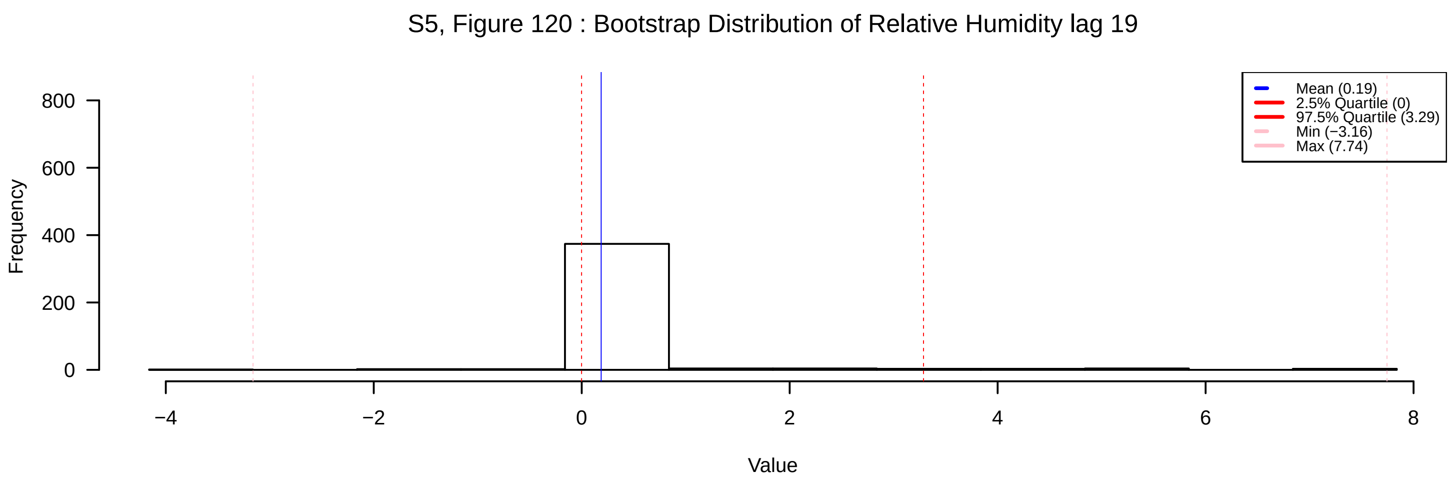
S5, Figure 120 : Bootstrap Distribution of Relative Humidity lag 19
Mean (0.19)
2.5% Quartile (0)
97.5% Quartile (3.29)
Min (−3.16)
Max (7.74)
Frequency
Value

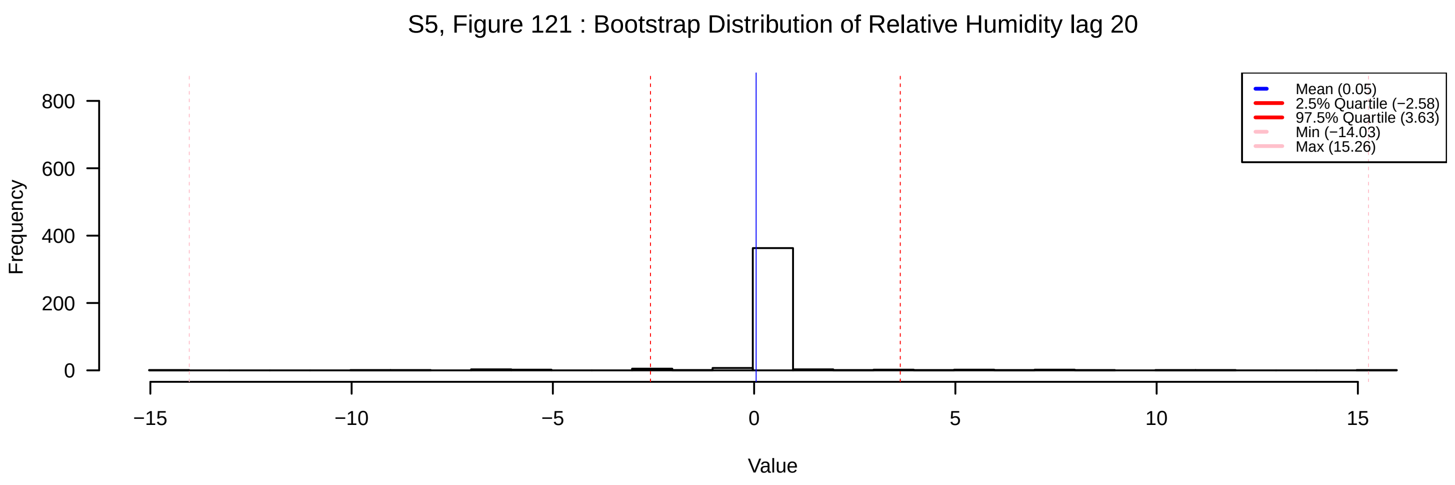
S5, Figure 121 : Bootstrap Distribution of Relative Humidity lag 20
Mean (0.05)
2.5% Quartile (−2.58)
97.5% Quartile (3.63)
Min (−14.03)
Max (15.26)
Frequency
Value

S5, Figure 122 : Bootstrap Distribution of Dewpoint Temperature:Air Temperature lag 1

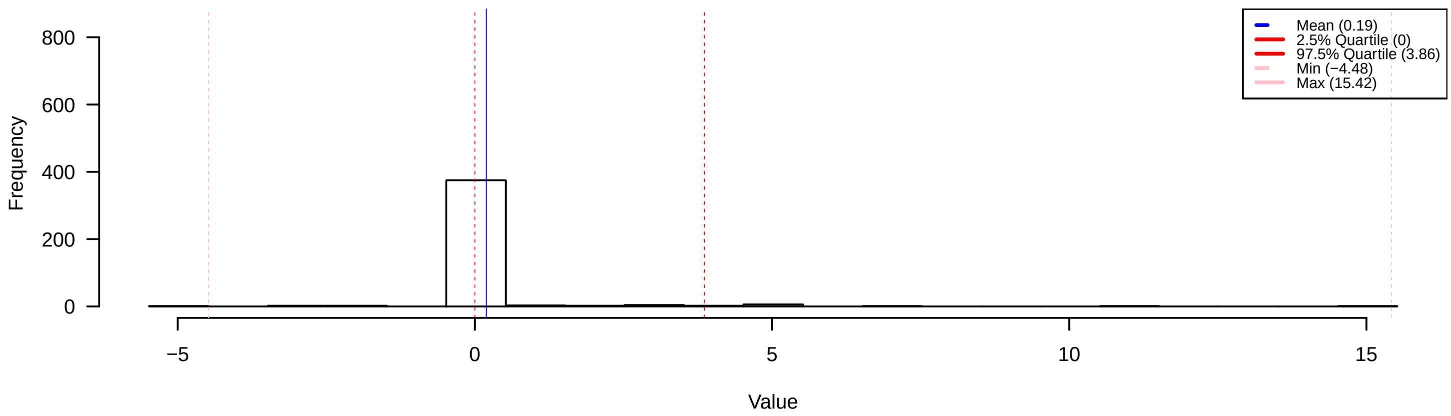

S5, Figure 123 : Bootstrap Distribution of Dewpoint Temperature:Air Temperature lag 2

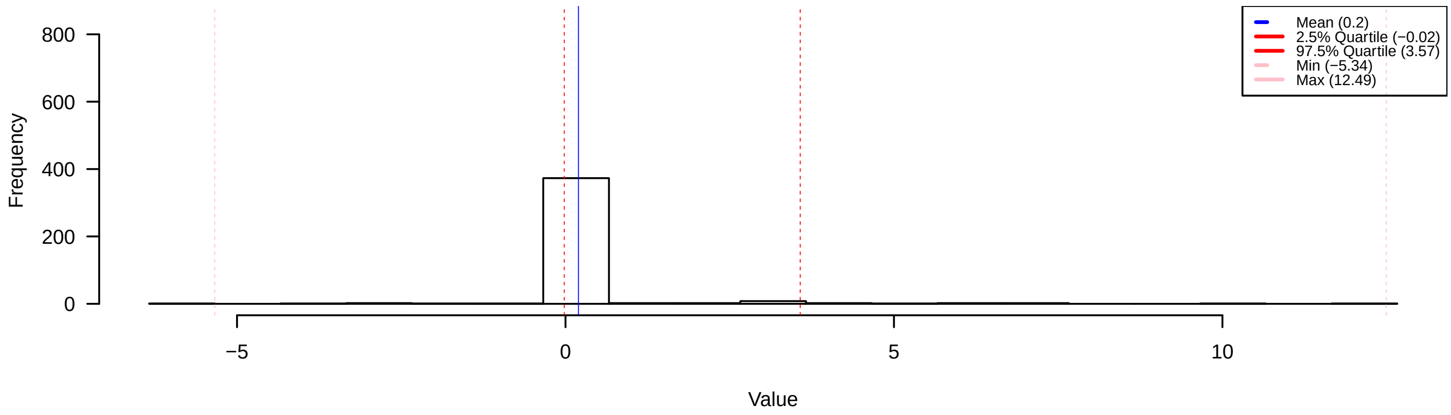

S5, Figure 124 : Bootstrap Distribution of Dewpoint Temperature:Air Temperature lag 3

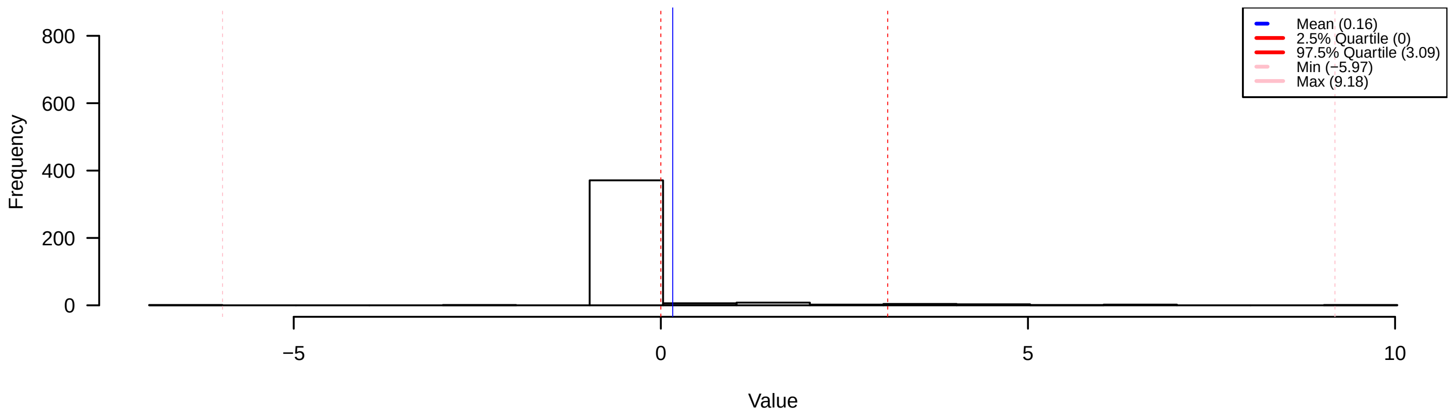

S5, Figure 125 : Bootstrap Distribution of Dewpoint Temperature:Air Temperature lag 4

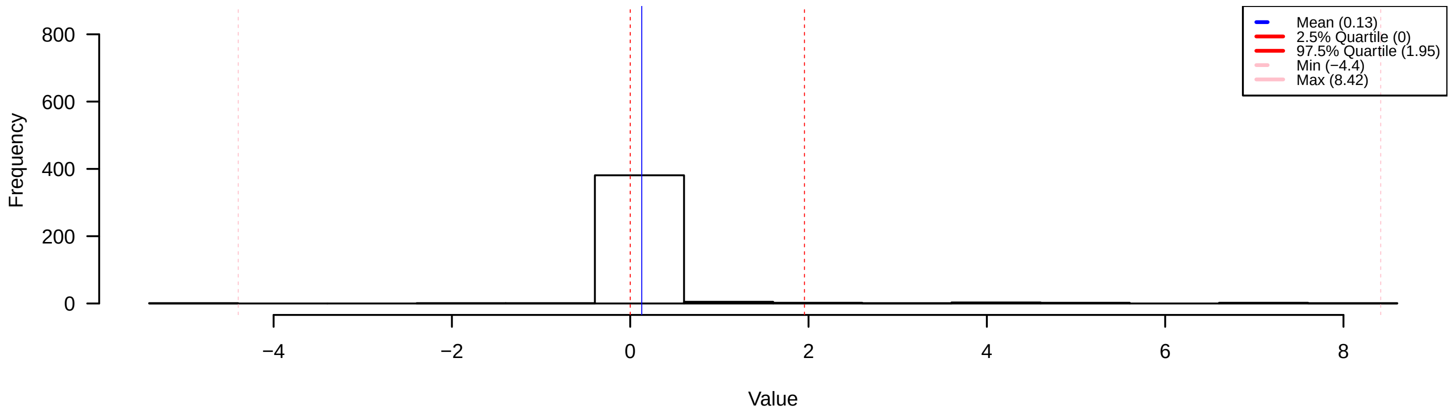

S5, Figure 126 : Bootstrap Distribution of Dewpoint Temperature:Air Temperature lag 5

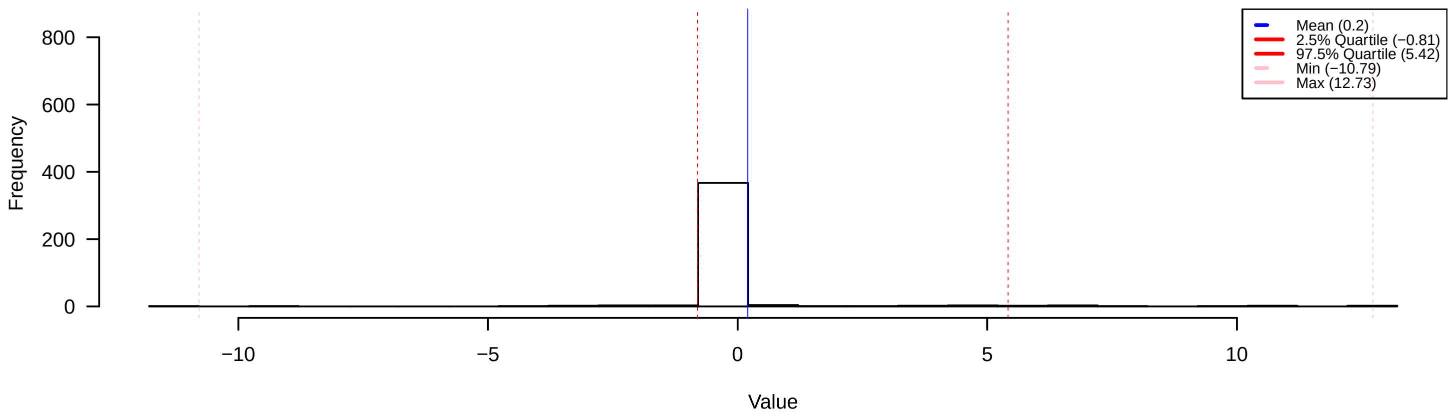

S5, Figure 127 : Bootstrap Distribution of Dewpoint Temperature:Air Temperature lag 6

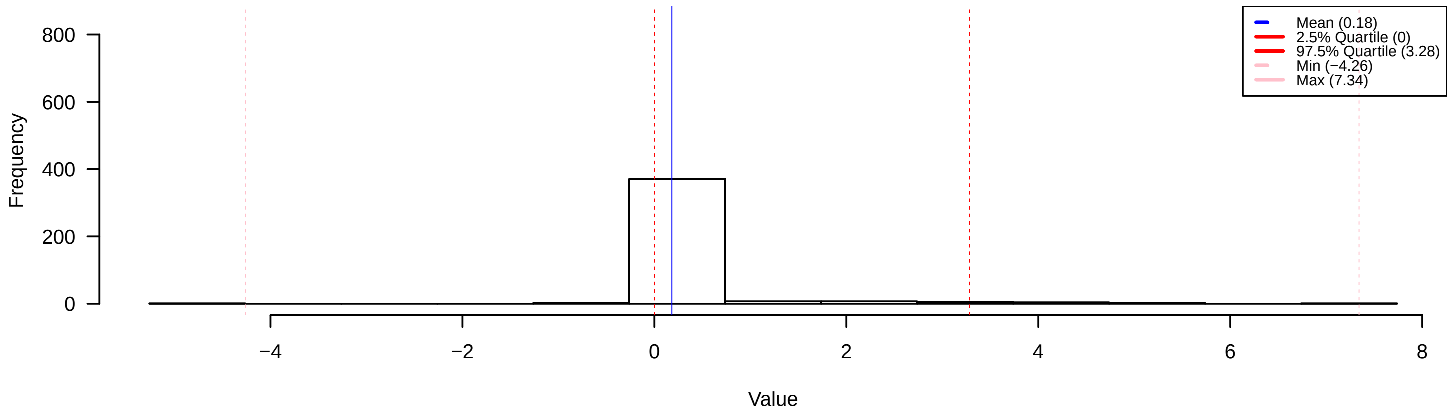

S5, Figure 128 : Bootstrap Distribution of Dewpoint Temperature:Air Temperature lag 7

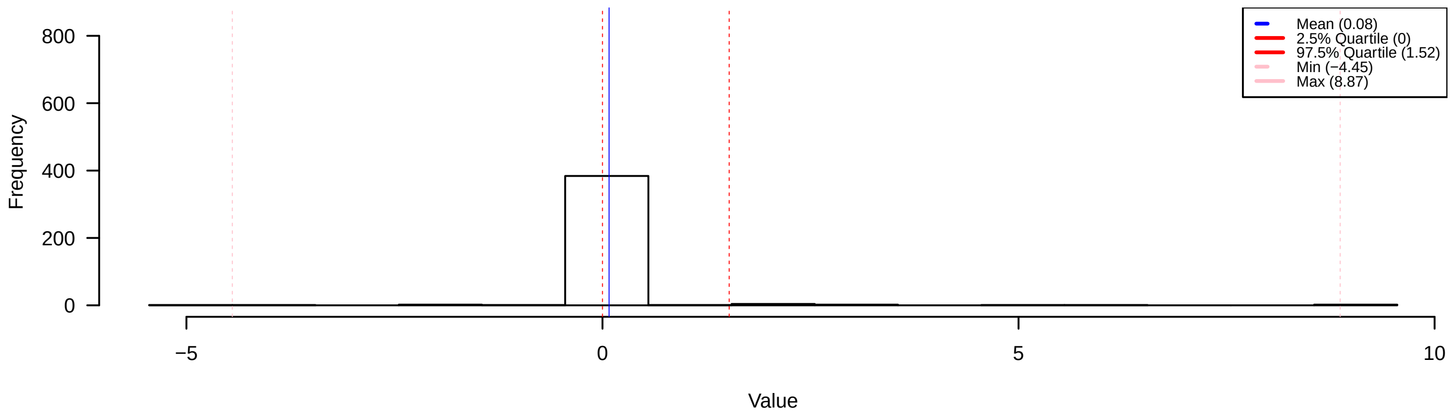

S5, Figure 129 : Bootstrap Distribution of Dewpoint Temperature:Air Temperature lag 8

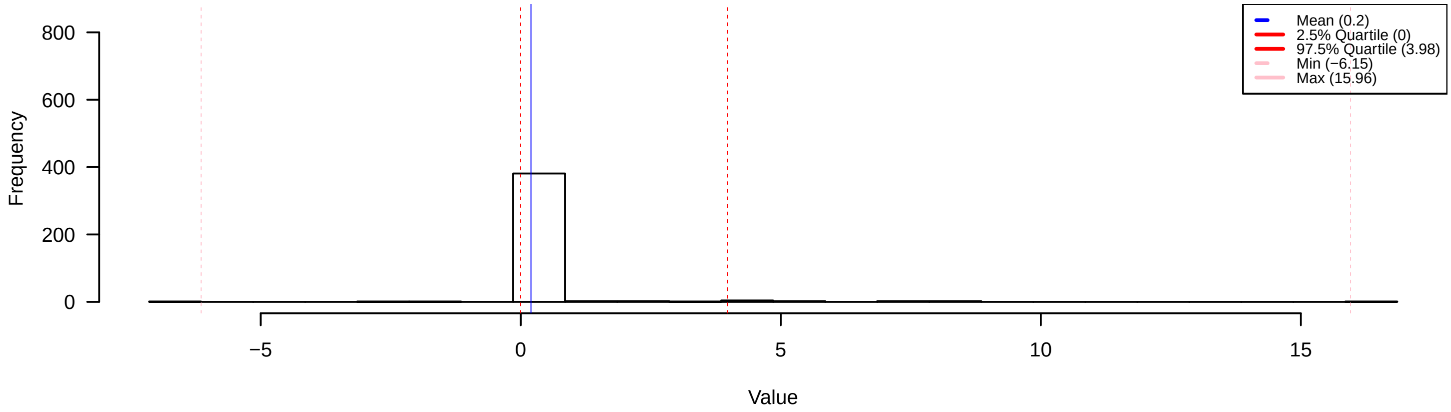

S5, Figure 130 : Bootstrap Distribution of Dewpoint Temperature:Air Temperature lag 9

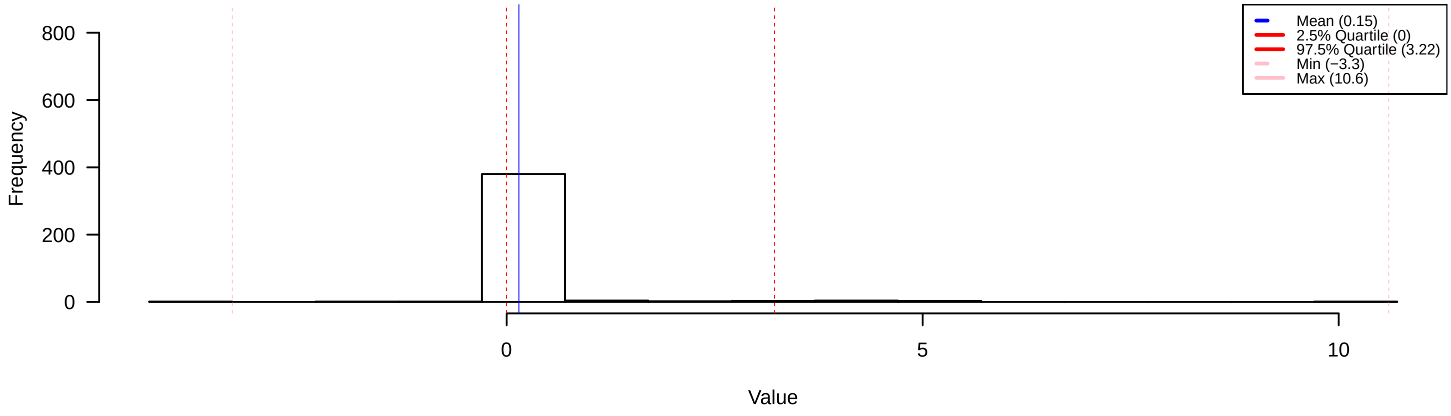

S5, Figure 131 : Bootstrap Distribution of Dewpoint Temperature:Air Temperature lag 10

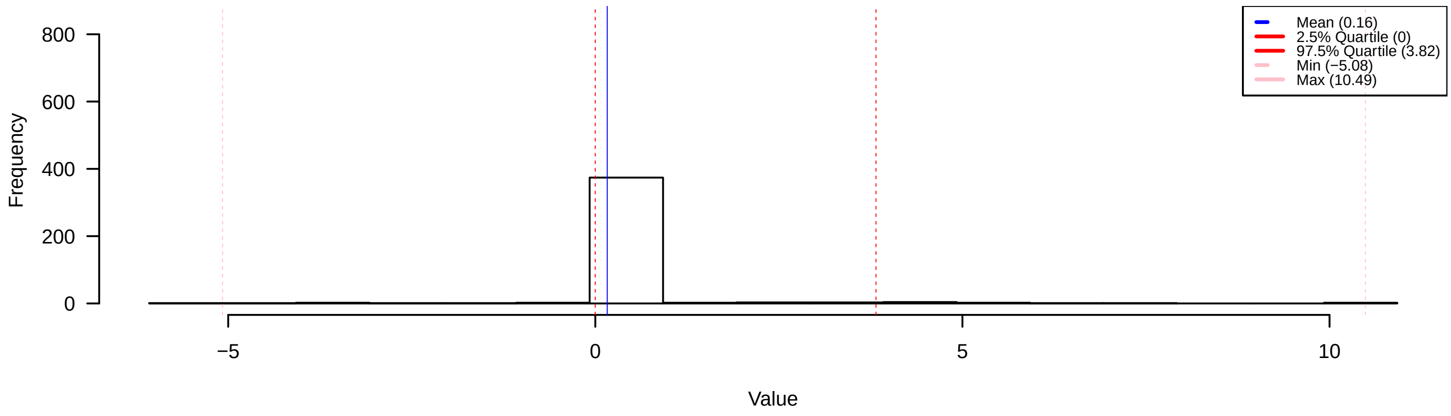

S5, Figure 132 : Bootstrap Distribution of Dewpoint Temperature:Air Temperature lag 11

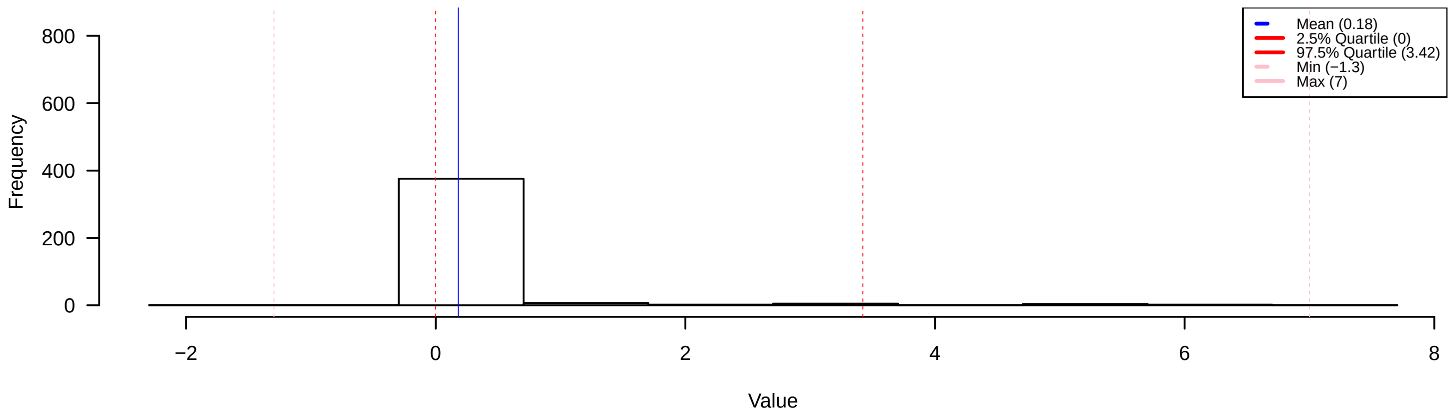

S5, Figure 133 : Bootstrap Distribution of Dewpoint Temperature:Air Temperature lag 12

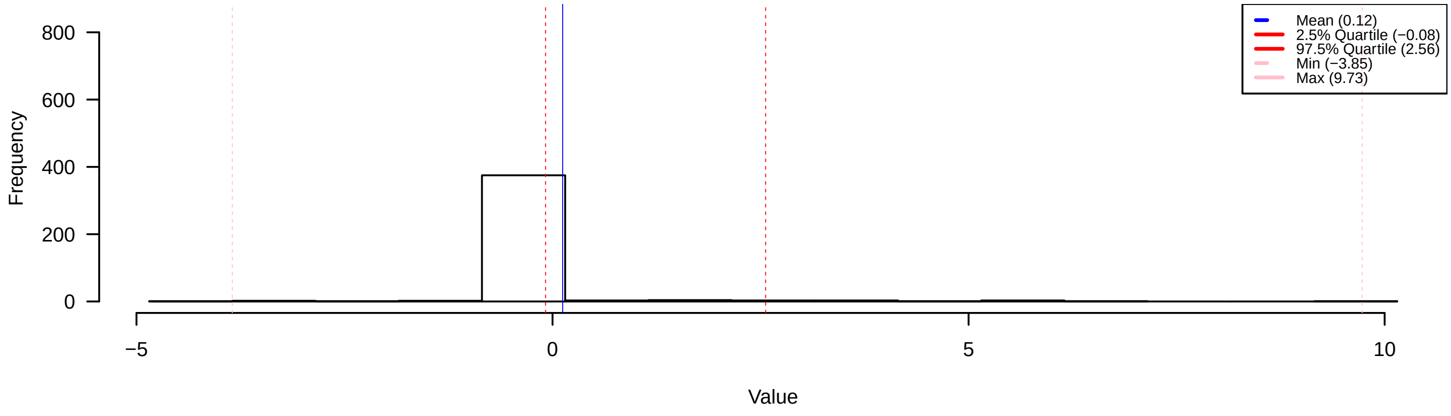

S5, Figure 134 : Bootstrap Distribution of Dewpoint Temperature:Air Temperature lag 13

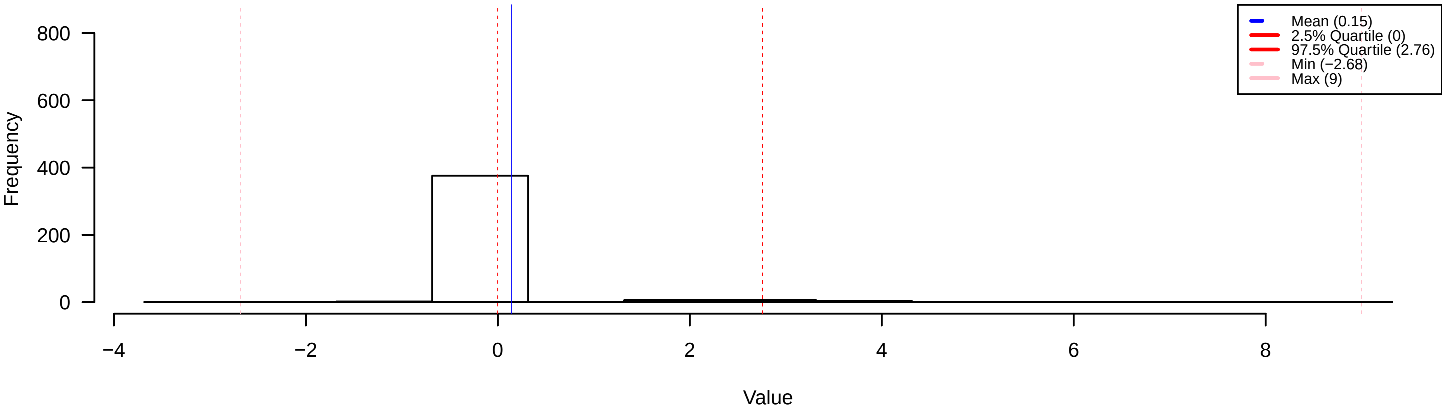

S5, Figure 135 : Bootstrap Distribution of Dewpoint Temperature:Air Temperature lag 14

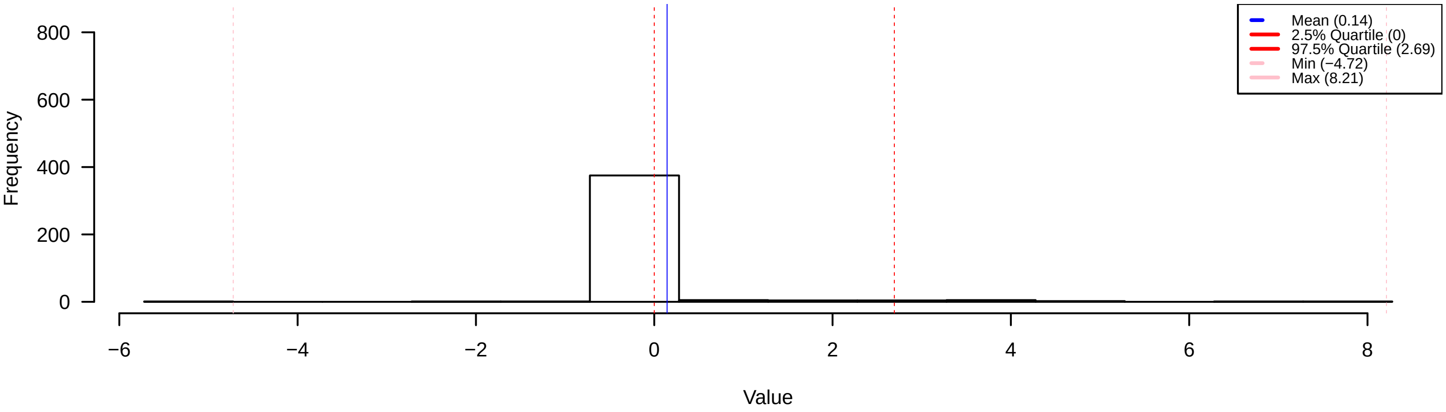

S5, Figure 136 : Bootstrap Distribution of Dewpoint Temperature:Air Temperature lag 15

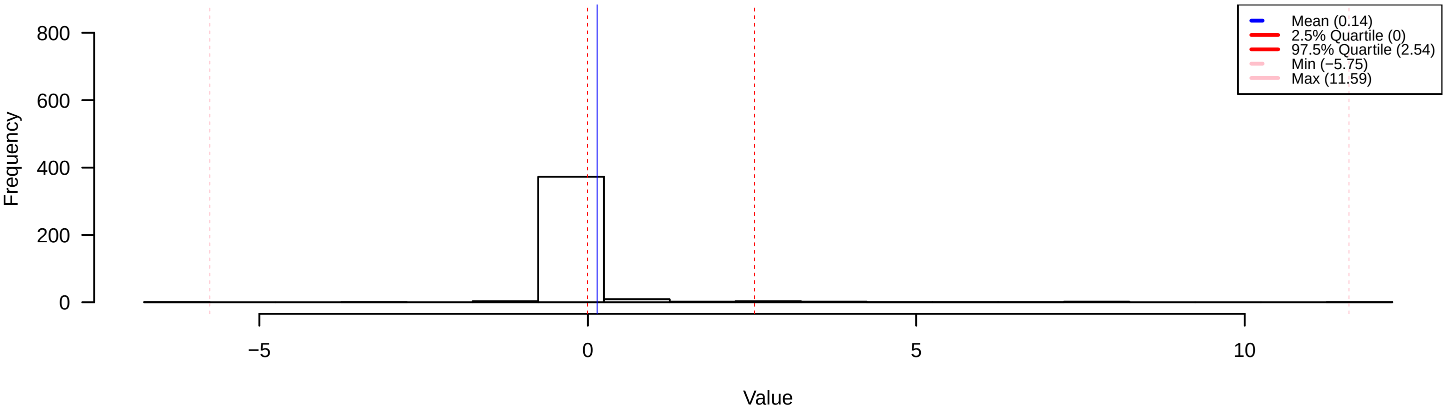

S5, Figure 137 : Bootstrap Distribution of Dewpoint Temperature:Air Temperature lag 16

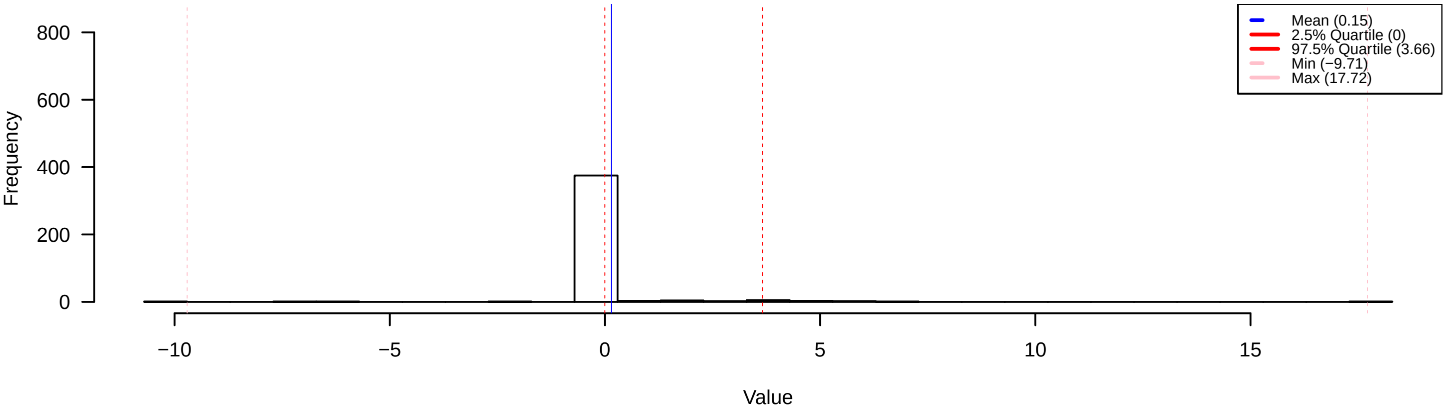

S5, Figure 138 : Bootstrap Distribution of Dewpoint Temperature:Air Temperature lag 17

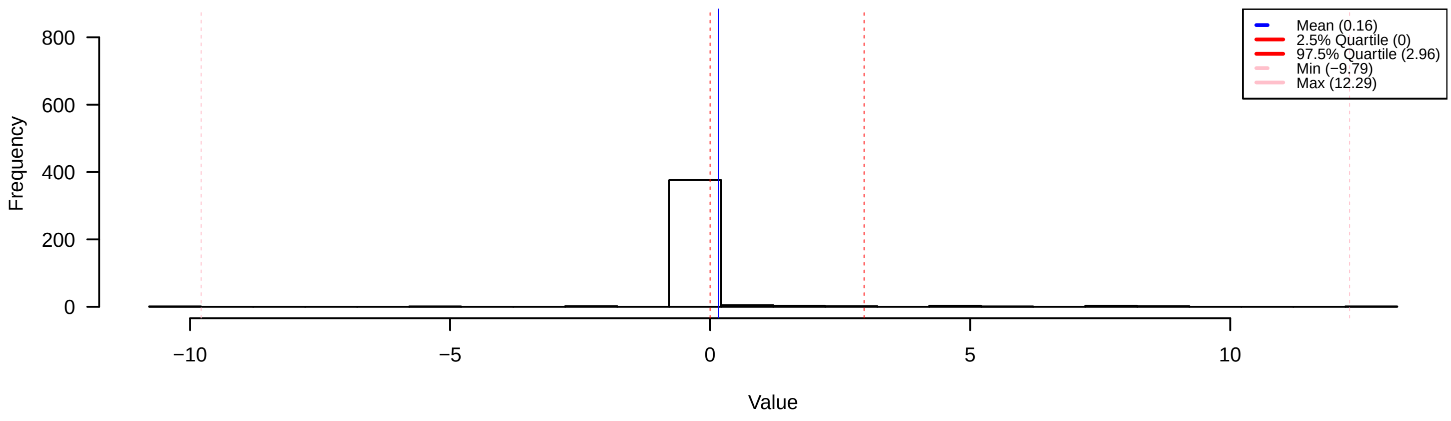

S5, Figure 139 : Bootstrap Distribution of Dewpoint Temperature:Air Temperature lag 18

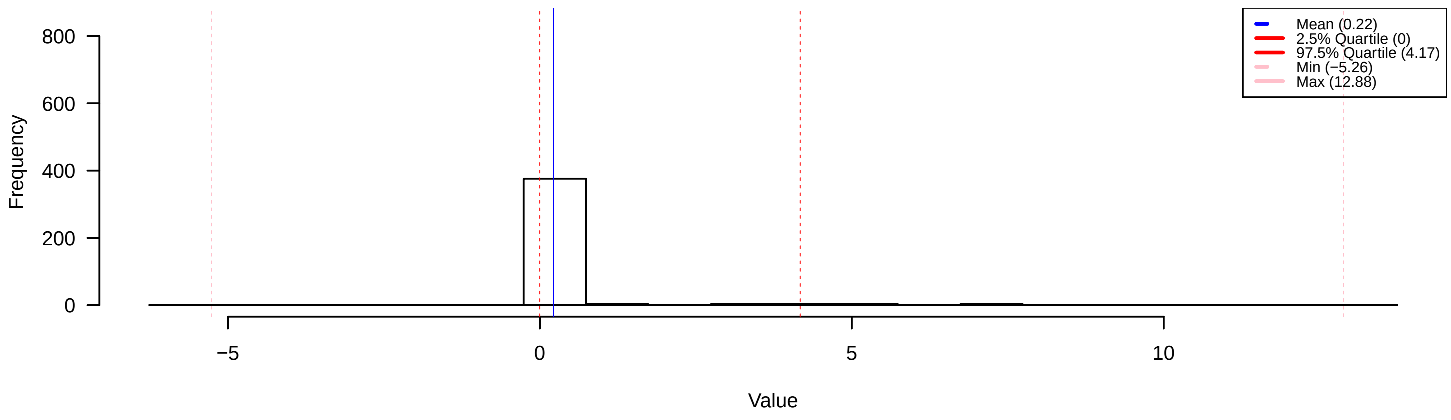

S5, Figure 140 : Bootstrap Distribution of Dewpoint Temperature:Air Temperature lag 19

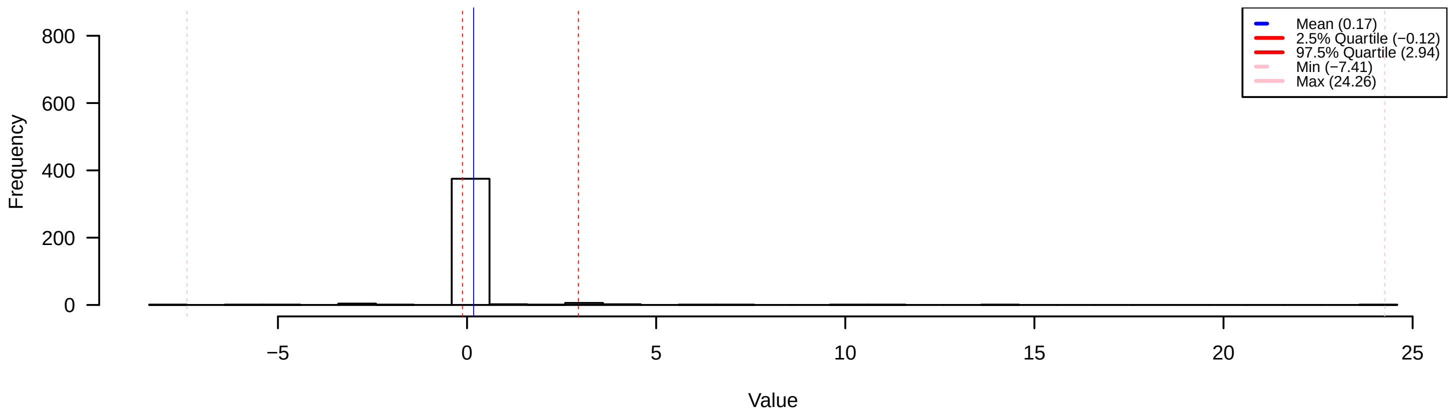

S5, Figure 141 : Bootstrap Distribution of Dewpoint Temperature:Air Temperature lag 20

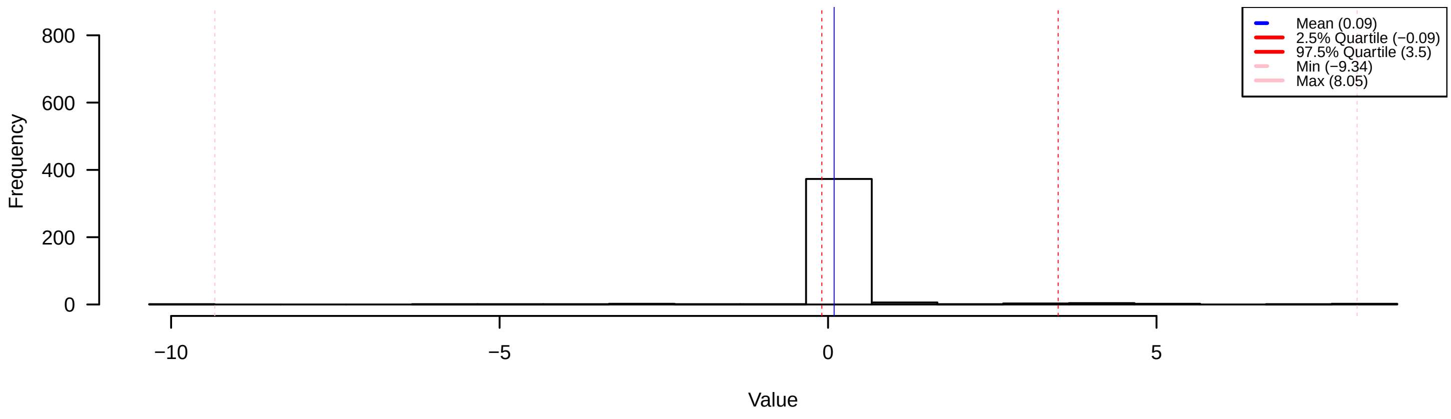

S5, Figure 142 : Bootstrap Distribution of Dewpoint Temperature:Absolute Humidity lag 1

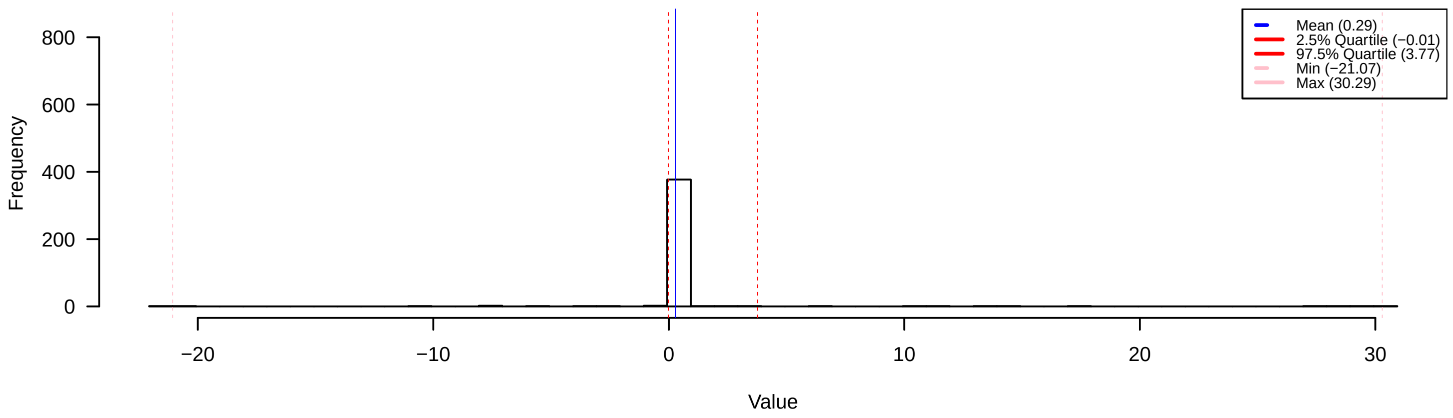

S5, Figure 143 : Bootstrap Distribution of Dewpoint Temperature:Absolute Humidity lag 2

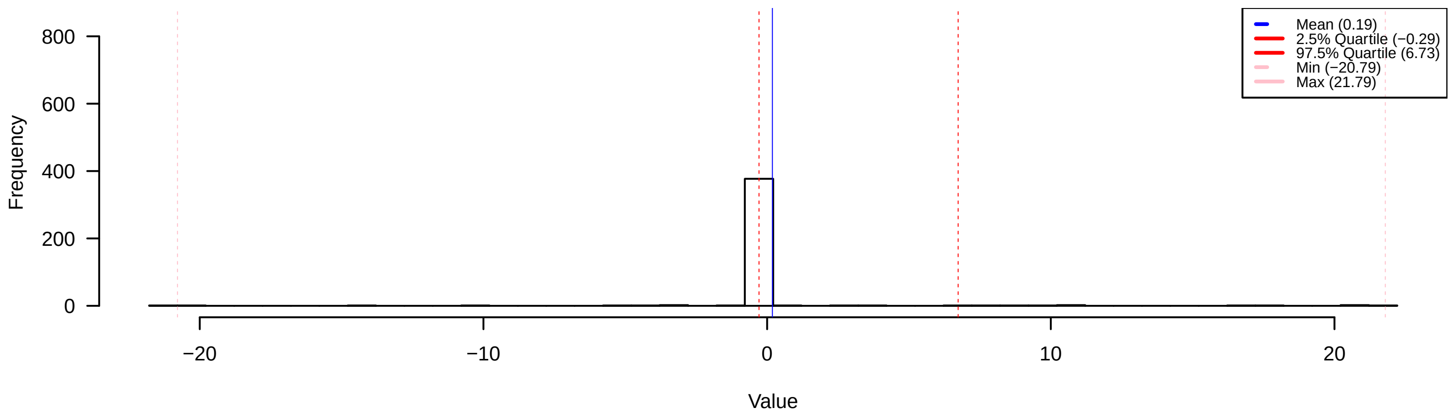

S5, Figure 144 : Bootstrap Distribution of Dewpoint Temperature:Absolute Humidity lag 3

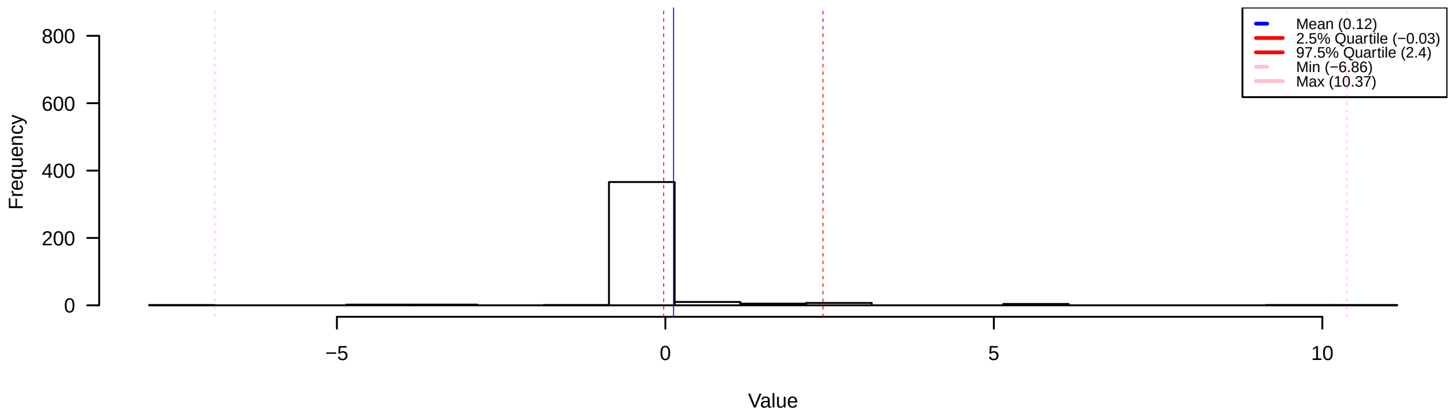

S5, Figure 145 : Bootstrap Distribution of Dewpoint Temperature:Absolute Humidity lag 4

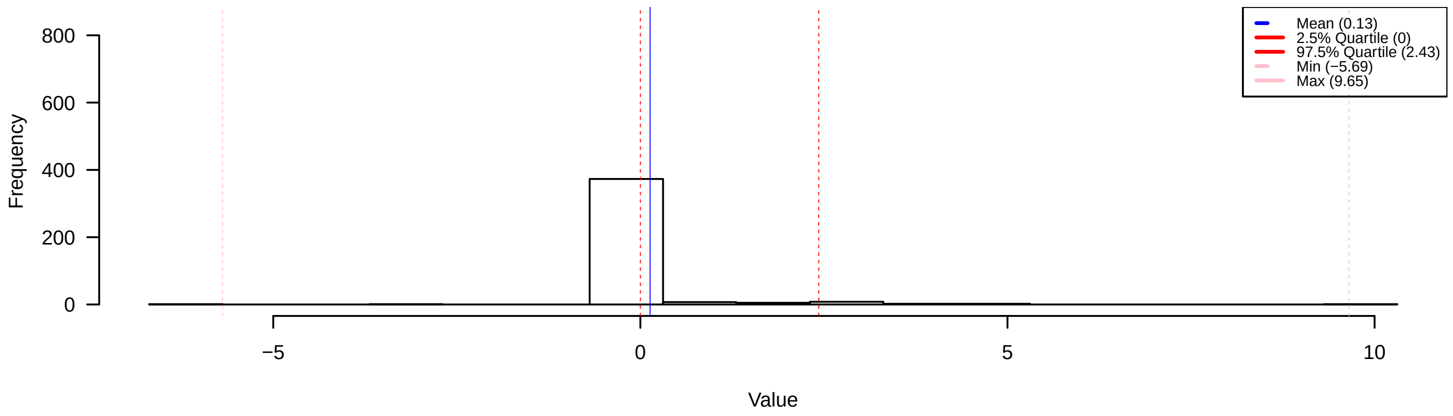

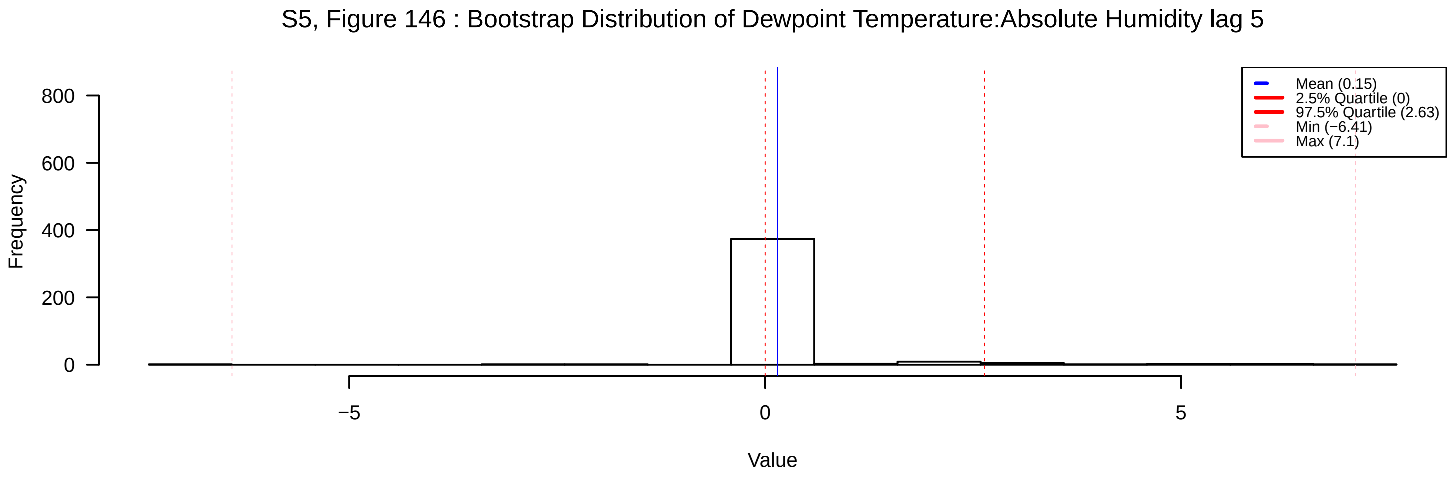
S5, Figure 146 : Bootstrap Distribution of Dewpoint Temperature:Absolute Humidity lag 5
Mean (0.15)
2.5% Quartile (0)
97.5% Quartile (2.63)
Min (−6.41)
Max (7.1)
Frequency
Value

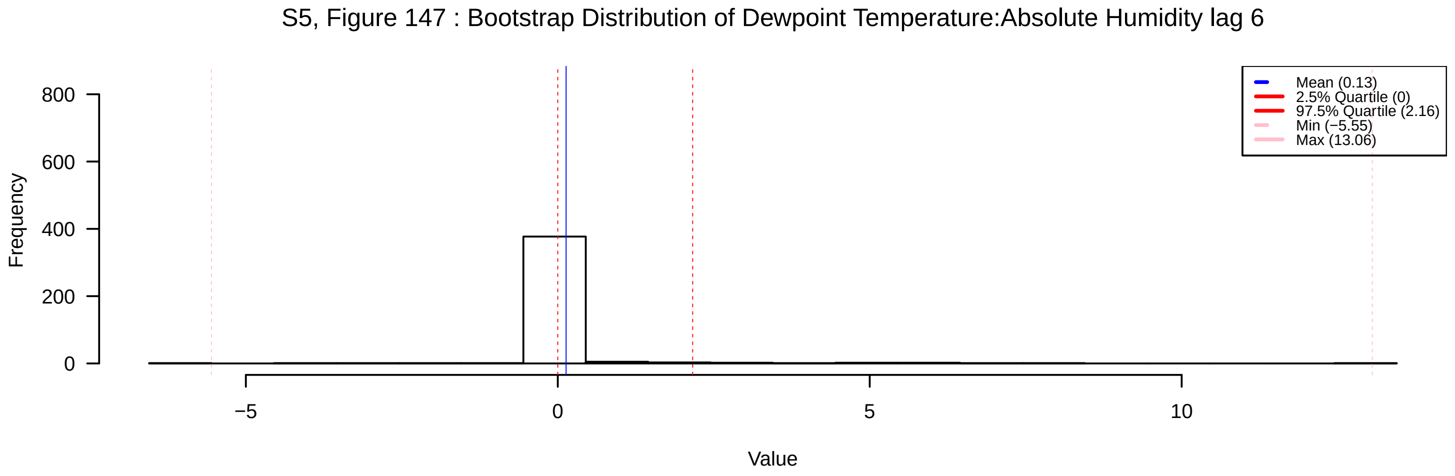
S5, Figure 147 : Bootstrap Distribution of Dewpoint Temperature:Absolute Humidity lag 6
Mean (0.13)
2.5% Quartile (0)
97.5% Quartile (2.16)
Min (−5.55)
Max (13.06)
Frequency
Value

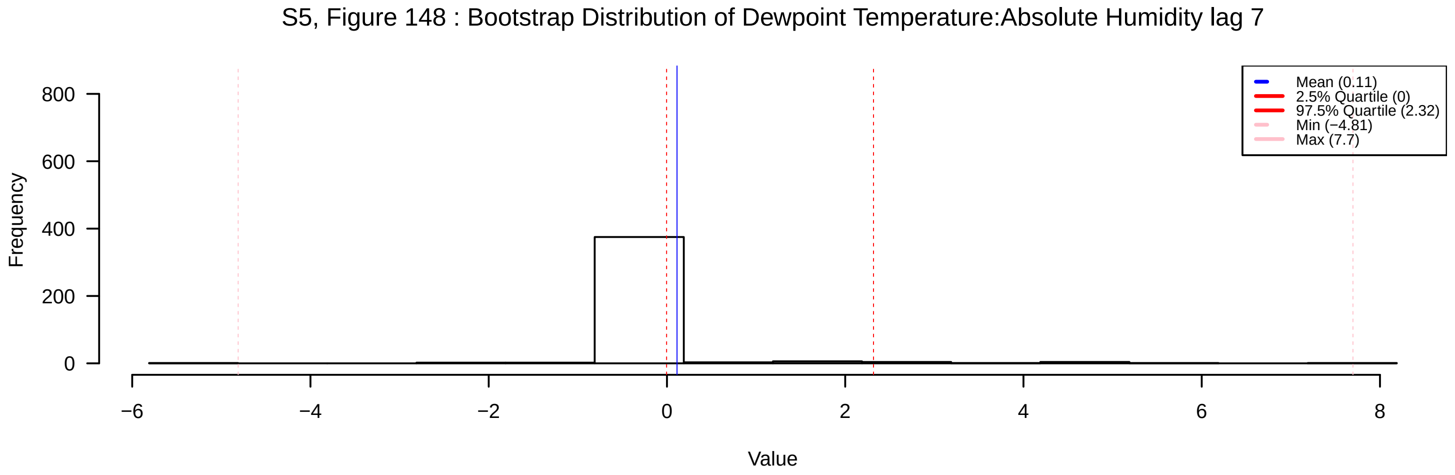
S5, Figure 148 : Bootstrap Distribution of Dewpoint Temperature:Absolute Humidity lag 7
Mean (0.11)
2.5% Quartile (0)
97.5% Quartile (2.32)
Min (−4.81)
Max (7.7)
Frequency
Value

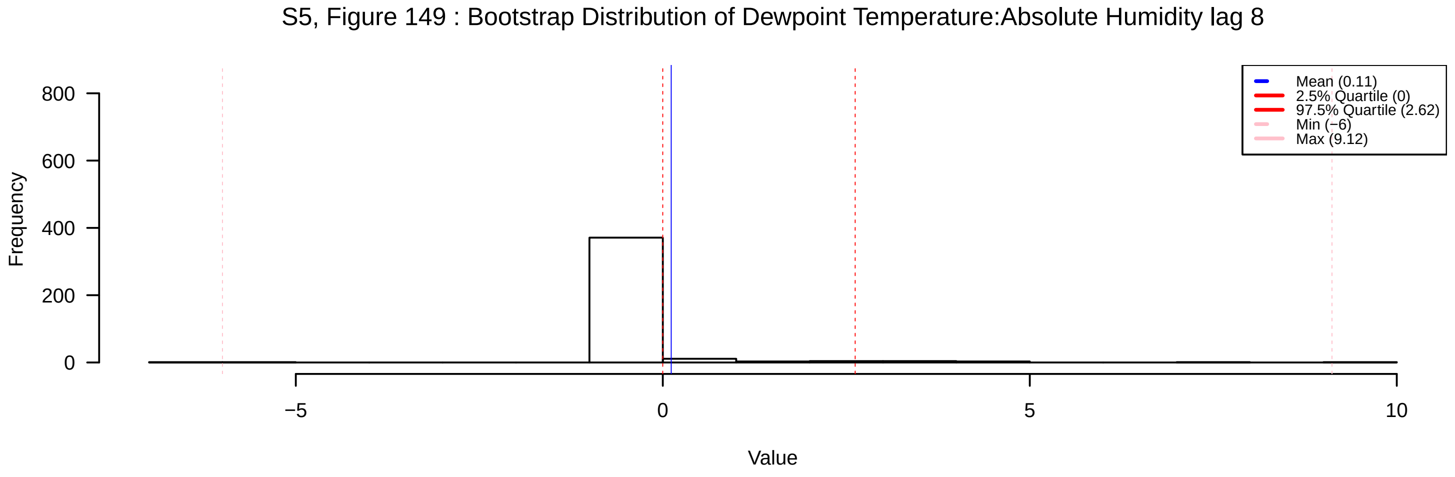
S5, Figure 149 : Bootstrap Distribution of Dewpoint Temperature:Absolute Humidity lag 8
Mean (0.11)
2.5% Quartile (0)
97.5% Quartile (2.62)
Min (−6)
Max (9.12)
Frequency
Value

S5, Figure 150 : Bootstrap Distribution of Dewpoint Temperature:Absolute Humidity lag 9

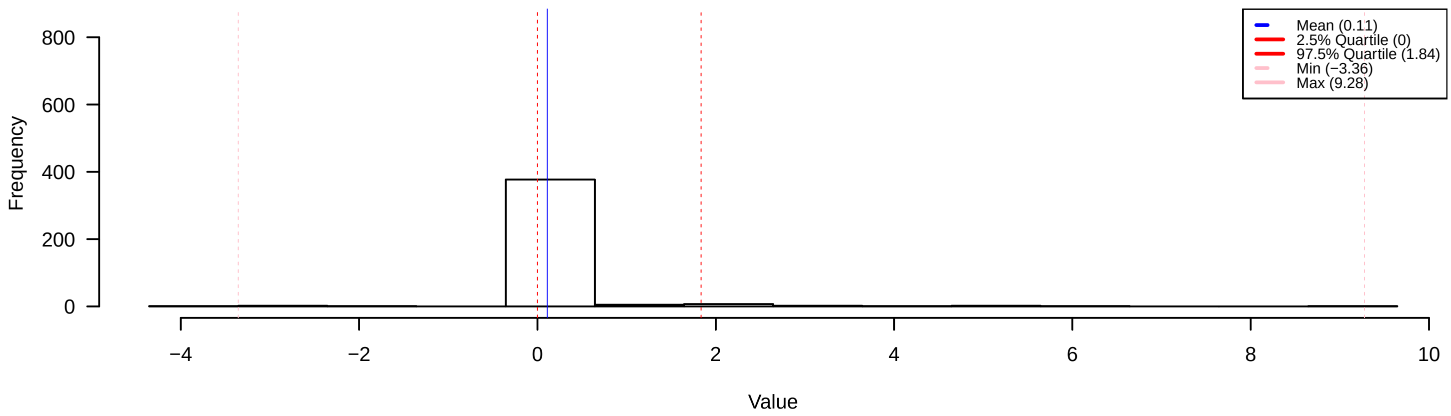

S5, Figure 151 : Bootstrap Distribution of Dewpoint Temperature:Absolute Humidity lag 10

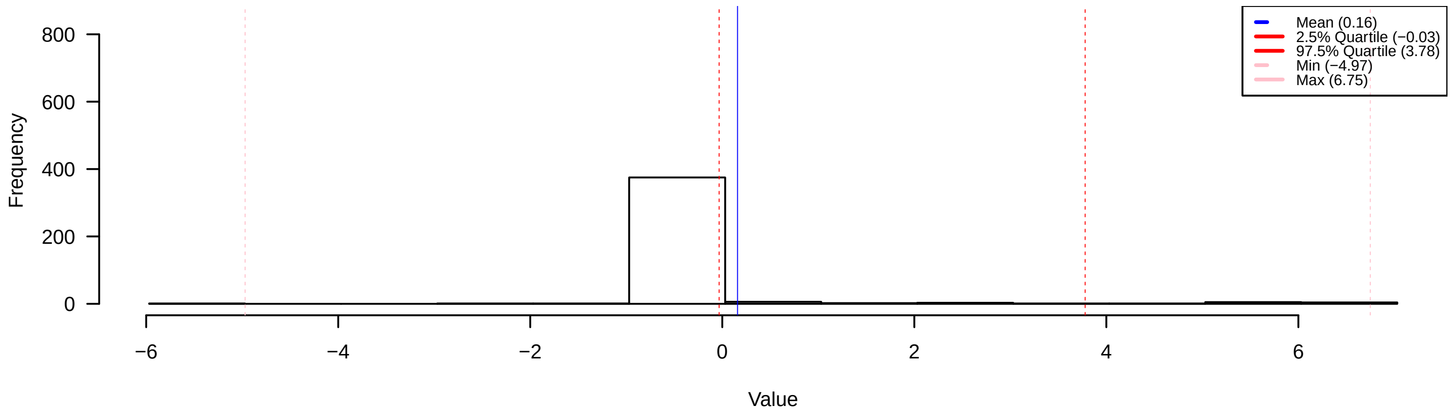

S5, Figure 152 : Bootstrap Distribution of Dewpoint Temperature:Absolute Humidity lag 11

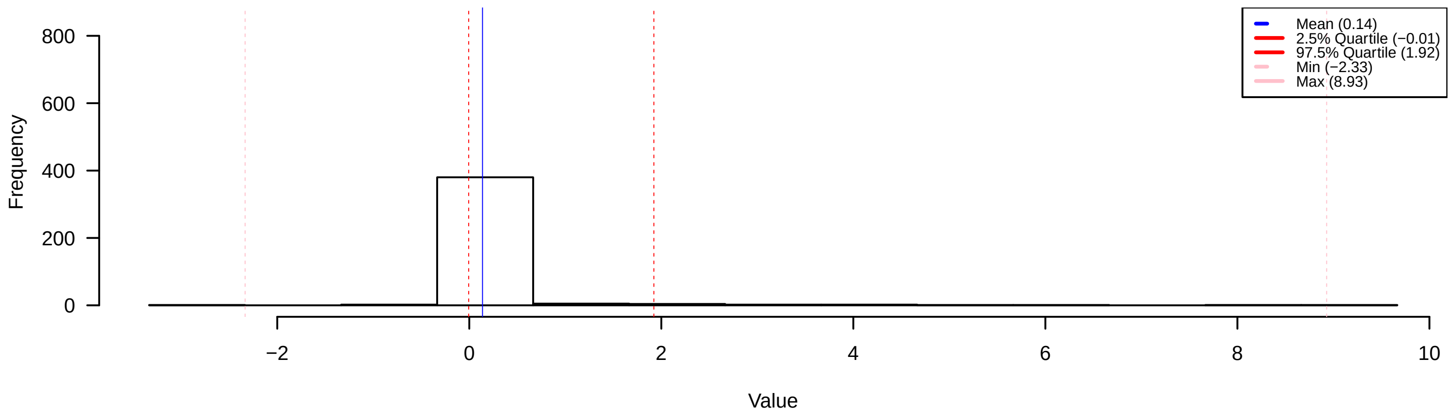

S5, Figure 153 : Bootstrap Distribution of Dewpoint Temperature:Absolute Humidity lag 12

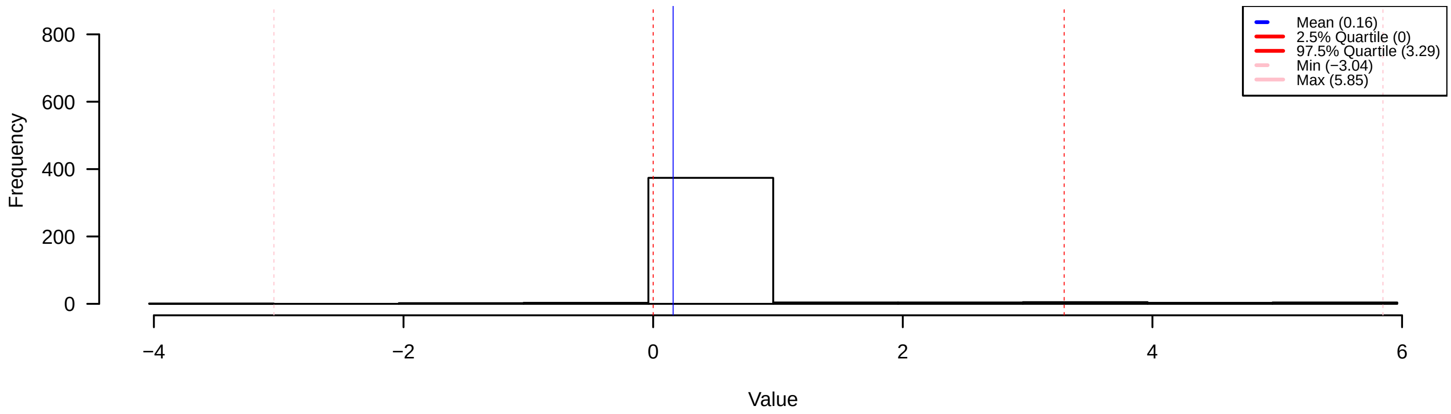

S5, Figure 154 : Bootstrap Distribution of Dewpoint Temperature:Absolute Humidity lag 13

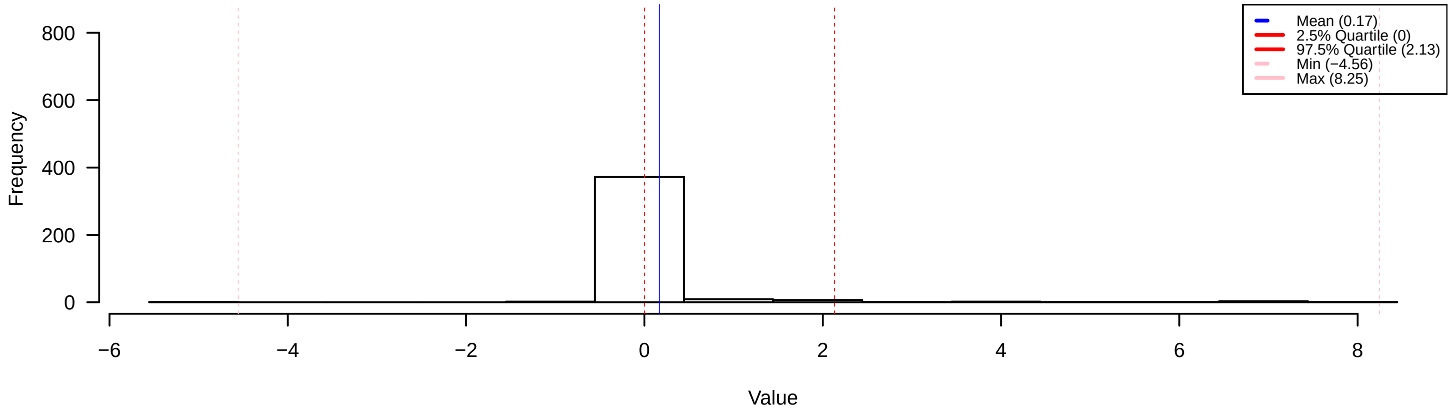

S5, Figure 155 : Bootstrap Distribution of Dewpoint Temperature:Absolute Humidity lag 14

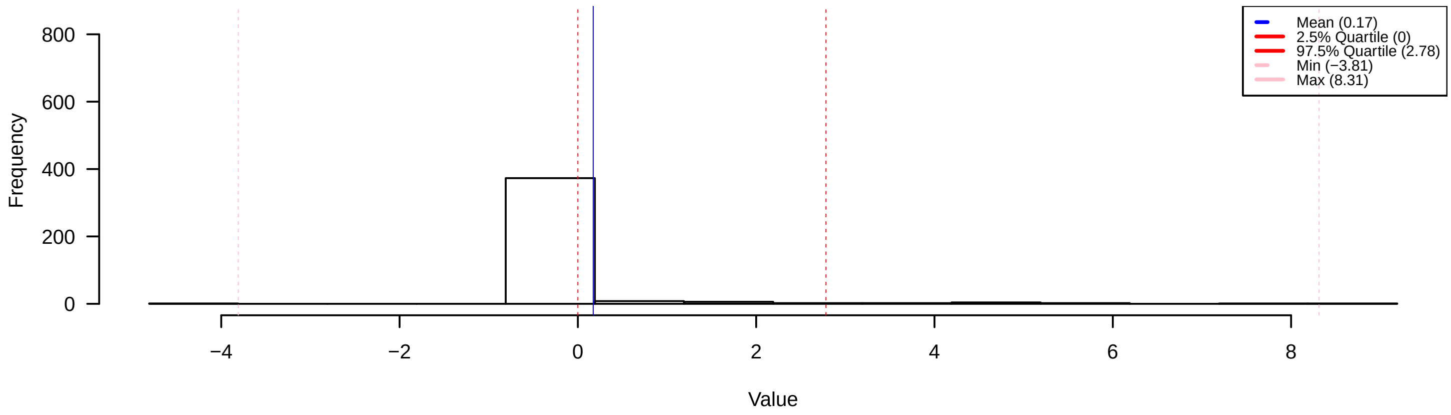

S5, Figure 156 : Bootstrap Distribution of Dewpoint Temperature:Absolute Humidity lag 15

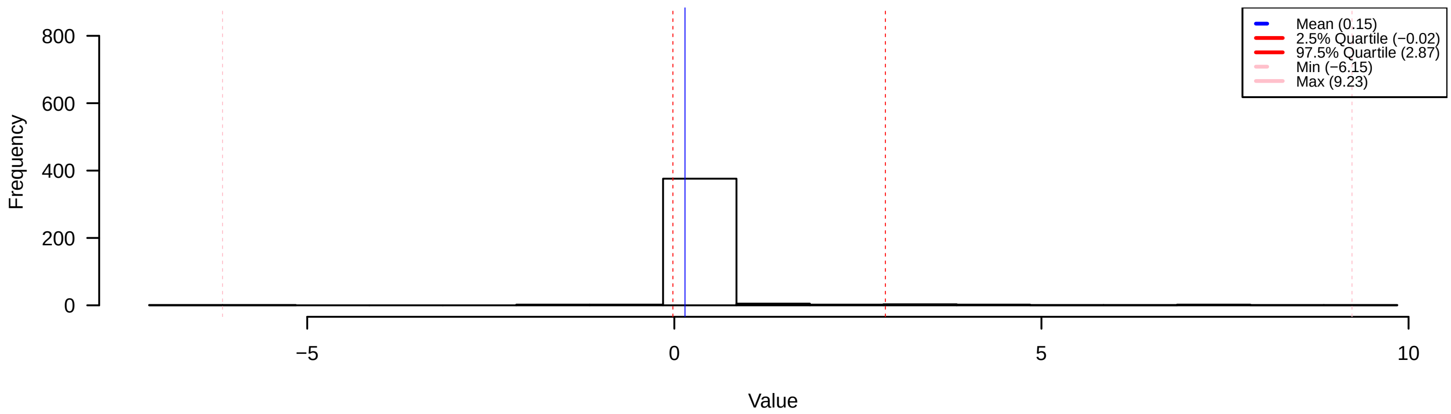

S5, Figure 157 : Bootstrap Distribution of Dewpoint Temperature:Absolute Humidity lag 16

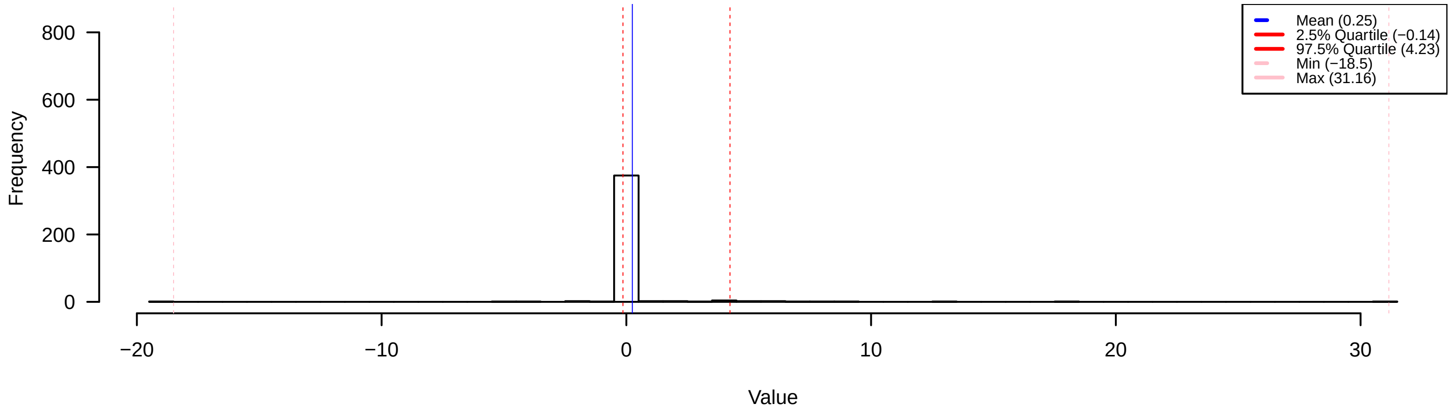

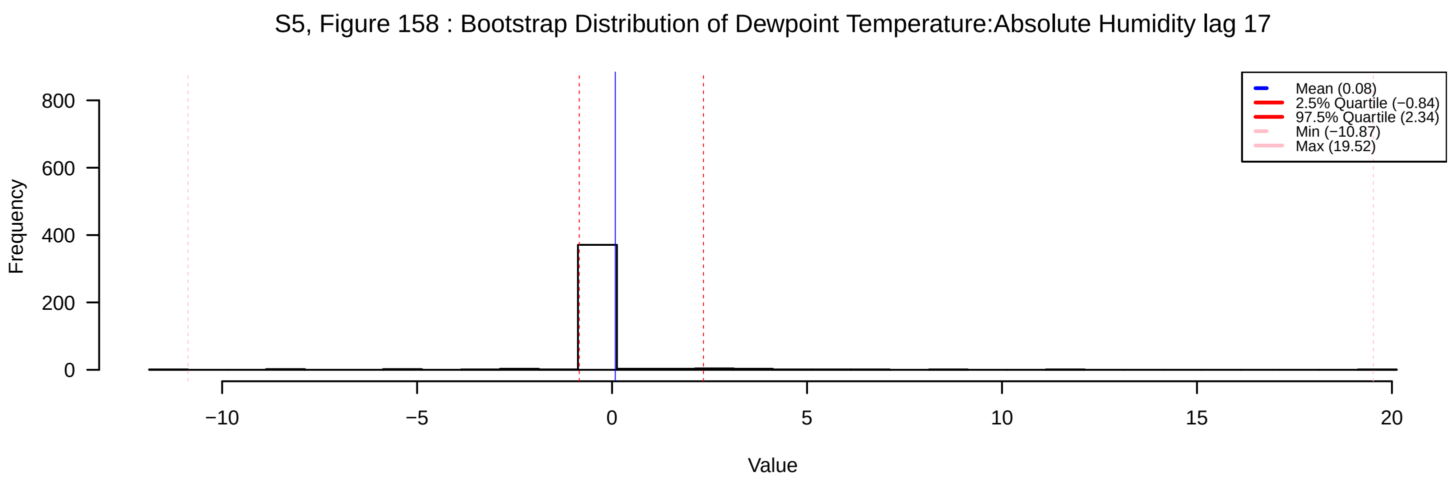
S5, Figure 158 : Bootstrap Distribution of Dewpoint Temperature:Absolute Humidity lag 17
Mean (0.08)
2.5% Quartile (−0.84)
97.5% Quartile (2.34)
Min (−10.87)
Max (19.52)
Frequency
Value

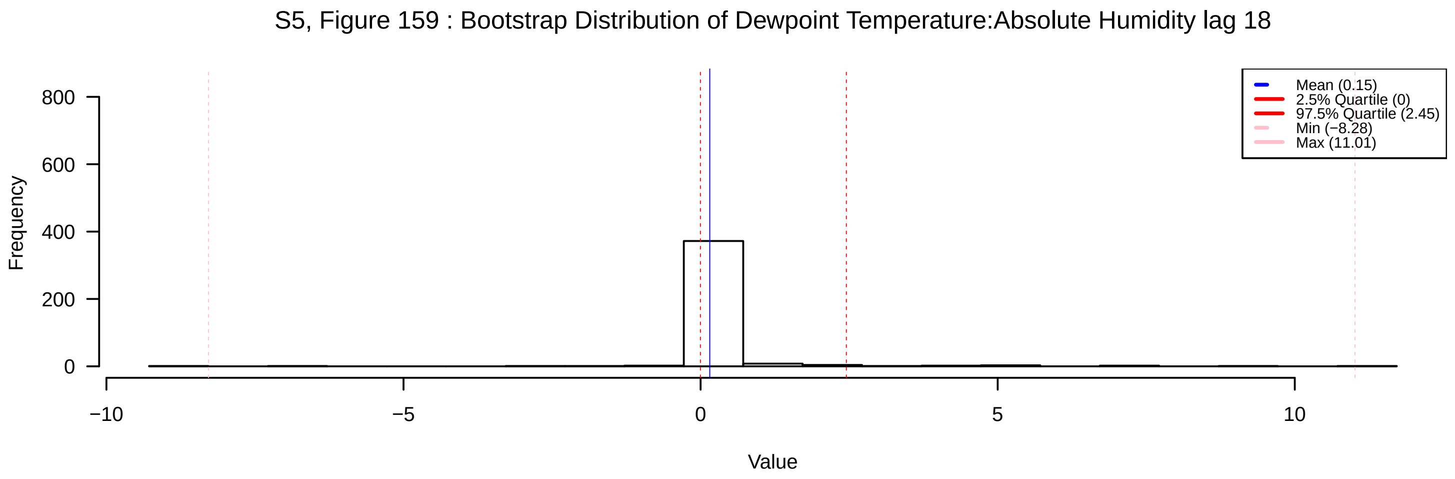
S5, Figure 159 : Bootstrap Distribution of Dewpoint Temperature:Absolute Humidity lag 18
Mean (0.15)
2.5% Quartile (0)
97.5% Quartile (2.45)
Min (−8.28)
Max (11.01)
Frequency
Value

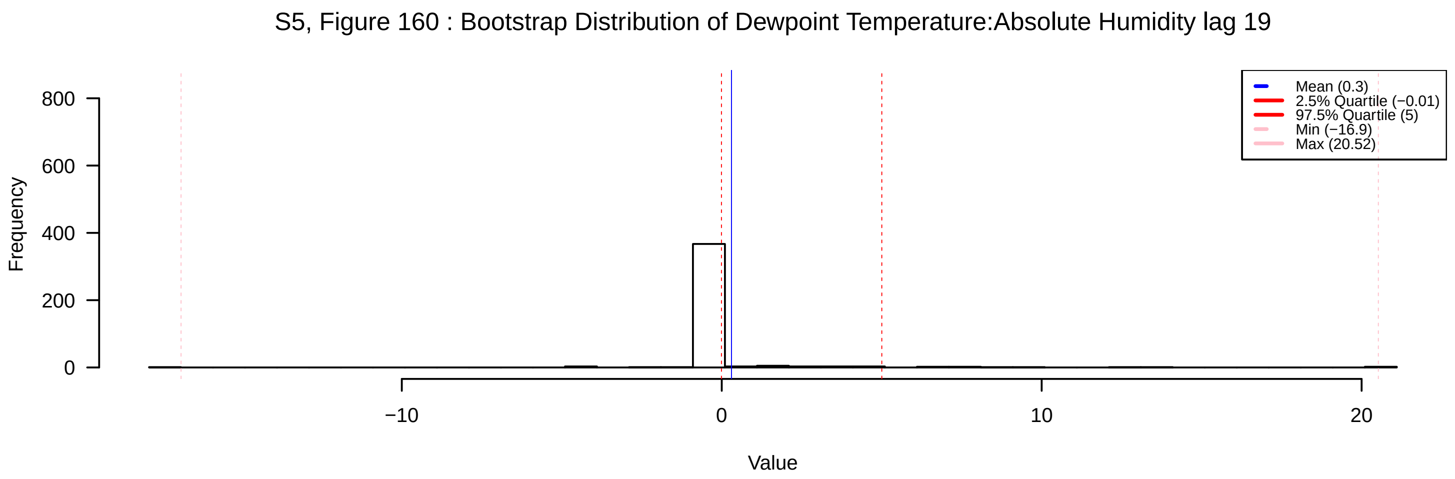
S5, Figure 160 : Bootstrap Distribution of Dewpoint Temperature:Absolute Humidity lag 19
Mean (0.3)
2.5% Quartile (−0.01)
97.5% Quartile (5)
Min (−16.9)
Max (20.52)
Frequency
Value

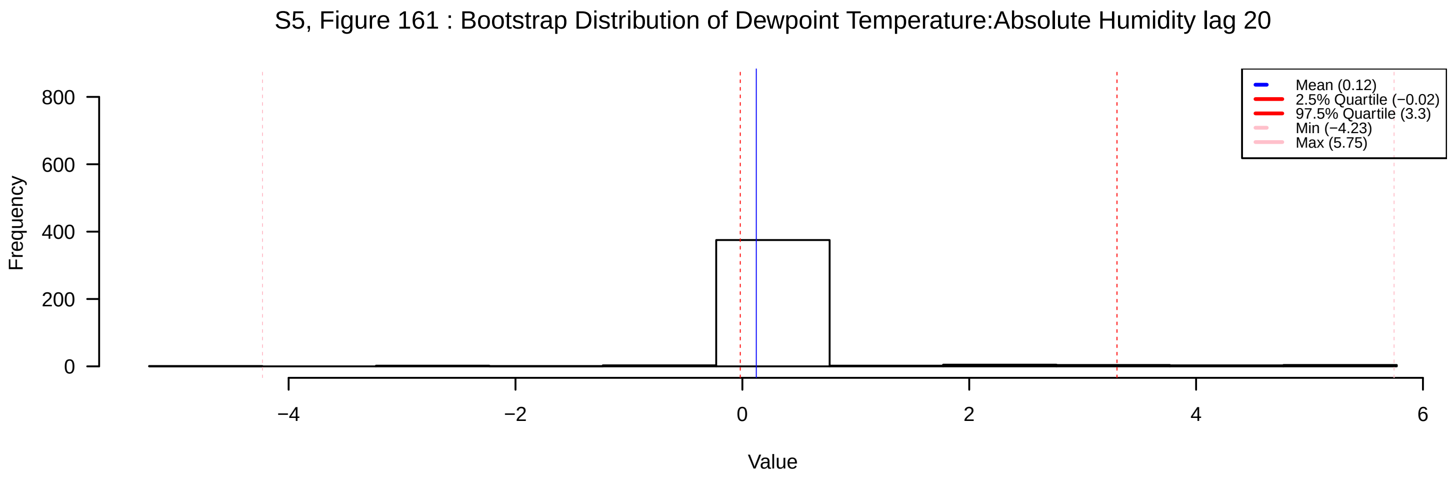
S5, Figure 161 : Bootstrap Distribution of Dewpoint Temperature:Absolute Humidity lag 20
Mean (0.12)
2.5% Quartile (−0.02)
97.5% Quartile (3.3)
Min (−4.23)
Max (5.75)
Frequency
Value

S5, Figure 162 : Bootstrap Distribution of Dewpoint Temperature:Relative Humidity lag 1

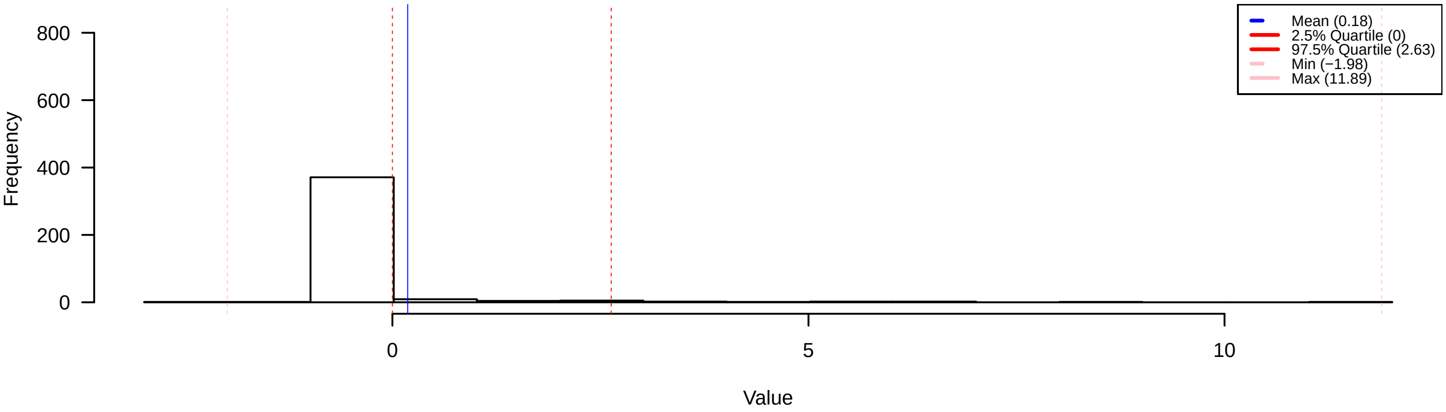

S5, Figure 163 : Bootstrap Distribution of Dewpoint Temperature:Relative Humidity lag 2

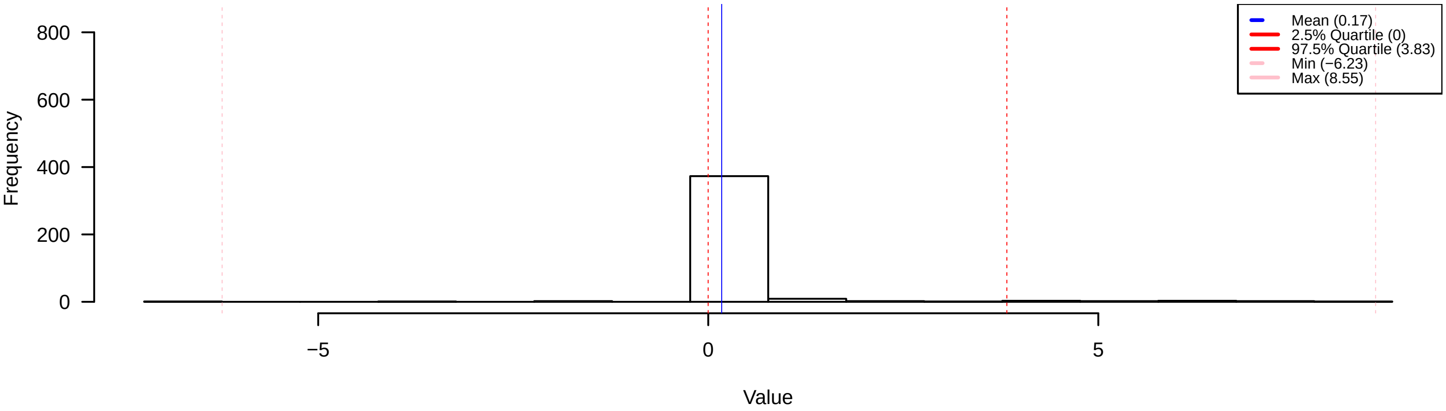

S5, Figure 164 : Bootstrap Distribution of Dewpoint Temperature:Relative Humidity lag 3

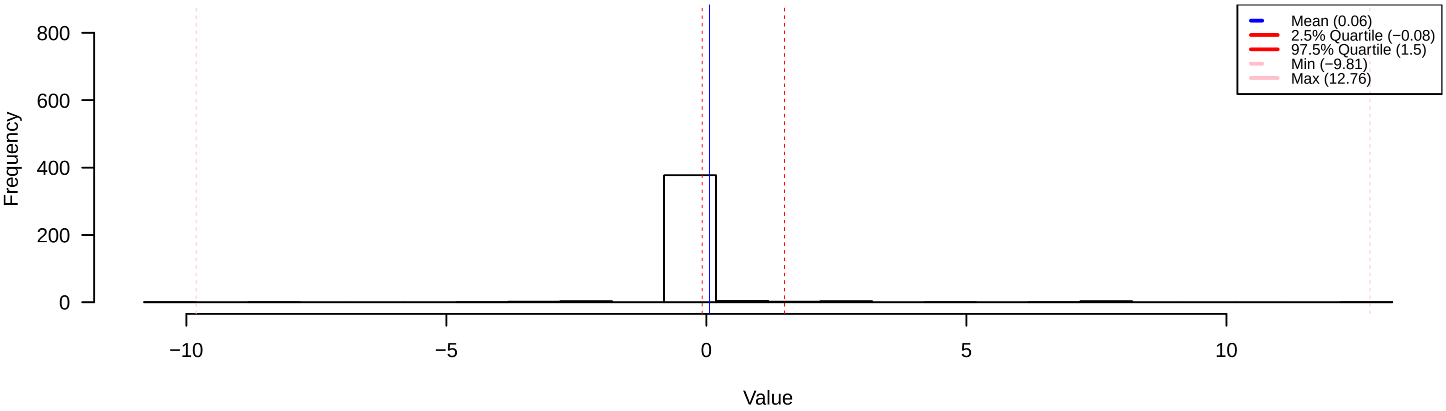

S5, Figure 165 : Bootstrap Distribution of Dewpoint Temperature:Relative Humidity lag 4

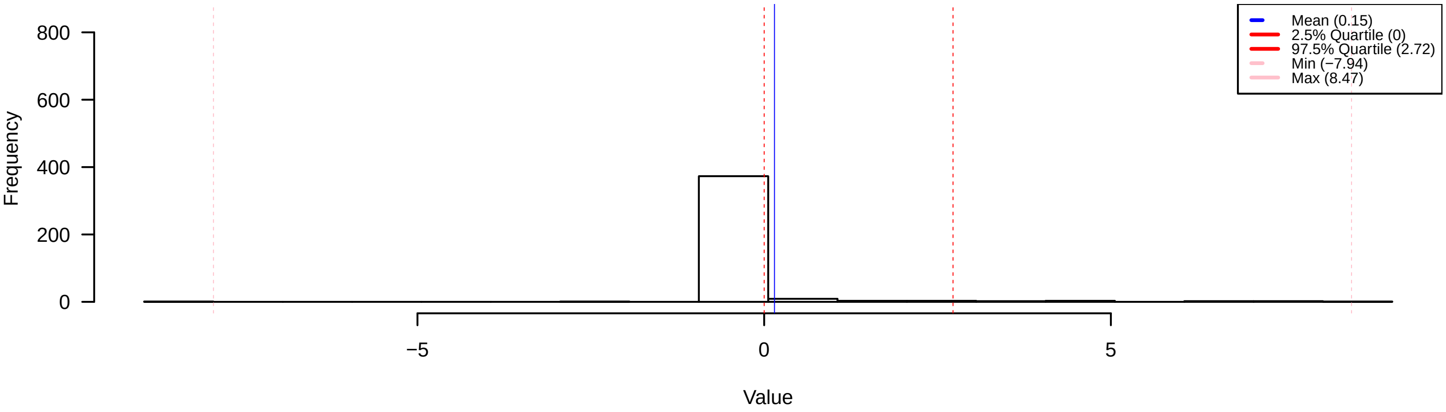

S5, Figure 166 : Bootstrap Distribution of Dewpoint Temperature:Relative Humidity lag 5

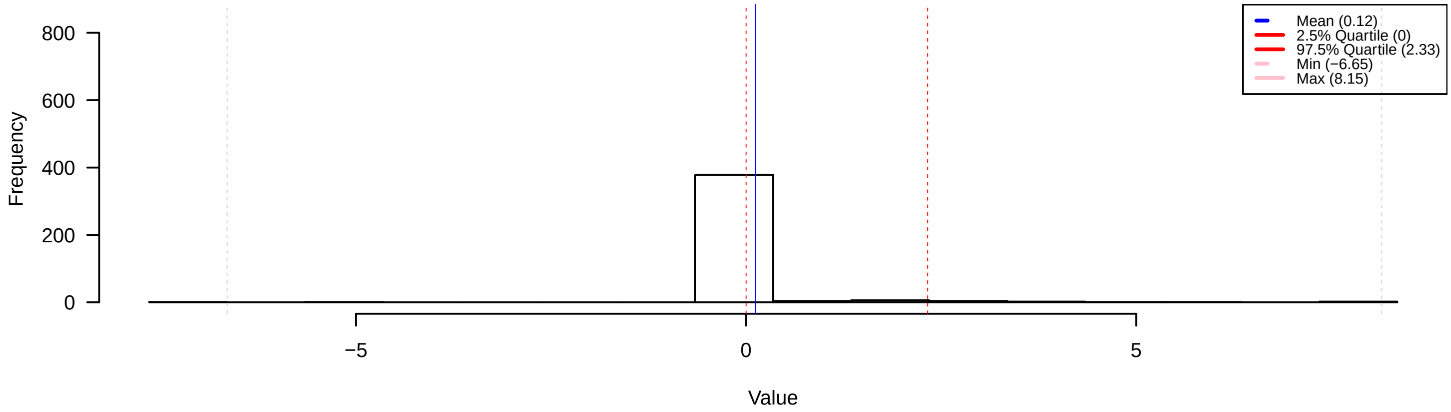

S5, Figure 167 : Bootstrap Distribution of Dewpoint Temperature:Relative Humidity lag 6

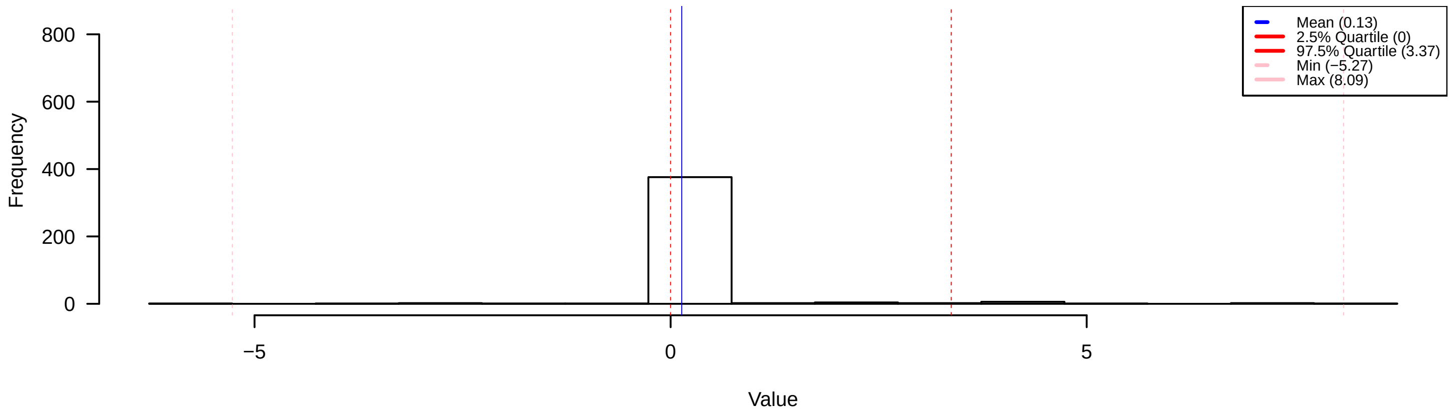

S5, Figure 168 : Bootstrap Distribution of Dewpoint Temperature:Relative Humidity lag 7

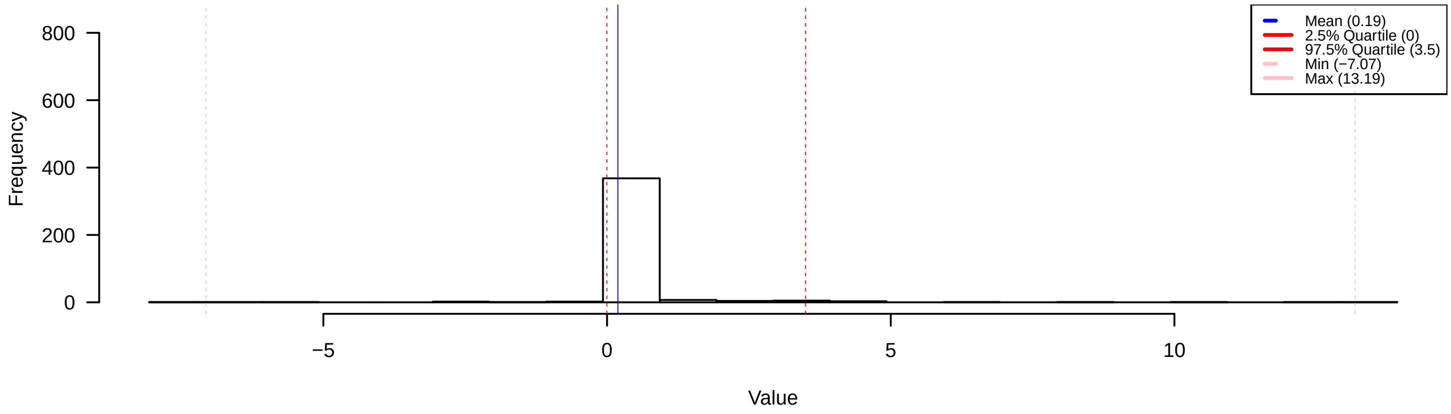

S5, Figure 169 : Bootstrap Distribution of Dewpoint Temperature:Relative Humidity lag 8

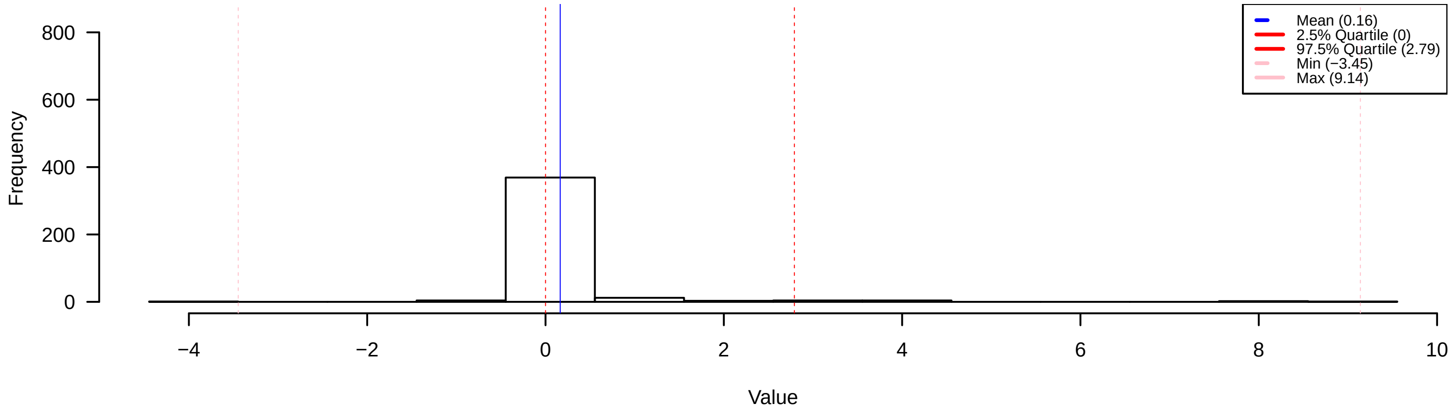

S5, Figure 170 : Bootstrap Distribution of Dewpoint Temperature:Relative Humidity lag 9

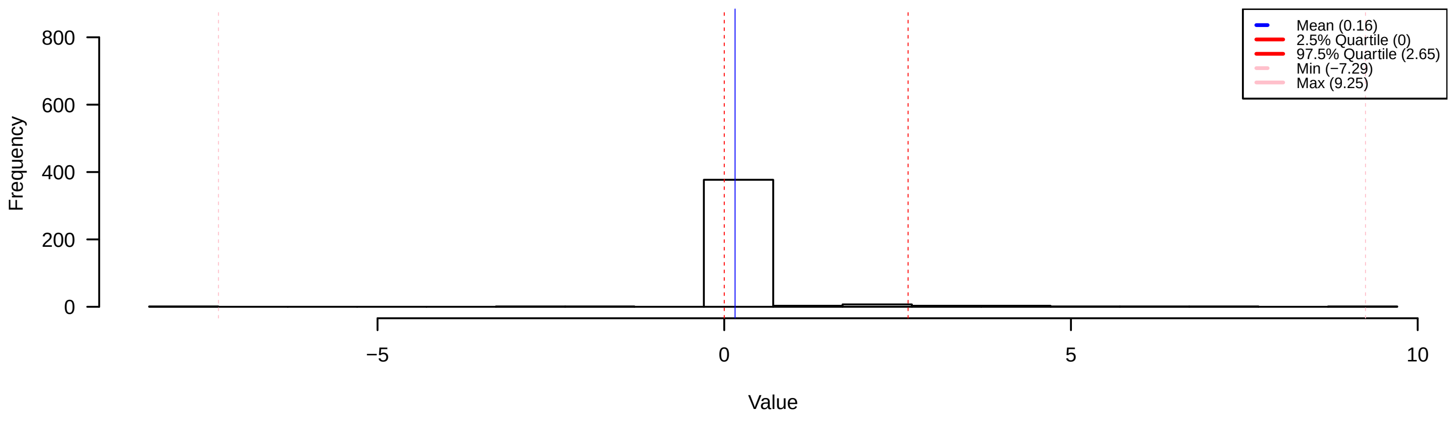

S5, Figure 171 : Bootstrap Distribution of Dewpoint Temperature:Relative Humidity lag 10

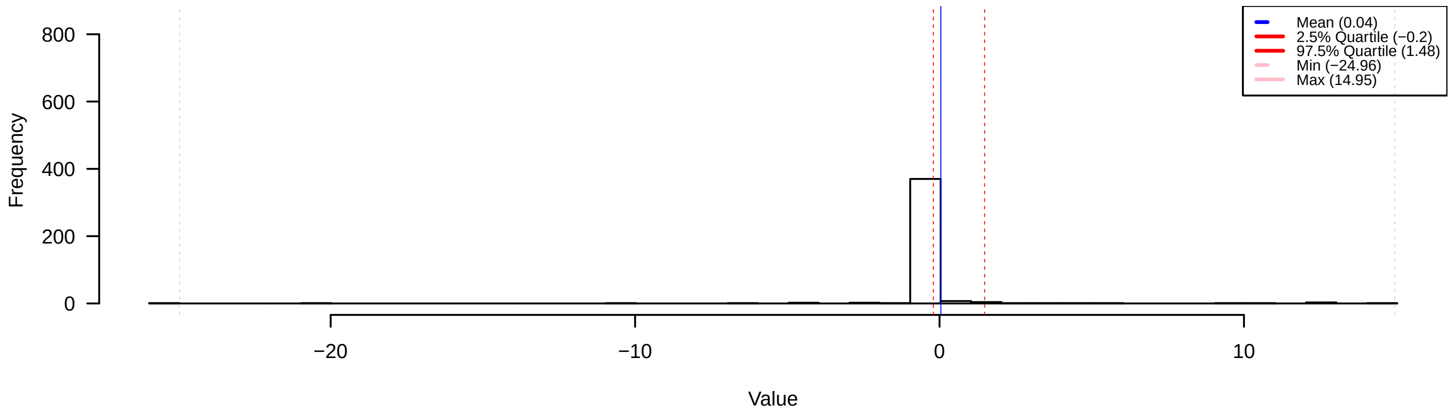

S5, Figure 172 : Bootstrap Distribution of Dewpoint Temperature:Relative Humidity lag 11

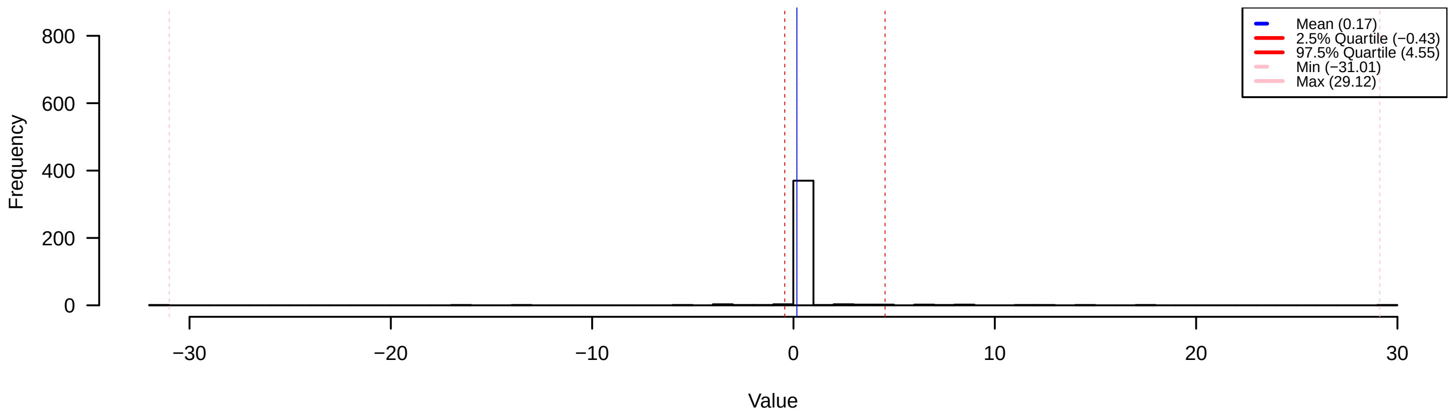

S5, Figure 173 : Bootstrap Distribution of Dewpoint Temperature:Relative Humidity lag 12

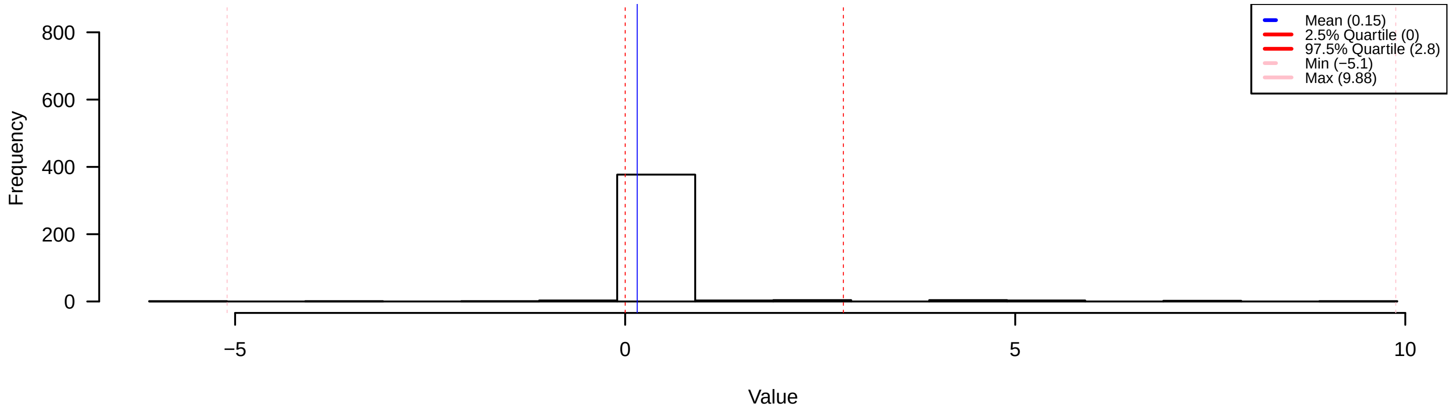

S5, Figure 174 : Bootstrap Distribution of Dewpoint Temperature:Relative Humidity lag 13

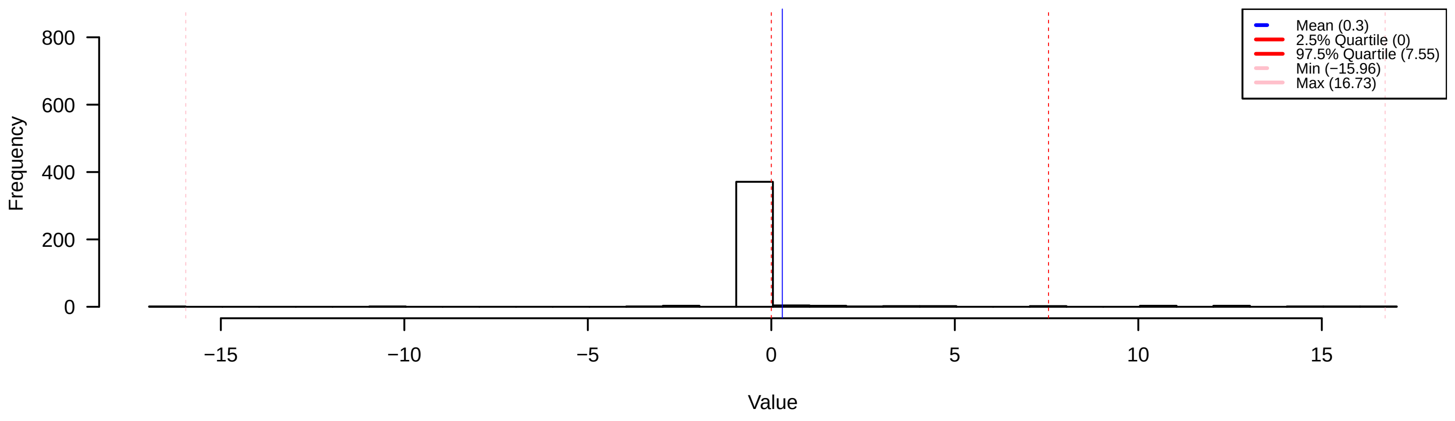

S5, Figure 175 : Bootstrap Distribution of Dewpoint Temperature:Relative Humidity lag 14

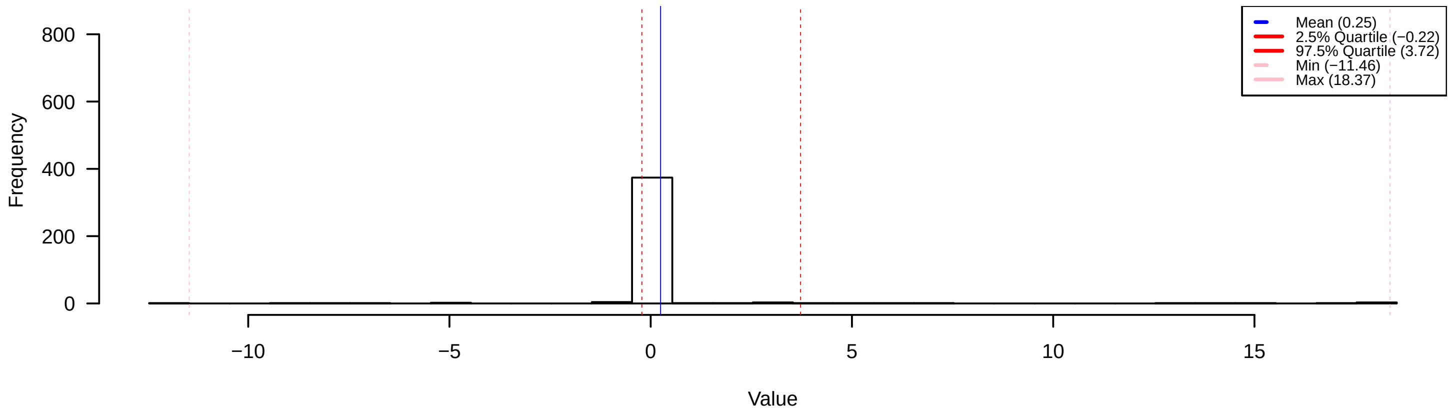

S5, Figure 176 : Bootstrap Distribution of Dewpoint Temperature:Relative Humidity lag 15

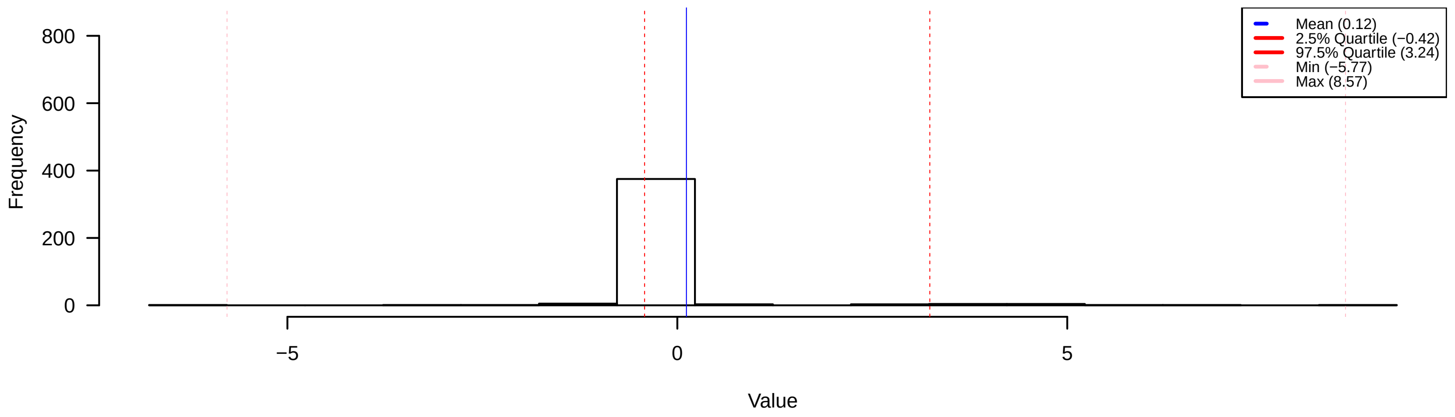

S5, Figure 177 : Bootstrap Distribution of Dewpoint Temperature:Relative Humidity lag 16

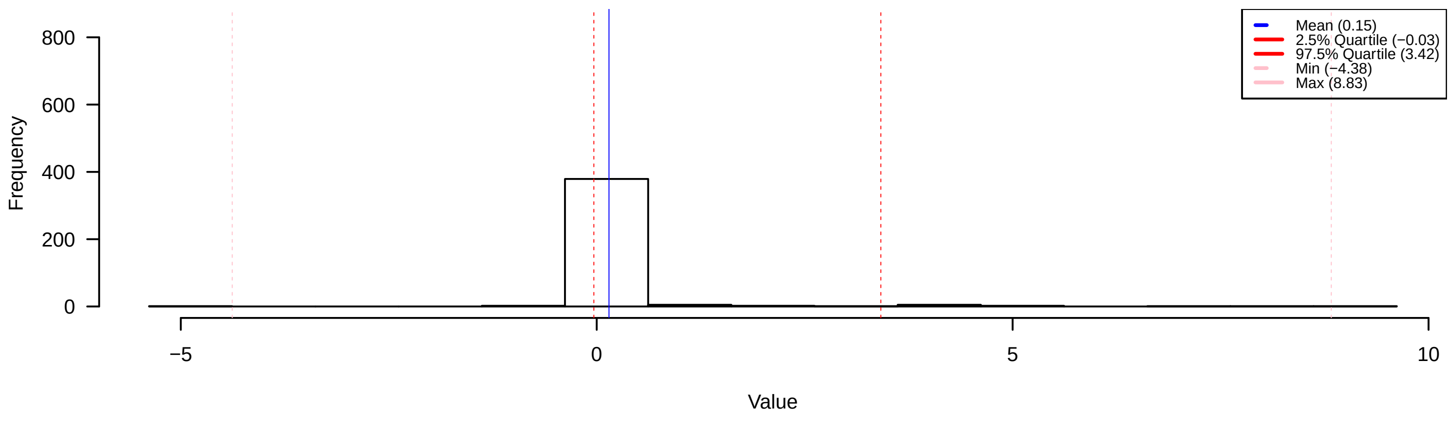

S5, Figure 178 : Bootstrap Distribution of Dewpoint Temperature:Relative Humidity lag 17

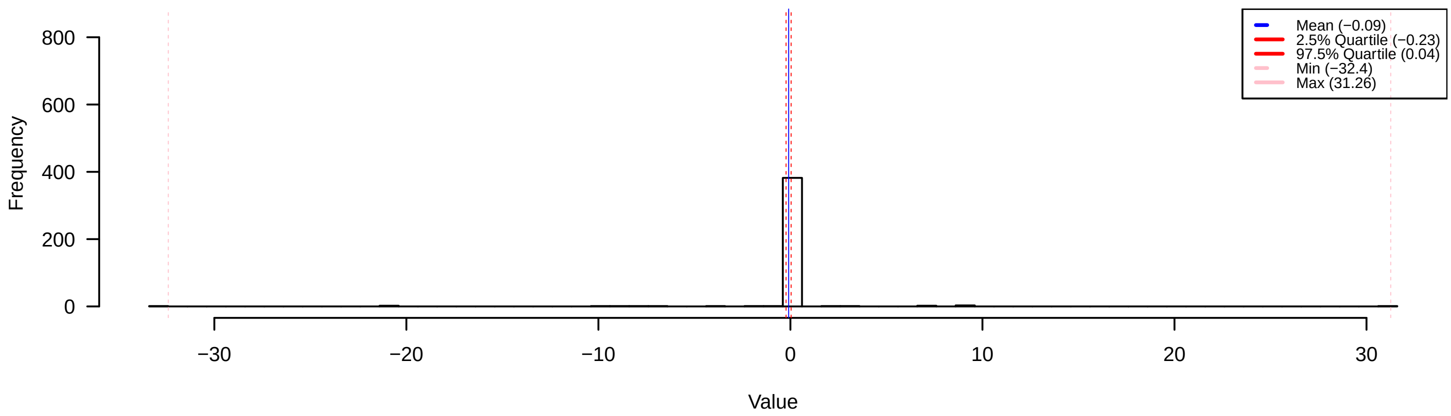

S5, Figure 179 : Bootstrap Distribution of Dewpoint Temperature:Relative Humidity lag 18

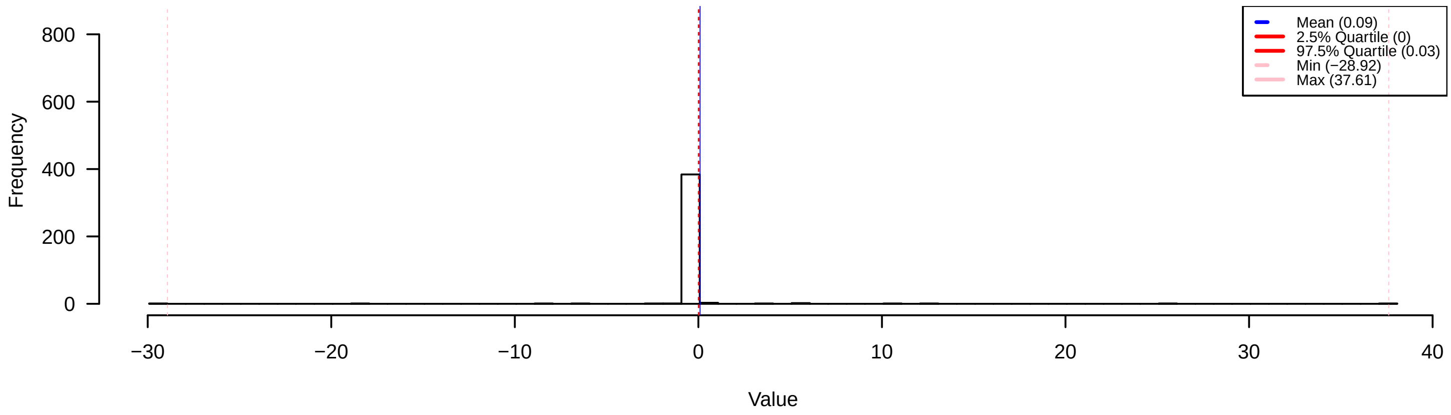

S5, Figure 180 : Bootstrap Distribution of Dewpoint Temperature:Relative Humidity lag 19

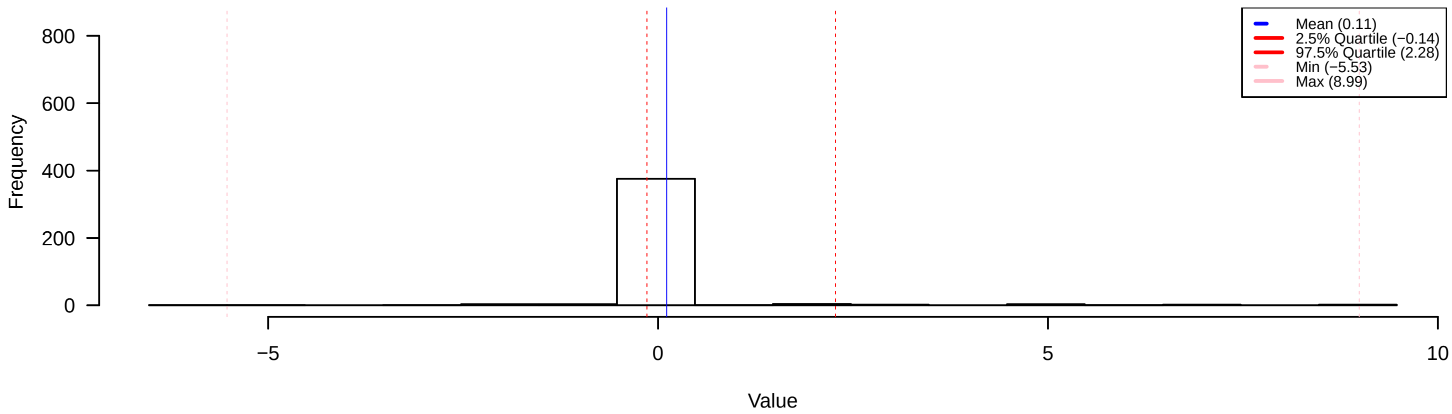

S5, Figure 181 : Bootstrap Distribution of Dewpoint Temperature:Relative Humidity lag 20

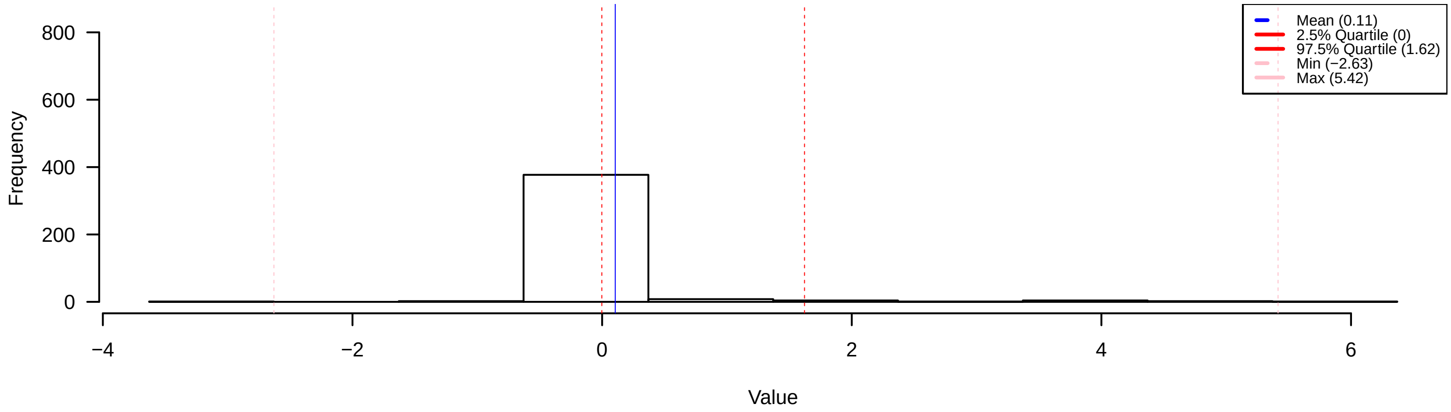

S5, Figure 182 : Bootstrap Distribution of Air Temperature:Absolute Humidity lag 1

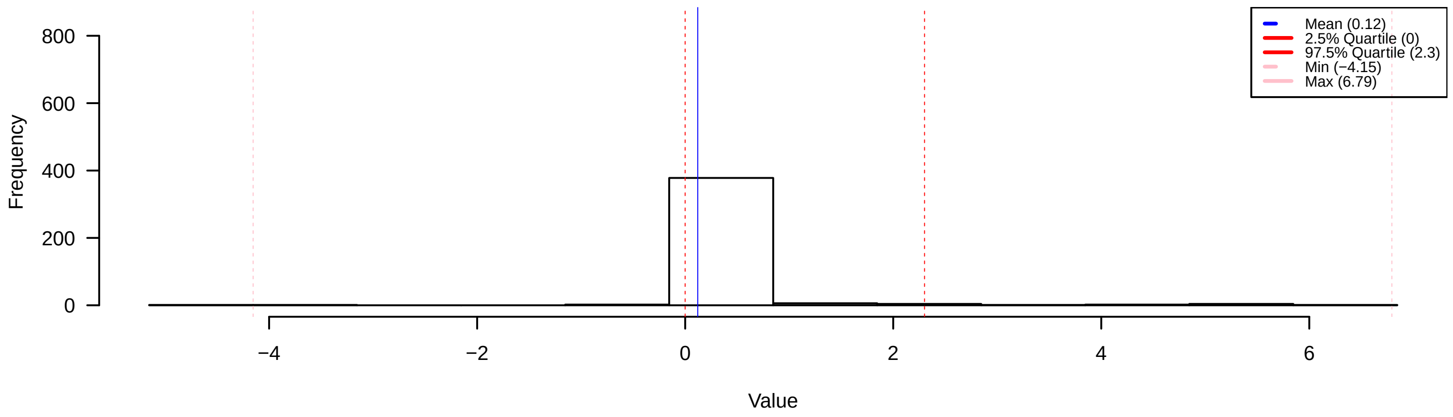

S5, Figure 183 : Bootstrap Distribution of Air Temperature:Absolute Humidity lag 2

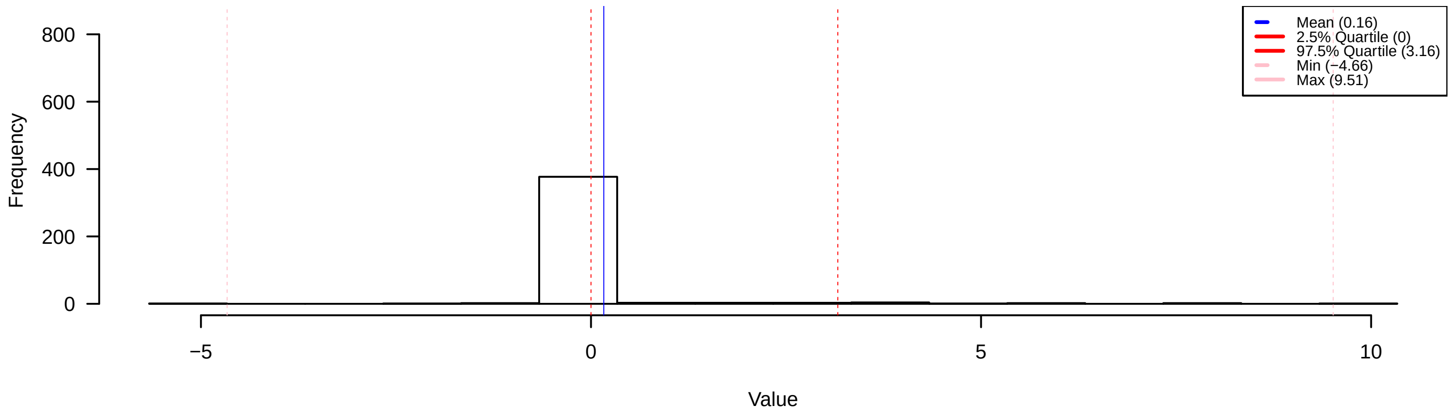

S5, Figure 184 : Bootstrap Distribution of Air Temperature:Absolute Humidity lag 3

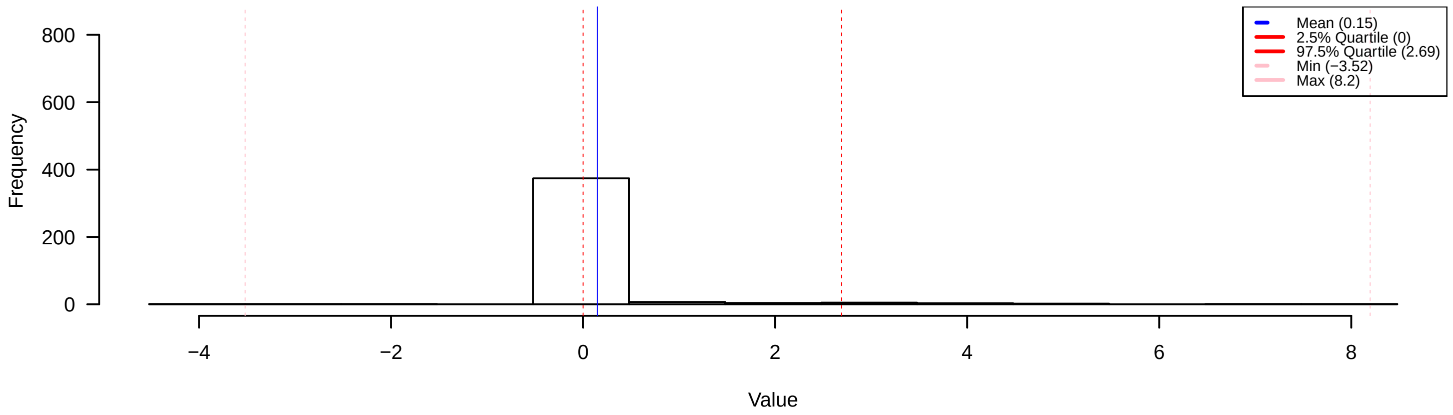

S5, Figure 185 : Bootstrap Distribution of Air Temperature:Absolute Humidity lag 4

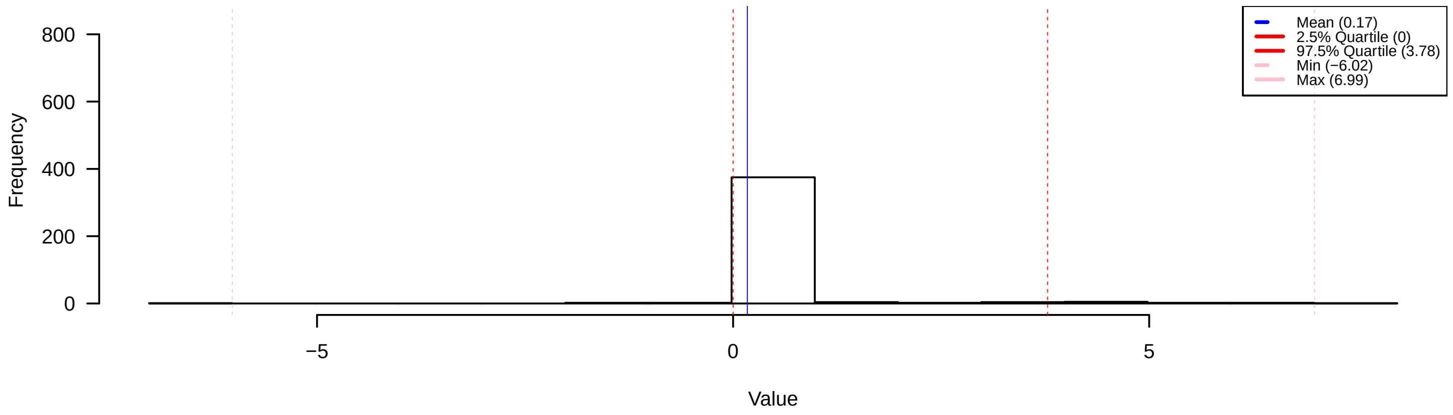

S5, Figure 186 : Bootstrap Distribution of Air Temperature:Absolute Humidity lag 5

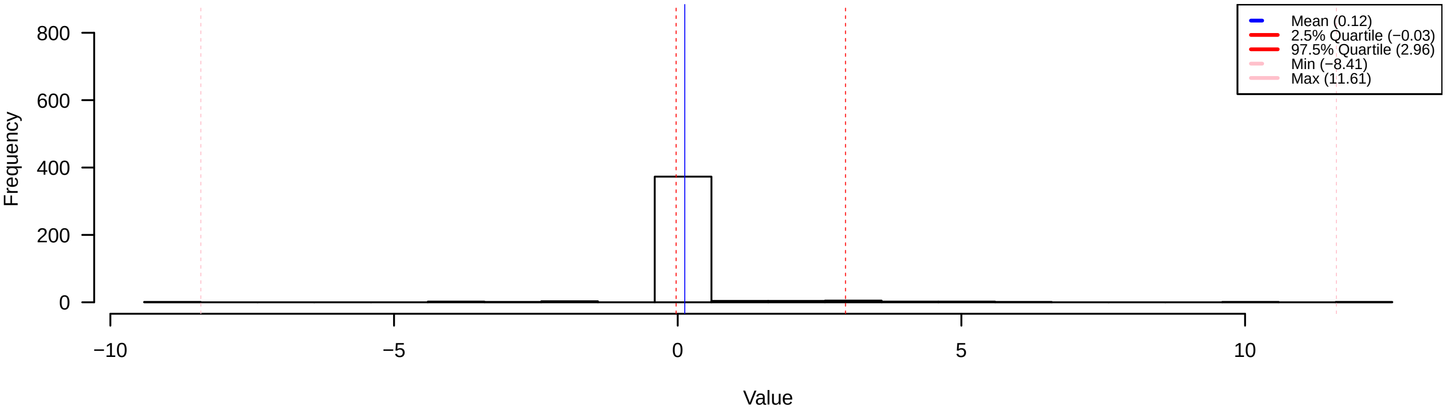

S5, Figure 187 : Bootstrap Distribution of Air Temperature:Absolute Humidity lag 6

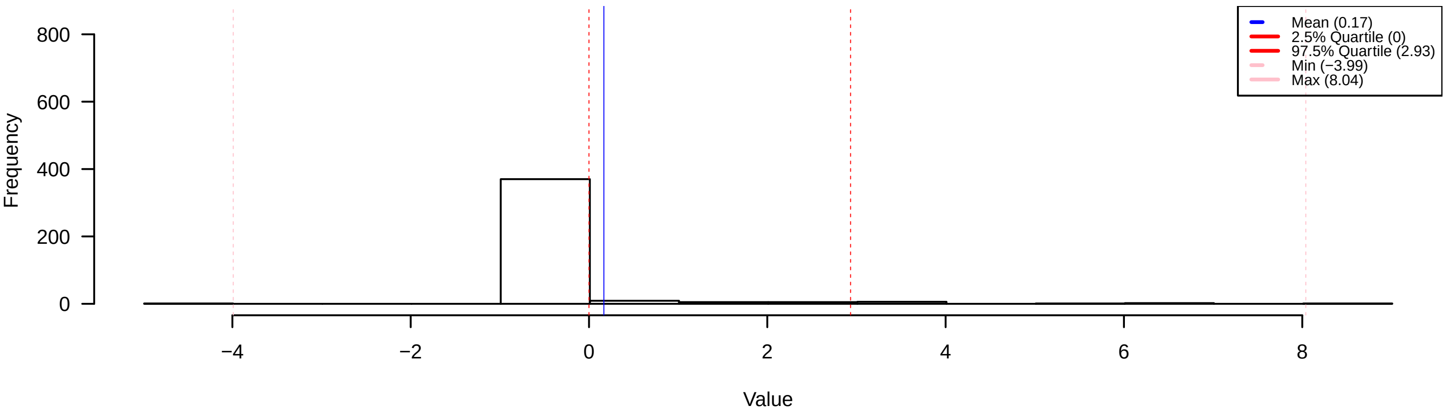

S5, Figure 188 : Bootstrap Distribution of Air Temperature:Absolute Humidity lag 7

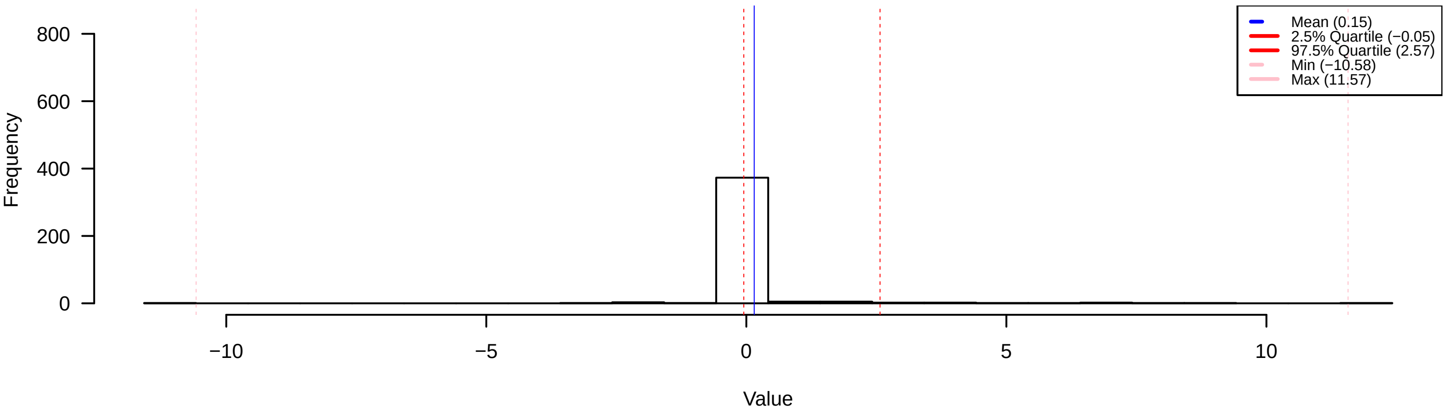

S5, Figure 189 : Bootstrap Distribution of Air Temperature:Absolute Humidity lag 8

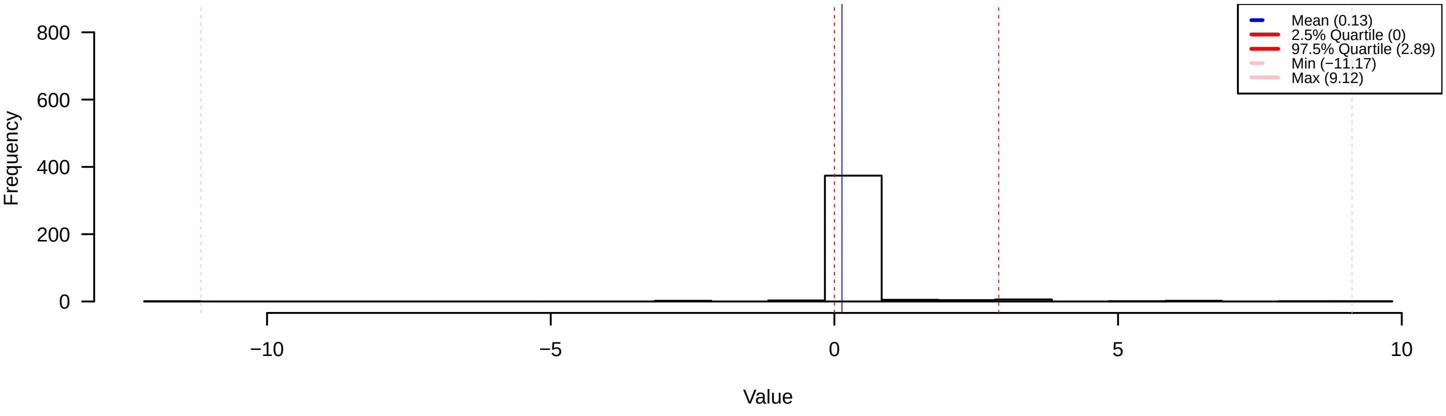

S5, Figure 190 : Bootstrap Distribution of Air Temperature:Absolute Humidity lag 9

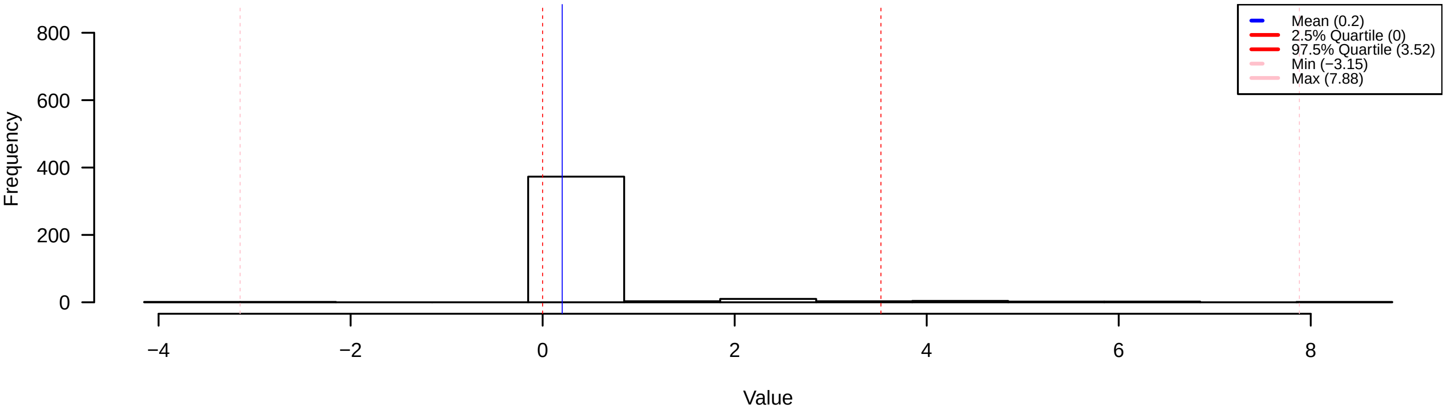

S5, Figure 191 : Bootstrap Distribution of Air Temperature:Absolute Humidity lag 10

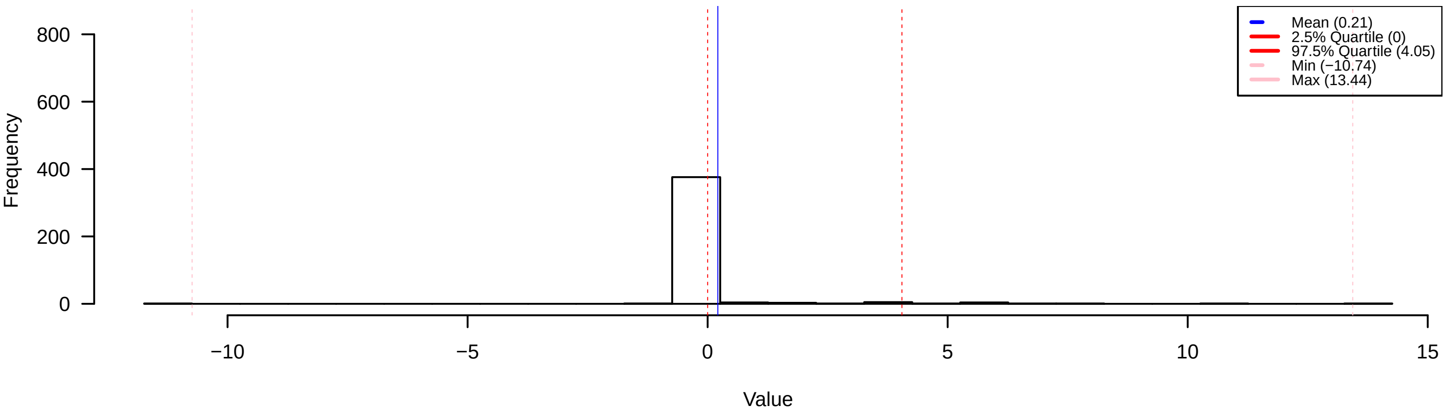

S5, Figure 192 : Bootstrap Distribution of Air Temperature:Absolute Humidity lag 11

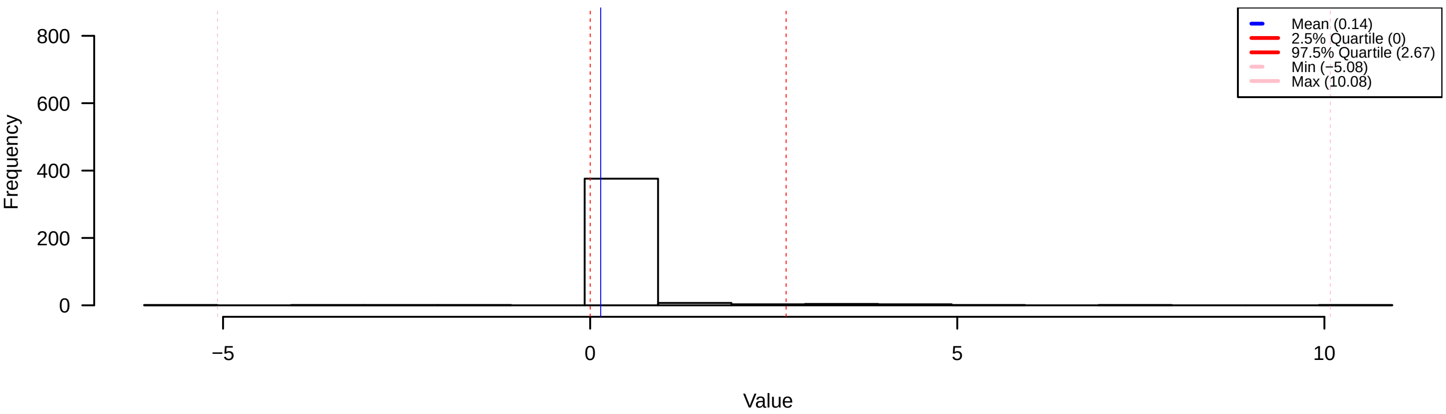

S5, Figure 193 : Bootstrap Distribution of Air Temperature:Absolute Humidity lag 12

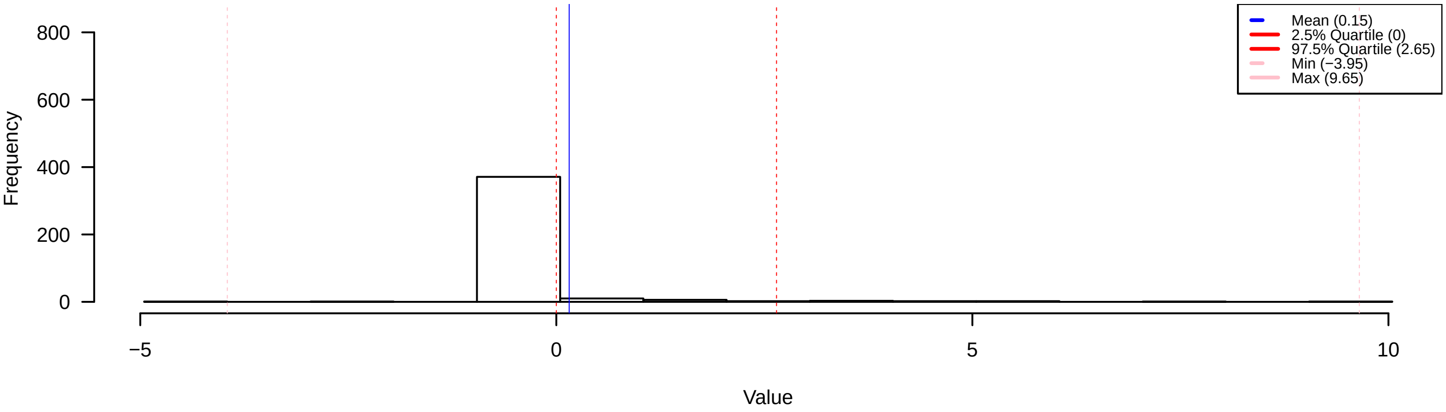

S5, Figure 194 : Bootstrap Distribution of Air Temperature:Absolute Humidity lag 13

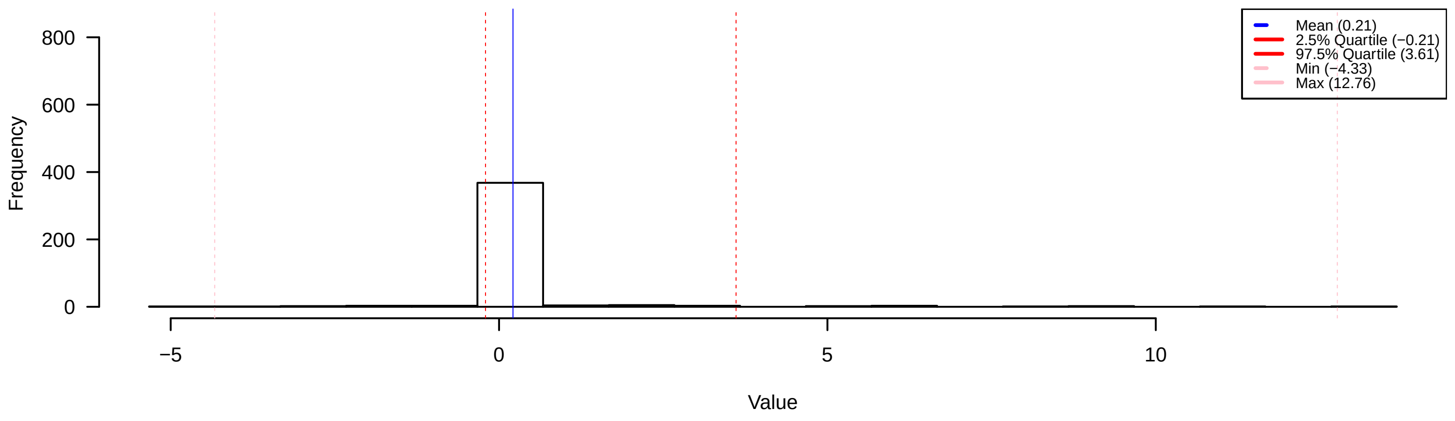

S5, Figure 195 : Bootstrap Distribution of Air Temperature:Absolute Humidity lag 14

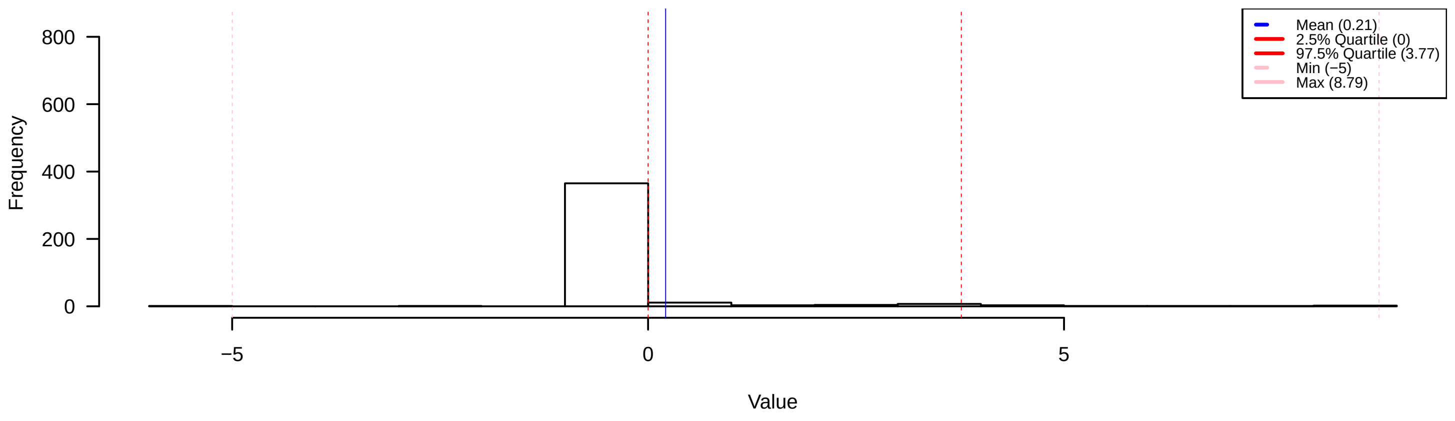

S5, Figure 196 : Bootstrap Distribution of Air Temperature:Absolute Humidity lag 15

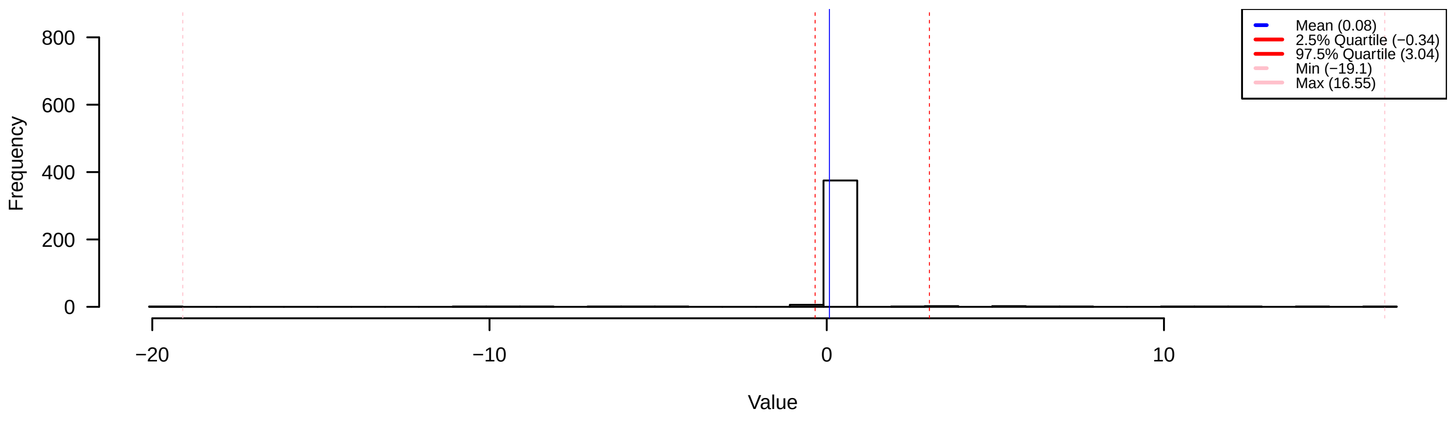

S5, Figure 197 : Bootstrap Distribution of Air Temperature:Absolute Humidity lag 16

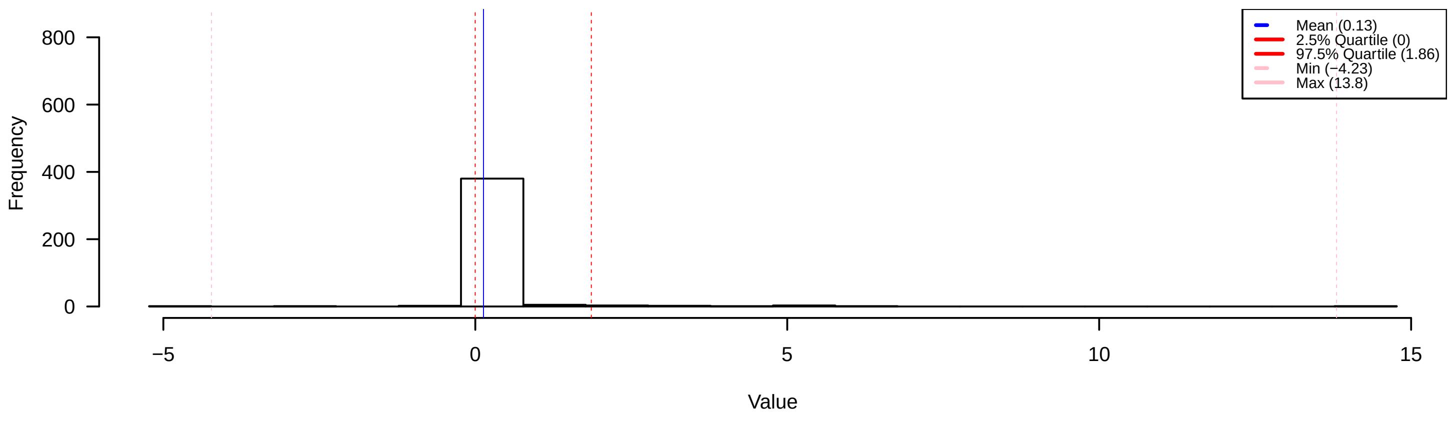

S5, Figure 198 : Bootstrap Distribution of Air Temperature:Absolute Humidity lag 17

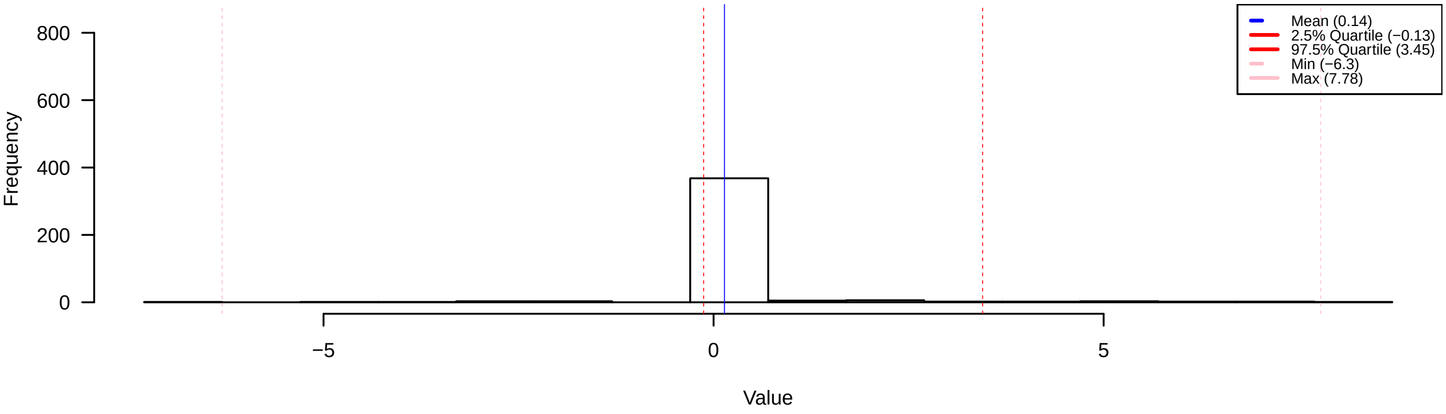

S5, Figure 199 : Bootstrap Distribution of Air Temperature:Absolute Humidity lag 18

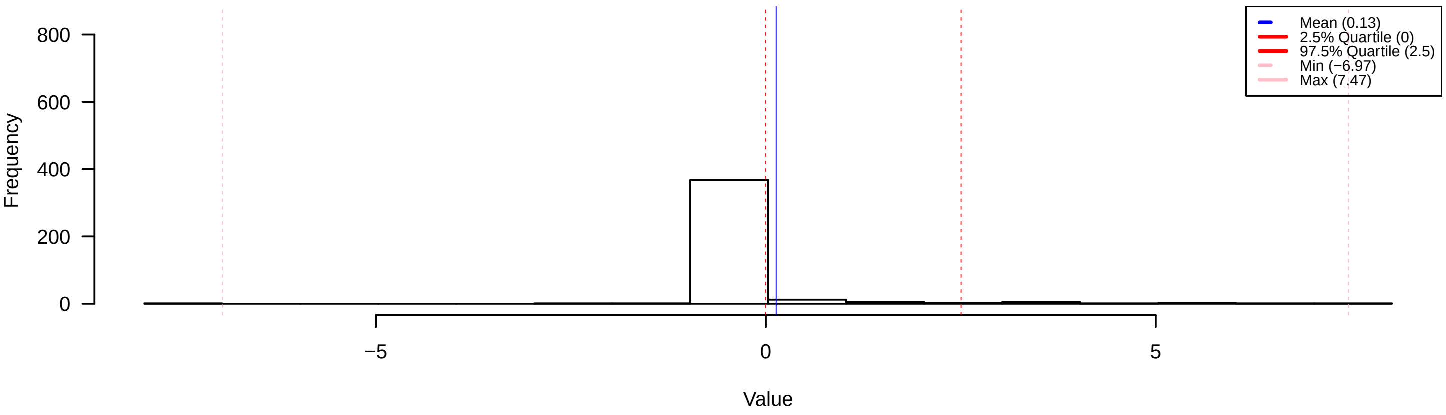

S5, Figure 200 : Bootstrap Distribution of Air Temperature:Absolute Humidity lag 19

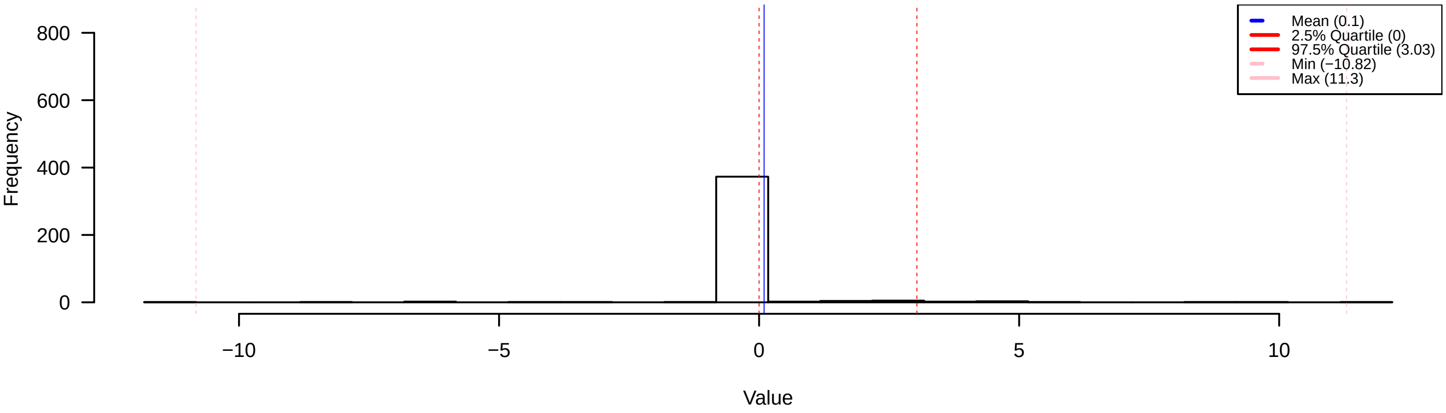

S5, Figure 201 : Bootstrap Distribution of Air Temperature:Absolute Humidity lag 20

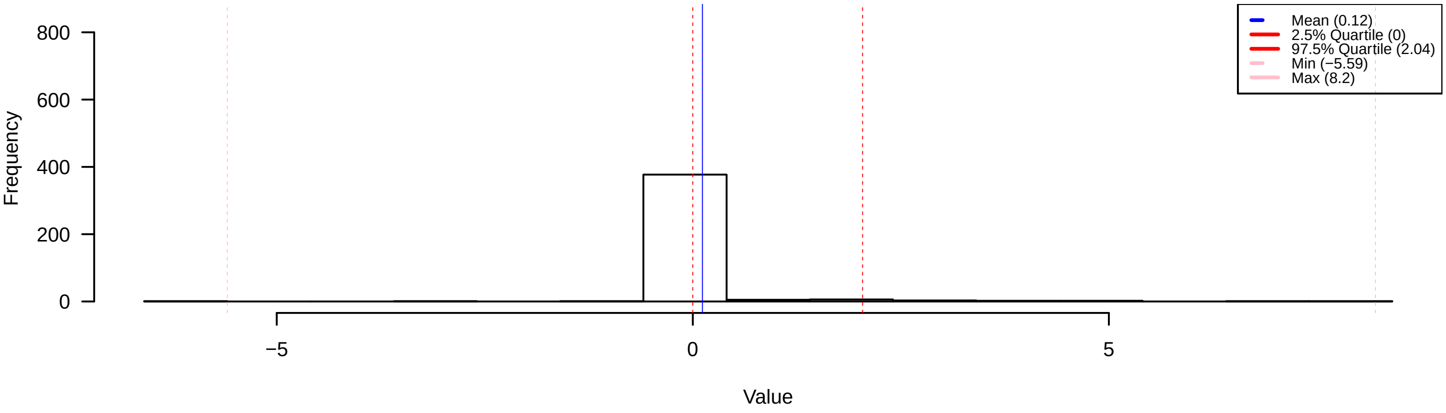

S5, Figure 202 : Bootstrap Distribution of Air Temperature:Relative Humidity lag 1

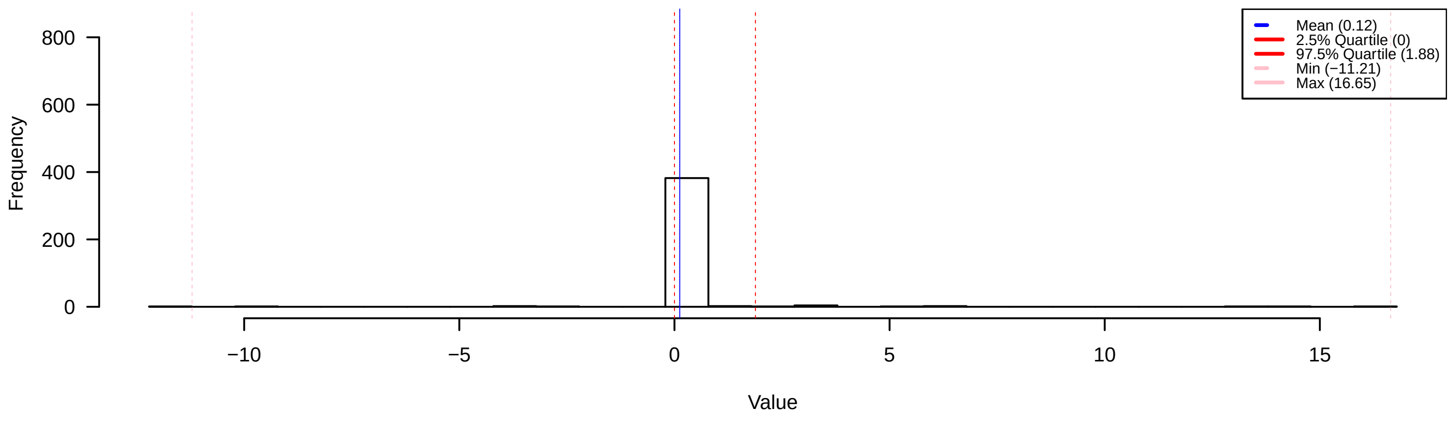

S5, Figure 203 : Bootstrap Distribution of Air Temperature:Relative Humidity lag 2

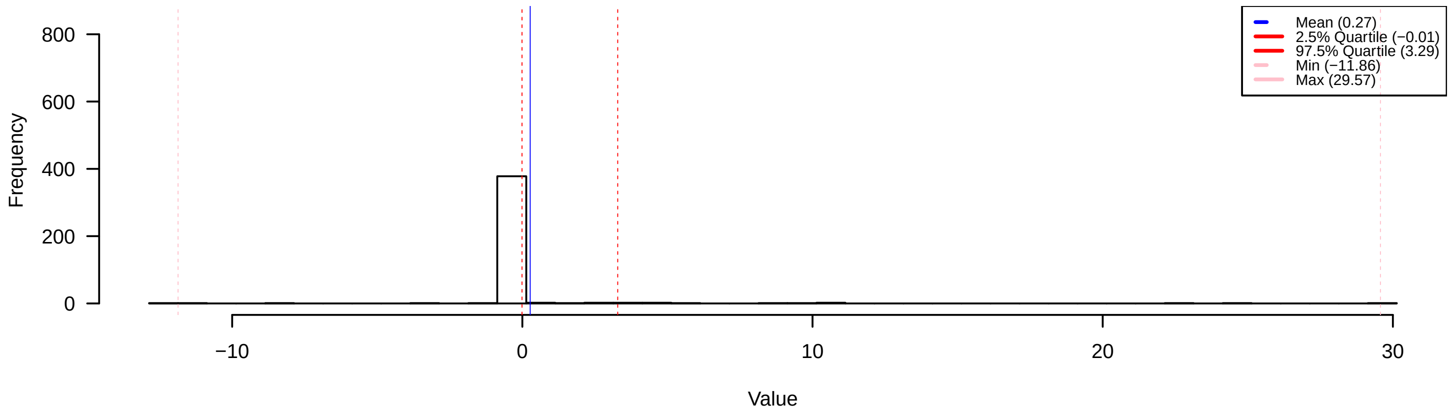

S5, Figure 204 : Bootstrap Distribution of Air Temperature:Relative Humidity lag 3

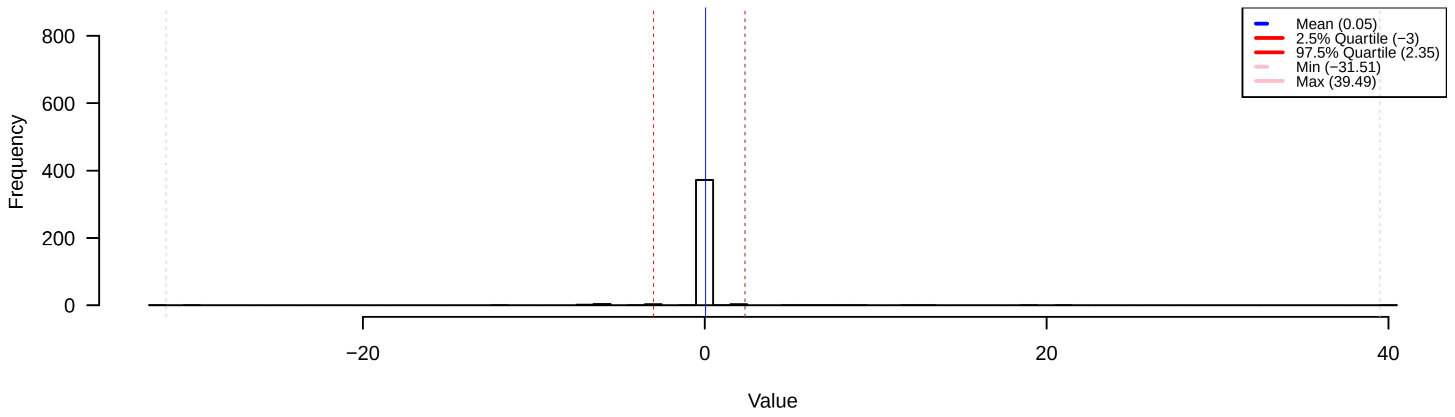

S5, Figure 205 : Bootstrap Distribution of Air Temperature:Relative Humidity lag 4

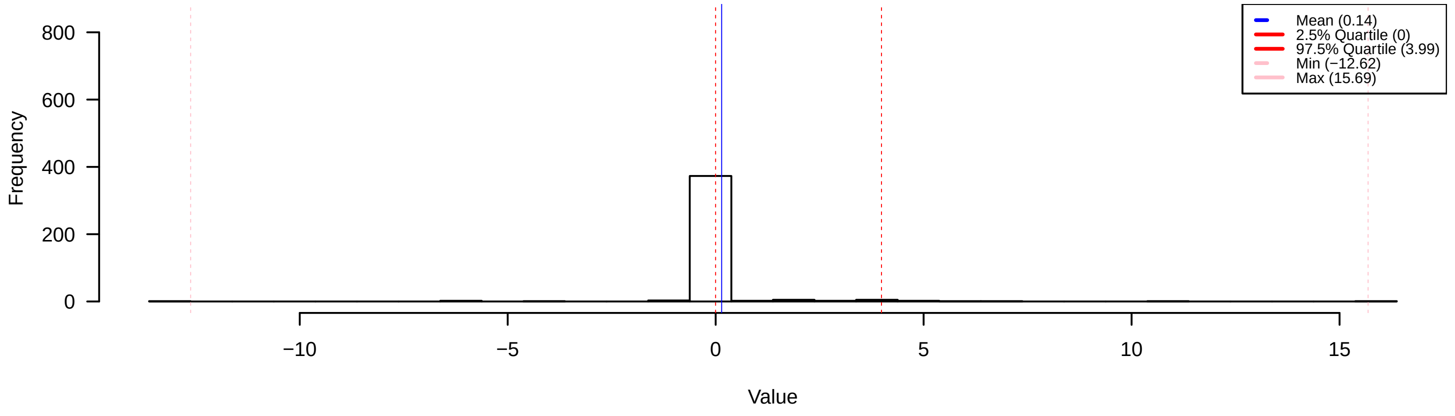

S5, Figure 206 : Bootstrap Distribution of Air Temperature:Relative Humidity lag 5

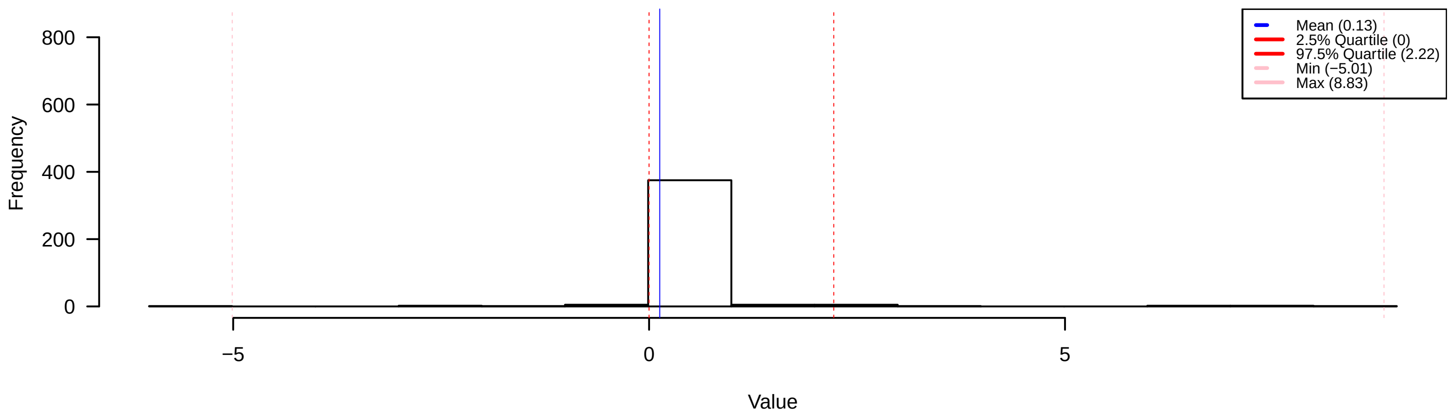

S5, Figure 207 : Bootstrap Distribution of Air Temperature:Relative Humidity lag 6

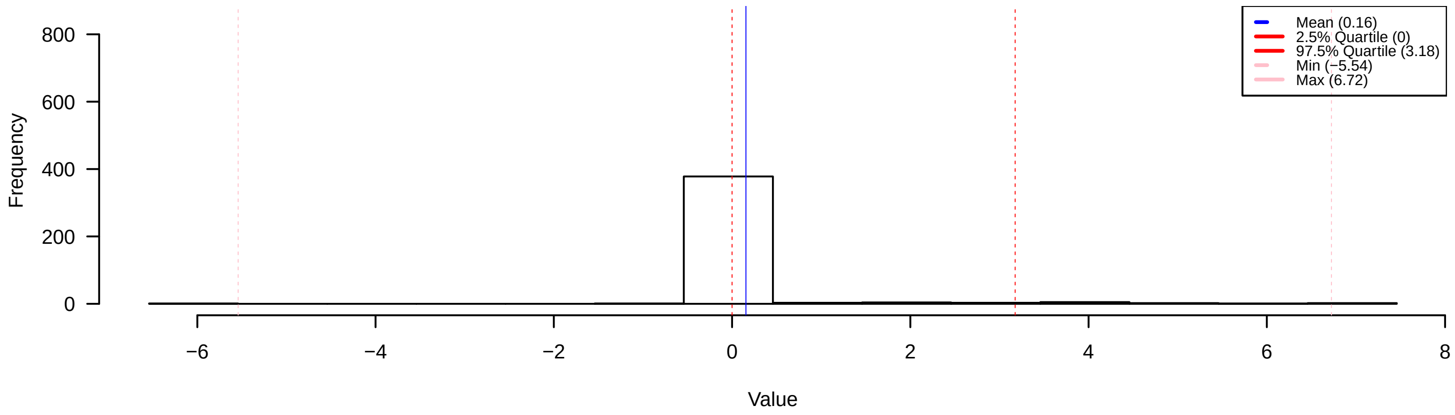

S5, Figure 208 : Bootstrap Distribution of Air Temperature:Relative Humidity lag 7

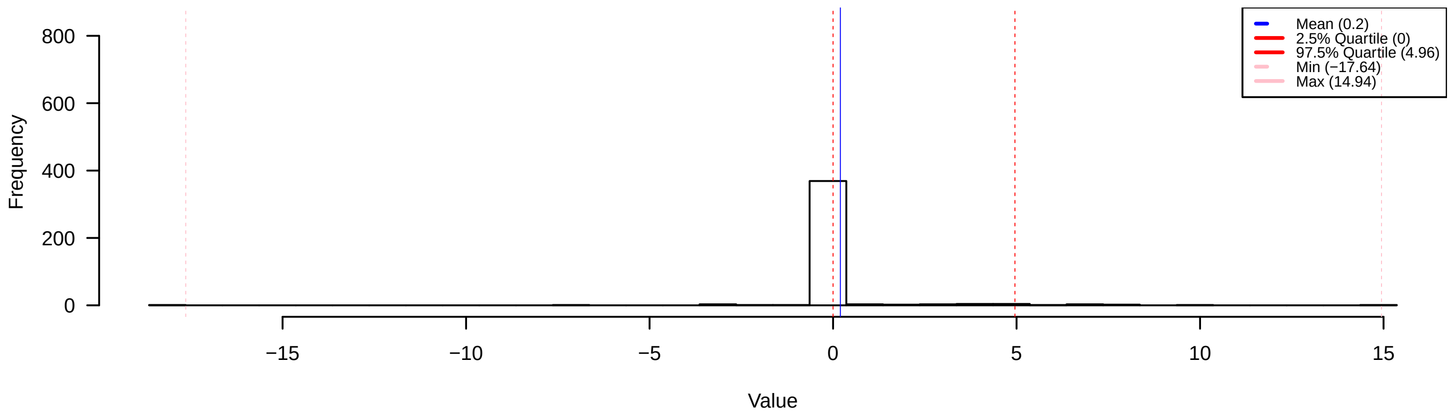

S5, Figure 209 : Bootstrap Distribution of Air Temperature:Relative Humidity lag 8

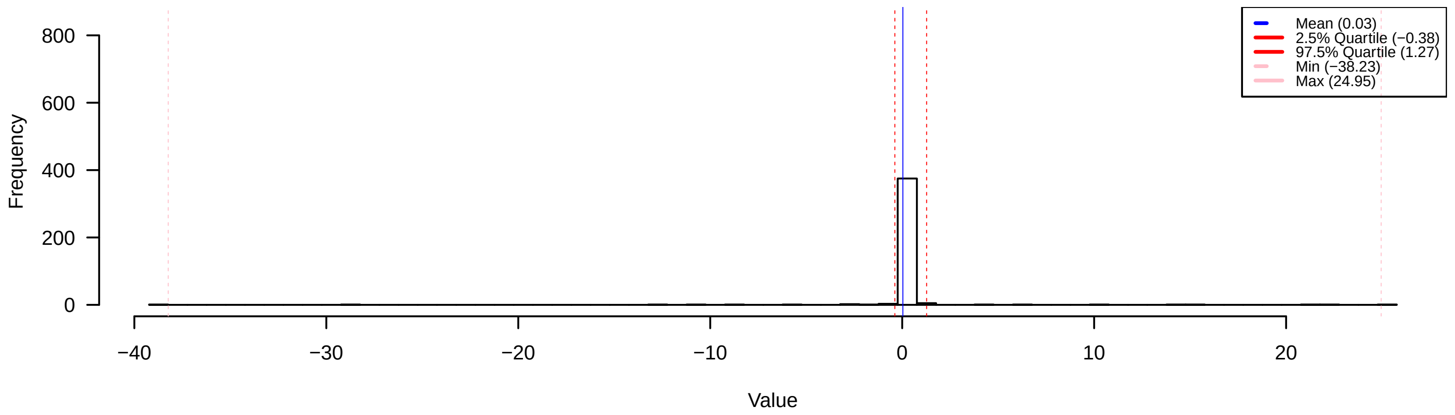

S5, Figure 210 : Bootstrap Distribution of Air Temperature:Relative Humidity lag 9

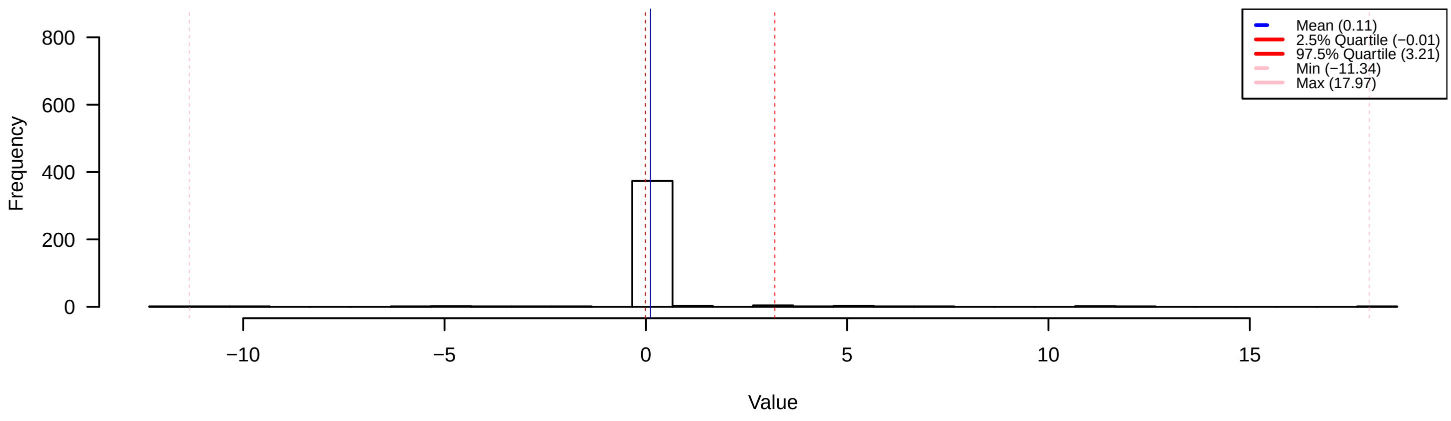

S5, Figure 211 : Bootstrap Distribution of Air Temperature:Relative Humidity lag 10

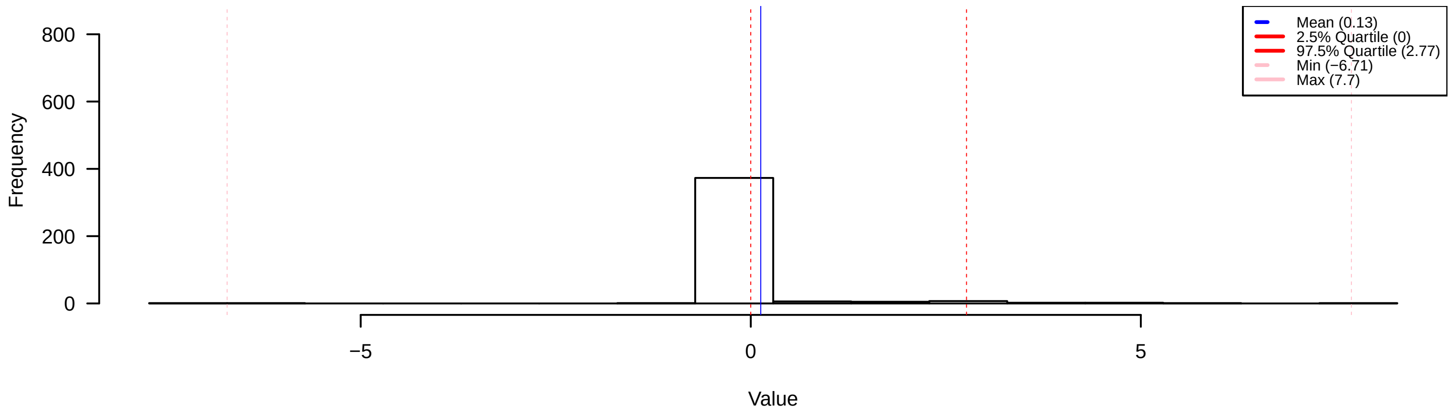

S5, Figure 212 : Bootstrap Distribution of Air Temperature:Relative Humidity lag 11

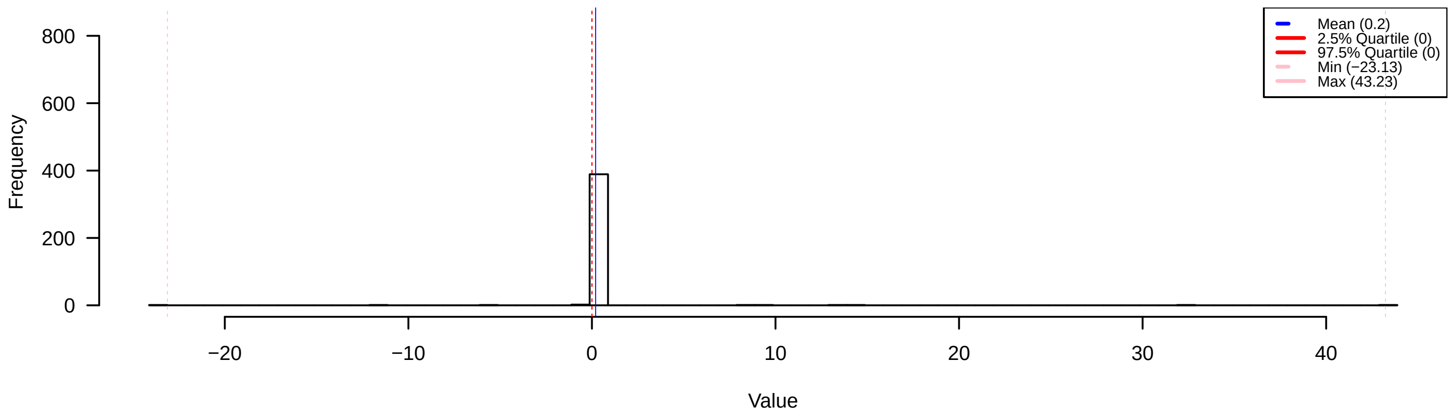

S5, Figure 213 : Bootstrap Distribution of Air Temperature:Relative Humidity lag 12

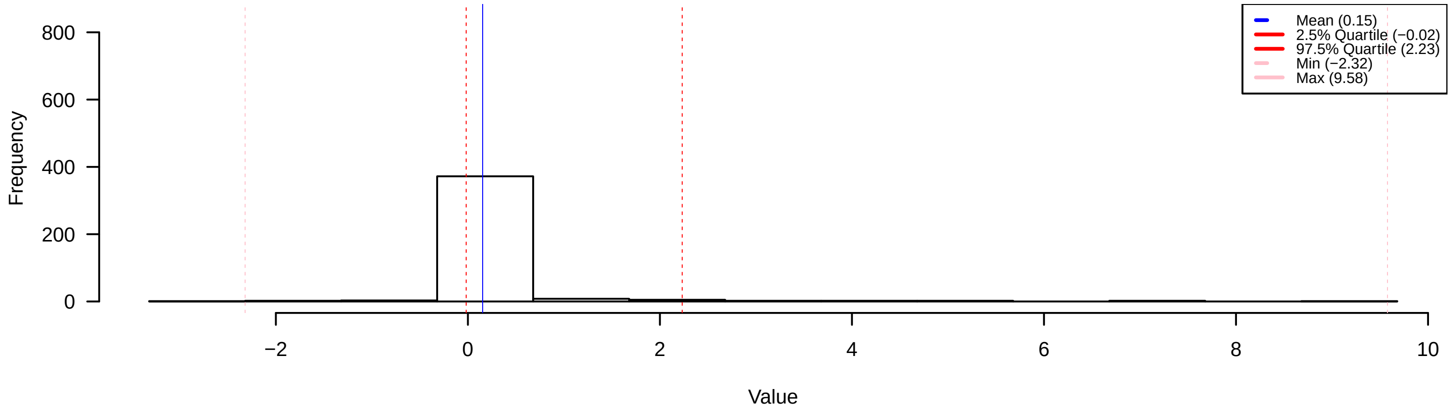

S5, Figure 214 : Bootstrap Distribution of Air Temperature:Relative Humidity lag 13

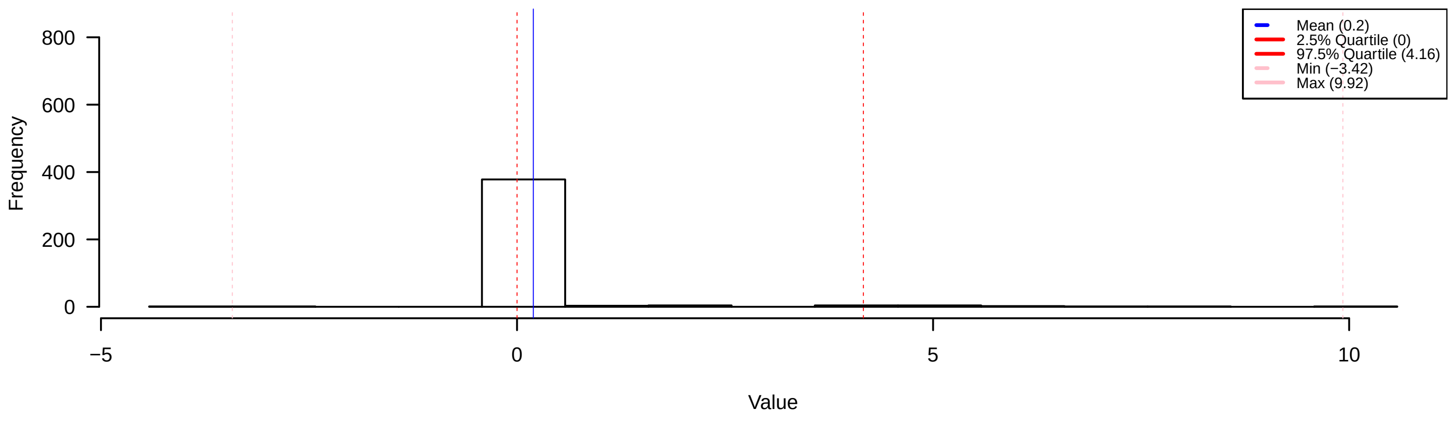

S5, Figure 215 : Bootstrap Distribution of Air Temperature:Relative Humidity lag 14

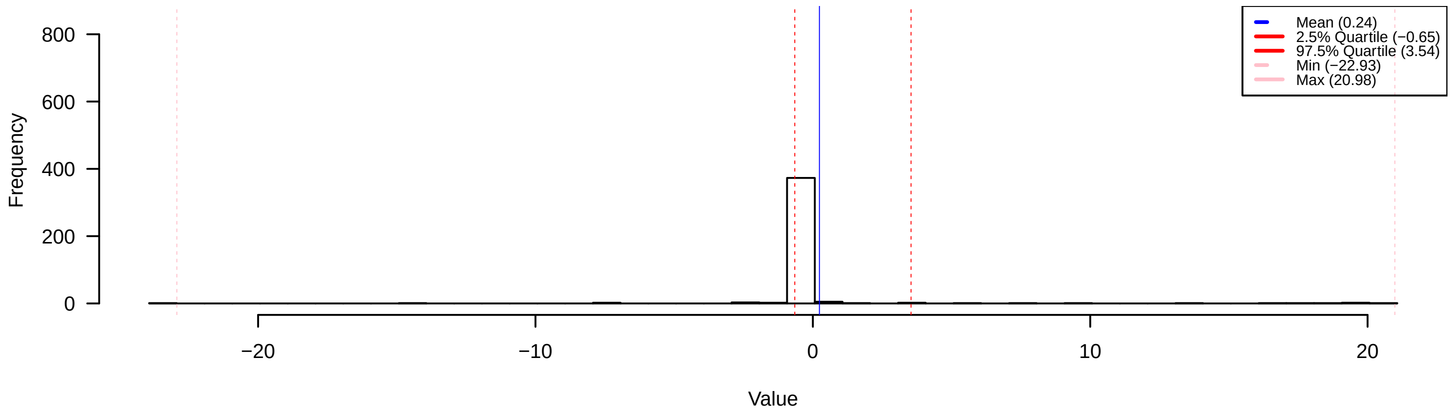

S5, Figure 216 : Bootstrap Distribution of Air Temperature:Relative Humidity lag 15

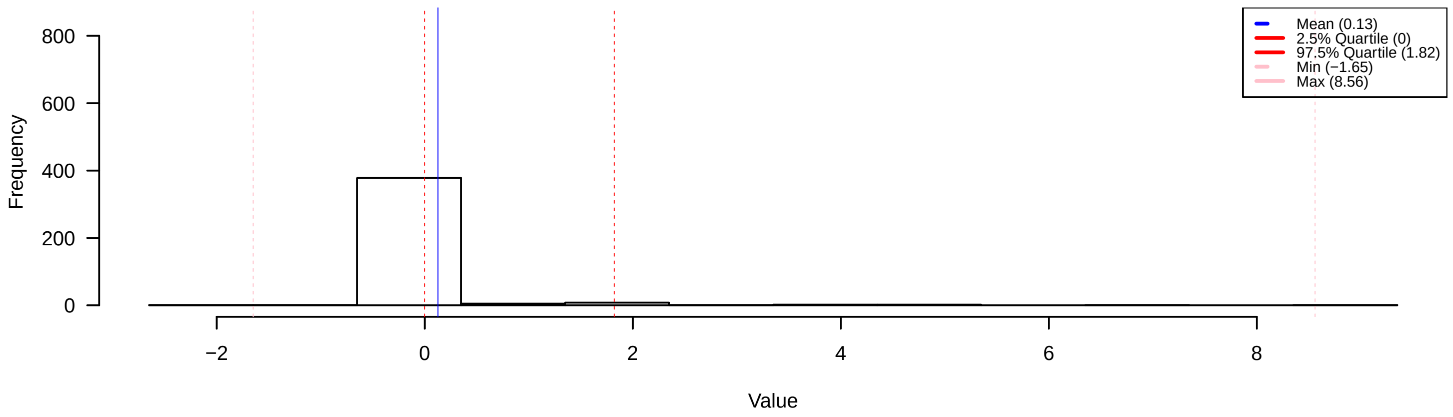

S5, Figure 217 : Bootstrap Distribution of Air Temperature:Relative Humidity lag 16

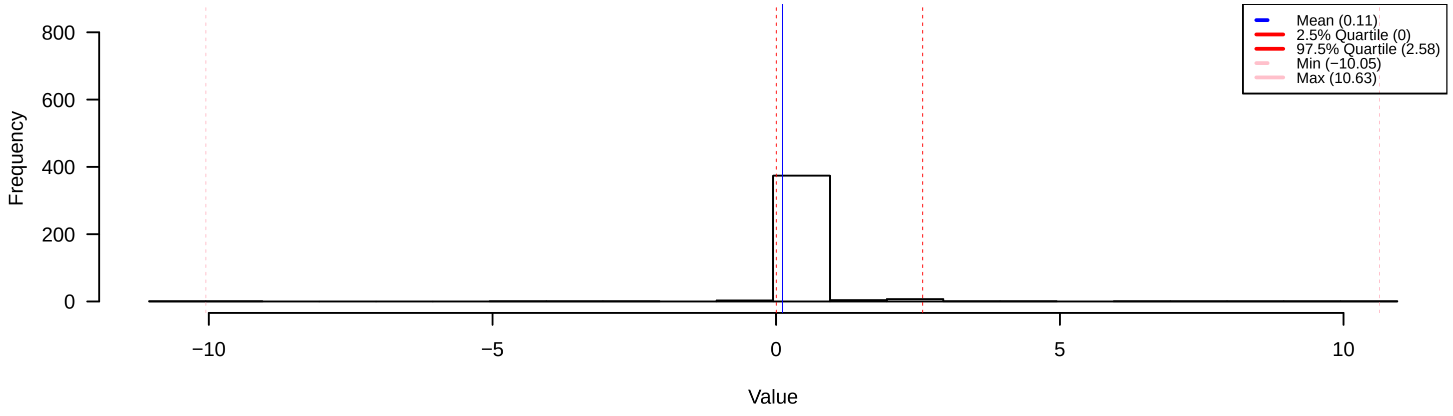

S5, Figure 218 : Bootstrap Distribution of Air Temperature:Relative Humidity lag 17

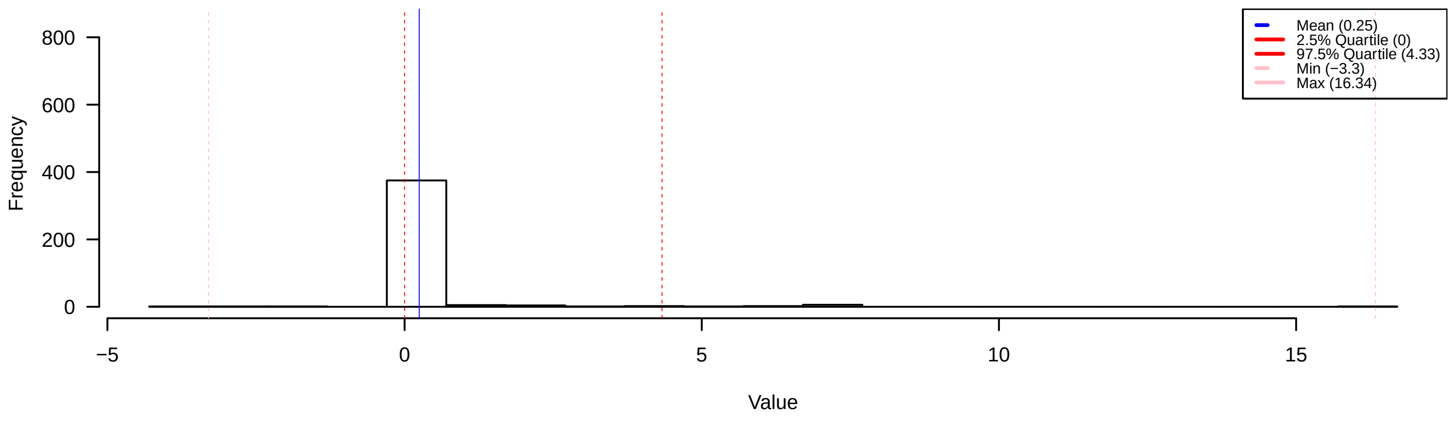

S5, Figure 219 : Bootstrap Distribution of Air Temperature:Relative Humidity lag 18

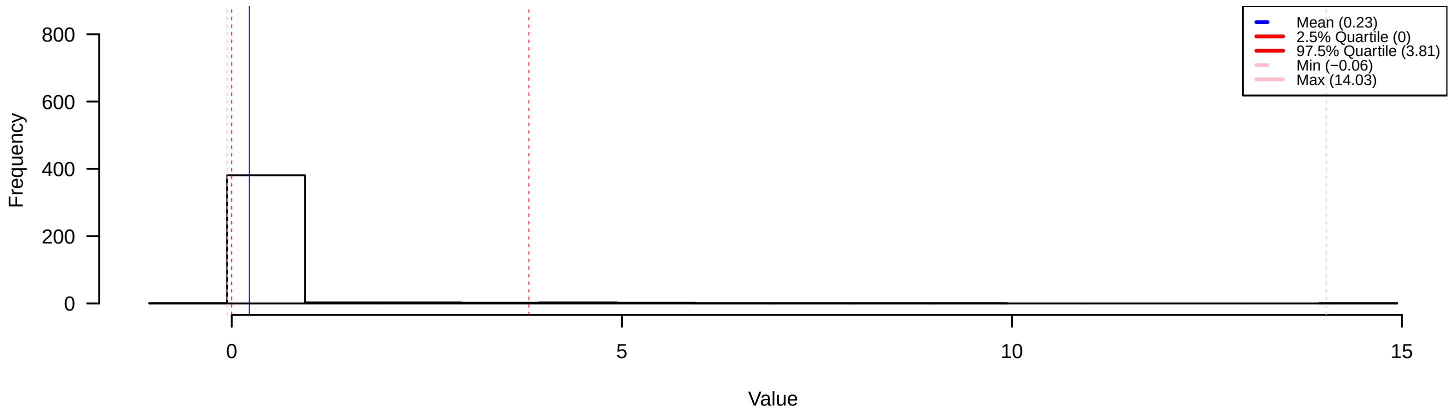

S5, Figure 220 : Bootstrap Distribution of Air Temperature:Relative Humidity lag 19

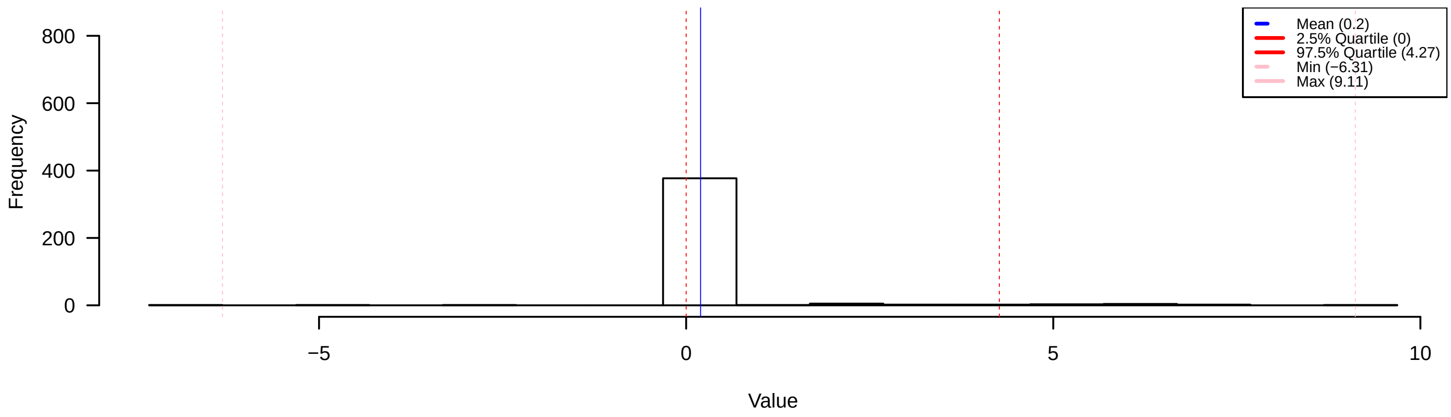

S5, Figure 221 : Bootstrap Distribution of Air Temperature:Relative Humidity lag 20

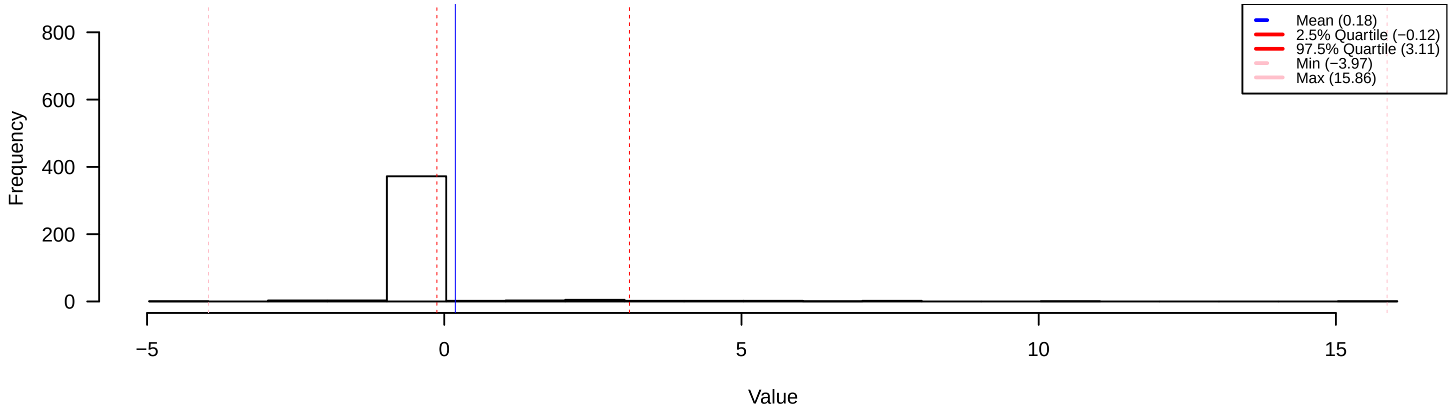

S5, Figure 222 : Bootstrap Distribution of Absolute Humidity:Relative Humidity lag 1

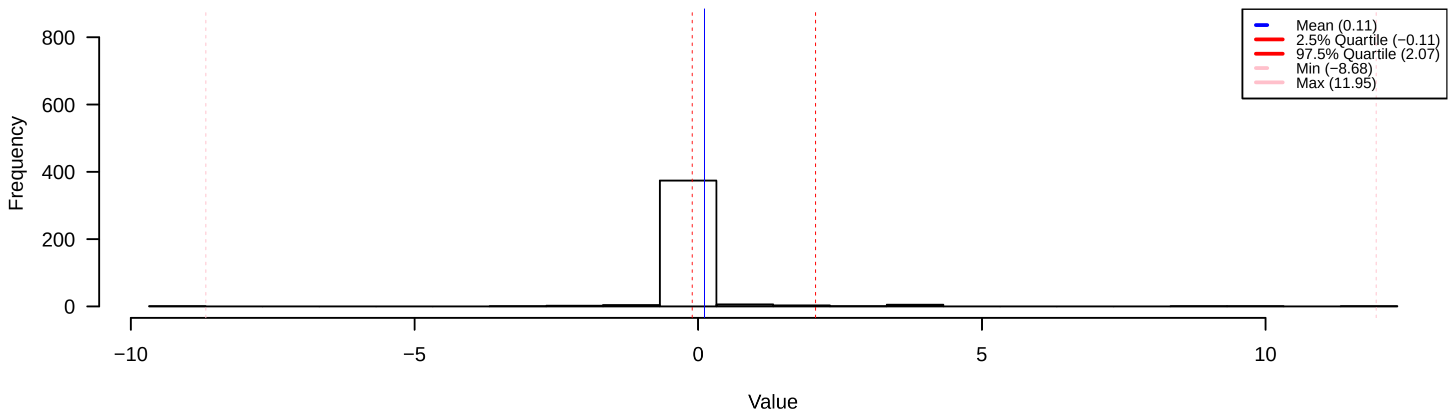

S5, Figure 223 : Bootstrap Distribution of Absolute Humidity:Relative Humidity lag 2

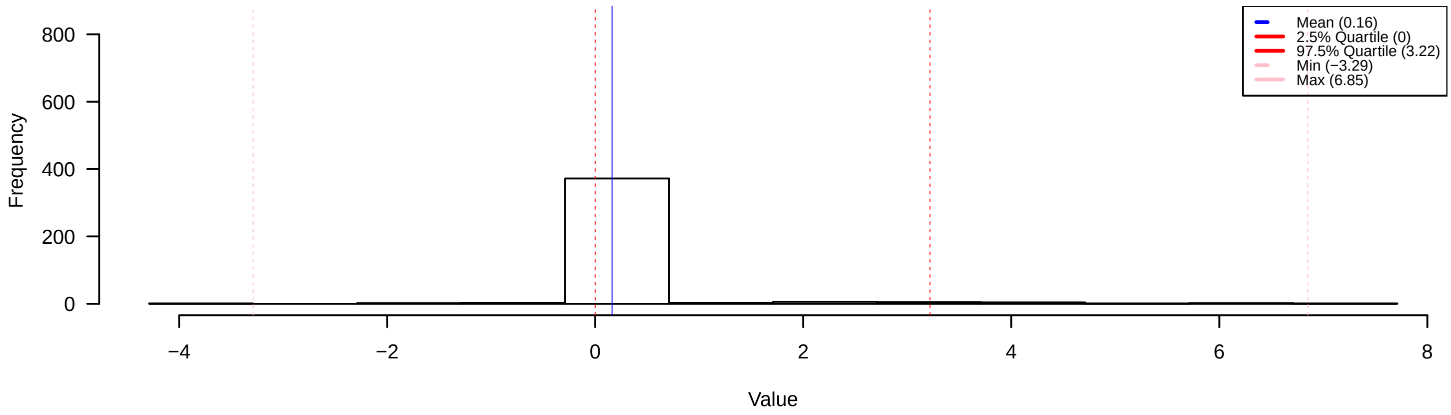

S5, Figure 224 : Bootstrap Distribution of Absolute Humidity:Relative Humidity lag 3

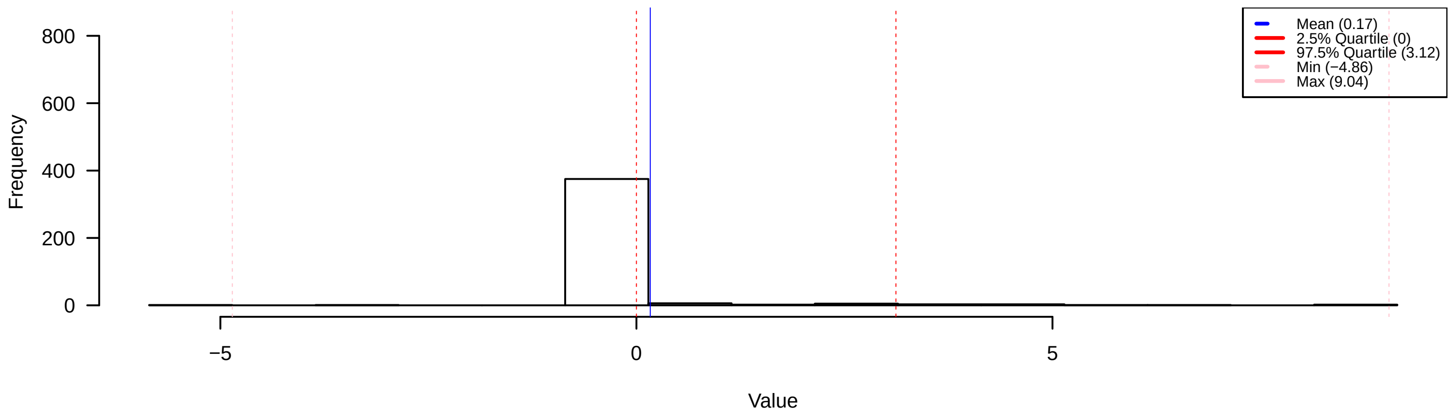

S5, Figure 225 : Bootstrap Distribution of Absolute Humidity:Relative Humidity lag 4

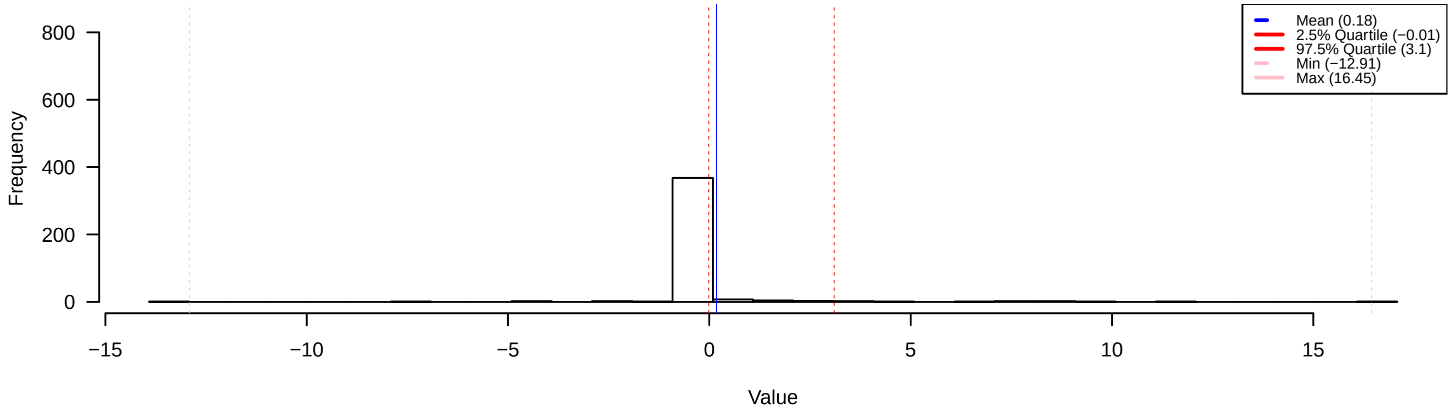

S5, Figure 226 : Bootstrap Distribution of Absolute Humidity:Relative Humidity lag 5

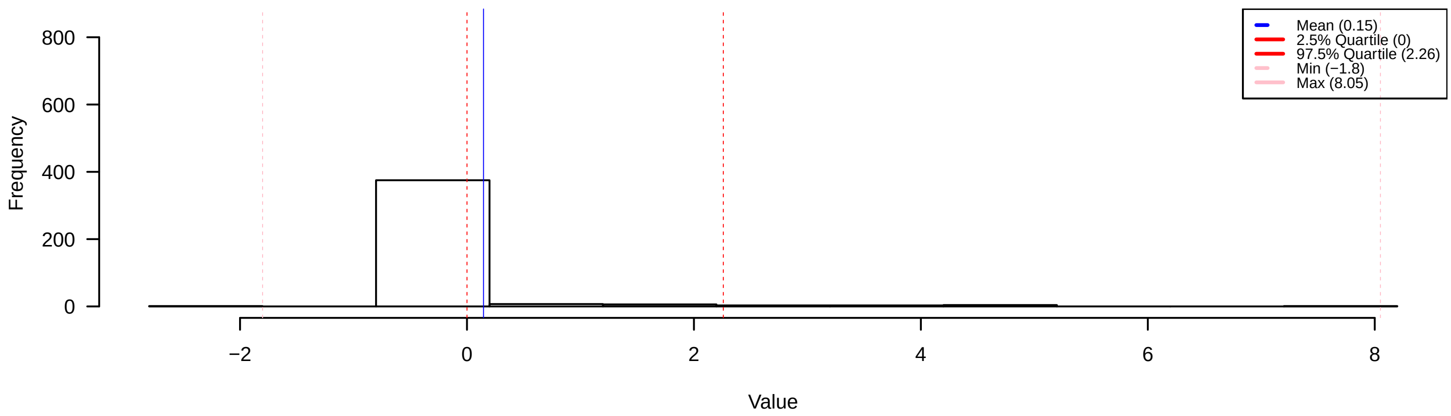

S5, Figure 227 : Bootstrap Distribution of Absolute Humidity:Relative Humidity lag 6

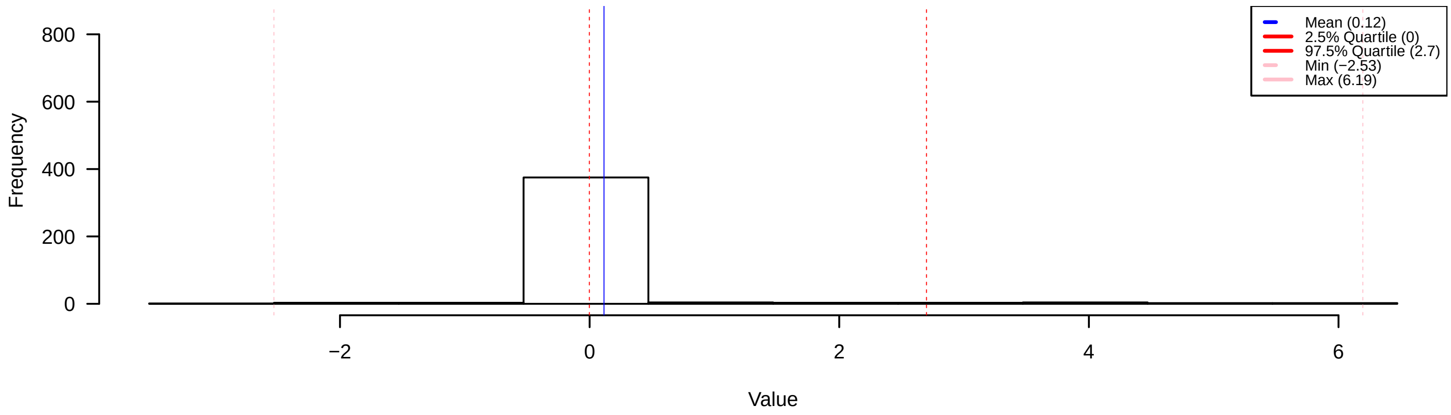

S5, Figure 228 : Bootstrap Distribution of Absolute Humidity:Relative Humidity lag 7

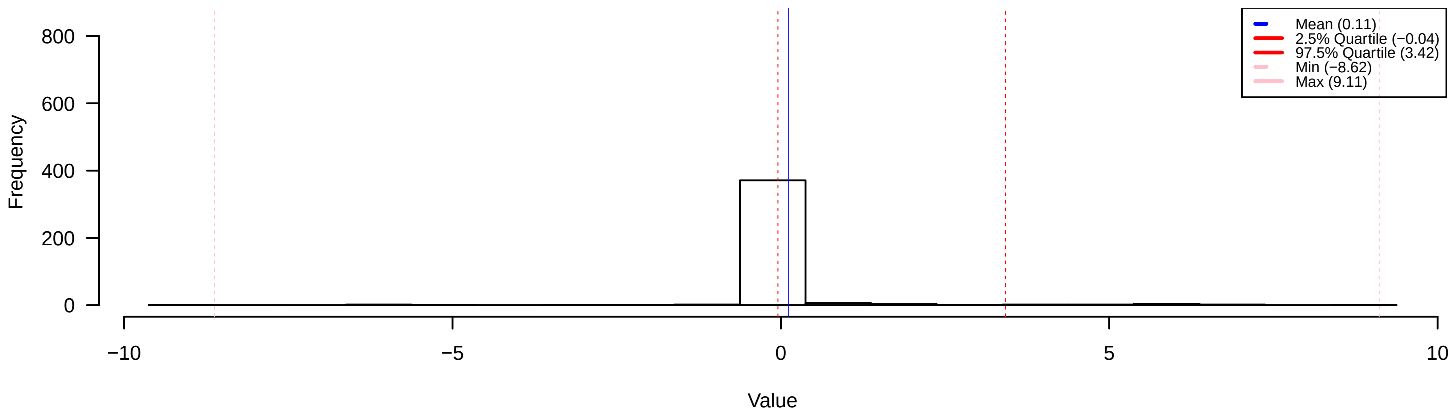

S5, Figure 229 : Bootstrap Distribution of Absolute Humidity:Relative Humidity lag 8

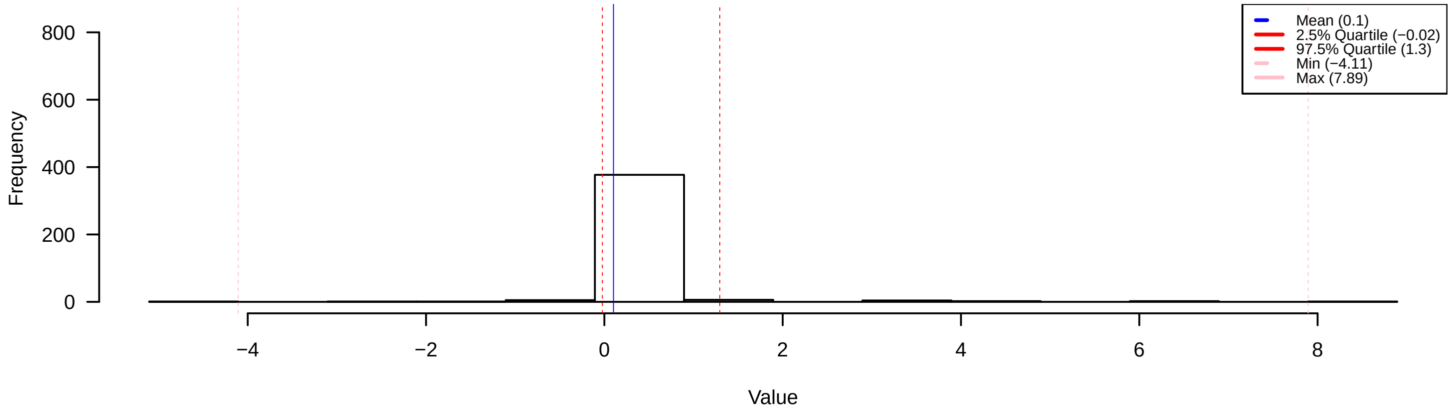

S5, Figure 230 : Bootstrap Distribution of Absolute Humidity:Relative Humidity lag 9

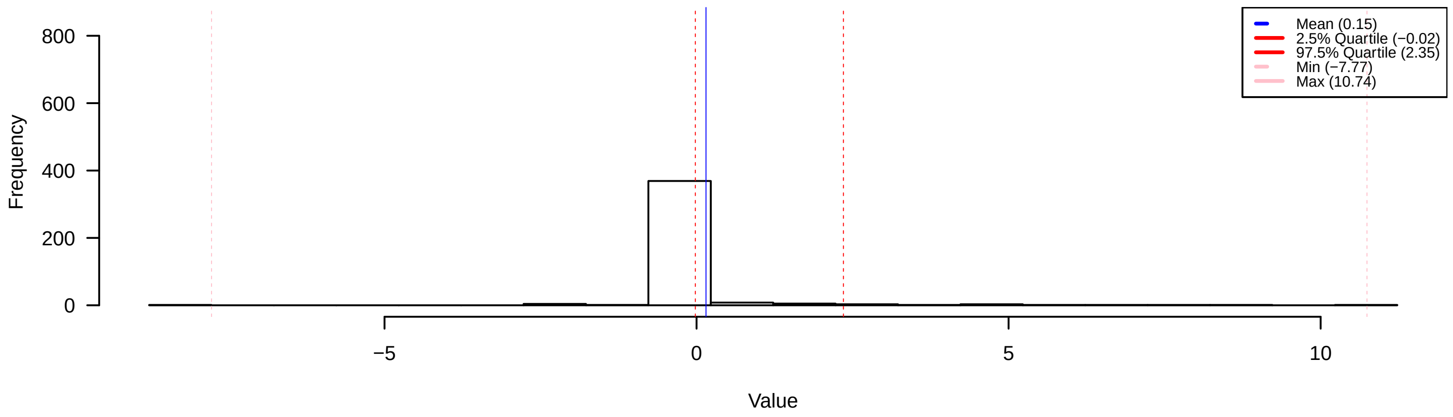

S5, Figure 231 : Bootstrap Distribution of Absolute Humidity:Relative Humidity lag 10

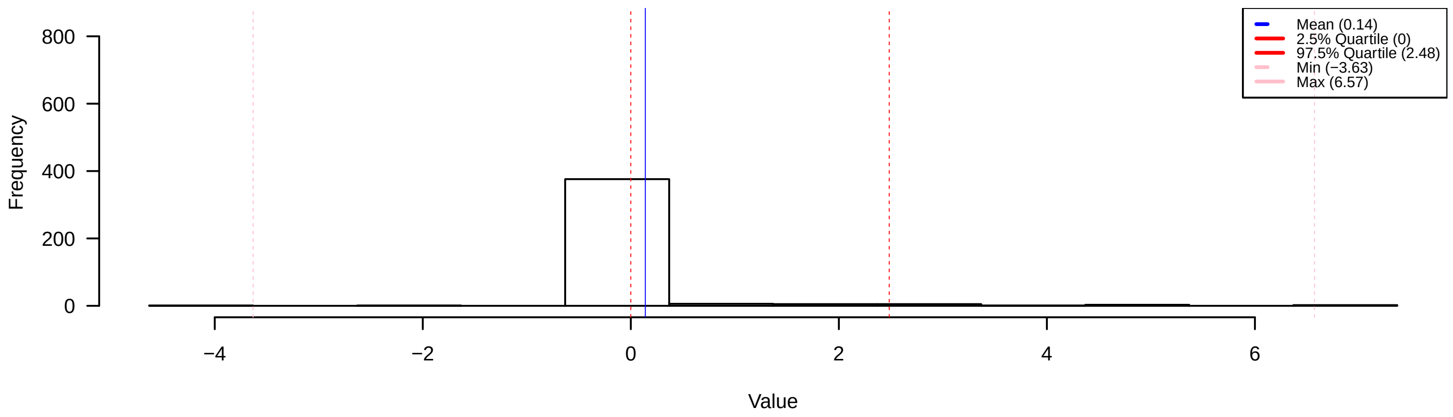

S5, Figure 232 : Bootstrap Distribution of Absolute Humidity:Relative Humidity lag 11

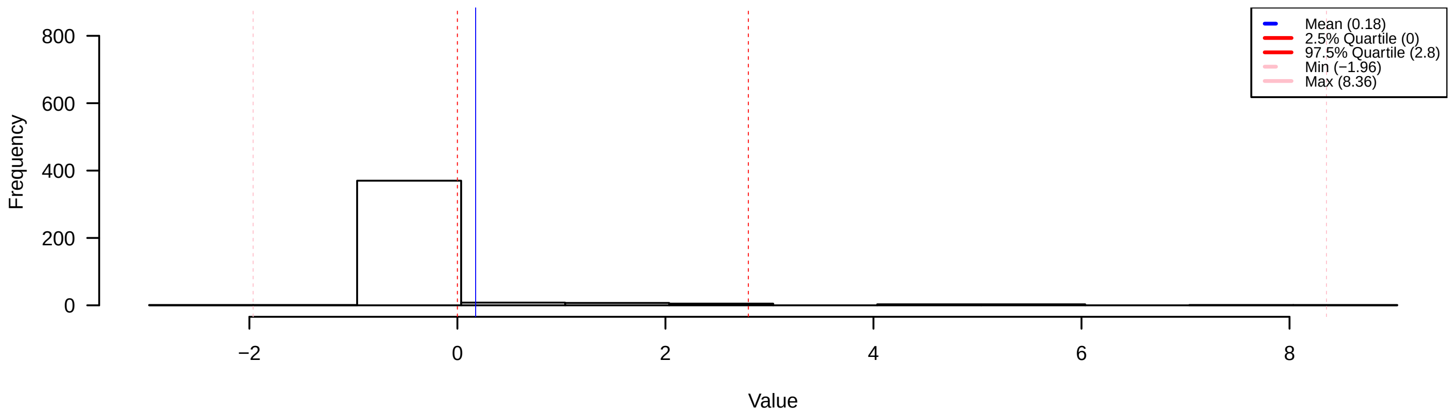

S5, Figure 233 : Bootstrap Distribution of Absolute Humidity:Relative Humidity lag 12

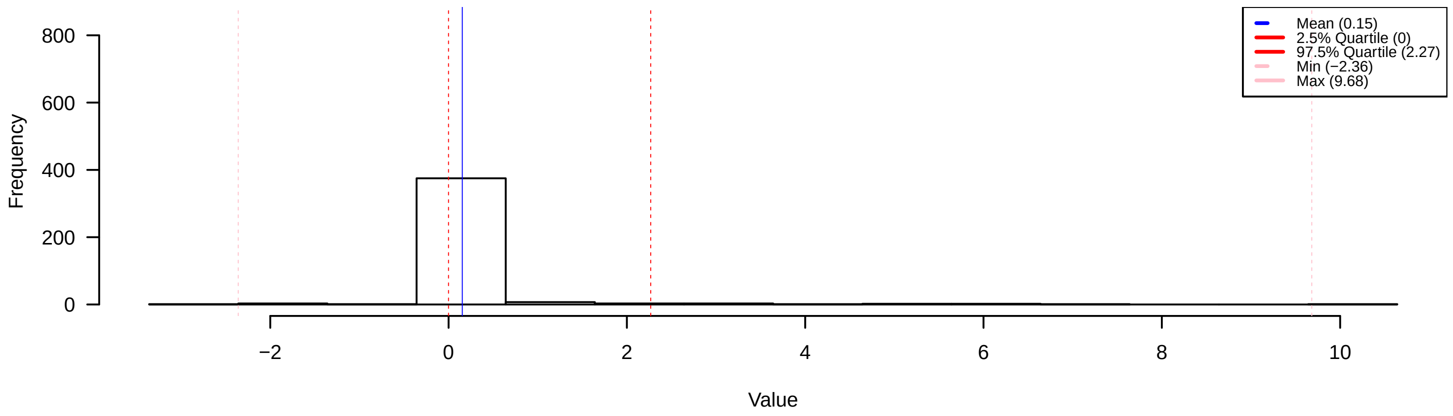

S5, Figure 234 : Bootstrap Distribution of Absolute Humidity:Relative Humidity lag 13

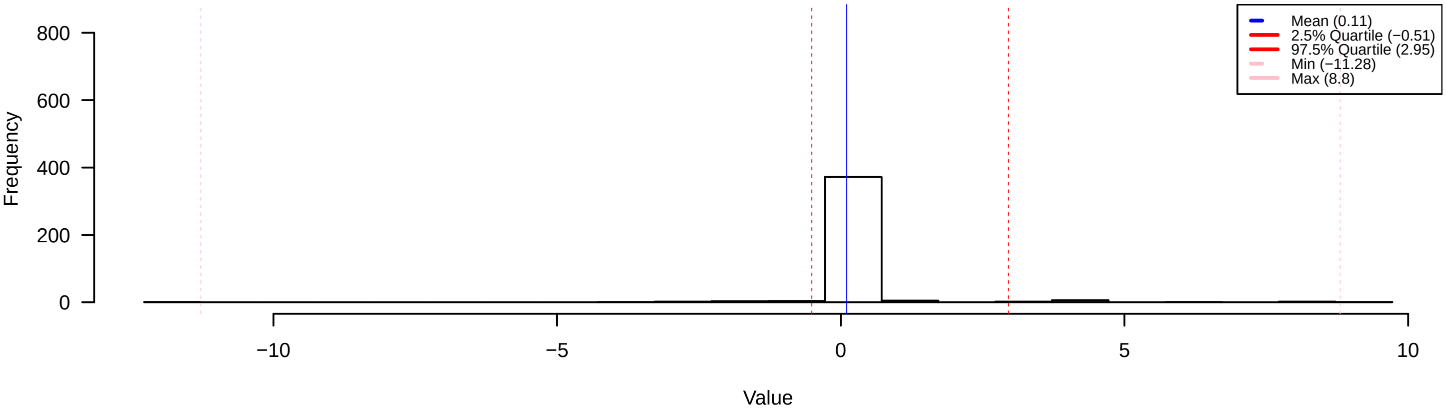

S5, Figure 235 : Bootstrap Distribution of Absolute Humidity:Relative Humidity lag 14

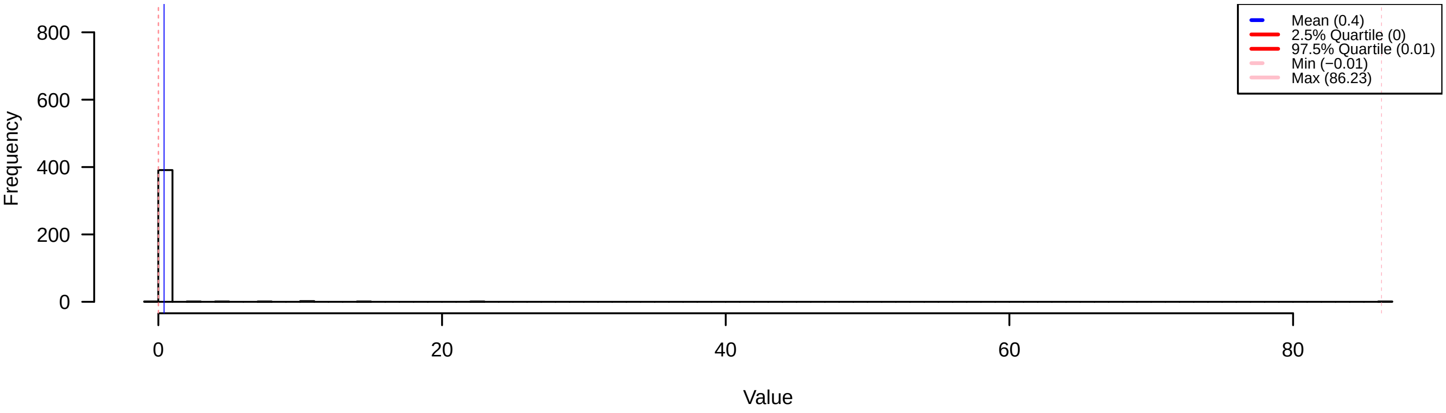

S5, Figure 236 : Bootstrap Distribution of Absolute Humidity:Relative Humidity lag 15

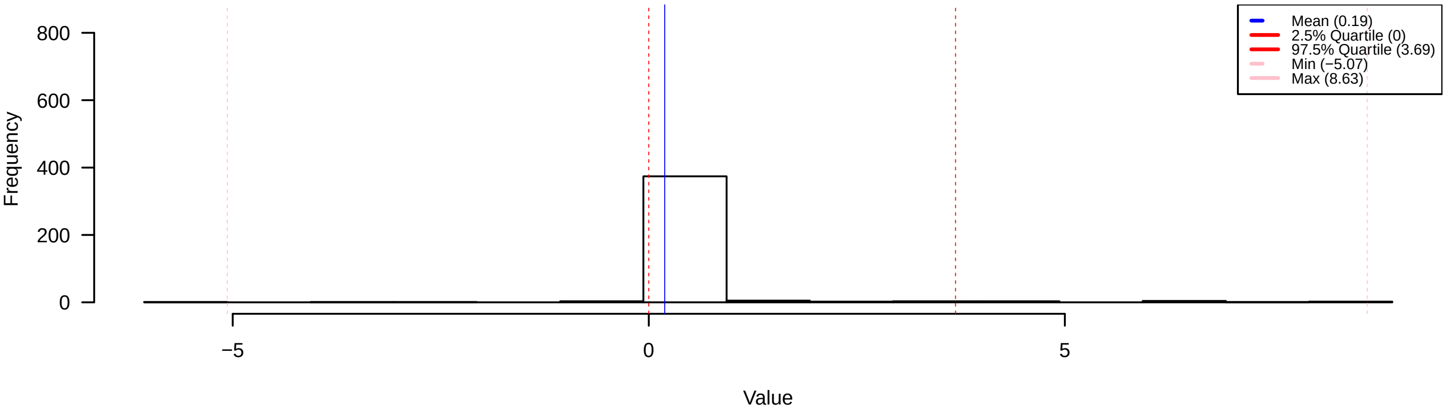

S5, Figure 237 : Bootstrap Distribution of Absolute Humidity:Relative Humidity lag 16

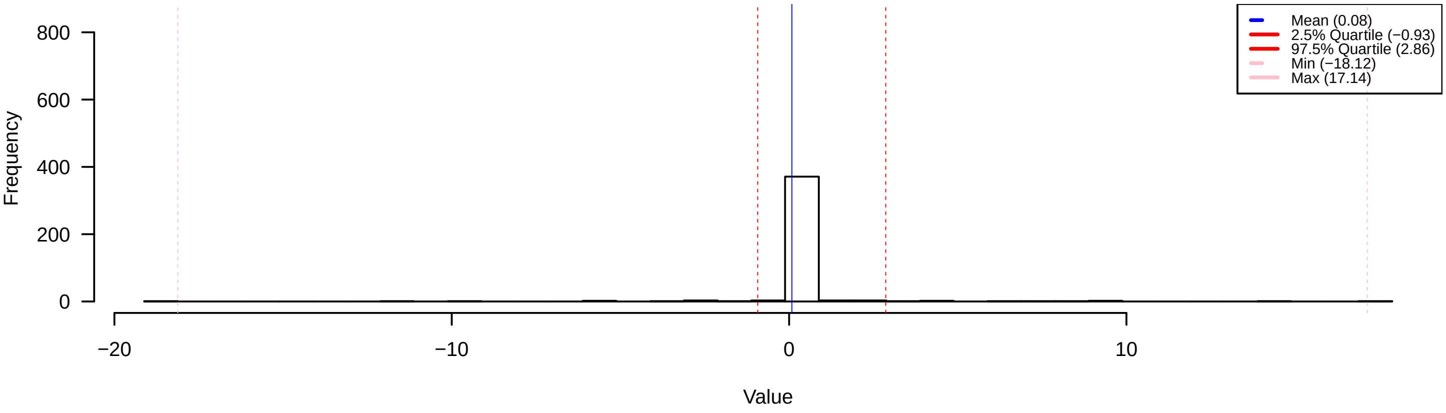

S5, Figure 238 : Bootstrap Distribution of Absolute Humidity:Relative Humidity lag 17

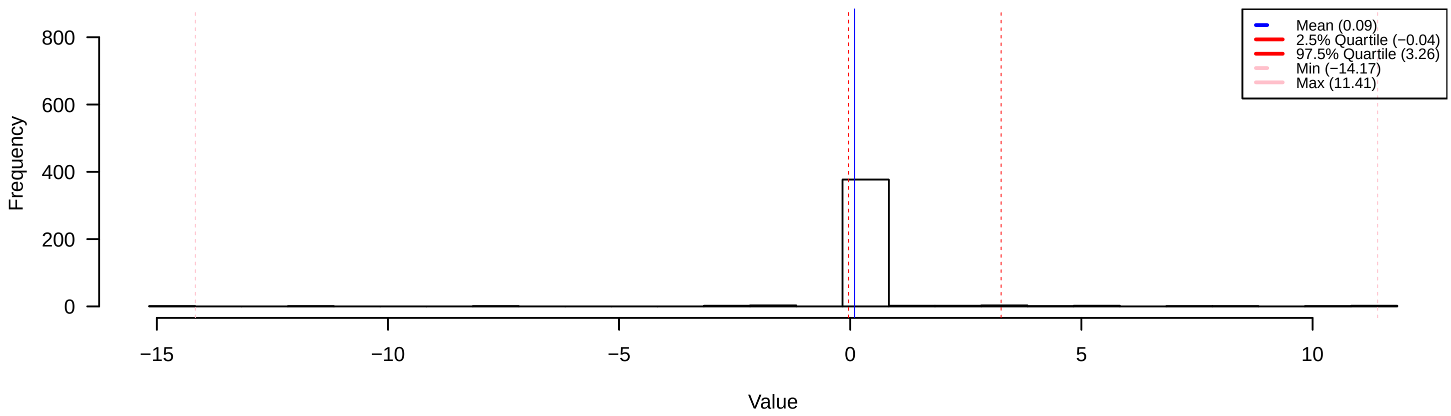

S5, Figure 239 : Bootstrap Distribution of Absolute Humidity:Relative Humidity lag 18

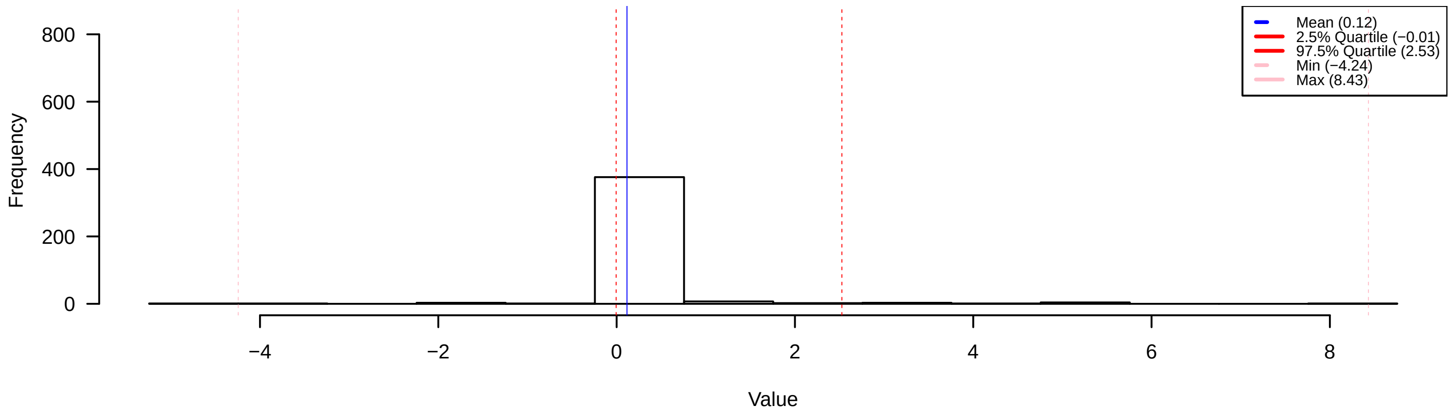

S5, Figure 240 : Bootstrap Distribution of Absolute Humidity:Relative Humidity lag 19

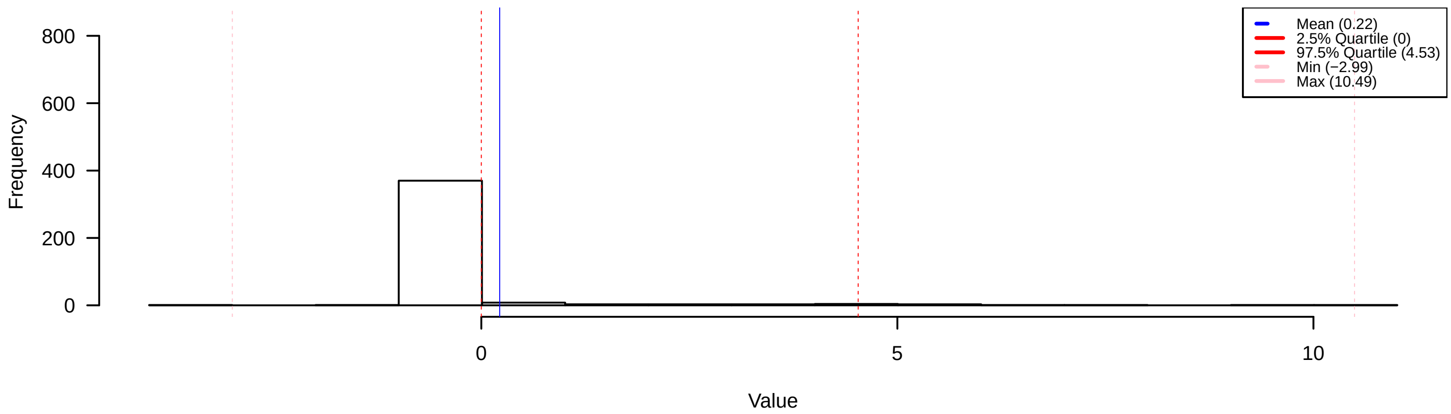

S5, Figure 241 : Bootstrap Distribution of Absolute Humidity:Relative Humidity lag 20

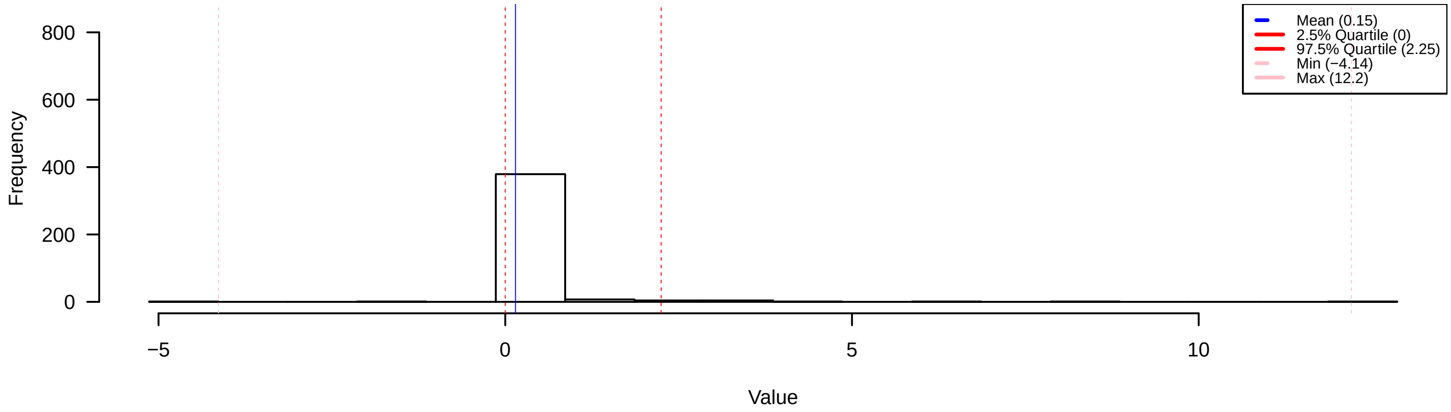

S5, Figure 242 : Bootstrap Distribution of Dewpoint Temperature Squared lag 1

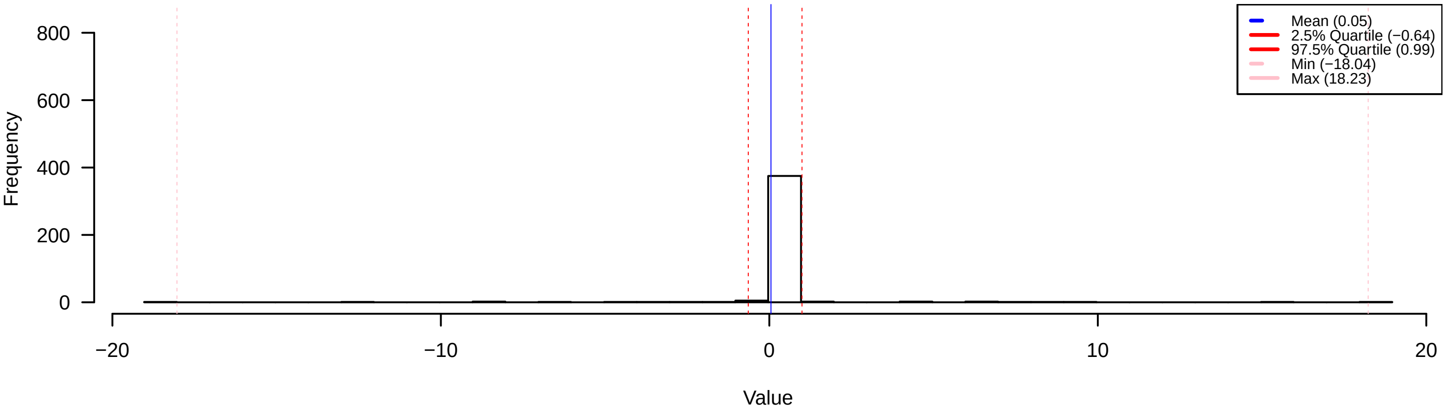

S5, Figure 243 : Bootstrap Distribution of Dewpoint Temperature Squared lag 2

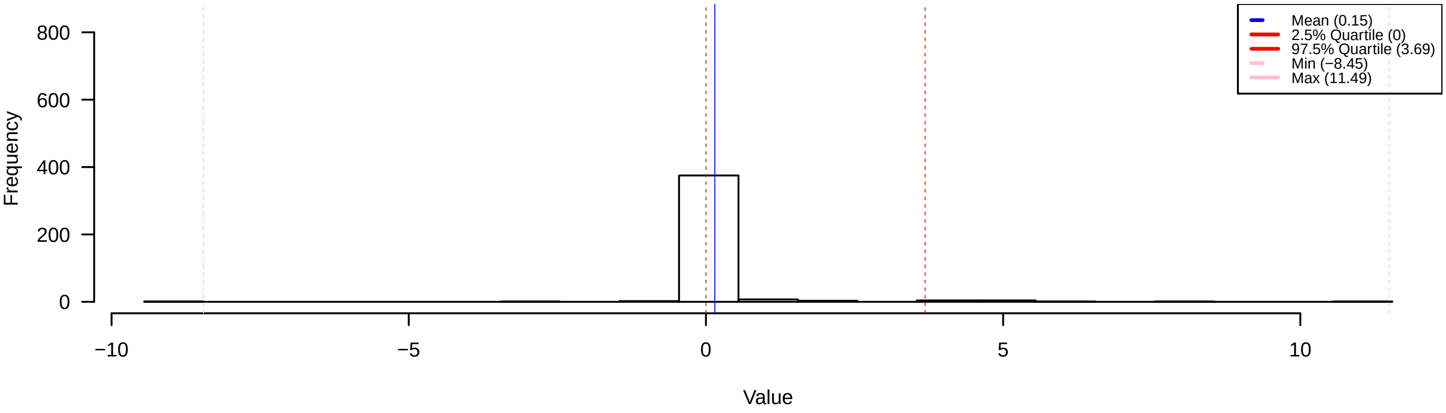

S5, Figure 244 : Bootstrap Distribution of Dewpoint Temperature Squared lag 3

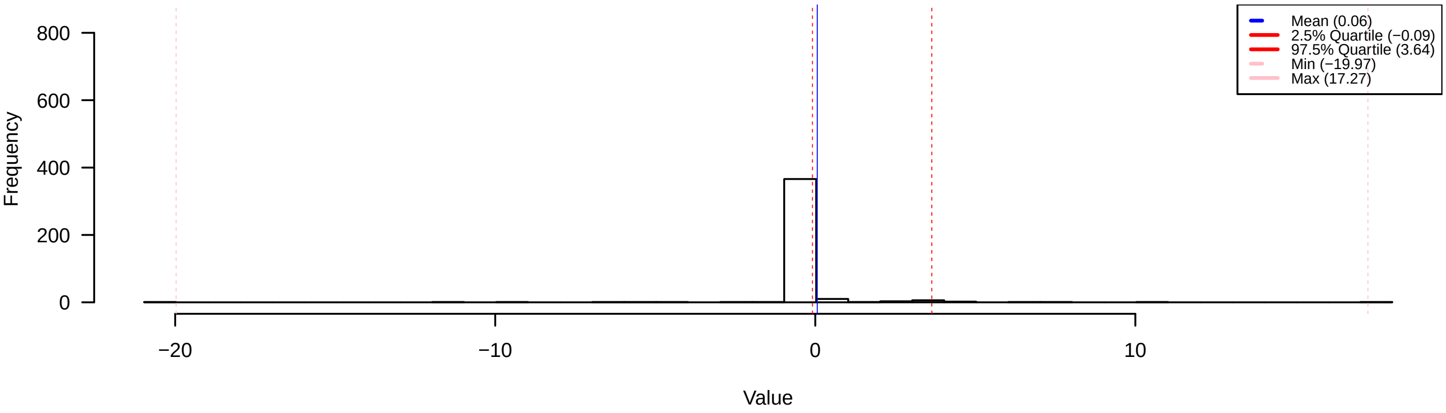

S5, Figure 245 : Bootstrap Distribution of Dewpoint Temperature Squared lag 4

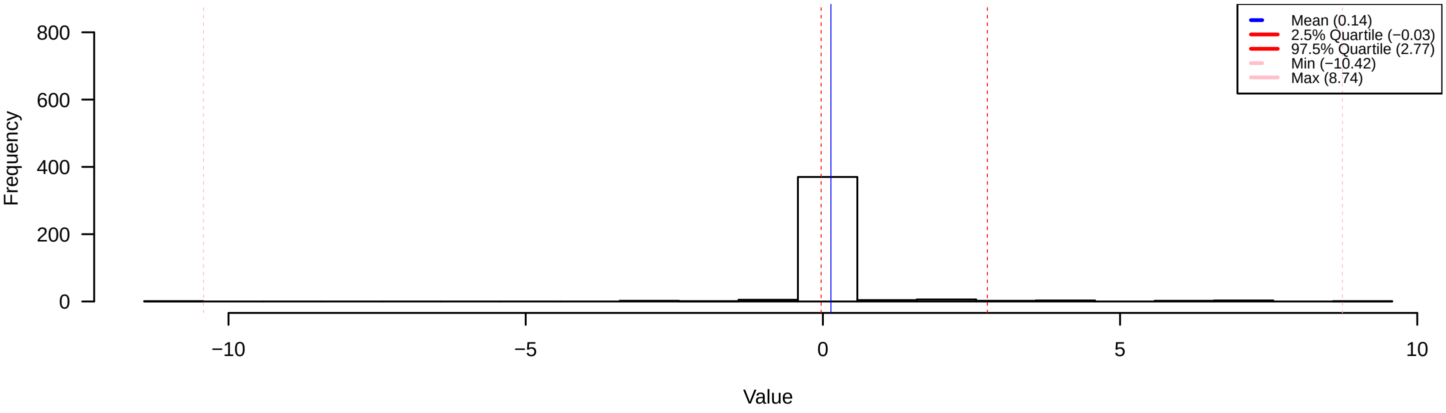

S5, Figure 246 : Bootstrap Distribution of Dewpoint Temperature Squared lag 5

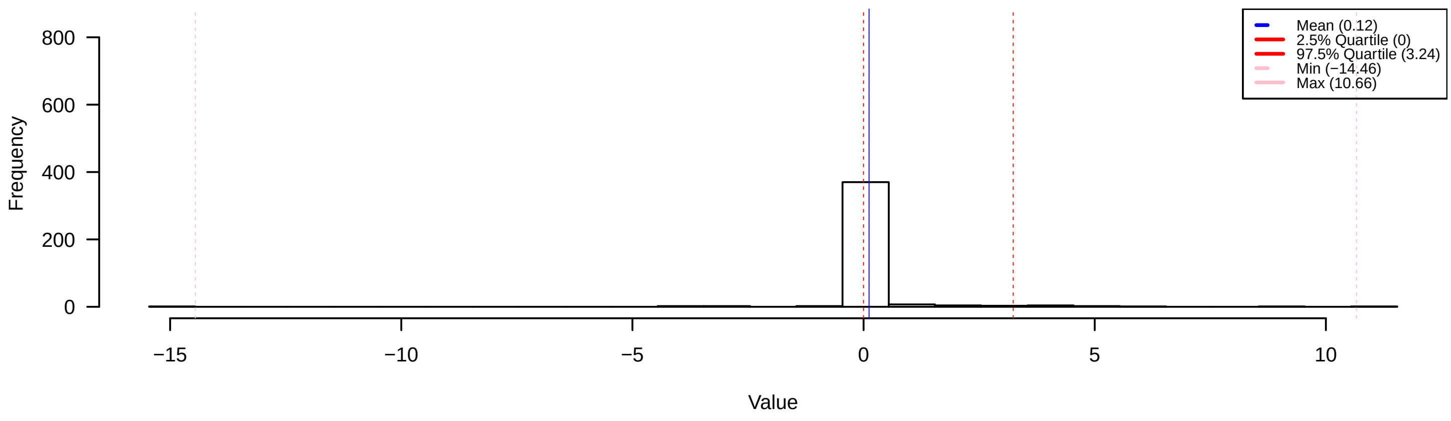

S5, Figure 247 : Bootstrap Distribution of Dewpoint Temperature Squared lag 6

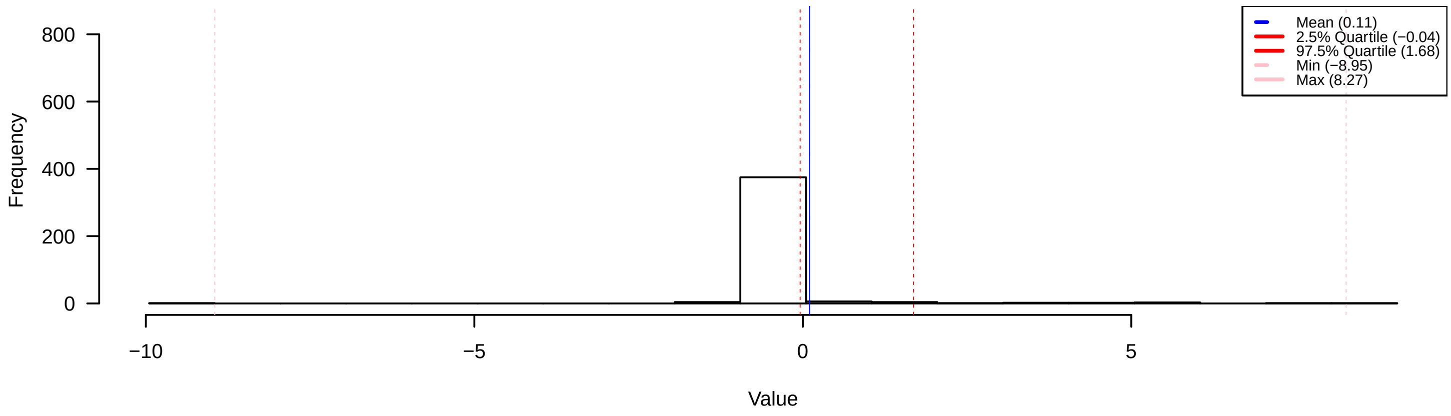

S5, Figure 248 : Bootstrap Distribution of Dewpoint Temperature Squared lag 7

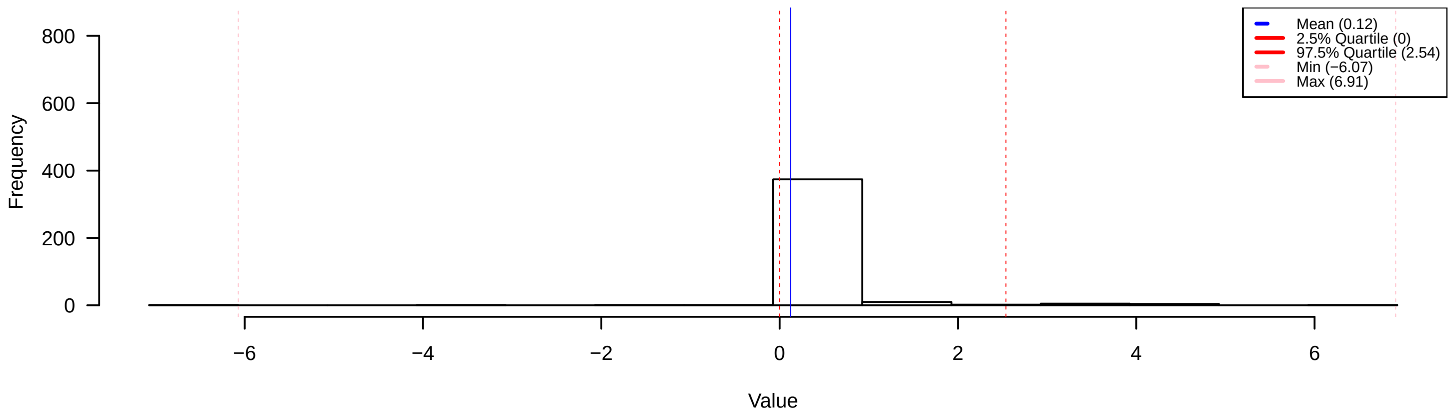

S5, Figure 249 : Bootstrap Distribution of Dewpoint Temperature Squared lag 8

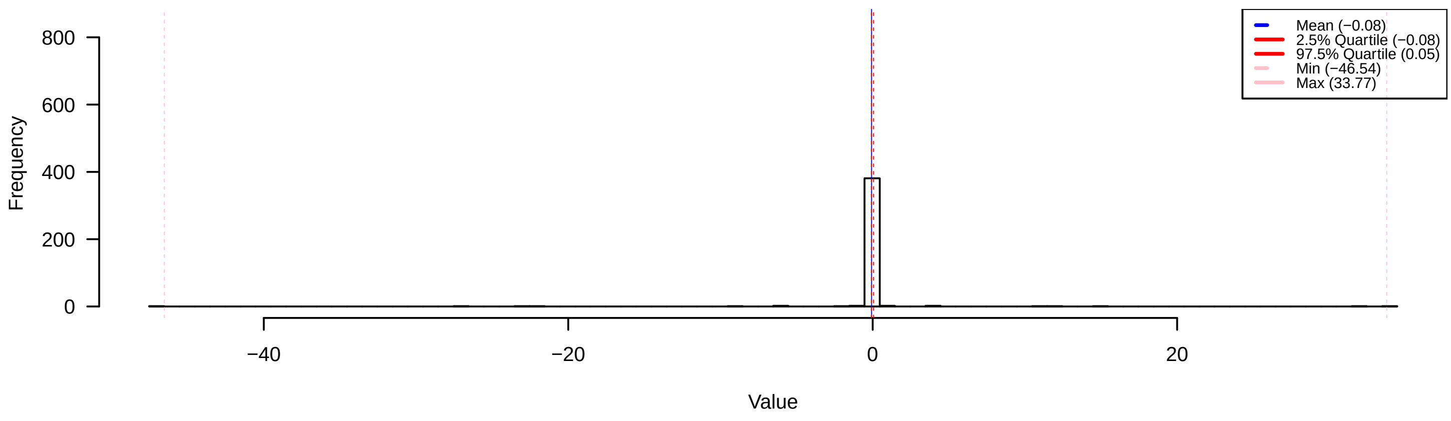

S5, Figure 250 : Bootstrap Distribution of Dewpoint Temperature Squared lag 9

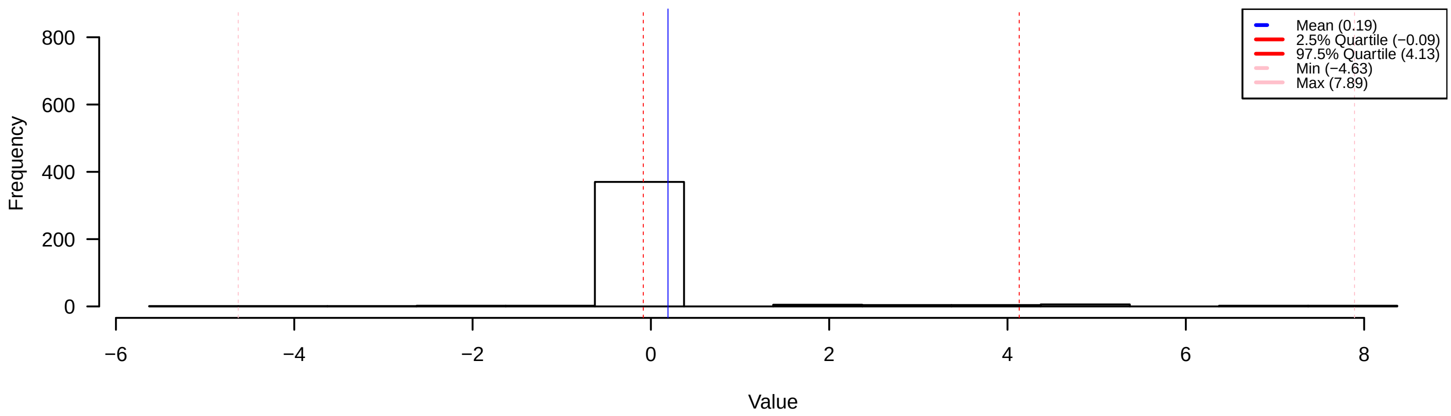

S5, Figure 251 : Bootstrap Distribution of Dewpoint Temperature Squared lag 10

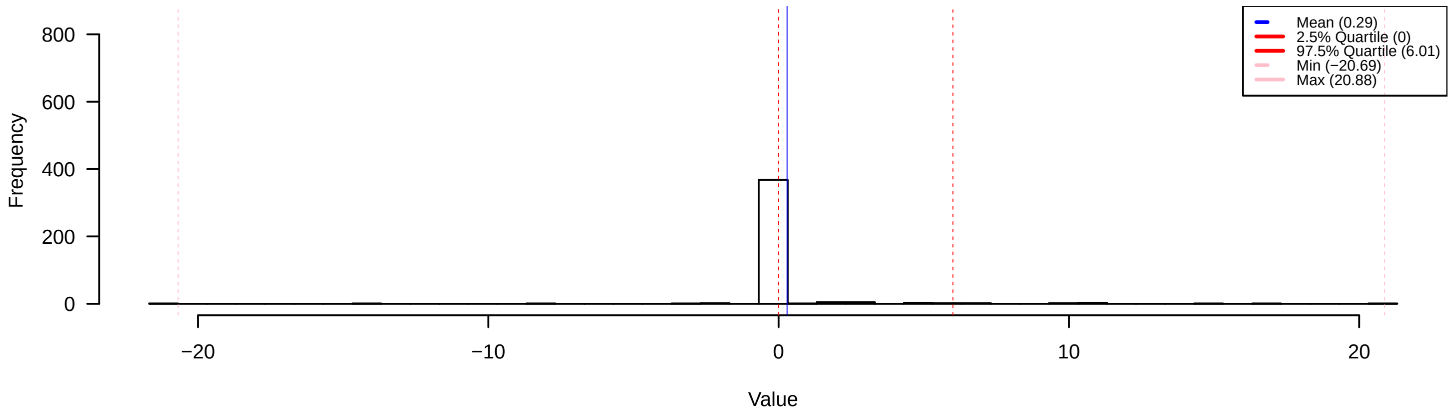

S5, Figure 252 : Bootstrap Distribution of Dewpoint Temperature Squared lag 11

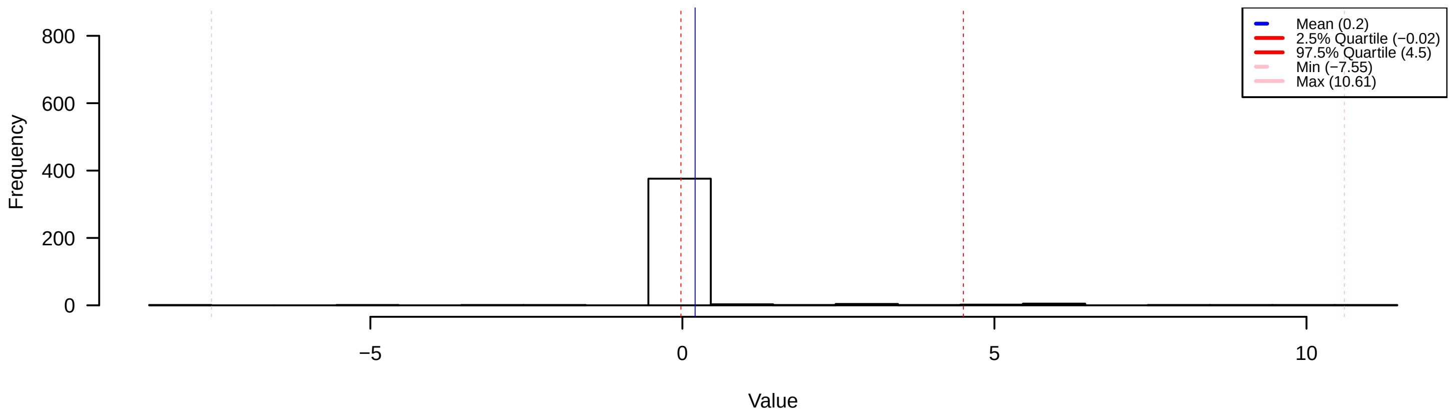

S5, Figure 253 : Bootstrap Distribution of Dewpoint Temperature Squared lag 12

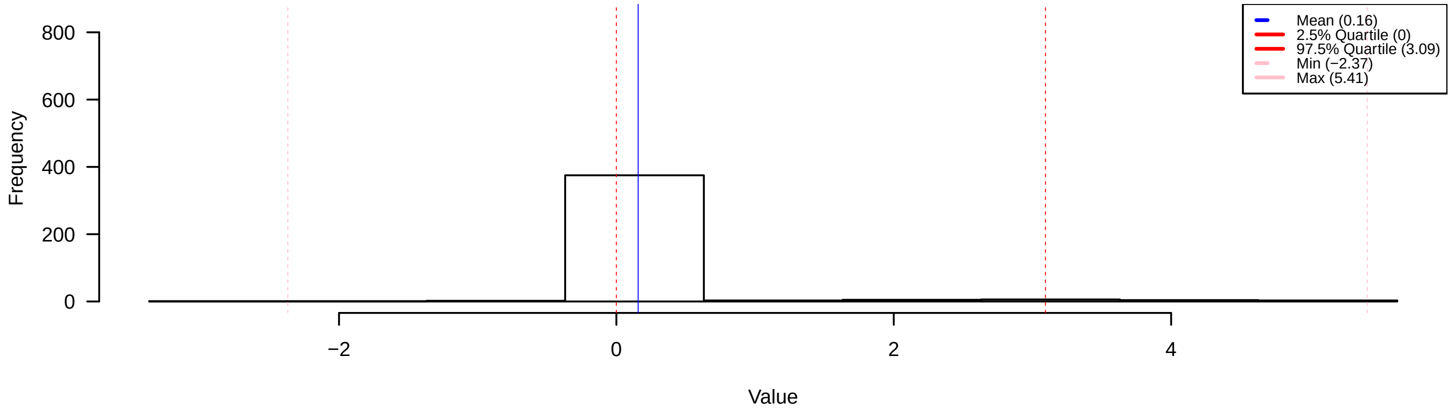

S5, Figure 254 : Bootstrap Distribution of Dewpoint Temperature Squared lag 13

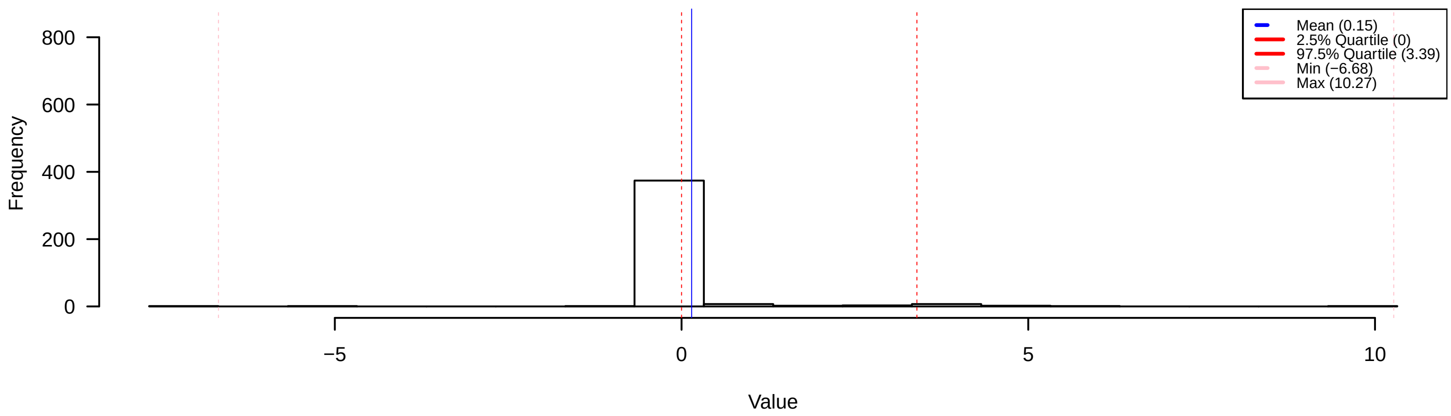

S5, Figure 255 : Bootstrap Distribution of Dewpoint Temperature Squared lag 14

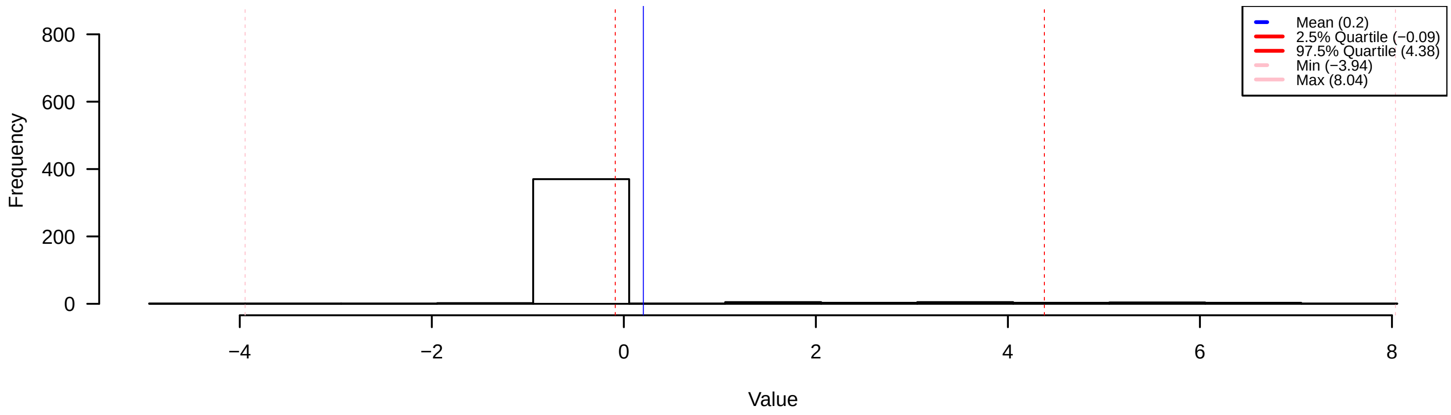

S5, Figure 256 : Bootstrap Distribution of Dewpoint Temperature Squared lag 15

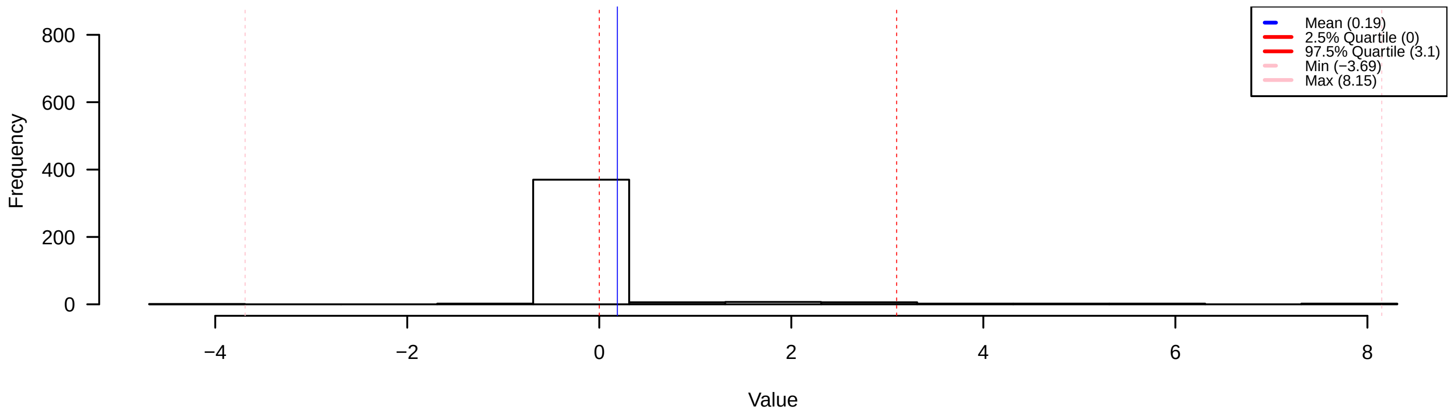

S5, Figure 257 : Bootstrap Distribution of Dewpoint Temperature Squared lag 16

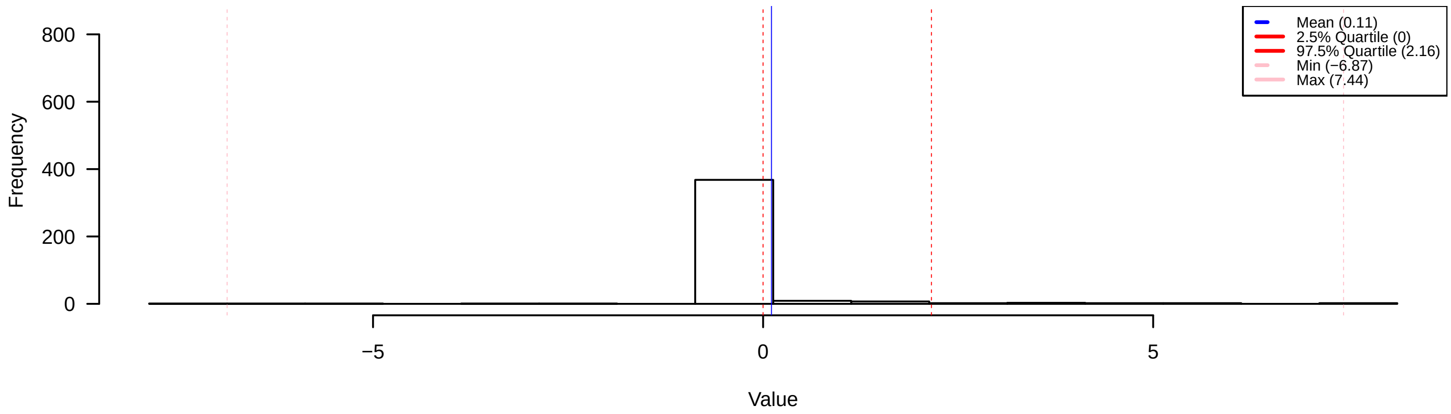

S5, Figure 258 : Bootstrap Distribution of Dewpoint Temperature Squared lag 17

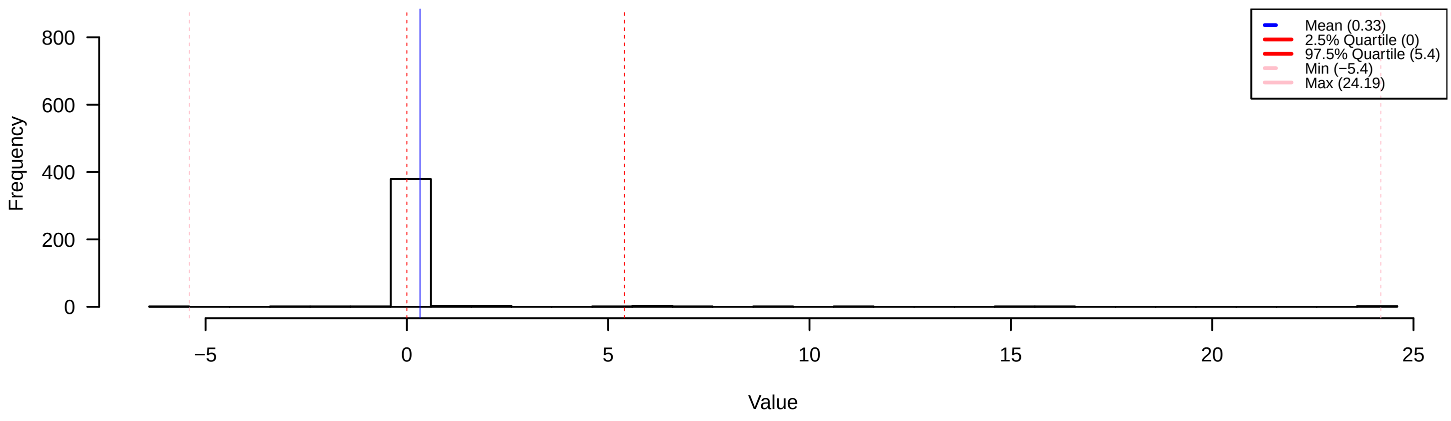

S5, Figure 259 : Bootstrap Distribution of Dewpoint Temperature Squared lag 18

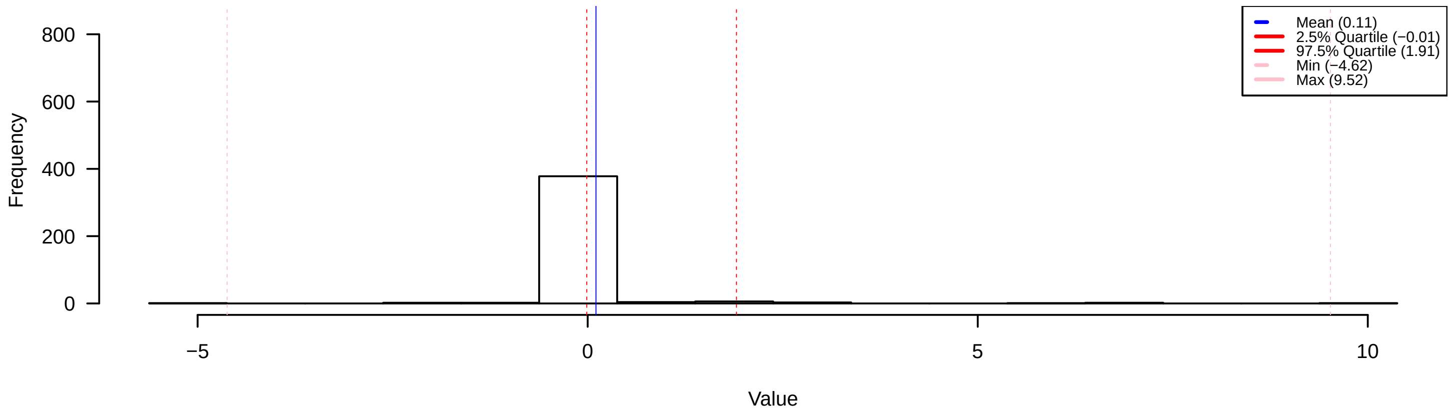

S5, Figure 260 : Bootstrap Distribution of Dewpoint Temperature Squared lag 19

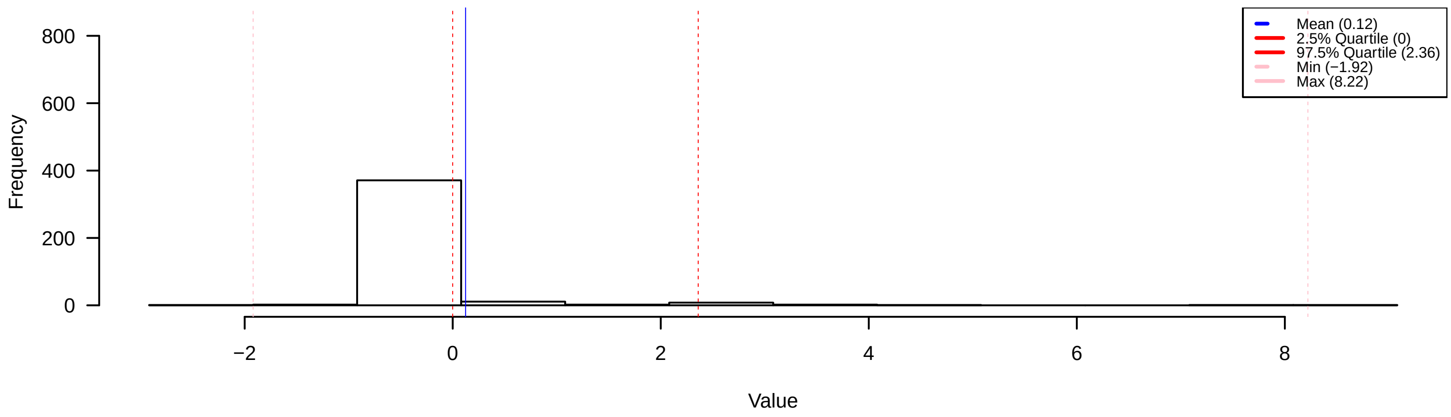

S5, Figure 261 : Bootstrap Distribution of Dewpoint Temperature Squared lag 20

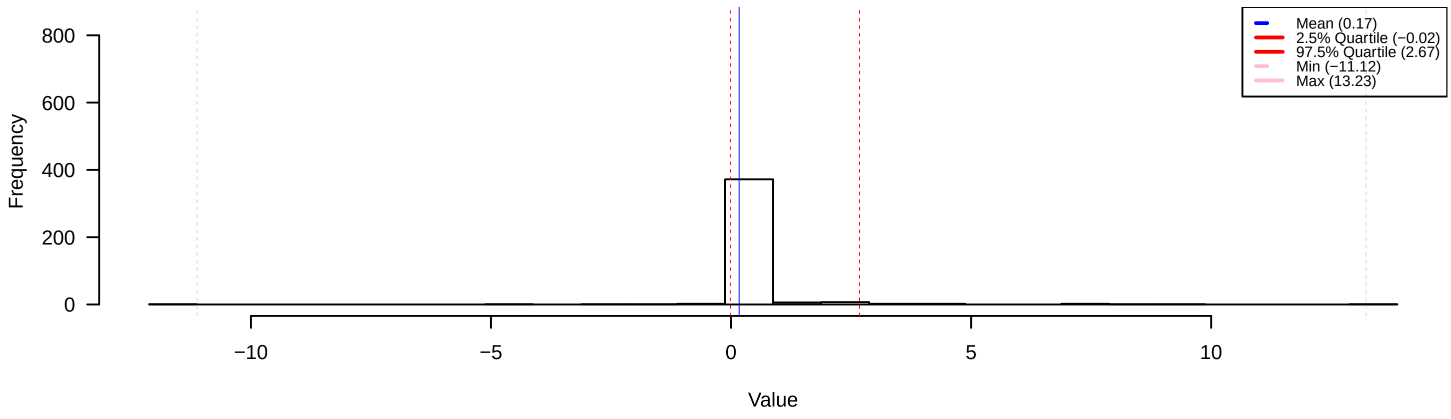

S5, Figure 262 : Bootstrap Distribution of Air Temperature Squared lag 1

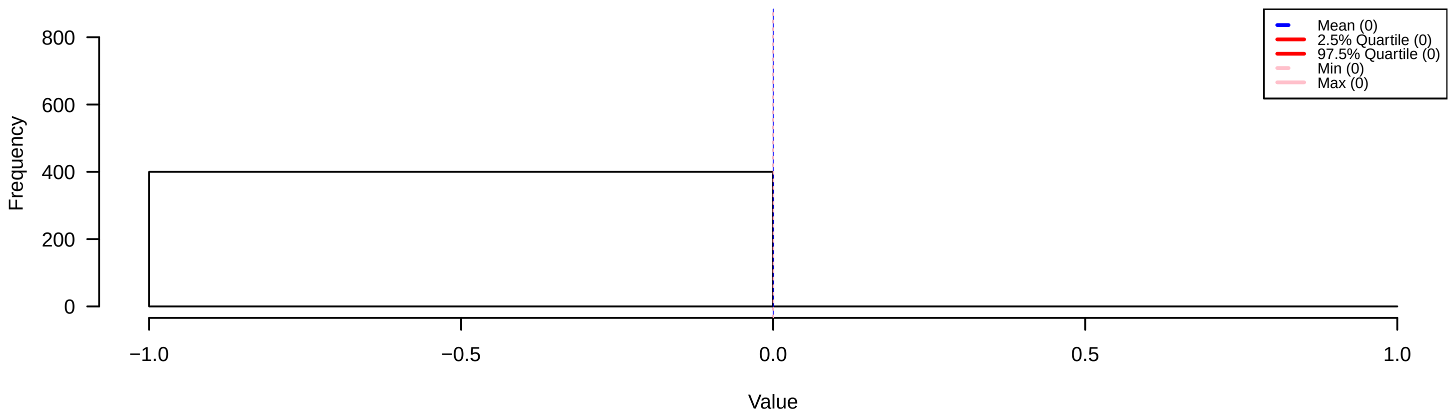

S5, Figure 263 : Bootstrap Distribution of Air Temperature Squared lag 2

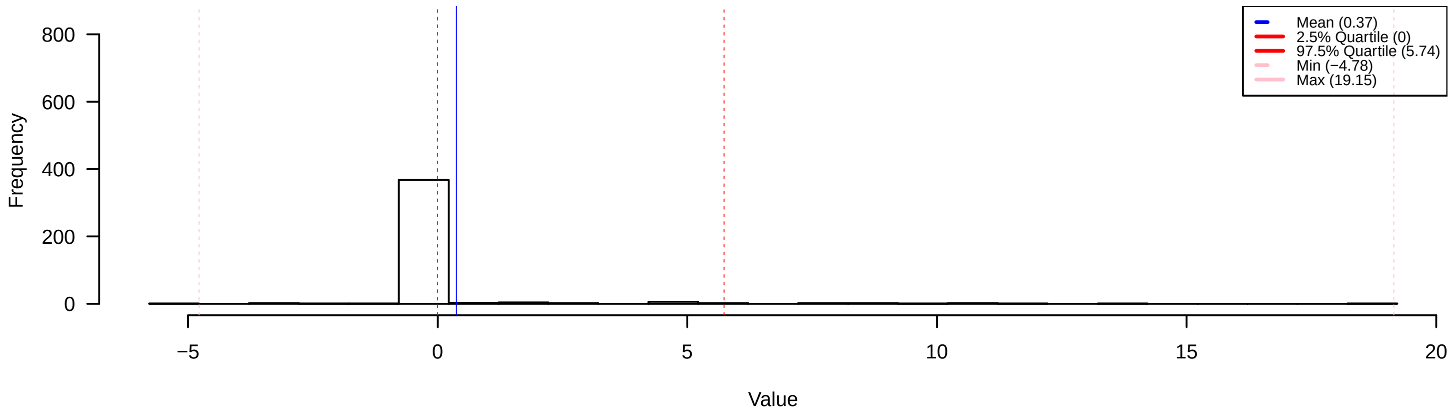

S5, Figure 264 : Bootstrap Distribution of Air Temperature Squared lag 3

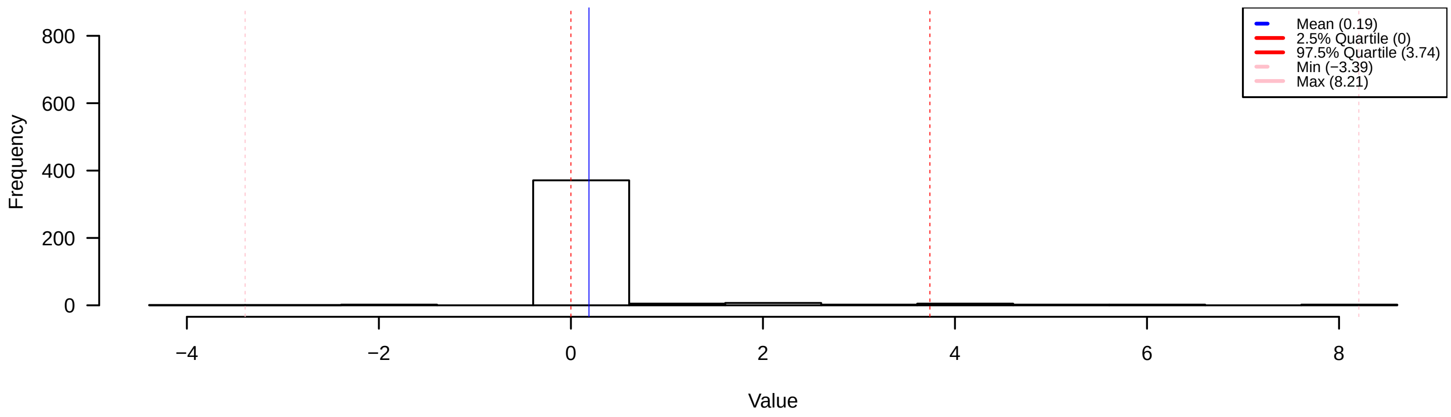

S5, Figure 265 : Bootstrap Distribution of Air Temperature Squared lag 4

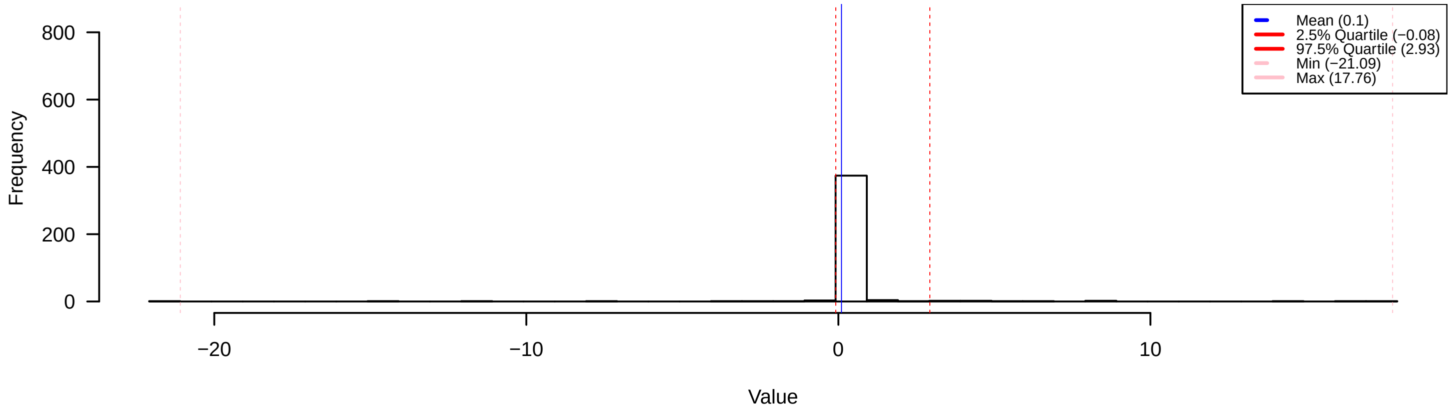

S5, Figure 266 : Bootstrap Distribution of Air Temperature Squared lag 5

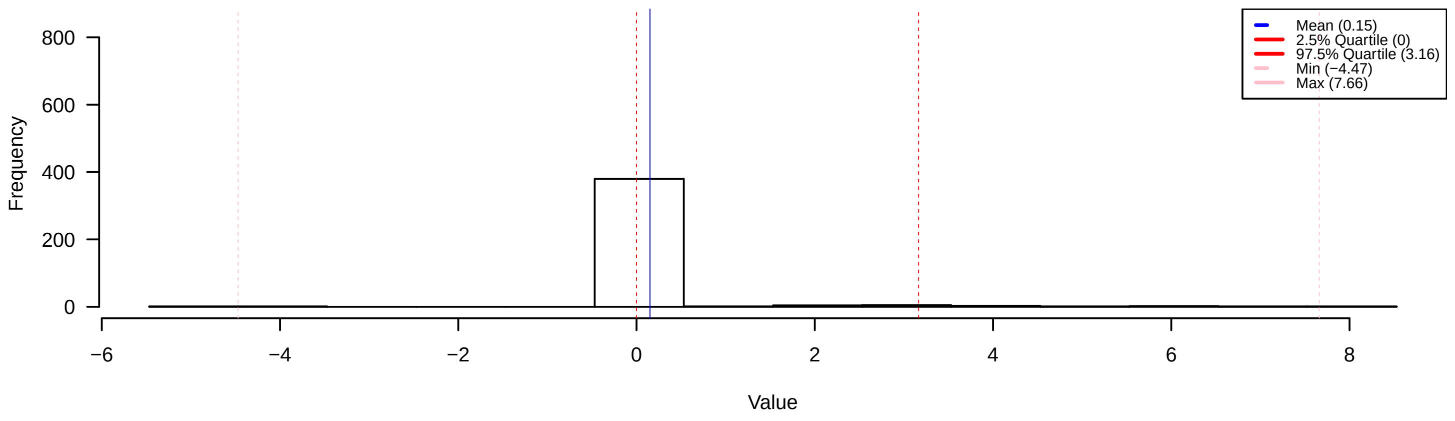

S5, Figure 267 : Bootstrap Distribution of Air Temperature Squared lag 6

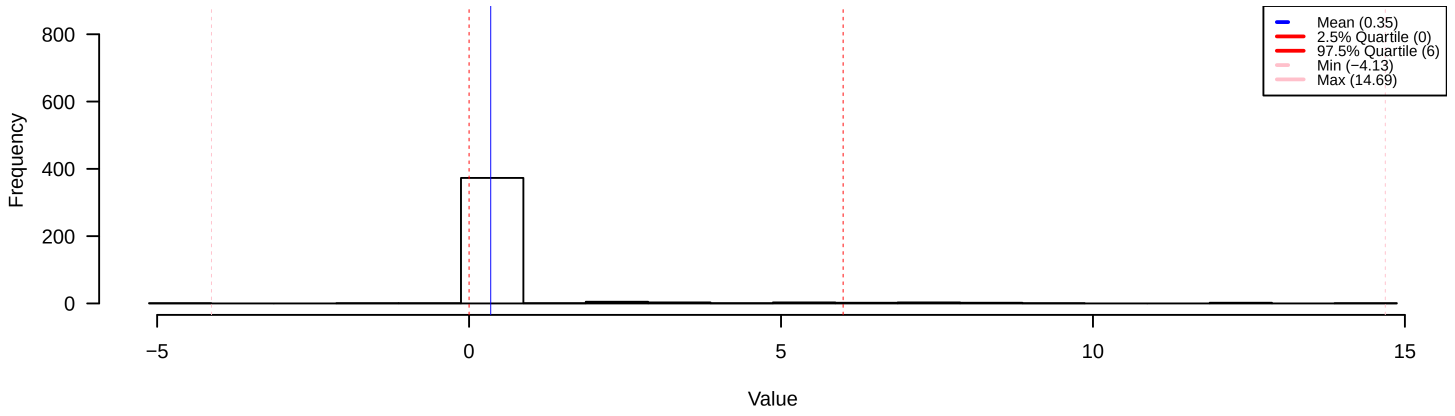

S5, Figure 268 : Bootstrap Distribution of Air Temperature Squared lag 7

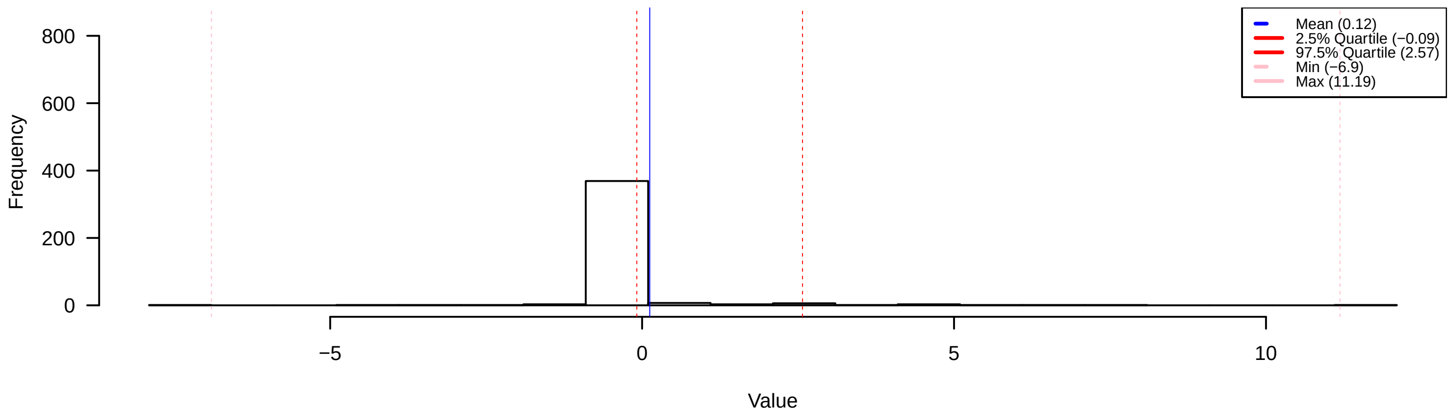

S5, Figure 269 : Bootstrap Distribution of Air Temperature Squared lag 8

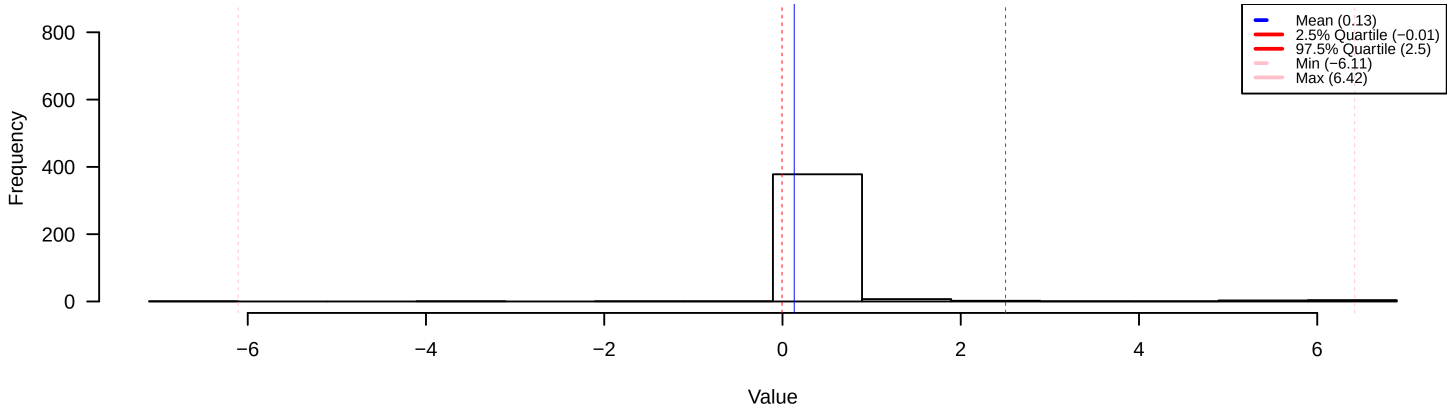

S5, Figure 270 : Bootstrap Distribution of Air Temperature Squared lag 9

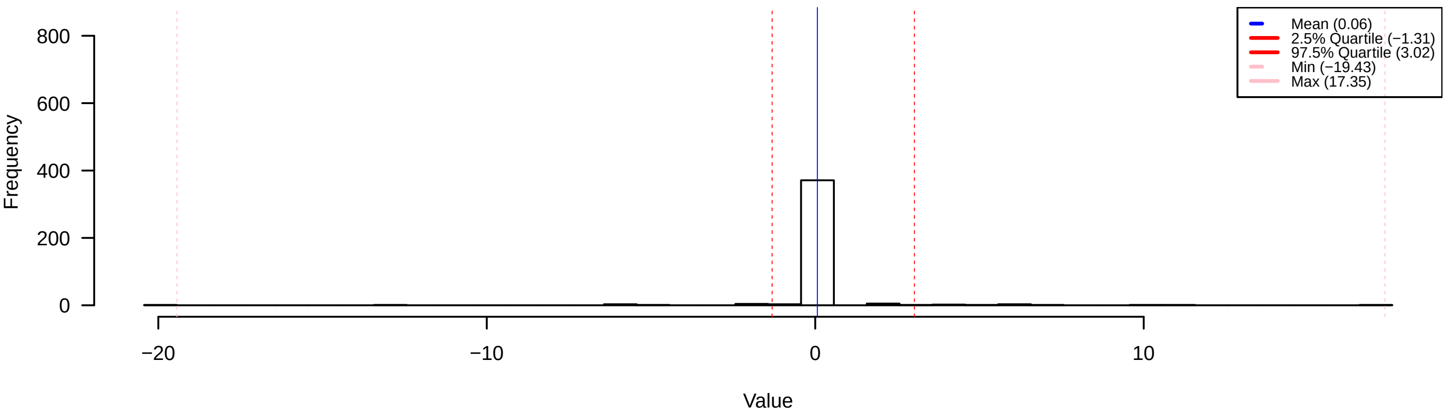

S5, Figure 271 : Bootstrap Distribution of Air Temperature Squared lag 10

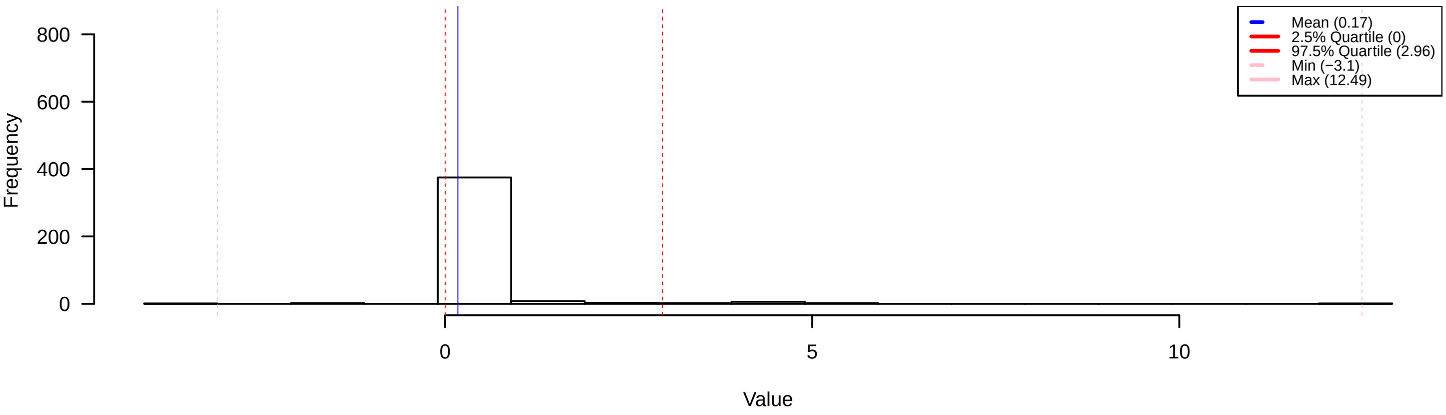

S5, Figure 272 : Bootstrap Distribution of Air Temperature Squared lag 11

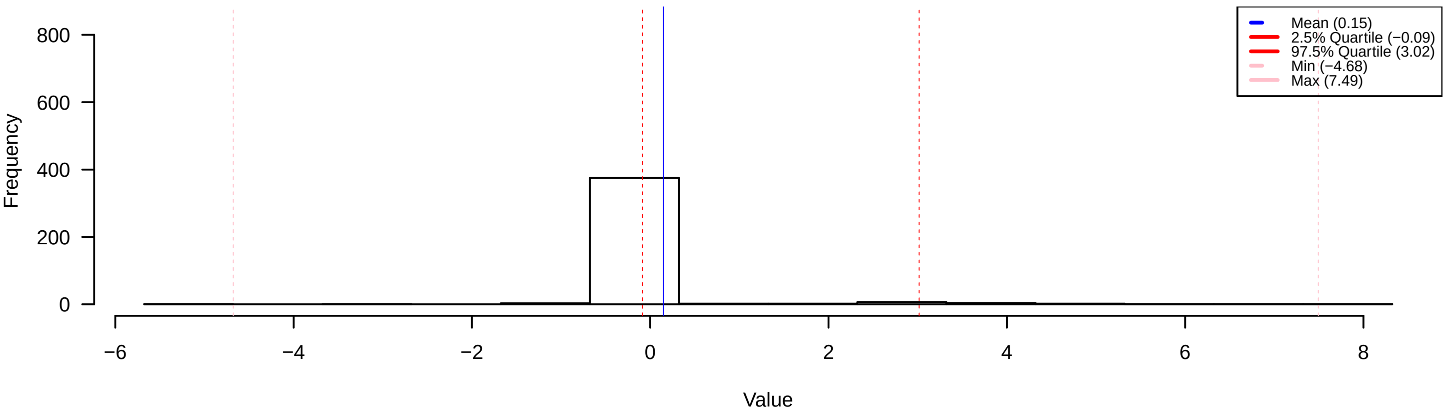

S5, Figure 273 : Bootstrap Distribution of Air Temperature Squared lag 12

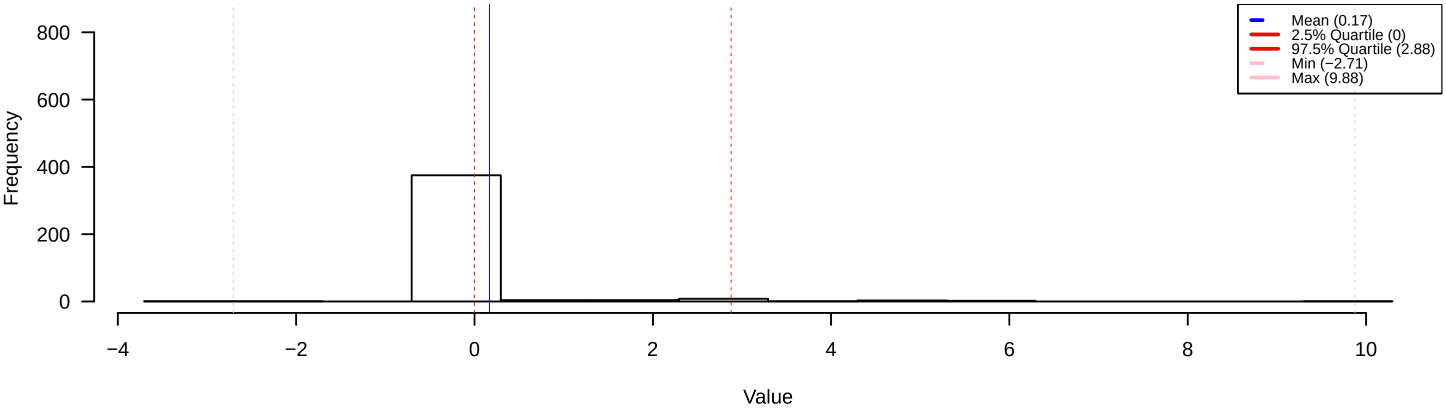

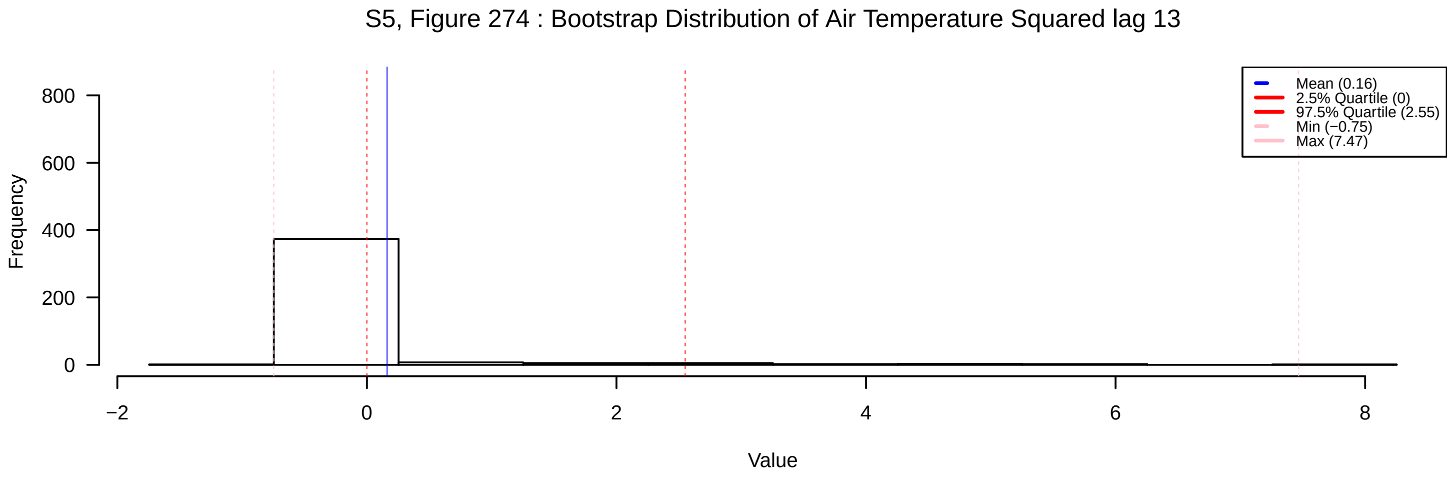
S5, Figure 274 : Bootstrap Distribution of Air Temperature Squared lag 13
Mean (0.16)
2.5% Quartile (0)
97.5% Quartile (2.55)
Min (−0.75)
Max (7.47)
Frequency
Value

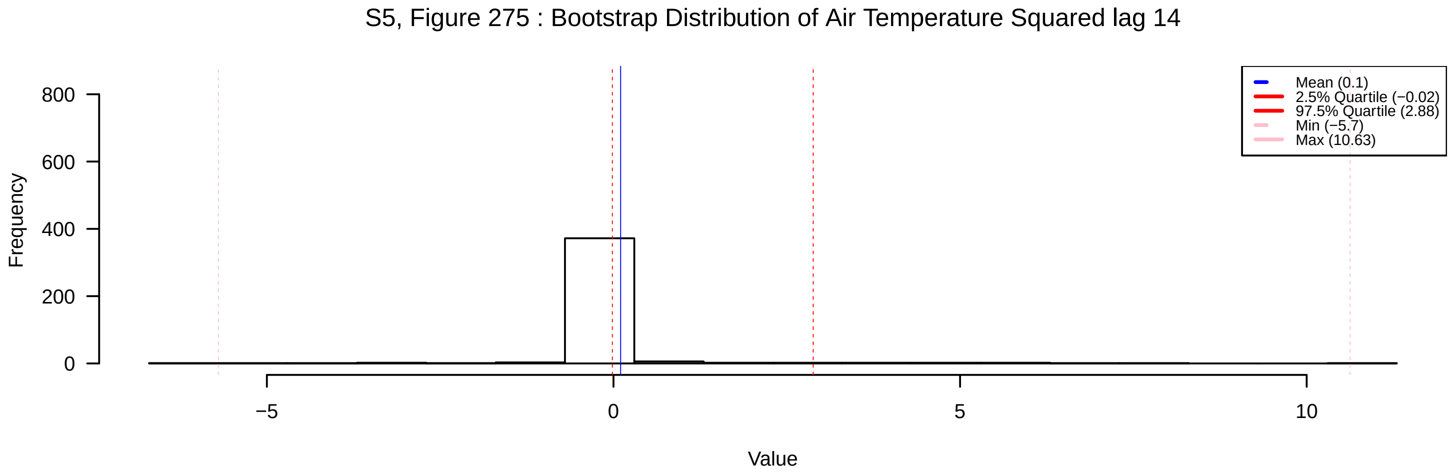
S5, Figure 275 : Bootstrap Distribution of Air Temperature Squared lag 14
Mean (0.1)
2.5% Quartile (−0.02)
97.5% Quartile (2.88)
Min (−5.7)
Max (10.63)
Frequency
Value

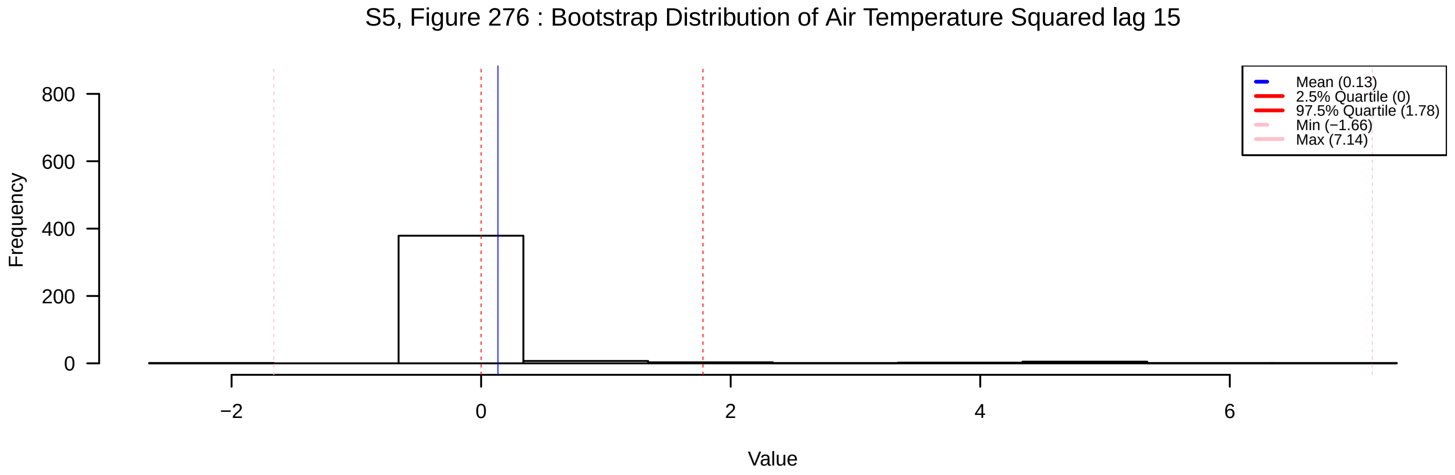
S5, Figure 276 : Bootstrap Distribution of Air Temperature Squared lag 15
Mean (0.13)
2.5% Quartile (0)
97.5% Quartile (1.78)
Min (−1.66)
Max (7.14)
Frequency
Value

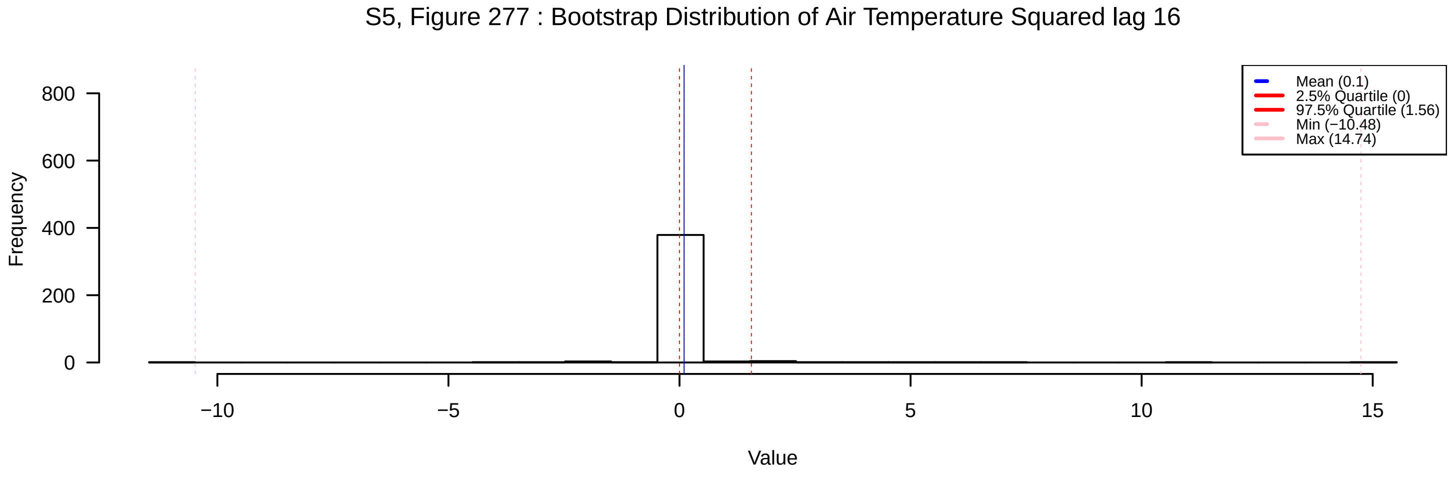
S5, Figure 277 : Bootstrap Distribution of Air Temperature Squared lag 16
Mean (0.1)
2.5% Quartile (0)
97.5% Quartile (1.56)
Min (−10.48)
Max (14.74)
Frequency
Value

S5, Figure 278 : Bootstrap Distribution of Air Temperature Squared lag 17

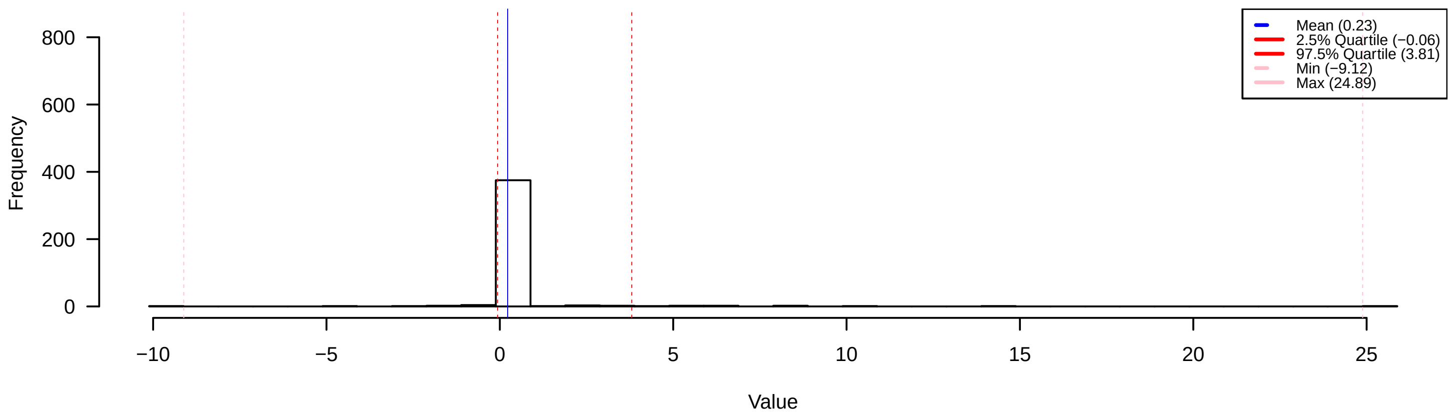

S5, Figure 279 : Bootstrap Distribution of Air Temperature Squared lag 18

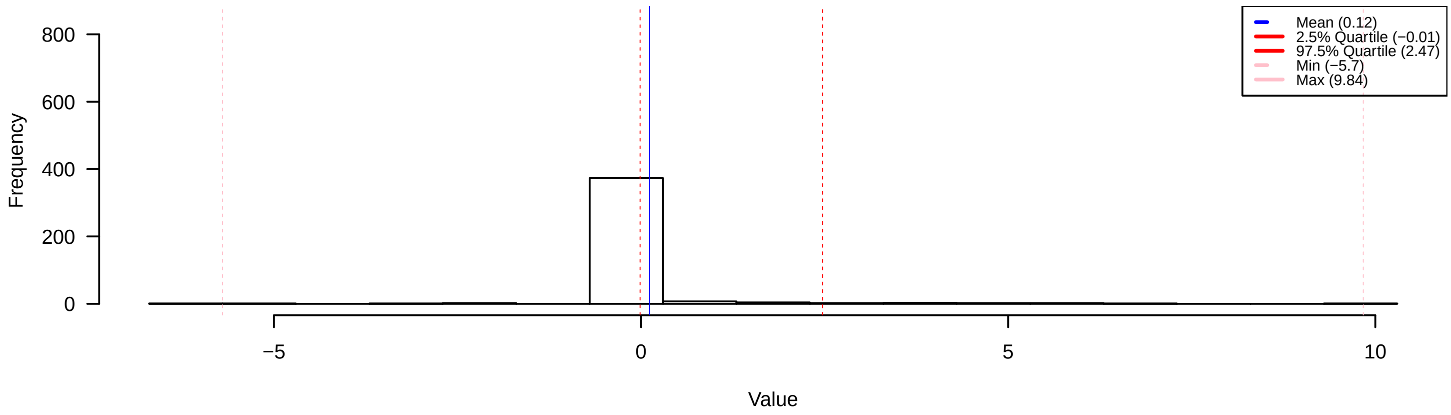

S5, Figure 280 : Bootstrap Distribution of Air Temperature Squared lag 19

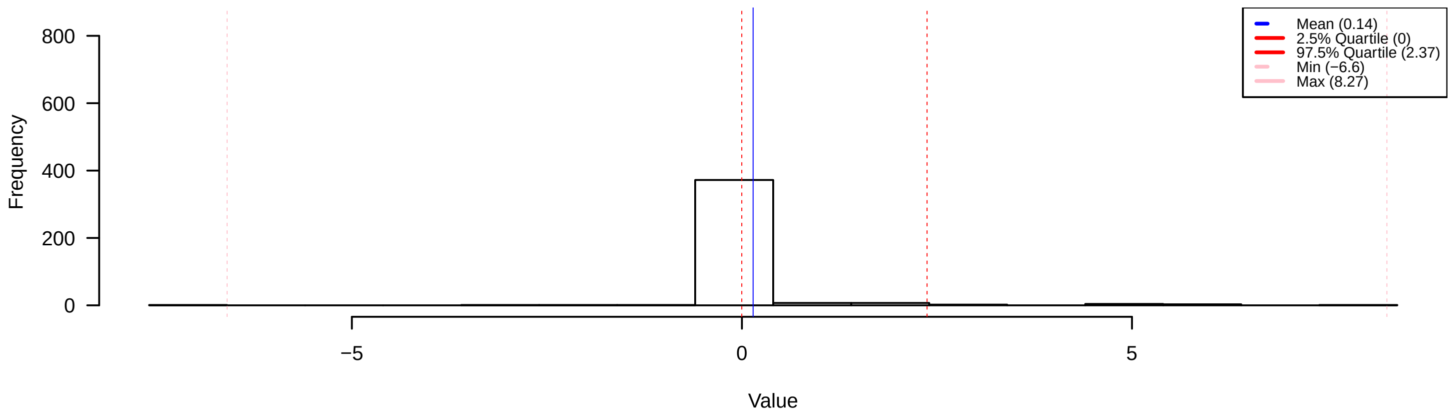

S5, Figure 281 : Bootstrap Distribution of Air Temperature Squared lag 20

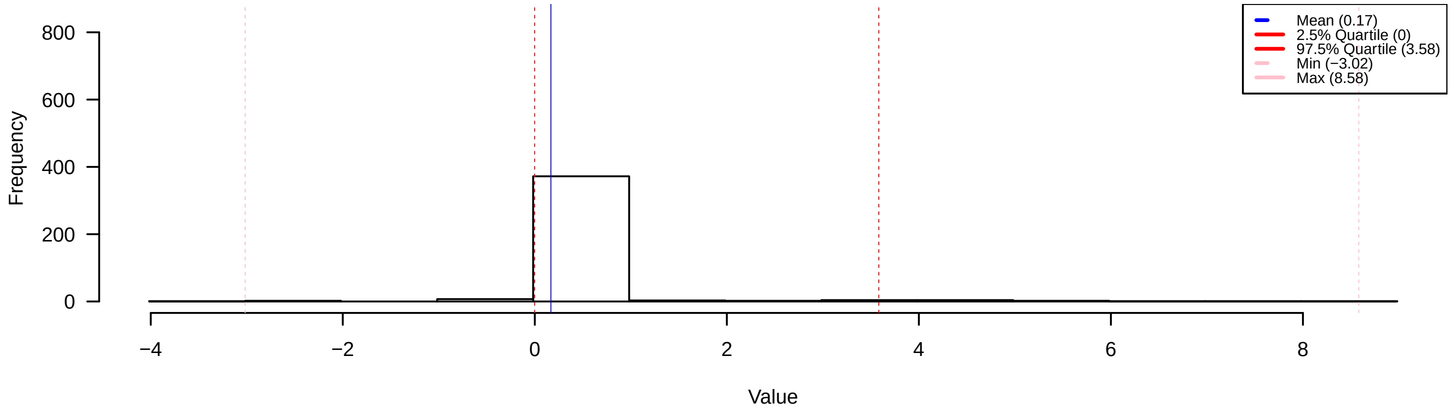

S5, Figure 282 : Bootstrap Distribution of Absolute Humidity Squared lag 1

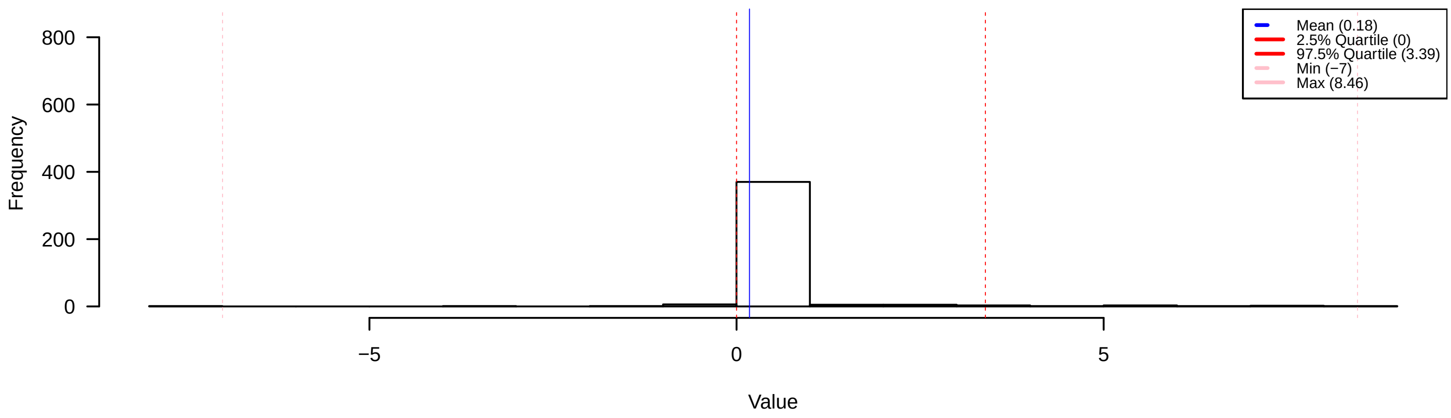

S5, Figure 283 : Bootstrap Distribution of Absolute Humidity Squared lag 2

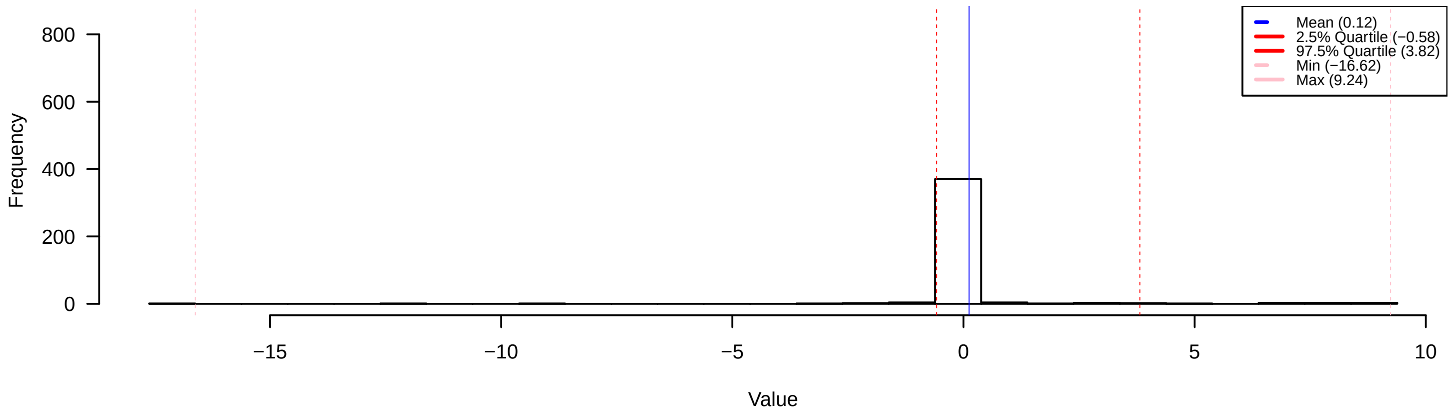

S5, Figure 284 : Bootstrap Distribution of Absolute Humidity Squared lag 3

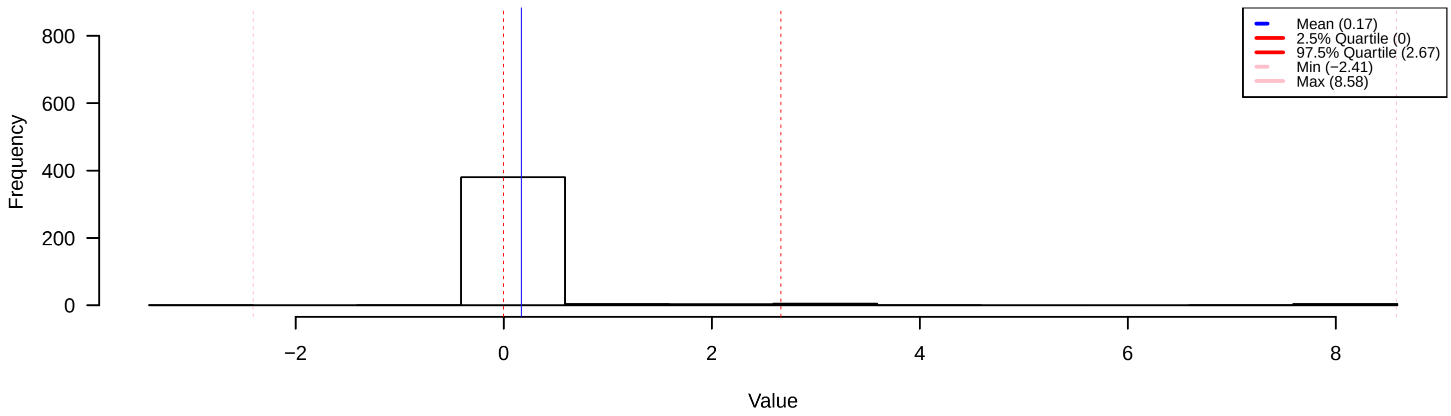

S5, Figure 285 : Bootstrap Distribution of Absolute Humidity Squared lag 4

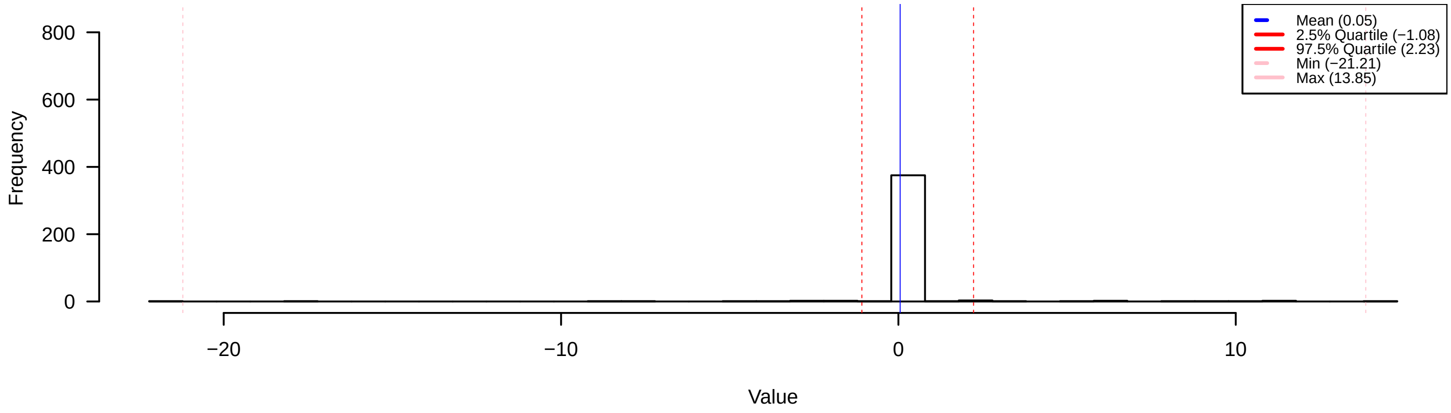

S5, Figure 286 : Bootstrap Distribution of Absolute Humidity Squared lag 5

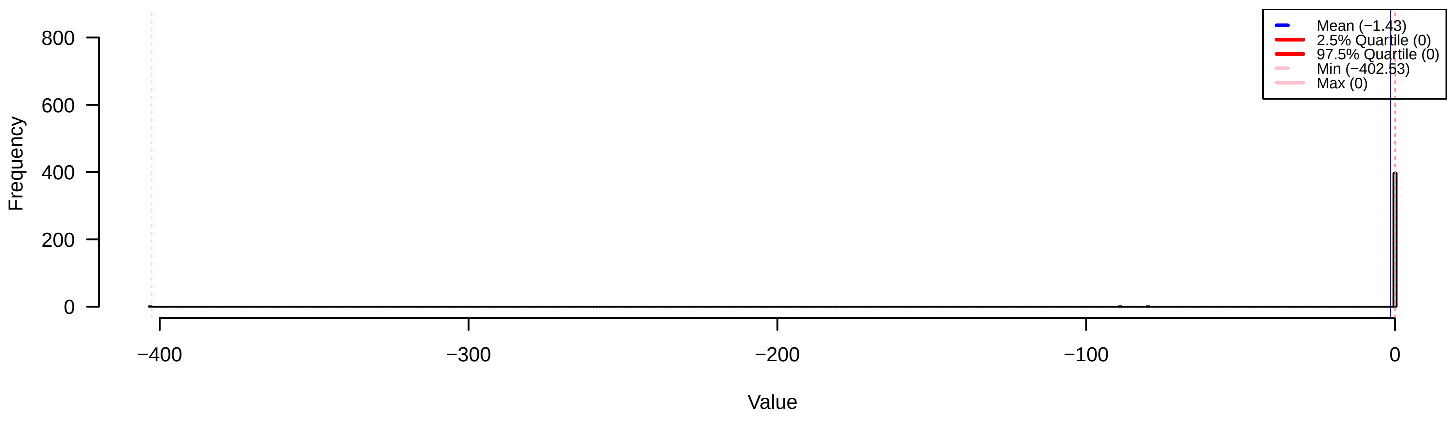

S5, Figure 287 : Bootstrap Distribution of Absolute Humidity Squared lag 6

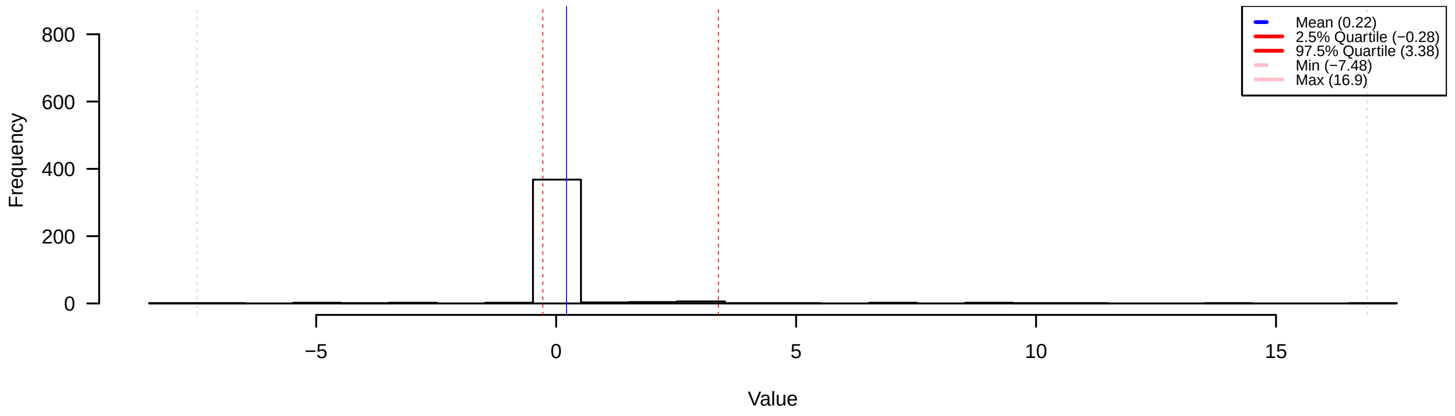

S5, Figure 288 : Bootstrap Distribution of Absolute Humidity Squared lag 7

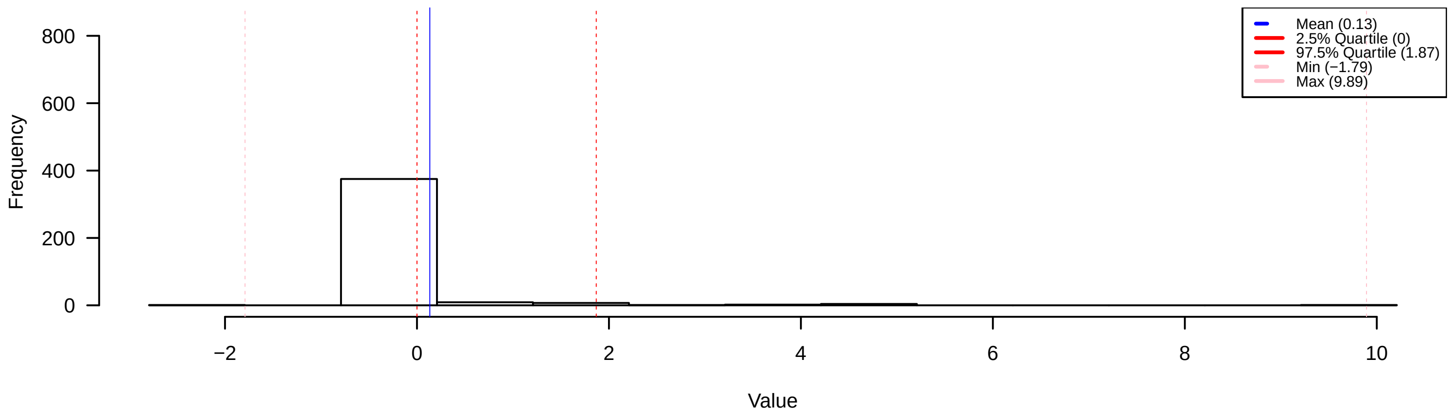

S5, Figure 289 : Bootstrap Distribution of Absolute Humidity Squared lag 8

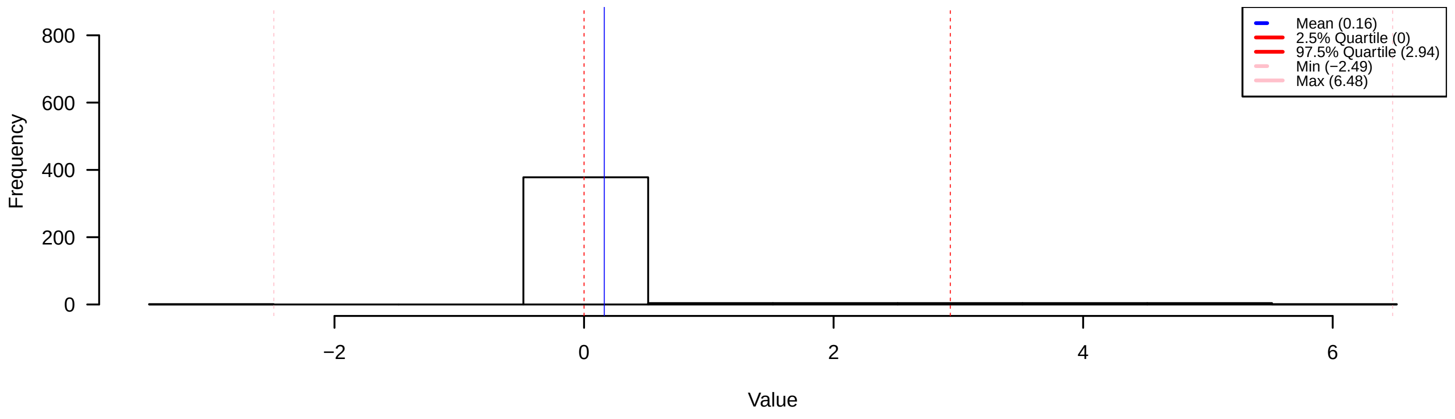

S5, Figure 290 : Bootstrap Distribution of Absolute Humidity Squared lag 9

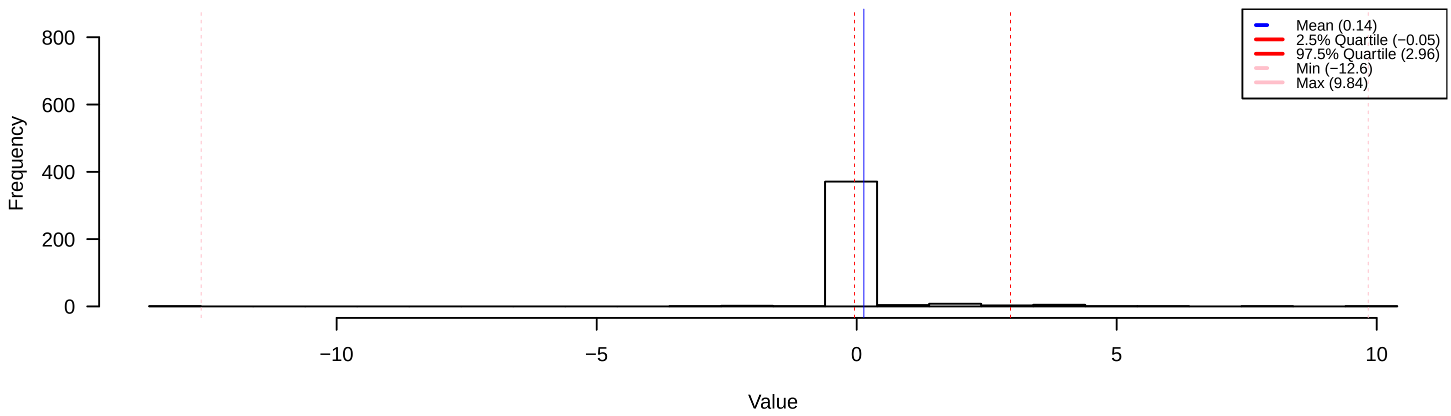

S5, Figure 291 : Bootstrap Distribution of Absolute Humidity Squared lag 10

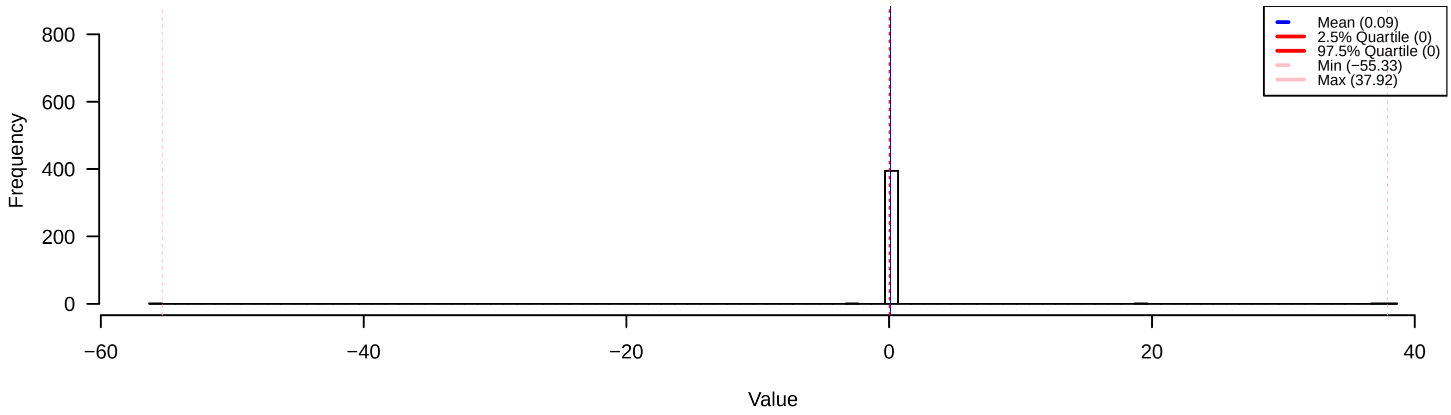

S5, Figure 292 : Bootstrap Distribution of Absolute Humidity Squared lag 11

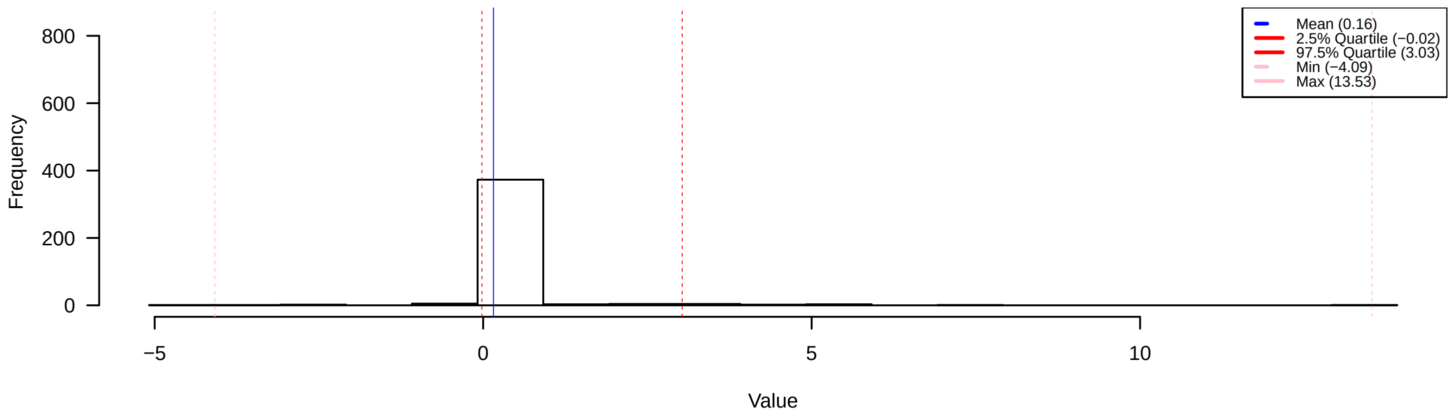

S5, Figure 293 : Bootstrap Distribution of Absolute Humidity Squared lag 12

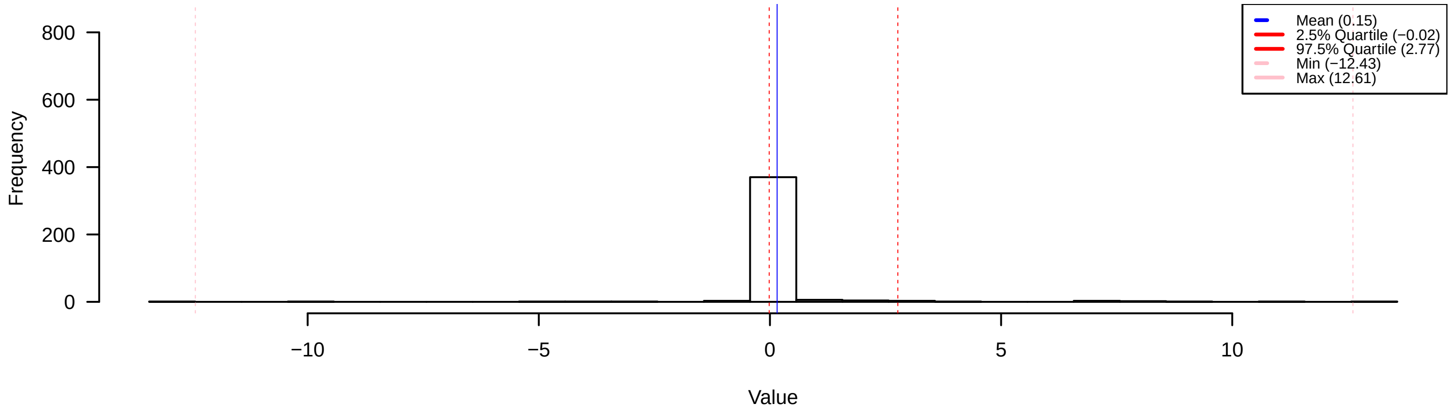

S5, Figure 294 : Bootstrap Distribution of Absolute Humidity Squared lag 13

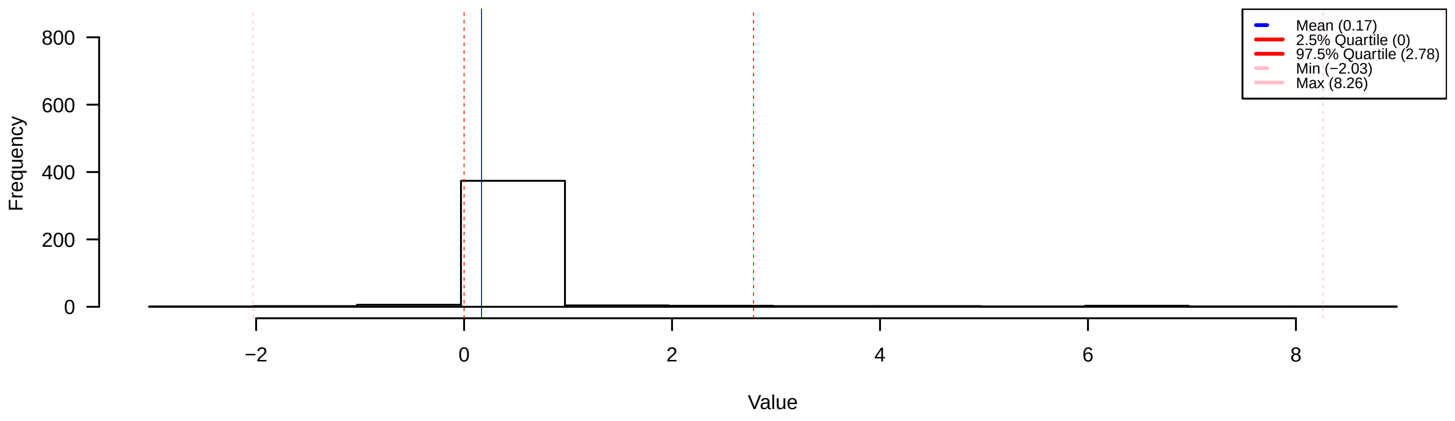

S5, Figure 295 : Bootstrap Distribution of Absolute Humidity Squared lag 14

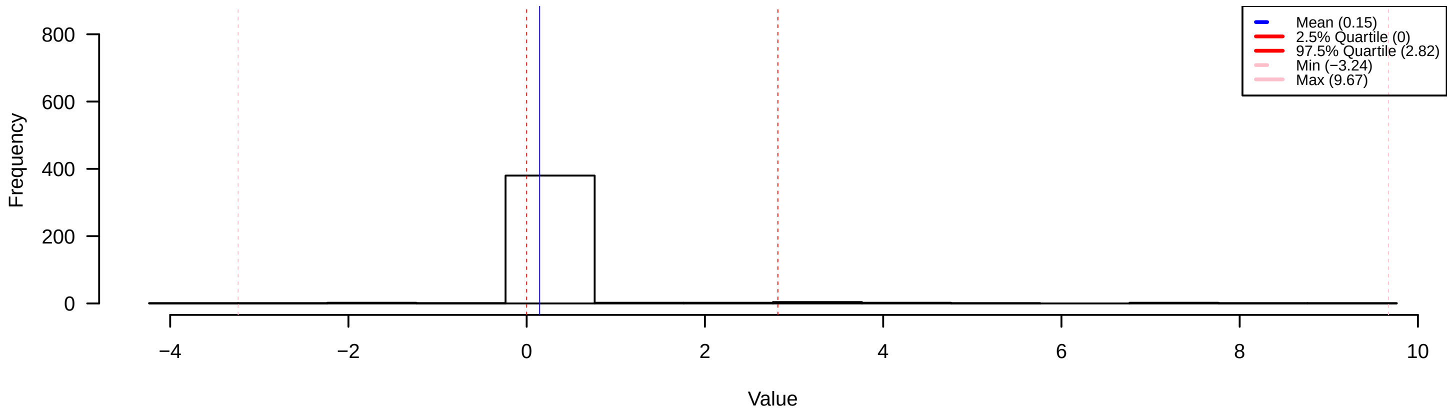

S5, Figure 296 : Bootstrap Distribution of Absolute Humidity Squared lag 15

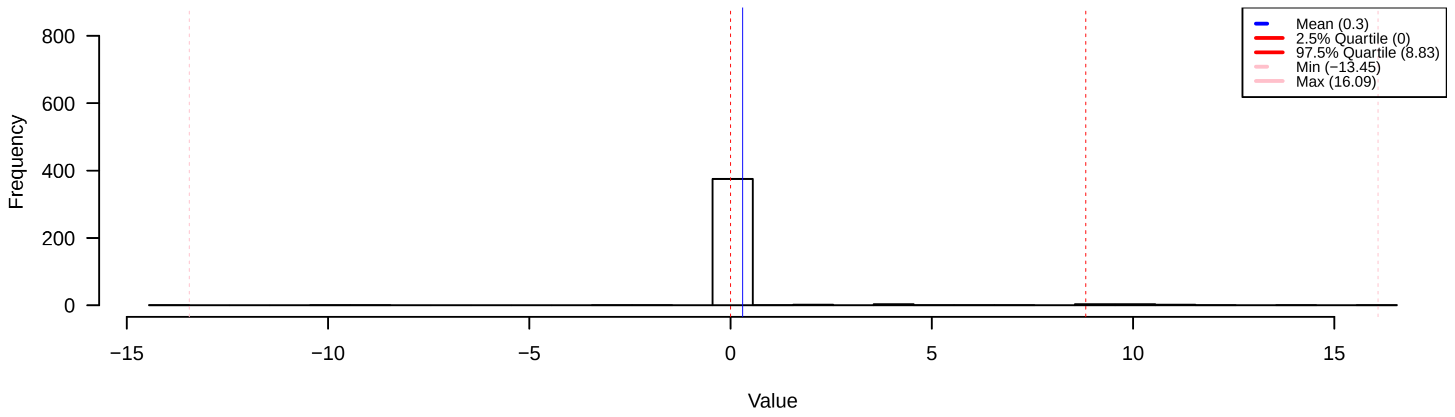

S5, Figure 297 : Bootstrap Distribution of Absolute Humidity Squared lag 16

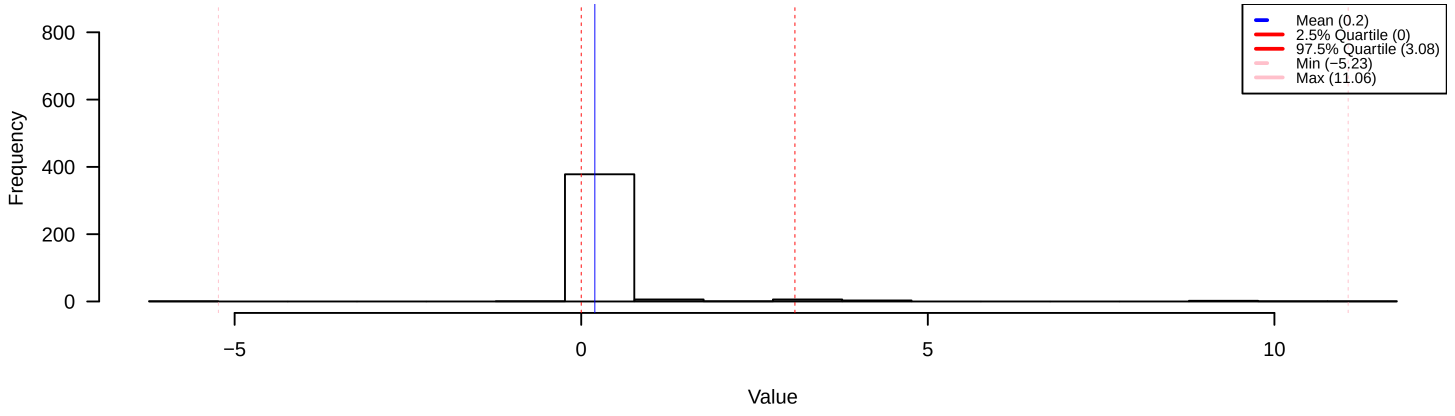

S5, Figure 298 : Bootstrap Distribution of Absolute Humidity Squared lag 17

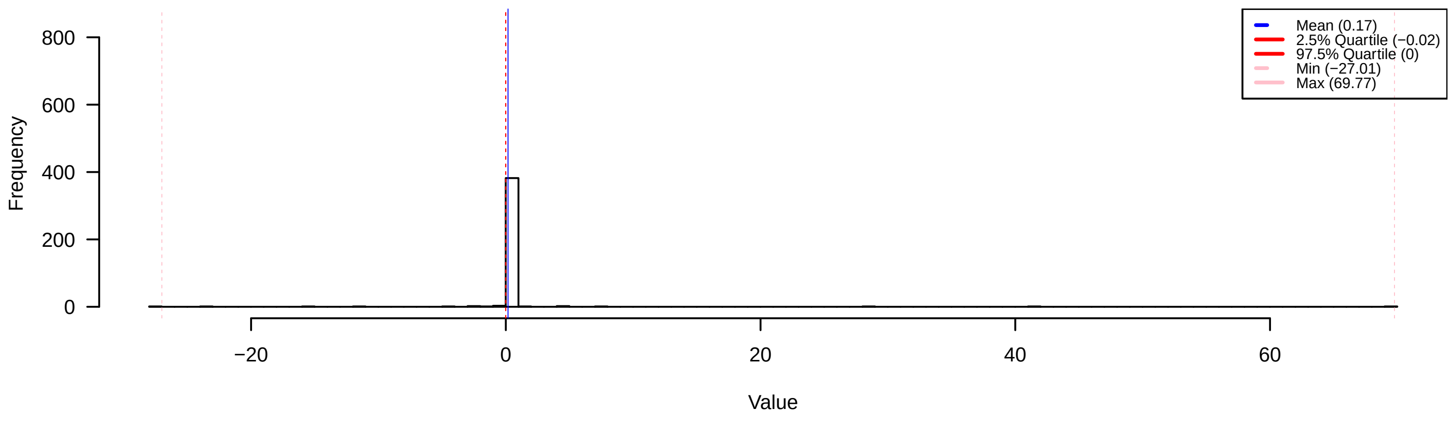

S5, Figure 299 : Bootstrap Distribution of Absolute Humidity Squared lag 18

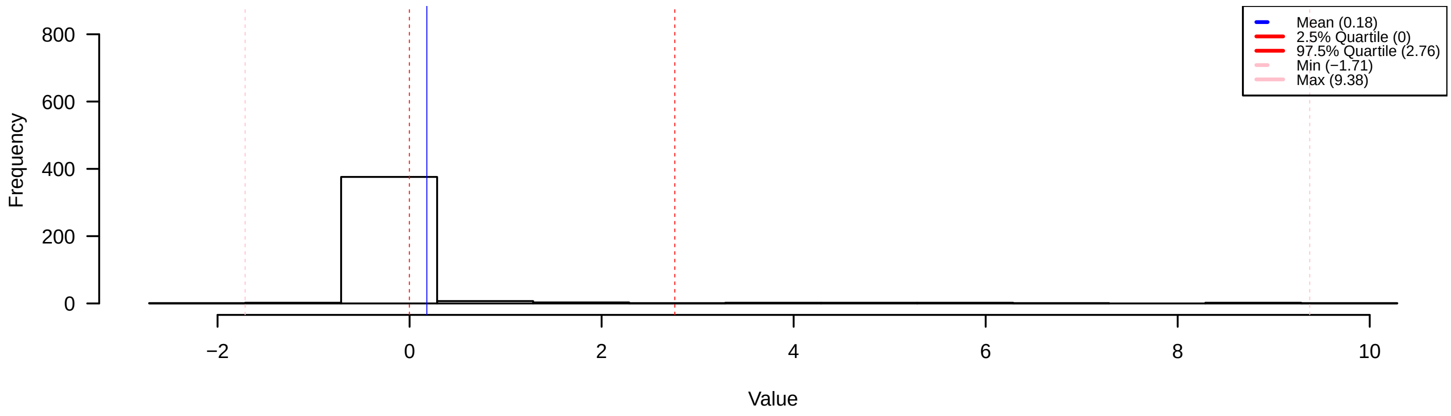

S5, Figure 300 : Bootstrap Distribution of Absolute Humidity Squared lag 19

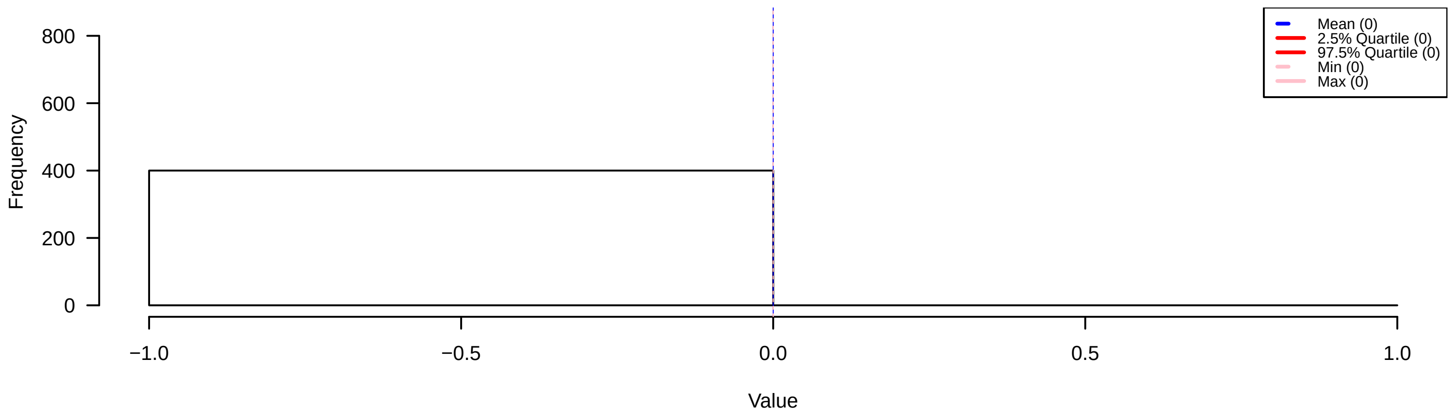

S5, Figure 301 : Bootstrap Distribution of Absolute Humidity Squared lag 20

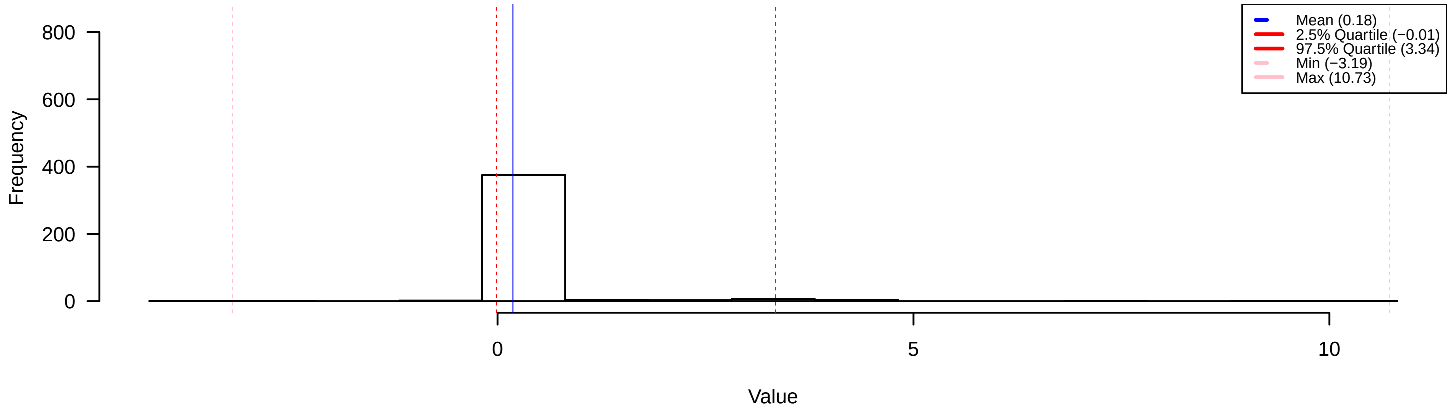

S5, Figure 302 : Bootstrap Distribution of Relative Humidity Squared lag 1

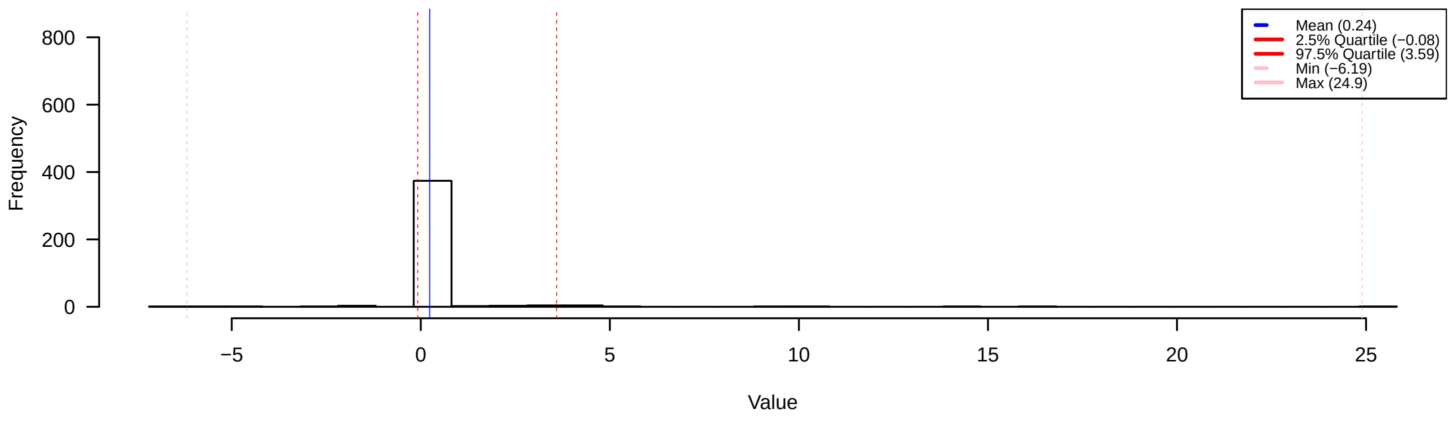

S5, Figure 303 : Bootstrap Distribution of Relative Humidity Squared lag 2

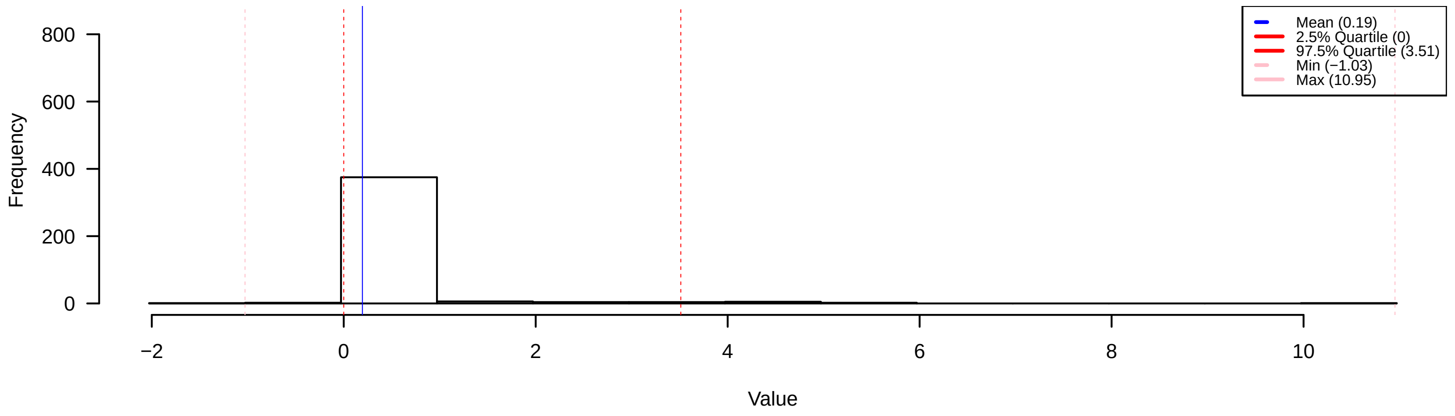

S5, Figure 304 : Bootstrap Distribution of Relative Humidity Squared lag 3

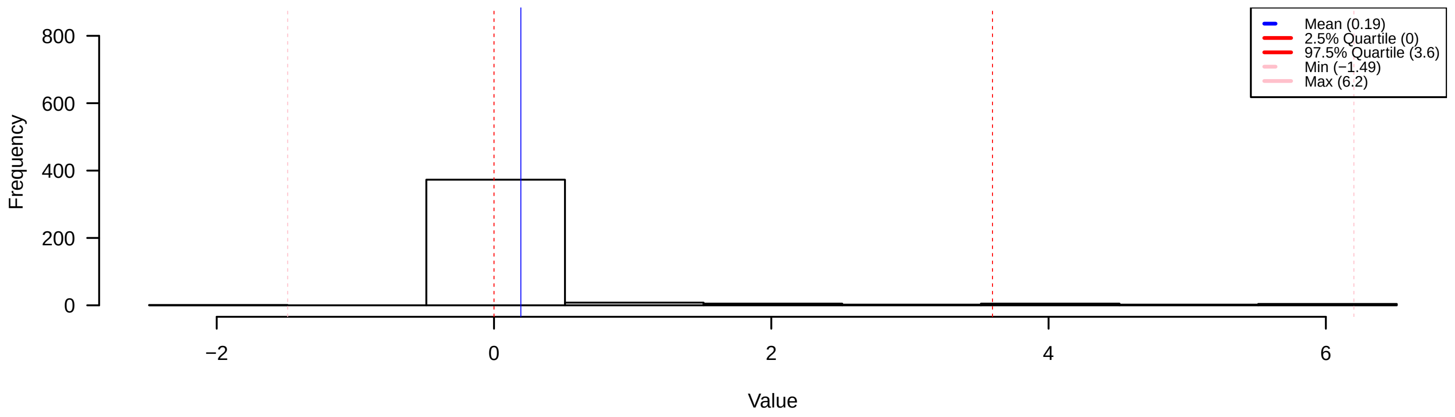

S5, Figure 305 : Bootstrap Distribution of Relative Humidity Squared lag 4

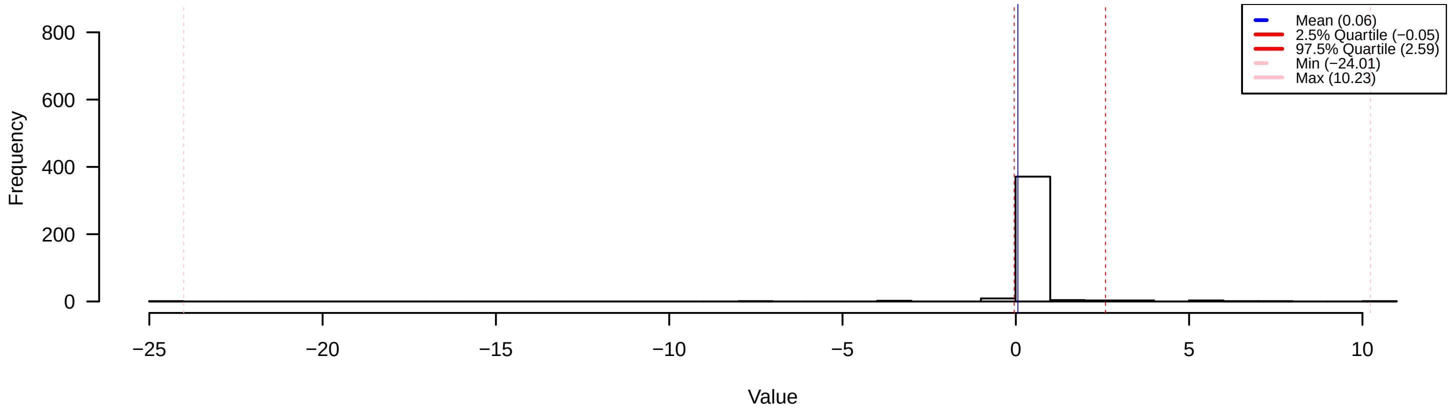

S5, Figure 306 : Bootstrap Distribution of Relative Humidity Squared lag 5

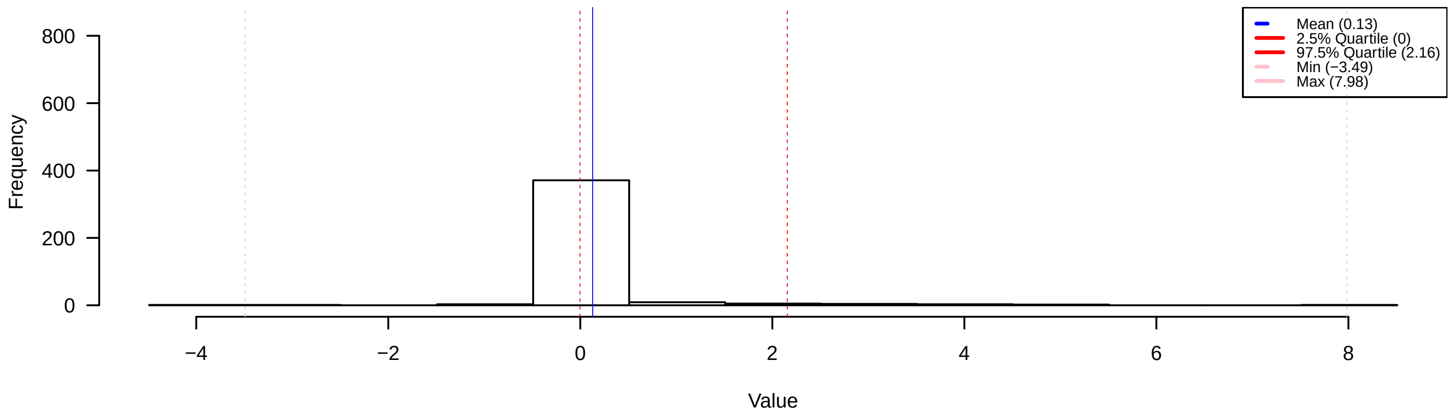

S5, Figure 307 : Bootstrap Distribution of Relative Humidity Squared lag 6

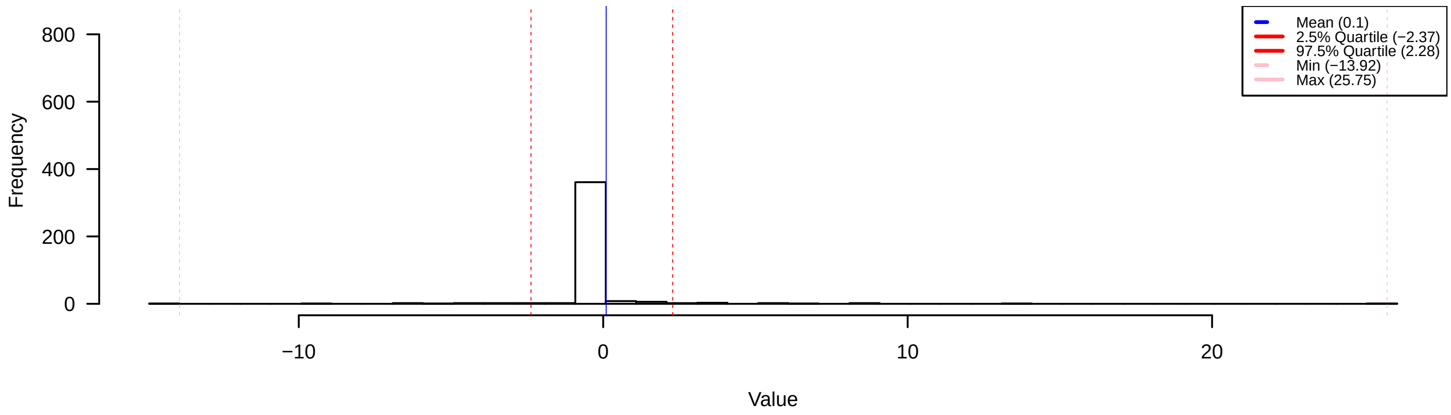

S5, Figure 308 : Bootstrap Distribution of Relative Humidity Squared lag 7

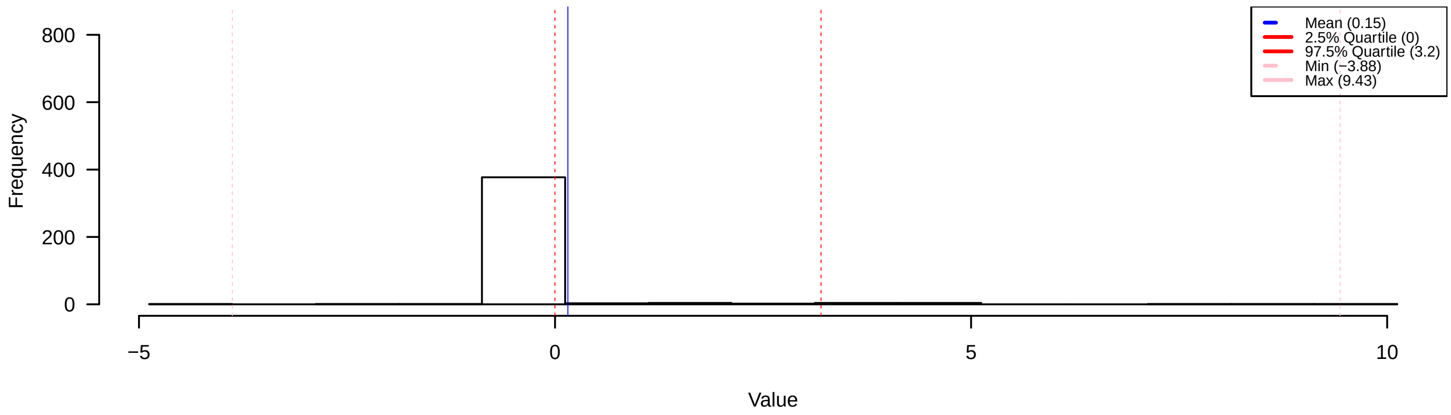

S5, Figure 309 : Bootstrap Distribution of Relative Humidity Squared lag 8

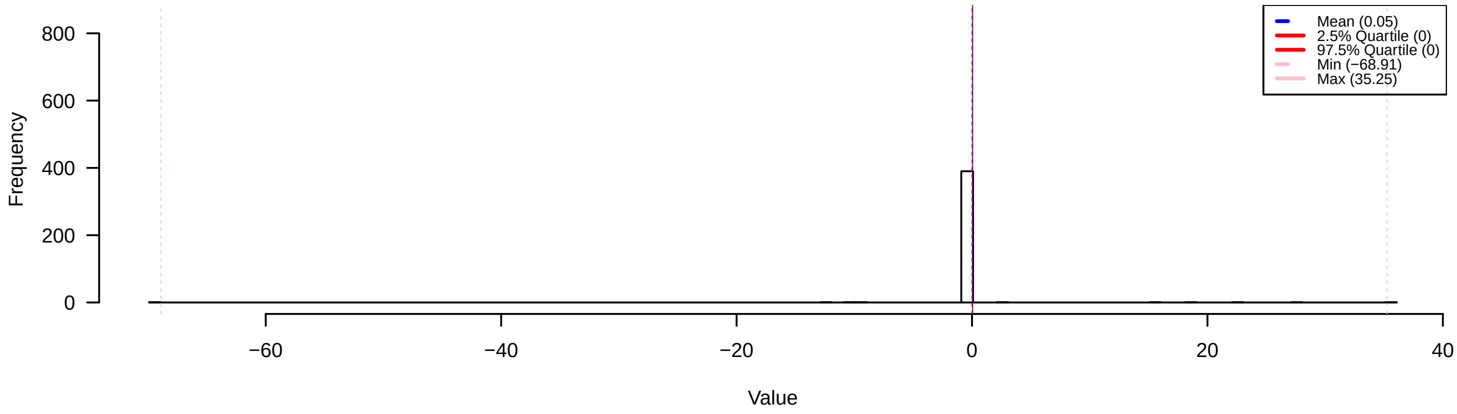

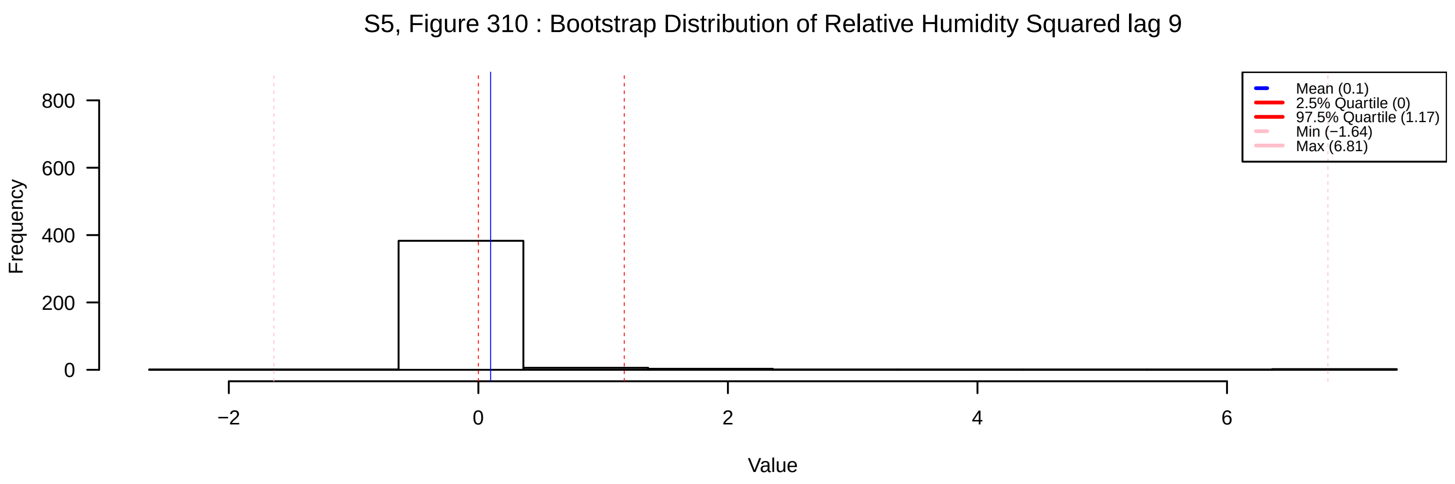
S5, Figure 310 : Bootstrap Distribution of Relative Humidity Squared lag 9
Mean (0.1)
2.5% Quartile (0)
97.5% Quartile (1.17)
Min (−1.64)
Max (6.81)
Frequency
Value

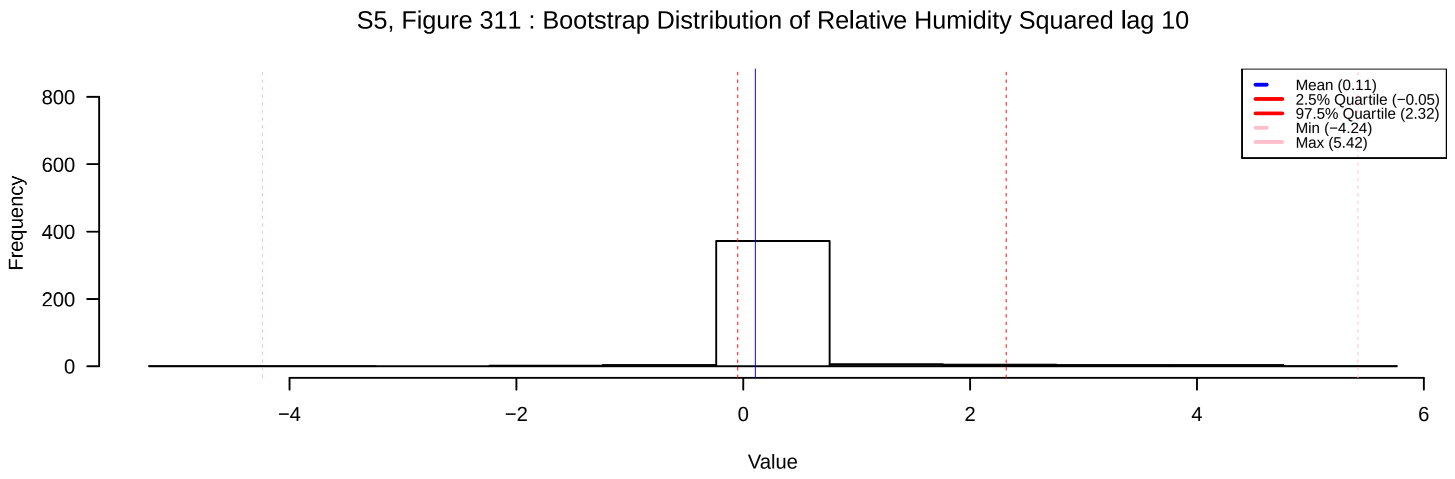
S5, Figure 311 : Bootstrap Distribution of Relative Humidity Squared lag 10
Mean (0.11)
2.5% Quartile (−0.05)
97.5% Quartile (2.32)
Min (−4.24)
Max (5.42)
Frequency
Value

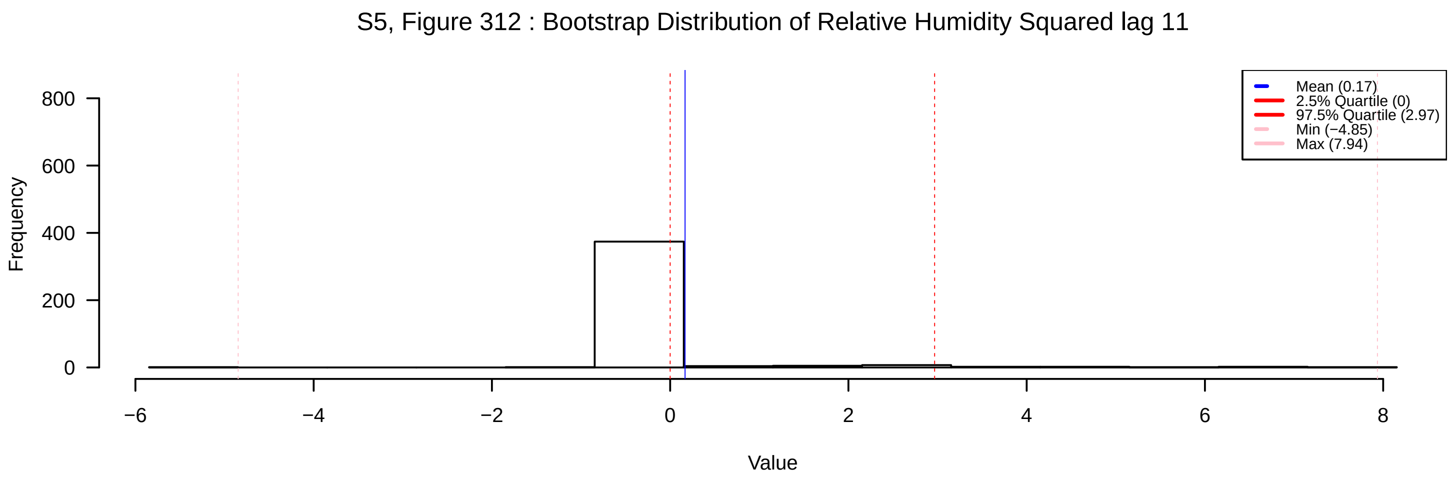
S5, Figure 312 : Bootstrap Distribution of Relative Humidity Squared lag 11
Mean (0.17)
2.5% Quartile (0)
97.5% Quartile (2.97)
Min (−4.85)
Max (7.94)
Frequency
Value

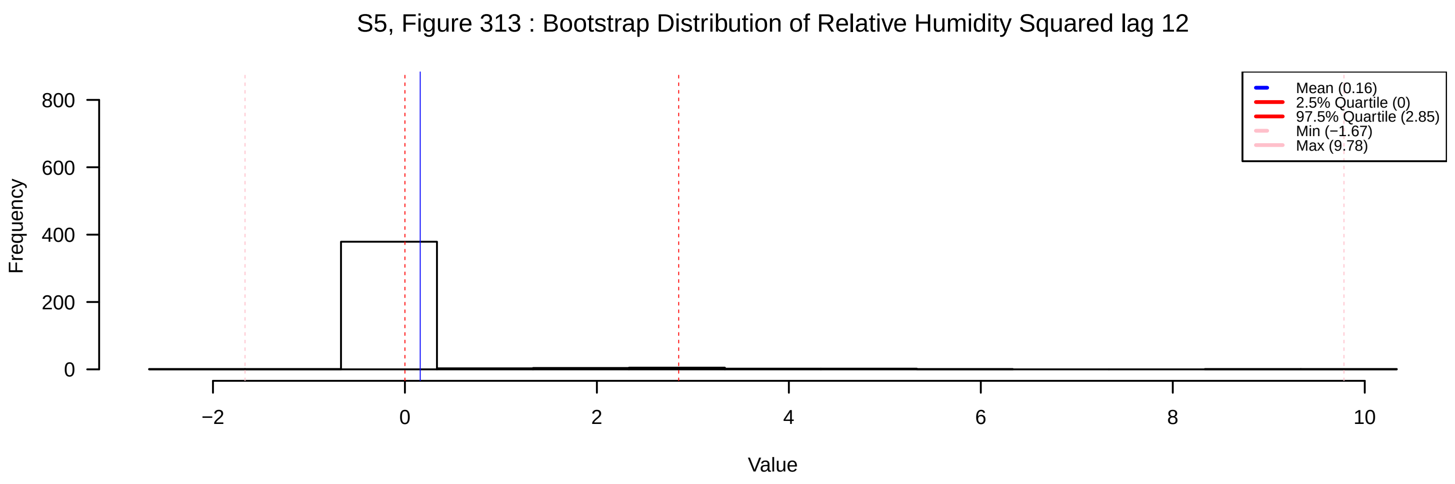
S5, Figure 313 : Bootstrap Distribution of Relative Humidity Squared lag 12
Mean (0.16)
2.5% Quartile (0)
97.5% Quartile (2.85)
Min (−1.67)
Max (9.78)
Frequency
Value

S5, Figure 314 : Bootstrap Distribution of Relative Humidity Squared lag 13

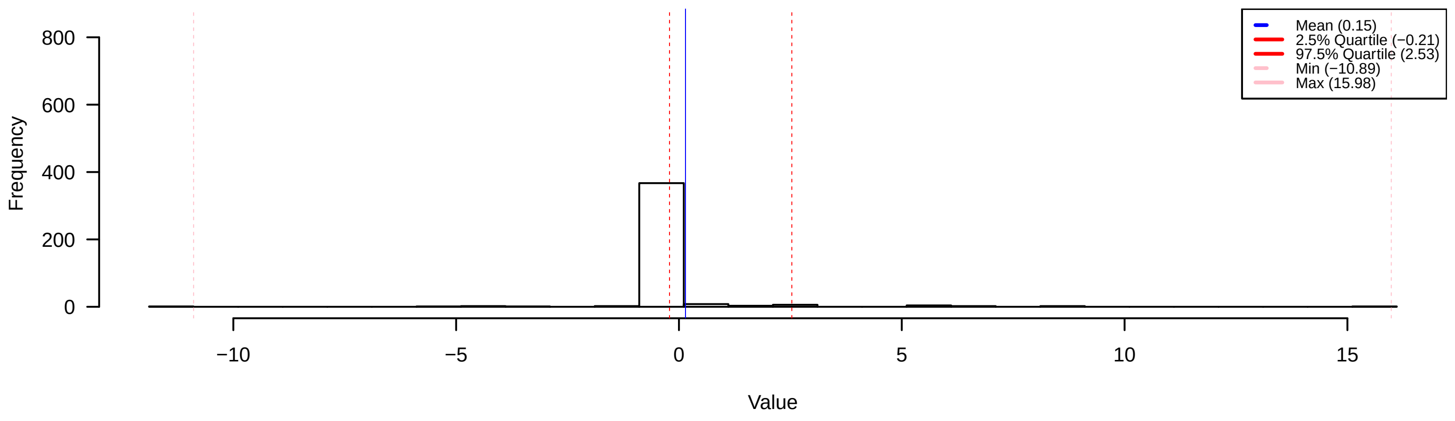

S5, Figure 315 : Bootstrap Distribution of Relative Humidity Squared lag 14

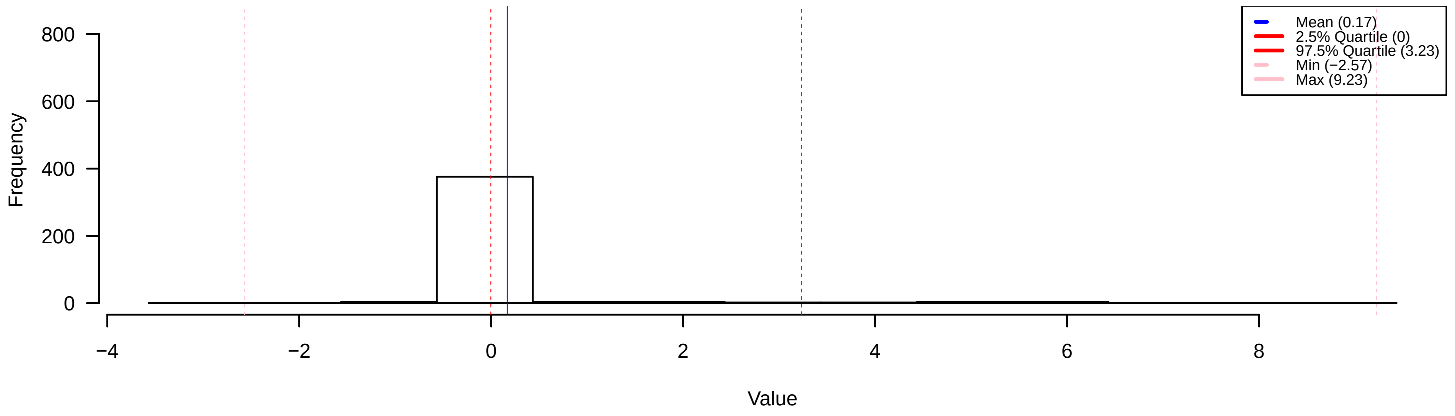

S5, Figure 316 : Bootstrap Distribution of Relative Humidity Squared lag 15

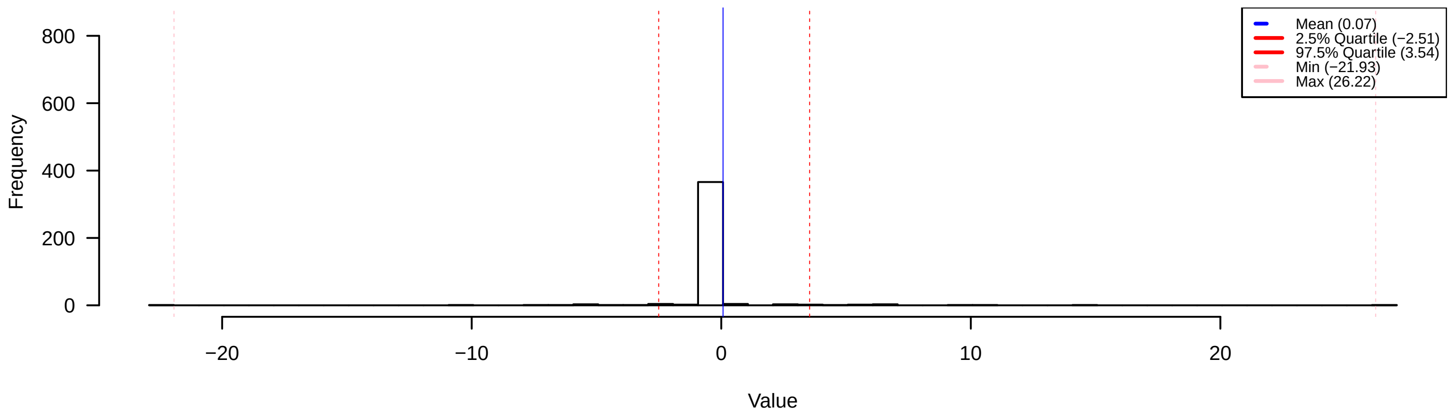

S5, Figure 317 : Bootstrap Distribution of Relative Humidity Squared lag 16

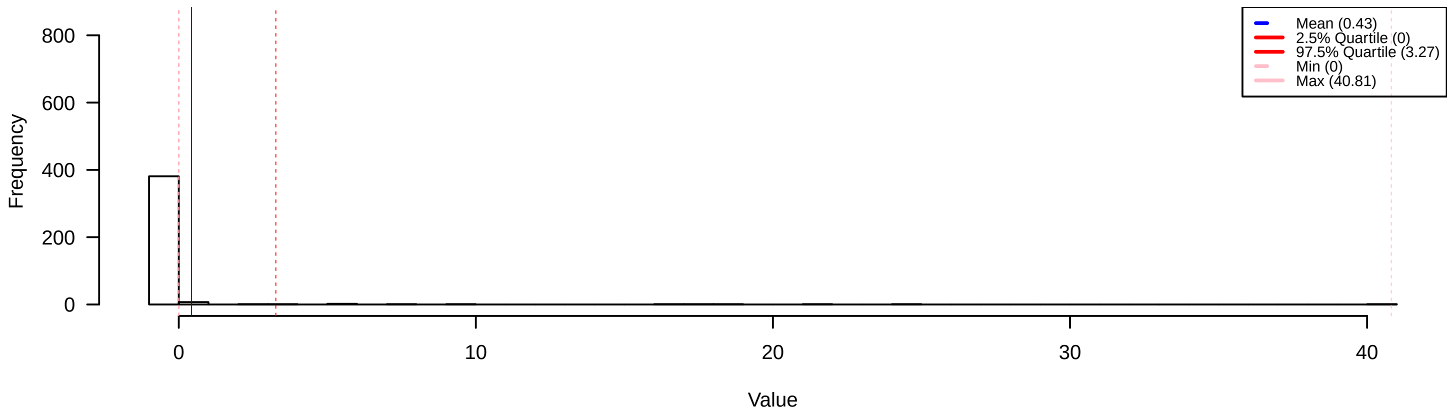

S5, Figure 318 : Bootstrap Distribution of Relative Humidity Squared lag 17

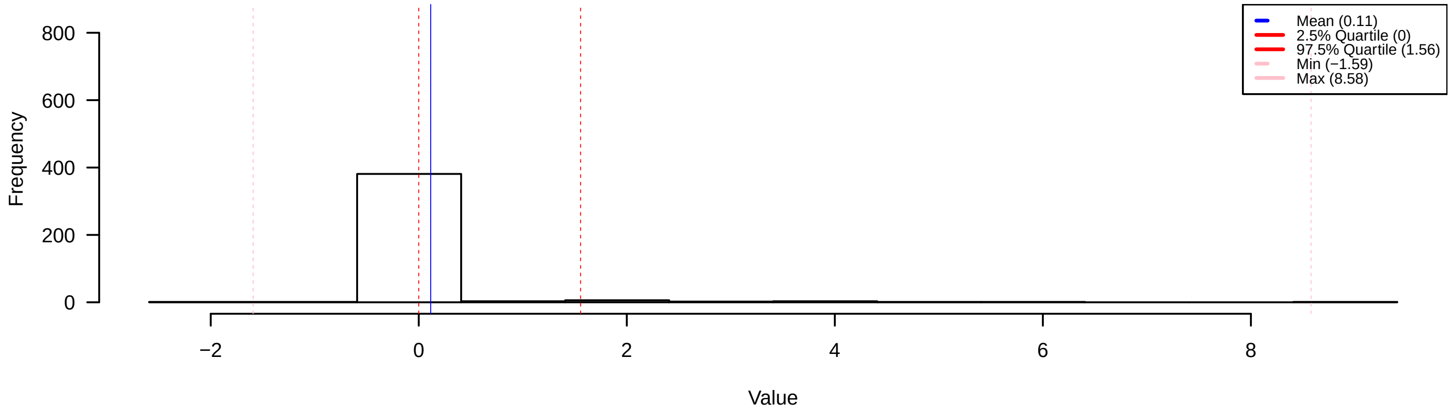

S5, Figure 319 : Bootstrap Distribution of Relative Humidity Squared lag 18

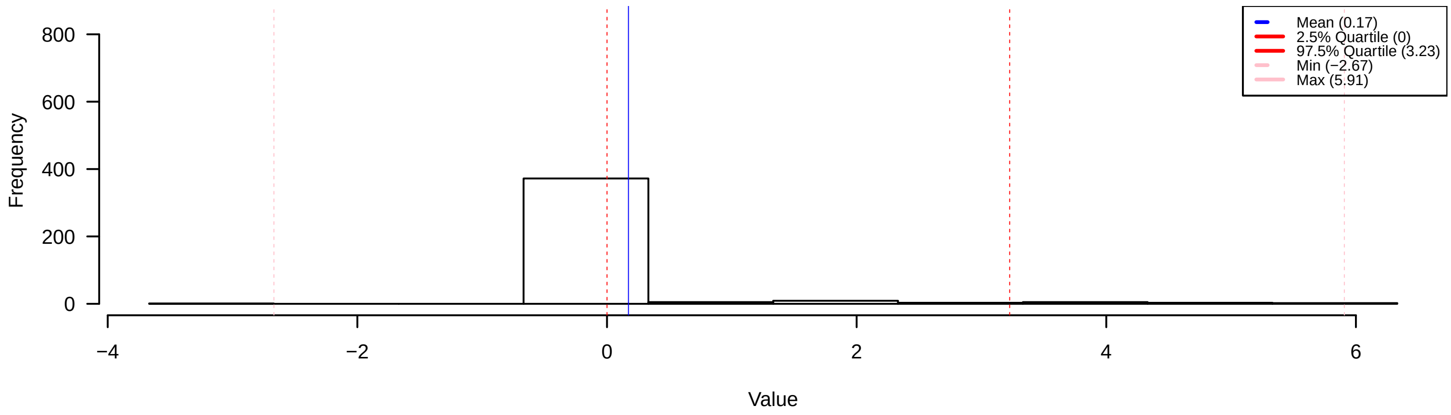

S5, Figure 320 : Bootstrap Distribution of Relative Humidity Squared lag 19

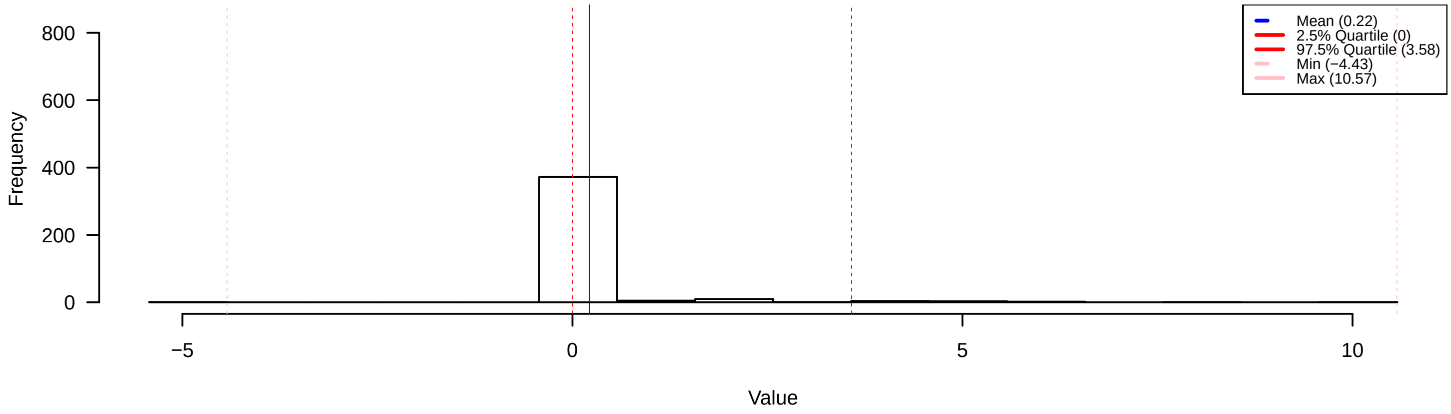

S5, Figure 321 : Bootstrap Distribution of Relative Humidity Squared lag 20

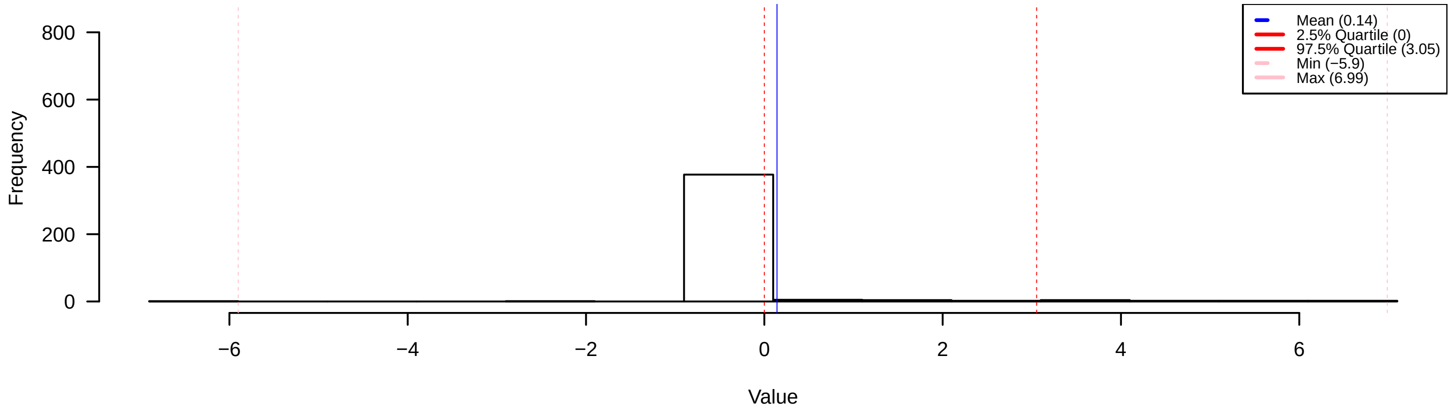

S5, Figure 322 : Bootstrap Distribution of Dewpoint Temperature:Air Temperature Squared lag 1

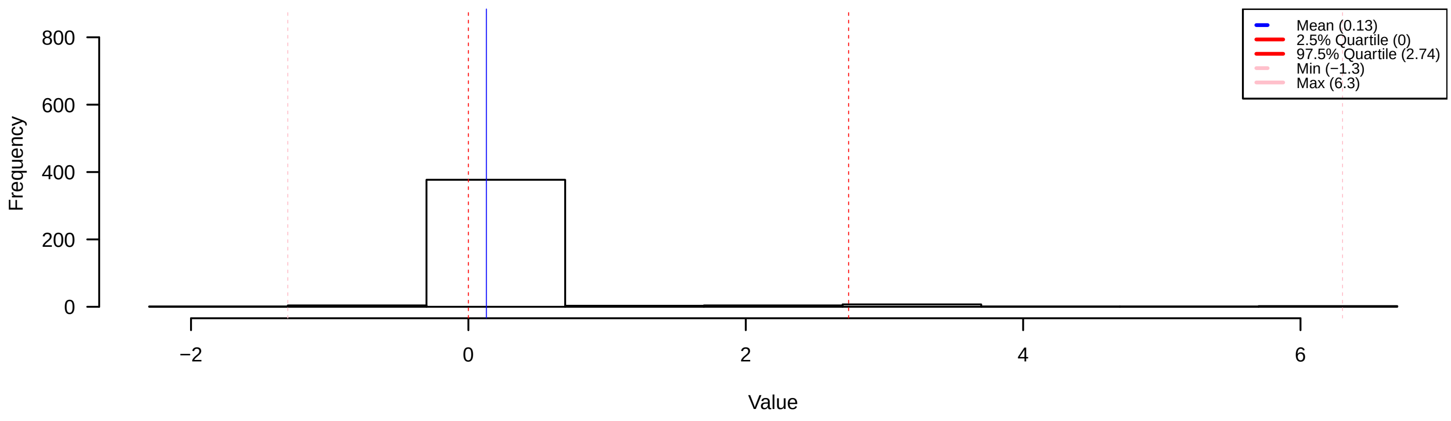

S5, Figure 323 : Bootstrap Distribution of Dewpoint Temperature:Air Temperature Squared lag 2

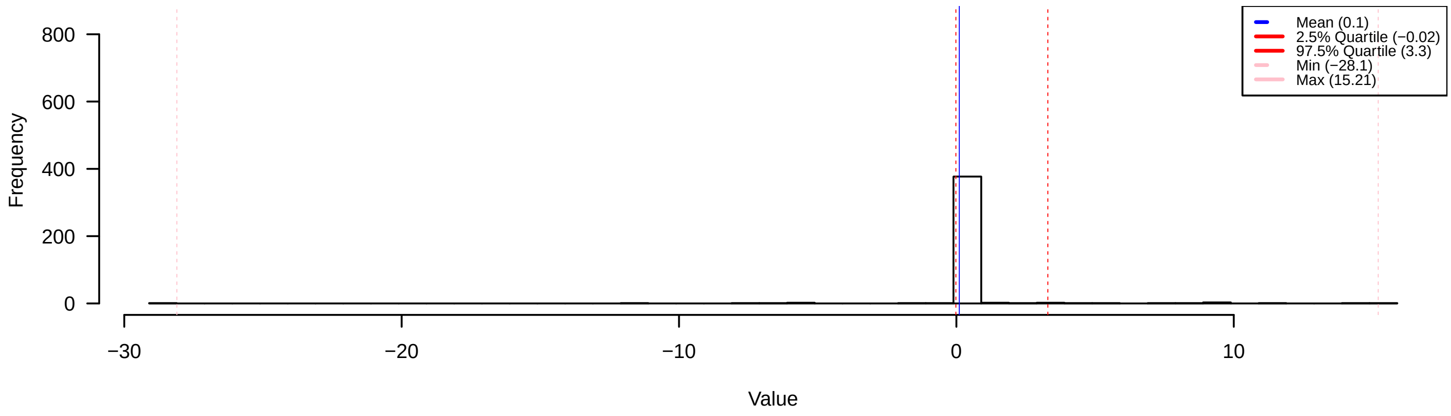

S5, Figure 324 : Bootstrap Distribution of Dewpoint Temperature:Air Temperature Squared lag 3

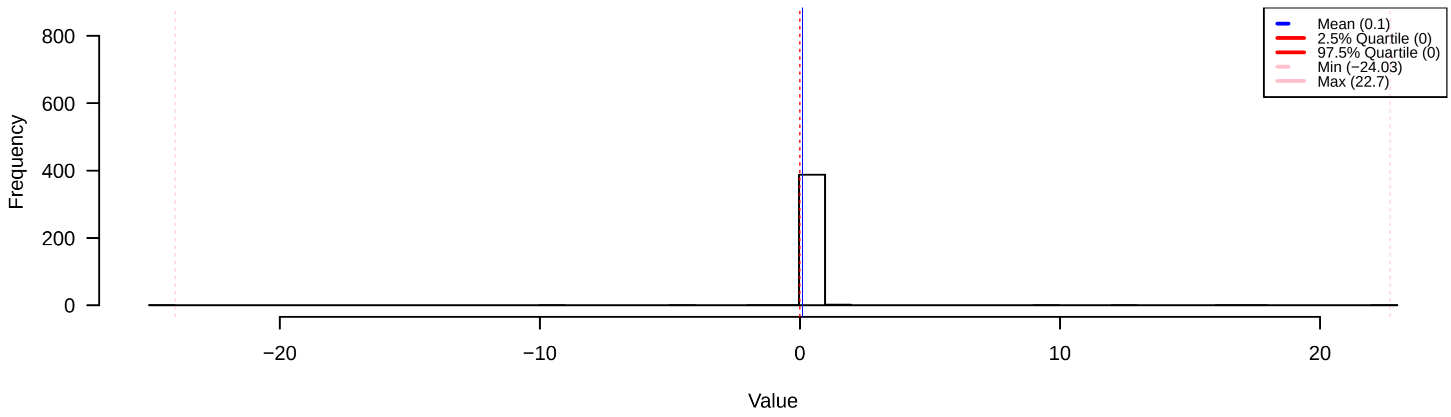

S5, Figure 325 : Bootstrap Distribution of Dewpoint Temperature:Air Temperature Squared lag 4

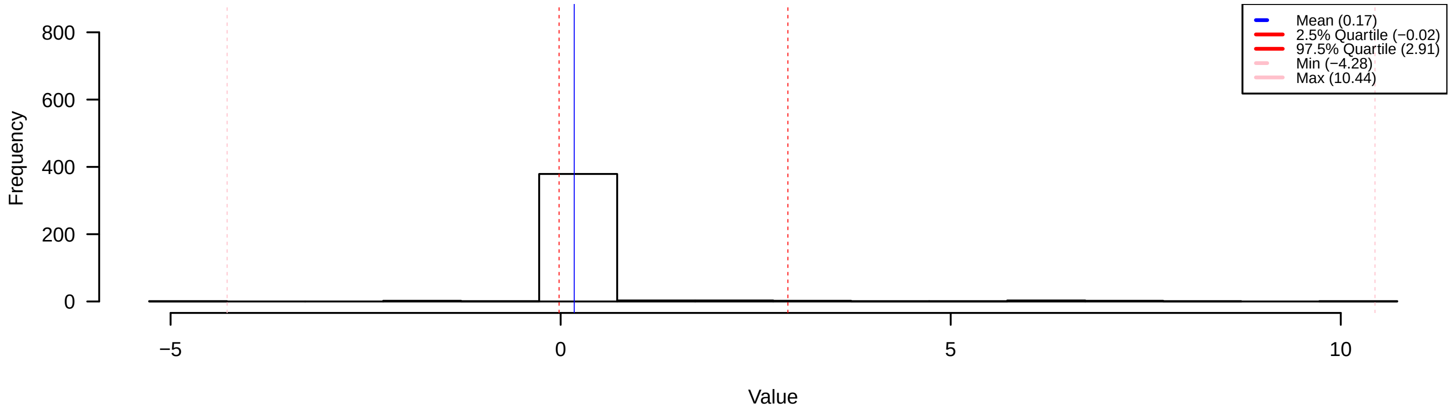

S5, Figure 326 : Bootstrap Distribution of Dewpoint Temperature:Air Temperature Squared lag 5

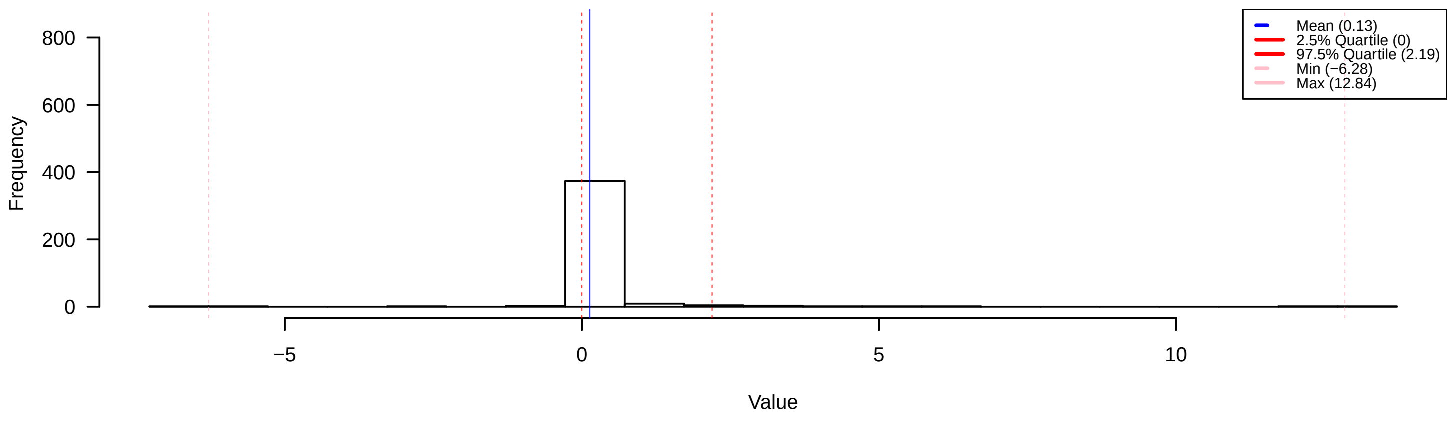

S5, Figure 327 : Bootstrap Distribution of Dewpoint Temperature:Air Temperature Squared lag 6

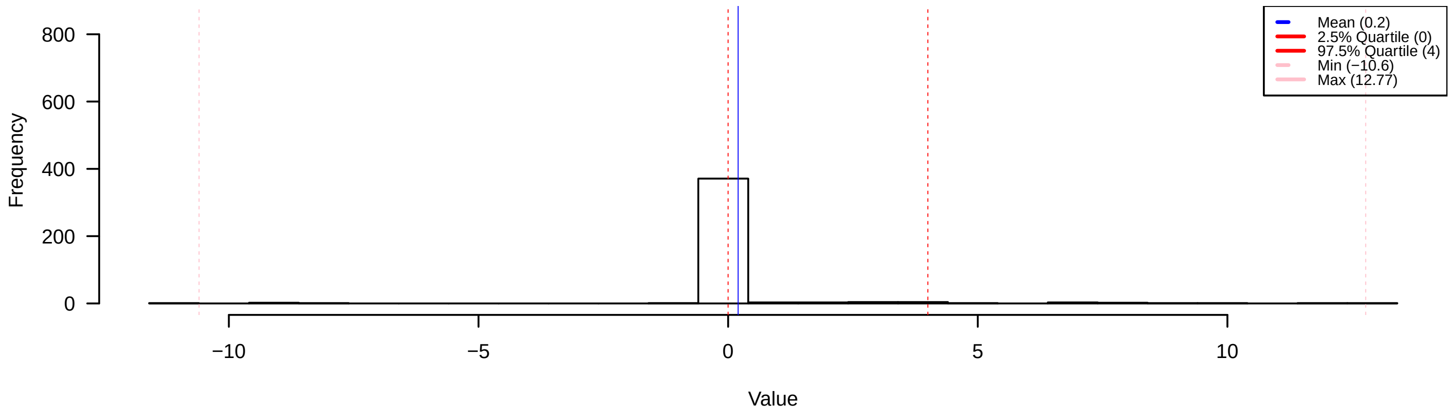

S5, Figure 328 : Bootstrap Distribution of Dewpoint Temperature:Air Temperature Squared lag 7

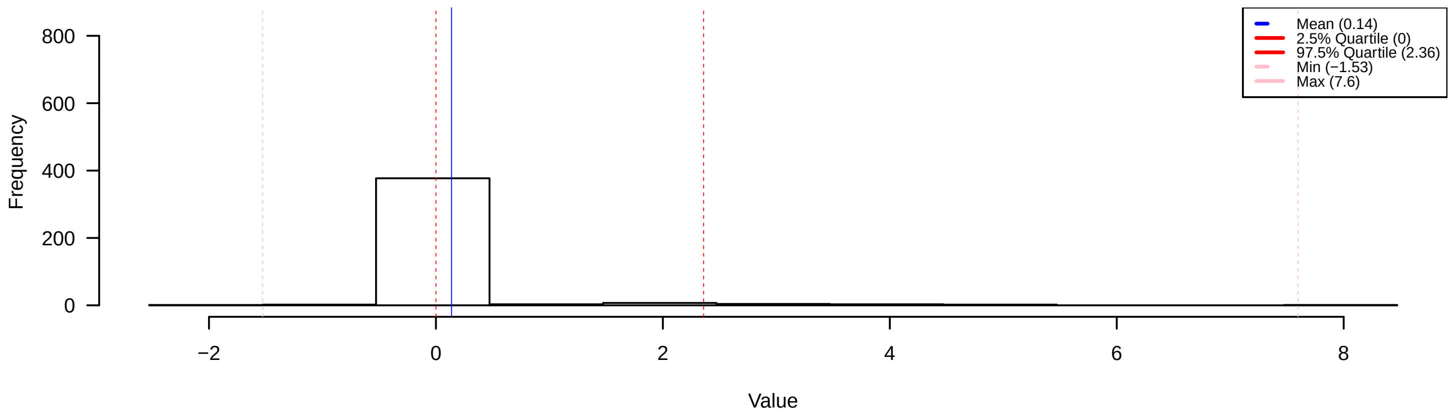

S5, Figure 329 : Bootstrap Distribution of Dewpoint Temperature:Air Temperature Squared lag 8

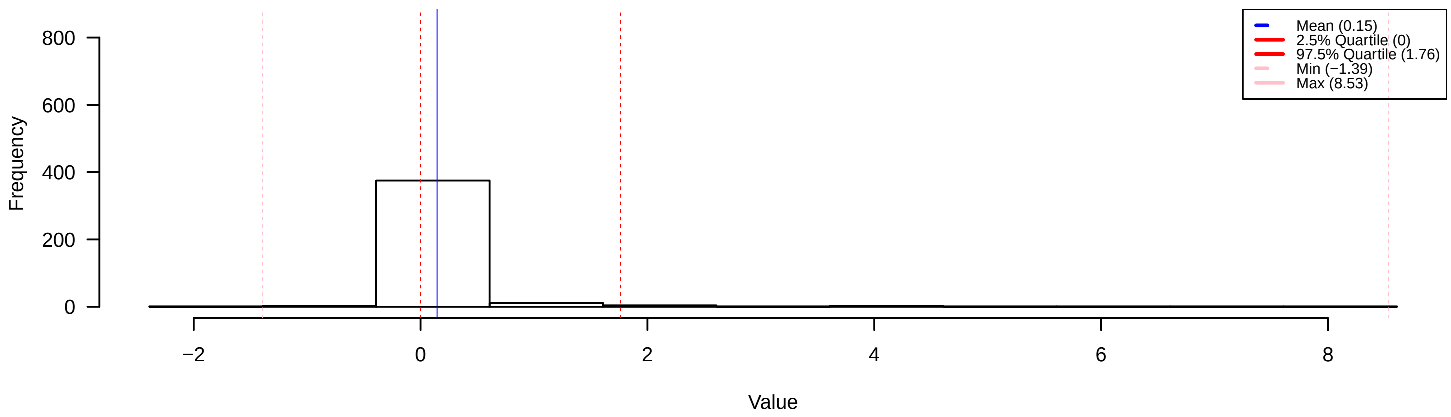

S5, Figure 330 : Bootstrap Distribution of Dewpoint Temperature:Air Temperature Squared lag 9

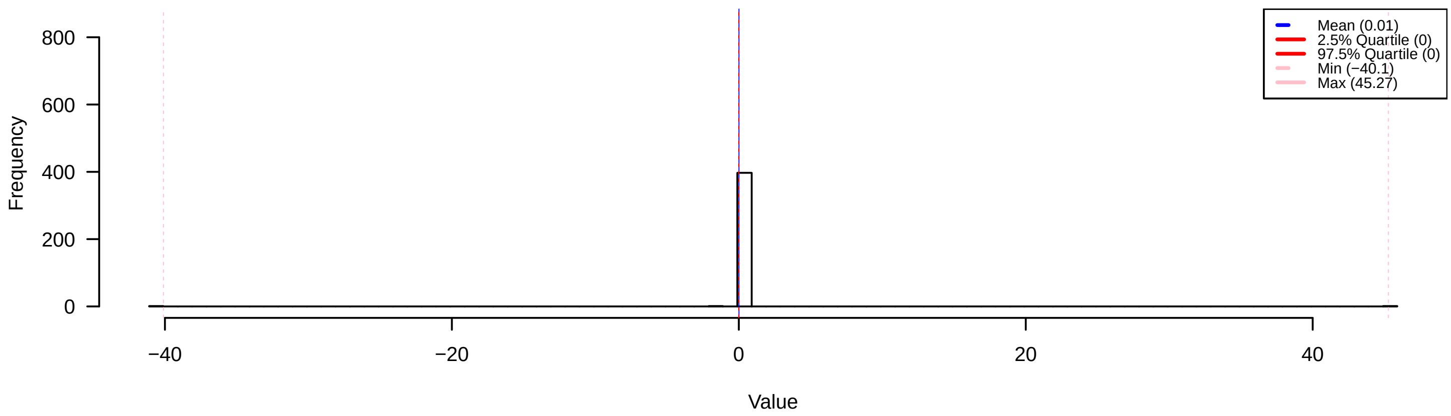

S5, Figure 331 : Bootstrap Distribution of Dewpoint Temperature:Air Temperature Squared lag 10

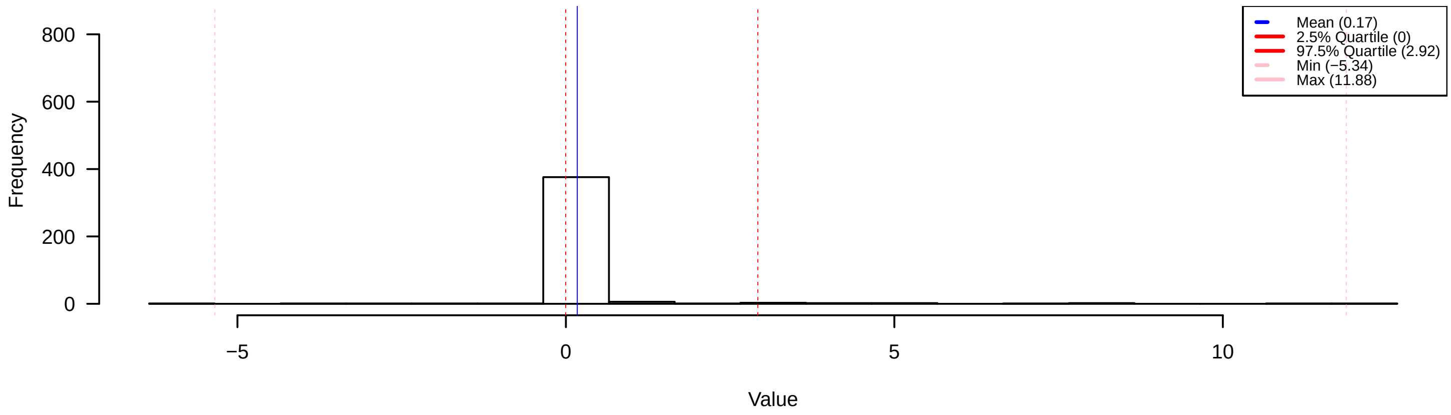

S5, Figure 332 : Bootstrap Distribution of Dewpoint Temperature:Air Temperature Squared lag 11

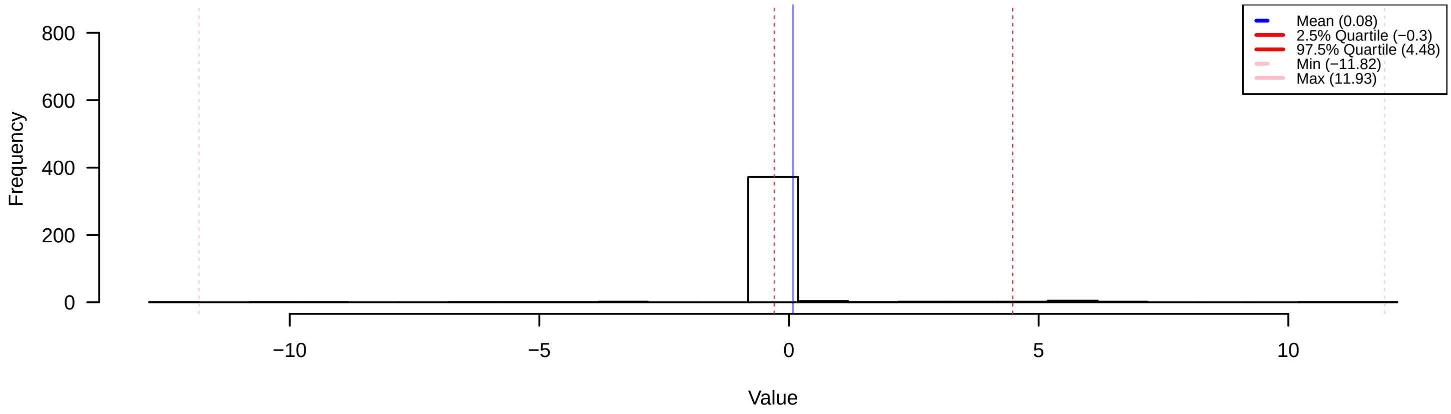

S5, Figure 333 : Bootstrap Distribution of Dewpoint Temperature:Air Temperature Squared lag 12

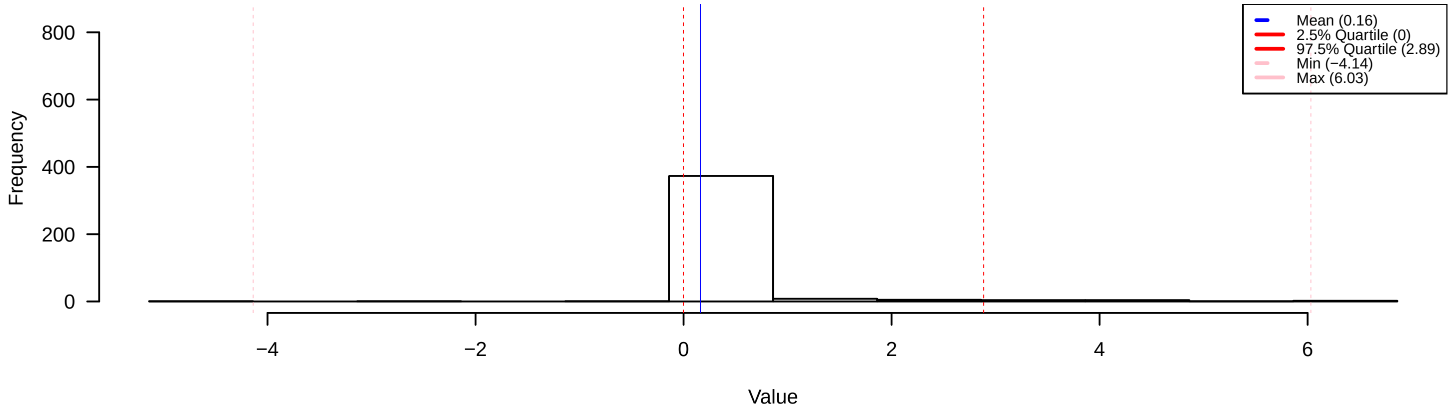

S5, Figure 334 : Bootstrap Distribution of Dewpoint Temperature:Air Temperature Squared lag 13

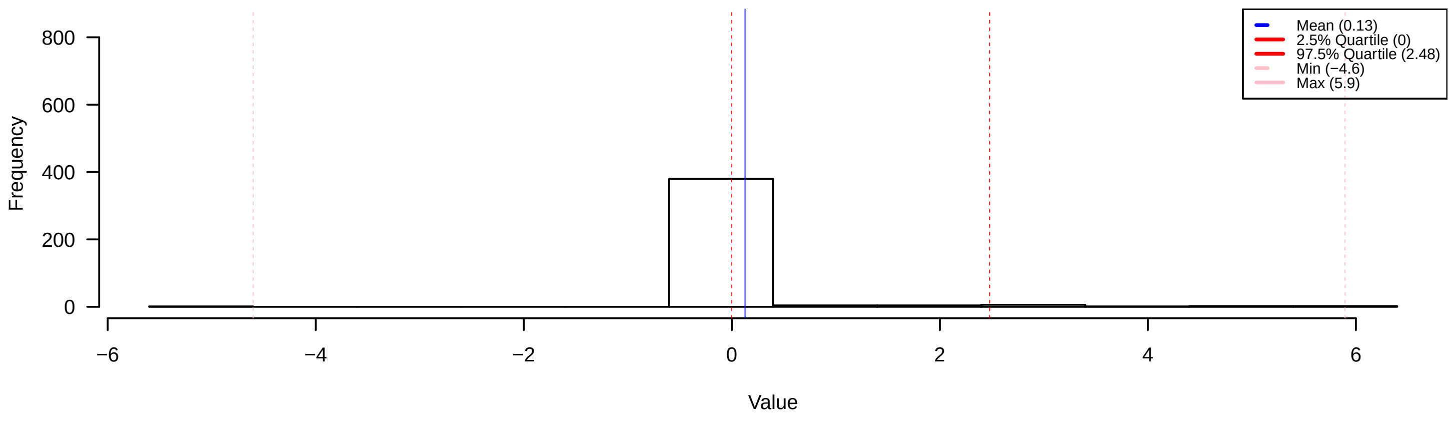

S5, Figure 335 : Bootstrap Distribution of Dewpoint Temperature:Air Temperature Squared lag 14

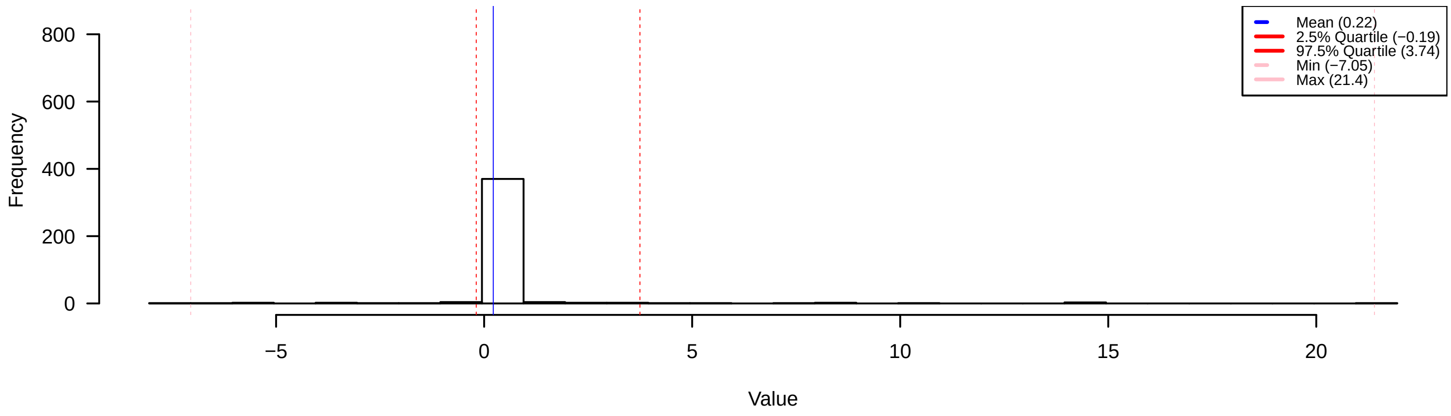

S5, Figure 336 : Bootstrap Distribution of Dewpoint Temperature:Air Temperature Squared lag 15

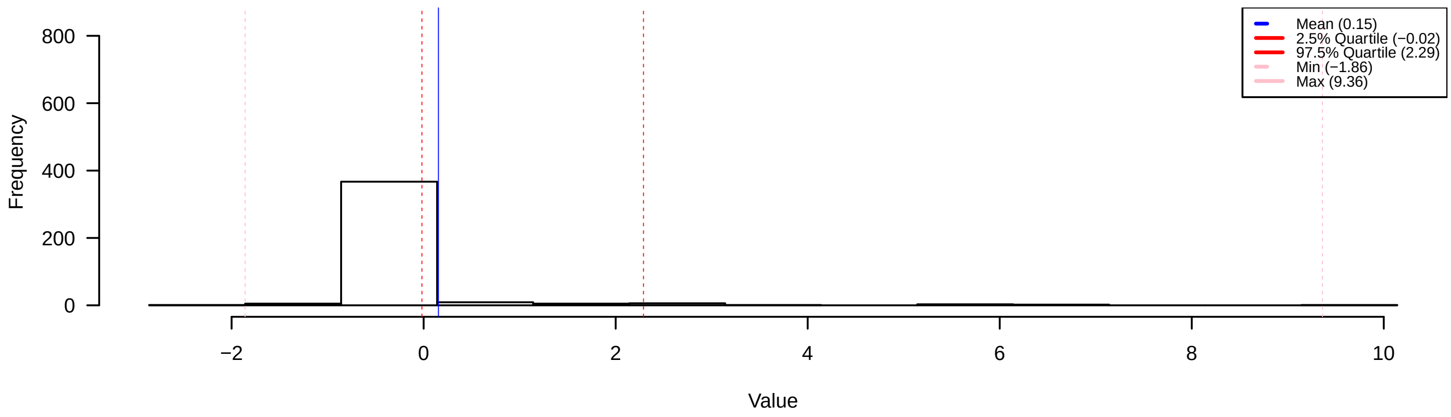

S5, Figure 337 : Bootstrap Distribution of Dewpoint Temperature:Air Temperature Squared lag 16

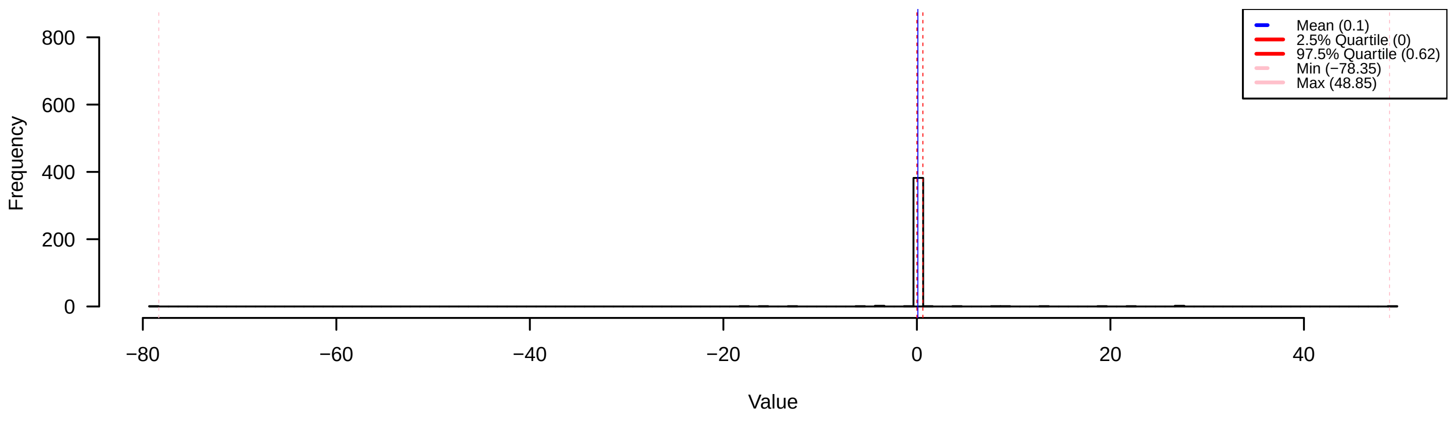

S5, Figure 338 : Bootstrap Distribution of Dewpoint Temperature:Air Temperature Squared lag 17

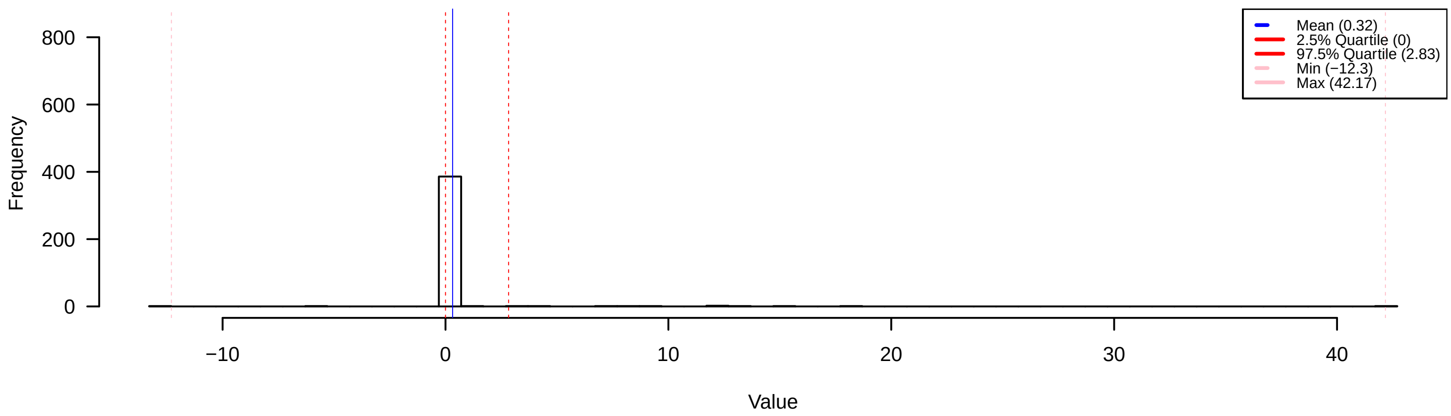

S5, Figure 339 : Bootstrap Distribution of Dewpoint Temperature:Air Temperature Squared lag 18

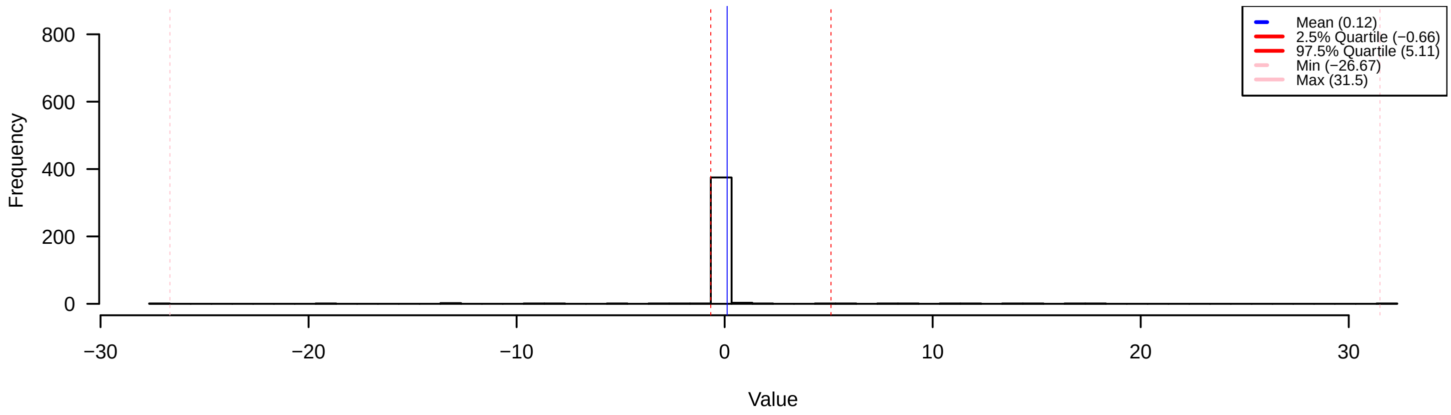

S5, Figure 340 : Bootstrap Distribution of Dewpoint Temperature:Air Temperature Squared lag 19

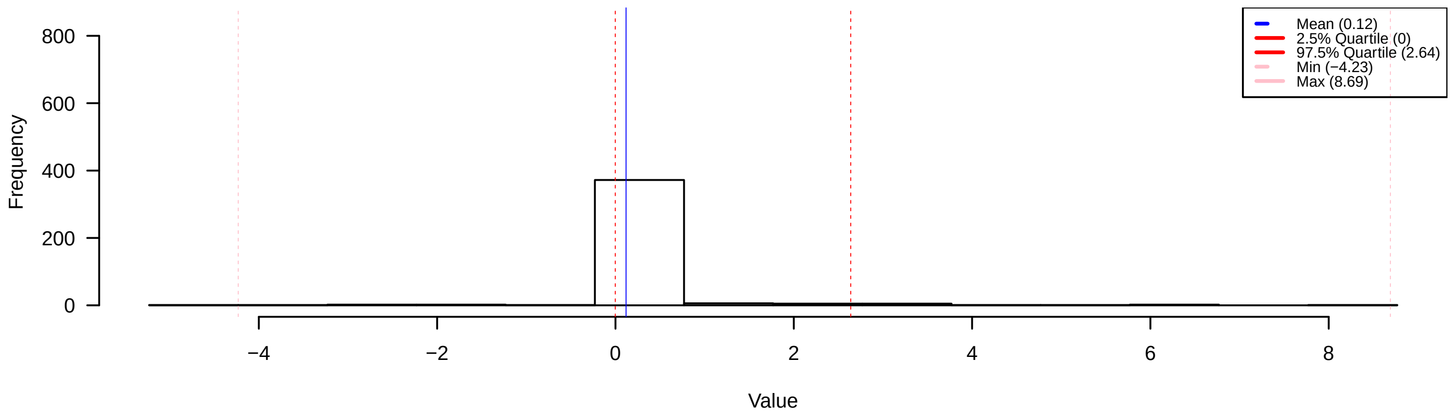

S5, Figure 341 : Bootstrap Distribution of Dewpoint Temperature:Air Temperature Squared lag 20

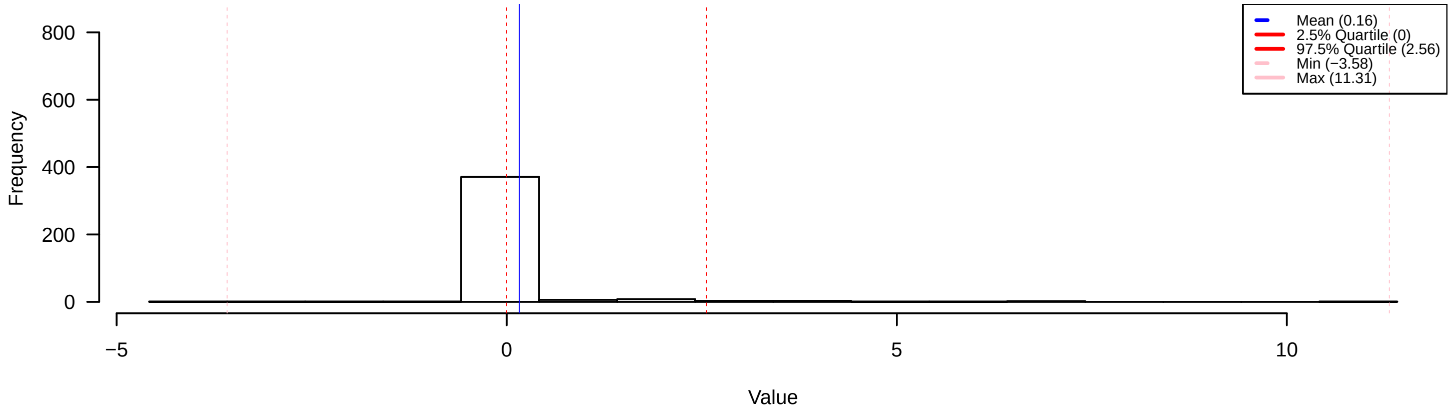

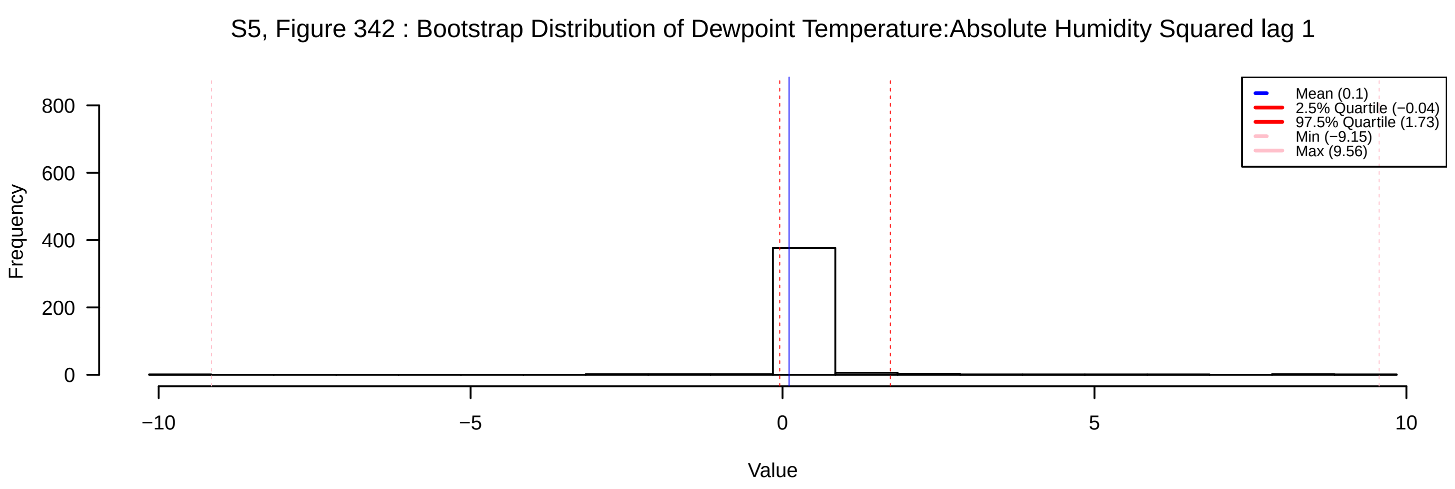
S5, Figure 342 : Bootstrap Distribution of Dewpoint Temperature:Absolute Humidity Squared lag 1
Mean (0.1)
2.5% Quartile (−0.04)
97.5% Quartile (1.73)
Min (−9.15)
Max (9.56)
Frequency
Value

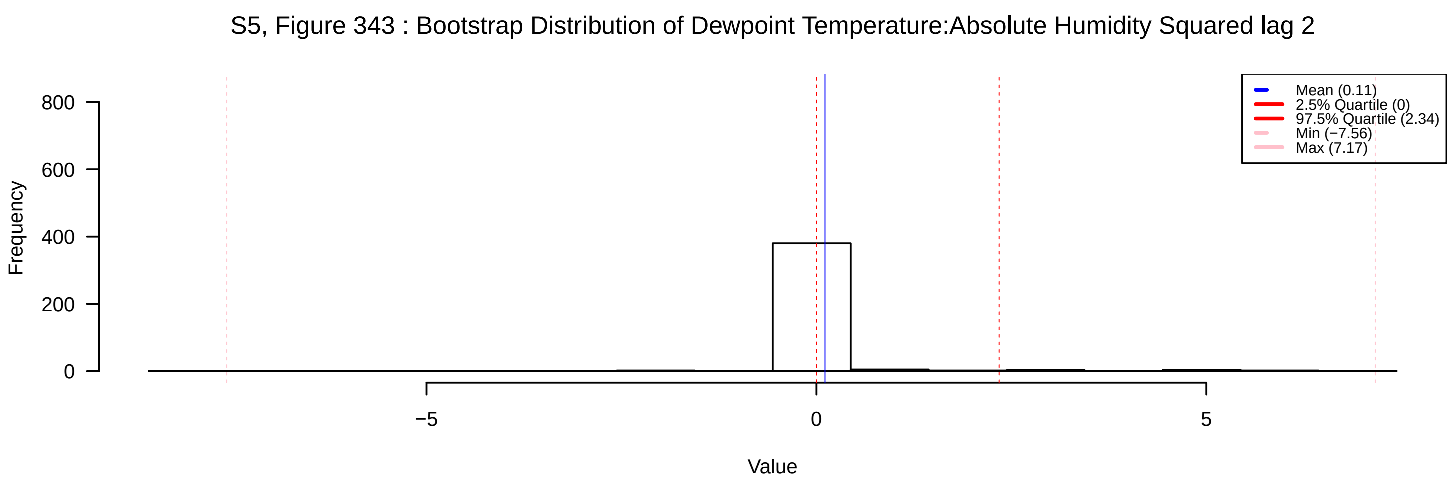
S5, Figure 343 : Bootstrap Distribution of Dewpoint Temperature:Absolute Humidity Squared lag 2
Mean (0.11)
2.5% Quartile (0)
97.5% Quartile (2.34)
Min (−7.56)
Max (7.17)
Frequency
Value

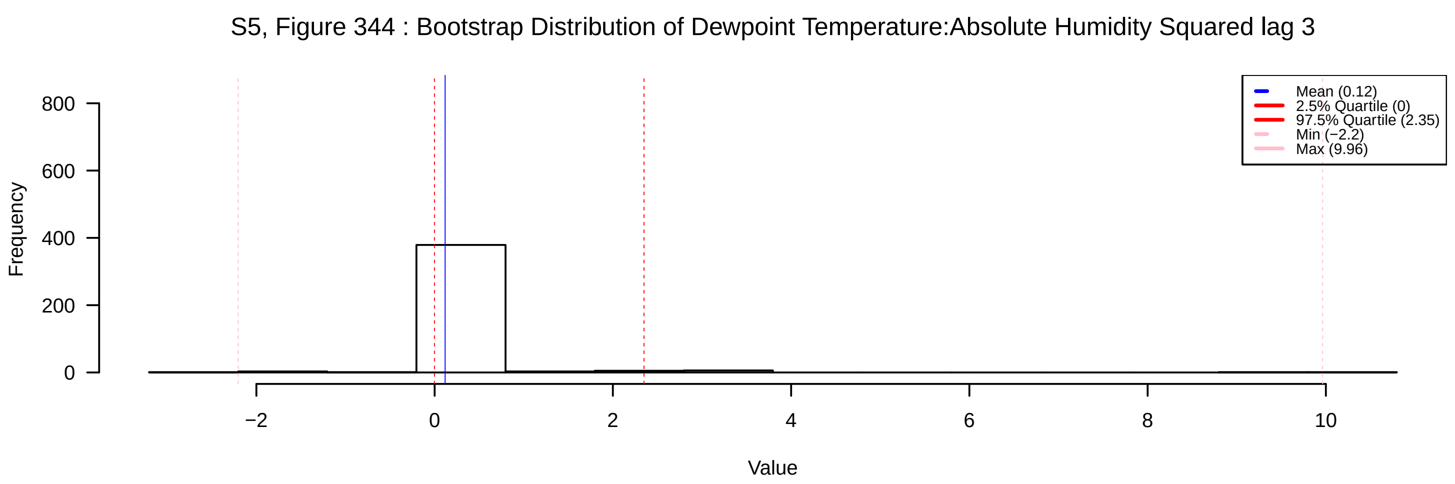
S5, Figure 344 : Bootstrap Distribution of Dewpoint Temperature:Absolute Humidity Squared lag 3
Mean (0.12)
2.5% Quartile (0)
97.5% Quartile (2.35)
Min (−2.2)
Max (9.96)
Frequency
Value

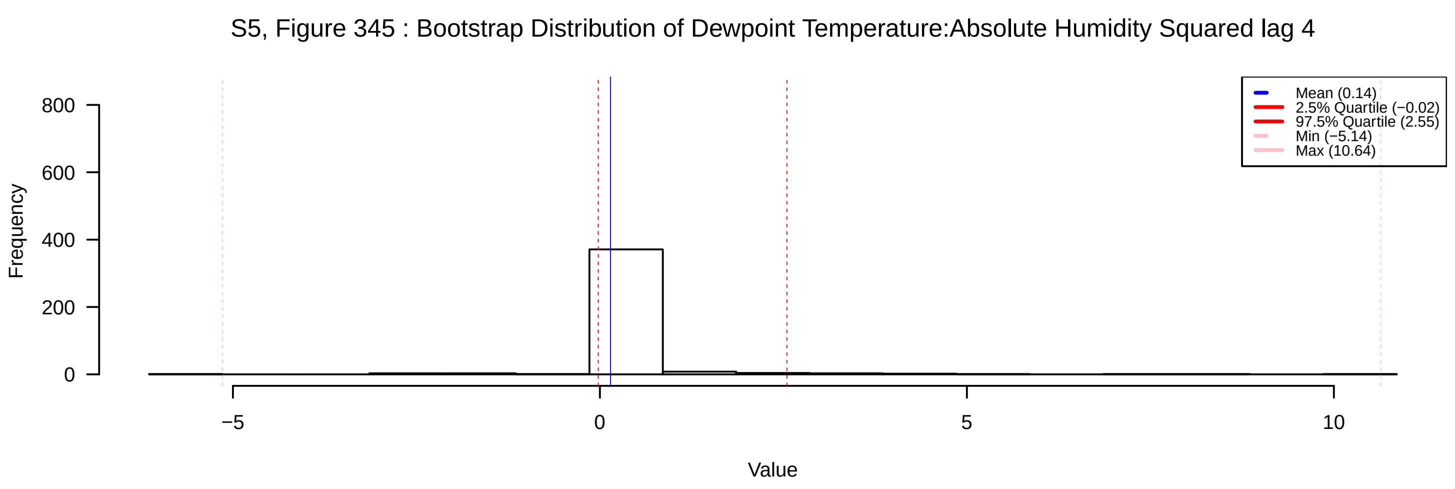
S5, Figure 345 : Bootstrap Distribution of Dewpoint Temperature:Absolute Humidity Squared lag 4
Mean (0.14)
2.5% Quartile (−0.02)
97.5% Quartile (2.55)
Min (−5.14)
Max (10.64)
Frequency
Value

S5, Figure 346 : Bootstrap Distribution of Dewpoint Temperature:Absolute Humidity Squared lag 5

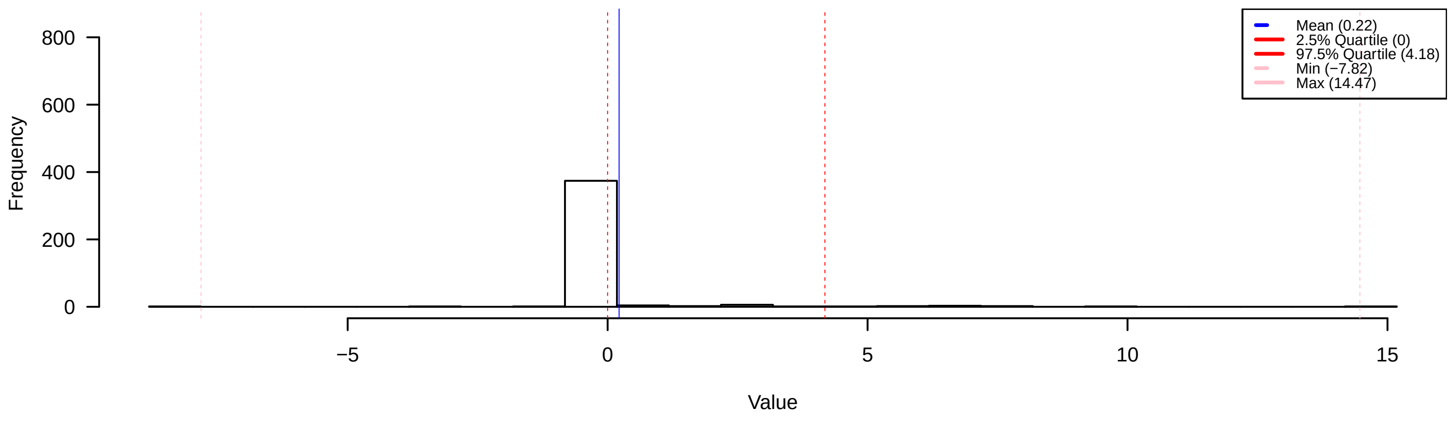

S5, Figure 347 : Bootstrap Distribution of Dewpoint Temperature:Absolute Humidity Squared lag 6

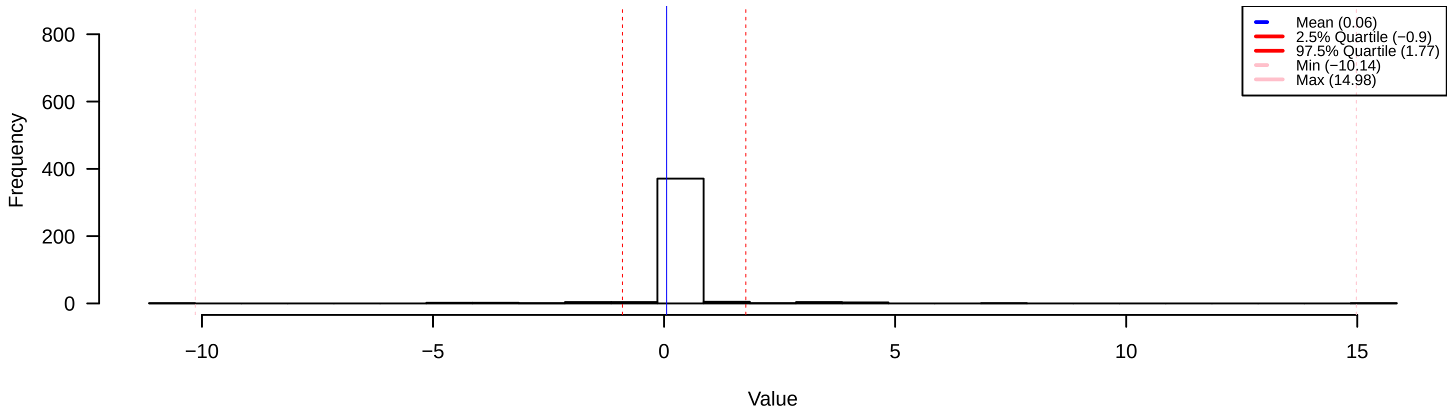

S5, Figure 348 : Bootstrap Distribution of Dewpoint Temperature:Absolute Humidity Squared lag 7

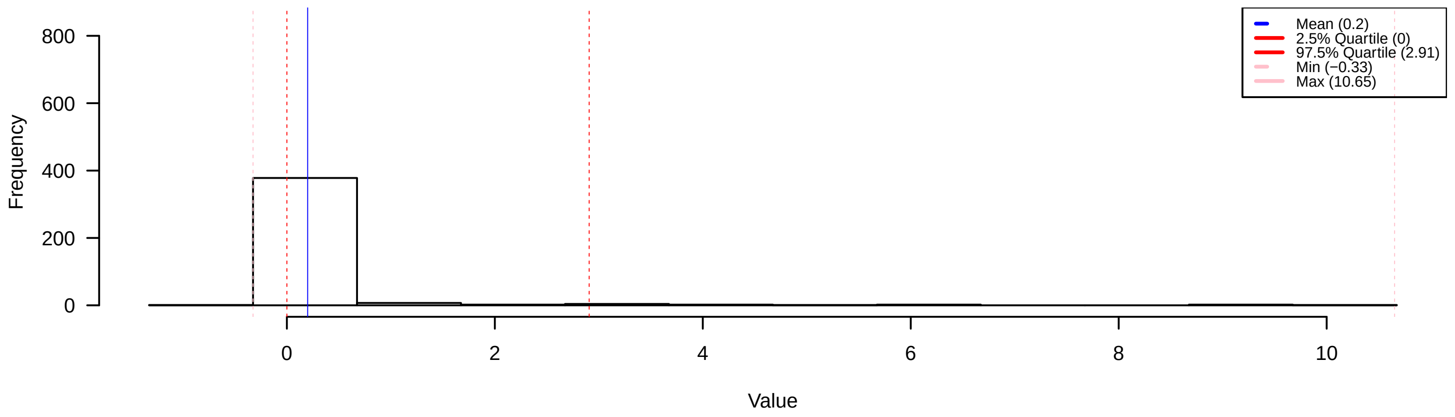

S5, Figure 349 : Bootstrap Distribution of Dewpoint Temperature:Absolute Humidity Squared lag 8

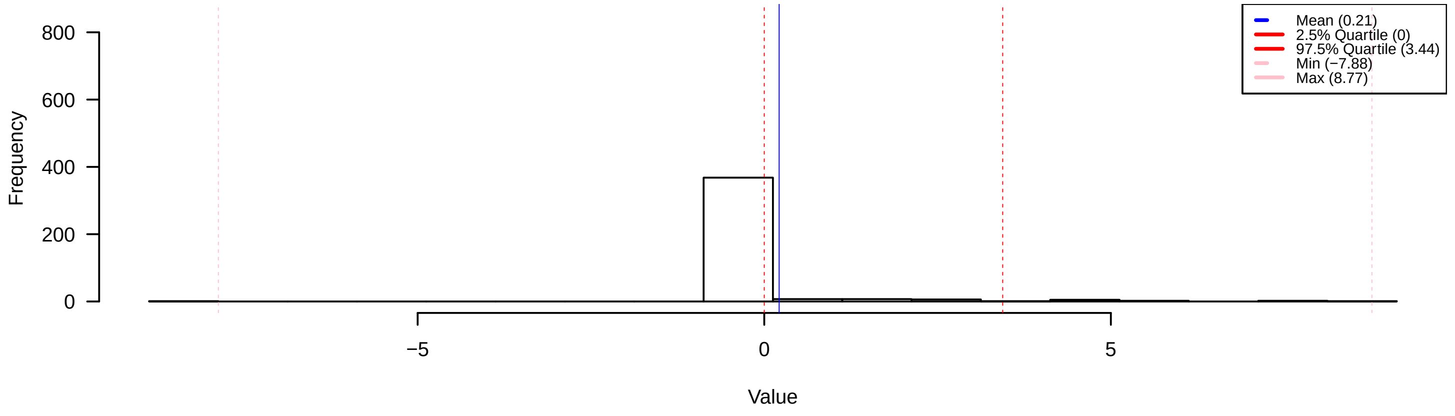

S5, Figure 350 : Bootstrap Distribution of Dewpoint Temperature:Absolute Humidity Squared lag 9

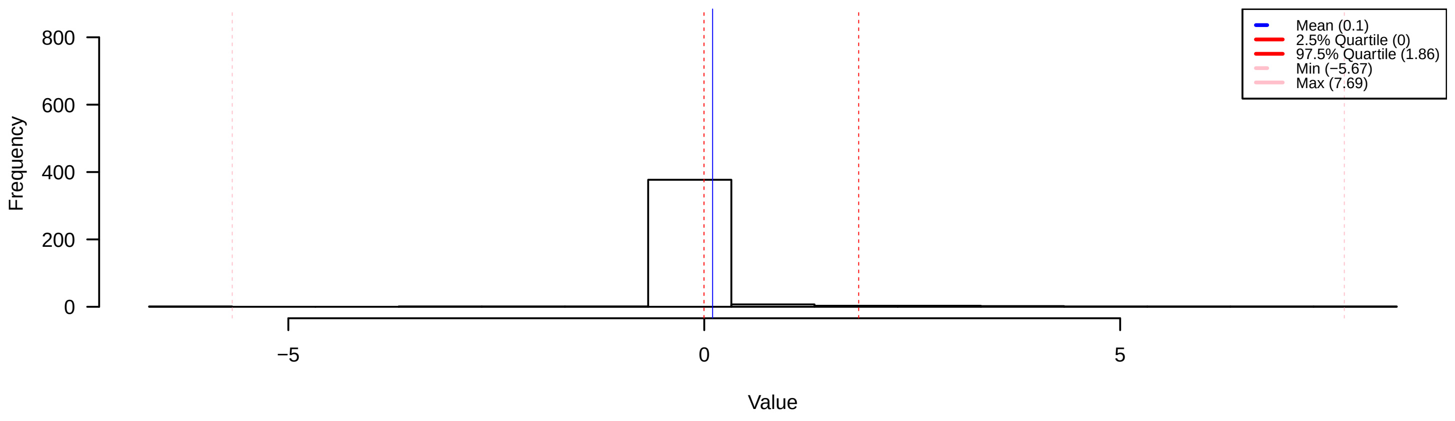

S5, Figure 351 : Bootstrap Distribution of Dewpoint Temperature:Absolute Humidity Squared lag 10

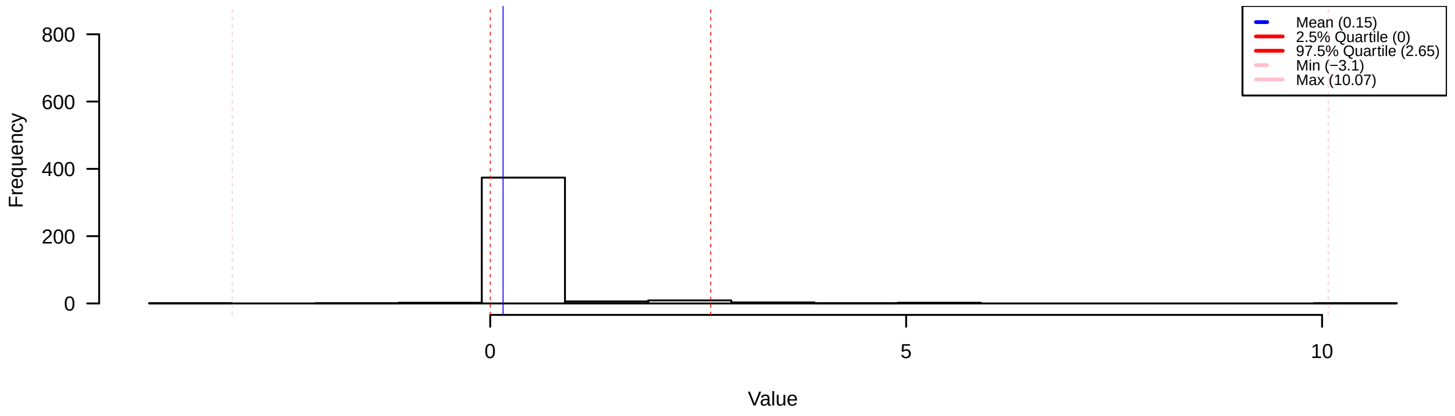

S5, Figure 352 : Bootstrap Distribution of Dewpoint Temperature:Absolute Humidity Squared lag 11

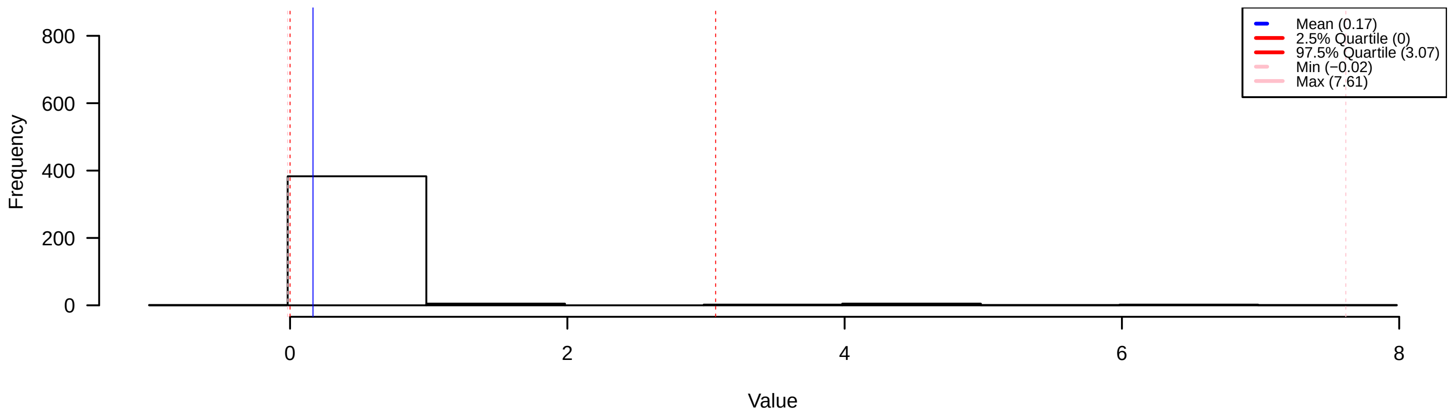

S5, Figure 353 : Bootstrap Distribution of Dewpoint Temperature:Absolute Humidity Squared lag 12

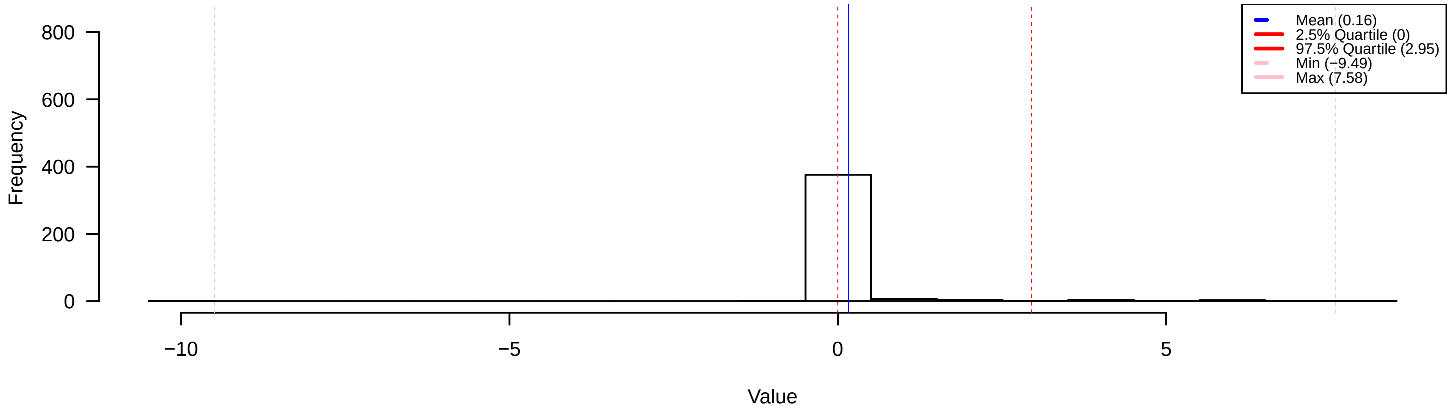

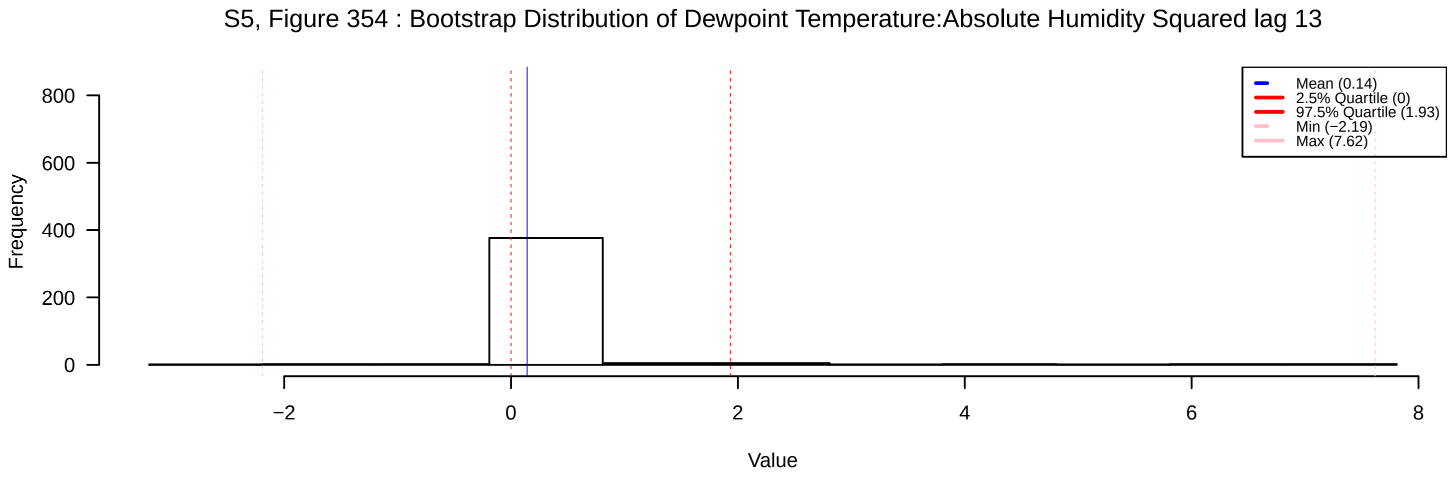
S5, Figure 354 : Bootstrap Distribution of Dewpoint Temperature:Absolute Humidity Squared lag 13
Mean (0.14)
2.5% Quartile (0)
97.5% Quartile (1.93)
Min (−2.19)
Max (7.62)
Frequency
Value

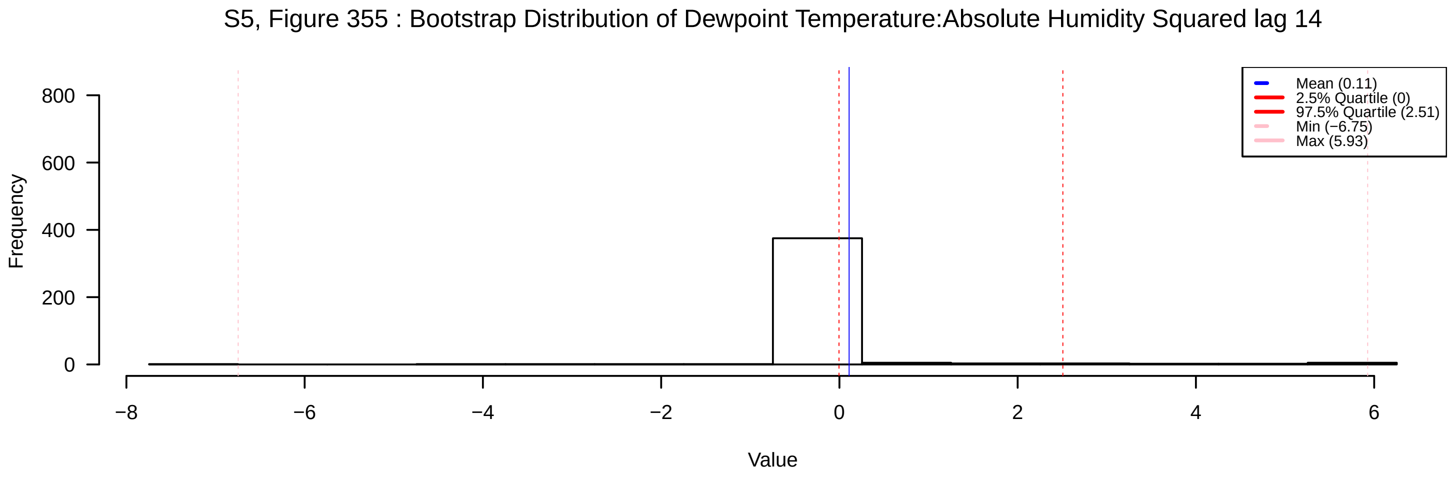
S5, Figure 355 : Bootstrap Distribution of Dewpoint Temperature:Absolute Humidity Squared lag 14
Mean (0.11)
2.5% Quartile (0)
97.5% Quartile (2.51)
Min (−6.75)
Max (5.93)
Frequency
Value

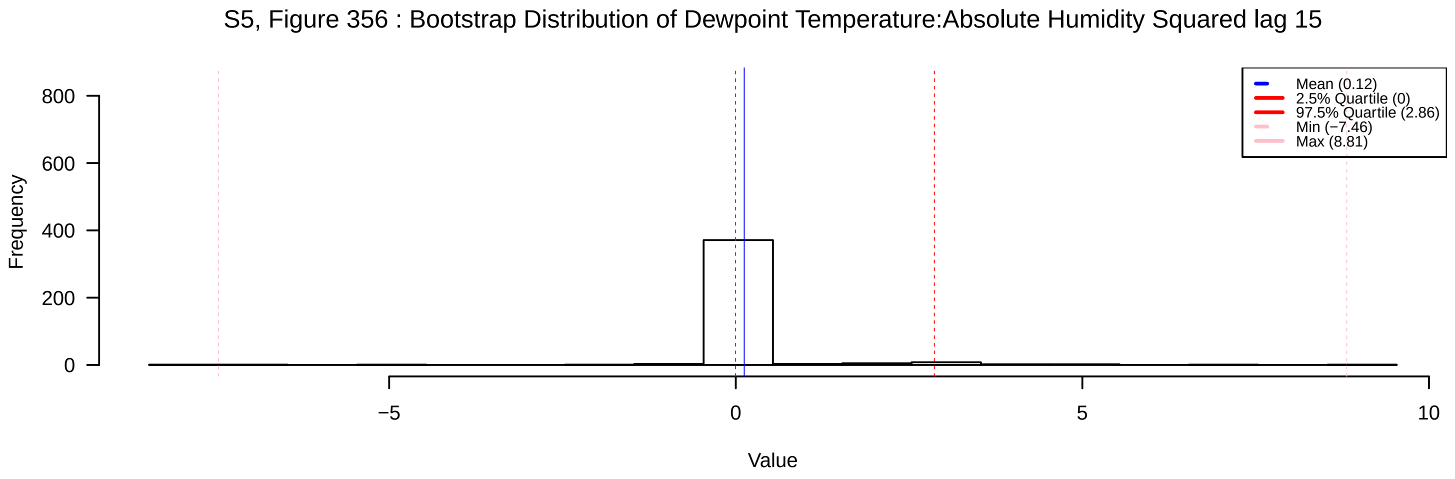
S5, Figure 356 : Bootstrap Distribution of Dewpoint Temperature:Absolute Humidity Squared lag 15
Mean (0.12)
2.5% Quartile (0)
97.5% Quartile (2.86)
Min (−7.46)
Max (8.81)
Frequency
Value

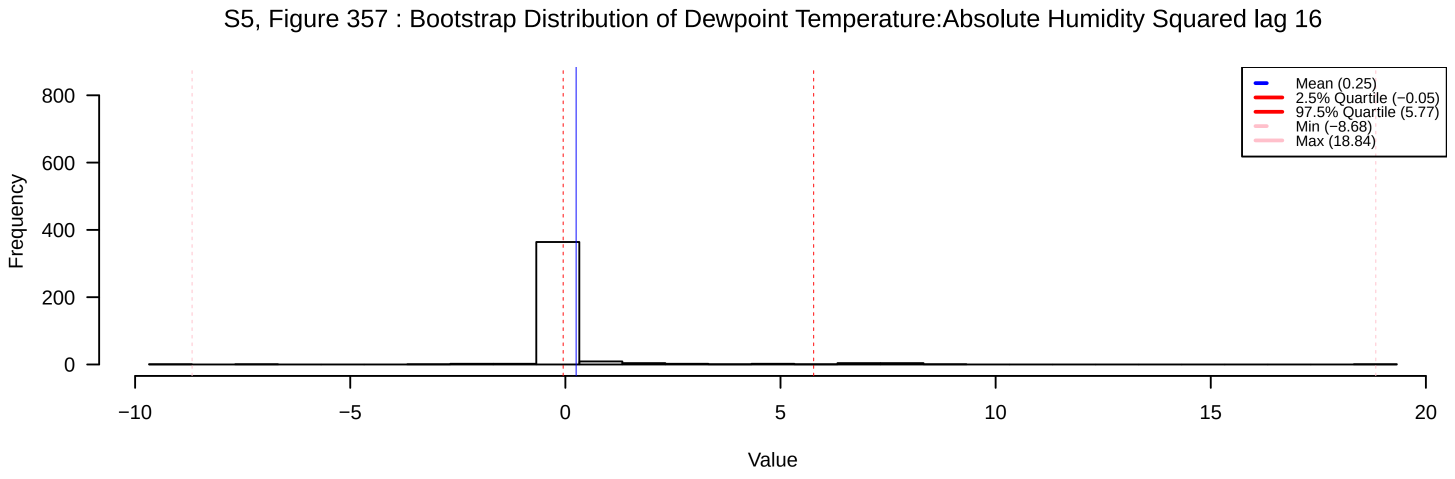
S5, Figure 357 : Bootstrap Distribution of Dewpoint Temperature:Absolute Humidity Squared lag 16
Mean (0.25)
2.5% Quartile (−0.05)
97.5% Quartile (5.77)
Min (−8.68)
Max (18.84)
Frequency
Value

S5, Figure 358 : Bootstrap Distribution of Dewpoint Temperature:Absolute Humidity Squared lag 17

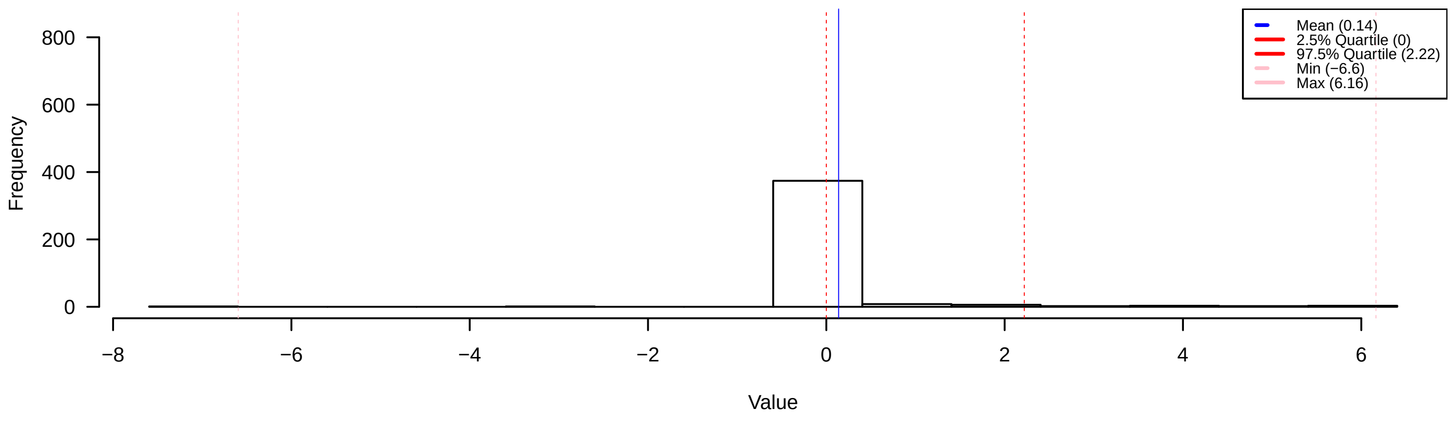

S5, Figure 359 : Bootstrap Distribution of Dewpoint Temperature:Absolute Humidity Squared lag 18

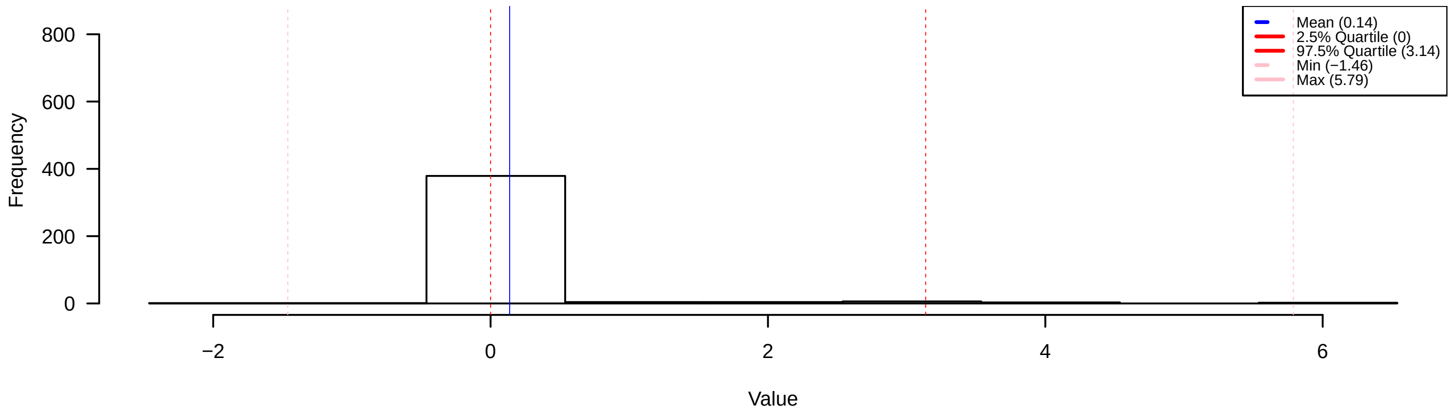

S5, Figure 360 : Bootstrap Distribution of Dewpoint Temperature:Absolute Humidity Squared lag 19

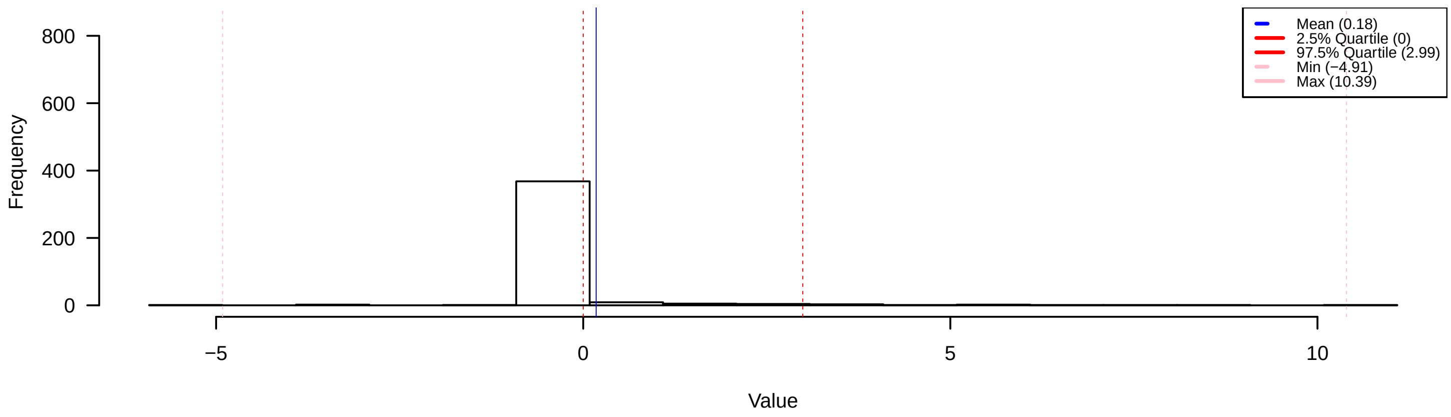

S5, Figure 361 : Bootstrap Distribution of Dewpoint Temperature:Absolute Humidity Squared lag 20

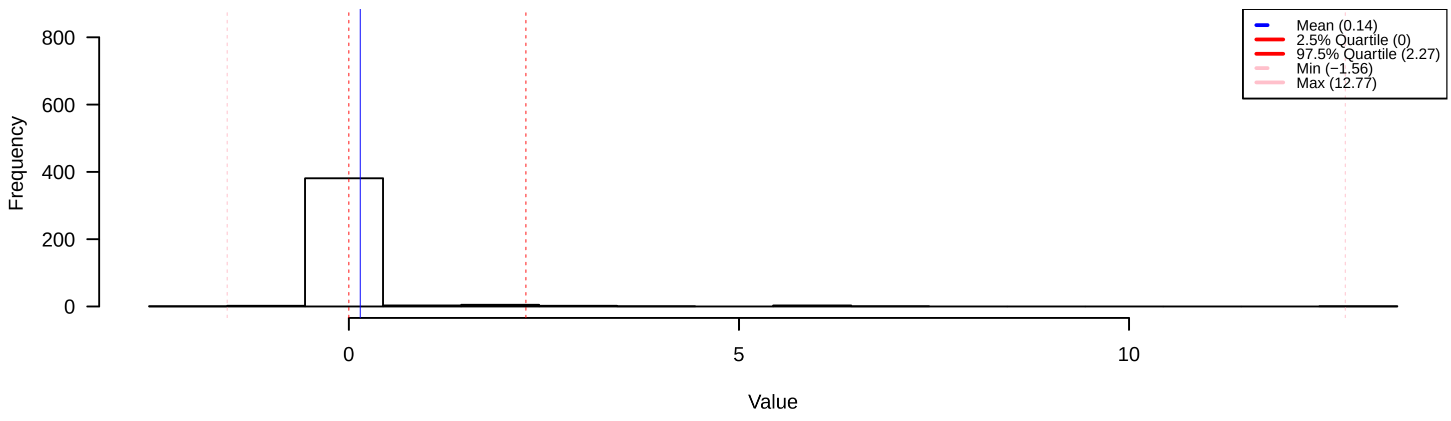

S5, Figure 362 : Bootstrap Distribution of Dewpoint Temperature:Relative Humidity Squared lag 1

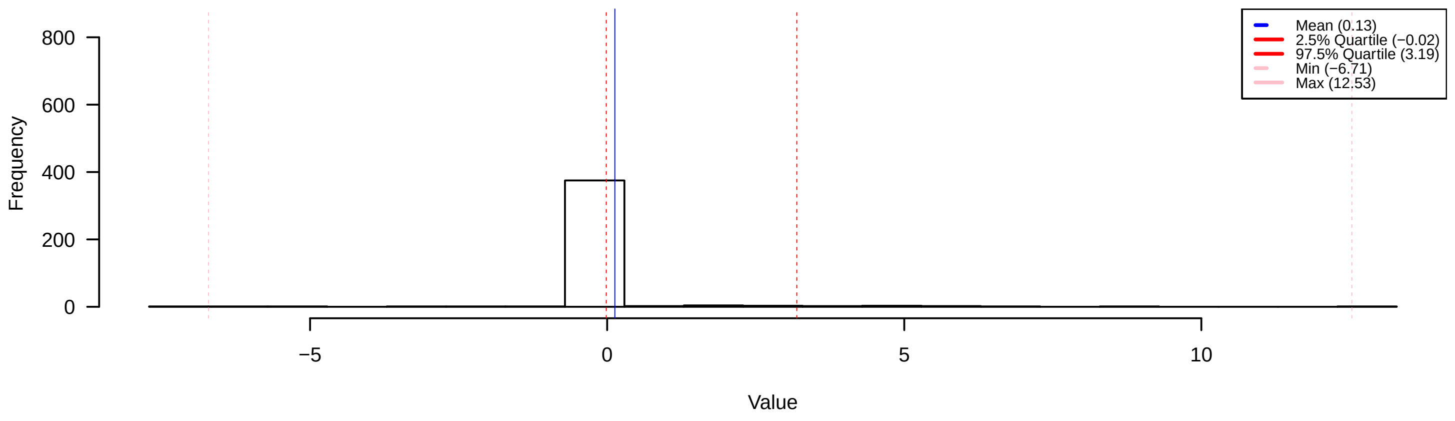

S5, Figure 363 : Bootstrap Distribution of Dewpoint Temperature:Relative Humidity Squared lag 2

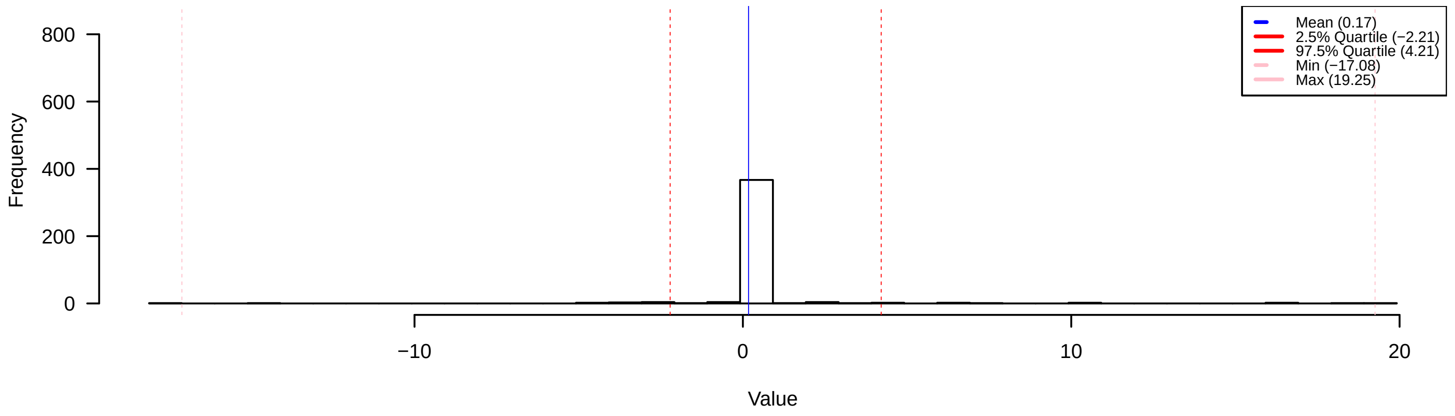

S5, Figure 364 : Bootstrap Distribution of Dewpoint Temperature:Relative Humidity Squared lag 3

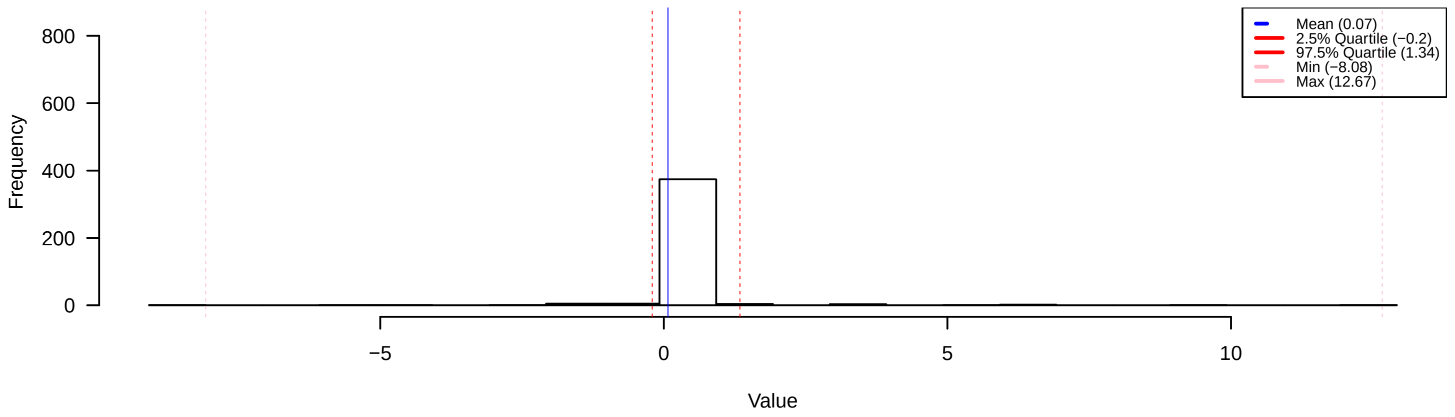

S5, Figure 365 : Bootstrap Distribution of Dewpoint Temperature:Relative Humidity Squared lag 4

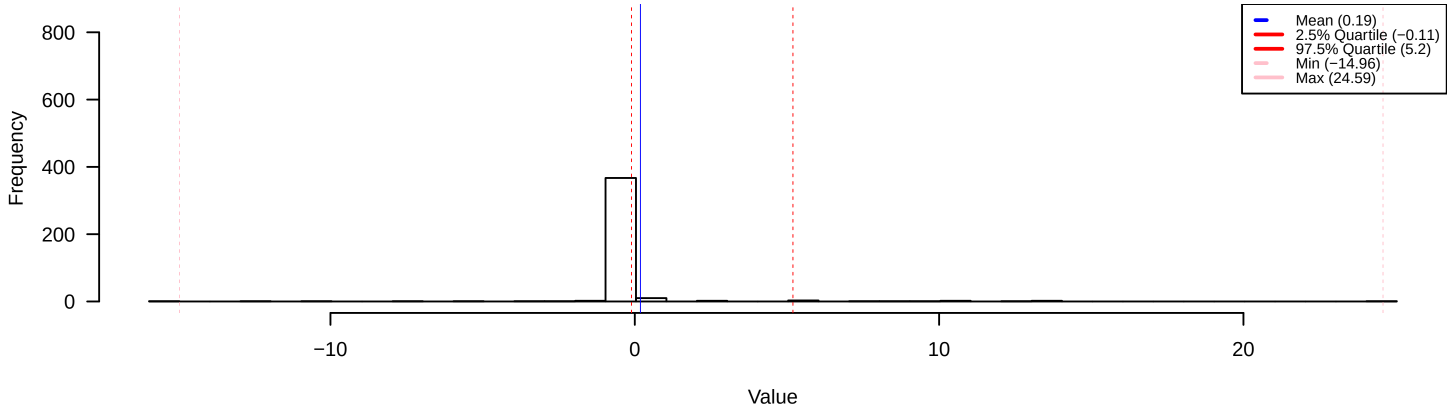

S5, Figure 366 : Bootstrap Distribution of Dewpoint Temperature:Relative Humidity Squared lag 5

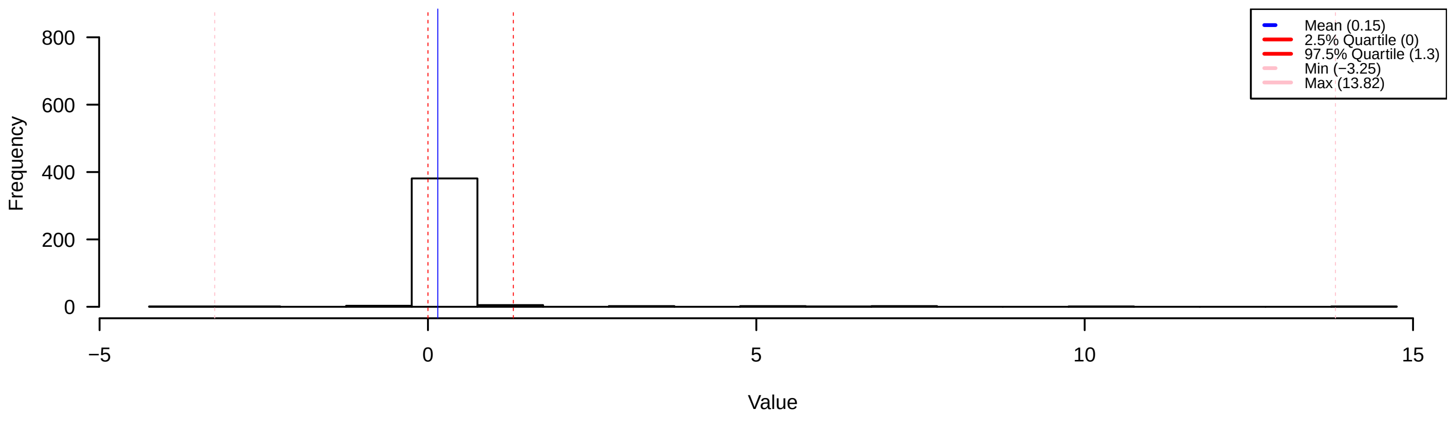

S5, Figure 367 : Bootstrap Distribution of Dewpoint Temperature:Relative Humidity Squared lag 6

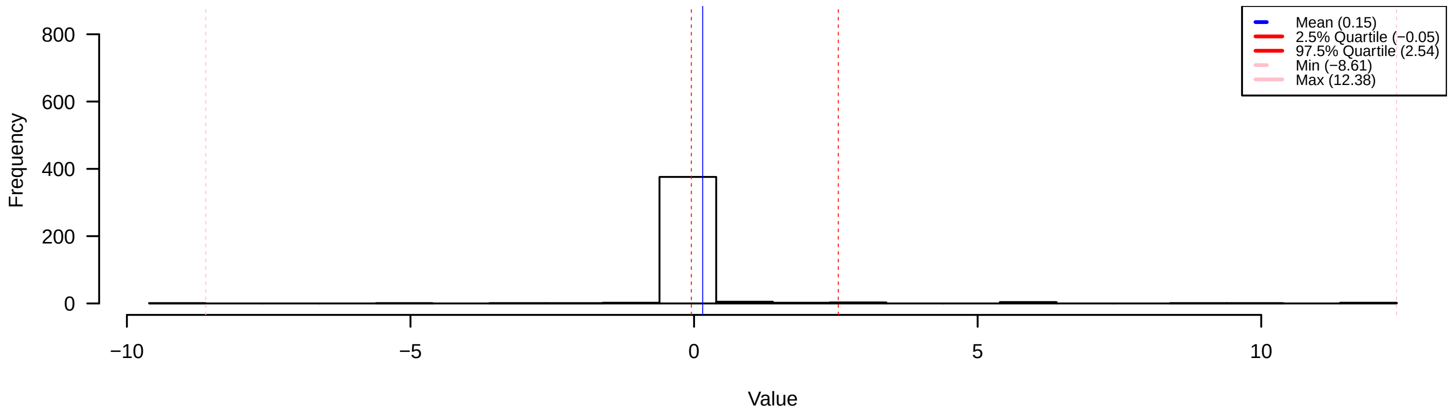

S5, Figure 368 : Bootstrap Distribution of Dewpoint Temperature:Relative Humidity Squared lag 7

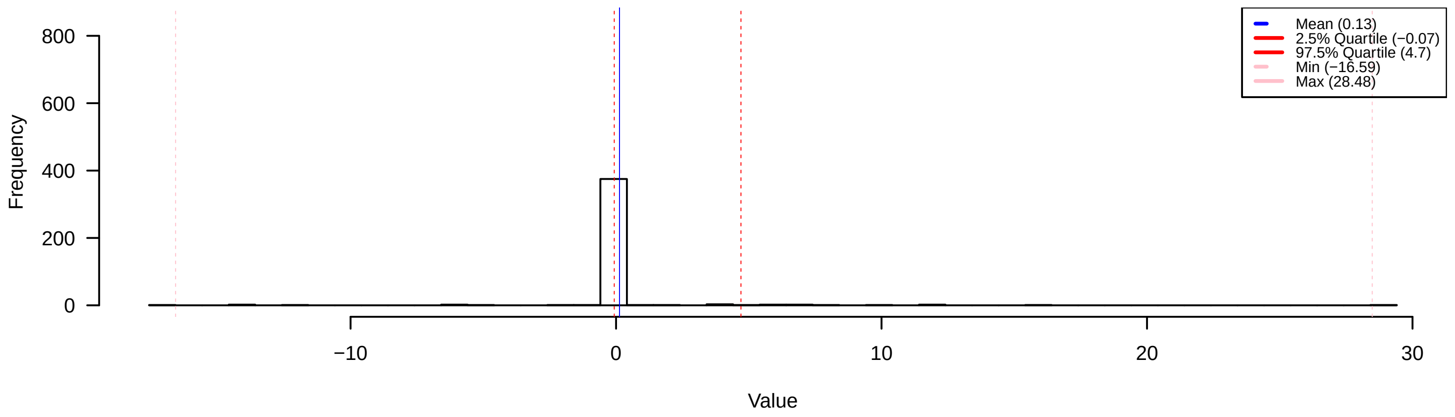

S5, Figure 369 : Bootstrap Distribution of Dewpoint Temperature:Relative Humidity Squared lag 8

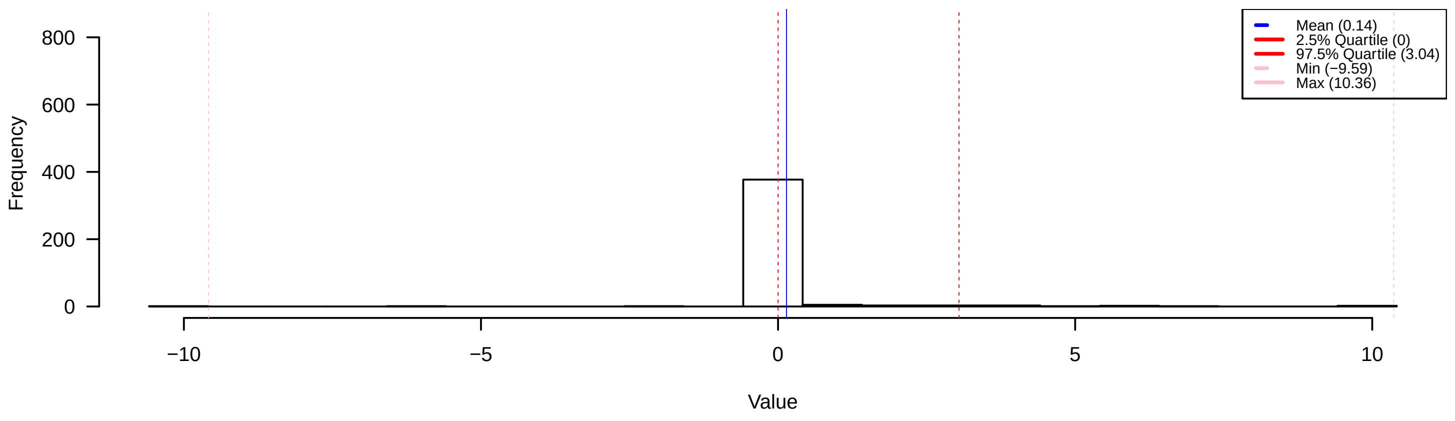

S5, Figure 370 : Bootstrap Distribution of Dewpoint Temperature:Relative Humidity Squared lag 9

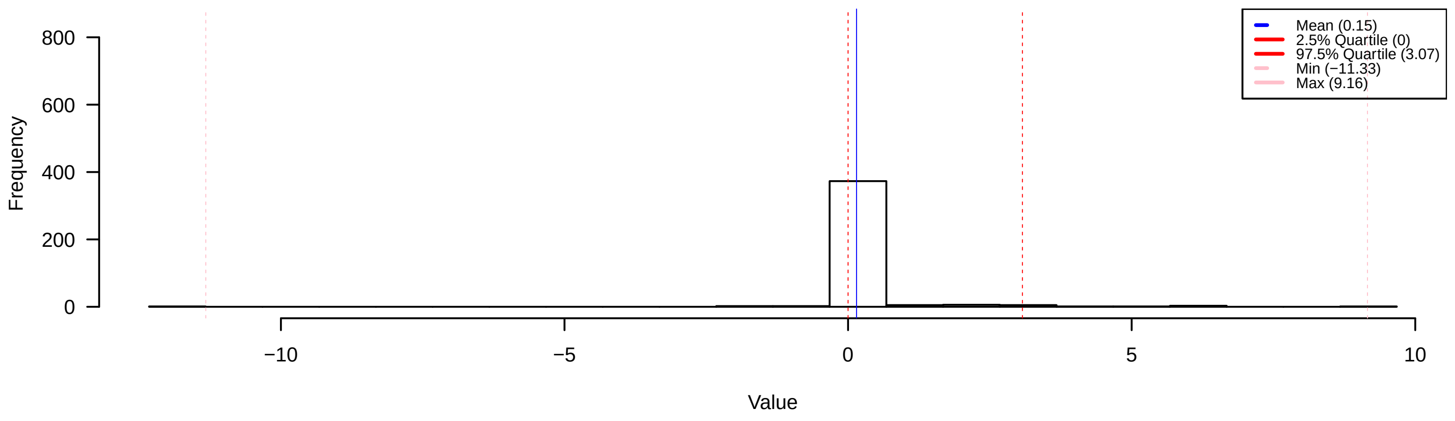

S5, Figure 371 : Bootstrap Distribution of Dewpoint Temperature:Relative Humidity Squared lag 10

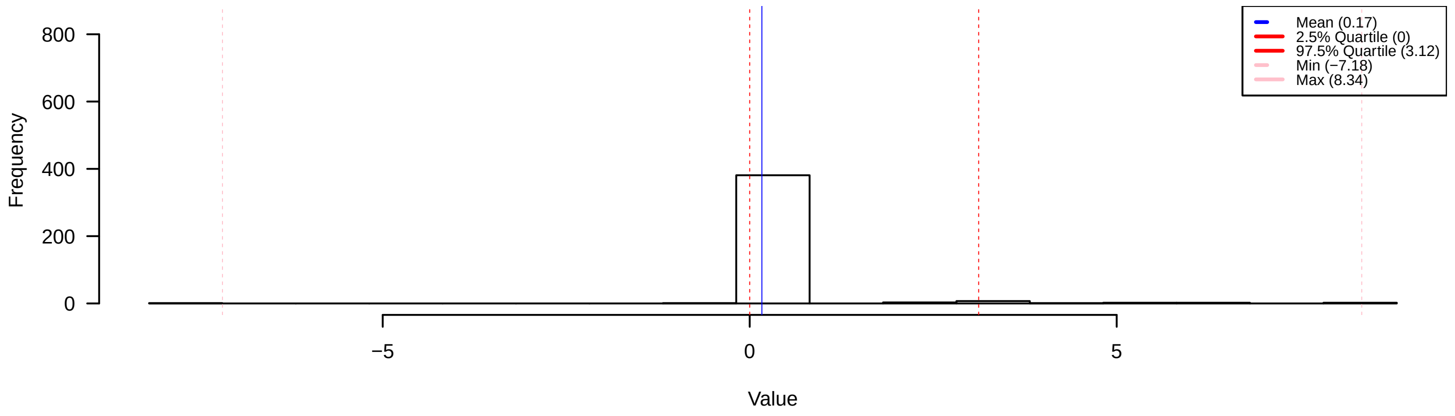

S5, Figure 372 : Bootstrap Distribution of Dewpoint Temperature:Relative Humidity Squared lag 11

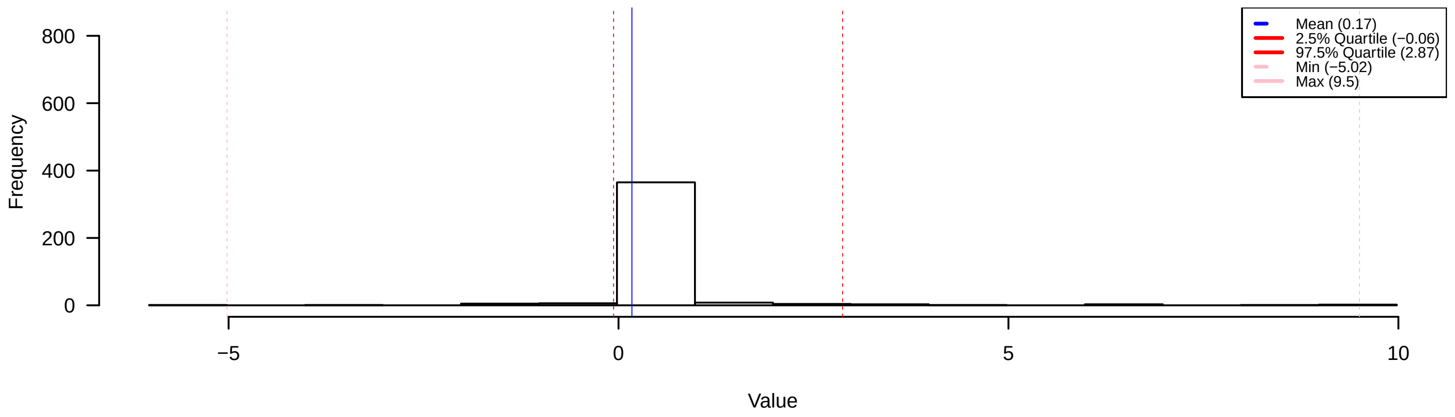

S5, Figure 373 : Bootstrap Distribution of Dewpoint Temperature:Relative Humidity Squared lag 12

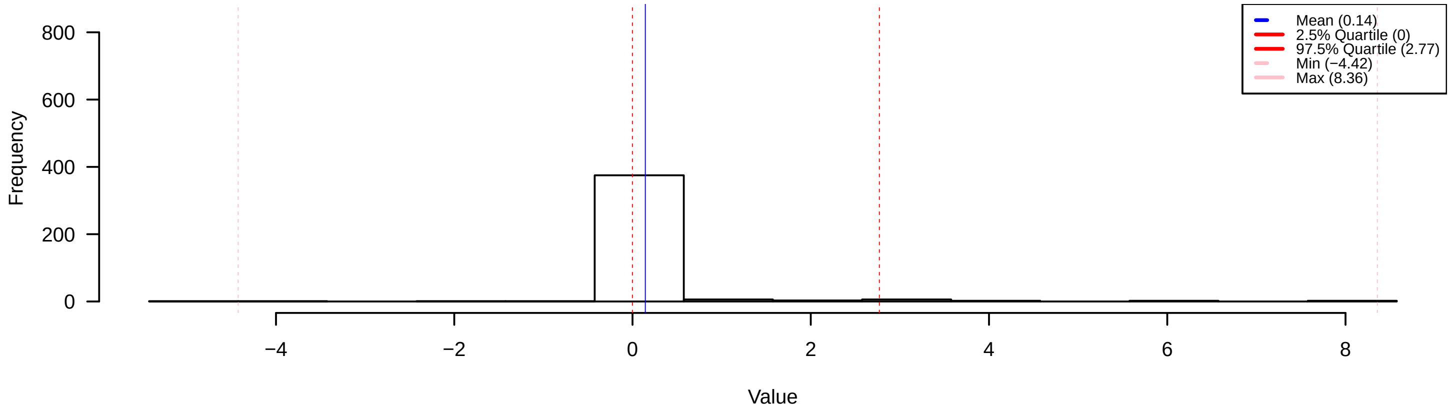

S5, Figure 374 : Bootstrap Distribution of Dewpoint Temperature:Relative Humidity Squared lag 13

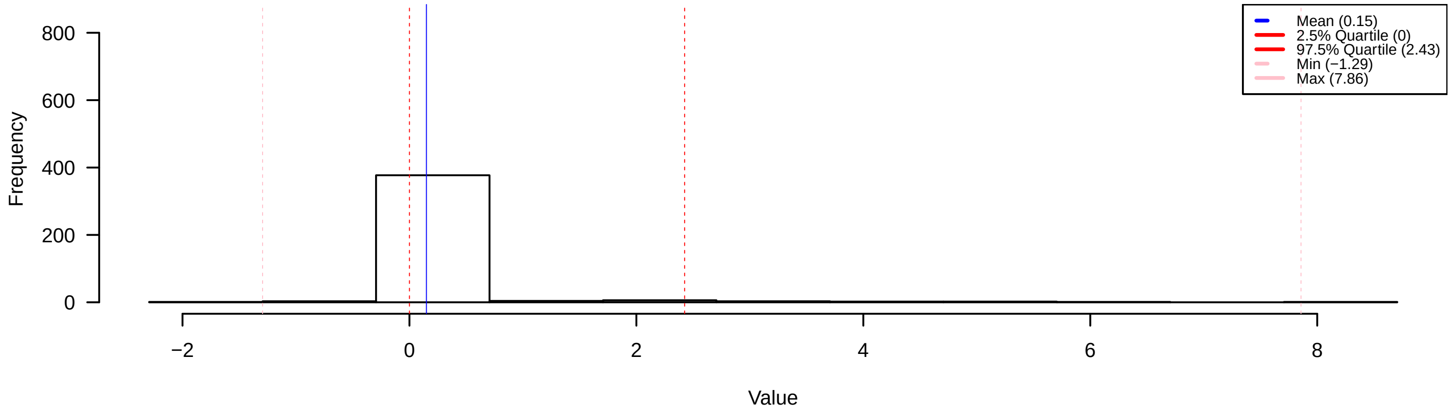

S5, Figure 375 : Bootstrap Distribution of Dewpoint Temperature:Relative Humidity Squared lag 14

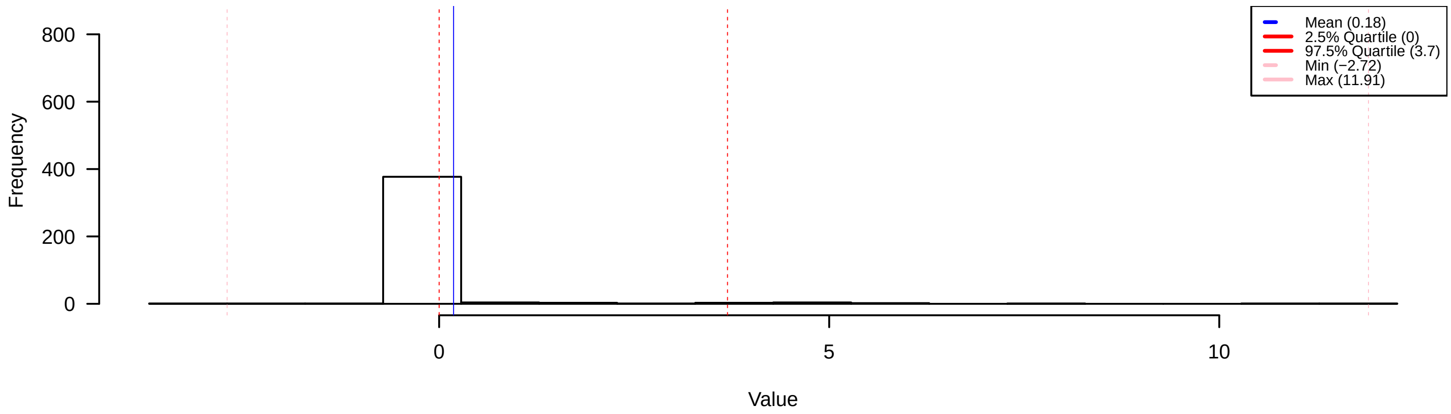

S5, Figure 376 : Bootstrap Distribution of Dewpoint Temperature:Relative Humidity Squared lag 15

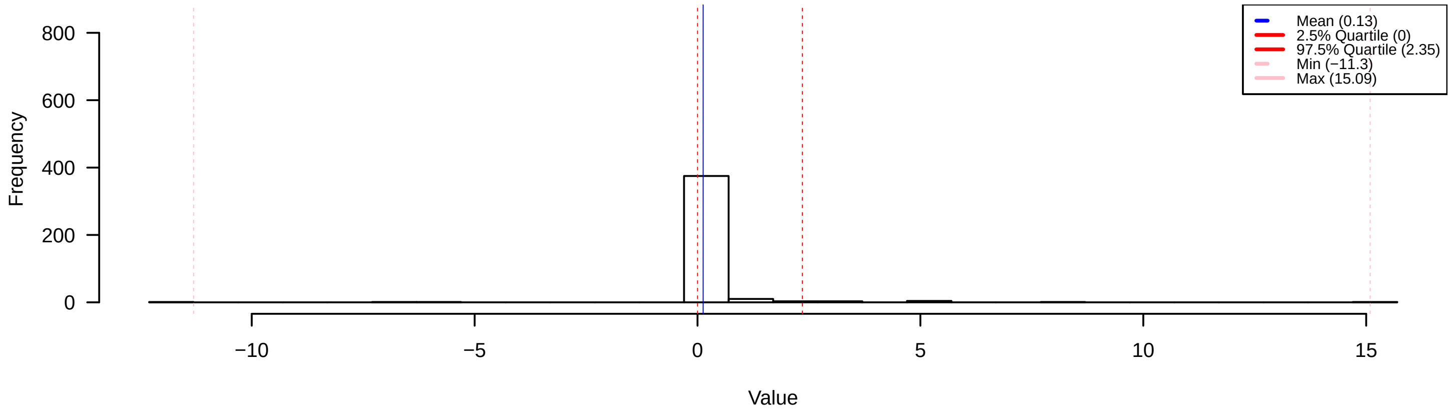

S5, Figure 377 : Bootstrap Distribution of Dewpoint Temperature:Relative Humidity Squared lag 16

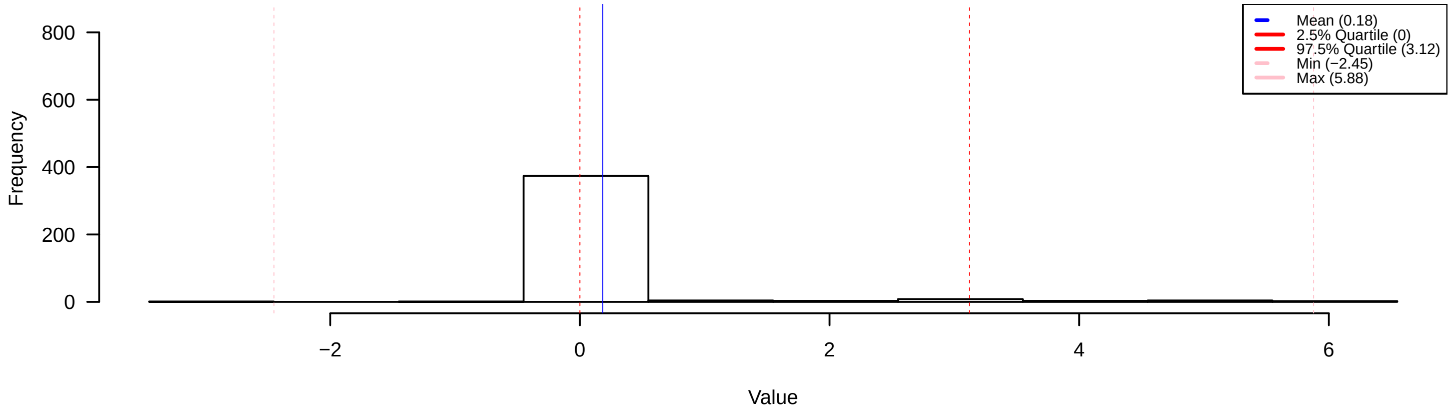

S5, Figure 378 : Bootstrap Distribution of Dewpoint Temperature:Relative Humidity Squared lag 17

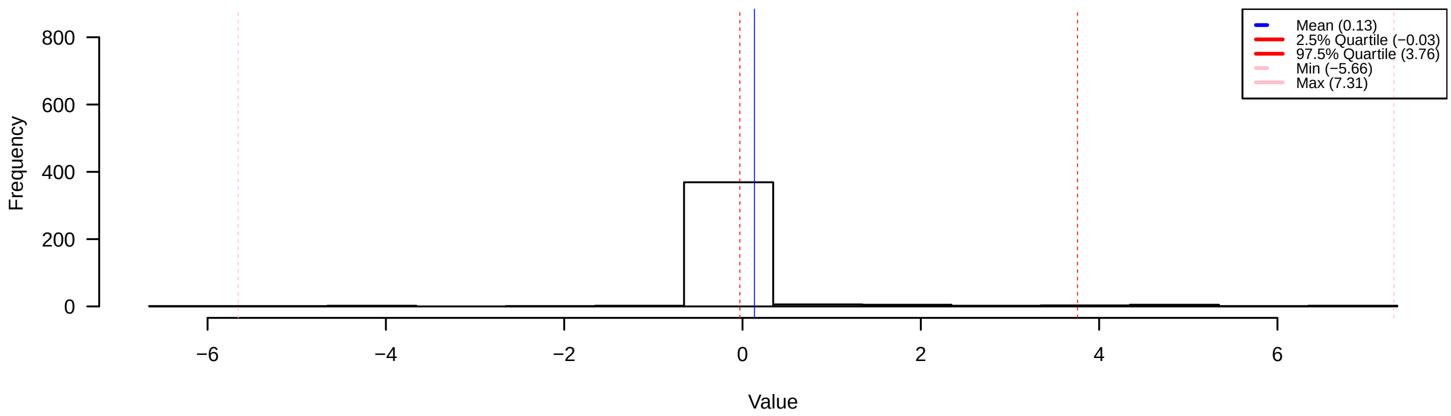

S5, Figure 379 : Bootstrap Distribution of Dewpoint Temperature:Relative Humidity Squared lag 18

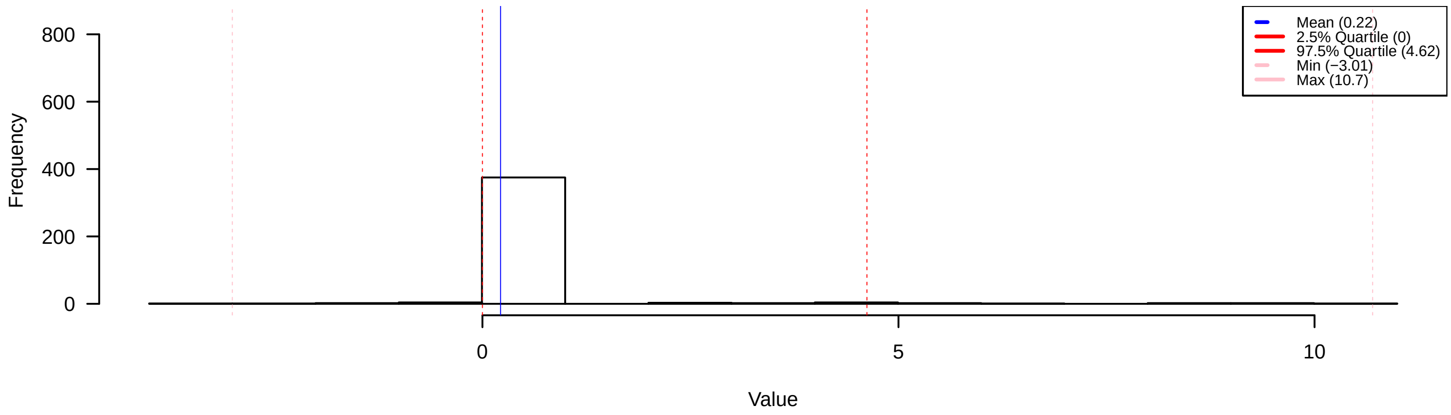

S5, Figure 380 : Bootstrap Distribution of Dewpoint Temperature:Relative Humidity Squared lag 19

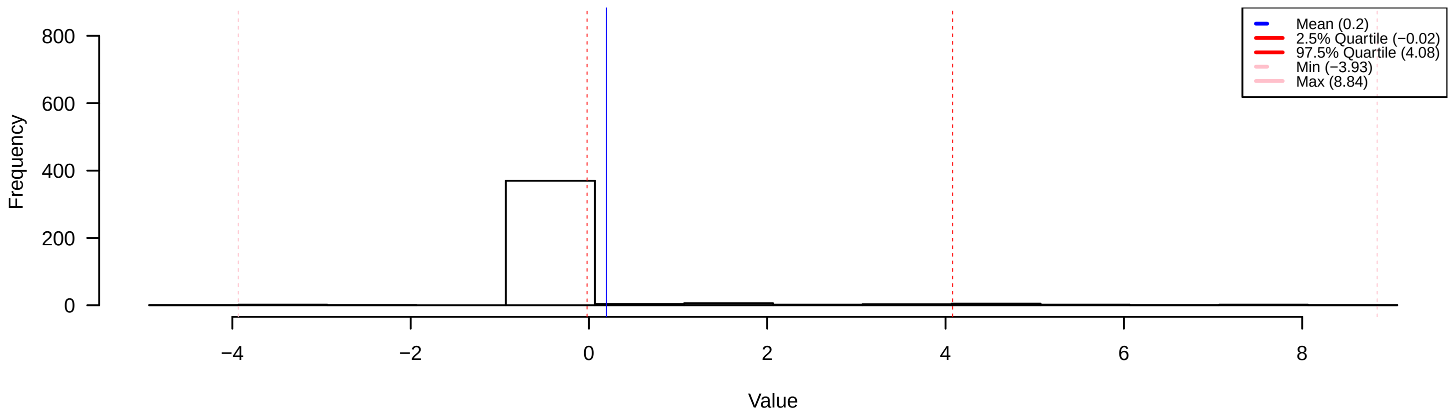

S5, Figure 381 : Bootstrap Distribution of Dewpoint Temperature:Relative Humidity Squared lag 20

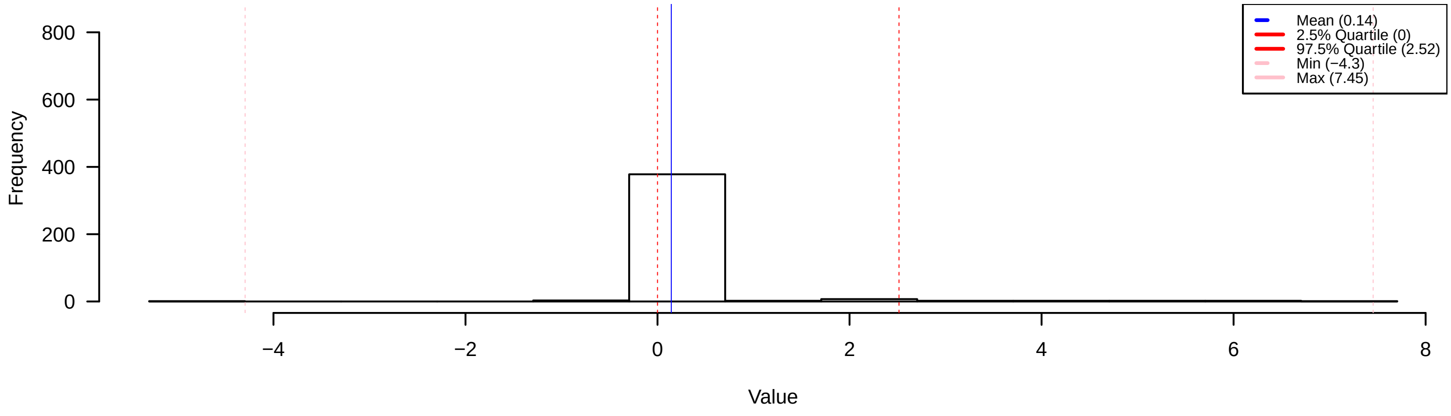

S5, Figure 382 : Bootstrap Distribution of Air Temperature:Absolute Humidity Squared lag 1

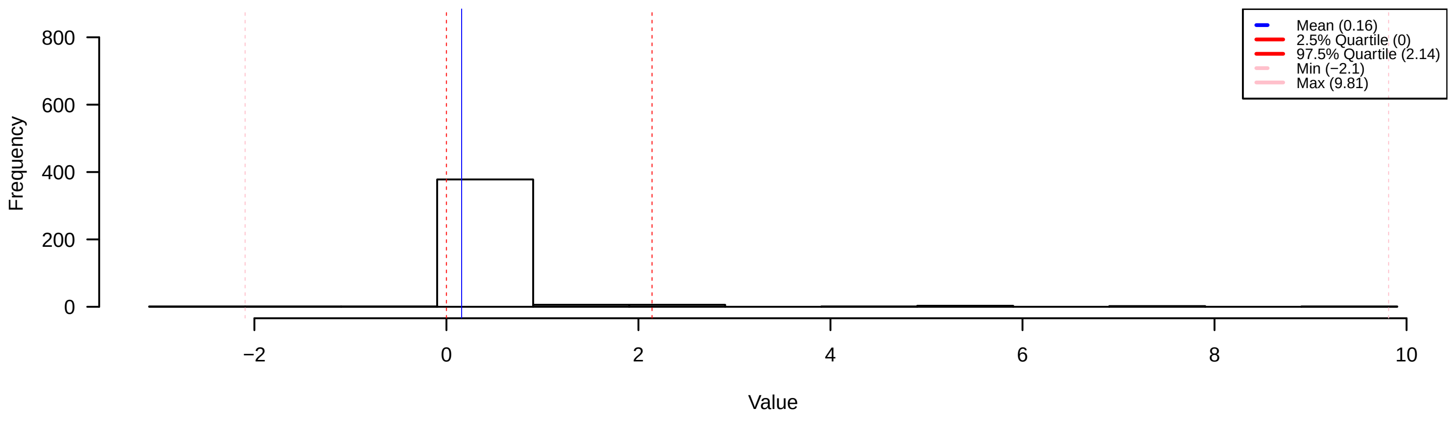

S5, Figure 383 : Bootstrap Distribution of Air Temperature:Absolute Humidity Squared lag 2

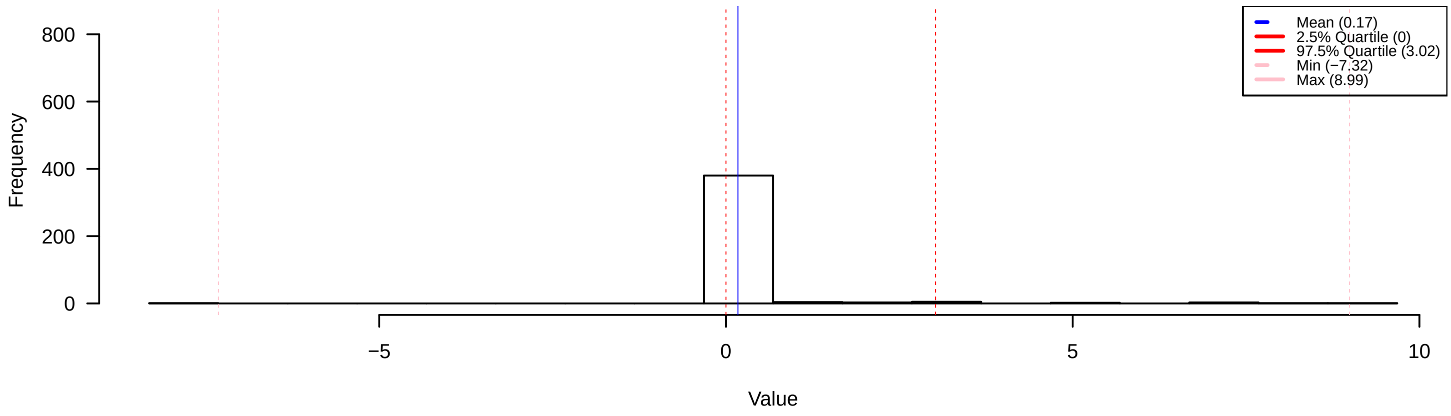

S5, Figure 384 : Bootstrap Distribution of Air Temperature:Absolute Humidity Squared lag 3

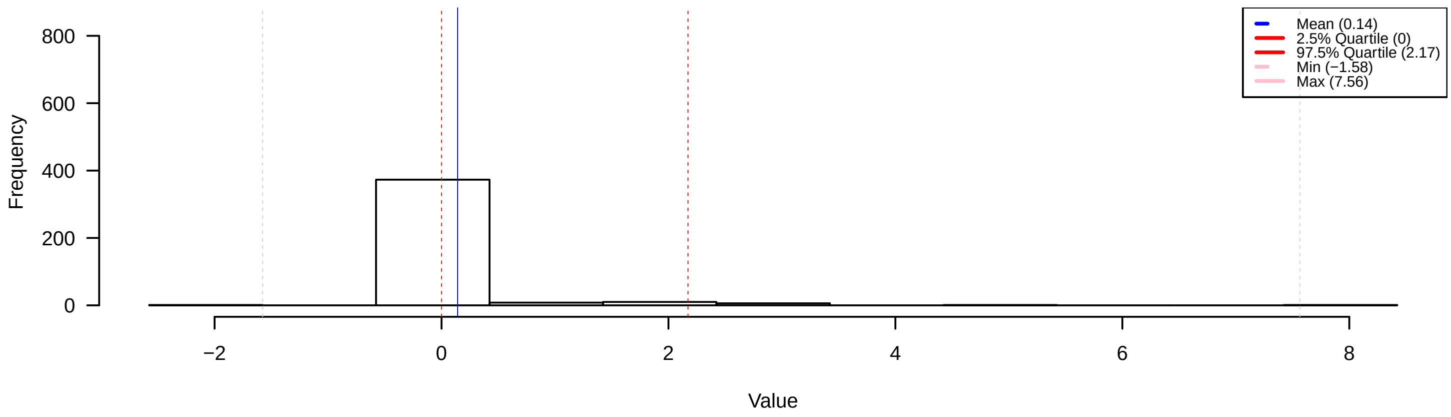

S5, Figure 385 : Bootstrap Distribution of Air Temperature:Absolute Humidity Squared lag 4

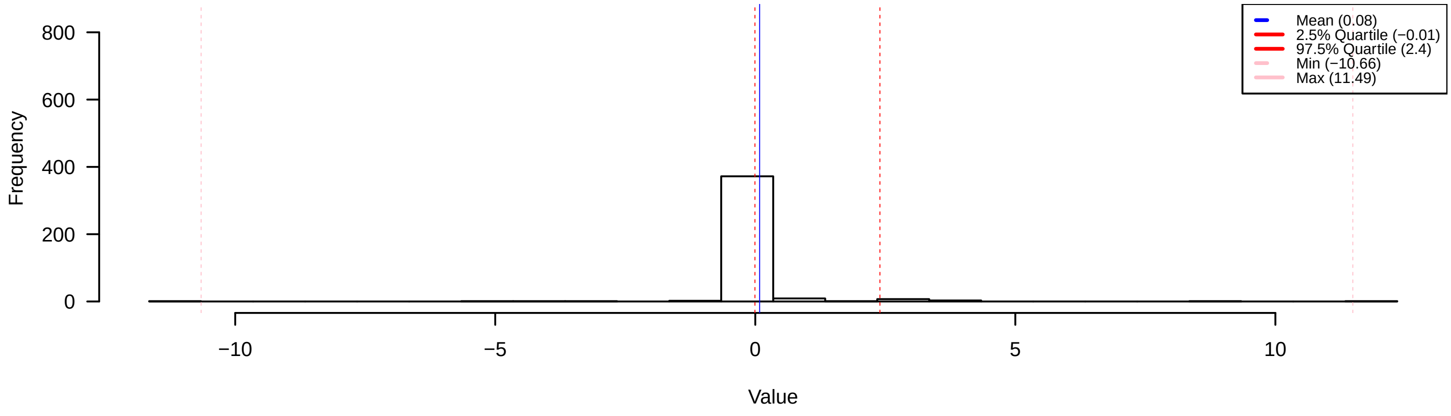

S5, Figure 386 : Bootstrap Distribution of Air Temperature:Absolute Humidity Squared lag 5

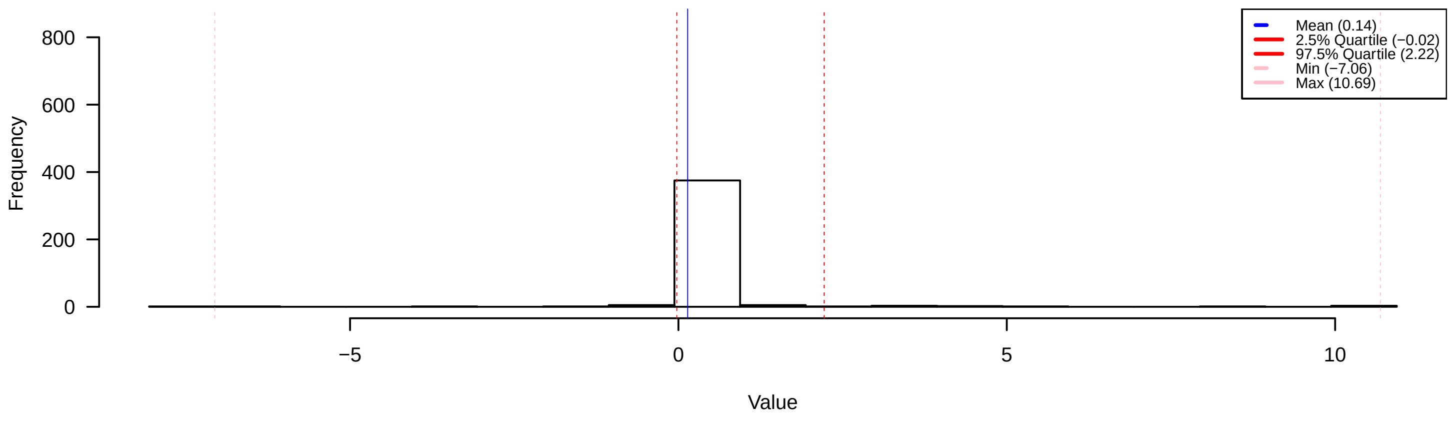

S5, Figure 387 : Bootstrap Distribution of Air Temperature:Absolute Humidity Squared lag 6

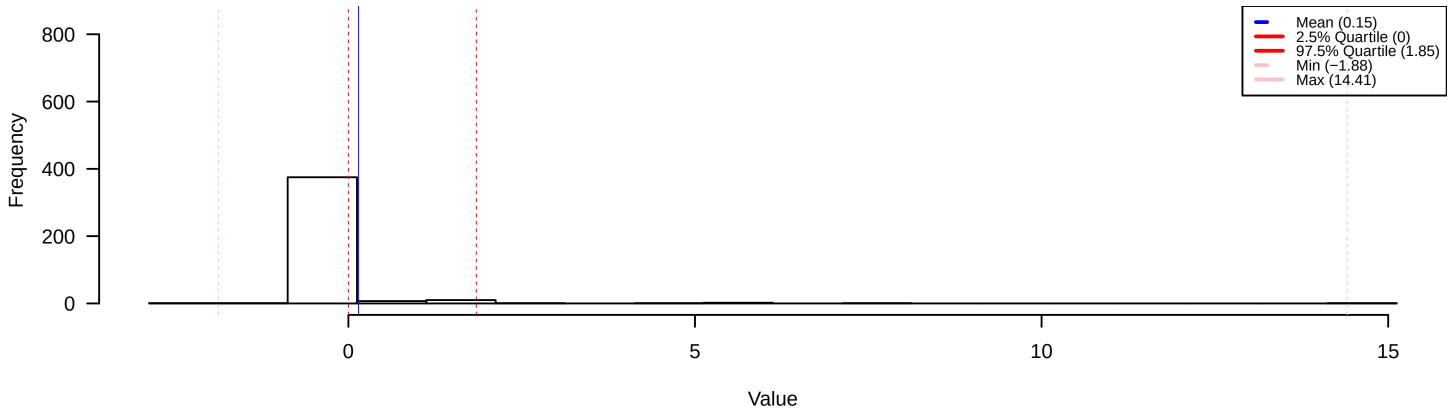

S5, Figure 388 : Bootstrap Distribution of Air Temperature:Absolute Humidity Squared lag 7

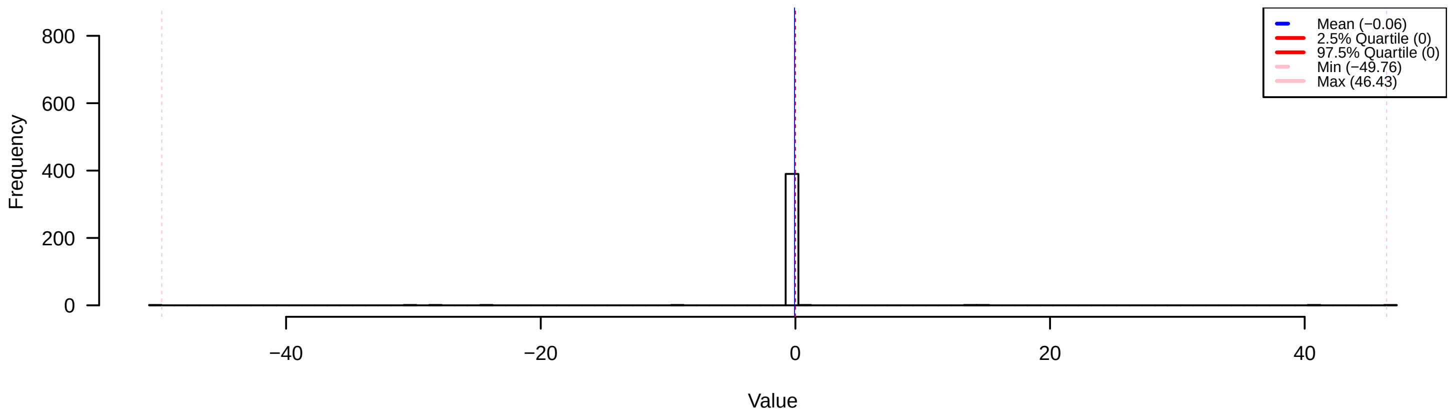

S5, Figure 389 : Bootstrap Distribution of Air Temperature:Absolute Humidity Squared lag 8

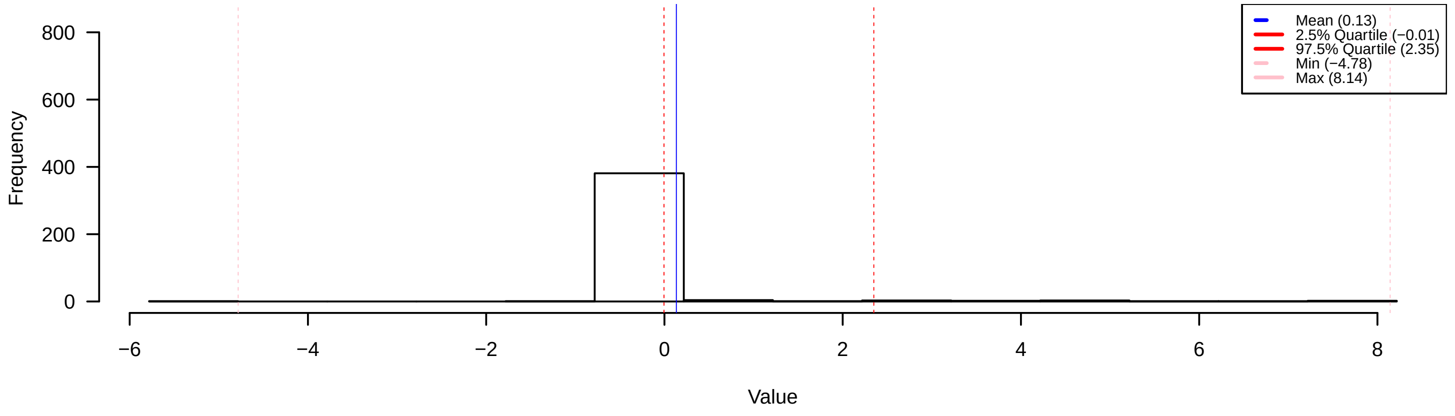

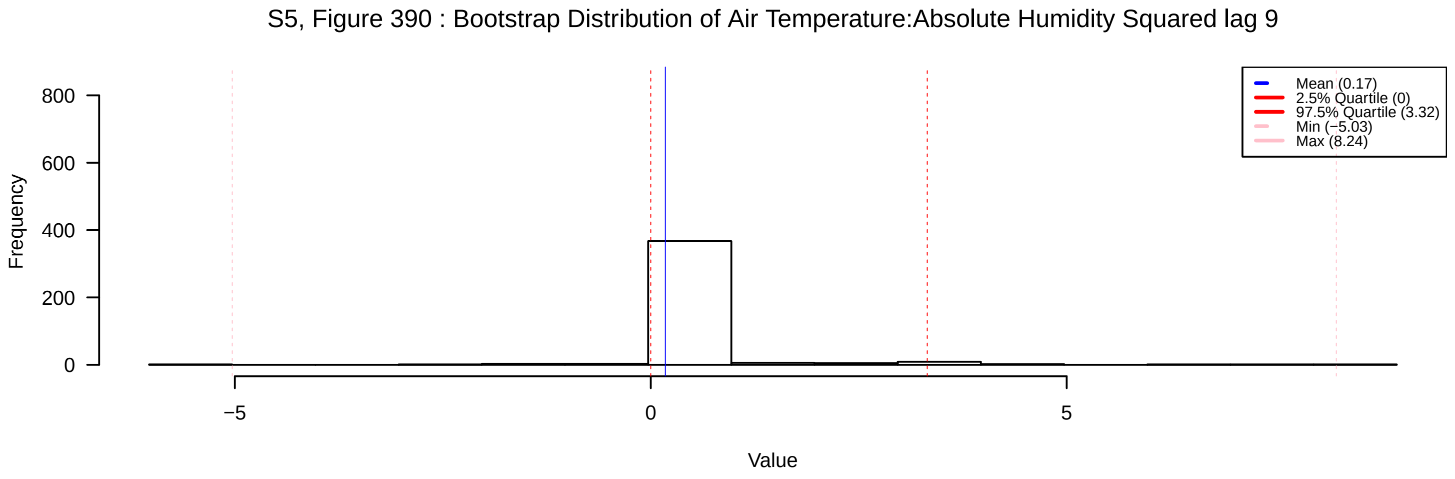
S5, Figure 390 : Bootstrap Distribution of Air Temperature:Absolute Humidity Squared lag 9
Mean (0.17)
2.5% Quartile (0)
97.5% Quartile (3.32)
Min (−5.03)
Max (8.24)
Frequency
Value

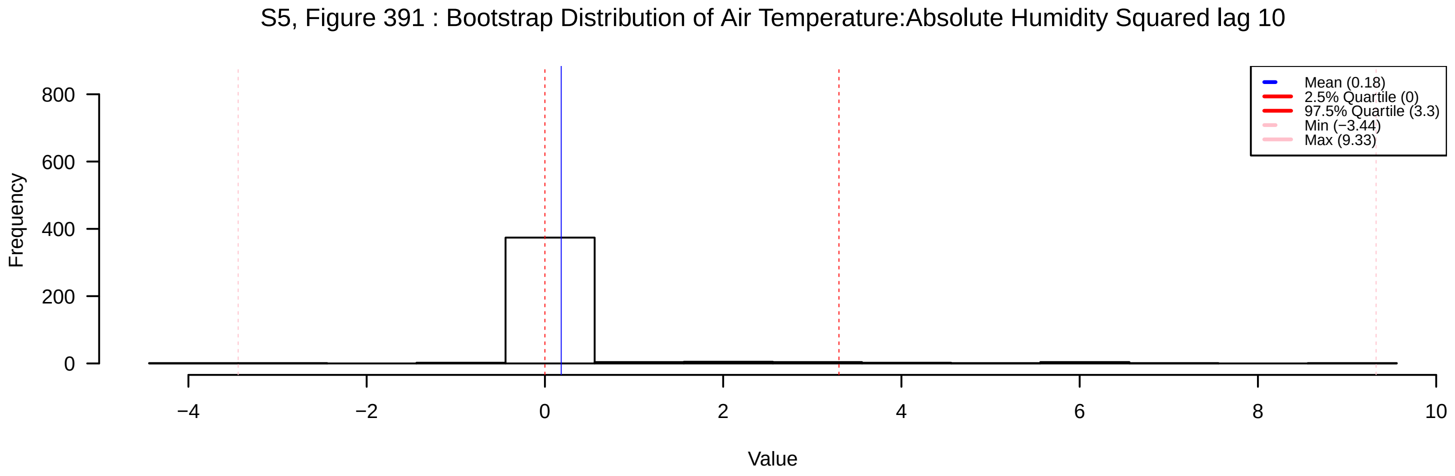
S5, Figure 391 : Bootstrap Distribution of Air Temperature:Absolute Humidity Squared lag 10
Mean (0.18)
2.5% Quartile (0)
97.5% Quartile (3.3)
Min (−3.44)
Max (9.33)
Frequency
Value

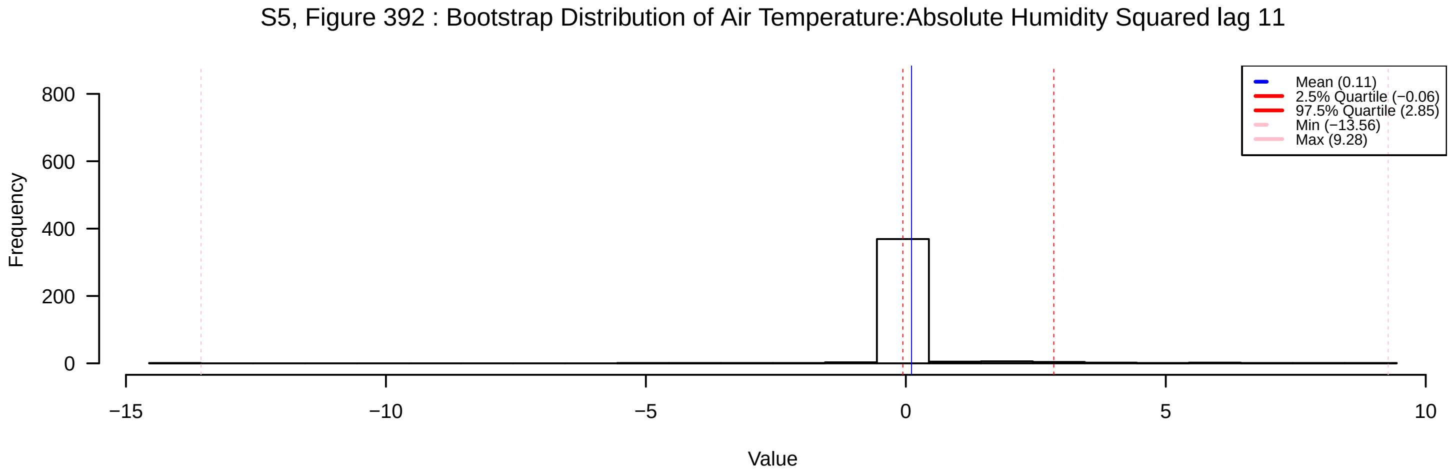
S5, Figure 392 : Bootstrap Distribution of Air Temperature:Absolute Humidity Squared lag 11
Mean (0.11)
2.5% Quartile (−0.06)
97.5% Quartile (2.85)
Min (−13.56)
Max (9.28)
Frequency
Value

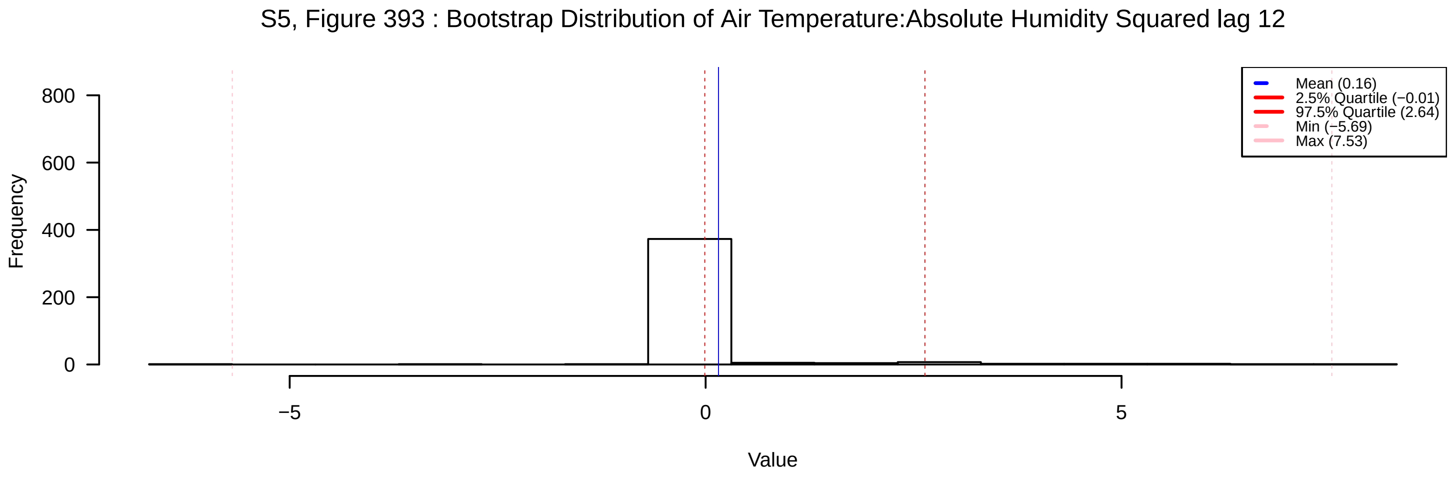
S5, Figure 393 : Bootstrap Distribution of Air Temperature:Absolute Humidity Squared lag 12
Mean (0.16)
2.5% Quartile (−0.01)
97.5% Quartile (2.64)
Min (−5.69)
Max (7.53)
Frequency
Value

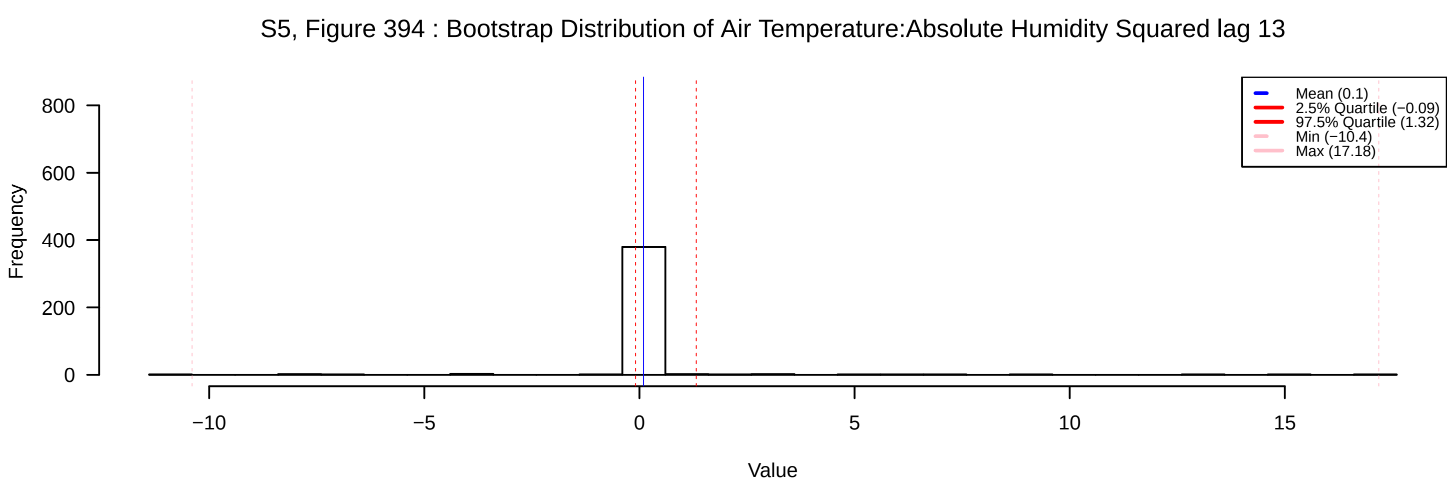
S5, Figure 394 : Bootstrap Distribution of Air Temperature:Absolute Humidity Squared lag 13
Frequency
Value
Mean (0.1)
2.5% Quartile (−0.09)
97.5% Quartile (1.32)
Min (−10.4)
Max (17.18)

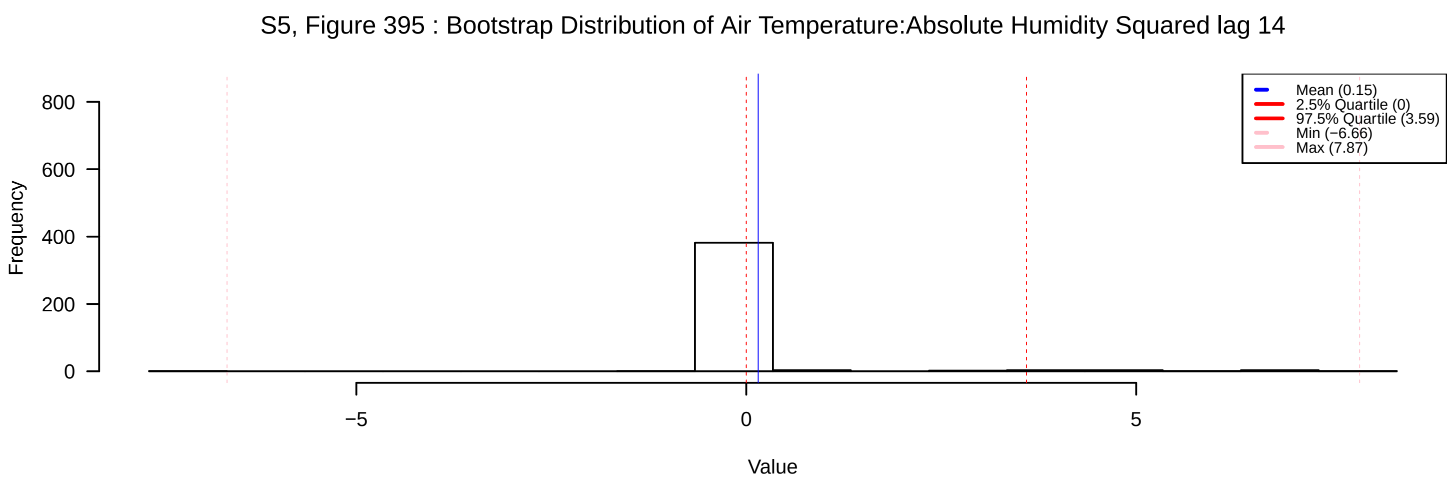
S5, Figure 395 : Bootstrap Distribution of Air Temperature:Absolute Humidity Squared lag 14
Frequency
Value
Mean (0.15)
2.5% Quartile (0)
97.5% Quartile (3.59)
Min (−6.66)
Max (7.87)

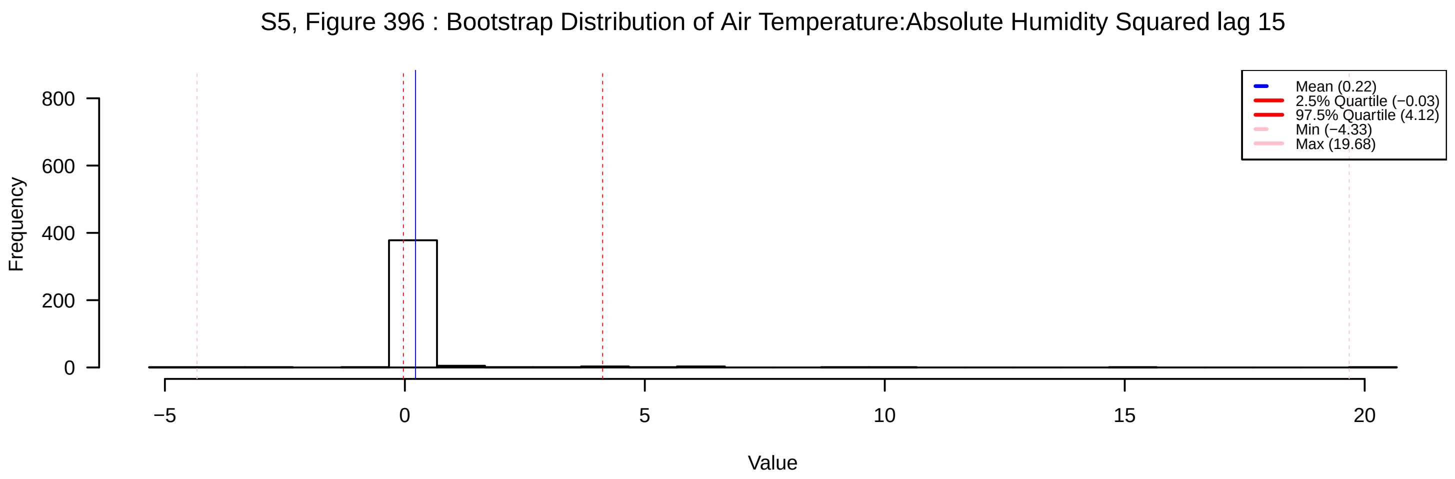
S5, Figure 396 : Bootstrap Distribution of Air Temperature:Absolute Humidity Squared lag 15
Frequency
Value
Mean (0.22)
2.5% Quartile (−0.03)
97.5% Quartile (4.12)
Min (−4.33)
Max (19.68)

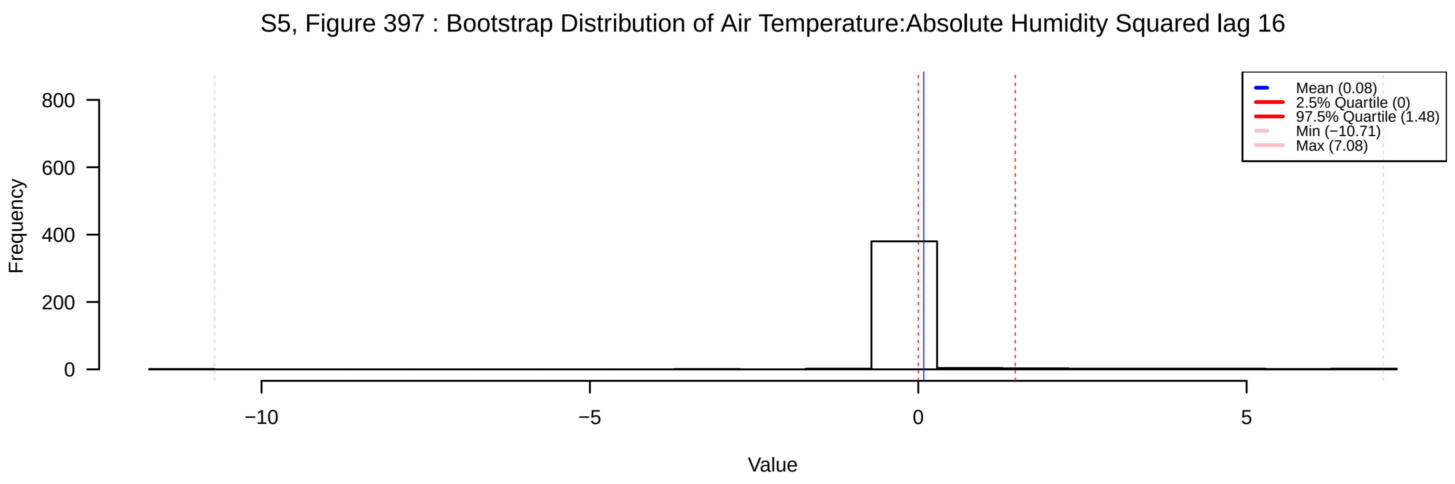
S5, Figure 397 : Bootstrap Distribution of Air Temperature:Absolute Humidity Squared lag 16
Frequency
Value
Mean (0.08)
2.5% Quartile (0)
97.5% Quartile (1.48)
Min (−10.71)
Max (7.08)

S5, Figure 398 : Bootstrap Distribution of Air Temperature:Absolute Humidity Squared lag 17

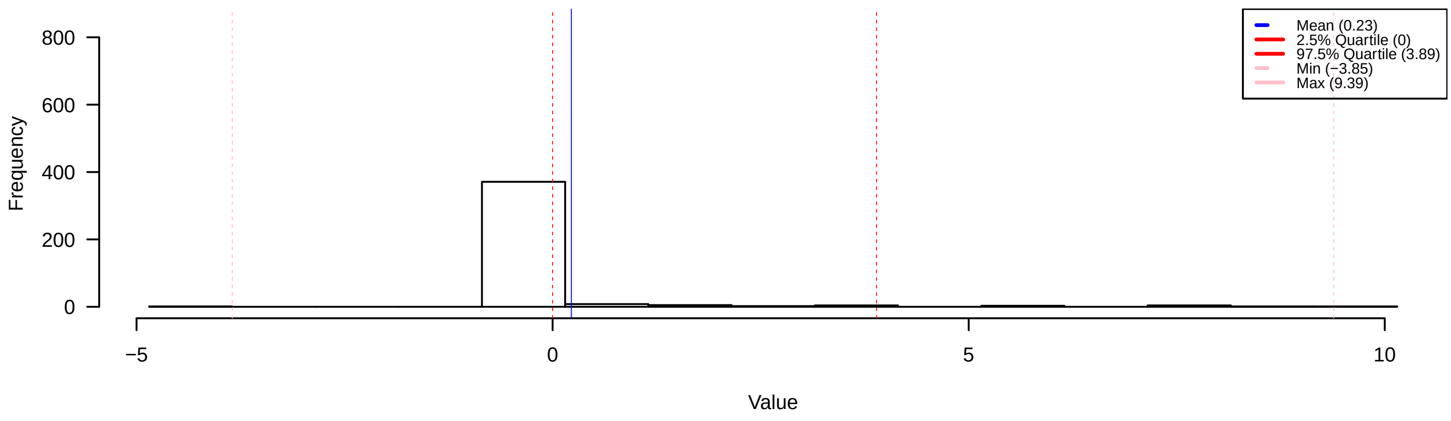

S5, Figure 399 : Bootstrap Distribution of Air Temperature:Absolute Humidity Squared lag 18

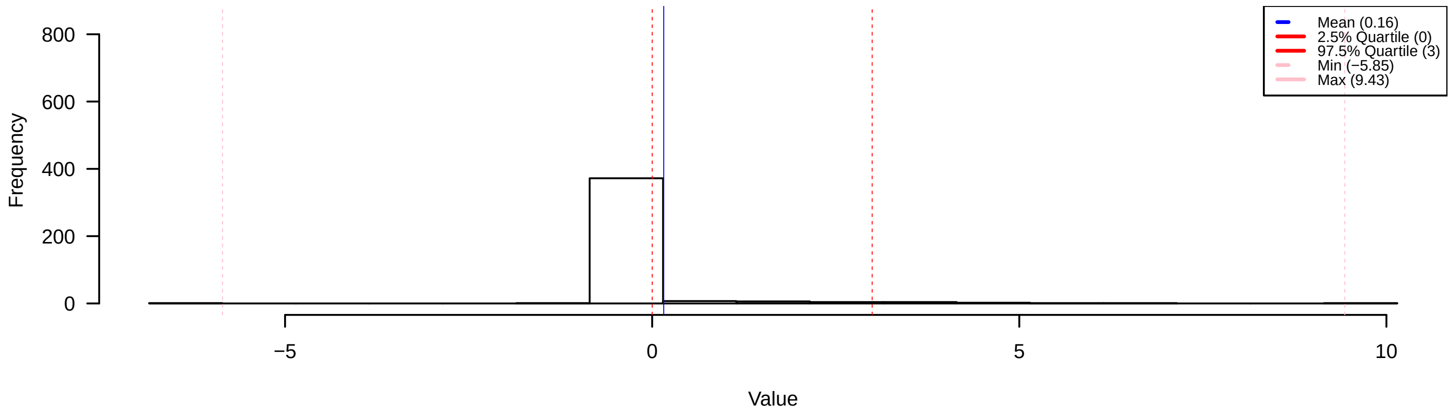

S5, Figure 400 : Bootstrap Distribution of Air Temperature:Absolute Humidity Squared lag 19

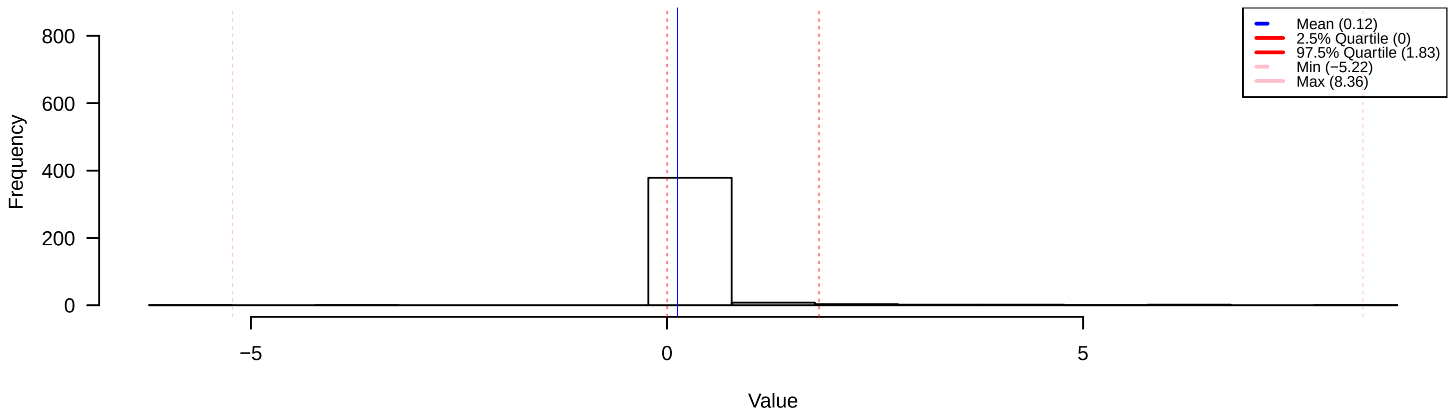

S5, Figure 401 : Bootstrap Distribution of Air Temperature:Absolute Humidity Squared lag 20

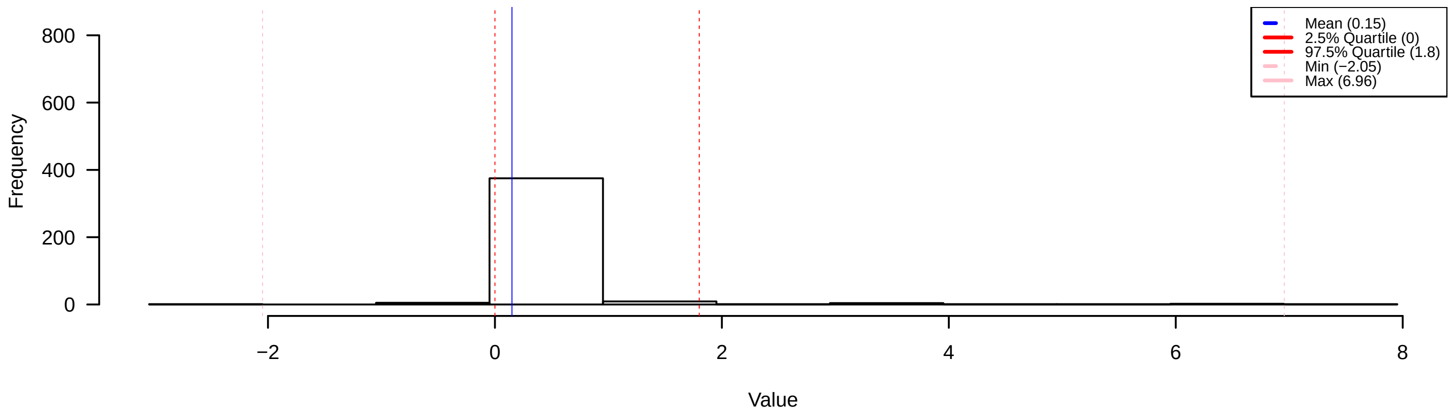

S5, Figure 402 : Bootstrap Distribution of Air Temperature:Relative Humidity Squared lag 1

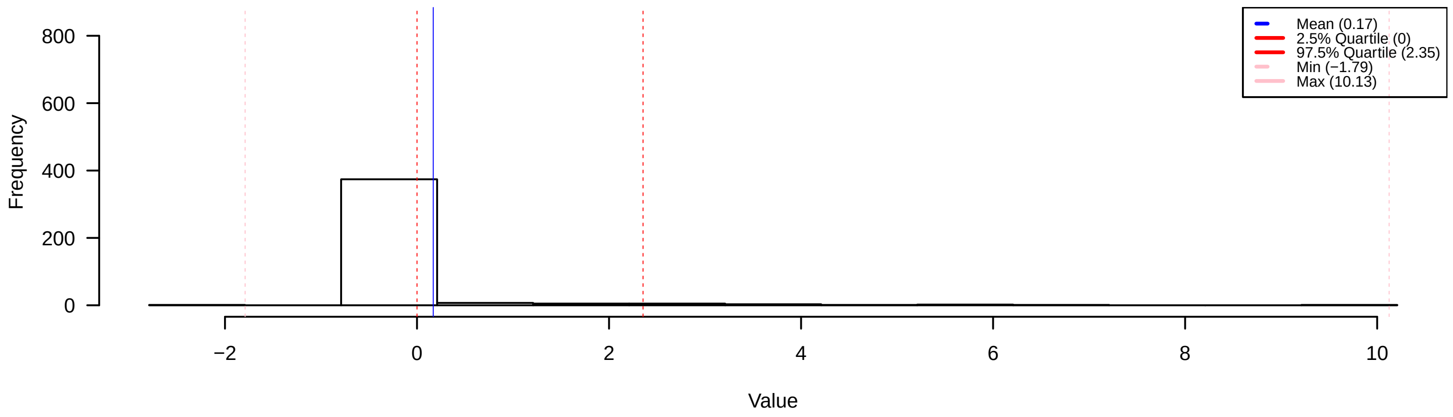

S5, Figure 403 : Bootstrap Distribution of Air Temperature:Relative Humidity Squared lag 2

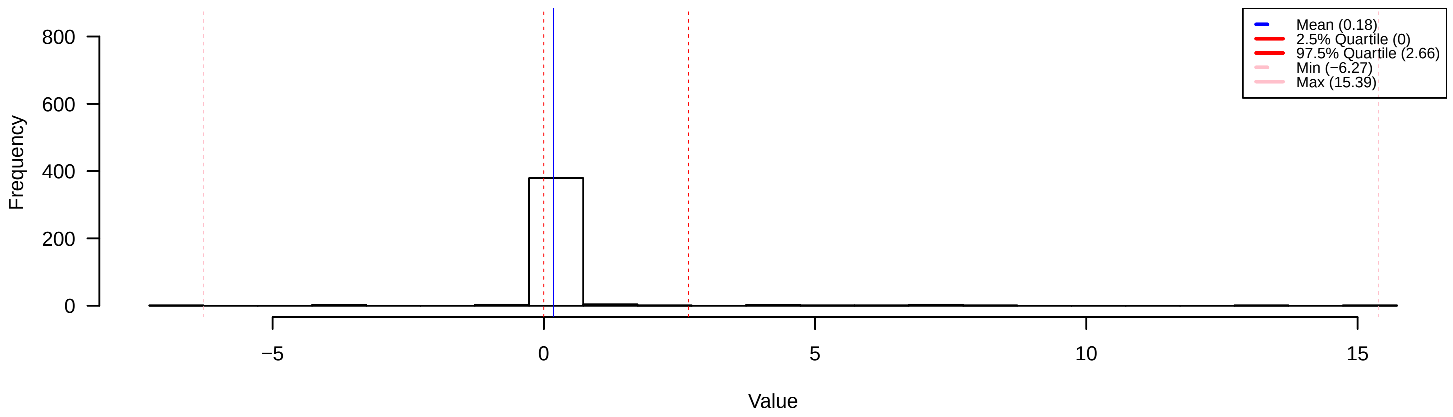

S5, Figure 404 : Bootstrap Distribution of Air Temperature:Relative Humidity Squared lag 3

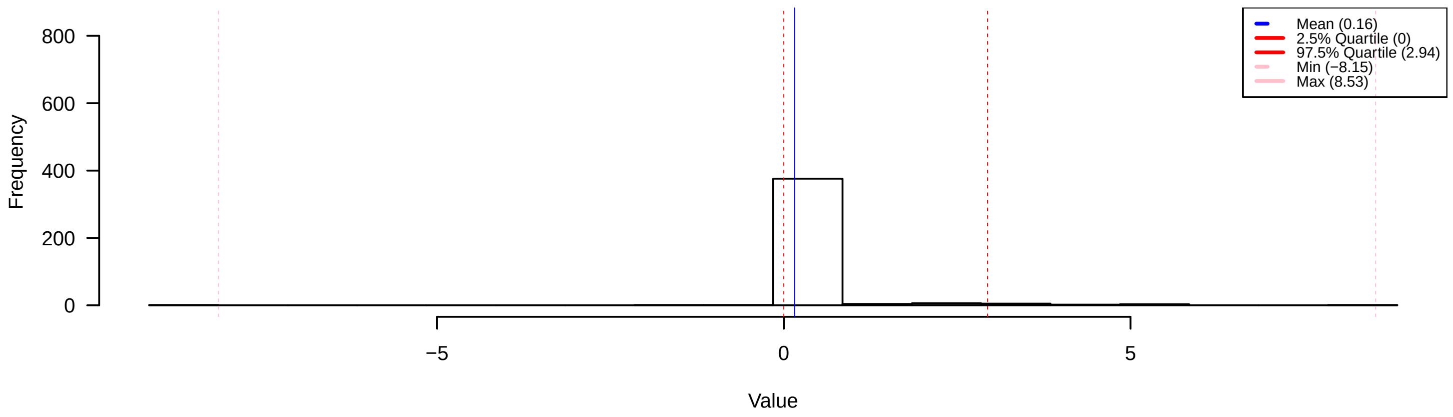

S5, Figure 405 : Bootstrap Distribution of Air Temperature:Relative Humidity Squared lag 4

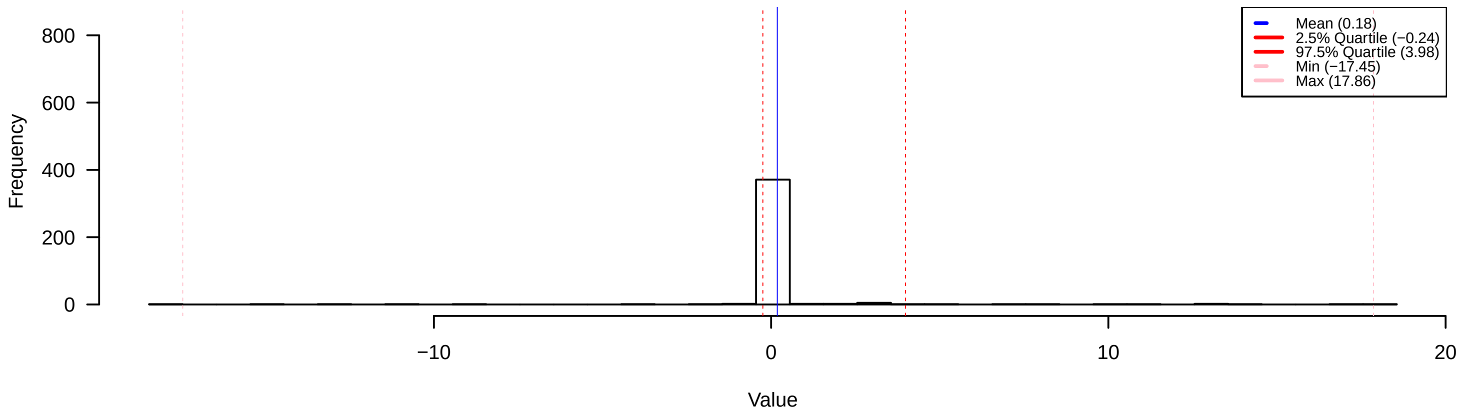

S5, Figure 406 : Bootstrap Distribution of Air Temperature:Relative Humidity Squared lag 5

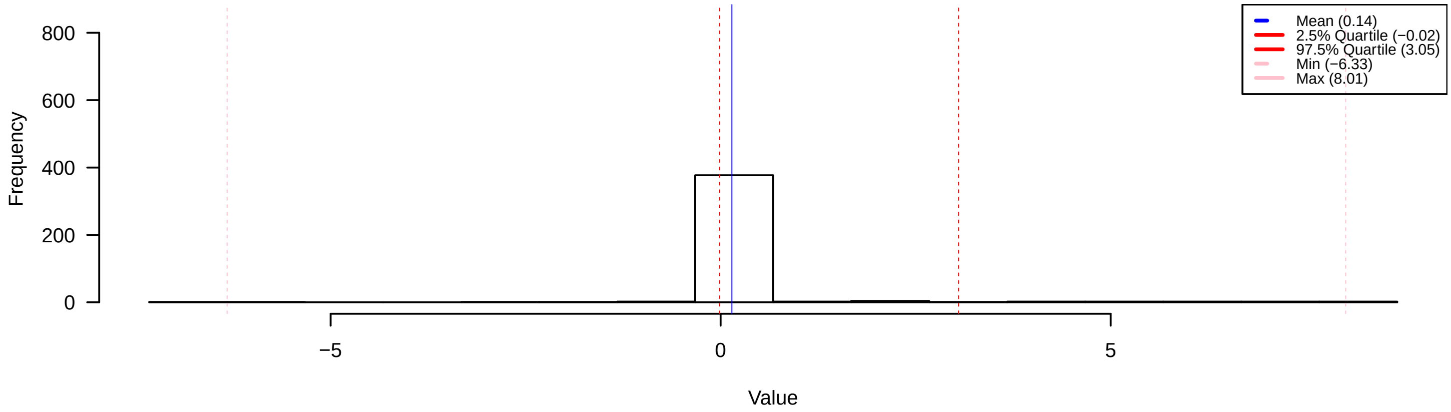

S5, Figure 407 : Bootstrap Distribution of Air Temperature:Relative Humidity Squared lag 6

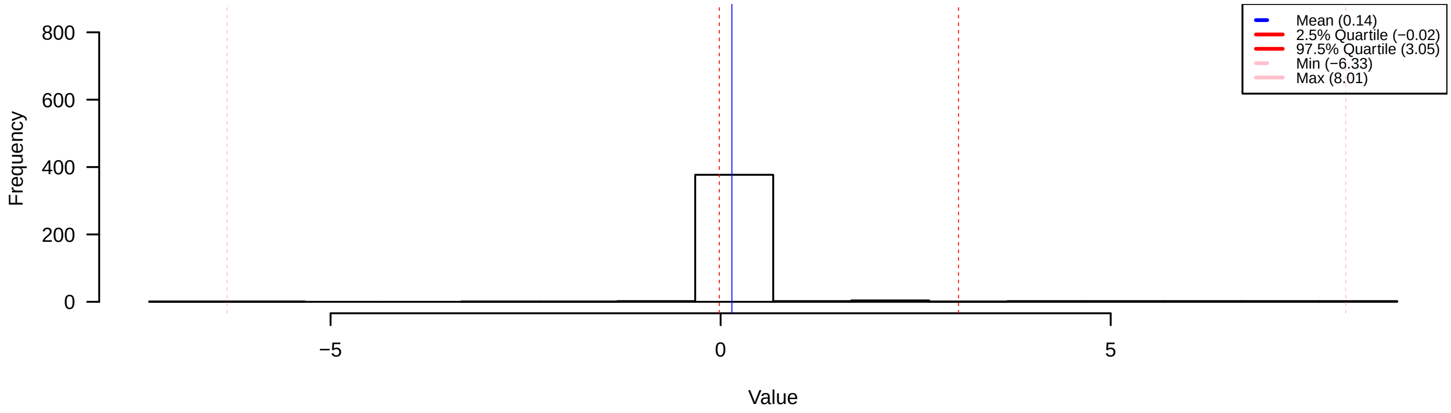

S5, Figure 408 : Bootstrap Distribution of Air Temperature:Relative Humidity Squared lag 7

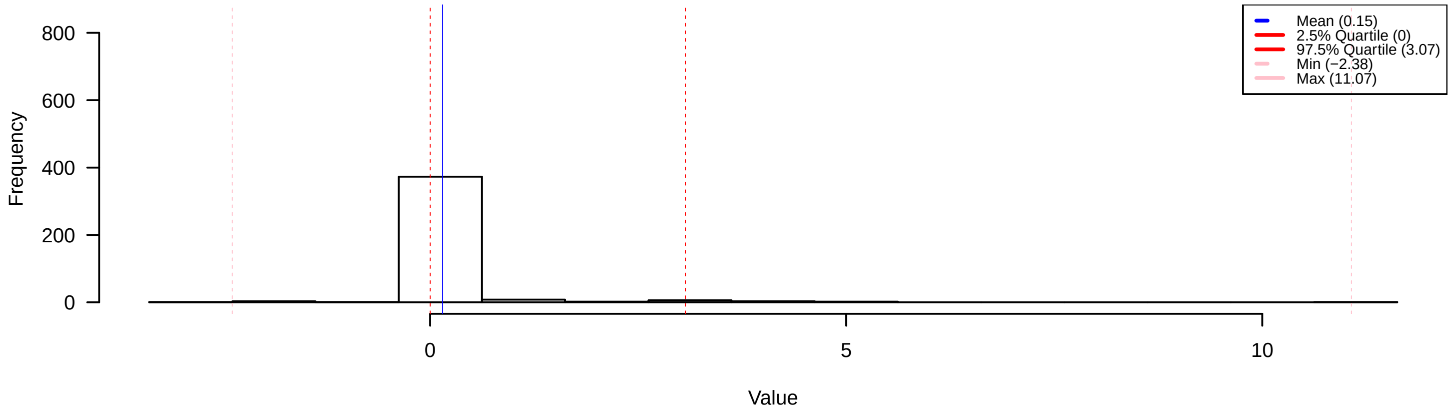

S5, Figure 409 : Bootstrap Distribution of Air Temperature:Relative Humidity Squared lag 8

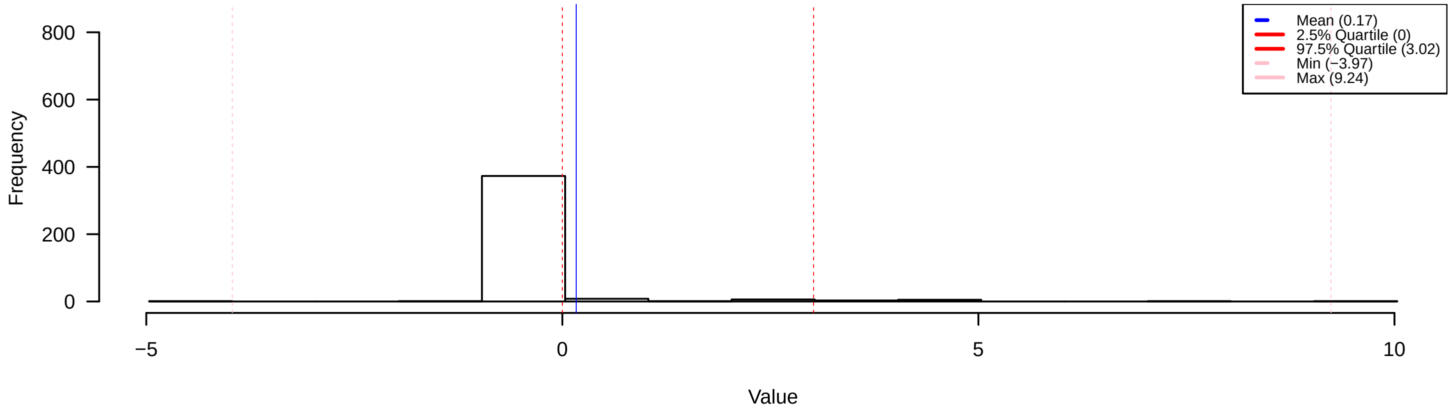

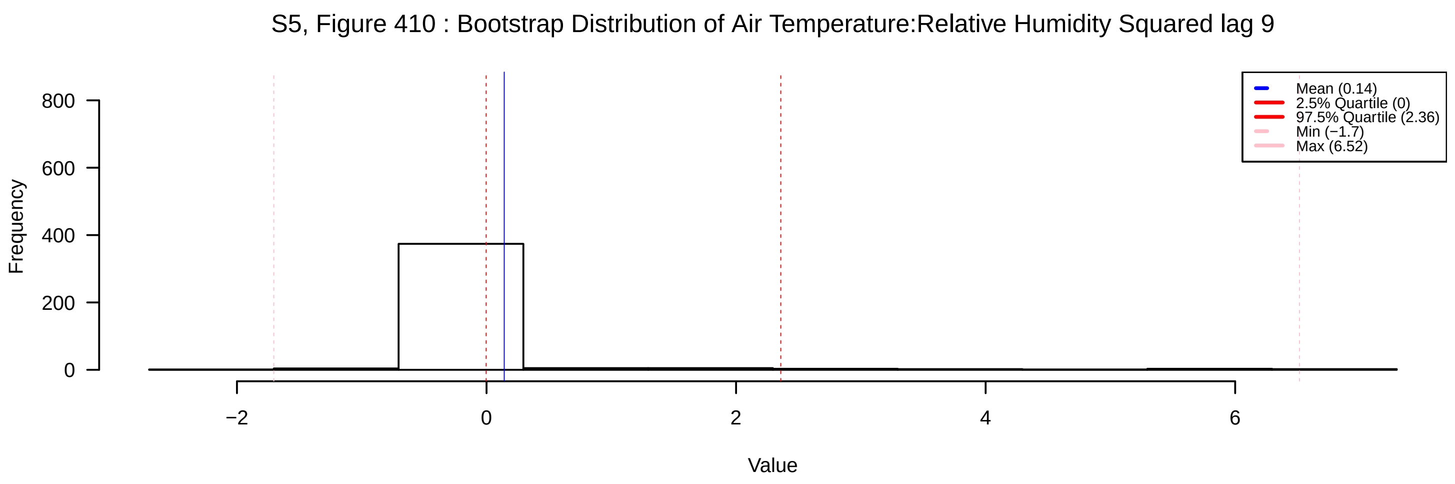
S5, Figure 410 : Bootstrap Distribution of Air Temperature:Relative Humidity Squared lag 9
Mean (0.14)
2.5% Quartile (0)
97.5% Quartile (2.36)
Min (−1.7)
Max (6.52)
Frequency
Value

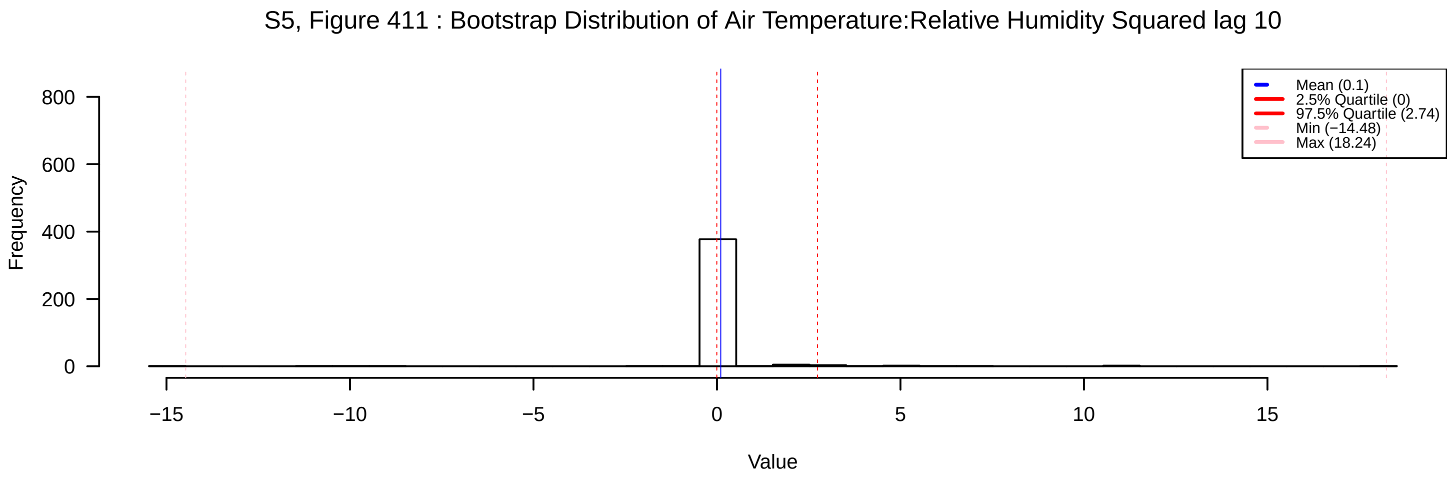
S5, Figure 411 : Bootstrap Distribution of Air Temperature:Relative Humidity Squared lag 10
Mean (0.1)
2.5% Quartile (0)
97.5% Quartile (2.74)
Min (−14.48)
Max (18.24)
Frequency
Value

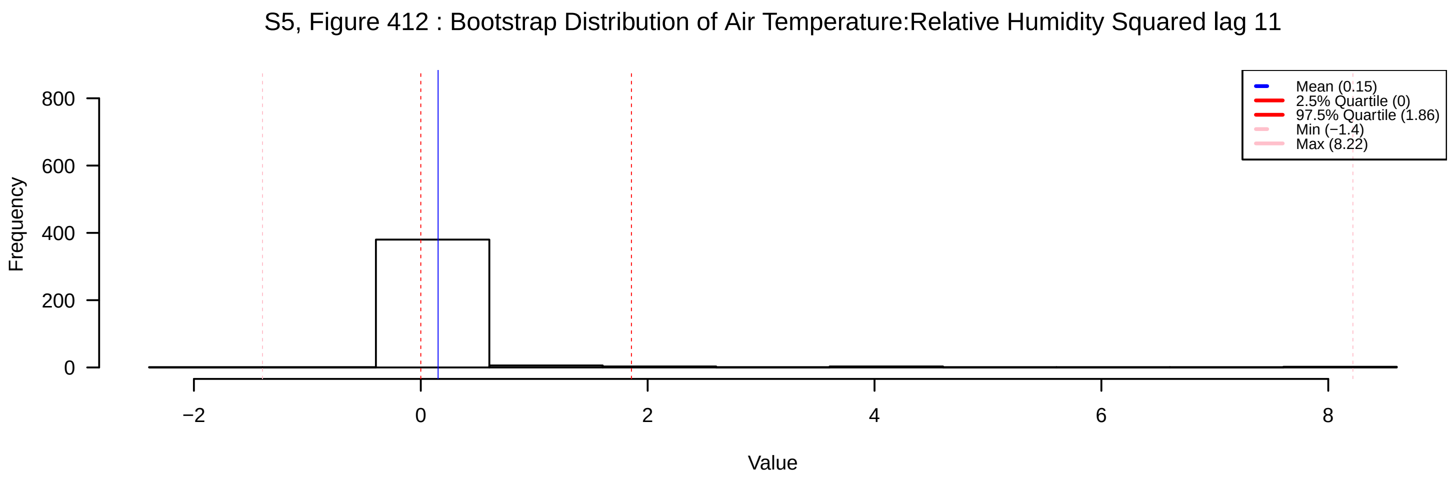
S5, Figure 412 : Bootstrap Distribution of Air Temperature:Relative Humidity Squared lag 11
Mean (0.15)
2.5% Quartile (0)
97.5% Quartile (1.86)
Min (−1.4)
Max (8.22)
Frequency
Value

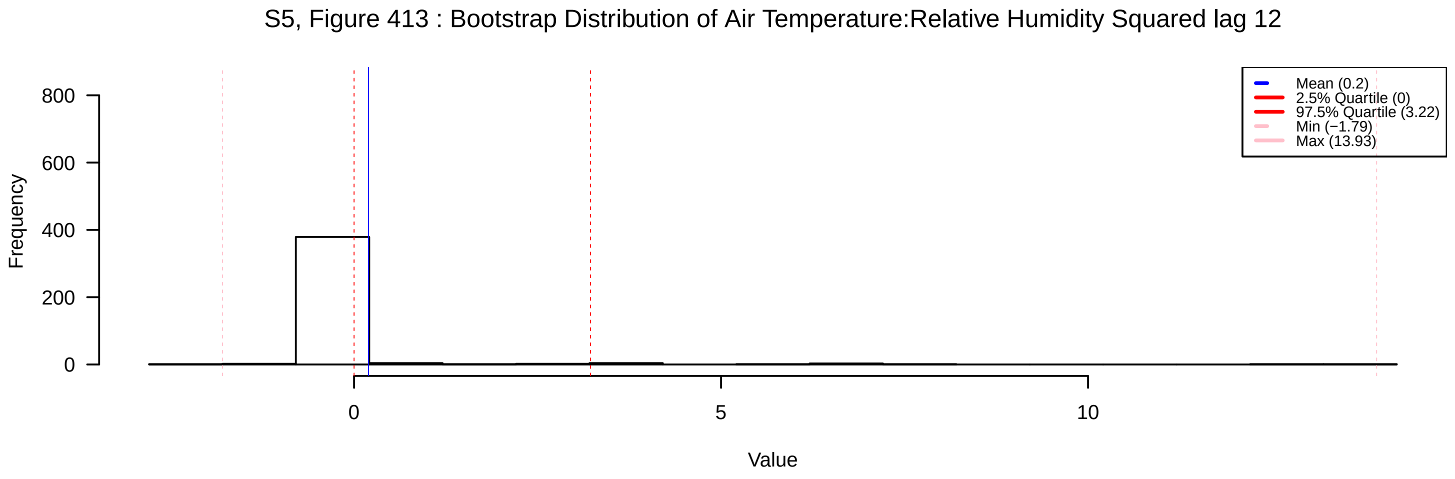
S5, Figure 413 : Bootstrap Distribution of Air Temperature:Relative Humidity Squared lag 12
Mean (0.2)
2.5% Quartile (0)
97.5% Quartile (3.22)
Min (−1.79)
Max (13.93)
Frequency
Value

S5, Figure 414 : Bootstrap Distribution of Air Temperature:Relative Humidity Squared lag 13

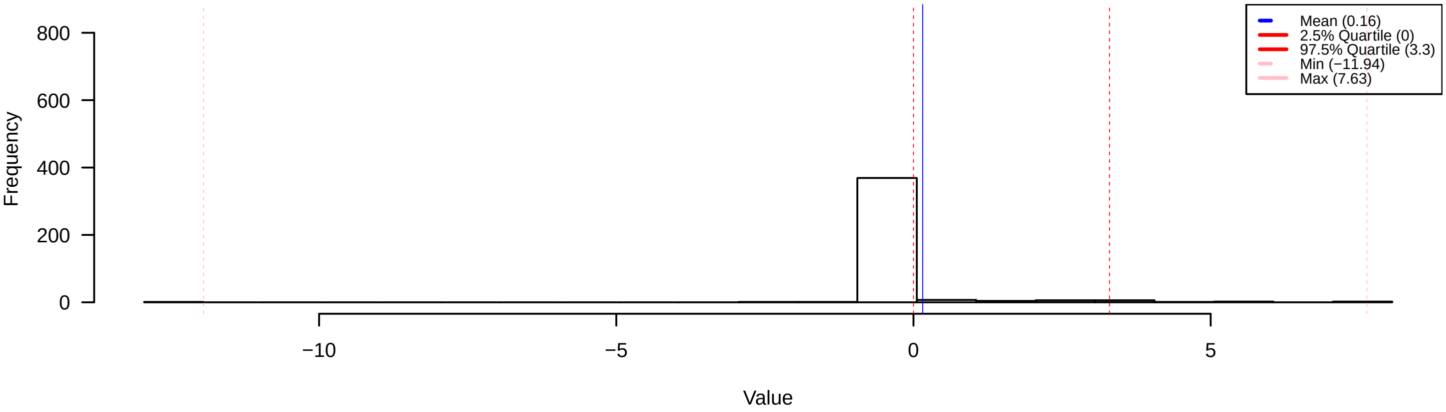

S5, Figure 415 : Bootstrap Distribution of Air Temperature:Relative Humidity Squared lag 14

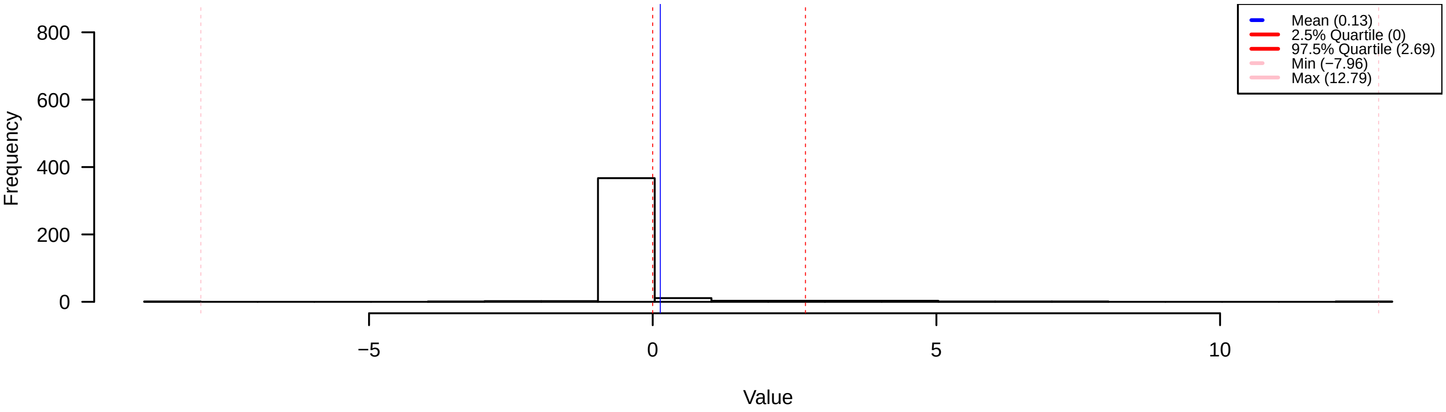

S5, Figure 416 : Bootstrap Distribution of Air Temperature:Relative Humidity Squared lag 15

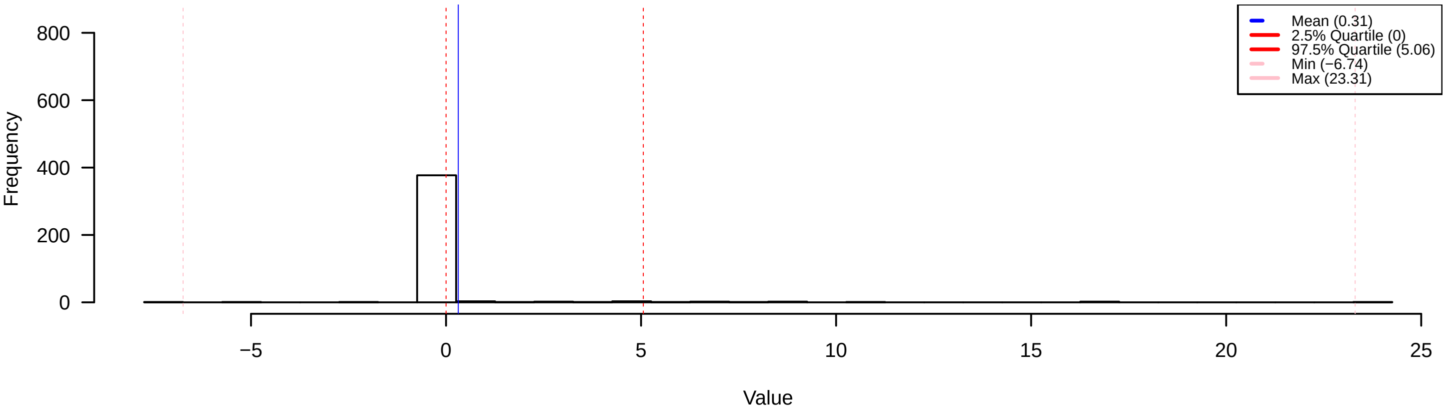

S5, Figure 417 : Bootstrap Distribution of Air Temperature:Relative Humidity Squared lag 16

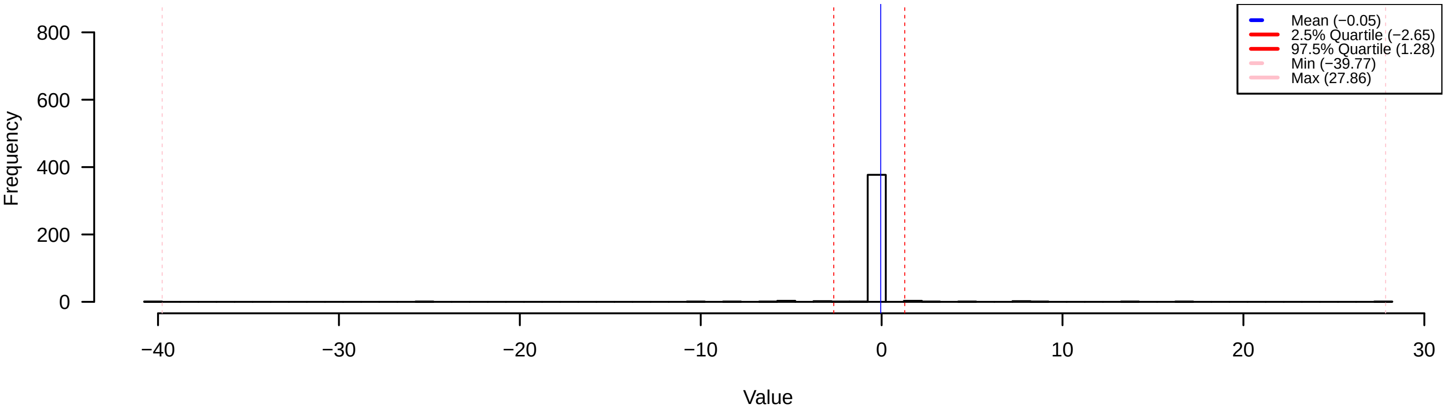

S5, Figure 418 : Bootstrap Distribution of Air Temperature:Relative Humidity Squared lag 17

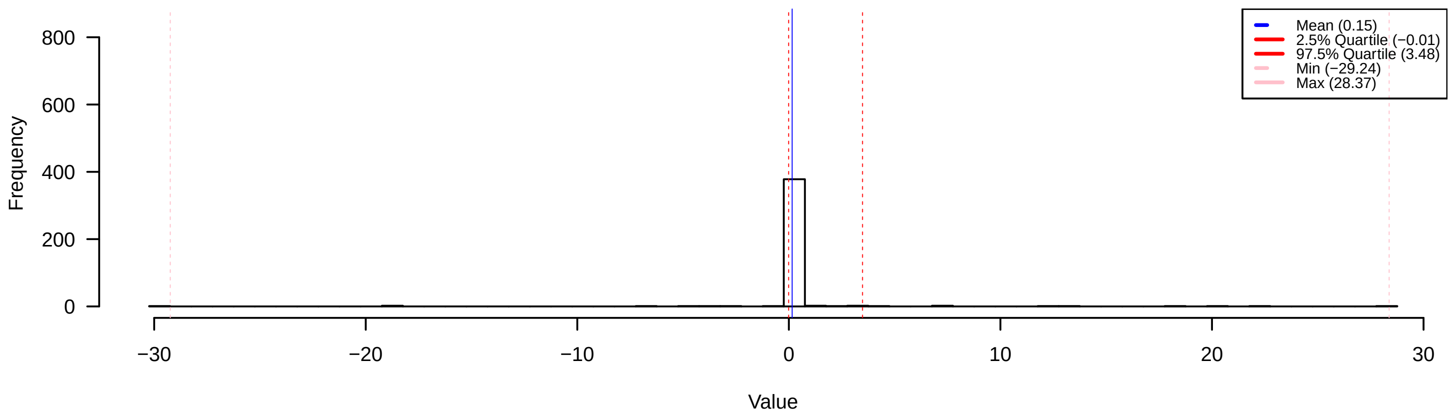

S5, Figure 419 : Bootstrap Distribution of Air Temperature:Relative Humidity Squared lag 18

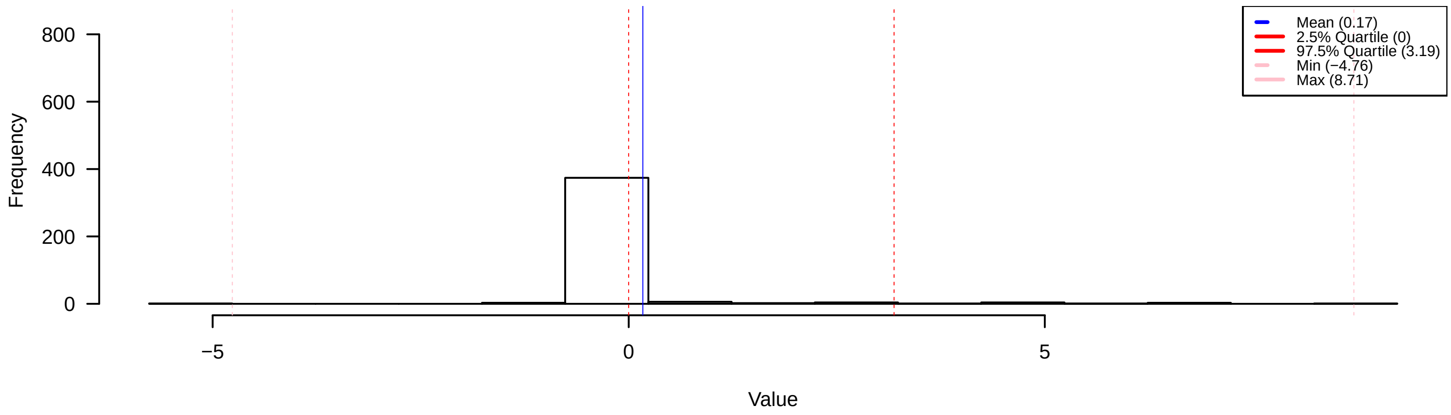

S5, Figure 420 : Bootstrap Distribution of Air Temperature:Relative Humidity Squared lag 19

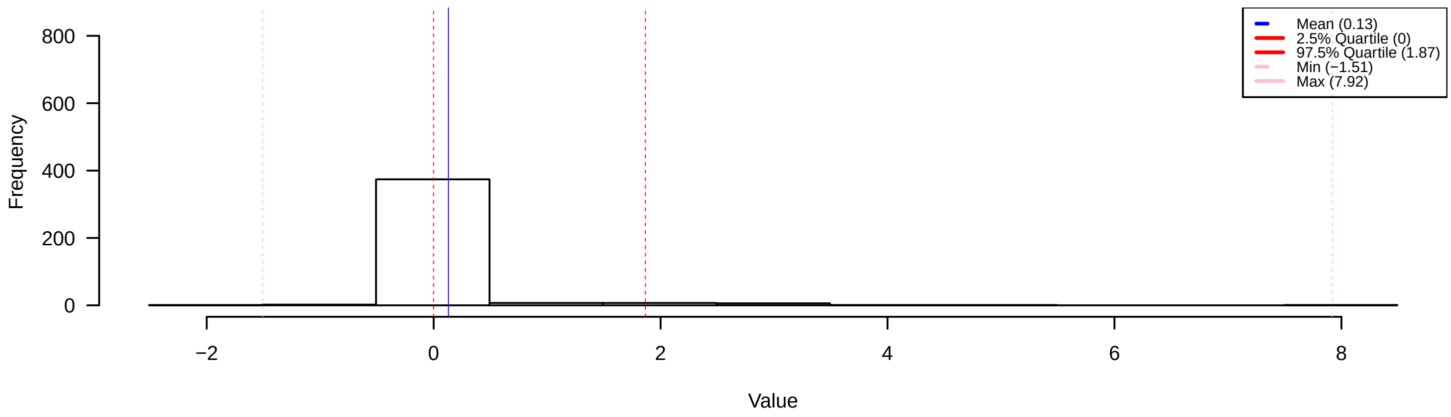

S5, Figure 421 : Bootstrap Distribution of Air Temperature:Relative Humidity Squared lag 20

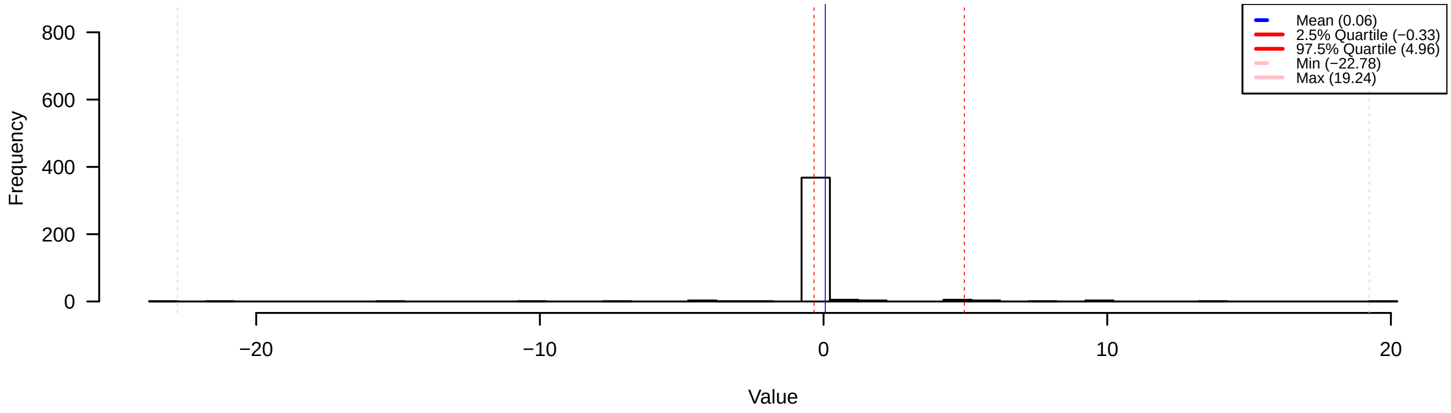

S5, Figure 422 : Bootstrap Distribution of Absolute Humidity:Relative Humidity Squared lag 1

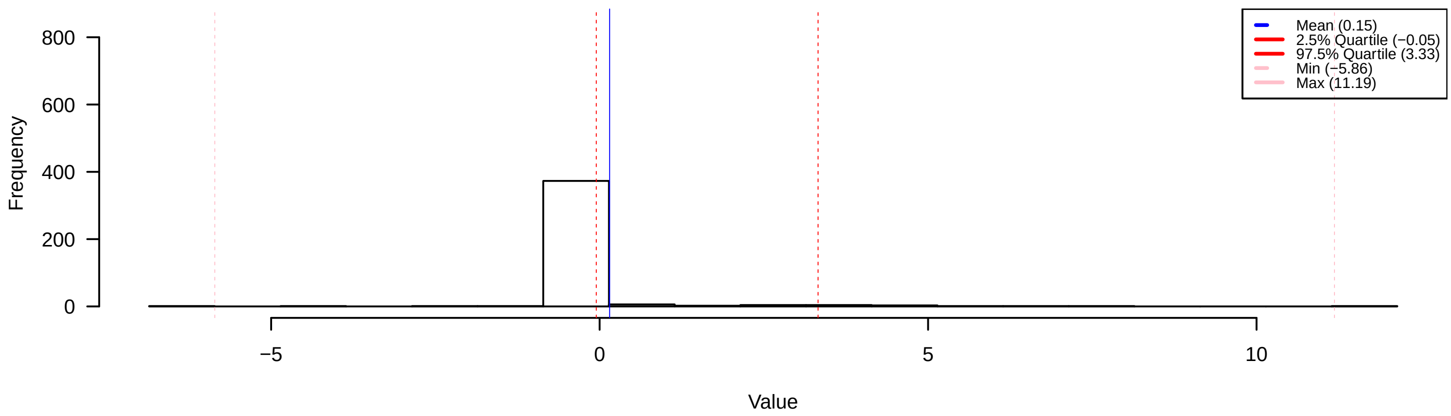

S5, Figure 423 : Bootstrap Distribution of Absolute Humidity:Relative Humidity Squared lag 2

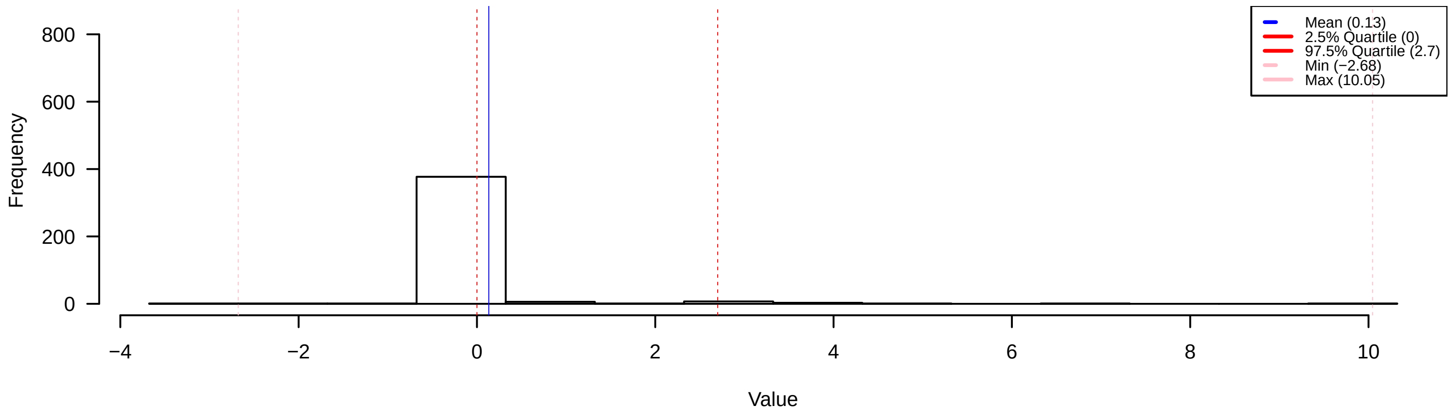

S5, Figure 424 : Bootstrap Distribution of Absolute Humidity:Relative Humidity Squared lag 3

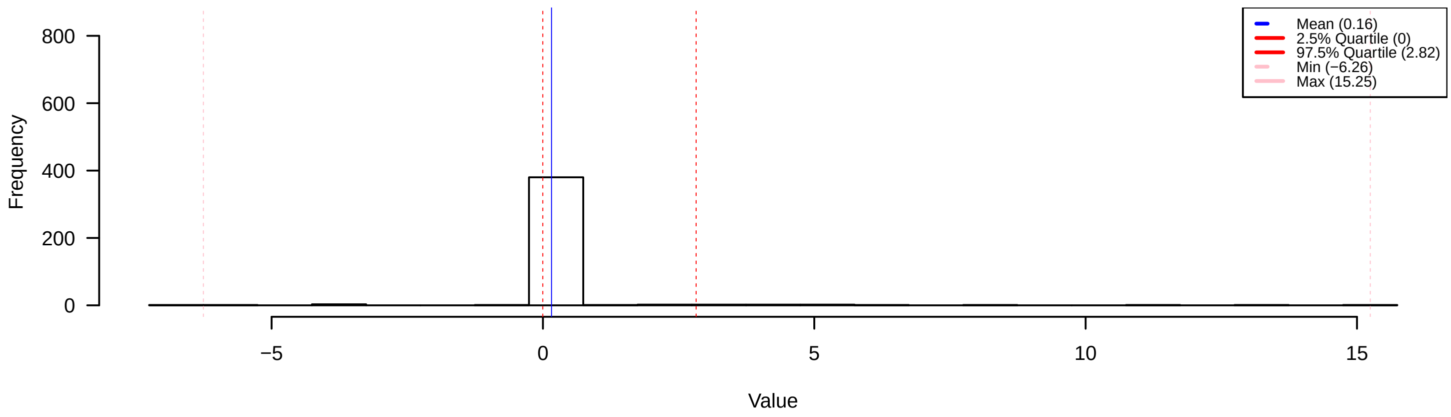

S5, Figure 425 : Bootstrap Distribution of Absolute Humidity:Relative Humidity Squared lag 4

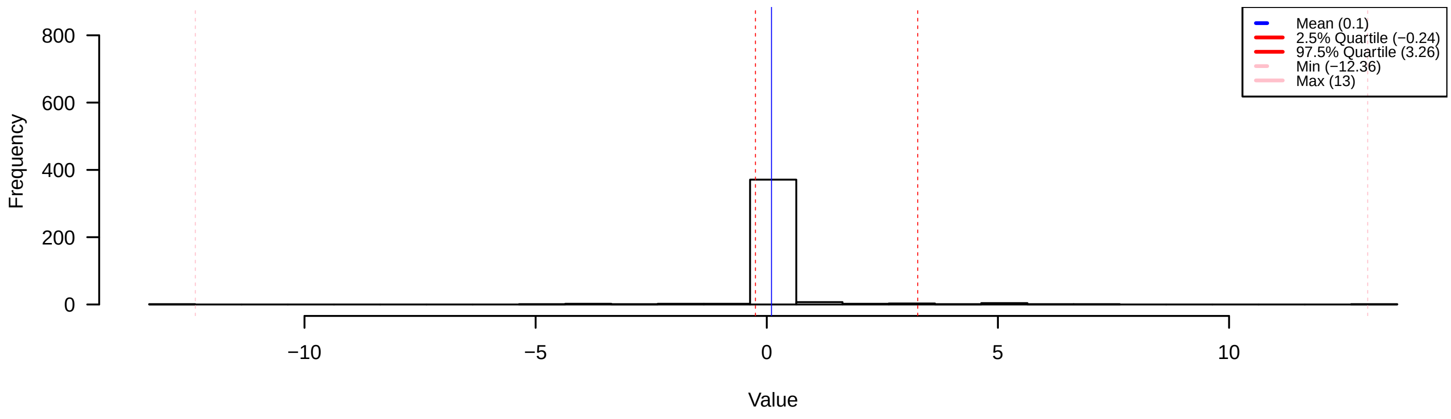

S5, Figure 426 : Bootstrap Distribution of Absolute Humidity:Relative Humidity Squared lag 5

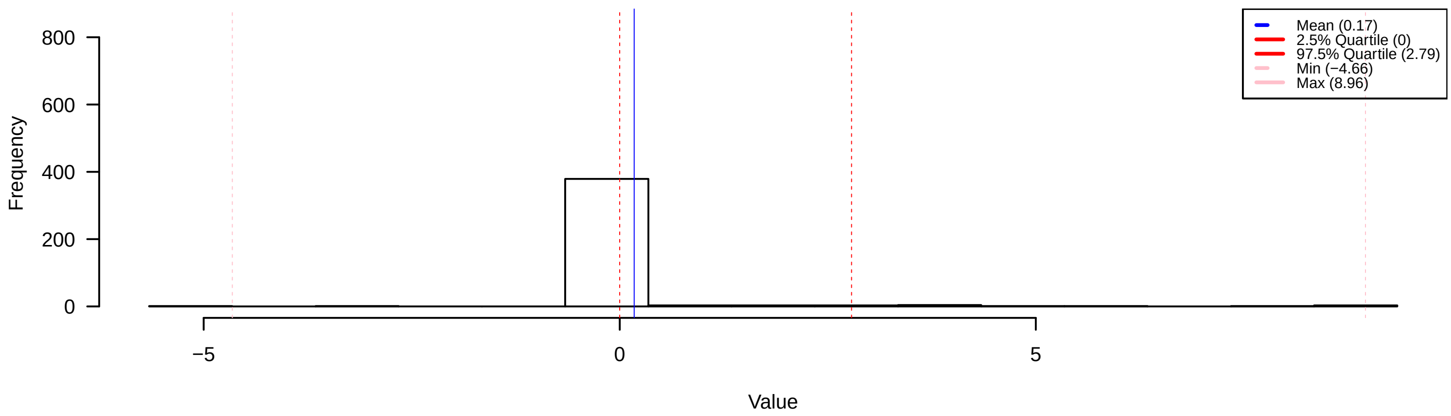

S5, Figure 427 : Bootstrap Distribution of Absolute Humidity:Relative Humidity Squared lag 6

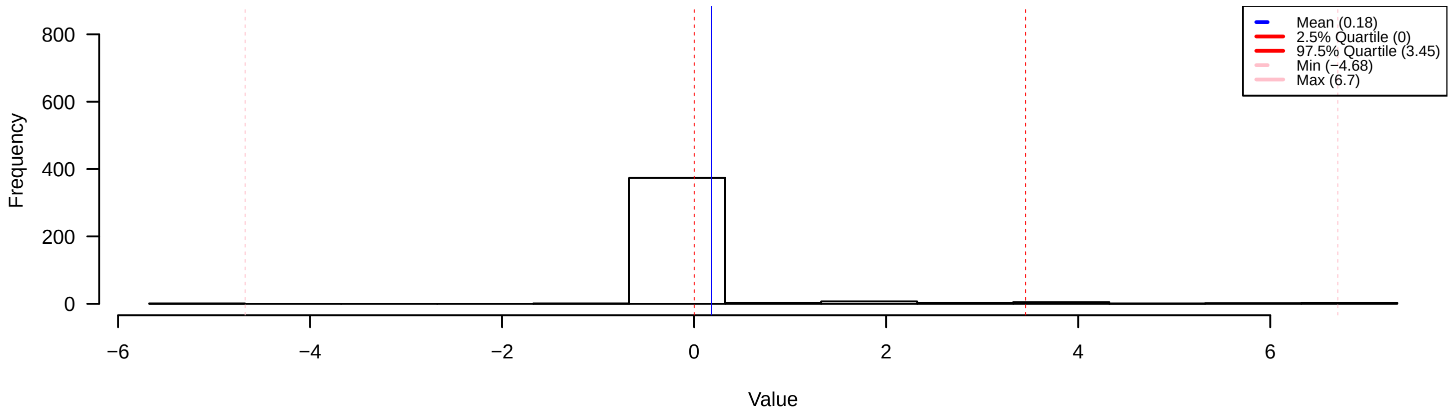

S5, Figure 428 : Bootstrap Distribution of Absolute Humidity:Relative Humidity Squared lag 7

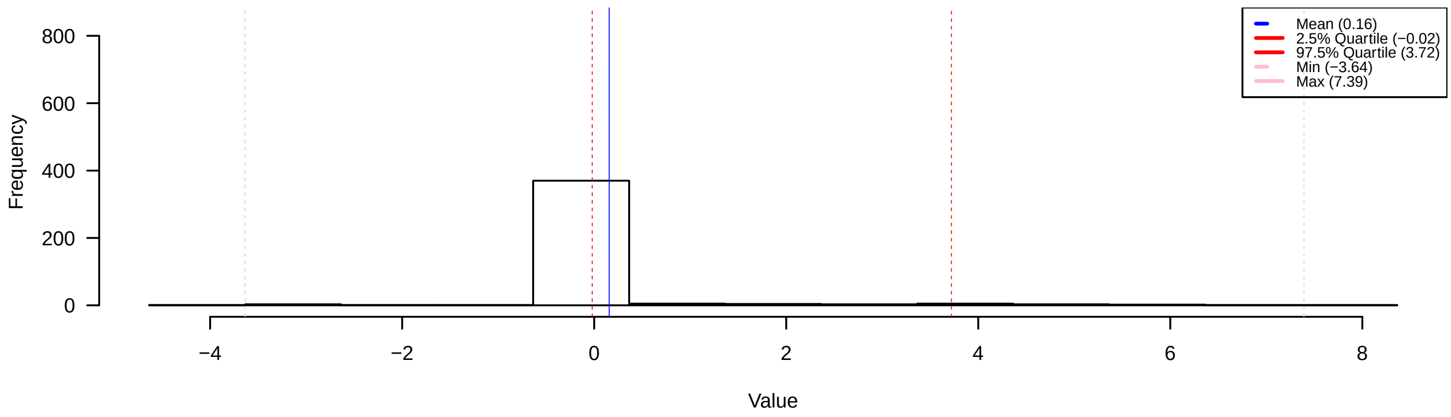

S5, Figure 429 : Bootstrap Distribution of Absolute Humidity:Relative Humidity Squared lag 8

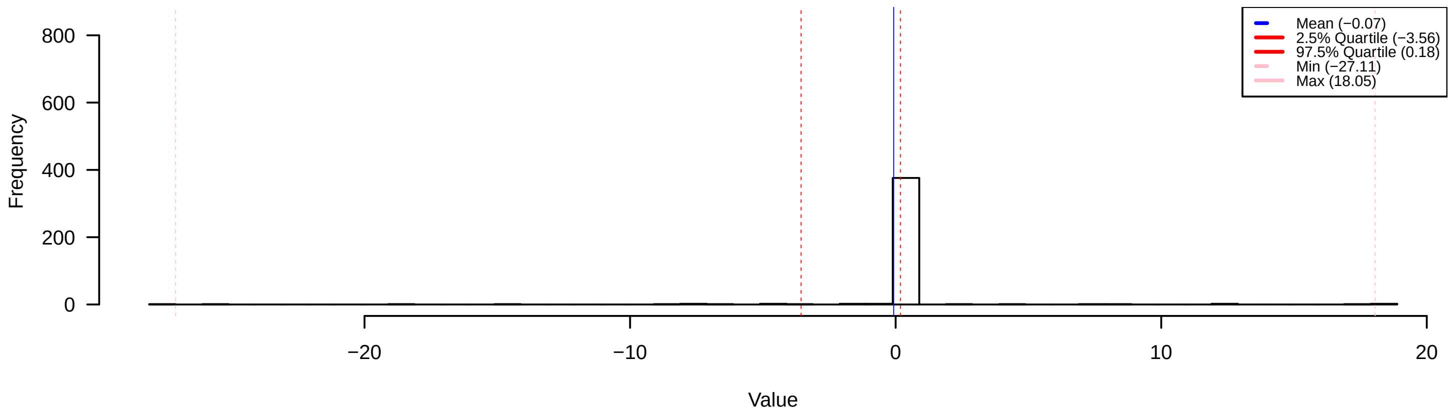

S5, Figure 430 : Bootstrap Distribution of Absolute Humidity:Relative Humidity Squared lag 9

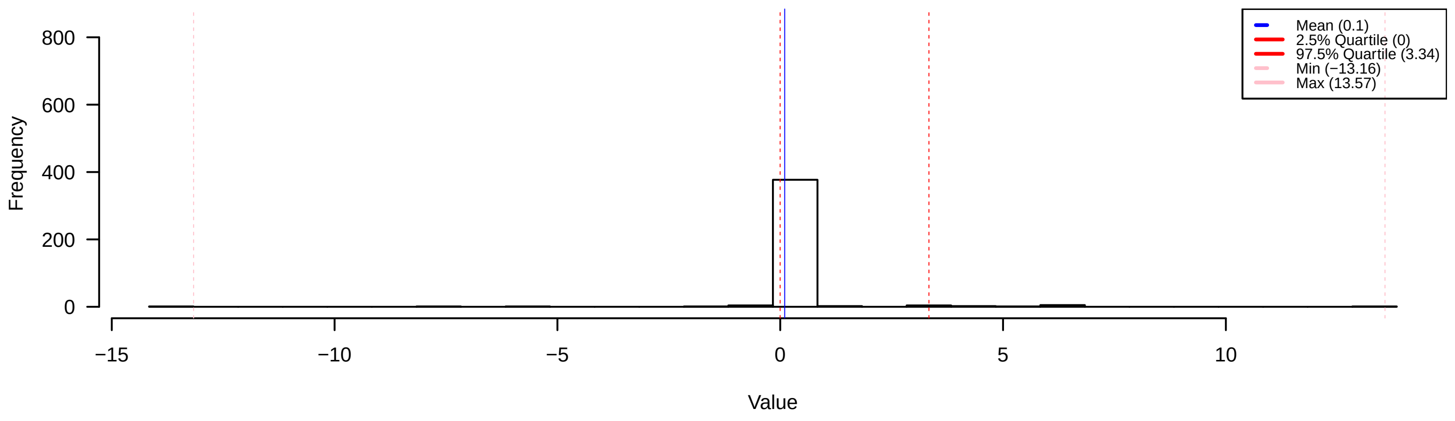

S5, Figure 431 : Bootstrap Distribution of Absolute Humidity:Relative Humidity Squared lag 10

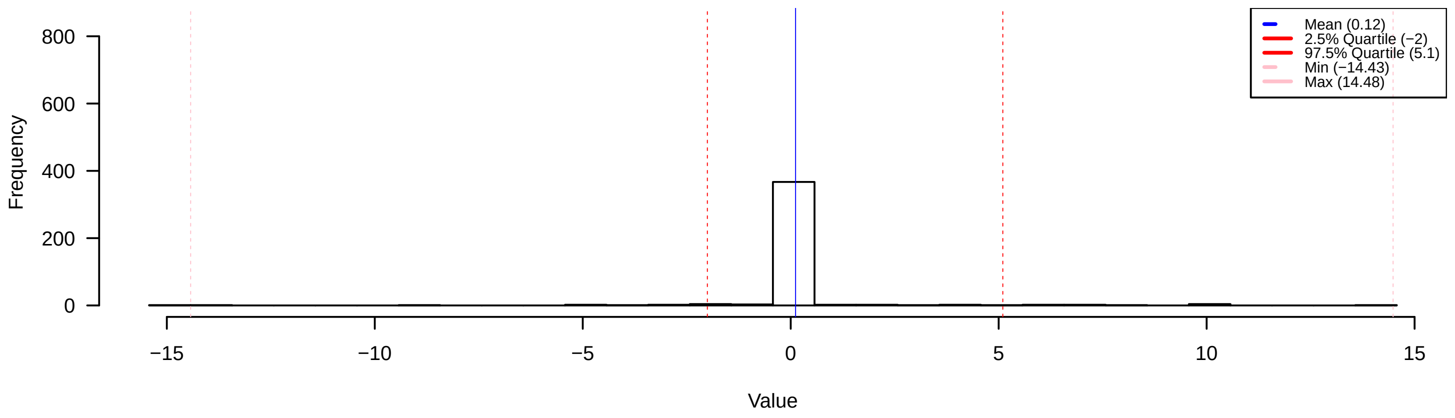

S5, Figure 432 : Bootstrap Distribution of Absolute Humidity:Relative Humidity Squared lag 11

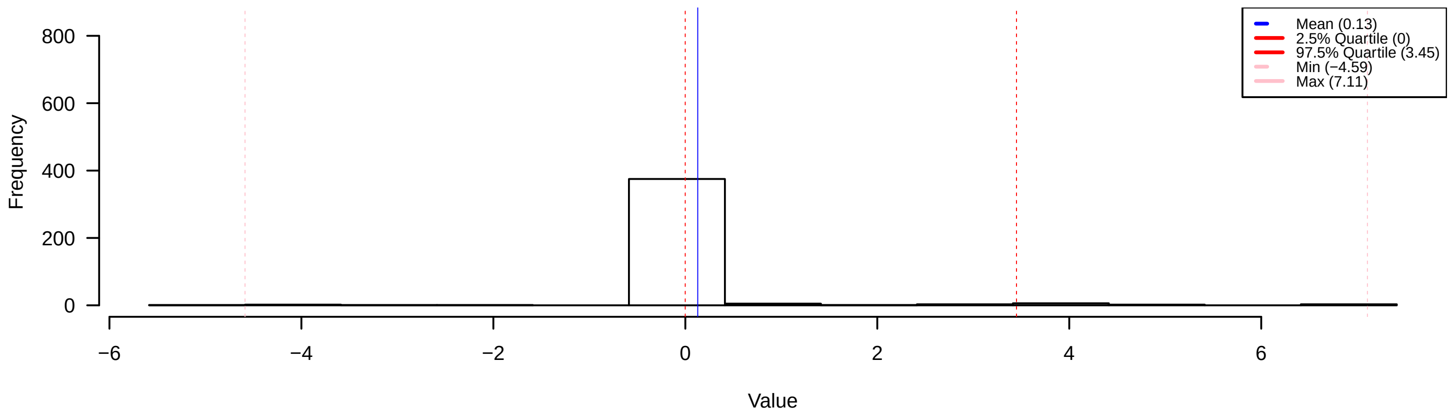

S5, Figure 433 : Bootstrap Distribution of Absolute Humidity:Relative Humidity Squared lag 12

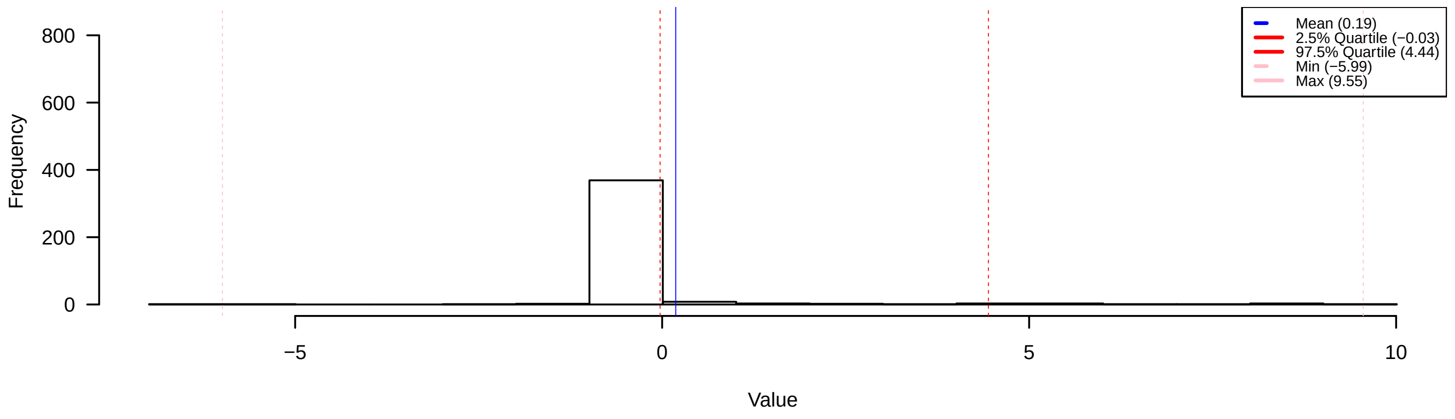

S5, Figure 434 : Bootstrap Distribution of Absolute Humidity:Relative Humidity Squared lag 13

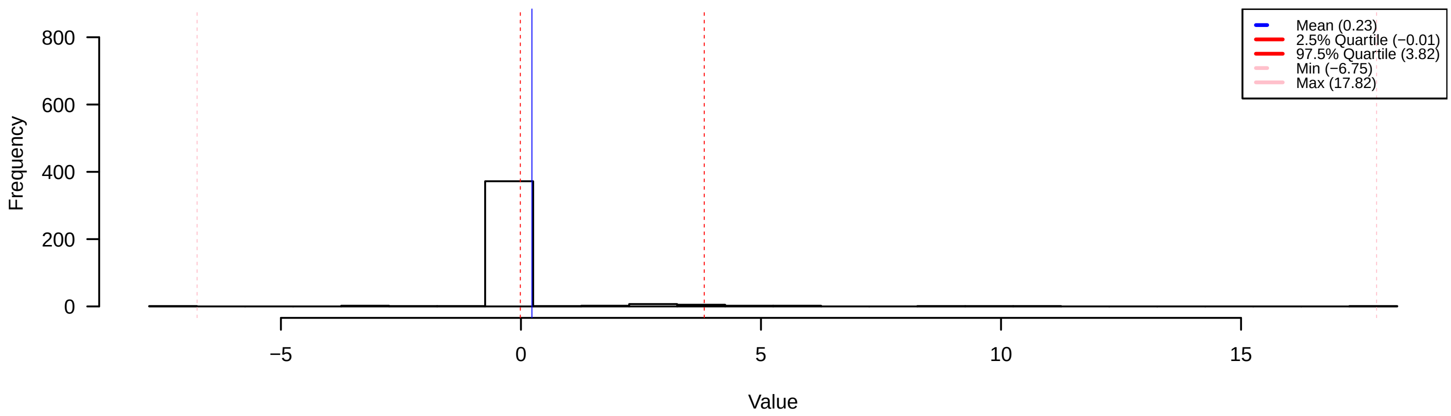

S5, Figure 435 : Bootstrap Distribution of Absolute Humidity:Relative Humidity Squared lag 14

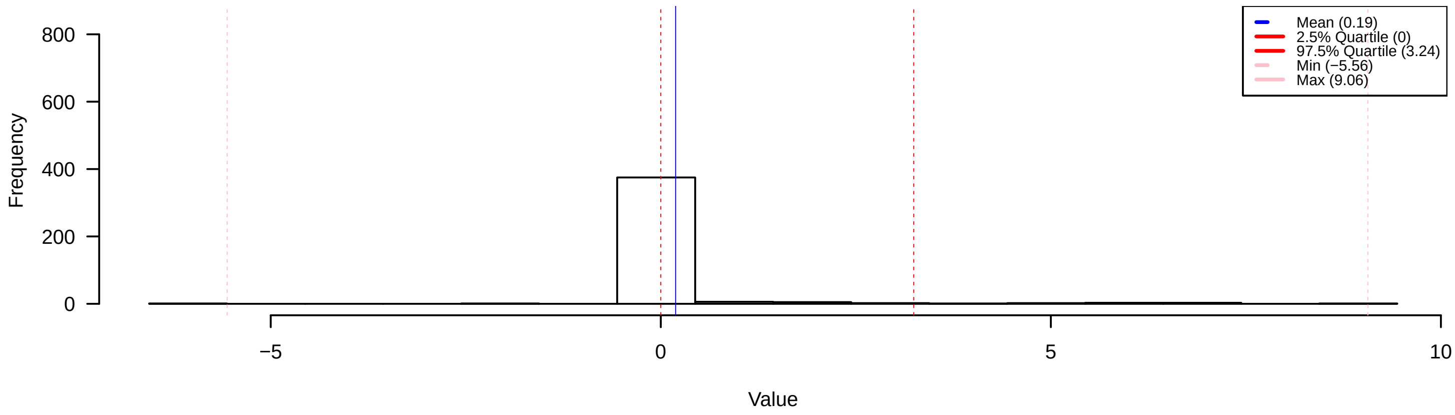

S5, Figure 436 : Bootstrap Distribution of Absolute Humidity:Relative Humidity Squared lag 15

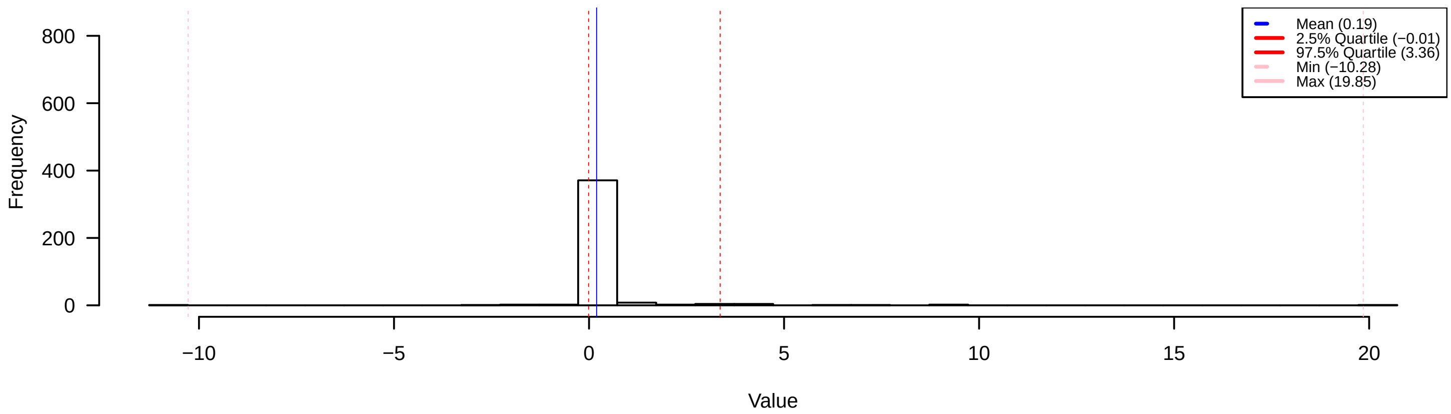

S5, Figure 437 : Bootstrap Distribution of Absolute Humidity:Relative Humidity Squared lag 16

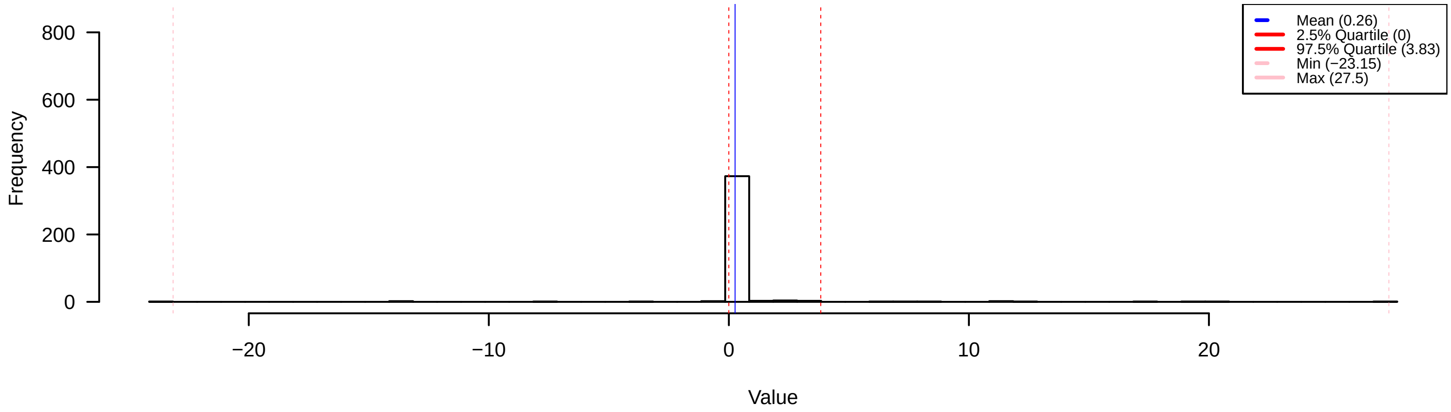

S5, Figure 438 : Bootstrap Distribution of Absolute Humidity:Relative Humidity Squared lag 17

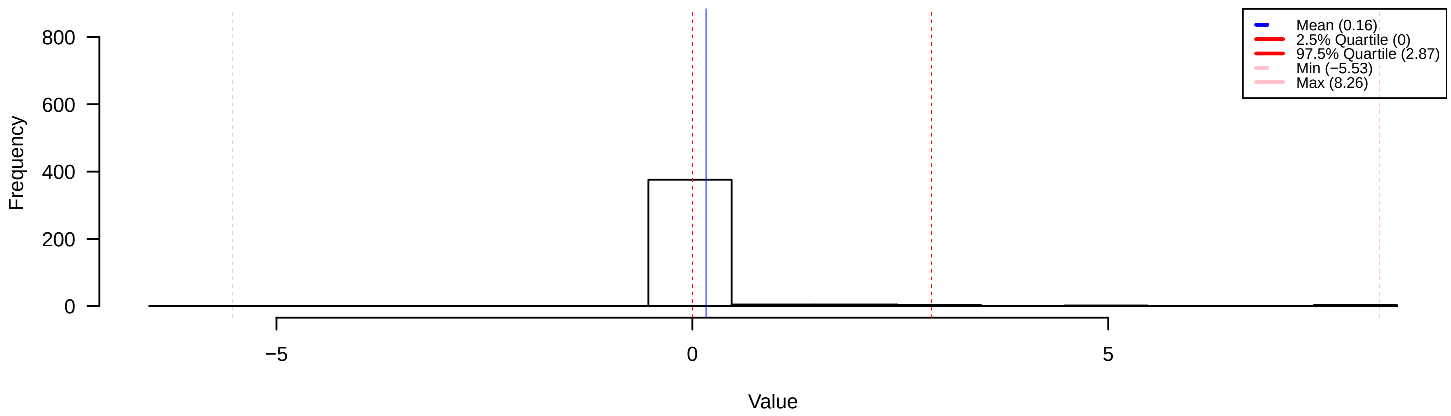

S5, Figure 439 : Bootstrap Distribution of Absolute Humidity:Relative Humidity Squared lag 18

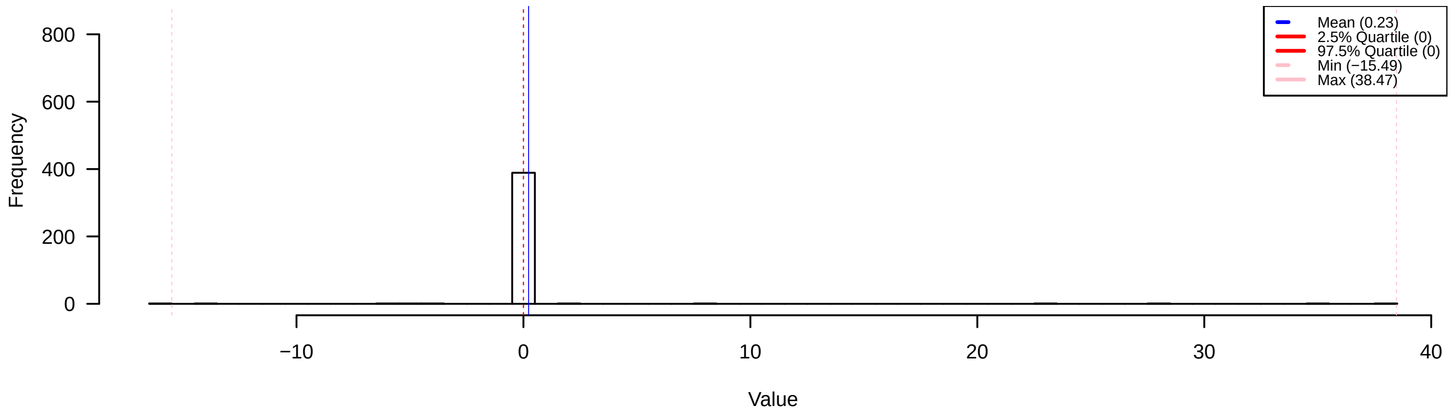

S5, Figure 440 : Bootstrap Distribution of Absolute Humidity:Relative Humidity Squared lag 19

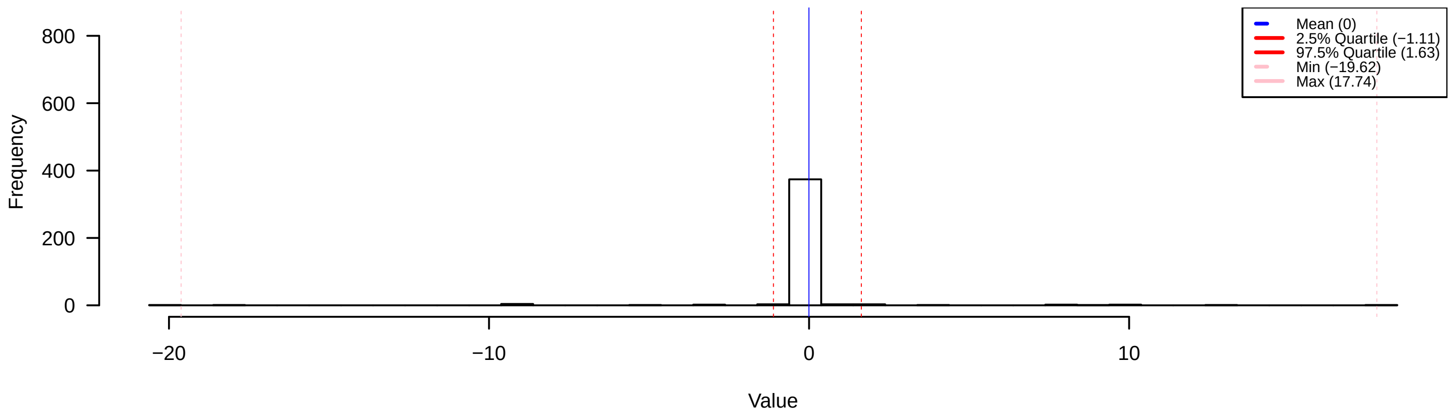

S5, Figure 441 : Bootstrap Distribution of Absolute Humidity:Relative Humidity Squared lag 20

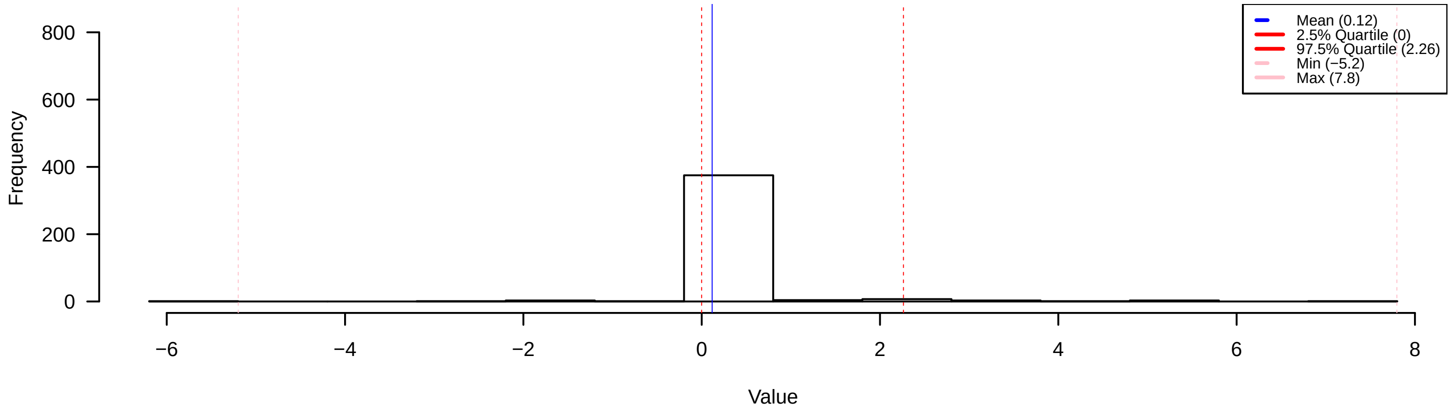

S6, Appendix Figure 442 : Curvature of Absolute Humidity lag 1

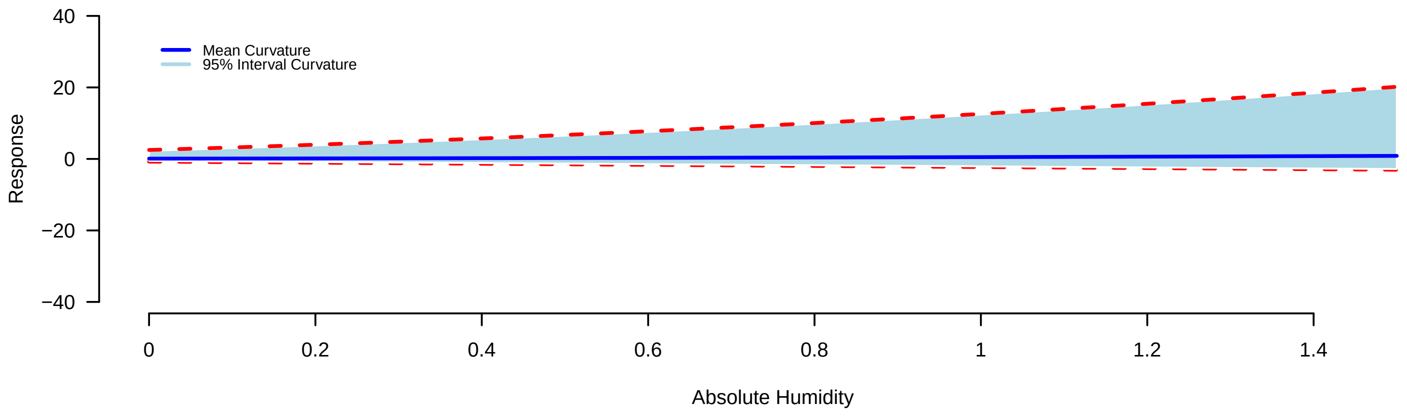

S6, Appendix Figure 443 : Curvature of Absolute Humidity lag 10

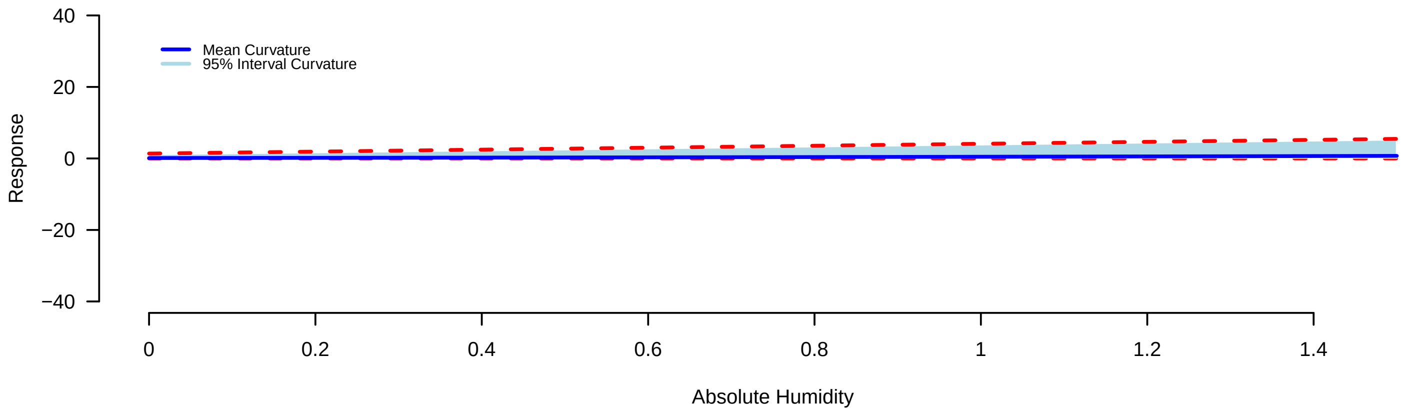

S6, Appendix Figure 444 : Curvature of Absolute Humidity lag 11

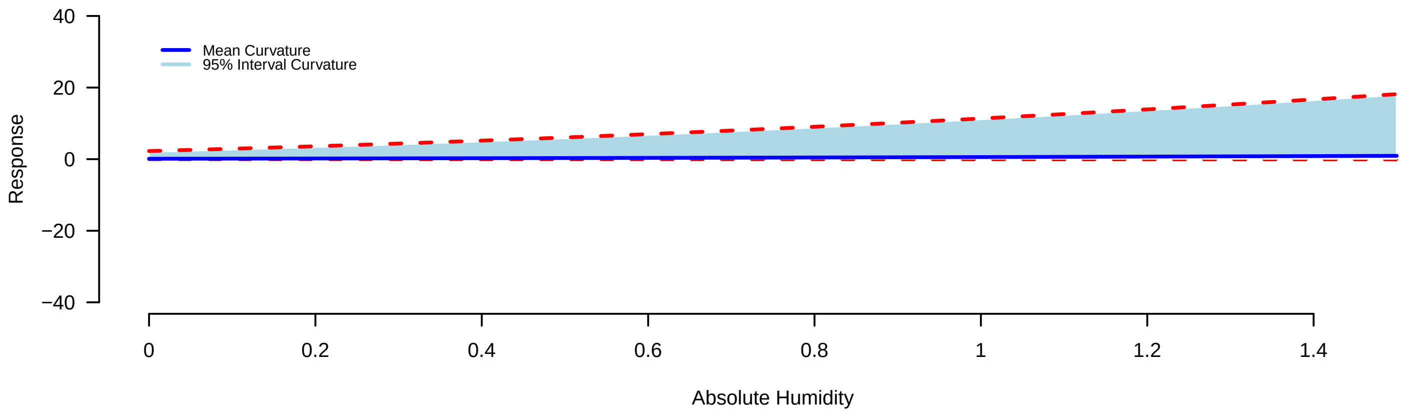

S6, Appendix Figure 445 : Curvature of Absolute Humidity lag 12

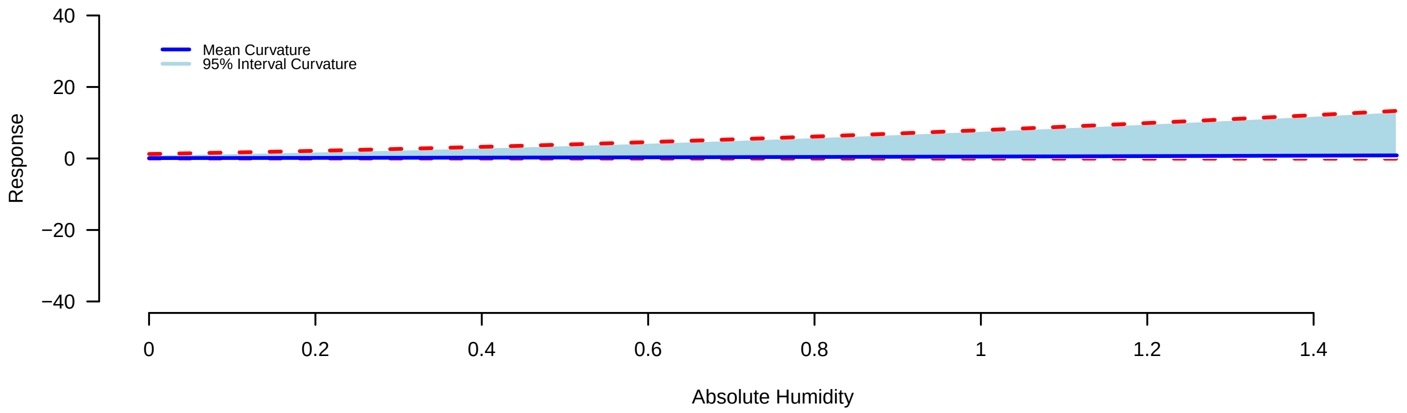

S6, Appendix Figure 446 : Curvature of Absolute Humidity lag 13

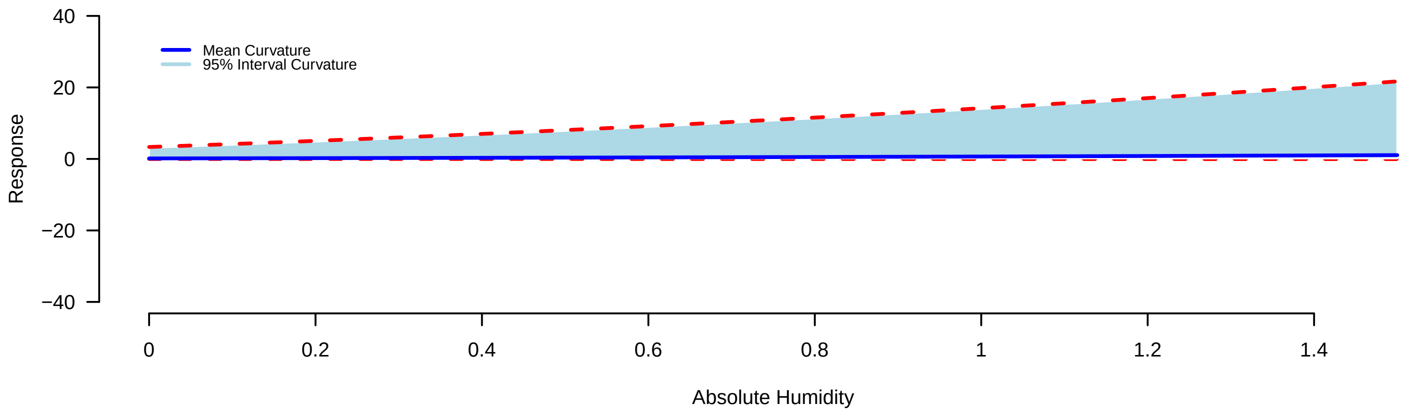

S6, Appendix Figure 447 : Curvature of Absolute Humidity lag 14

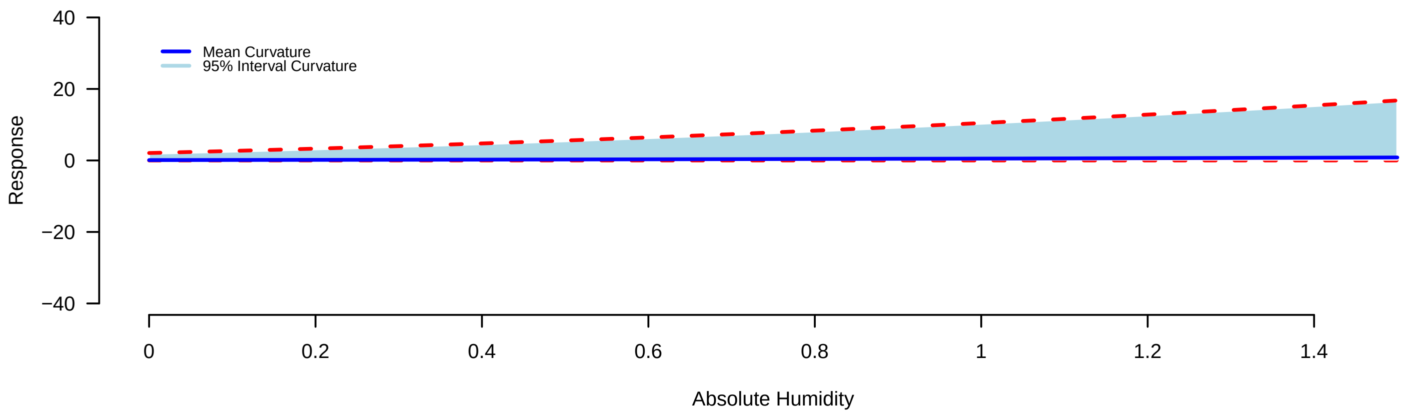

S6, Appendix Figure 448 : Curvature of Absolute Humidity lag 15

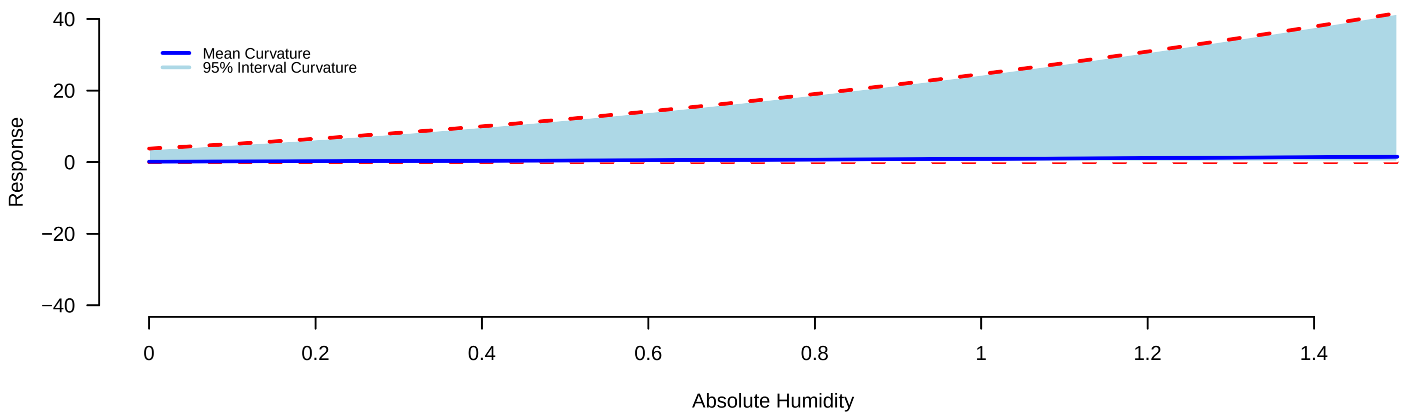

S6, Appendix Figure 449 : Curvature of Absolute Humidity lag 16

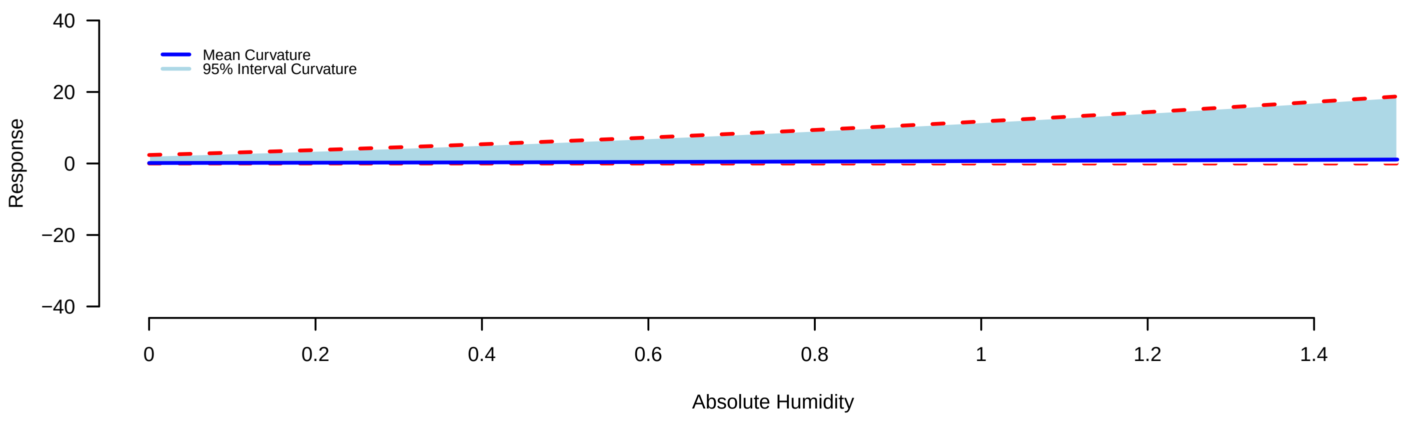

S6, Appendix Figure 450 : Curvature of Absolute Humidity lag 17

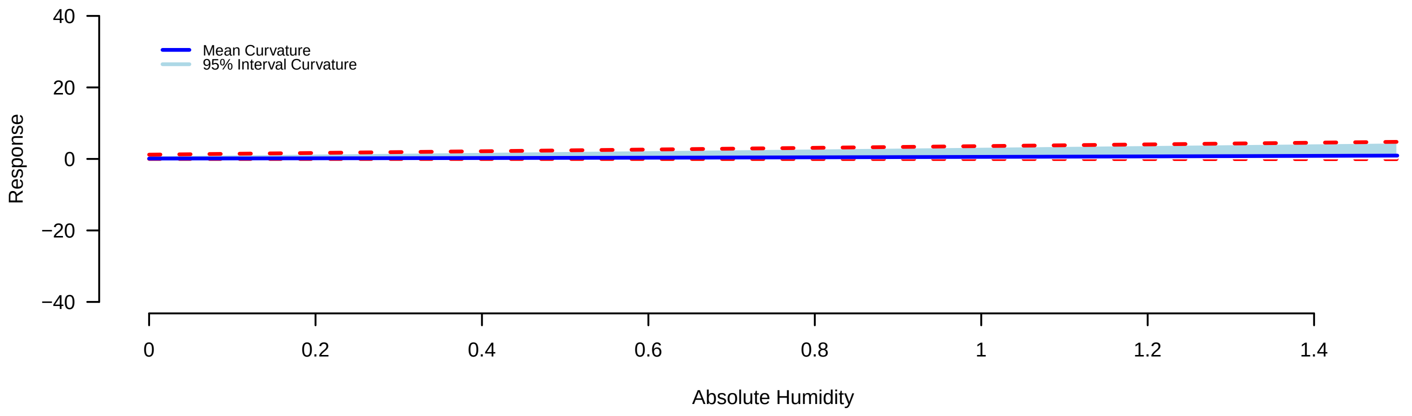

S6, Appendix Figure 451 : Curvature of Absolute Humidity lag 18

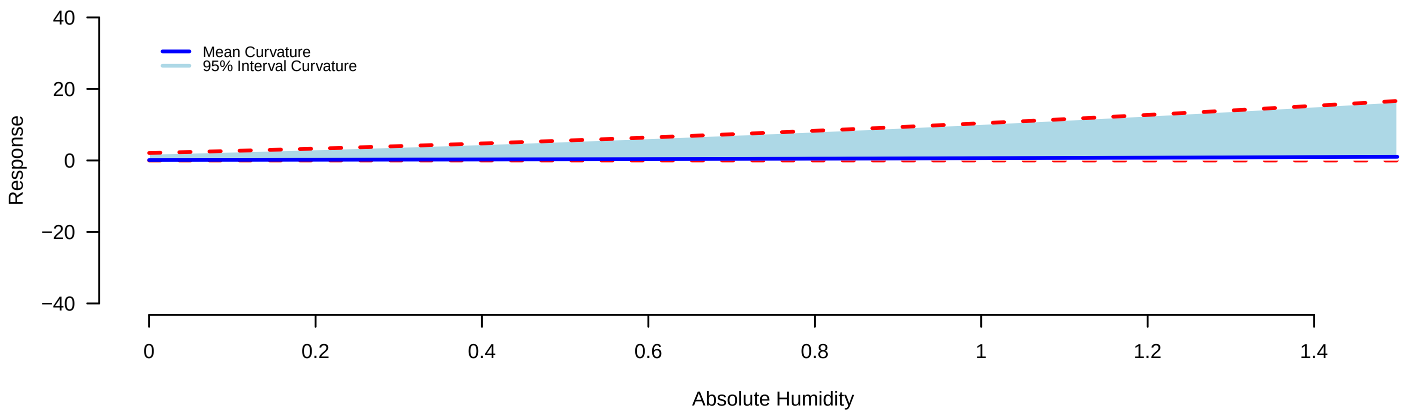

S6, Appendix Figure 452 : Curvature of Absolute Humidity lag 19

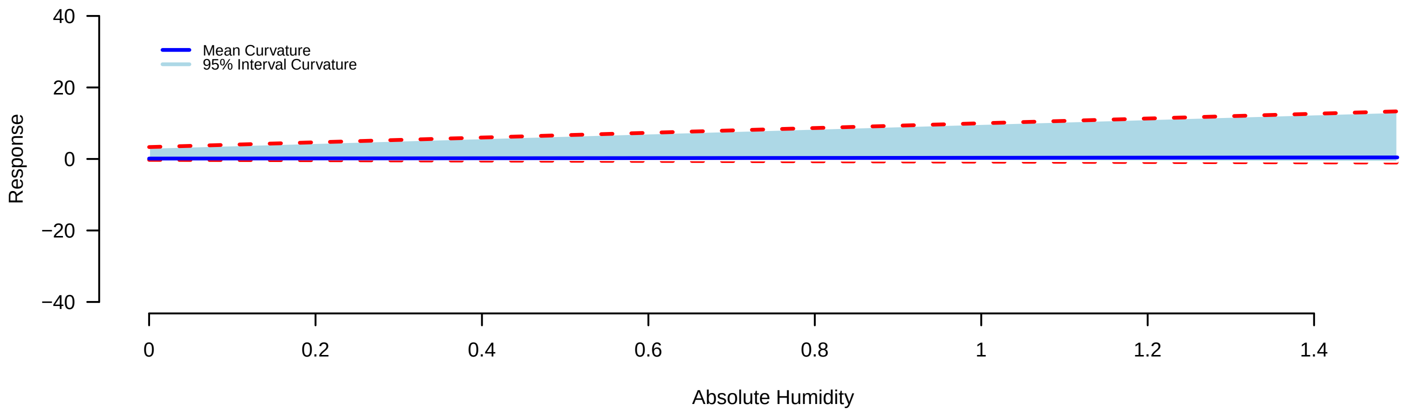

S6, Appendix Figure 453 : Curvature of Absolute Humidity lag 2

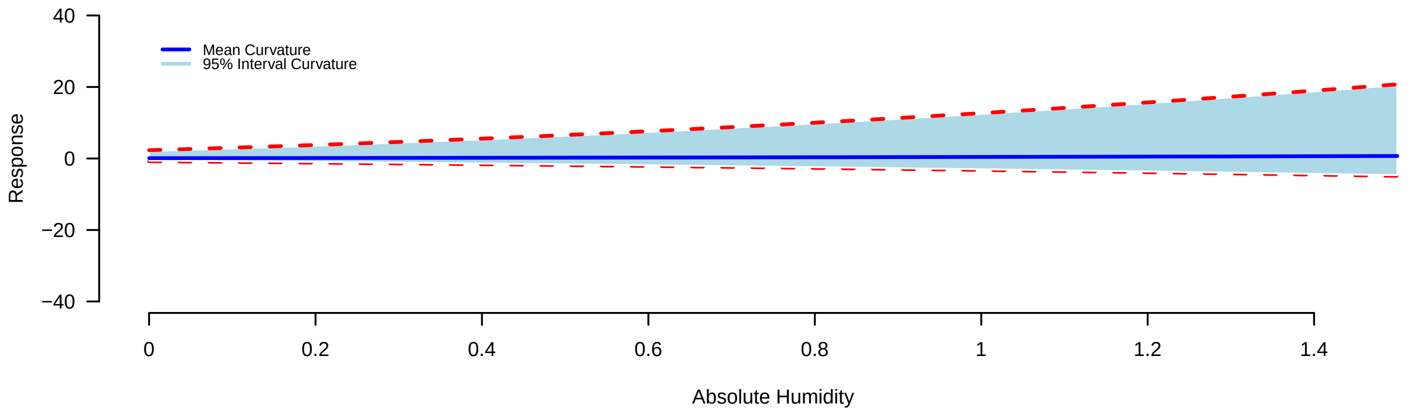

S6, Appendix Figure 454 : Curvature of Absolute Humidity lag 20

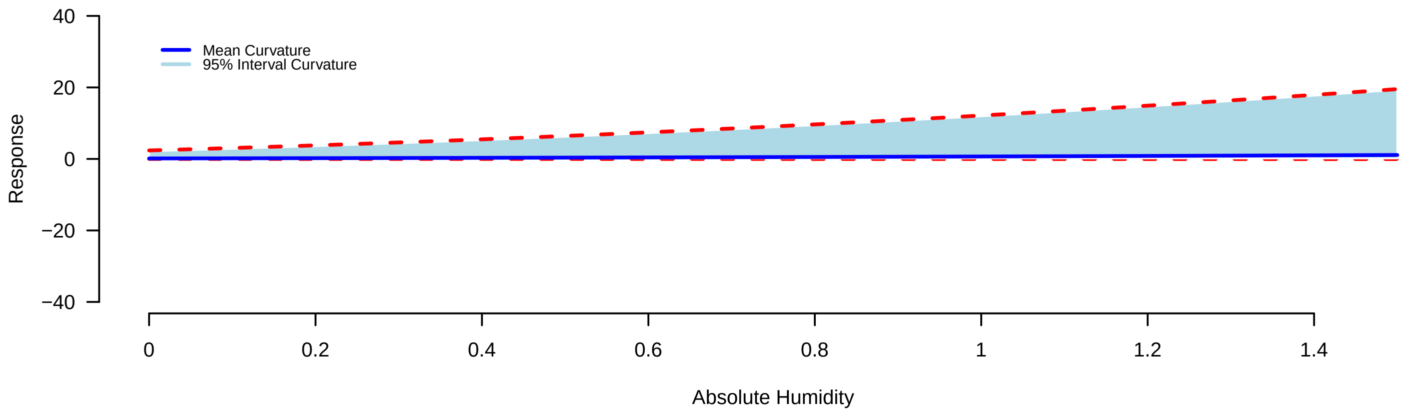

S6, Appendix Figure 455 : Curvature of Absolute Humidity lag 3

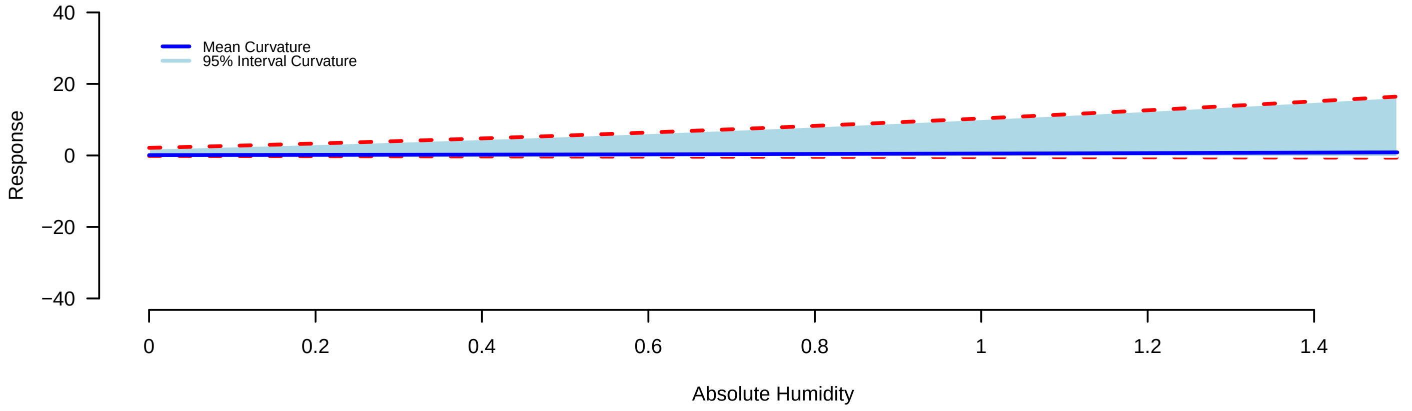

S6, Appendix Figure 456 : Curvature of Absolute Humidity lag 4

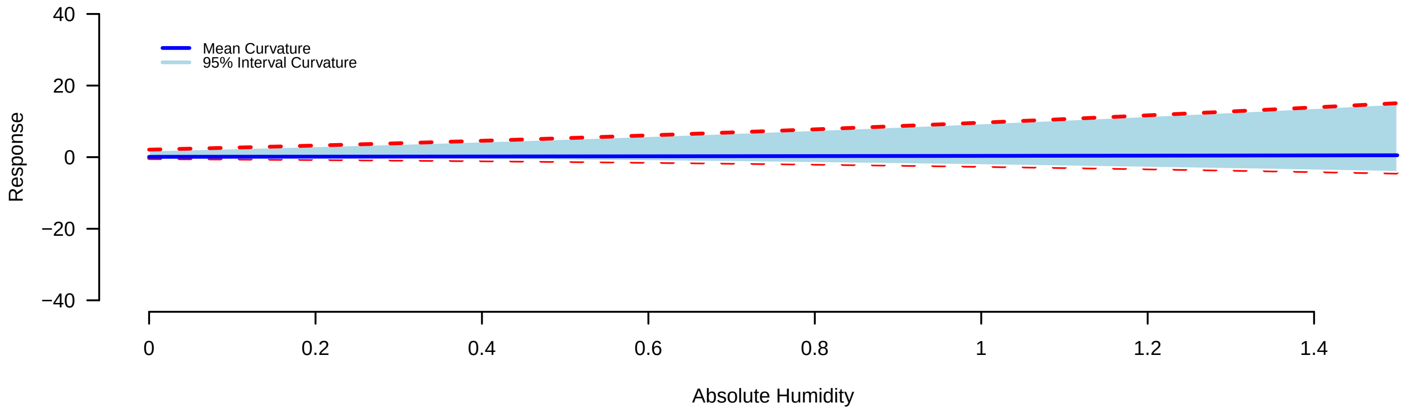

S6, Appendix Figure 457 : Curvature of Absolute Humidity lag 5

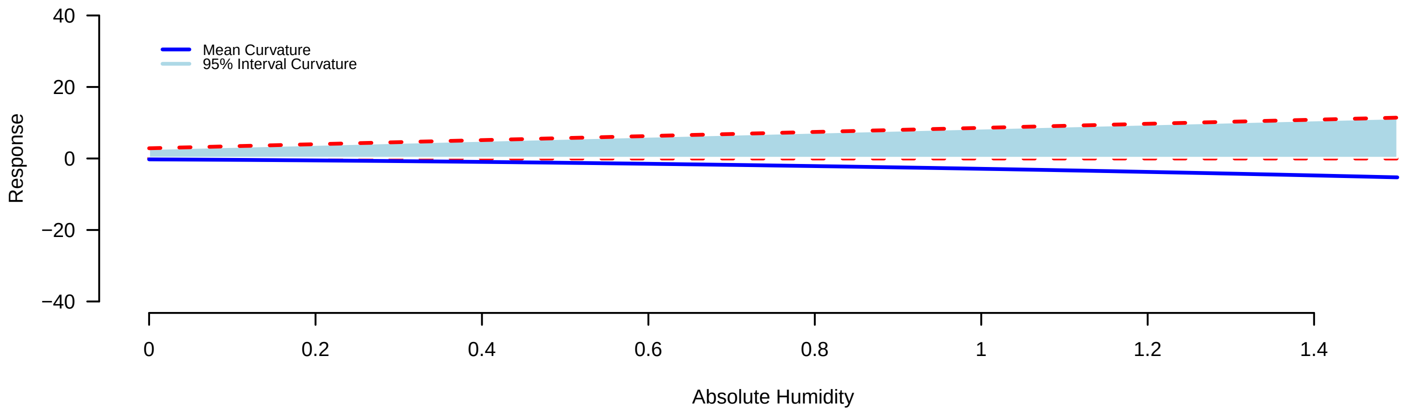

S6, Appendix Figure 458 : Curvature of Absolute Humidity lag 6

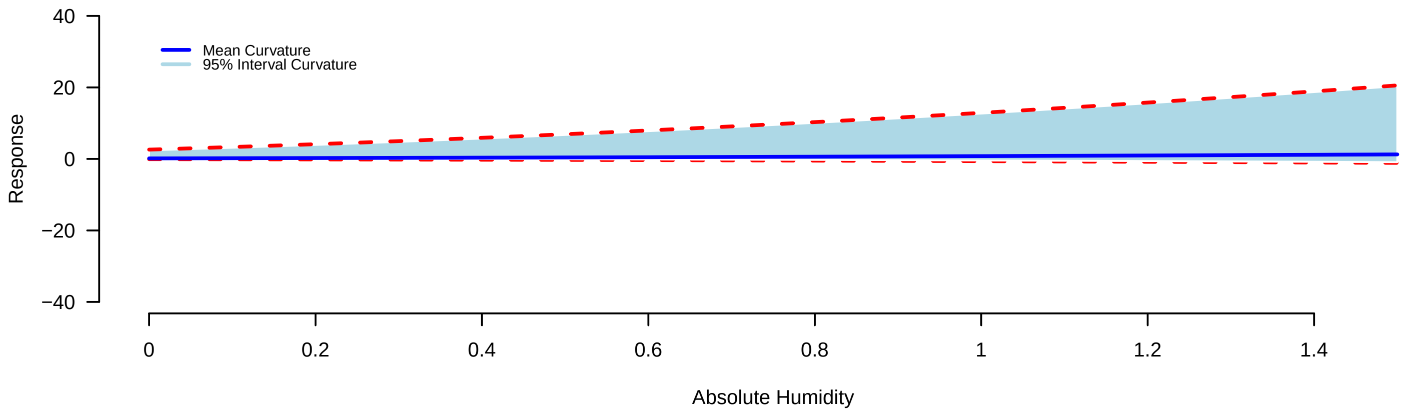

S6, Appendix Figure 459 : Curvature of Absolute Humidity lag 7

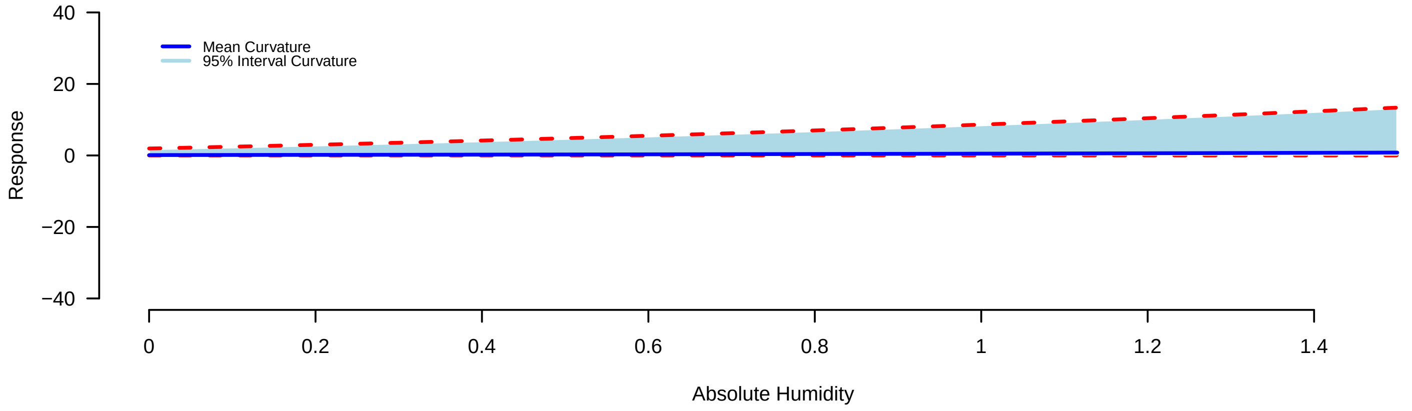

S6, Appendix Figure 460 : Curvature of Absolute Humidity lag 8

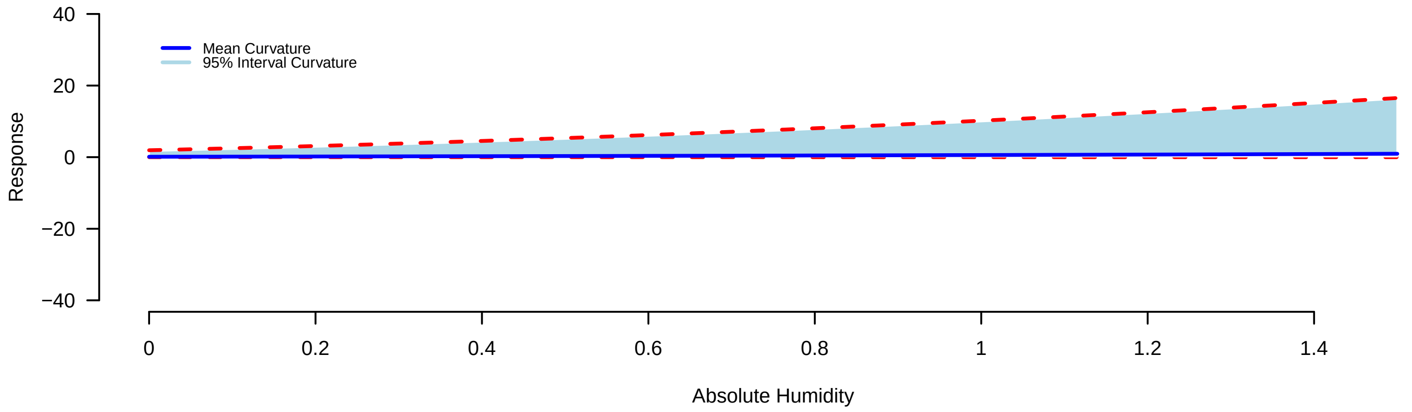

S6, Appendix Figure 461 : Curvature of Absolute Humidity lag 9

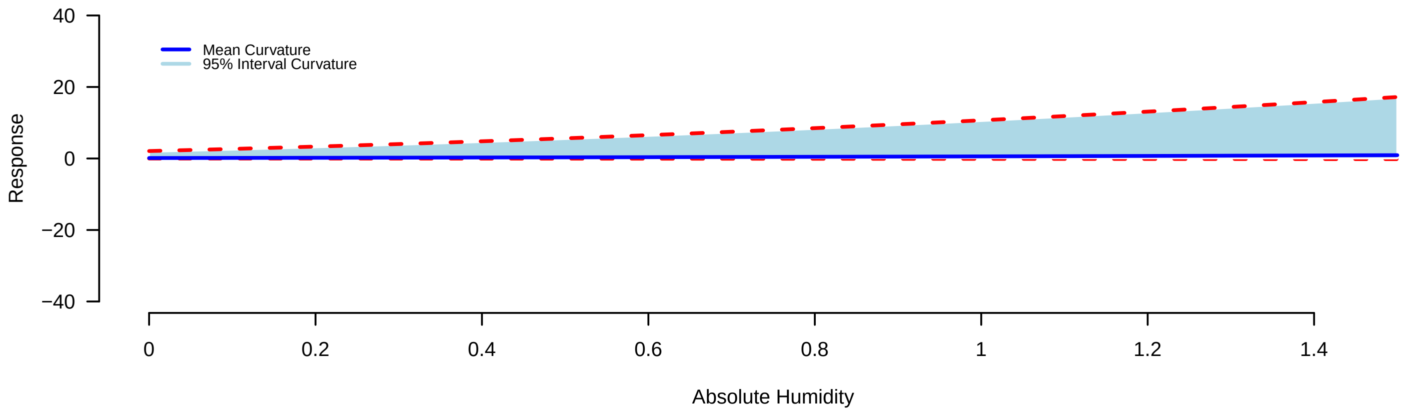

S6, Appendix Figure 462 : Curvature of Absolute Humidity:Relative Humidity lag 1

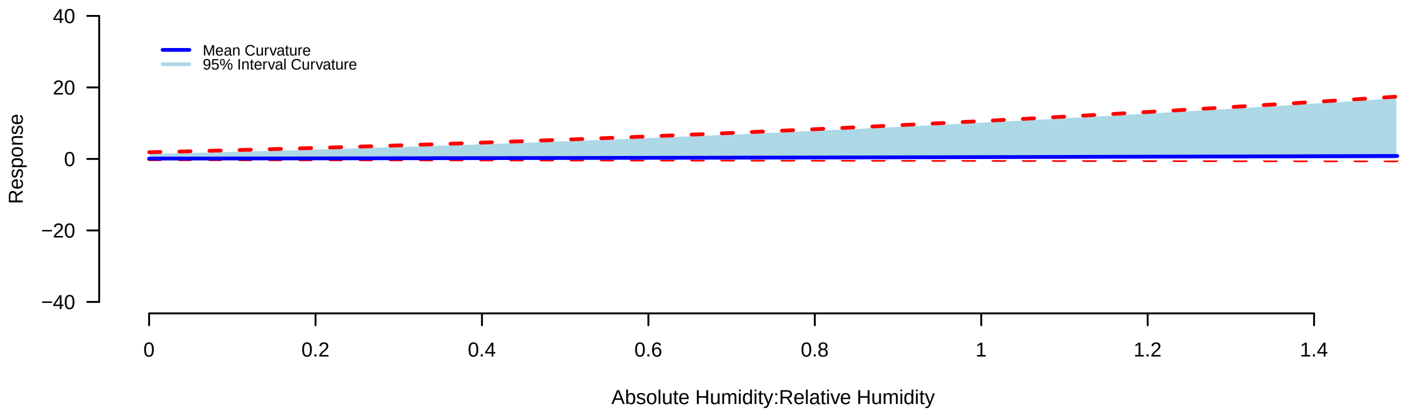

S6, Appendix Figure 463 : Curvature of Absolute Humidity:Relative Humidity lag 10

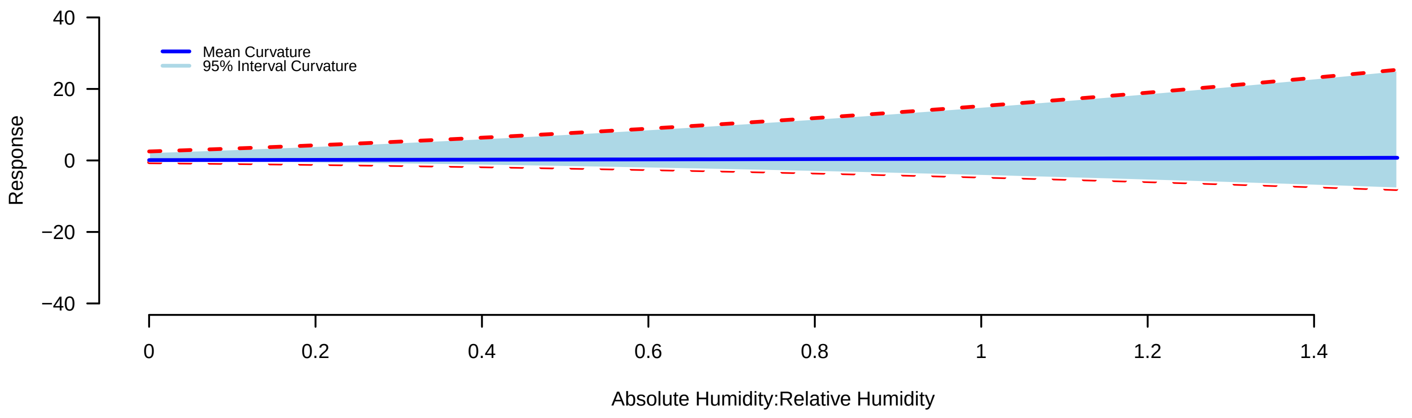

S6, Appendix Figure 464 : Curvature of Absolute Humidity:Relative Humidity lag 11

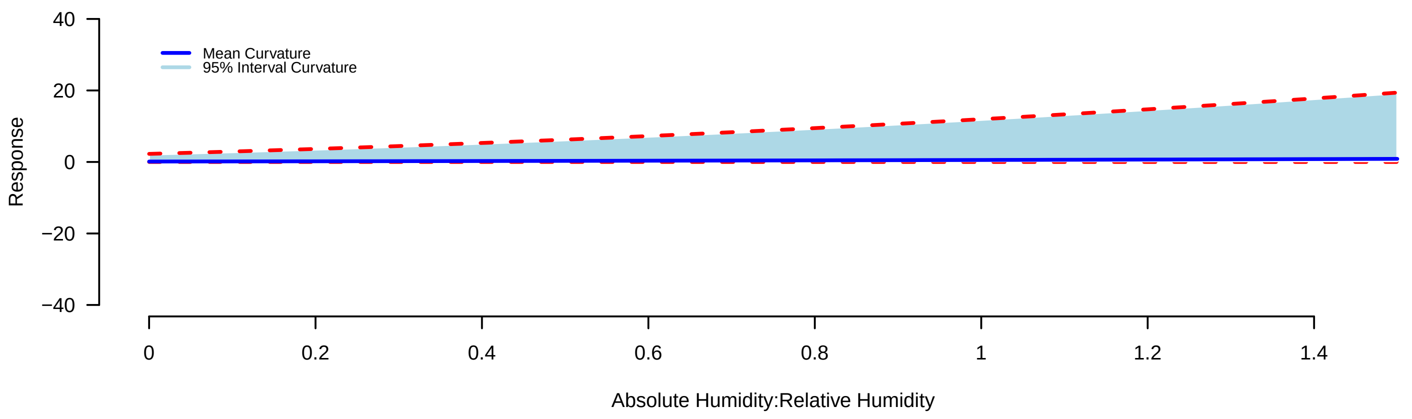

S6, Appendix Figure 465 : Curvature of Absolute Humidity:Relative Humidity lag 12

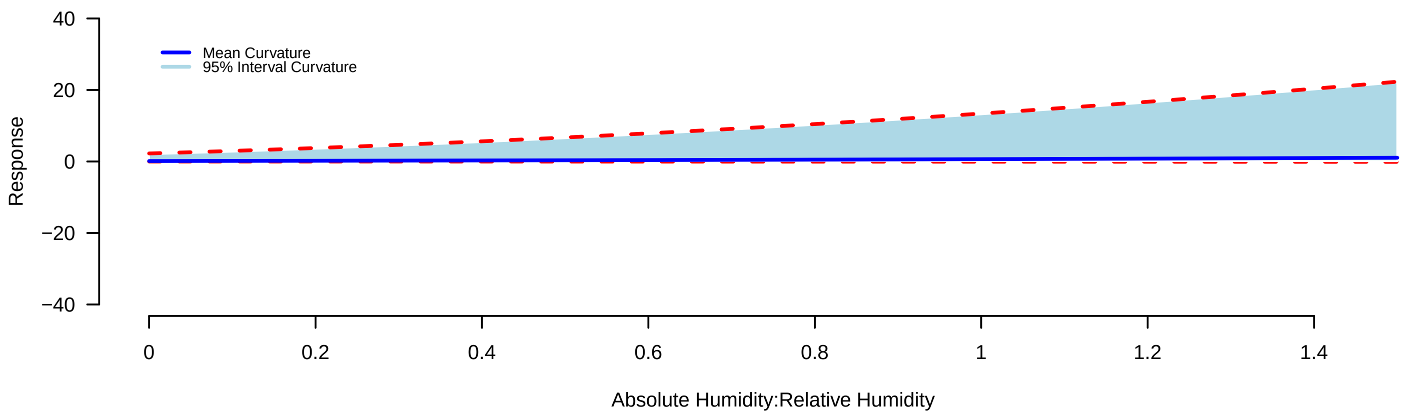

S6, Appendix Figure 466 : Curvature of Absolute Humidity:Relative Humidity lag 13

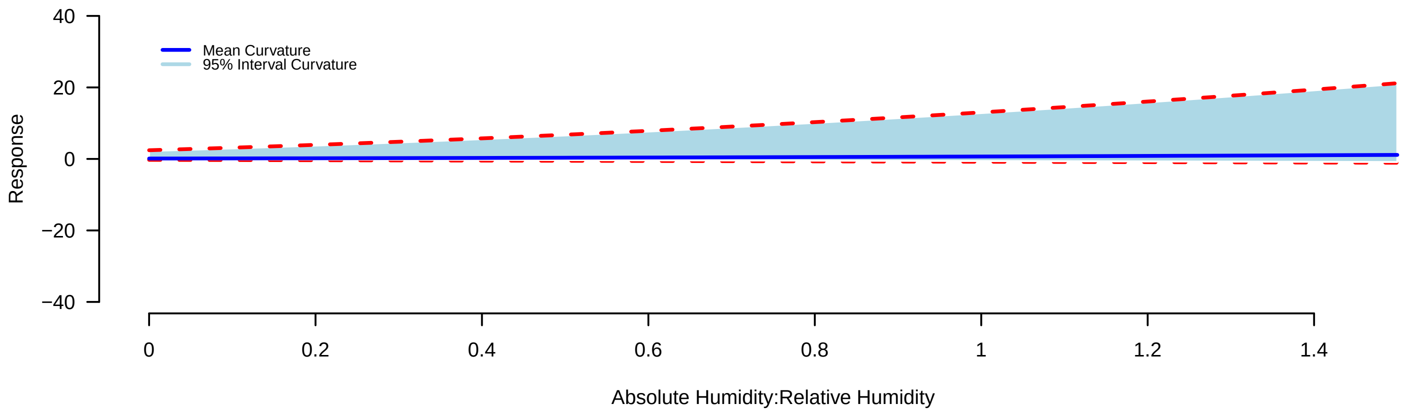

S6, Appendix Figure 467 : Curvature of Absolute Humidity:Relative Humidity lag 14

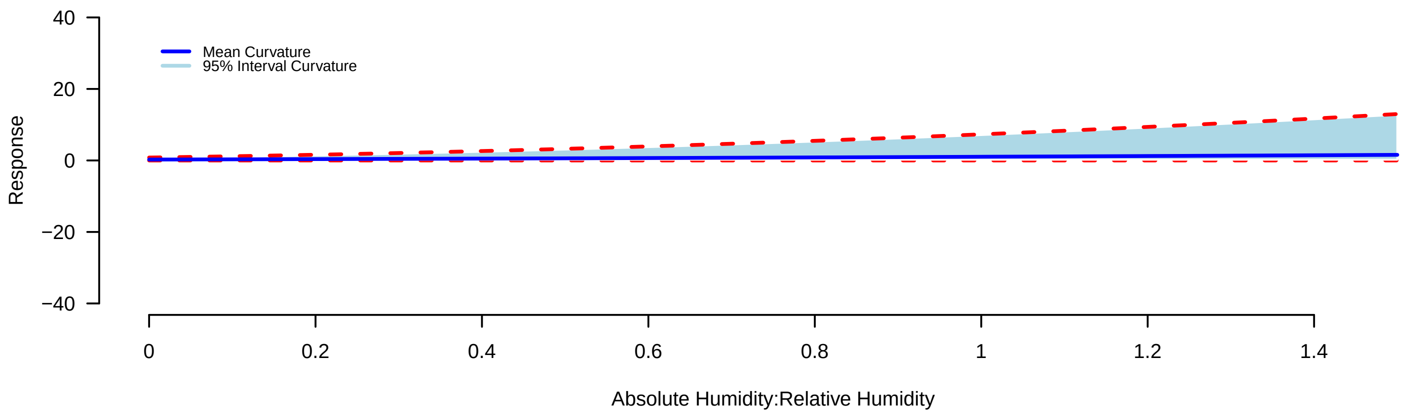

S6, Appendix Figure 468 : Curvature of Absolute Humidity:Relative Humidity lag 15

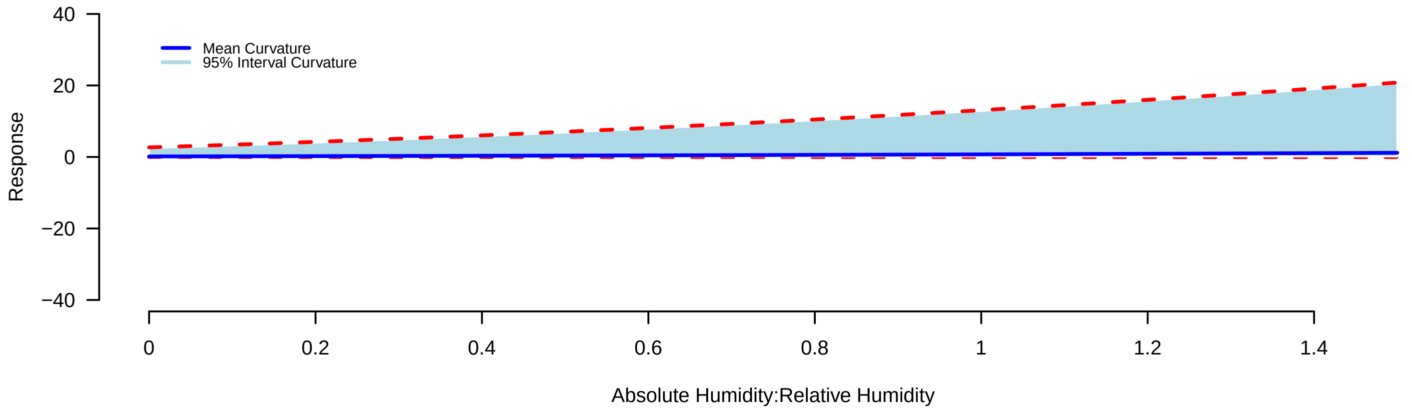

S6, Appendix Figure 469 : Curvature of Absolute Humidity:Relative Humidity lag 16

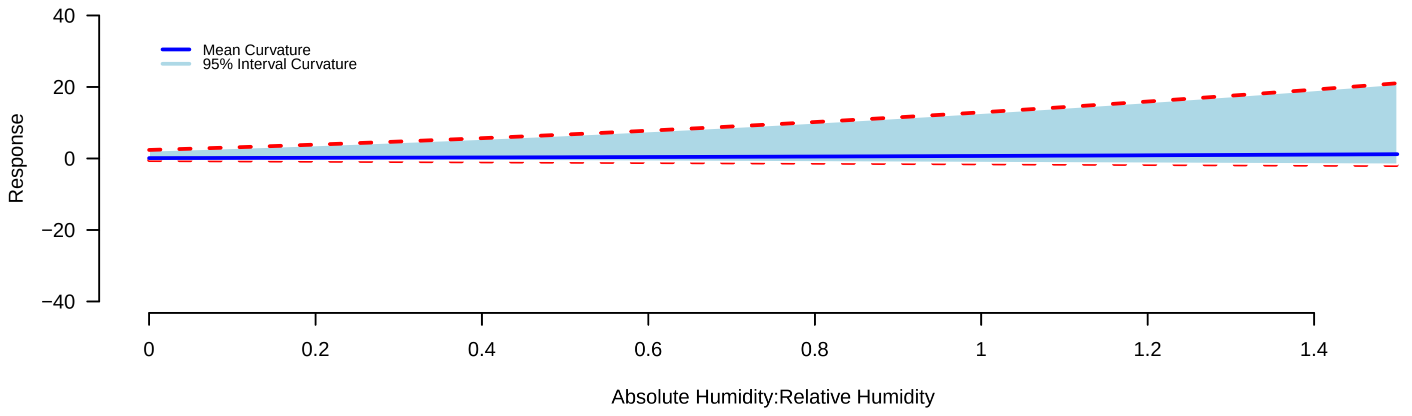

S6, Appendix Figure 470 : Curvature of Absolute Humidity:Relative Humidity lag 17

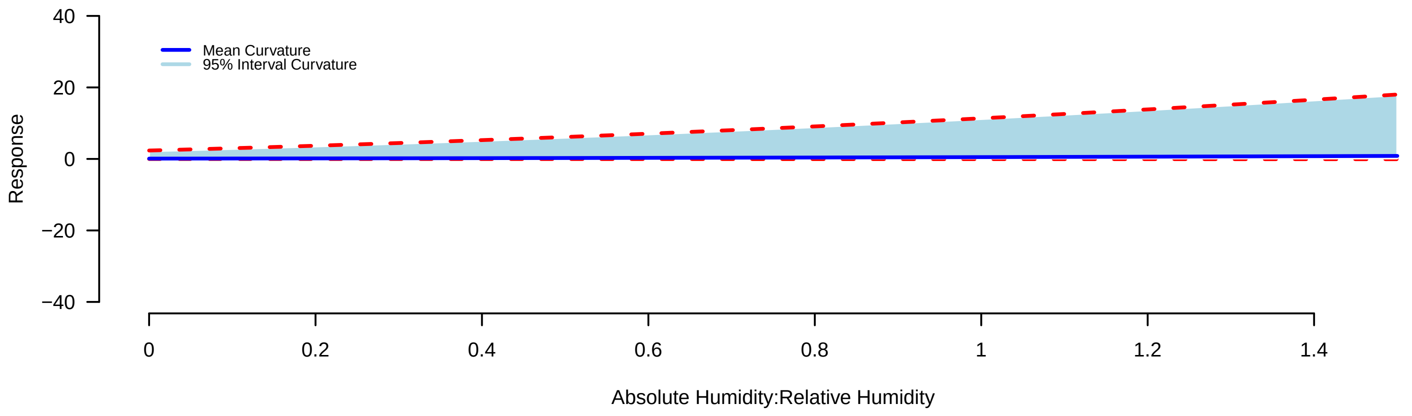

S6, Appendix Figure 471 : Curvature of Absolute Humidity:Relative Humidity lag 18

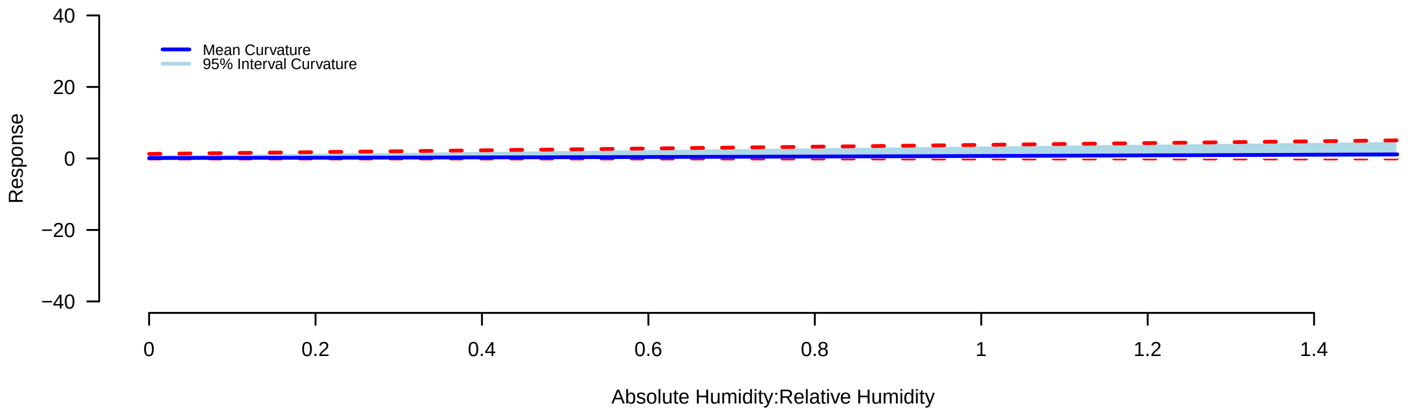

S6, Appendix Figure 472 : Curvature of Absolute Humidity:Relative Humidity lag 19

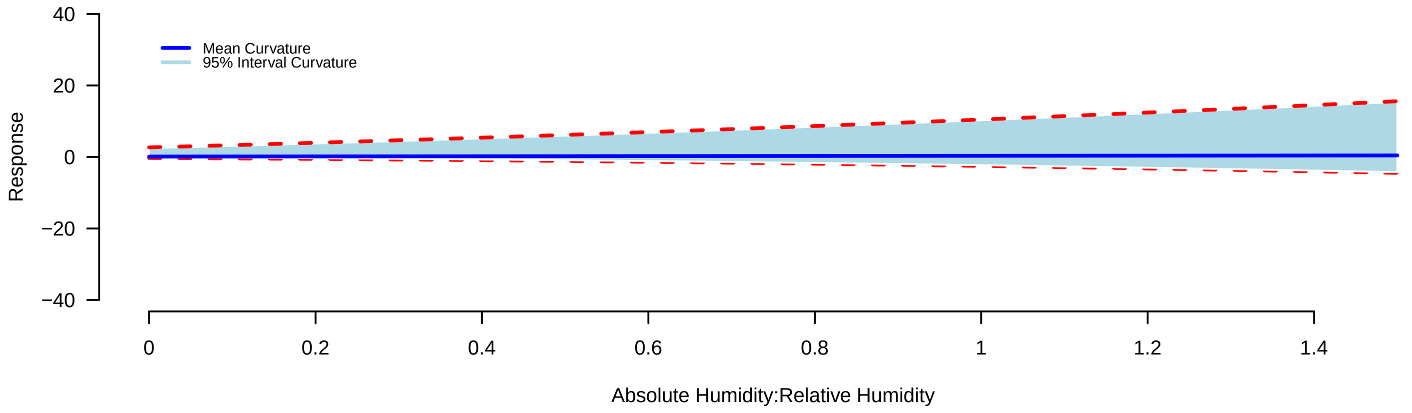

S6, Appendix Figure 473 : Curvature of Absolute Humidity:Relative Humidity lag 2

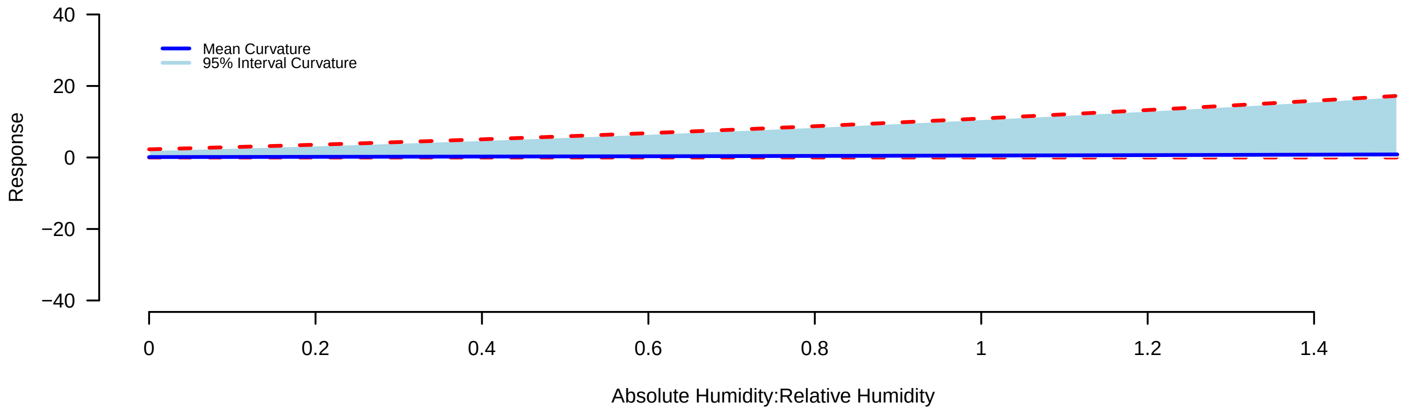

S6, Appendix Figure 474 : Curvature of Absolute Humidity:Relative Humidity lag 20

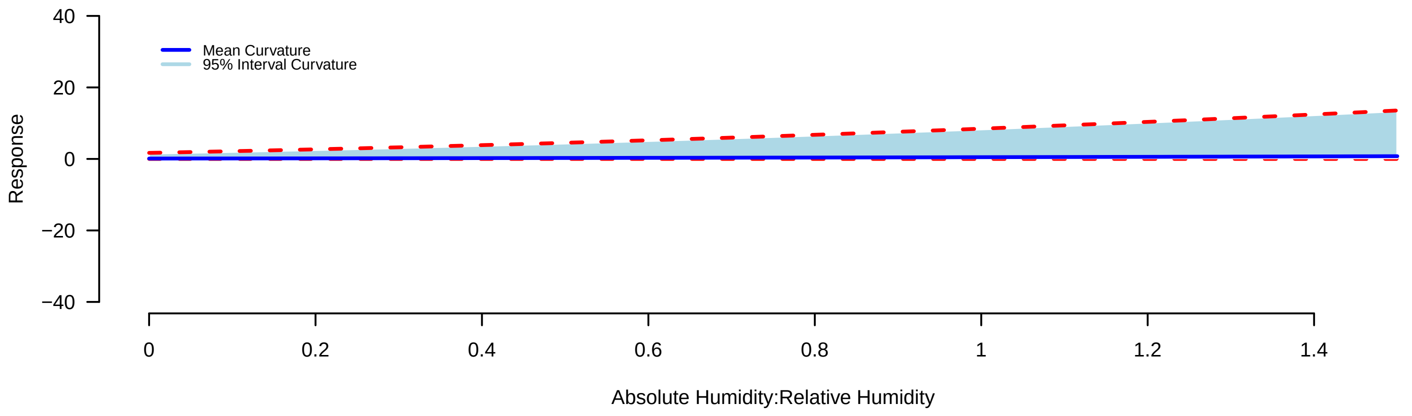

S6, Appendix Figure 475 : Curvature of Absolute Humidity:Relative Humidity lag 3

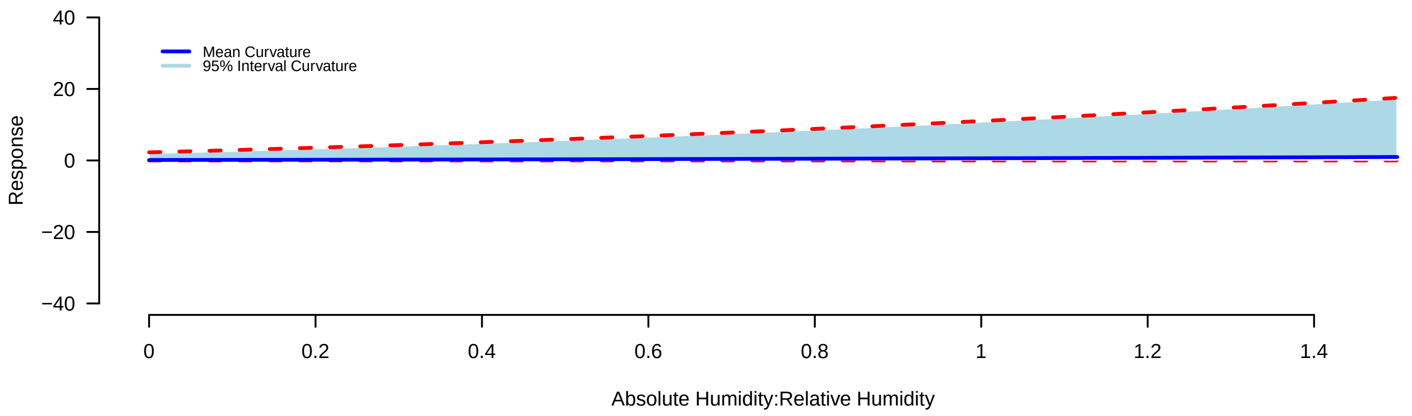

S6, Appendix Figure 476 : Curvature of Absolute Humidity:Relative Humidity lag 4

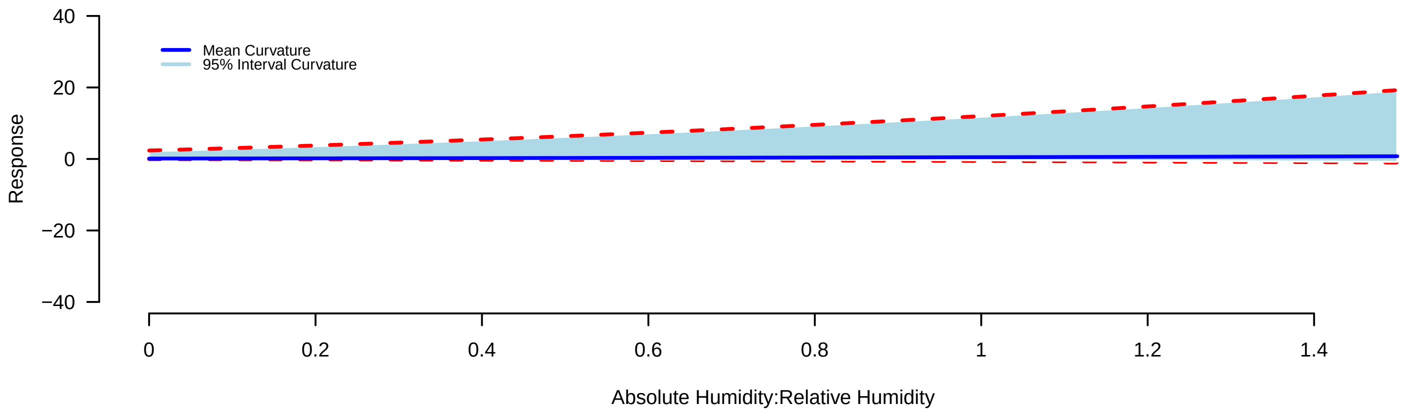

S6, Appendix Figure 477 : Curvature of Absolute Humidity:Relative Humidity lag 5

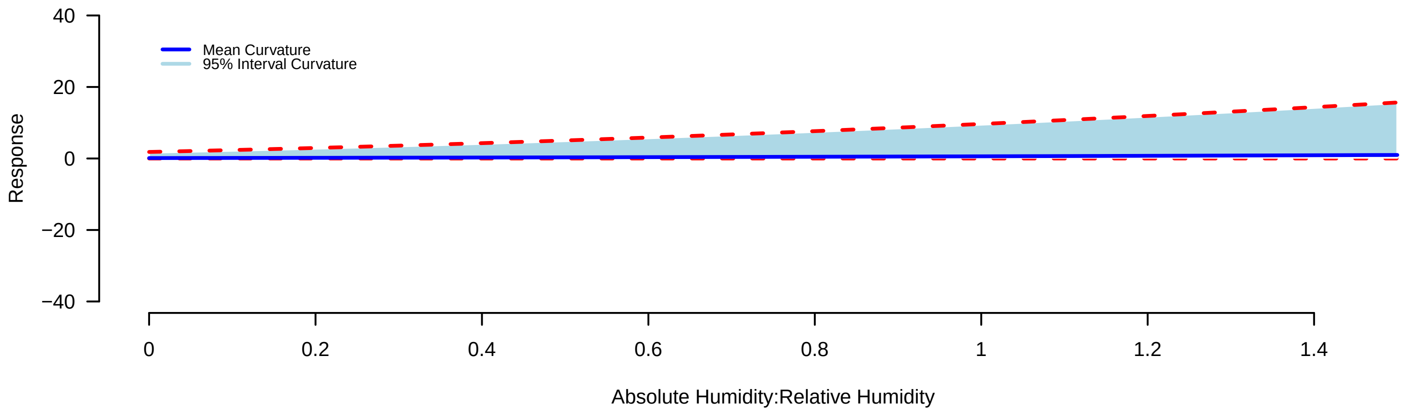

S6, Appendix Figure 478 : Curvature of Absolute Humidity:Relative Humidity lag 6

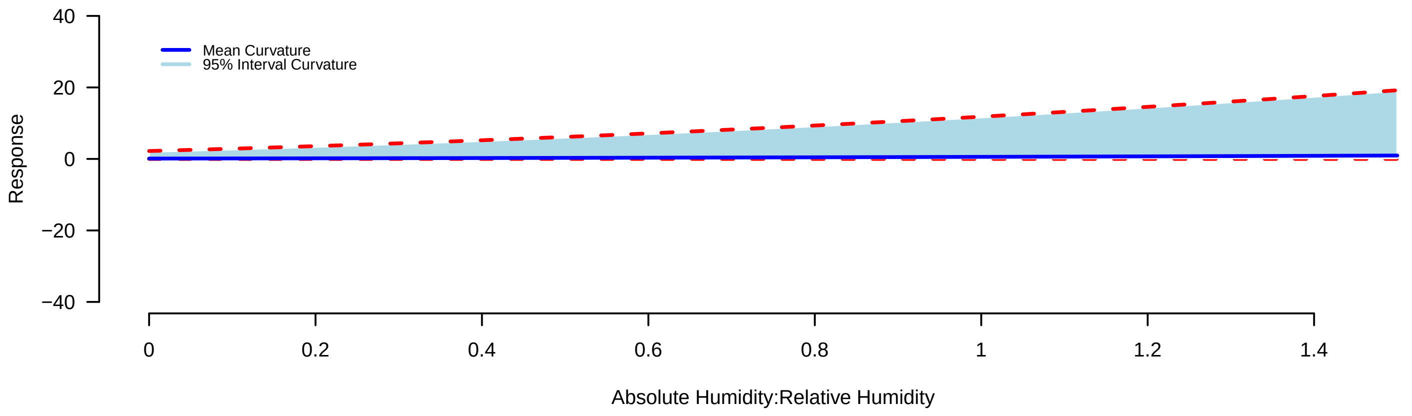

S6, Appendix Figure 479 : Curvature of Absolute Humidity:Relative Humidity lag 7

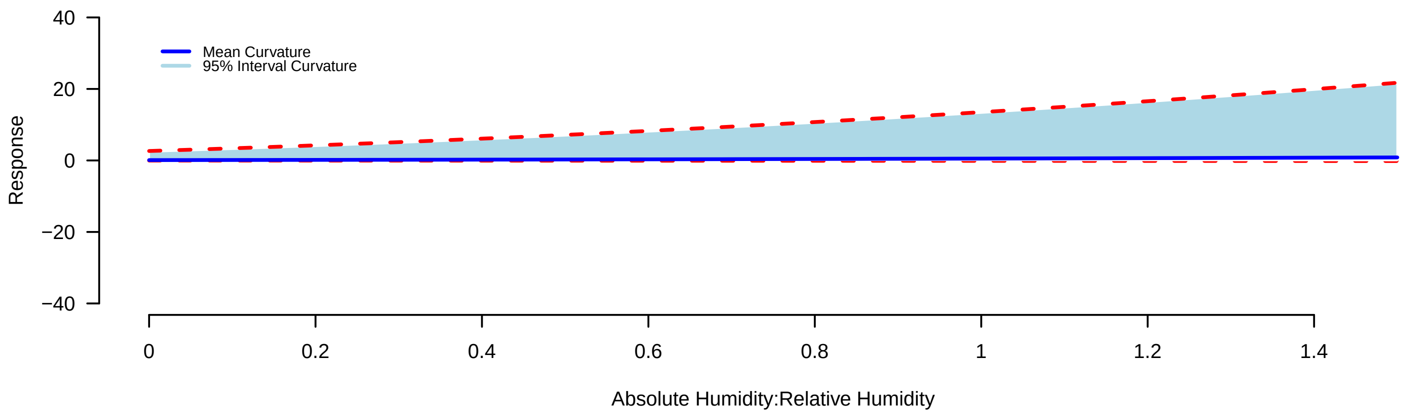

S6, Appendix Figure 480 : Curvature of Absolute Humidity:Relative Humidity lag 8

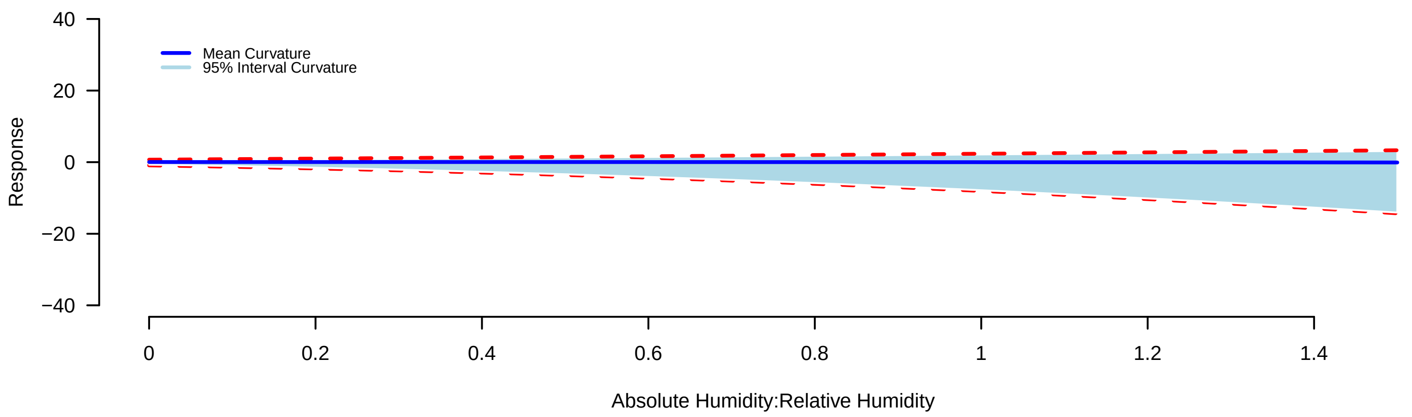

S6, Appendix Figure 481 : Curvature of Absolute Humidity:Relative Humidity lag 9

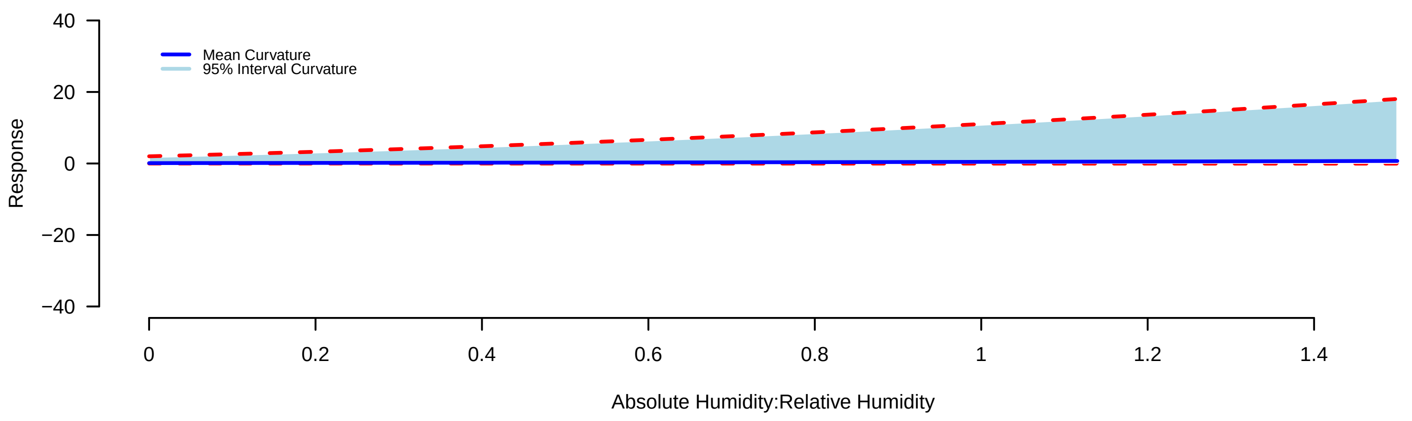

S6, Appendix Figure 482 : Curvature of Air Temperature lag 1

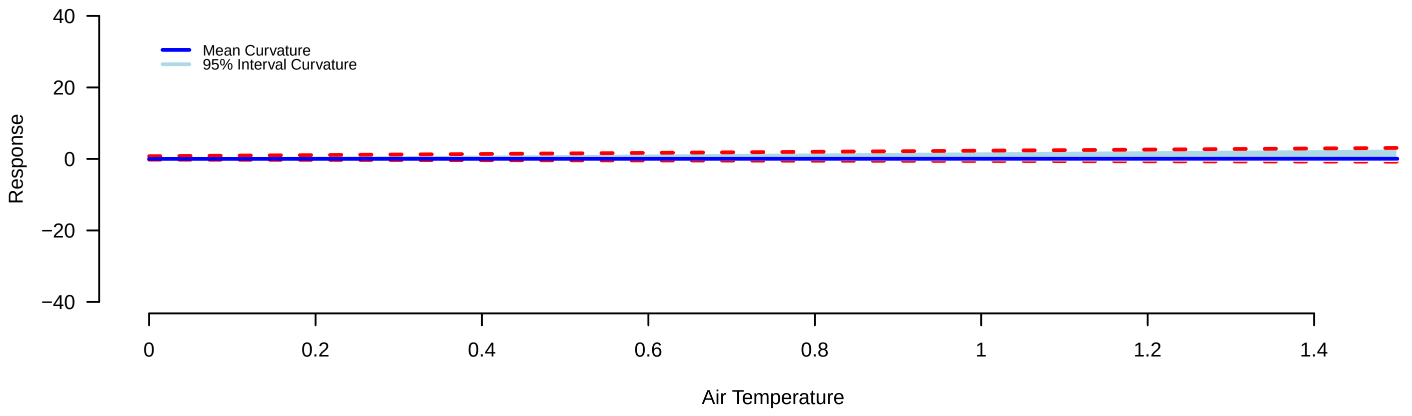

S6, Appendix Figure 483 : Curvature of Air Temperature lag 10

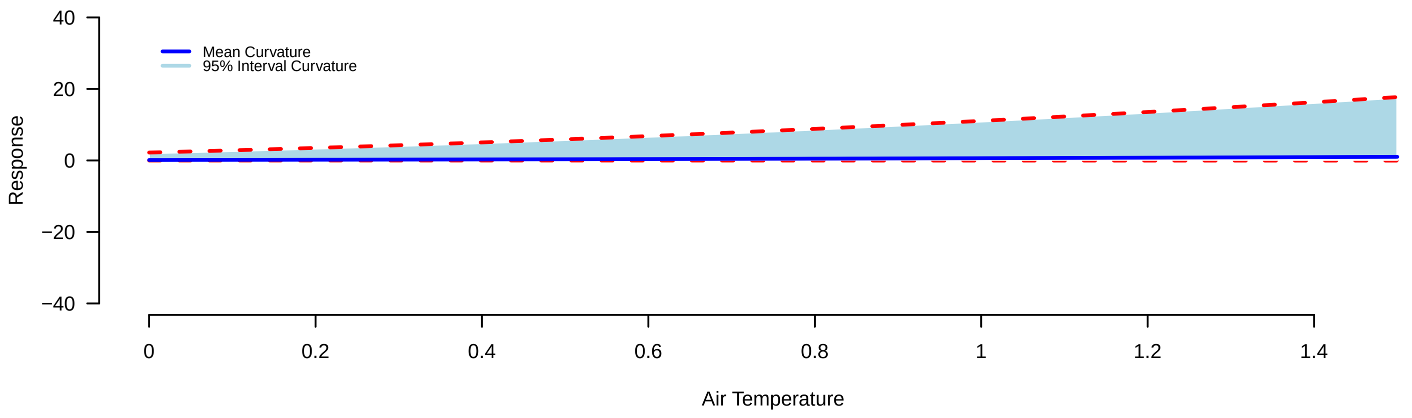

S6, Appendix Figure 484 : Curvature of Air Temperature lag 11

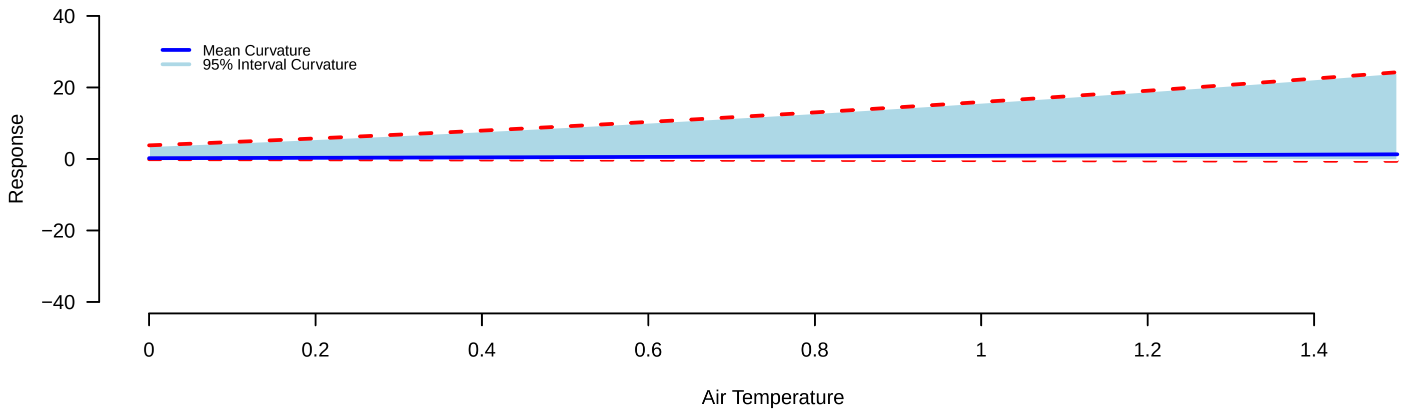

S6, Appendix Figure 485 : Curvature of Air Temperature lag 12

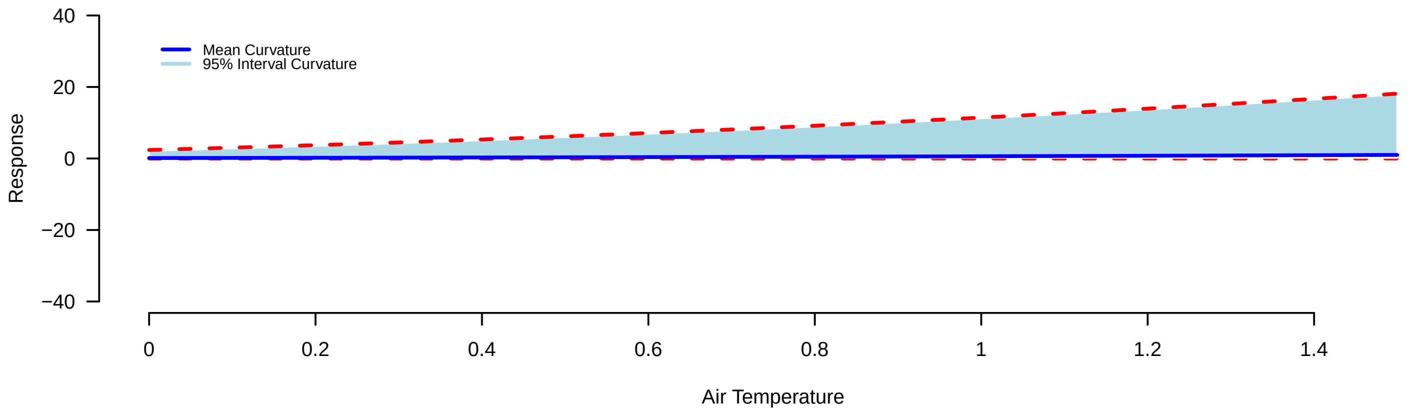

S6, Appendix Figure 486 : Curvature of Air Temperature lag 13

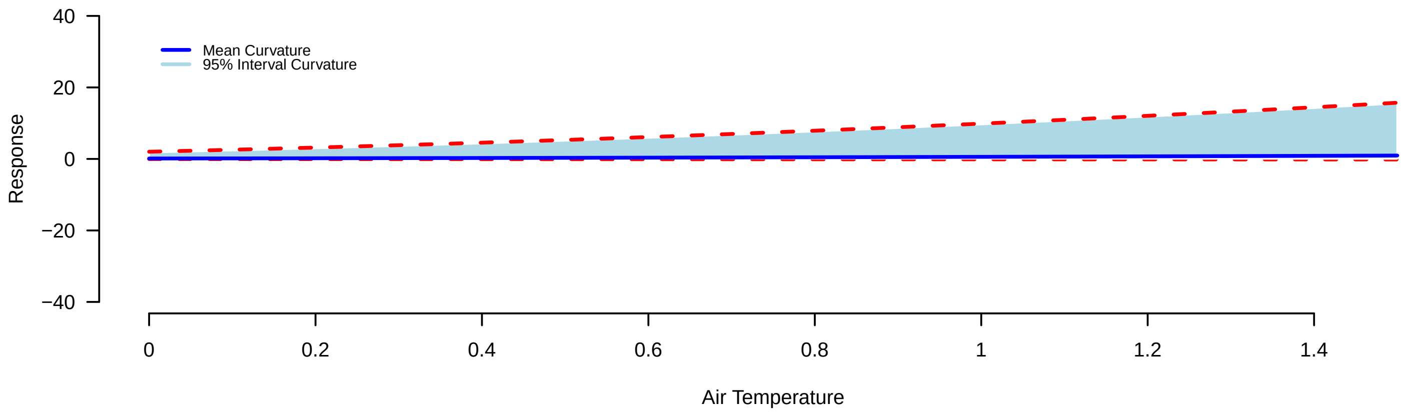

S6, Appendix Figure 487 : Curvature of Air Temperature lag 14

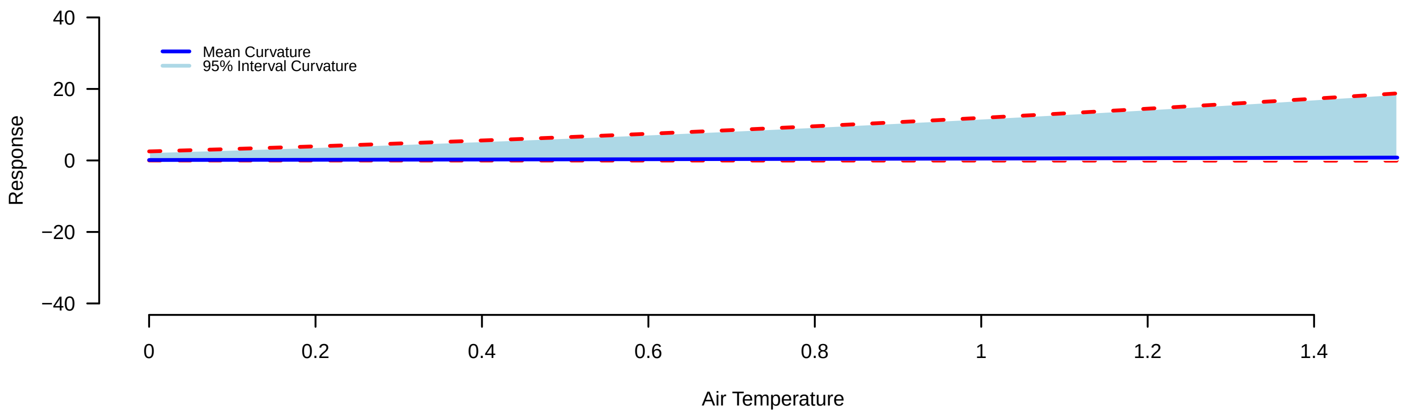

S6, Appendix Figure 488 : Curvature of Air Temperature lag 15

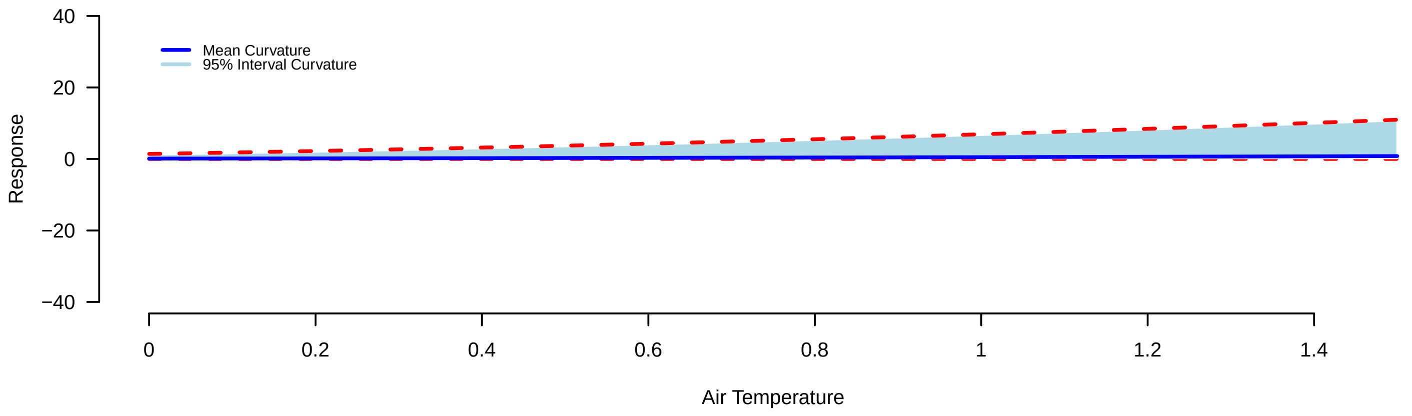

S6, Appendix Figure 489 : Curvature of Air Temperature lag 16

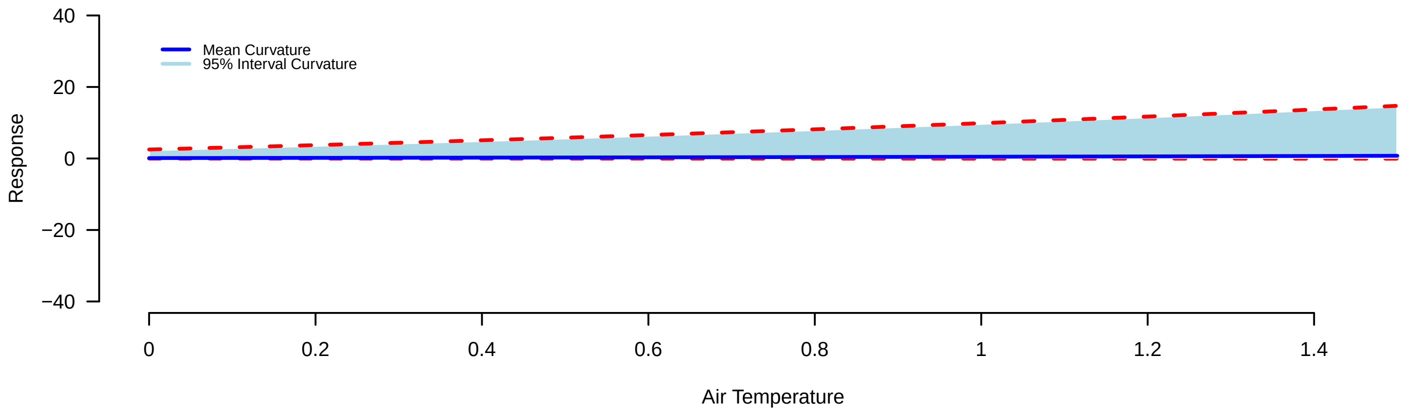

S6, Appendix Figure 490 : Curvature of Air Temperature lag 17

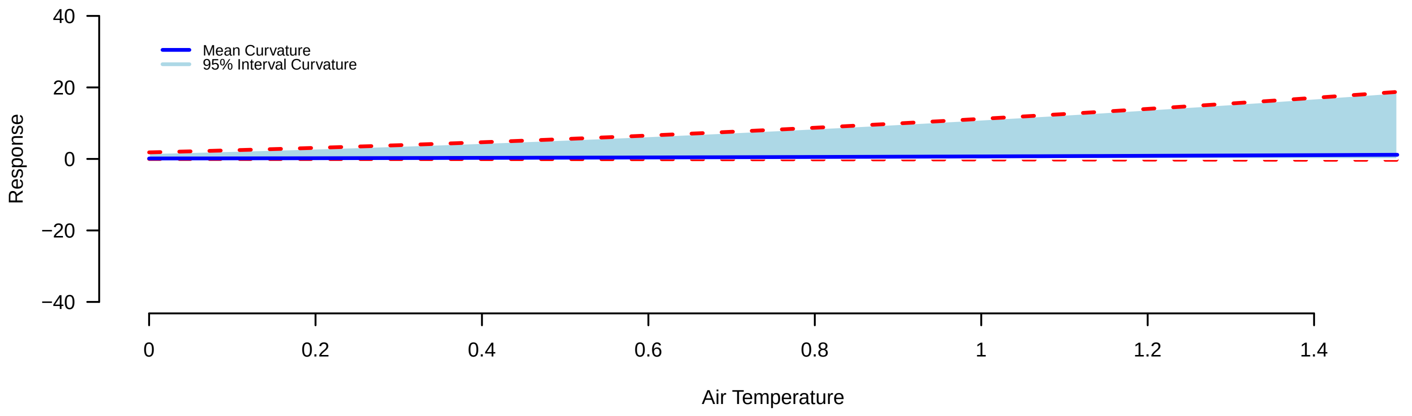

S6, Appendix Figure 491 : Curvature of Air Temperature lag 18

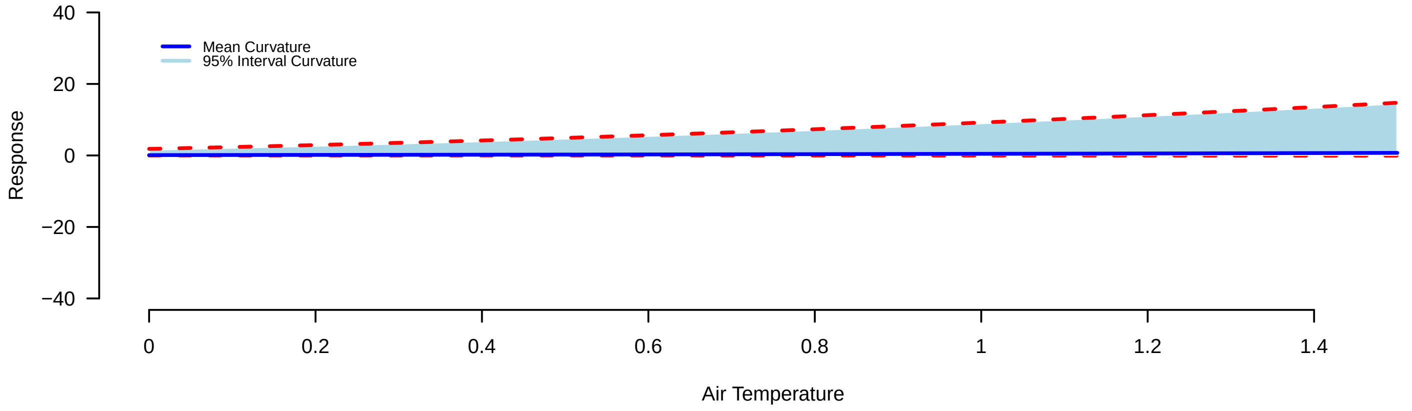

S6, Appendix Figure 492 : Curvature of Air Temperature lag 19

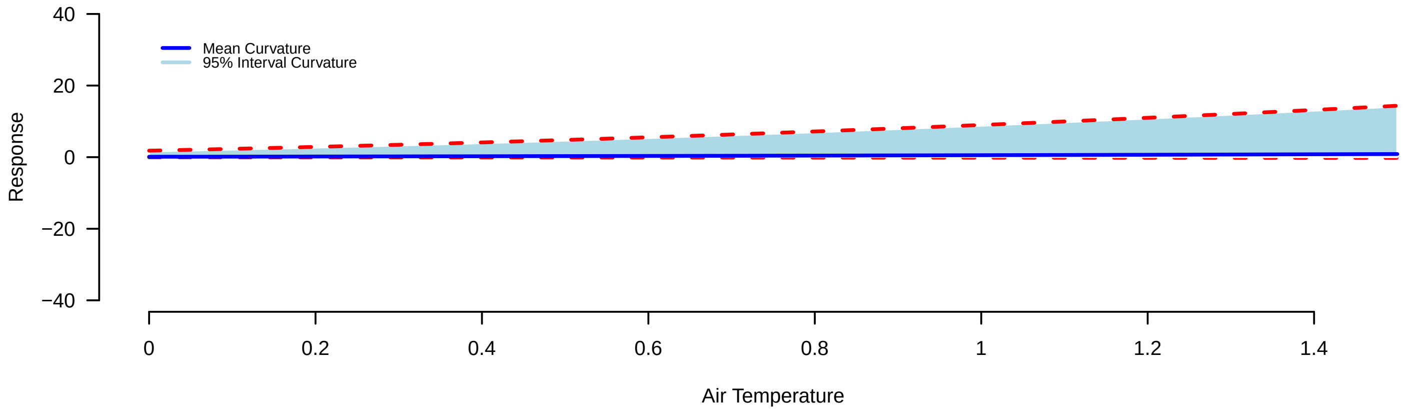

S6, Appendix Figure 493 : Curvature of Air Temperature lag 2

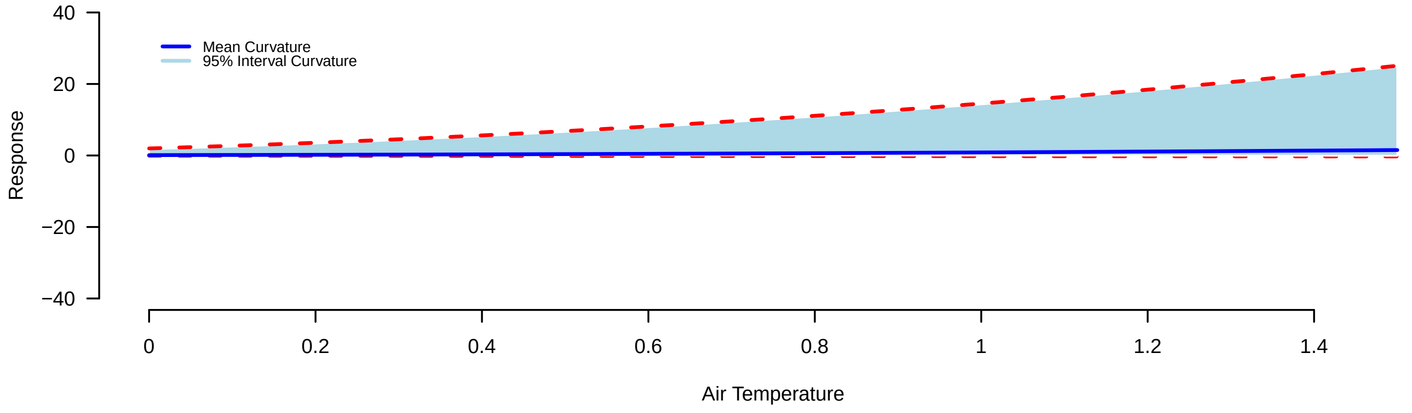

S6, Appendix Figure 494 : Curvature of Air Temperature lag 20

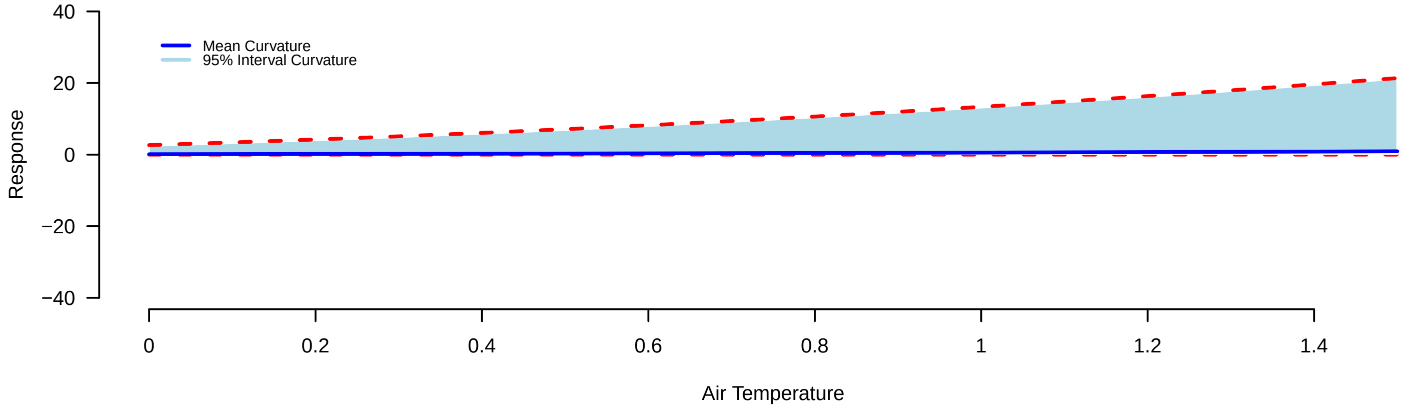

S6, Appendix Figure 495 : Curvature of Air Temperature lag 3

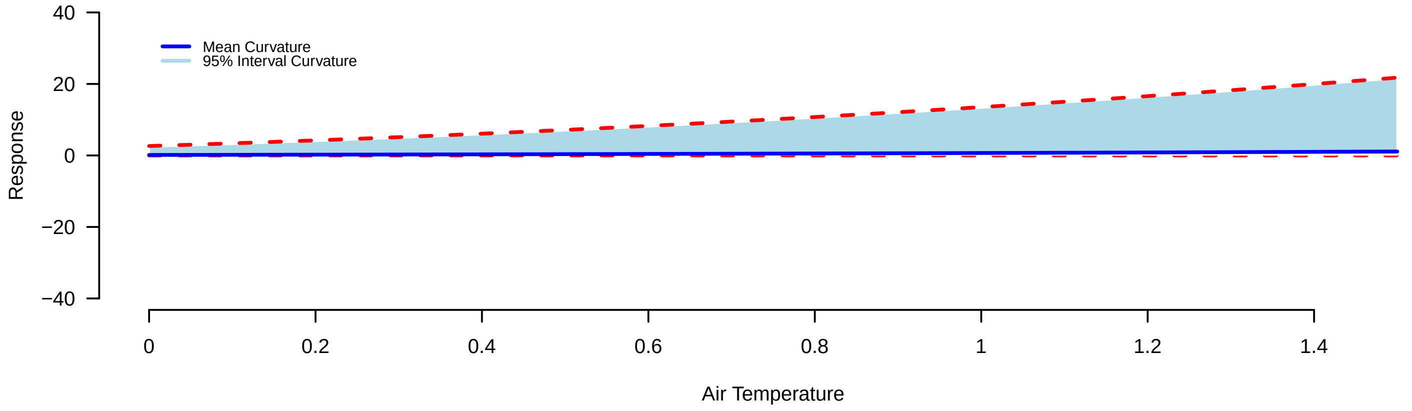

S6, Appendix Figure 496 : Curvature of Air Temperature lag 4

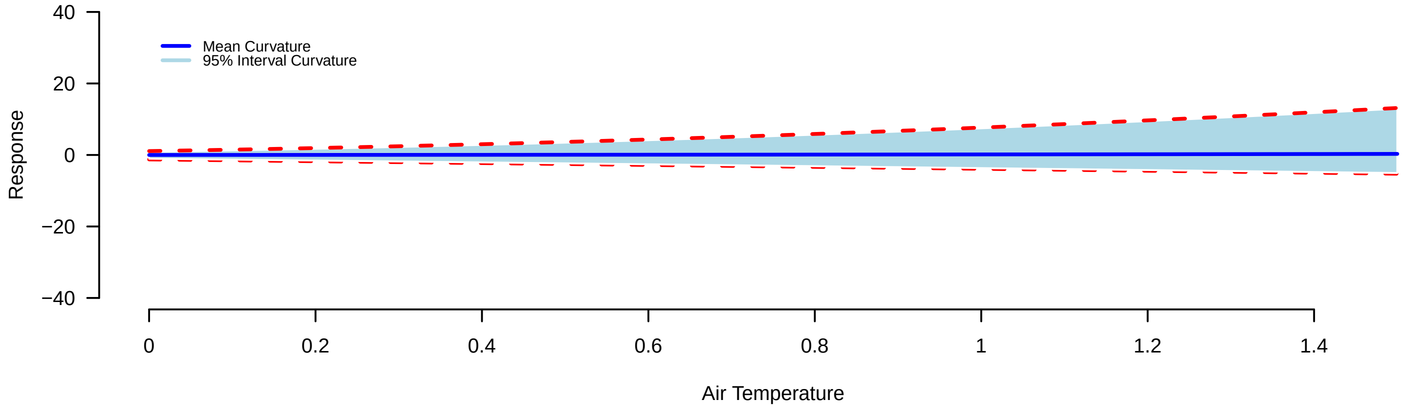

S6, Appendix Figure 497 : Curvature of Air Temperature lag 5

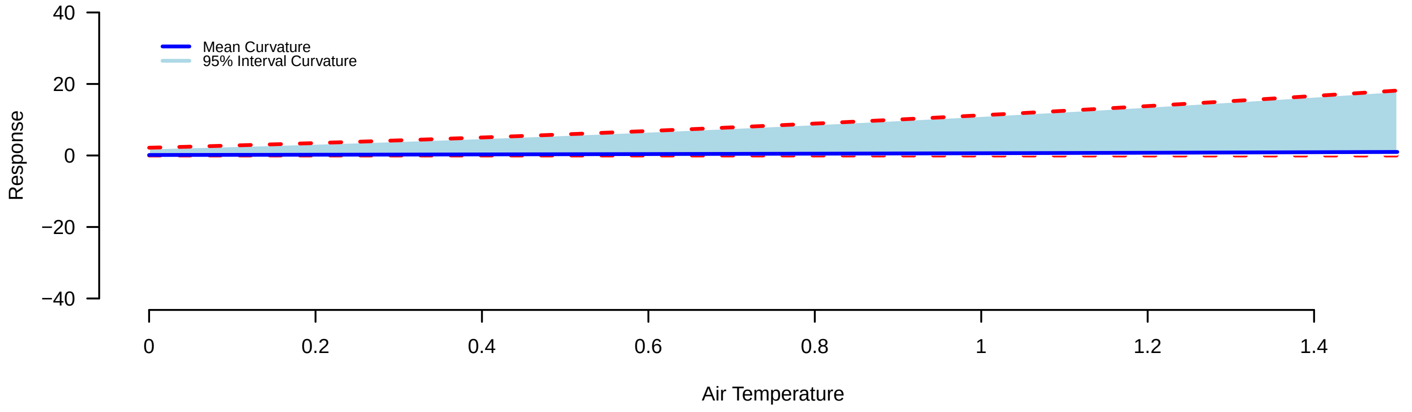

S6, Appendix Figure 498 : Curvature of Air Temperature lag 6

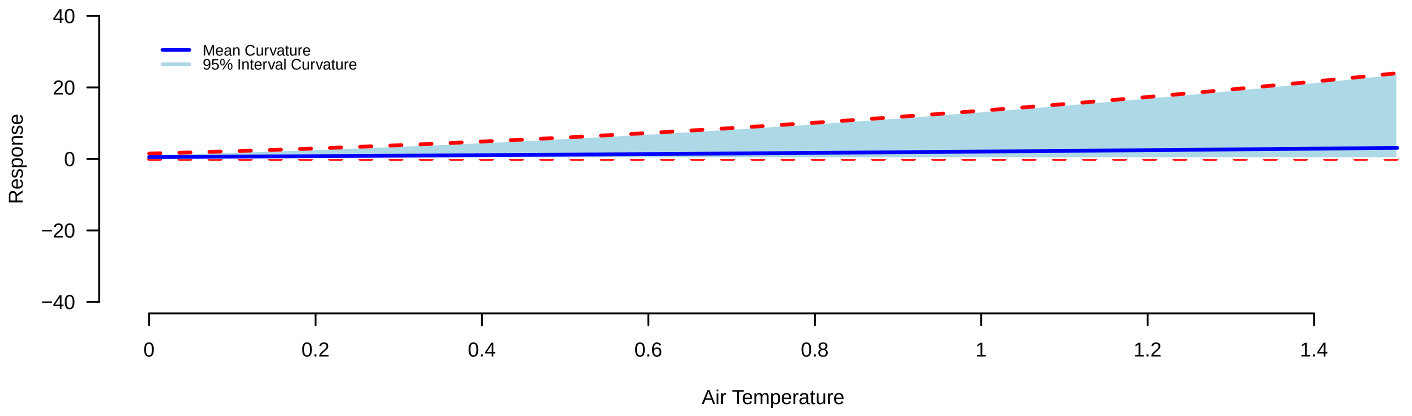

S6, Appendix Figure 499 : Curvature of Air Temperature lag 7

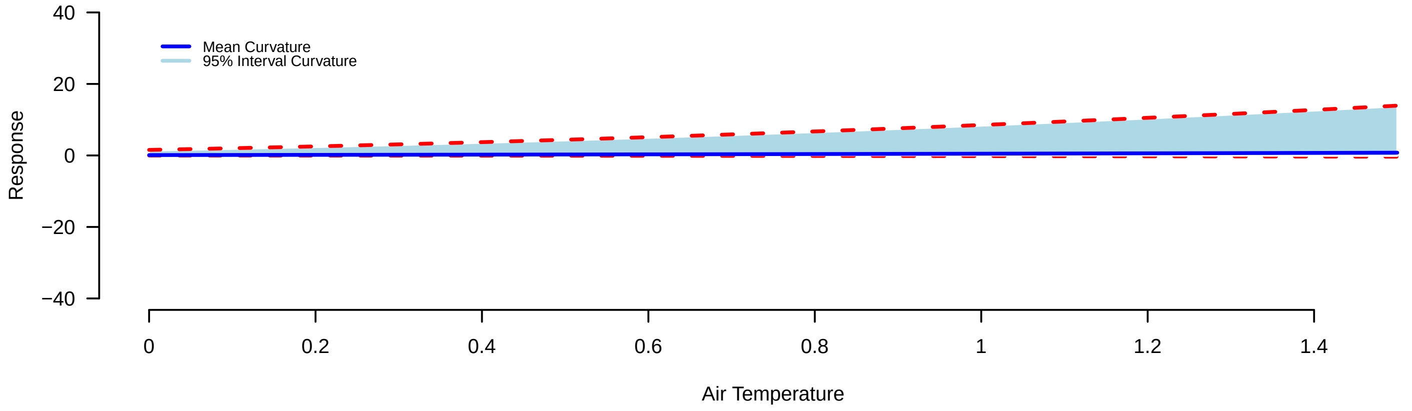

S6, Appendix Figure 500 : Curvature of Air Temperature lag 8

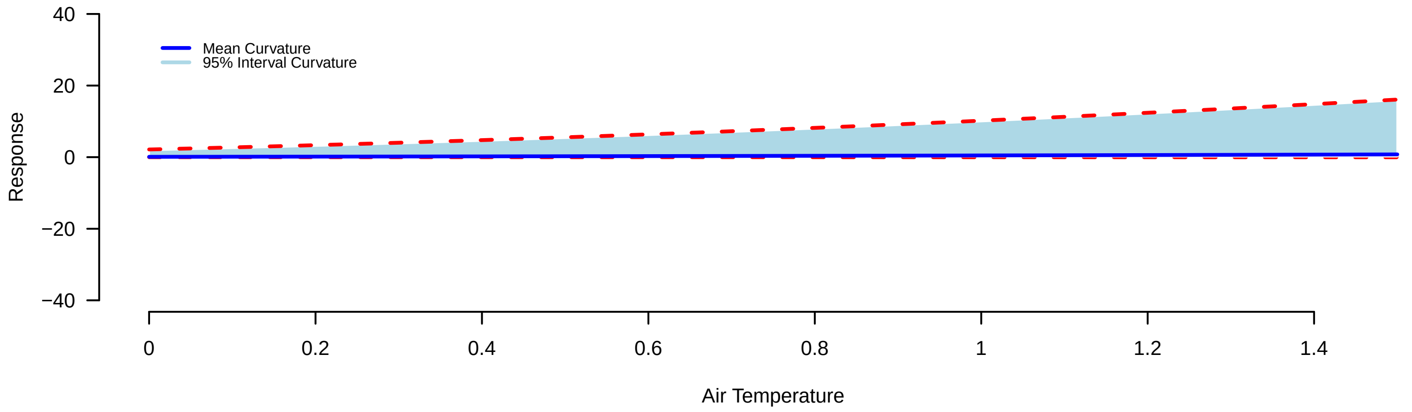

S6, Appendix Figure 501 : Curvature of Air Temperature lag 9

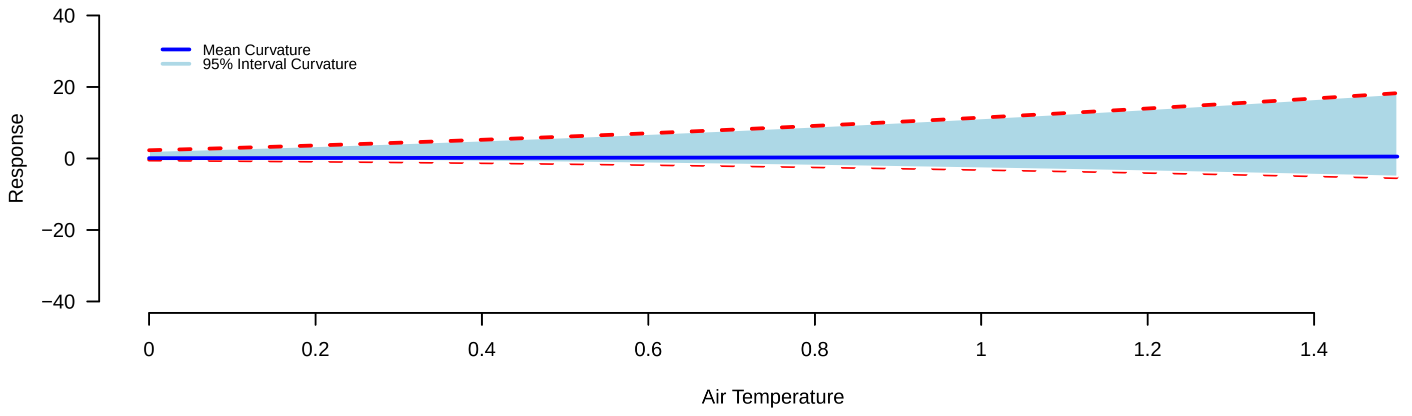

S6, Appendix Figure 502 : Curvature of Air Temperature:Absolute Humidity lag 1

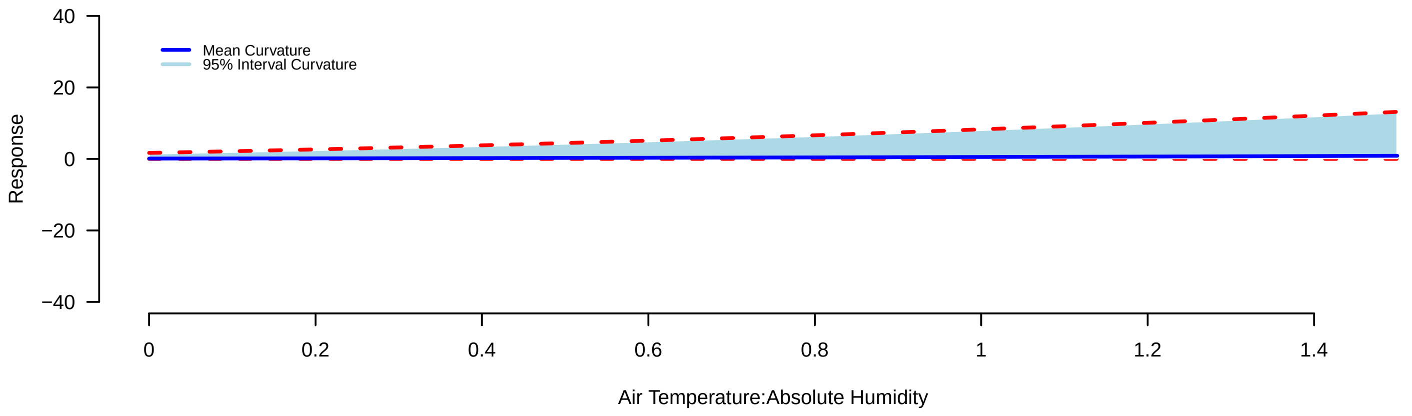

S6, Appendix Figure 503 : Curvature of Air Temperature:Absolute Humidity lag 10

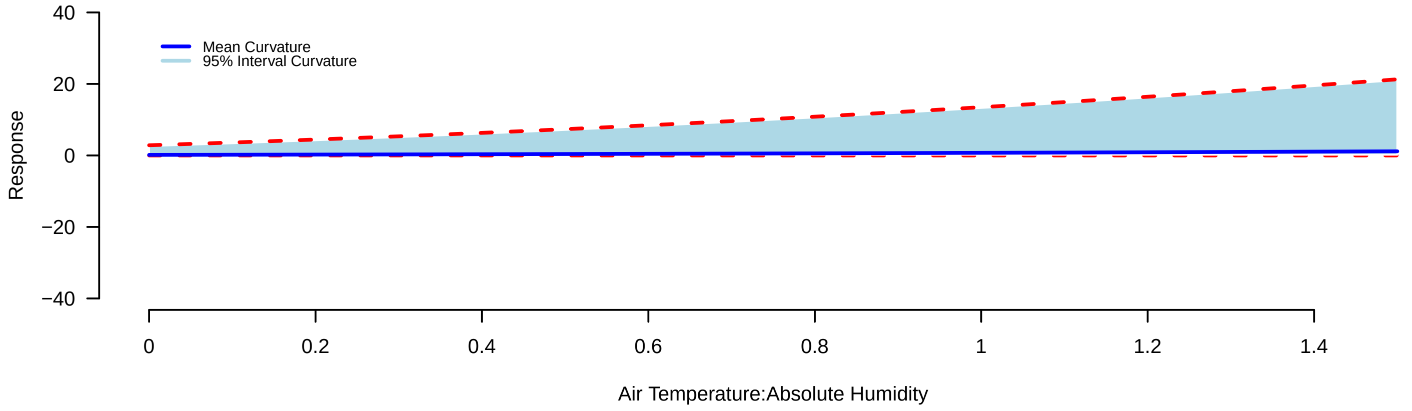

S6, Appendix Figure 504 : Curvature of Air Temperature:Absolute Humidity lag 11

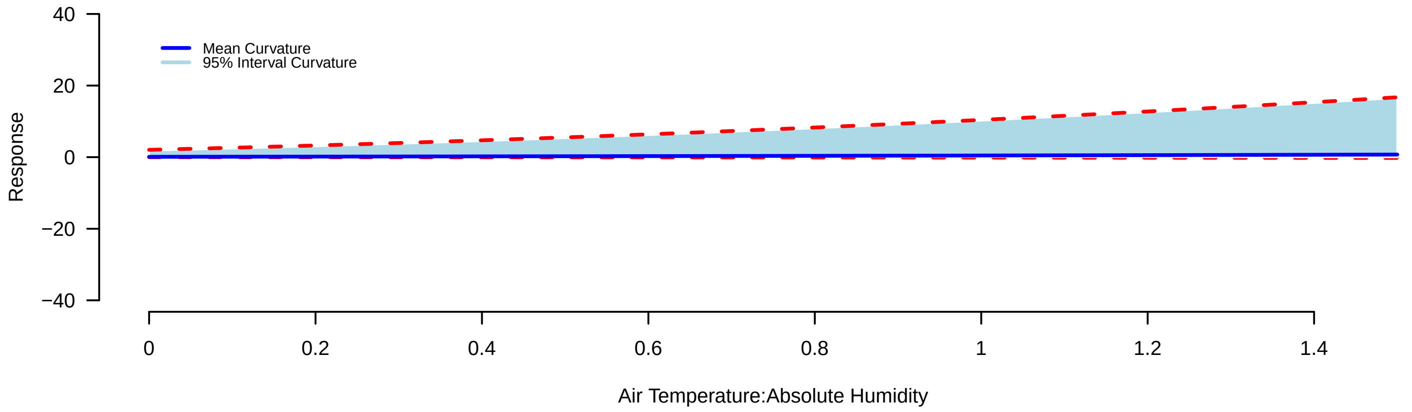

S6, Appendix Figure 505 : Curvature of Air Temperature:Absolute Humidity lag 12

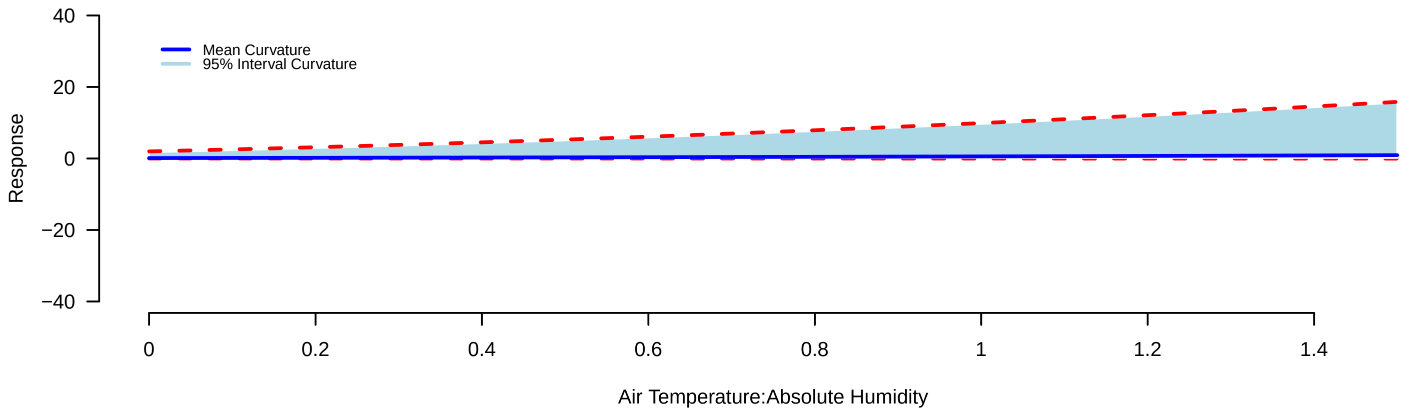

S6, Appendix Figure 506 : Curvature of Air Temperature:Absolute Humidity lag 13

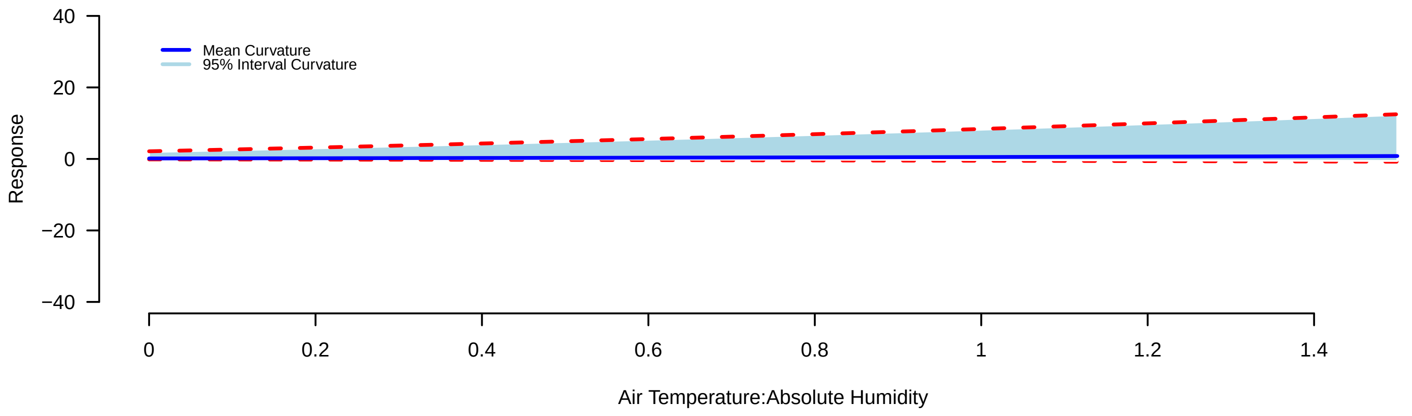

S6, Appendix Figure 507 : Curvature of Air Temperature:Absolute Humidity lag 14

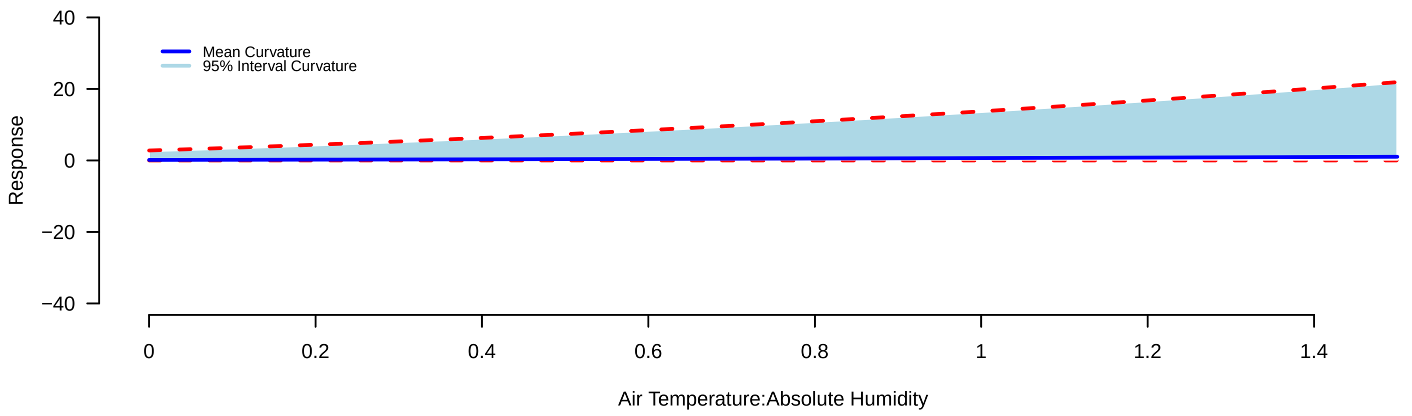

S6, Appendix Figure 508 : Curvature of Air Temperature:Absolute Humidity lag 15

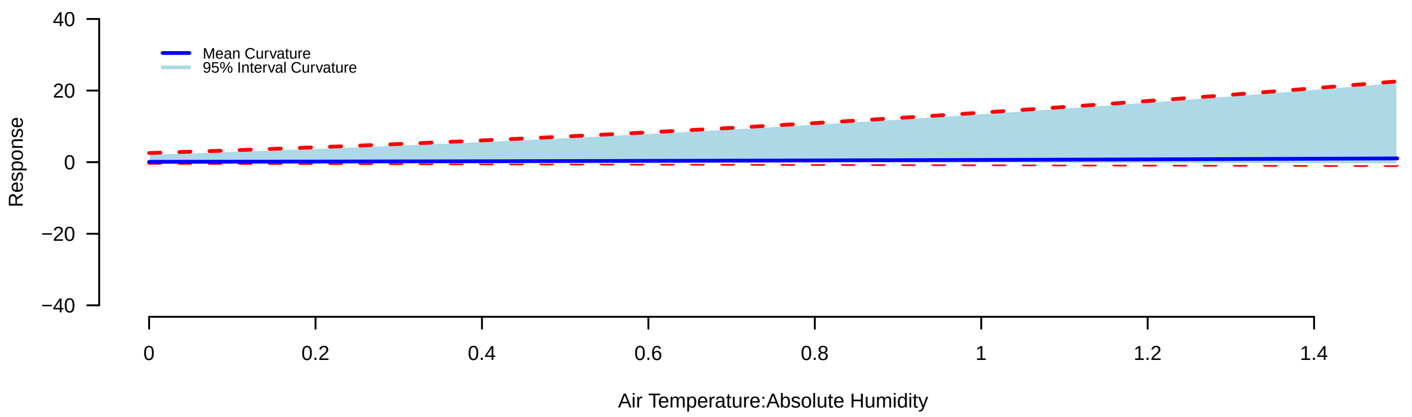

S6, Appendix Figure 509 : Curvature of Air Temperature:Absolute Humidity lag 16

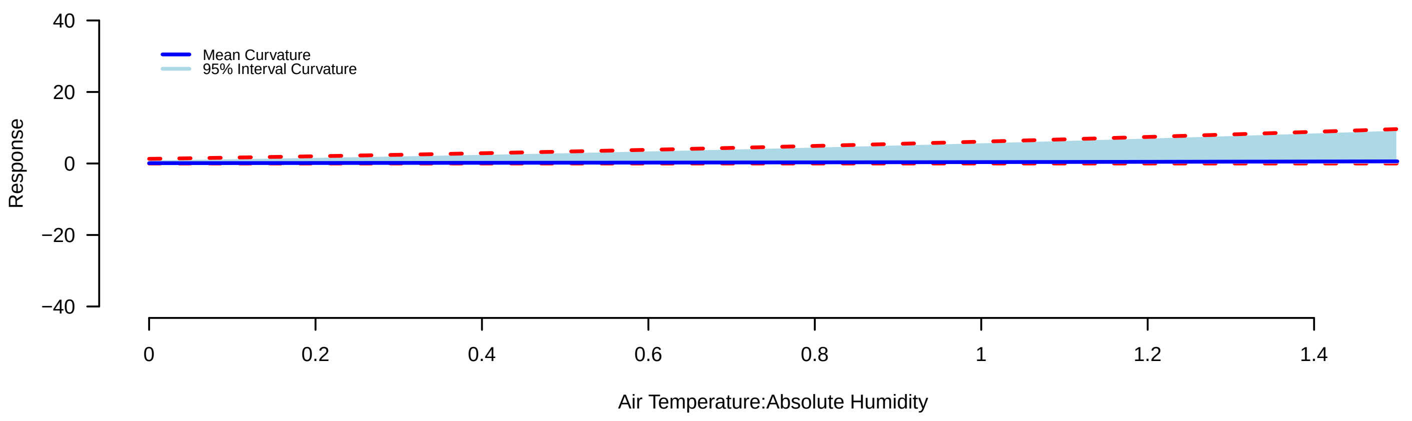

S6, Appendix Figure 510 : Curvature of Air Temperature:Absolute Humidity lag 17

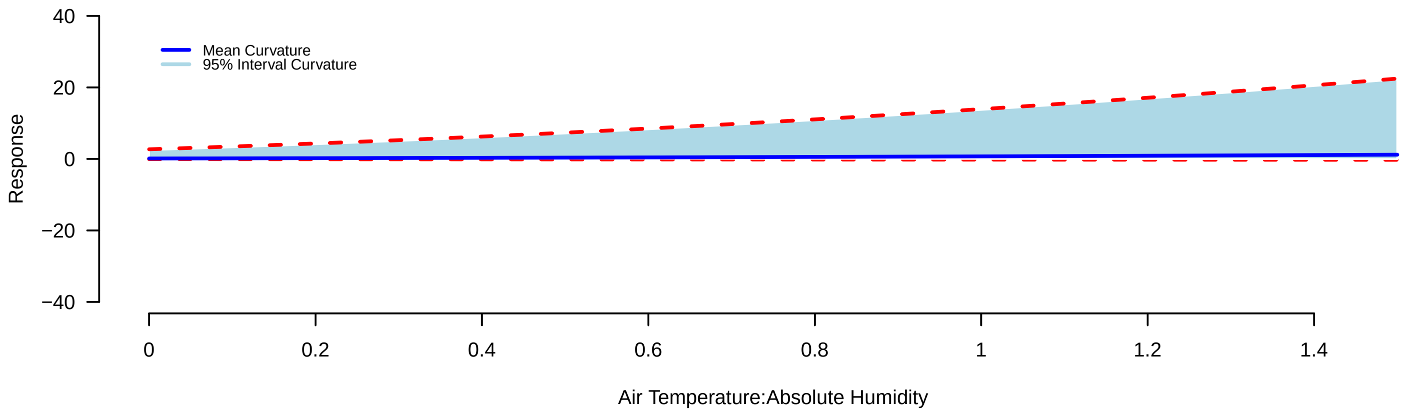

S6, Appendix Figure 511 : Curvature of Air Temperature:Absolute Humidity lag 18

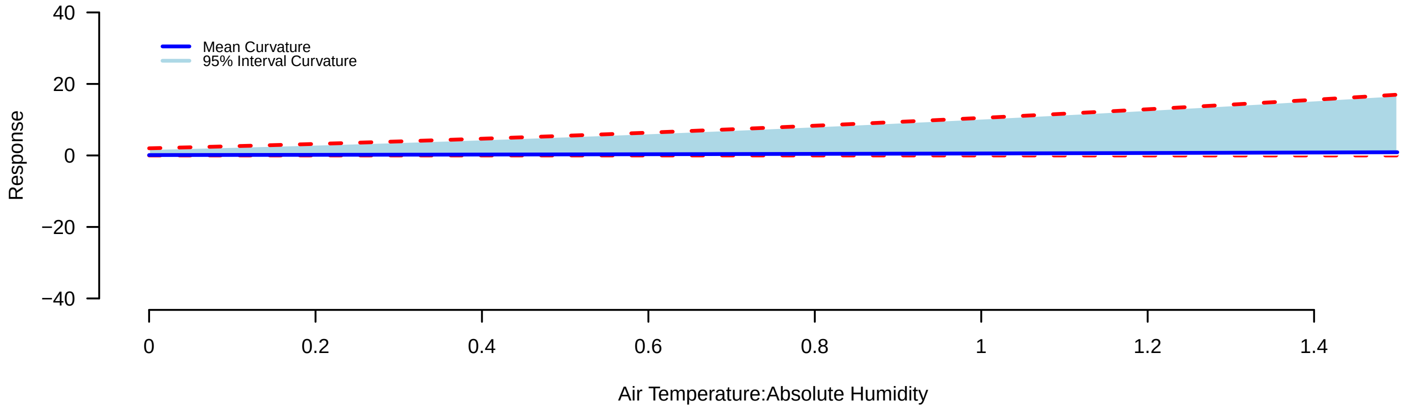

S6, Appendix Figure 512 : Curvature of Air Temperature:Absolute Humidity lag 19

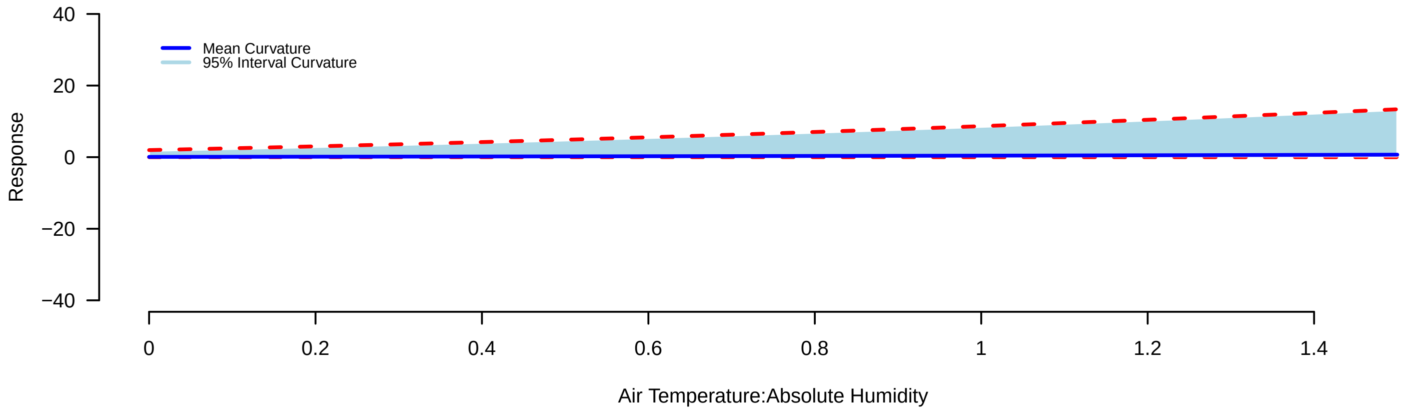

S6, Appendix Figure 513 : Curvature of Air Temperature:Absolute Humidity lag 2

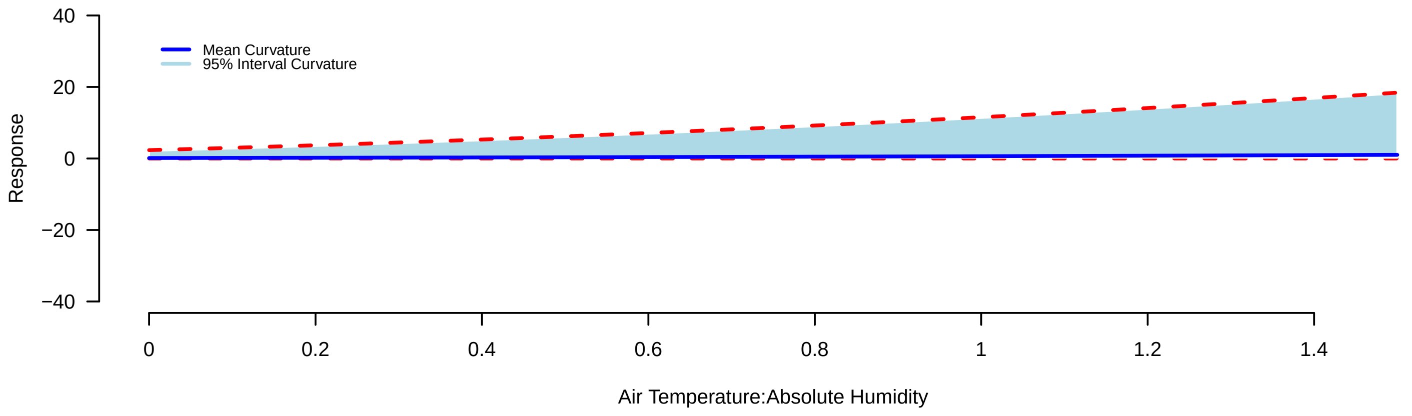

S6, Appendix Figure 514 : Curvature of Air Temperature:Absolute Humidity lag 20

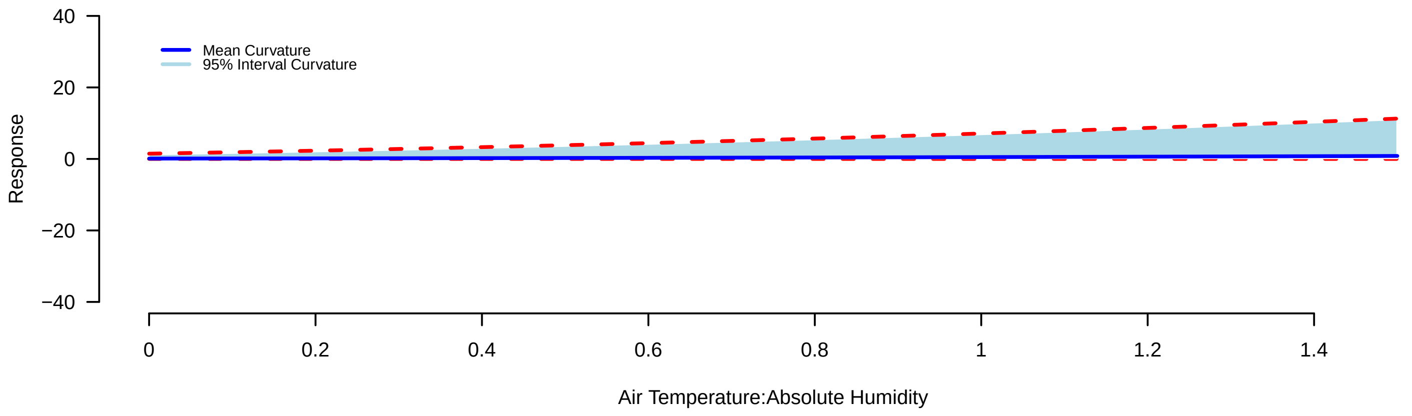

S6, Appendix Figure 515 : Curvature of Air Temperature:Absolute Humidity lag 3

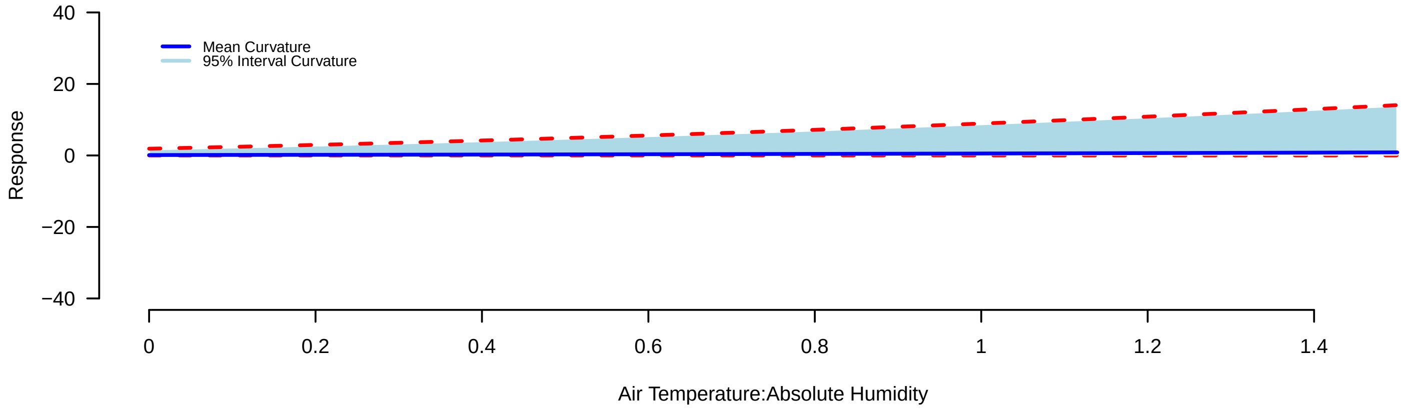

S6, Appendix Figure 516 : Curvature of Air Temperature:Absolute Humidity lag 4

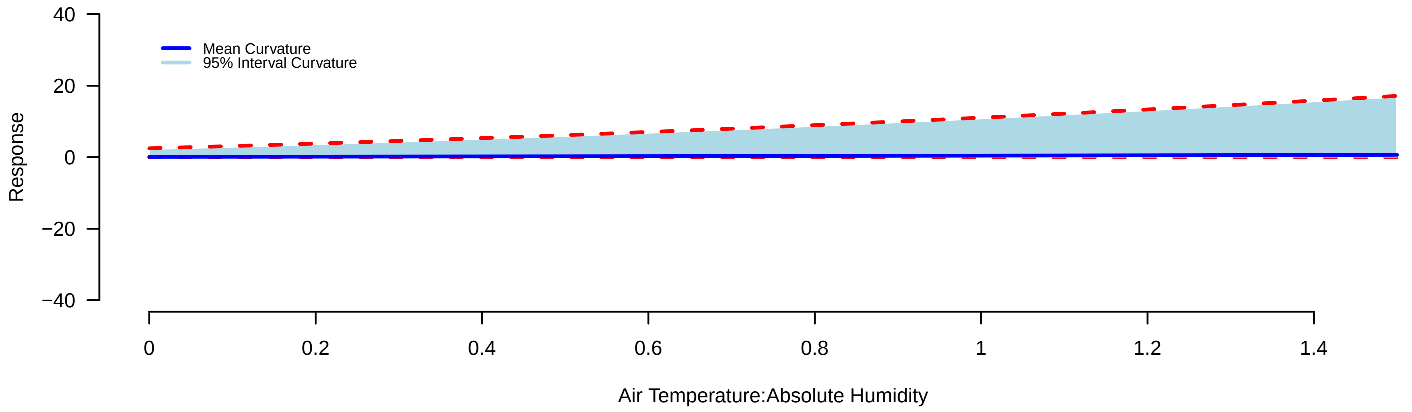

S6, Appendix Figure 517 : Curvature of Air Temperature:Absolute Humidity lag 5

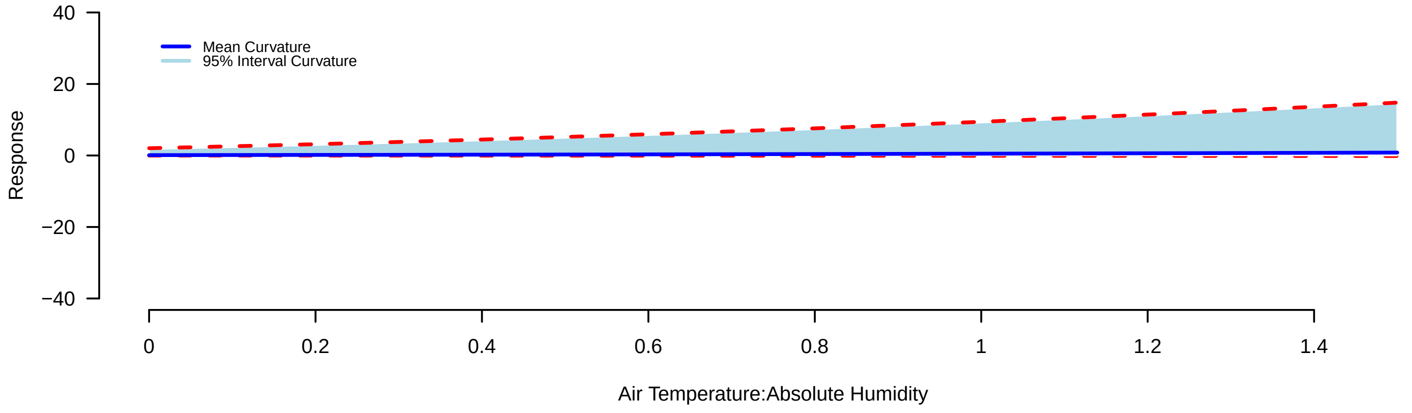

S6, Appendix Figure 518 : Curvature of Air Temperature:Absolute Humidity lag 6

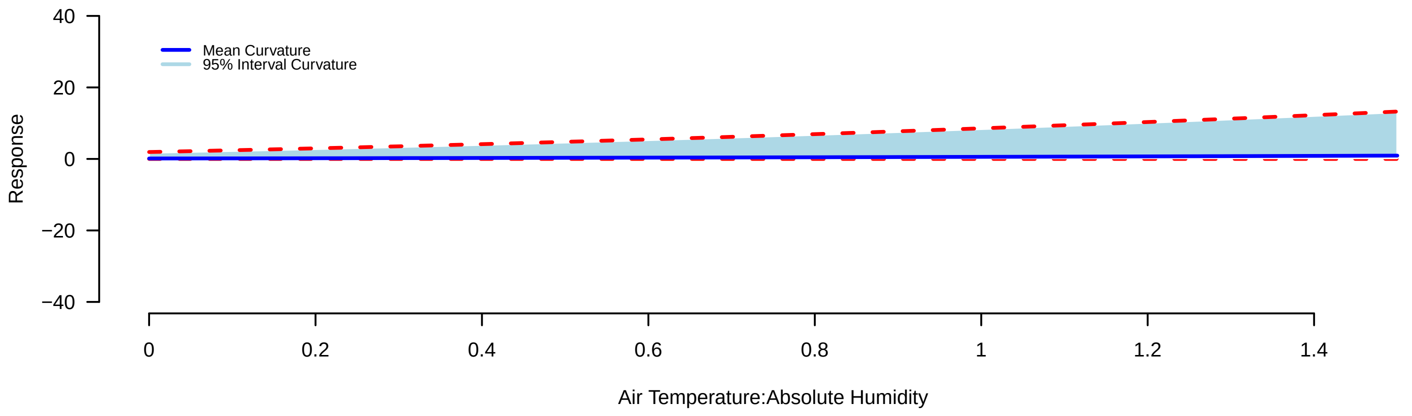

S6, Appendix Figure 519 : Curvature of Air Temperature:Absolute Humidity lag 7

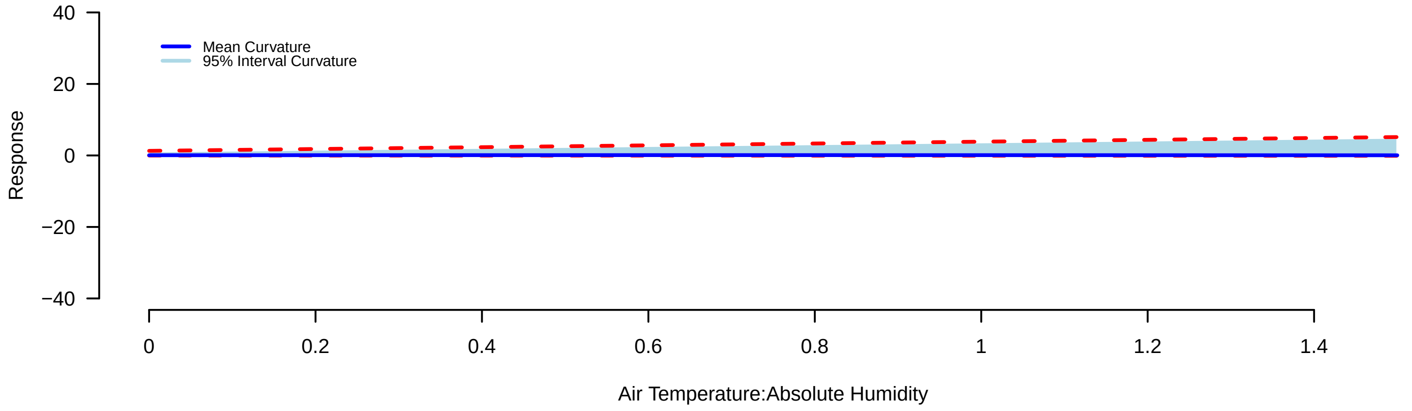

S6, Appendix Figure 520 : Curvature of Air Temperature:Absolute Humidity lag 8

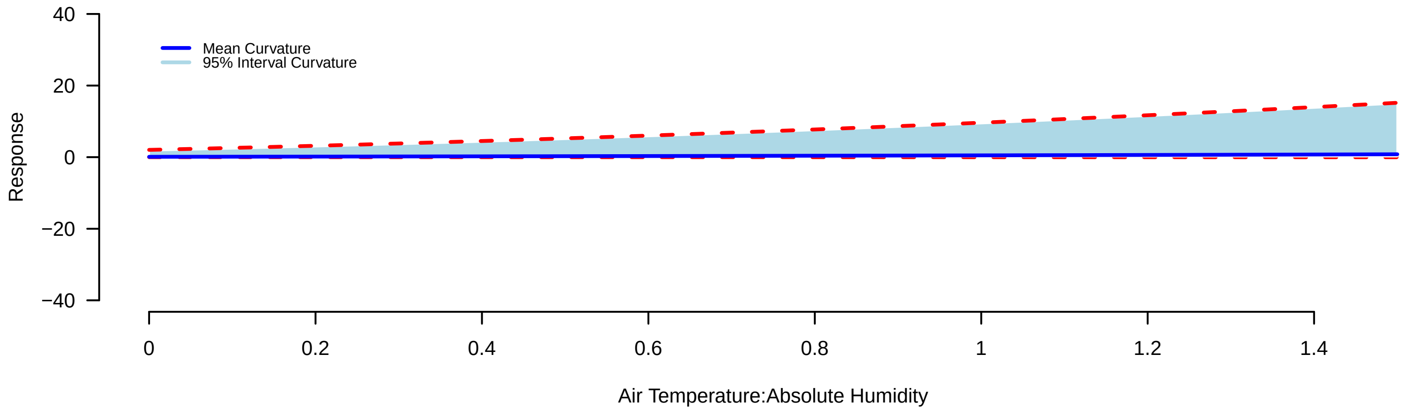

S6, Appendix Figure 521 : Curvature of Air Temperature:Absolute Humidity lag 9

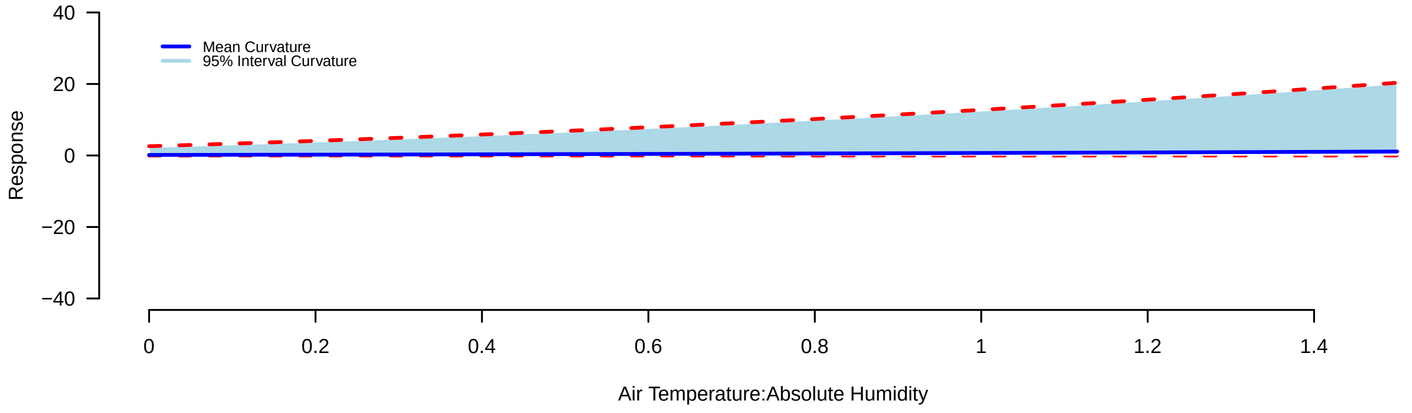

S6, Appendix Figure 522 : Curvature of Air Temperature:Relative Humidity lag 1

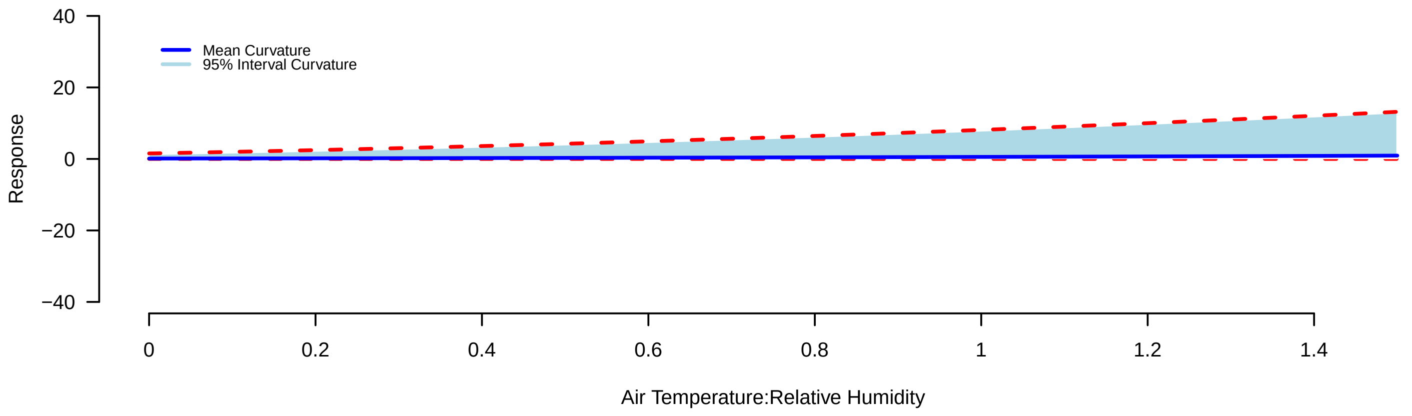

S6, Appendix Figure 523 : Curvature of Air Temperature:Relative Humidity lag 10

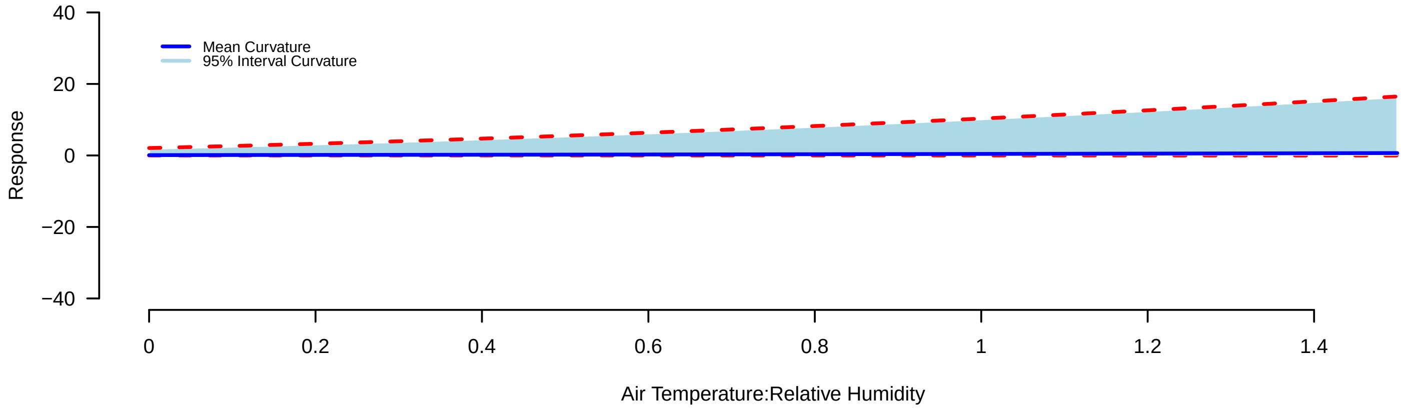

S6, Appendix Figure 524 : Curvature of Air Temperature:Relative Humidity lag 11

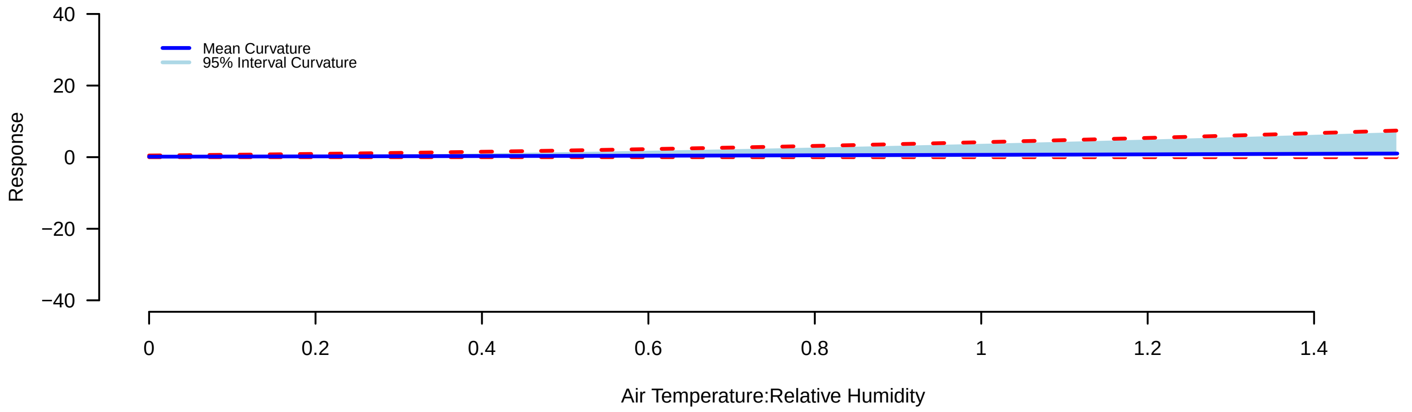

S6, Appendix Figure 525 : Curvature of Air Temperature:Relative Humidity lag 12

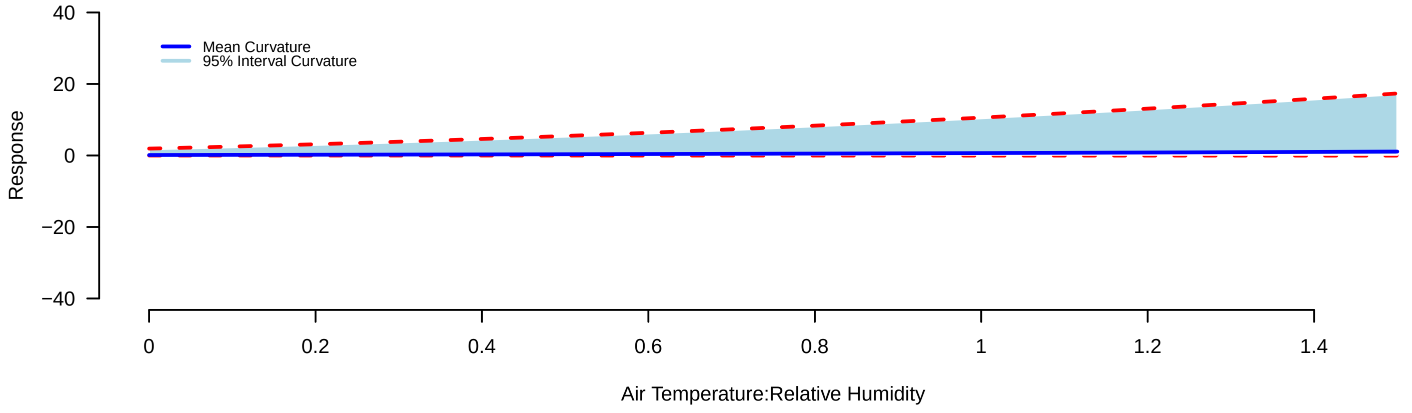

S6, Appendix Figure 526 : Curvature of Air Temperature:Relative Humidity lag 13

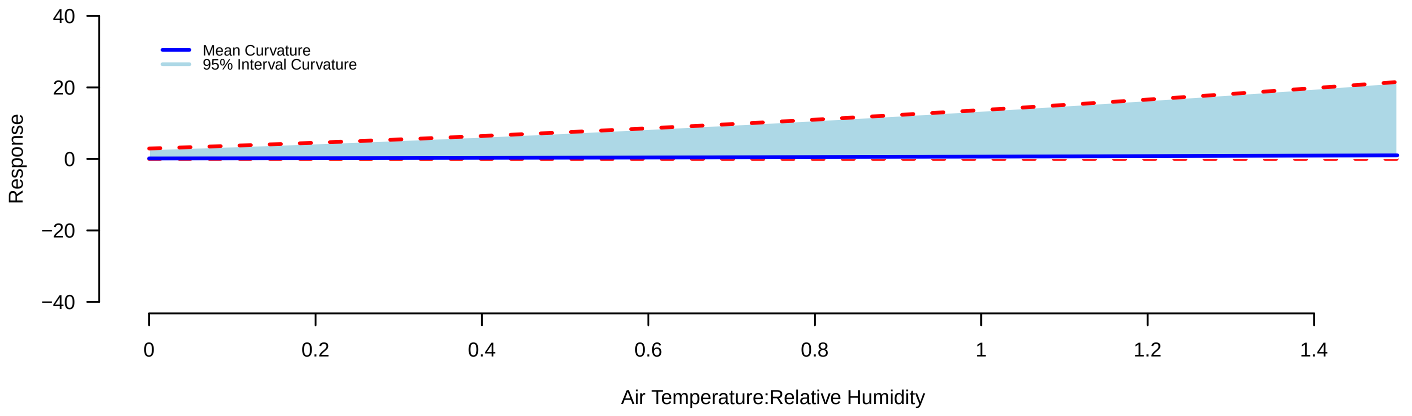

S6, Appendix Figure 527 : Curvature of Air Temperature:Relative Humidity lag 14

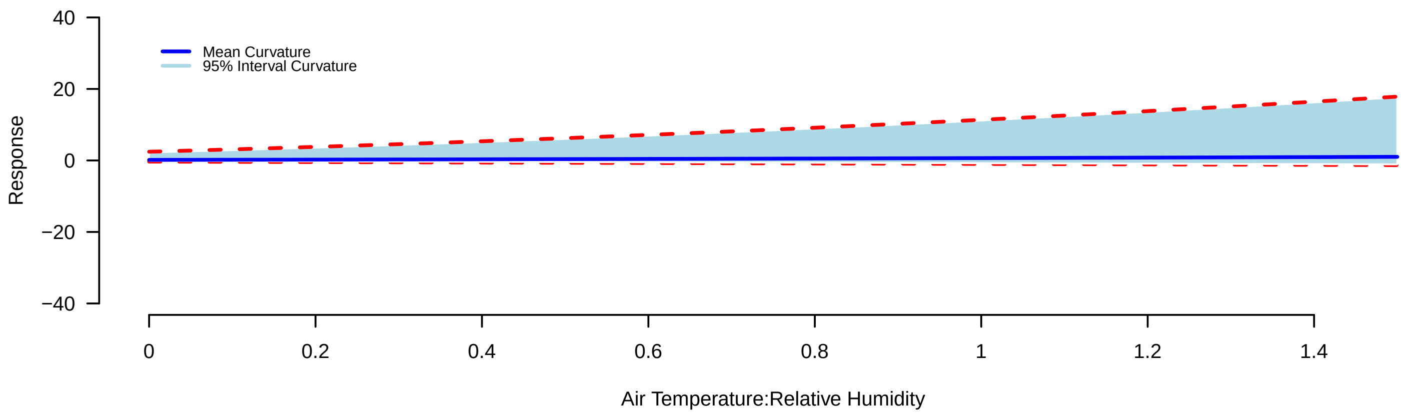

S6, Appendix Figure 528 : Curvature of Air Temperature:Relative Humidity lag 15

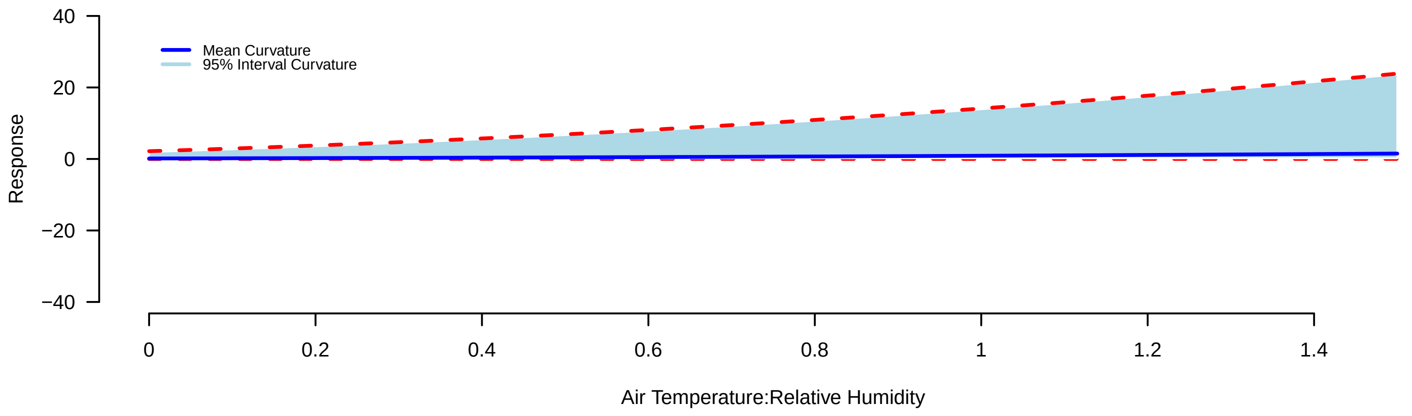

S6, Appendix Figure 529 : Curvature of Air Temperature:Relative Humidity lag 16

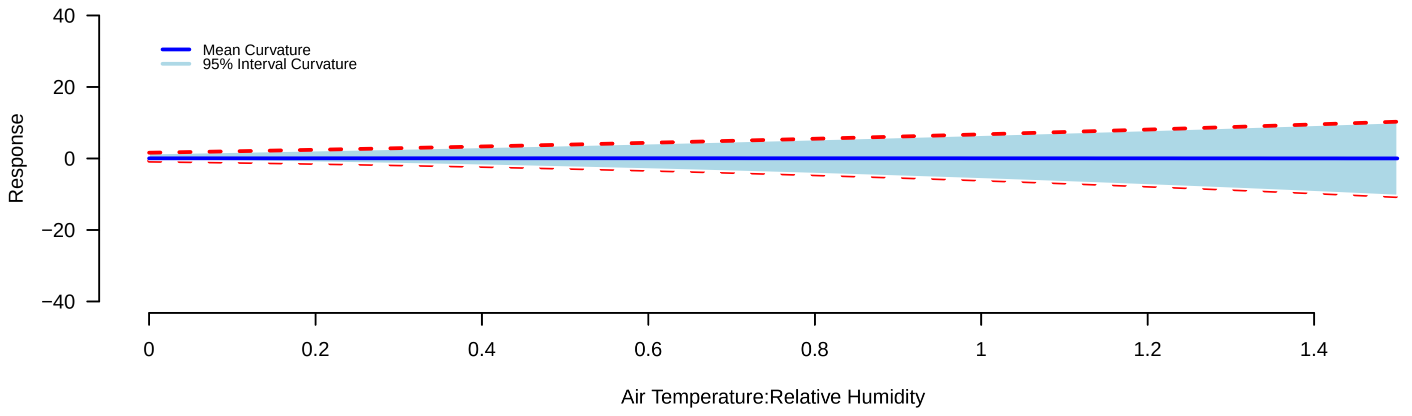

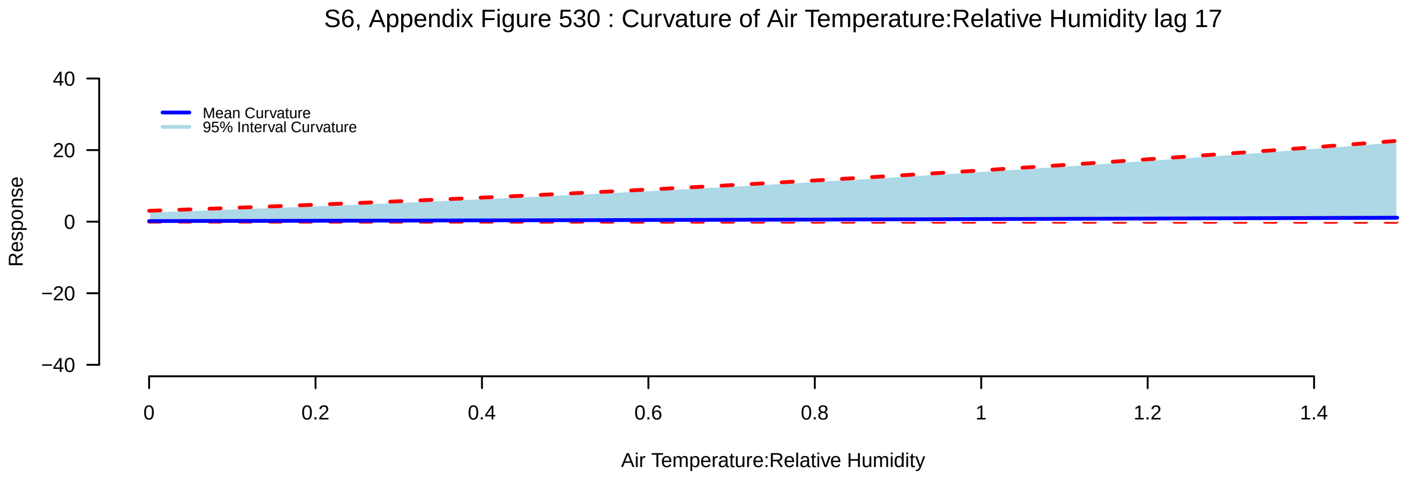

S6, Appendix Figure 530 : Curvature of Air Temperature:Relative Humidity lag 17
Mean Curvature
95% Interval Curvature
Response
Air Temperature:Relative Humidity

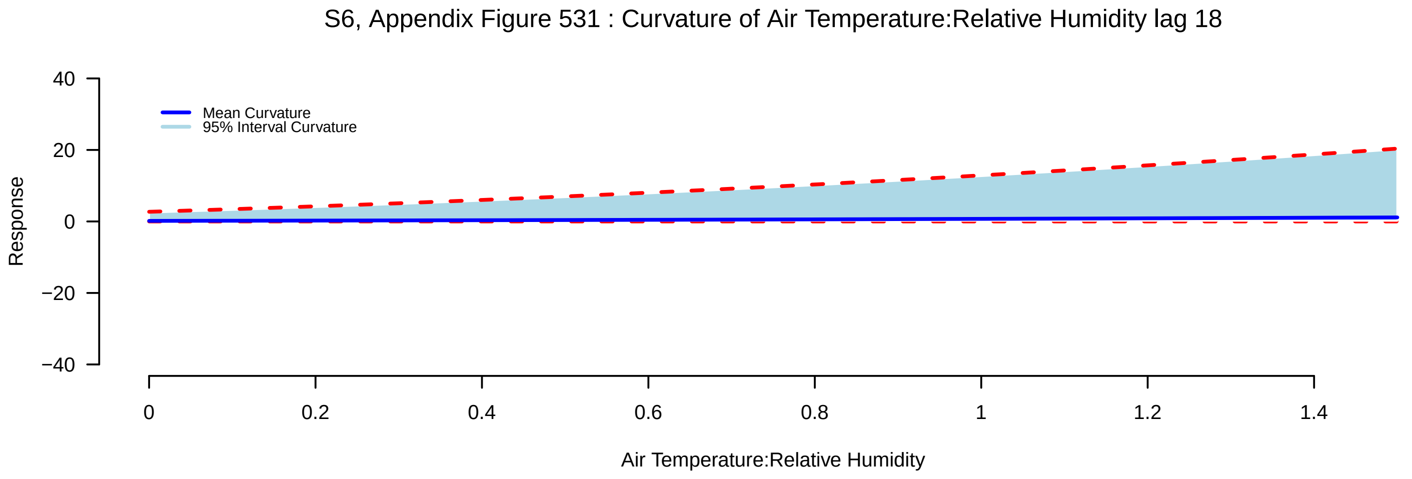

S6, Appendix Figure 531 : Curvature of Air Temperature:Relative Humidity lag 18
Mean Curvature
95% Interval Curvature
Response
Air Temperature:Relative Humidity

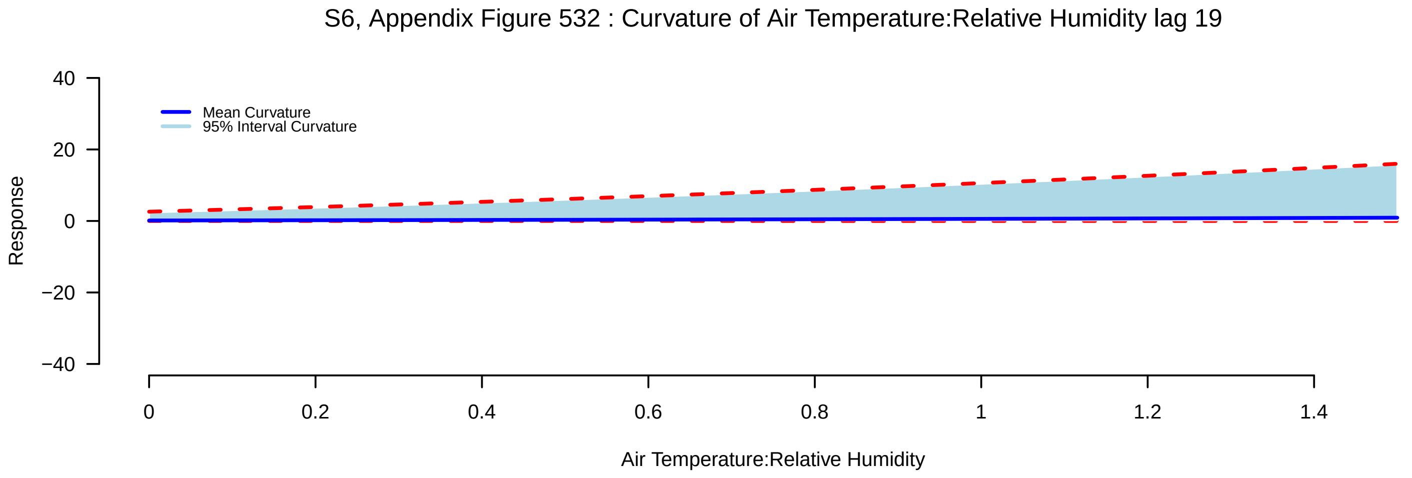

S6, Appendix Figure 532 : Curvature of Air Temperature:Relative Humidity lag 19
Mean Curvature
95% Interval Curvature
Response
Air Temperature:Relative Humidity

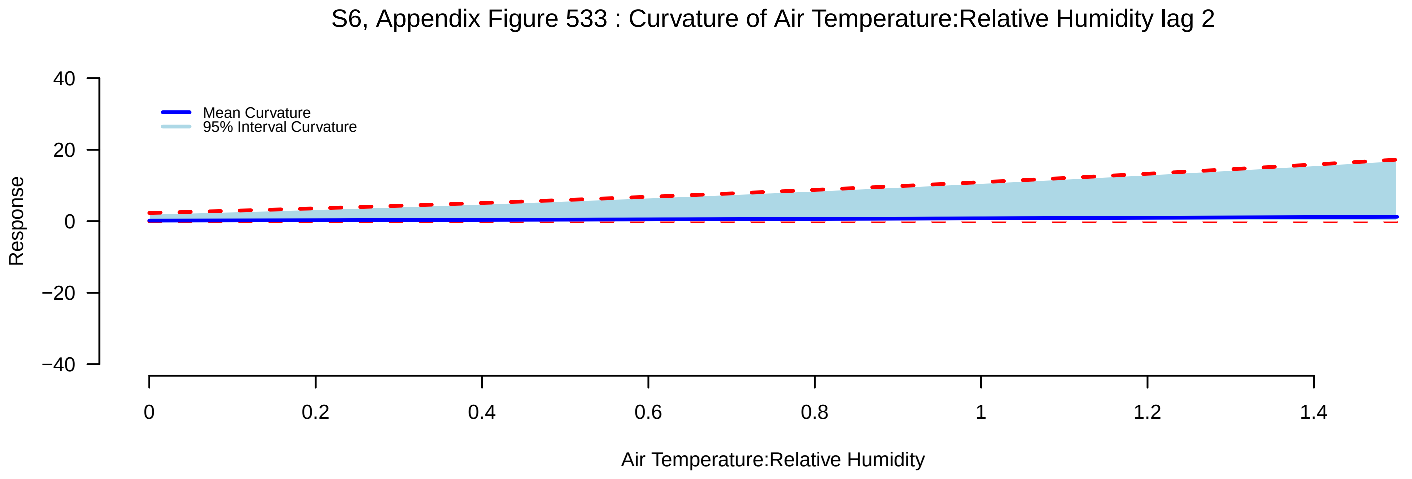

S6, Appendix Figure 533 : Curvature of Air Temperature:Relative Humidity lag 2
Mean Curvature
95% Interval Curvature
Response
Air Temperature:Relative Humidity

S6, Appendix Figure 534 : Curvature of Air Temperature:Relative Humidity lag 20

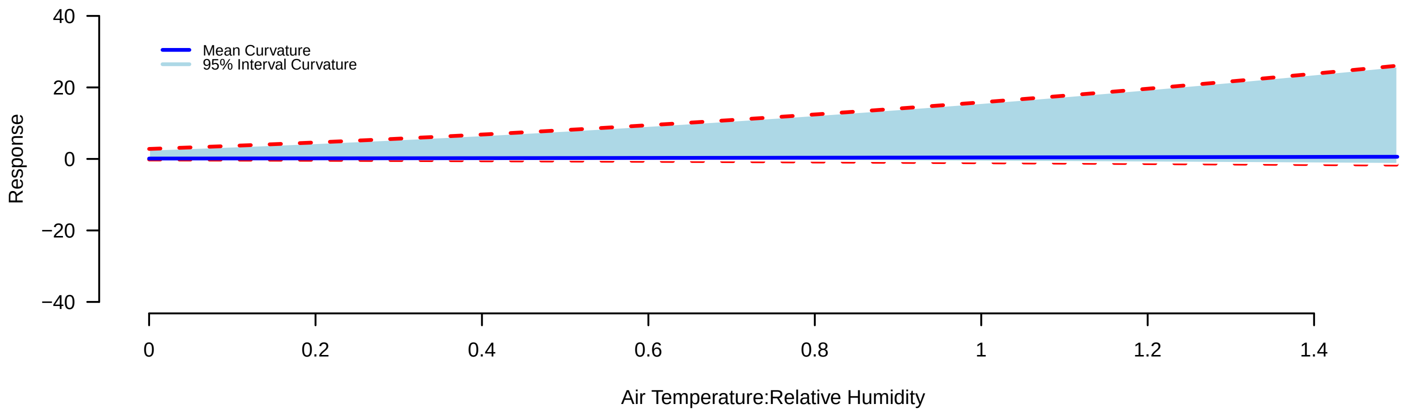

S6, Appendix Figure 535 : Curvature of Air Temperature:Relative Humidity lag 3

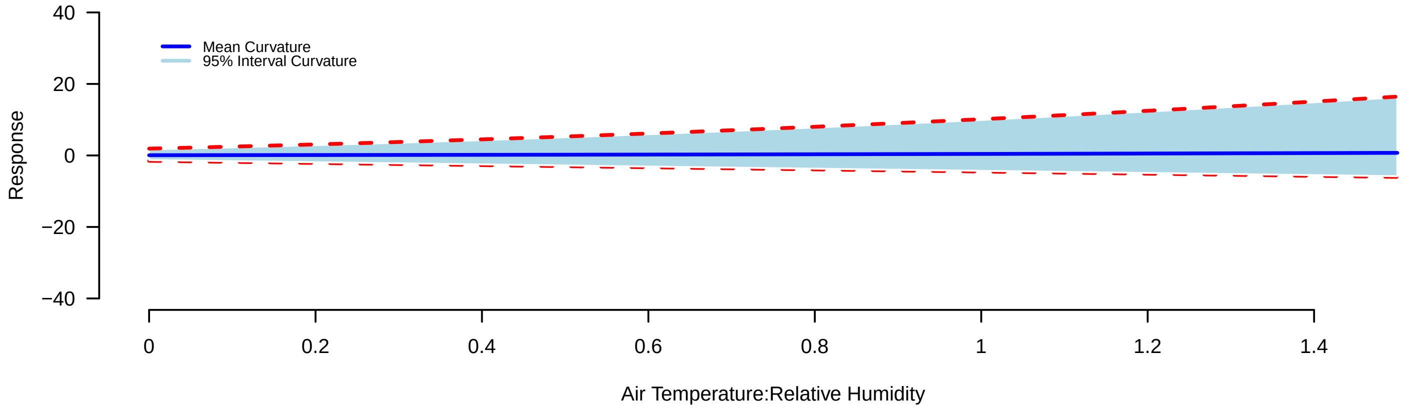

S6, Appendix Figure 536 : Curvature of Air Temperature:Relative Humidity lag 4

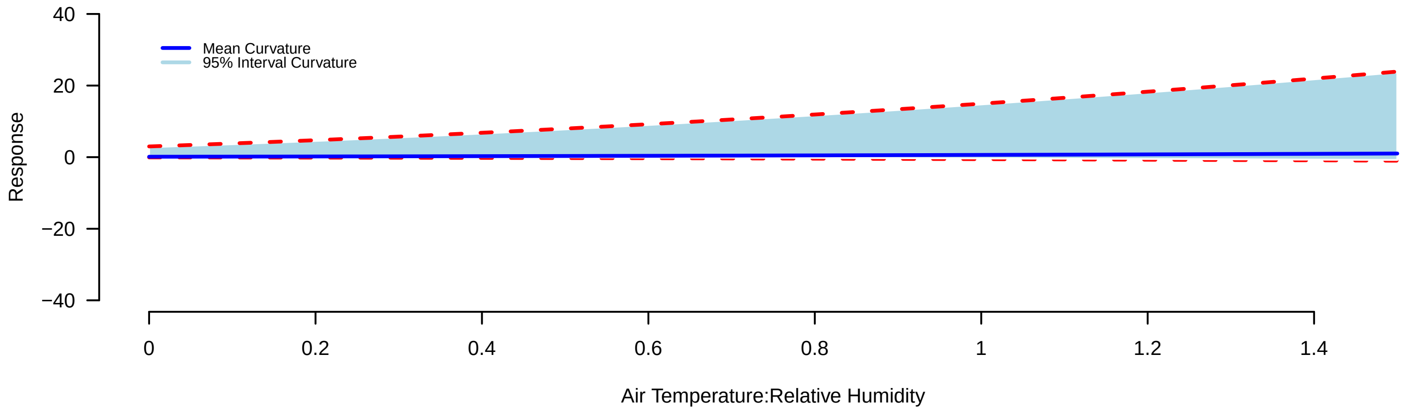

S6, Appendix Figure 537 : Curvature of Air Temperature:Relative Humidity lag 5

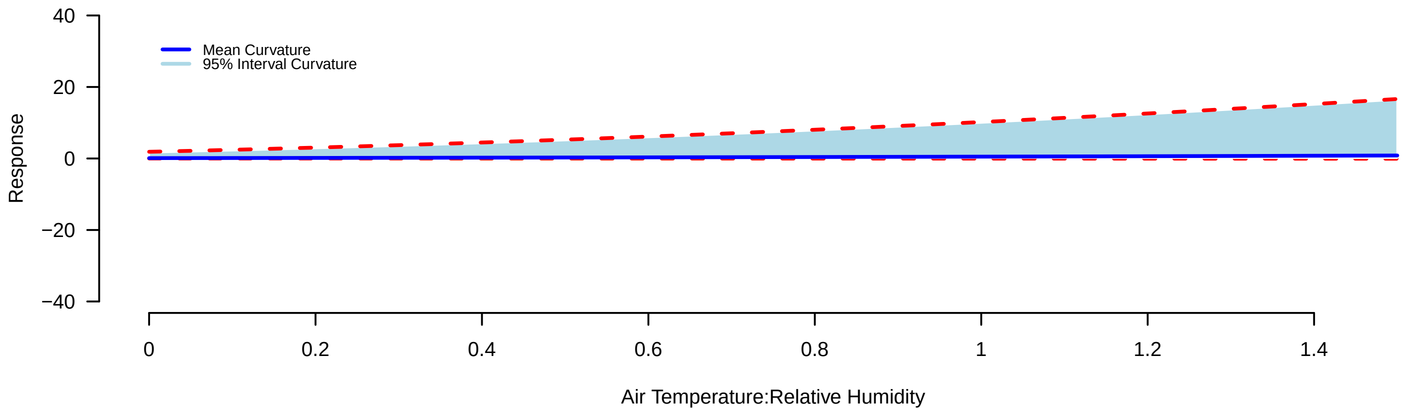

S6, Appendix Figure 538 : Curvature of Air Temperature:Relative Humidity lag 6

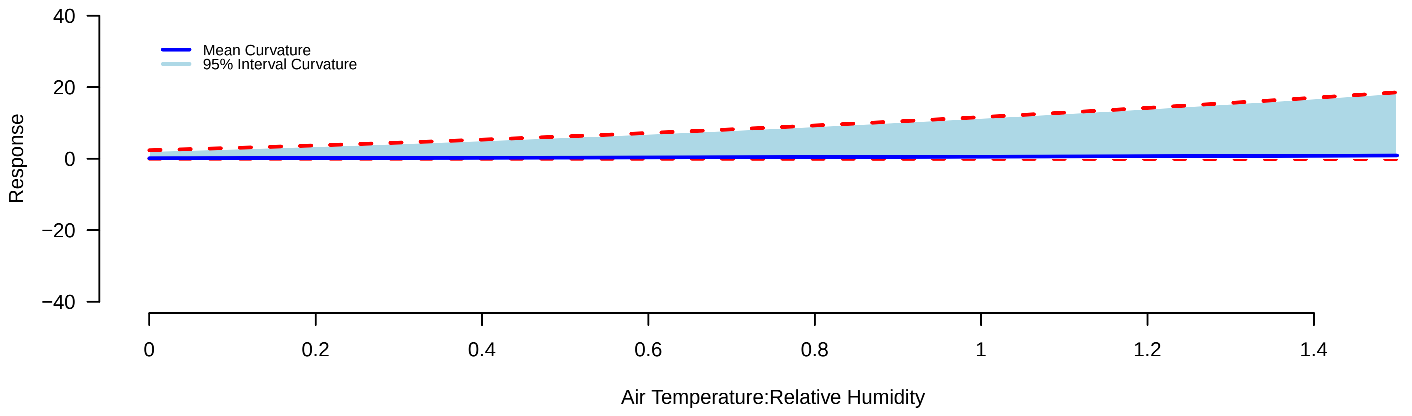

S6, Appendix Figure 539 : Curvature of Air Temperature:Relative Humidity lag 7

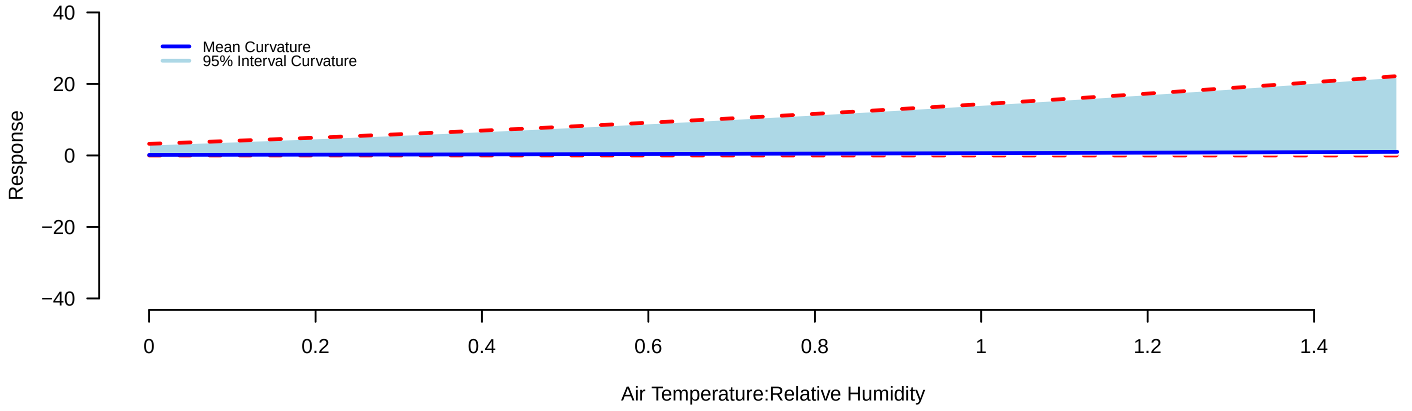

S6, Appendix Figure 540 : Curvature of Air Temperature:Relative Humidity lag 8

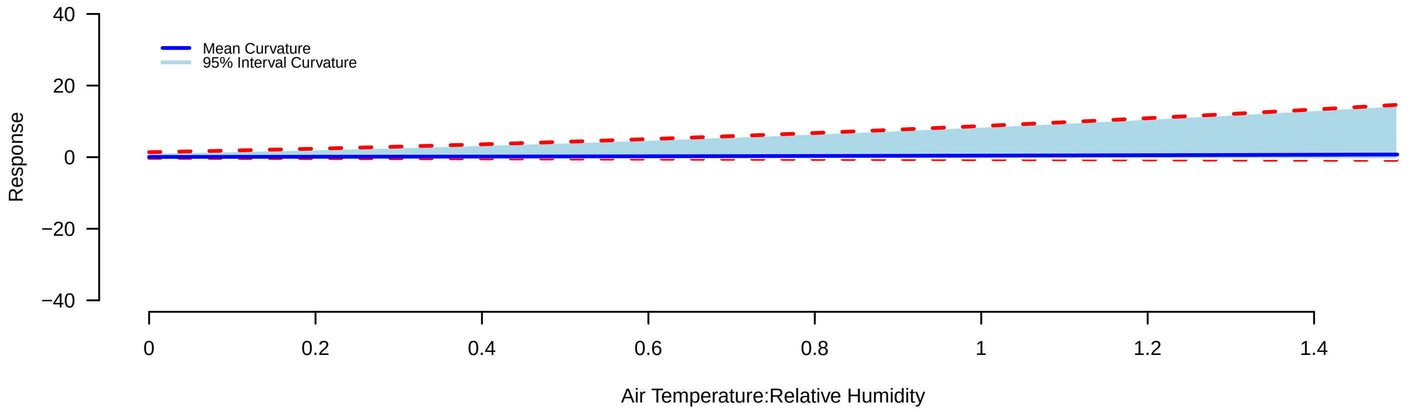

S6, Appendix Figure 541 : Curvature of Air Temperature:Relative Humidity lag 9

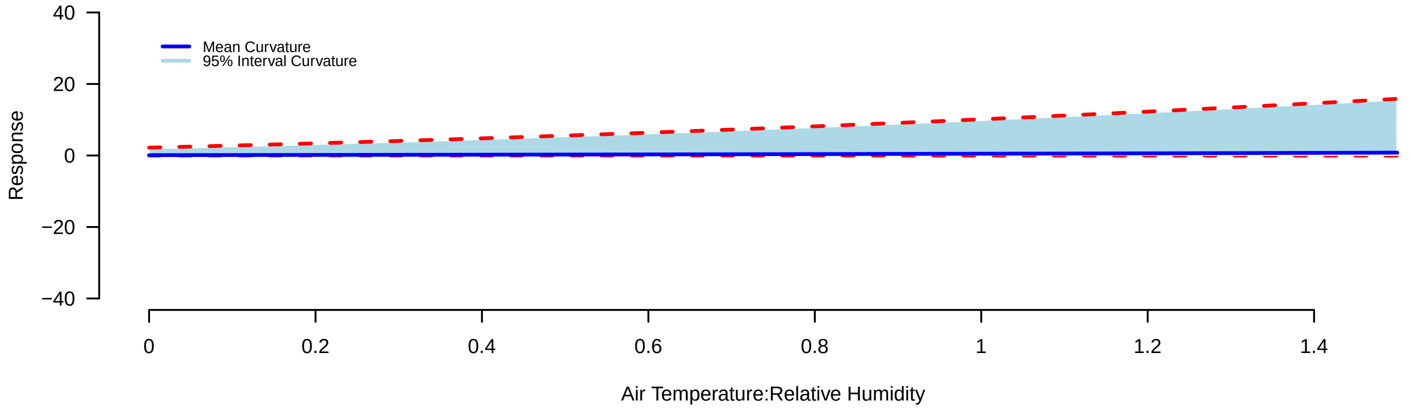

Section 7, Appendix Figure 541 : Bootstrap Inclusion Probability

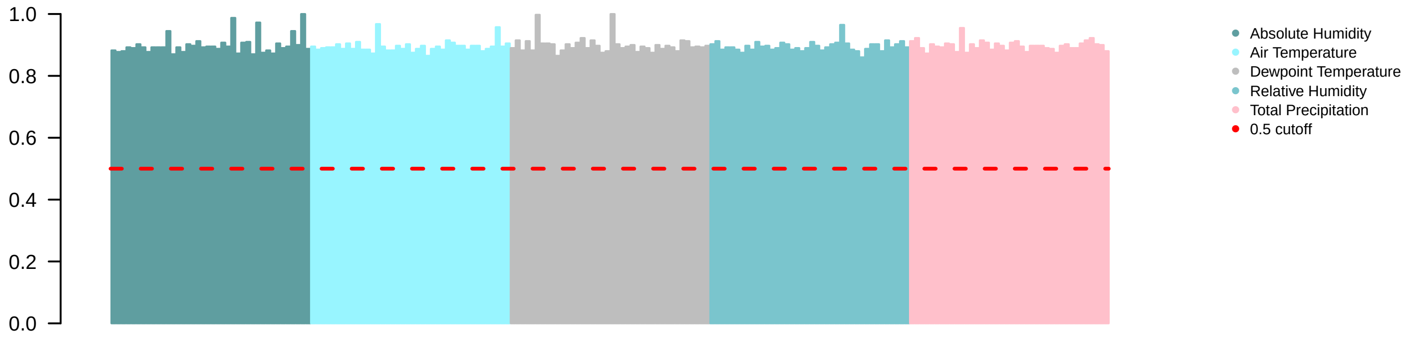

Supplement: S2 Appendix — (PDF) [file pcbi.1007839.s002.pdf]
