# Supplementary material for: Inference on dengue epidemics with Bayesian regime switching models
Source: PLoS Comput Biol. 2020 May 1;16(5):e1007839. doi: 10.1371/journal.pcbi.1007839 (PMC7219790; doi:10.1371/journal.pcbi.1007839)
Supplement: S3 Appendix — (PDF) [file pcbi.1007839.s003.pdf]

Technical Appendix 3

Inference on Dengue epidemics with Bayesian regime switching models

February 8, 2020

1 Stochastic Susceptible-Infected-Recovered Model

In order to provide a mechanistic interpretation of the labelled regimes, in addition to looking at posterior transition probabilities and the data fit to the regimes on actual case count data, we fit BRS to data simulated using a stochastic Susceptible-Infected-Recovered (sSIR) model. The sSIR model was used due to its ability to generate time series of disease case counts [1]. Simulated infected individuals were pre-processed through normalization and differencing, with regime classification conducted by fitting Bayesian regime switching following the same estimation steps conducted for dengue case counts.

Simulating disease case counts over time proceeds by first creating separate epidemic and endemic infection functions with random variables X and Y_Θ with the following parameterization, where w_t, Θ, S_t, I_t represent the seasonality, state (epidemic or endemic), number of susceptible and infected individuals at time t respectively:

$$I_t(w_{t-1}, I_{t-1}, S_{t-1}, \Theta = 1) = X + Y_{\Theta=\text{epidemic}}$$
$$I_t(w_{t-1}, I_{t-1}, S_{t-1}, \Theta = 1) = Y_{\Theta=\text{endemic}}$$

Y_Θ is characterized using a binomial distribution with the number of susceptible S_{t-1} at the previous time point parameterizing the number of draws for $Y_{\Theta=\text{epidemic}}$ and $Y_{\Theta=\text{endemic}}$. The probability of being drawn is dependent on the lagged observed daily total precipitation of Singapore w_{t-1}, to represent seasonal forcing of disease and the ratio of infected and susceptible cases $\frac{I_{t-1}}{S_{t-1}}$ to allow the proportion of infected in the previous period to affect the chance of being infected currently. An additional non-negative sinusoidal term $|\sin(t \times \pi \times A)|$ was added to the probability of being drawn in $Y_{\Theta=\text{epidemic}}$ in order to characterize the rise and fall of case counts in an epidemic period. A, B, C are normalization constants to make the probability of being drawn in $Y_{\Theta=\text{epidemic}}, Y_{\Theta=\text{endemic}}$ to be between 0 to 1.

$$Y_{\Theta=\text{epidemic}} \sim \text{Bin}\left(n = S_{t-1}, p = \frac{1}{C}\exp(\frac{1}{B}w_{t-1} + \frac{I_{t-1}}{S_{t-1}} + \frac{1}{B}|\sin(t \times \pi \times \frac{1}{A})|)\right)$$
$$Y_{\Theta=\text{endemic}} \sim \text{Bin}\left(n = S_{t-1}, p = \frac{1}{C}\exp(\frac{1}{B}w_{t-1} + \frac{I_{t-1}}{S_{t-1}})\right)$$

The additional X term for the epidemic infection function characterizes how infection case counts are more likely to fluctuate within an epidemic period:

$$X \sim \text{Normal}(\mu = 0, \sigma = 25)$$

We denote static and unequal transitions between endemic and epidemic phases of the simulated disease to simulate irregular durations between both phases. Transitions out of the epidemic phase are more probable compared to transitions out of the endemic phase, as an outbreak phase is more likely to end, compared to a non-outbreak phase. Therefore, the following parameters were used to denote transitions to the two phases of transmissions:

$$\text{P}(\Theta_t = \text{Epidemic}|\Theta_{t-1} = \text{Epidemic}) = 0.99, \text{P}(\Theta_t = \text{Endemic}|\Theta_{t-1} = \text{Epidemic}) = 0.01$$
$$\text{P}(\Theta_t = \text{Endemic}|\Theta_{t-1} = \text{Endemic}) = 0.998, \text{P}(\Theta_t = \text{Epidemic}|\Theta_{t-1} = \text{Endemic}) = 0.002$$

Finally, the following sSIR difference equations are iterated forward in time to provide a simulated time series of infected individuals, which takes into account the birth/death dynamics of a population through μ and recovery from infections γ:

$$S_t = S_{t-1} - I_t(w_{t-1}, I_{t-1}, S_{t-1}, \Theta_t) - \mu S_{t-1} + \mu N_{t-1}$$
$$I_t = I_{t-1} + I_t(w_{t-1}, I_{t-1}, S_{t-1}, \Theta_t) - \gamma I_t - \mu I_{t-1}$$
$$R_t = R_{t-1} + \gamma I_t - \mu R_{t-1}$$

To initialize our model, we set the initial values and parameterize the model using the following terms:

Parameter	Value	Justification
N_1	5610000	Population of Singapore [2]
S_1	(5610000-1000)/2	$(N_1 - I_1)/2$
I_1	1000	
R_1	(5610000-1000)/2	$(N_1 - I_1)/2$
w_t	Time varying	Daily total precipitation of Singapore from 2000-2017 [2]
μ	1/(82 x 365)	1/Average life expectancy of Singaporeans [2]
γ	1/4	1/Average recovery rate of dengue [3]
A	200	Normalization Constant
B	10000	Normalization Constant
C	10	Normalization Constant

2 Fitting Bayesian Regime Switching

The simulation was run for a total of 6600 days, corresponding to the length of observed w_t. The first 2000 days were discarded as burnin, with the remaining observations aggregated into weekly observations. Normalization of the simulated data set then follows by subtracting simulations from its minimum value and then divided by the range of simulated values. These simulations were then differenced and placed into the BRS estimation strategy with 1-3 lags, with the final number of lags determined by whether that particular BRS specification could sufficiently account for residual autocorrelation in the simulated dataset. In order to provide mechanistic interpretations of the regimes, we plotted the simulated infection function $I_t(w_{t-1}, I_{t-1}, S_{t-1}, \Theta_t)$ against the estimated regimes across time in the simulated dataset using BRS to look at whether large changes in infection probability characterize these regimes.

References

[1] Fred Brauer. Compartmental models in epidemiology. In *Mathematical epidemiology*, pages 19–79. Springer, 2008.

[2] Department of Statistics Singapore. Yearbook of statistics singapore, 2018, 2018.

[3] World Health Organization et al. Dengue and severe dengue. Technical report, World Health Organization. Regional Office for the Eastern Mediterranean, 2014.
